# Transcriptome Profiling Reveals a Petunia Transcription Factor, *PhCOL4*, Contributing to Antiviral RNA Silencing

**DOI:** 10.3389/fpls.2022.876428

**Published:** 2022-04-14

**Authors:** Yingru Xu, Xiaotong Ji, Zhuangzhuang Xu, Yanping Yuan, Xiling Chen, Derong Kong, Yanlong Zhang, Daoyang Sun

**Affiliations:** ^1^College of Landscape Architecture and Arts, Northwest A&F University, Yangling, China; ^2^National Engineering Technology Research Center for Oil Peony, Northwest A&F University, Yangling, China

**Keywords:** petunia, transcriptome, virus-induced gene silencing, tobacco rattle virus, transcription factor

## Abstract

RNA silencing is a common antiviral mechanism in eukaryotic organisms. However, the transcriptional regulatory mechanism that controls the RNA silencing process remains elusive. Here, we performed high-depth transcriptome analysis on petunia (*Petunia hybrida*) leaves infected with tobacco rattle virus (TRV) strain PPK20. A total of 7,402 differentially expressed genes (DEGs) were identified. Of them, some RNA silencing-related transcripts, such as *RNA-dependent RNA polymerases* (*RDR*s), *Dicer-like RNase III enzymes* (*DCL*s), and *Argonautes* (*AGO*s), were induced by viral attack. Furthermore, we performed TRV-based virus-induced gene silencing (VIGS) assay on 39 DEGs encoding putative transcription factors (TFs), using green fluorescent protein (GFP) and *phytoene desaturase* (*PhPDS*) as reporters. Results showed that the down-regulation of *PhbHLH41*, *PhbHLH93*, *PhZPT4-3*, *PhCOL4*, *PhHSF*-*B3A*, *PhNAC90*, and *PhWRKY75* led to enhanced TRV accumulation and inhibited *PhPDS*-silenced photobleaching phenotype. In contrast, silencing of *PhERF22* repressed virus accumulation and promoted photobleaching development. Thus, these TFs were identified as potential positive and negative regulators of antiviral RNA silencing, respectively. One positive regulator *PhCOL4*, belonging to the B-box zinc finger family, was selected for further functional characterization. Silencing and transient overexpression of *PhCOL4* resulted in decreased and increased expression of several RNA silencing-related genes. DNA affinity purification sequencing analysis revealed that PhCOL4 targeted *PhRDR6* and *PhAGO4*. Dual luciferase and yeast one-hybrid assays determined the binding of PhCOL4 to the *PhRDR6* and *PhAGO4* promoters. Our findings suggest that TRV-GFP-*PhPDS*-based VIGS could be helpful to identify transcriptional regulators of antiviral RNA silencing.

## Introduction

RNA silencing is a highly conserved and sequence-specific RNA degradation mechanism in plants. Under biotic stresses such as virus infection, RNA silencing can function as a natural defense mechanism against foreign nucleic acids introduced by viruses (Wang and Metzlaff, 2005). RNA silencing is triggered by the specific RNAs with double-stranded features (Llave, 2010). The whole process can be divided into three phases: initiation, effector, and amplification (Csorba and Burgyán, 2016). RNA silencing initiates when double-stranded RNA (dsRNA) is recognized by Dicer-like RNase III enzyme (DCL) and is cleaved into small pieces of 21–24-nt small interfering RNAs (siRNAs). The 3′ terminal of siRNA is 2′-*O*-methylated by a methyltransferase HEN1 to maintain its structural stability (Ruiz-Ferrer and Voinnet, 2009). Then, the mature siRNA is incorporated into the RNA-induced silencing complex (RISC), which contains the core argonaute (AGO) protein for targeting and down-regulating homologous mRNA (Liu and Chen, 2016). Next, RNA-dependent RNA polymerase (RDR) uses primary siRNA as a primer to synthesize dsRNA, which is re-processed into secondary siRNAs by DCL enzyme to amplify the silencing signals (Pumplin and Voinnet, 2013). At the end of RNA silencing process, transcription of viral RNAs can be substantially reduced at the molecular level, thereby enhancing host resistance to viral attack.

A set of crucial proteins such as RDRs, DCLs, and AGOs have been known to be implicated in antiviral RNA silencing pathway in plants (Garcia-Ruiz et al., 2010). In the model plant *Arabidopsis*, a total of 6 RDRs, 4 DCLs, and 10 AGOs have been identified and characterized (Eamens et al., 2008). RDR1, RDR2, and RDR6 can resist the infections of various RNA viruses through the generation of secondary siRNAs (Gaffar and Koch, 2019). The *RDR6* mutant displays increased susceptibility to cucumber mosaic virus (CMV) and potato virus X (PVX) in *Nicotiana tabacum* (Li et al., 2012). *RDR1* mutation results in compromised resistance of tobacco plants to tobacco mosaic virus (TMV) (Vaucheret, 2006). Simultaneous suppression of *RDR1*, *RDR2*, and *RDR6* causes a decreased resistance to turnip mosaic virus (TuMV) in *Arabidopsis* (Garcia-Ruiz et al., 2010).

In addition, DCL1 cleaves the imperfect hairpin RNA (hpRNA) into 21-nt miRNA (Henderson et al., 2006), while DCL2, DCL3, and DCL4 process long and nearly perfect dsRNA into 22-nt, 24-nt, and 21-nt siRNAs, respectively (Bologna and Voinnet, 2014). DCL1 has a minor contribution to the cleavage of dsRNA since only a small amount of virus-derived siRNAs are detected in PVX-infected *Arabidopsis dcl2*/*dcl3*/*dcl4* triple mutant (Ruiz-Ferrer and Voinnet, 2009; Jaubert et al., 2011). Instead, DCL1 mainly functions by negatively regulating the transcription of *DCL3* and *DCL4* (Qu et al., 2008). DCL2 and DCL4 exhibit major, redundant, but hierarchical functions in antiviral defense. DCL2 carves out an important role when DCL4 is absent or suppressed. Reduced transcription of *DCL2* and *DCL4* leads to hyper-susceptibility to virus infection (Deleris et al., 2006; Pumplin and Voinnet, 2013). In contrast, DCL3 seems to play a minor role in RNA silencing-induced antiviral defense but a vital role in RNA-directed DNA methylation (Singh et al., 2019).

Most AGO proteins in *Arabidopsis* possess antiviral activities. Among them, AGO1 and AGO2 are the primary effectors against RNA viruses, while AGO4 functions predominately in defending against DNA viruses (Carbonell and Carrington, 2015; Kenesi et al., 2021). AGO1 has been revealed to be involved in antiviral response to turnip crinkle virus (Azevedo et al., 2010) and CMV (Zhang et al., 2006). AGO2 plays an essential role in resisting CMV (Harvey et al., 2011), PVX (Jaubert et al., 2011), and TuMV (Manacorda et al., 2021). AGO1 and AGO7 function cooperatively to ensure the removal of viral RNAs with different secondary structures (Qu et al., 2008). Furthermore, AGO proteins exhibit variable binding properties to siRNAs arising from cleaved dsRNA. AGO4 binds to DCL3-dependent 24-nt siRNA and participates in chromatin modification (Gao et al., 2010). AGO1 interacts with 21-nt and 22-nt small RNAs through different mechanisms. AGO4, AGO6, and AGO9 bind to 24-nt siRNA to act specifically in the DNA methylation pathway (Havecker et al., 2010; Zhu et al., 2011).

Apart from the key enzymes mentioned above, antiviral RNA silencing in plants also requires the function of transcription factors (TFs) which regulate the spatiotemporal expression of the above enzyme-encoding genes. Various TF families, including AP2/ERF (Fischer and Dröge-Laser, 2004), ARF (Zhang et al., 2020), bHLH (Aparicio and Pallás, 2017), bZIP (Herath and Verchot, 2021), HD-ZIP (Zou et al., 2016), HSF (Merkling et al., 2015), MADS (Yang et al., 2014), MYB (Li et al., 2018), NAC (Sun et al., 2019), WRKY (Freeborough et al., 2021), and zinc finger (Sera, 2017), have been demonstrated to participate in the defense response to virus infection. A broad range of biological pathways associated with innate immunity or defensive hormone signaling are potentially controlled by these TFs. However, few studies on TFs involved in RNA silencing-elicited antiviral defense have been reported.

Virus-induced gene silencing (VIGS) is a high-throughput reverse genetic technology, which was developed based on antiviral RNA silencing mechanism (Unver and Budak, 2009). The VIGS system requires the construction of an artificial viral vector, such as tobacco rattle virus (TRV), carrying endogenous gene fragments. The inoculation of viral constructs can cause the degradation of target genes through RNA silencing machinery in plants. In previous studies, we identified an ethylene-responsive element binding factor PhERF2 and an ocs element binding factor PhOBF1, which played critical roles in antiviral RNA silencing in petunia (Sun et al., 2016, 2017). Inclusion of *PhERF2* or *PhOBF1* fragment in the TRV vector containing reporter genes *PhPDS* and *PhCHS* led to impaired leaf photobleaching and white-corollas phenotypes. PhERF2 and PhOBF1 functioned as crucial participants in the defense against TRV, CMV, or TMV infection, and affected the transcription of several *RDR*, *DCL*, and *AGO* genes in the RNA silencing pathway. These results suggested that *PhPDS* or *PhCHS* can be used as an effective reporter gene for VIGS-based RNA silencing studies. A similar approach has been reported in *Arabidopsis*, in which a TRV-*AtPDS* construct was used to assess the antiviral function of *AGO1-27* and *DCL2*/*DCL3*/*DCL4* (Ma et al., 2015). These studies prompt us to identify novel regulators of antiviral RNA silencing using VIGS method. Here, we combined high-depth Illumina RNA sequencing (RNA-Seq) and TRV-GFP-*PhPDS*-based VIGS assays to screen key TFs potentially involved in antiviral RNA silencing in petunia. A number of candidate TFs that can modulate, either positively or negatively, viral resistance through RNA silencing were obtained. In particular, a zinc finger TF, annotated as *PhCOL4*, was validated for its essential role in antiviral RNA silencing.

## Materials and Methods

### Plant Materials and Growth Conditions

The seeds of petunia (*Petunia hybrida*) cultivar ‘Primetime Blue’ were purchased from Goldsmith Seeds Inc. (Gilroy, CA, United States). They were planted in a 72-well plastic tray filled with the soil mix of peat moss, perlite, and vermiculite at 2:1:1 by volume. The tray was placed into a growth chamber at 25/22°C temperature (day/night) with a photoperiod of 16/8 h (light/dark) and a 70% relative humidity. Young seedlings were transplanted to small plastic pots containing the same soil mix for inoculation with wild TRV strain and *Agrobacterium* culture bearing TRV-GFP-*PhPDS* or pCAMBIA2300 constructs. Wild TRV strain-inoculated petunia leaves at three stages were used for Illumina RNA-Seq. The leaves infected with various TRV constructs were used for VIGS assay. The leaves infiltrated with pCAMBIA2300 constructs were used for transient overexpression assay. To examine the abundances of transcripts, the inoculated or uppermost systemically infected leaves were collected for RNA extraction.

### Inoculation of the Wild Type Tobacco Rattle Virus PPK20

Tobacco rattle virus strain PPK20 was preserved in tobacco (*N. tabacum*) cultivar ‘NC82’ plantlets. For infectious sap preparation, the young leaves infected with TRV (PPK20) were harvested and homogenized with a small amount of kieselguhr in 100 mM phosphate buffer at pH 7.0 (1:6, w/v) using a mortar and pestle. The viral preparations were mixed with 10% of 600-mesh carborundum powder by weight, and then mechanically inoculated onto the fully expanded healthy young leaves (Tian and Valkonen, 2013). To ensure sufficient viral propagation, the rub-inoculation process was performed once more within half an hour. The inoculum was flushed with sterile deionized water. The symptom occurrence was observed during the post-inoculation periods. Relative accumulation levels of TRV (RNA1 and RNA2) were determined by real-time quantitative PCR (RT-qPCR) analysis.

### RNA Preparation and Illumina Sequencing

Petunia leaves infected with TRV (PPK20) were collected to extract total RNA using TRIzol reagent (Invitrogen, Carlsbad, CA, United States). Three independent biological replicates were used for each stage. RNA samples were further purified to eliminate contaminant DNA using RNase-free DNase I (Promega, Madison, WI, United States). RNA integrity was examined by observing the major RNA species (*18S* and *28S rRNA*) through 1.5% agarose gel electrophoresis. RNA yield was quantified using a NanoDrop ND-2000c Spectrophotometer (NanoDrop Technologies, Wilmington, DE, United States). Illumina sequencing and generation of transcriptome data were conducted at Gene *Denovo* Bio-Technology Co., LTD. (Guangzhou, China). In brief, the general experimental procedure includes mRNA enrichment and fragmentation, cDNA synthesis, adaptor ligation, and ligated products sequencing as previously described (Sun et al., 2019).

### *De novo* Assembly of Transcripts

High-quality clean reads were produced by removing adaptor, low-quality, and unknown nucleotides of raw reads through a Perl script tool. The resulting clean reads were assembled via Trinity program (v2.1.1) (Grabherr et al., 2011). The redundancy of assembled products was removed to obtain unique fragments using iAssembler software (Zheng et al., 2011). The fragments over 200 bp were enriched using TIGR Gene Indices clustering tools (TGICL, v2.1) (Pertea et al., 2003), and regarded as unigenes. The unigenes were mapped to the reference genome^1^ of *P. axillaris*, which is one of *P. hybrida*’s parents. Novel transcripts were determined using Cuffcompare software through a threshold for length ≥ 200 bp and exon number ≥ 2 (Trapnell et al., 2012), and annotated against the public protein databases including NR, Swiss-Prot, COG, and KEGG.

### Differentially Expressed Gene Analysis

Read counts of unigenes were calculated and standardized to the fragments per kilobase of transcript per million mapped reads (FPKM), which were used to quantify the expression levels of unigenes through Cufflinks program (v2.1.1) (Trapnell et al., 2010). Differentially expressed genes (DEGs) were identified using edgeR package (v3.12) (Chen et al., 2014). Statistical significance of DEGs was evaluated with a cutoff of fold change (FC) ≥ 2.0 and false discovery rate (FDR) ≤ 0.05. Blast2GO tool (v2.3.5) and WEGO program^2^ were used to gain knowledge about Gene Ontology (GO) classification of DEGs (Conesa et al., 2005; Ye et al., 2006). To assign putative metabolic processes to DEGs, the pathway enrichment analysis was carried out using a BLAST search against the Kyoto Encyclopedia of Genes and Genomes (KEGG) database^3^ (Wixon and Kell, 2000). *P*-values were calculated using a previously reported method and subjected to FDR correction (Lu et al., 2012). Significantly enriched pathways were characterized using FDR ≤ 0.05 as a cutoff.

### Measurement of Endogenous Hormones

Petunia leaves inoculated with TRV (PPK20) at different time points were harvested. For ethylene (ET) detection, the leaves were placed in a 50-ml plastic tube at 25°C for 4 h, with an airproof rubber plug embedded into the cap. The parafilm was used to seal the connection gaps. A 3-ml gas sample was withdrawn from the tube using a gas-tight syringe with a needle, and applied to a gas chromatograph (GC-8A; Shimadzu, Kyoto, Japan) for measurement of ET release. The examination of other hormones, including abscisic acid (ABA), jasmonic acid (JA), and salicylic acid (SA), was performed as previously described (Mornya and Cheng, 2011) with some minor modification. Briefly, petunia samples were extracted in 80% methanol and 1 mM butylated hydroxytoluence on a rotary shaker at 4°C for 1 h. After a complete homogenization, the mixture was centrifuged at 12,000 rpm for 20 min at 4°C. The supernatant was collected and purified through a 0.45 μm filter. It was concentrated to near dryness in N_2_ gas and then dissolved in 50% methanol. The resulting solution was analyzed by a high performance liquid chromatography-electrospray ionization tandem mass spectrometry (HPLC-ESI-MS/MS). All chemical substances purchased from Sigma-Aldrich (St Louis, MO, United States) were used as the reference standards.

### Real-Time Quantitative PCR

Total RNA from petunia leaves was extracted as mentioned above. After purification and quantification, about 2–5 μg of RNA samples were used to synthesize first-strand cDNA with SuperScript III reverse transcriptase (Invitrogen, Carlsbad, CA, United States) according to the manufacturer’s recommendations. The resulting cDNA products were fivefold diluted by ddH_2_O and restored at –20°C until further use. The specific primer pairs were designed based on the known cDNA sequences of genes tested via Primer Premier5^4^ (Supplementary Table S1). *26S ribosomal RNA* served as a reference gene. RT-qPCR assay was performed using the SYBR Green PCR Master Mix (2×) (Applied Biosystems, Foster City, CA, United Stataes) in a LightCycler480 Real-Time PCR System (Roche Diagnostic, Basel, Switzerland). The experiment was repeated three times using independent RNA samples. Relative expression levels of genes were analyzed using a previously described protocol (Livak and Schmittgen, 2001).

### Virus-Induced Gene Silencing Assay

The TRV-GFP construct was generated as previously described (Tian et al., 2014). A 138-bp fragment of *PhPDS* cDNA was amplified using the forward and reverse primers with a *Sac*I and an *Xho*I restriction site, respectively. It was ligated into the corresponding sites of TRV-GFP plasmid to form the TRV-GFP-*PhPDS* construct. Thirty-nine selected candidate TF genes were cloned into the TRV-GFP-*PhPDS* construct between *Bam*HI and *Kpn*I sites using the Seamless Cloning and Assembly Kit (Novoprotein, Shanghai, China). Oligonucleotide primers used for gene fragment amplification and expression analysis are listed in Supplementary Table S1. The resulting TRV plasmids were electrotransformed into *A. tumefaciens* strain GV3101. The agro-inoculation was performed according to a previously described protocol (Sun et al., 2017). Three-week-old seedlings of petunia were used for the agro-inoculation. For expression assessment of *PhPDS* and TF genes, the sequences beyond the inserted fragments were used for primer design (Supplementary Table S1). For GFP fluorescence detection, fluorescent foci were visualized using a portable blue light-emitting diode flashlight as an excitation source at 450 nm (LUYOR-3260RB, LUYOR, Shanghai, China). The images were taken using a digital camera (EOS 20D, Canon, Tokyo, Japan) with a bypass emission filter (LUV-495, LUYOR, Shanghai, China). The intensity of fluorescence was quantified using ImageJ software (NIH, Bethesda, MD, United States).

### Transient Overexpression of *PhCOL4*

*Agrobacterium*-mediated transient assay was performed as previously described (Mußmann et al., 2011) with some minor changes. The complete coding region of *PhCOL4* (Supplementary Figure S1) was cloned into pCAMBIA2300 vector between *Eco*RI and *Sac*I sites. The resulting plasmids were transformed into *A. tumefaciens* strain LBA4404. The transformed bacteria were cultured in liquid LB media supplemented with 40 mg⋅L^–1^ kanamycin for overnight at 28°C. The *Agrobacterium* cells were harvested and resuspended to an OD600 of 4.0 in the infiltration buffer containing 10 mM MgCl_2_, 10 mM MES, and 200 μM acetosyringone. The suspension was infiltrated into fully developed petunia leaves through a syringe. At 6 days post infiltration, the leaves were collected for gene expression analysis.

### DNA Affinity Purification Sequencing Assay

DAP-Seq binding experiment was carried out according to a previously described method (Yao et al., 2020). In brief, genomic DNA (gDNA) was extracted from petunia leaves using CTAB method, and fragmented through a Covaris M220 (Woburn, MA, United States). Purification of fragmented gDNA was performed using AMPure XP beads (Beckman Coulter, Indianapolis, IN, United States). DNA library was prepared using the NEXTFLEX Rapid DNA-Seq Kit (PerkinElmer, Austin, TX, United States). The coding sequence of *PhCOL4* was introduced into pFN19K HaloTag T7 SP6 Flexi expression vector. The TNT SP6 Coupled Wheat Germ Extract System (Promega, Madison, WI, United States) was used to express the Halo-PhCOL4 fusion proteins, which were then captured by Magne Halo Tag Beads (Promega, Madison, WI, United States). The proteins bound to beads were incubated with gDNA fragments at 25°C for 2 h. A high temperature of 98°C for 10 min was used to denature the proteins and release the bound gDNA fragments. The fragments were PCR-amplified, purified, and sequenced. For data processing, all reads were mapped to the reference genome of *P. axillaris*^5^. Peak analysis was conducted using Macs2 tool. Distribution of peaks in petunia genome sequence was analyzed using Homer (Heinz et al., 2010). Functional annotation of corresponding genes was determined through a blast against NR, NT, Swissprot, and Pfam databases. Protein-bound motifs were predicted using MEME-Chip suite 5.0.5 (Machanick and Bailey, 2011).

### Dual Luciferase Assay

Dual luciferase assay was performed using a method as previously described (Sun et al., 2019). The coding sequence of *PhCOL4* was ligated into pGreenII 62-SK vector, which was regarded as the effector. To construct the reporters, the promoter sequences of *PhRDR6* and *PhAGO4* (Supplementary Figure S1) were amplified using the primers listed in Supplementary Table S1, and cloned into pGreenII 0800-LUC vector expressing firefly luciferase (LUC) and *Renilla* luciferase (REN). The resulting plasmids were transformed into *A. tumefaciens* strain GV3101. Co-infiltration with effector and reporter constructs was carried out on petunia young leaves. LUC and REN activities were measured using a Tecan Infinite M200 luminometer (Männedorf, Switzerland). The data were expressed as a ratio of LUC to REN.

### Yeast One-Hybrid Assay

The 35-bp fragments containing predicted PhCOL4-bound motifs in the *PhRDR6* and *PhAGO4* promoters were amplified using the primers (Supplementary Table S1). The fragments were ligated into pHIS2 vector to generate the baits. The full-length open reading frame region of *PhCOL4* was fused to the GAL4 activation domain in pGADT7-Rec vector as the pray. All constructs were co-transformed into yeast strain Y187, which was grown and spotted on SD-*Ura-His-Leu* medium supplemented with 100 mM 3-aminotriazole (3-AT) at different dilutions.

## Results

### Accumulation of Tobacco Rattle Virus PPK20 in Petunia

**FIGURE 1:**
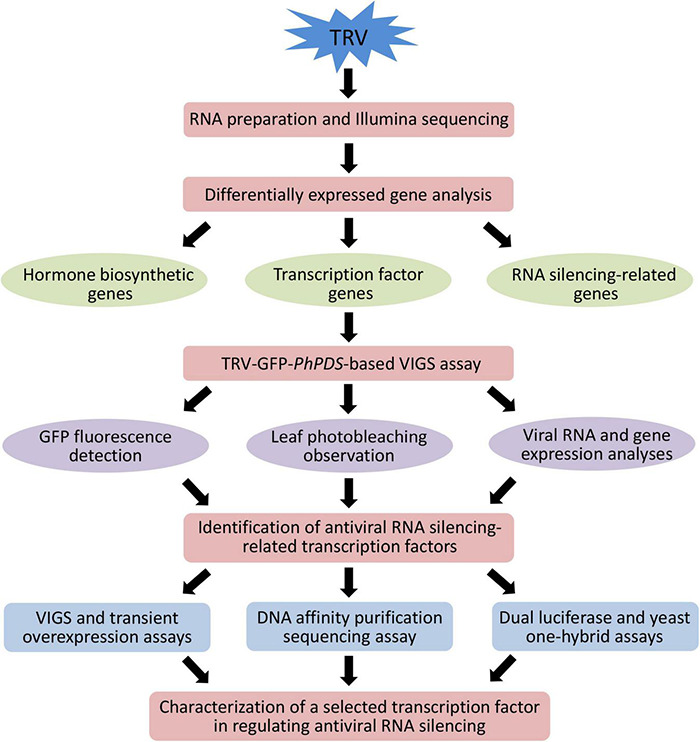
Schematic representation of the experimental strategy to identify important transcription factors involved in antiviral RNA silencing in petunia.

**FIGURE 2:**
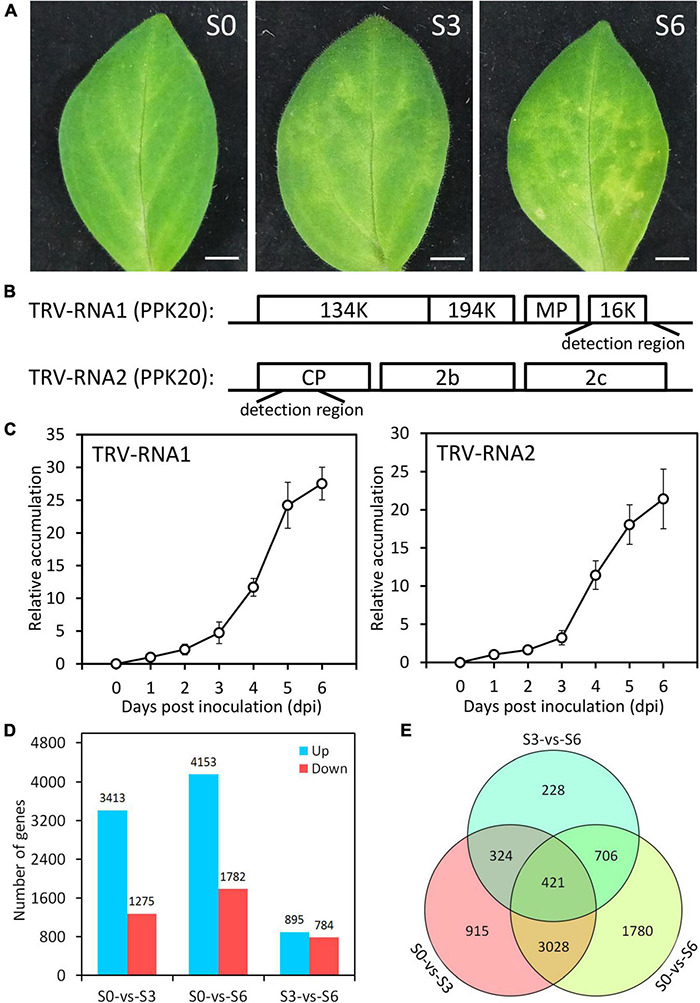
Symptom development and transcriptome data evaluation in TRV PPk20-infected petunia leaves. **(A)** Representative phenotypes of petunia leaves at 0 (S0), 3 (S3), and 6 (S6) days post inoculation with TRV (PPK20). Scale bars = 5.0 mm. **(B)** Schematic diagram of the regions used for detection of TRV-RNA1 and TRV-RNA2 levels. **(C)** Relative accumulation levels of TRV-RNA1 and TRV-RNA2 in TRV (PPK20)-inoculated petunia leaves at intervals. Error bars represent standard error of the mean from three biological replicates. Transcript abundances were standardized to *26S rRNA*. **(D)** Number of up-regulated and down-regulated unigenes in TRV (PPK20)-infected petunia leaves for the pairwise comparisons of S0-vs.-S3, S0-vs.-S6, and S3-vs.-S6. **(E)** Venn diagram of unique and shared differentially expressed unigenes for different pairwise comparisons.

To illustrate transcriptional regulatory mechanism of antiviral RNA silencing in petunia, we used an integrative strategy of TRV (PPK20)-infected Illumina RNA-seq and TRV-GFP-*PhPDS*-based VIGS to identify the TFs regulating the RNA silencing machinery. An important candidate TF was selected for further functional characterization. VIGS and transient overexpression assays were performed to analyze the impact of the identified TF on the expression of RNA silencing components. The target genes downstream of the TF were determined through DAP-Seq, dual luciferase, and yeast one-hybrid assays (Figure 1). Young leaves of *P. hybrida* at six-leaf-stage were inoculated with TRV (PPK20). A clear symptom development was observed in the inoculated leaves, which exhibited slight leaf yellowing at 3 days post inoculation (dpi) and severe leaf mottling or chlorosis at 6 dpi (Figure 2A). Two regions from TRV (PPK20) genomic RNA1 and RNA2 were chosen for quantification of virus RNA levels (Figure 2B). Virus accumulation analysis showed that virus RNA levels of both TRV-RNA1 and TRV-RNA2 increased gradually through 3 dpi and escalated sharply from 4 to 5 dpi. Then, the levels of TRV RNA transcripts continued to increase but at a much lower speed from 5 to 6 dpi (Figure 2C). Based on the trend of symptom development and viral growth, the infection process of TRV (PPK20) in petunia leaves was divided into three stages: 0 dpi (S0), 3 dpi (S3), and 6 dpi (S6), which were selected for transcriptome analysis.

### Illumina RNA Sequencing and *de novo* Assembly

After data filtration, a total of 141,740,216, 118,866,564, and 117,466,686 clean RNA sequencing reads were obtained for petunia leaves at S0, S3, and S6, respectively. At each stage, 74.2% (105,214,806), 73.1% (86,903,750), and 73.3% (86,084,852) of these reads could be mapped to petunia reference genome. *De novo* assembly led to the generation of 34,486 unigenes, including 32,928 mapped and 1,558 novel genes (Table 1). The functions of 24,462 mapped unigenes were annotated based on protein databases (Table 1 and Supplementary Table S2). The sequences of all novel transcripts are shown in Supplementary Figure S2.

**TABLE 1:** Summary of TRV PPK20-infected petunia transcriptome sequencing dataset.

Items	Number
Raw reads at S0 (samples 1–3)	145,011,112
Raw reads at S3 (samples 1–3)	121,488,068
Raw reads at S6 (samples 1–3)	120,055,408
Clean reads at S0 (samples 1–3)	141,740,216
Clean reads at S3 (samples 1–3)	118,866,564
Clean reads at S6 (samples 1–3)	117,466,686
Mapped reads at S0 (samples 1–3)	105,214,806
Mapped reads at S3 (samples 1–3)	86,903,750
Mapped reads at S6 (samples 1–3)	86,084,852
Total generated unigenes	34,486
Mapped reference unigenes	32,928
Annotated reference unigenes	24,462
Novel unigenes	1,558

### Detection of Differentially Expressed Genes During Tobacco Rattle Virus PPK20 Infection

Compared to S0, S3 displayed 3,413 up-regulated and 1,275 down-regulated unigenes. A large number of DEGs were detected for S6, which has 4,153 up-regulated and 1,782 down-regulated unigenes. When compared to S3, S6 only has a moderate number of DEGs: 895 up-regulated and 784 down-regulated (Figure 2D and Supplementary Tables S3–S5). The DEGs (7,402 in total) from pairwise comparison among S0, S3, and S6 were shown in Figure 2E. The highest number (3,449) of overlapped DEGs was observed between S0-S3 and S0-S6. Consistently, less shared DEGs (745) were found between S0-S3 and S3-S6, and 1,127 overlapped DEGs between S0–S6 and S3–S6. These data suggest that, compared to the control S0, the infected S3 and S6 have relatively closer transcriptome profiles with each other.

Gene Ontology (GO) enrichment analysis was carried out to investigate the functions of identified DEGs. At the molecular function level, the most significantly enriched GO terms correspond to “metabolic process,” “binding,” and “membrane” (Supplementary Figure S3A). At the biochemical pathway level, 1,581 DEGs for S0-S3, 2,007 DEGs for S0-S6, and 428 DEGs for S3-S6 were categorized into 126, 127, and 101 biochemical pathways, respectively. Among these pathways, “protein processing in endoplasmic reticulum,” “Plant–pathogen interaction,” and “Plant hormone signal transduction” were significantly enriched (Supplementary Figure S3B and Supplementary Table S6).

### Induction of Endogenous Hormones and Their Biosynthetic Genes by Tobacco Rattle Virus PPK20 Infection

**FIGURE 3:**
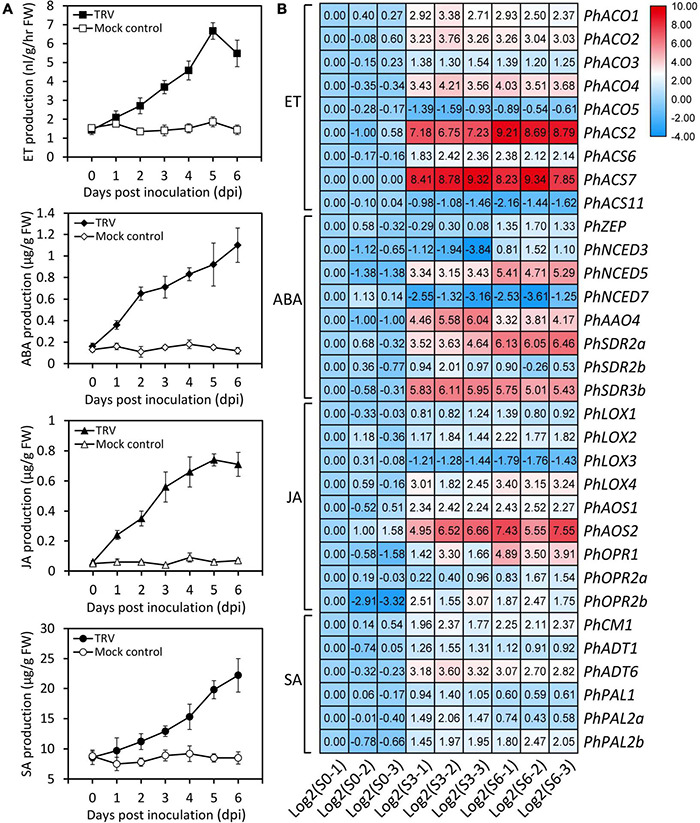
Increased accumulation of several endogenous hormones and transcription of hormone biosynthetic genes by TRV PPK20 infection. **(A)** Accumulation levels of ethylene (ET), abscisic acid (ABA), jasmonic acid (JA), and salicylic acid (SA) in petunia leaves at various days post inoculation with TRV (PPK20). Error bars represent standard error of the mean from three biological replicates. **(B)** Expression levels of ET, ABA, JA, and SA biosynthesis-related genes in petunia leaves at three stages of TRV (PPK20) infection based on transcriptome data. The FPKMs of unigenes at the stage of S0-1 are normalized to 1.00, and transcript levels of all unigenes are transformed with Log2.

Plant hormones are known to play an important role in defense against viruses. “Plant hormone signal transduction” was detected as significantly enriched pathway in our identified DEGs after TRV (PPK20) infection. Therefore, we measured the levels of common plant hormones ET, ABA, JA, and SA during the TRV (PPK20)-infection process. Results showed that the accumulation of these hormones was significantly boosted by TRV (PPK20) infection. The highest levels of ET and JA in petunia leaves were found at 5 dpi, while ABA and SA contents peaked at 6 dpi (Figure 3A). These results are consistent with our transcriptome data, which showed that ET biosynthetic genes, including *PhACO1*, *PhACO2*, *PhACO3*, *PhACO4*, *PhASC2*, *PhACS6*, and *PhACS7*, were significantly up-regulated during TRV (PPK20) infection. The transcription of *PhZEP*, *PhNCED3*, *PhNCED5*, *PhAAO4*, *PhSDR2a*, *PhSDR2b*, and *PhSDR3b* rather than *PhNCED7* in the ABA biosynthesis pathway was induced by TRV (PPK20) infection. In addition, most genes involved in JA production exhibited elevated expression except *PhLOX3*. Several SA biosynthetic genes, such as *PhCM1*, *PhADT*s (1 and 6), and *PhPAL*s (1, 2a, and 2b), were also up-regulated upon TRV (PPK20) infection (Figure 3B).

### Identification of Antiviral RNA Silencing-Related Structural Genes and Transcriptional Regulators

**FIGURE 4:**
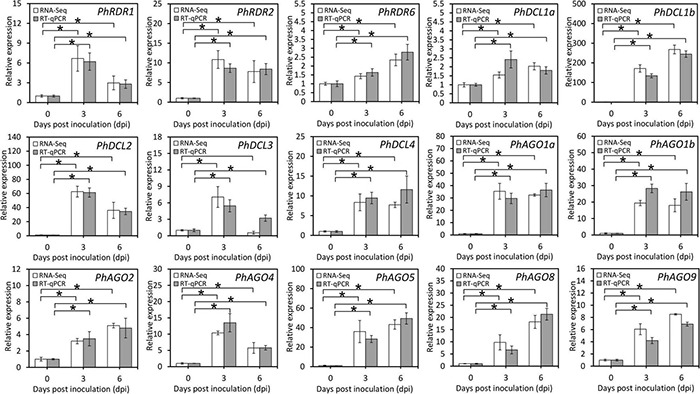
Expression of transcripts involved in the RNA silencing pathway in TRV PPK20-infected petunia leaves. RNA-Seq and RT-qPCR analysis of transcript levels of RNA silencing-associated structural unigenes, including some *RDR*s, *DCL*s, and *AGO*s, in petunia leaves at different phases of TRV (PPK20) infection. *26S rRNA* was used as a reference gene. Error bars represent standard error of the mean from three biological replicates. Statistical significance was determined using Student’s *t*-test (*P* < 0.05) and shown as asterisks.

Considering the essential roles of RDR, DCL, and AGO in antiviral RNA silencing, their transcripts differentially expressed during TRV (PPK20) infection were selected from the transcriptome data. Results showed that *PhRDR1*, *PhRDR2*, *PhRDR6*, *PhDCL1a*, *PhDCL1b*, *PhDCL2*, *PhDCL3*, *PhDCL4*, *PhAGO1a*, *PhAGO1b*, *PhAGO2*, *PhAGO4*, *PhAGO5*, *PhAGO8*, and *PhAGO9* were dramatically up-regulated in TRV (PPK20)-infected petunia leaves. The highest transcriptional changes correspond to *PhDCL1b* whose expression rose by 130–260 times, followed by a 34–62 times increase in *PhDCL2* transcription. In addition, the transcription of *PhDCL1a* and *PhRDR6* increased moderately by less than three times (Figure 4).

**FIGURE 5:**
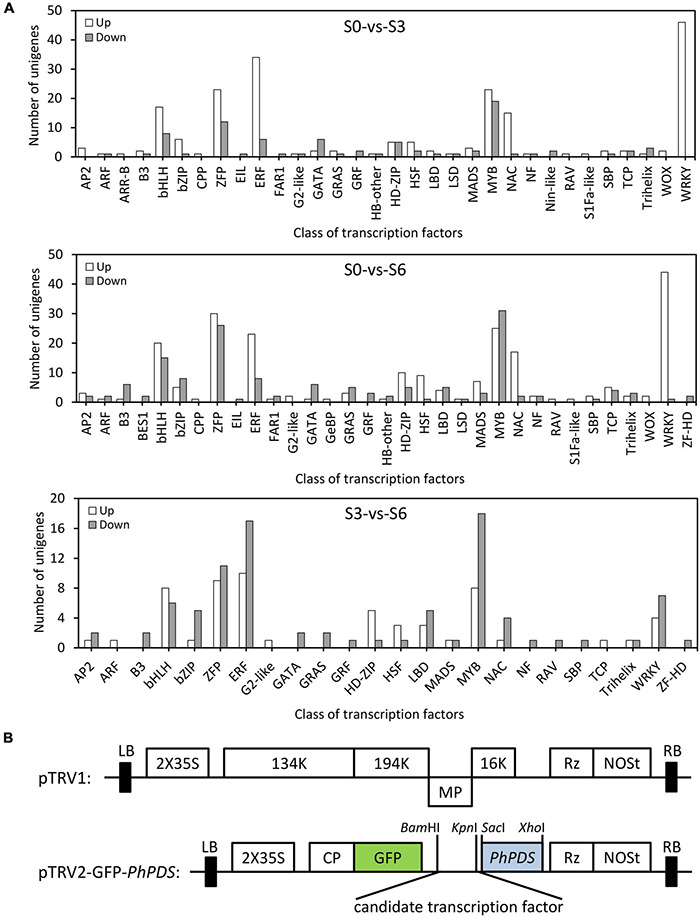
Distribution of differentially expressed transcription factors in petunia leaves upon TRV PPK20 infection. **(A)** Number of up-regulated (up) and down-regulated (down) transcription factors in TRV (PPK20)-inoculated petunia leaves for the pairwise comparisons of S0-vs.-S3, S0-vs.-S6, and S3-vs.-S6. **(B)** Schematic diagram of the TRV-GFP-*PhPDS* construct carrying the fragment of candidate transcription factor.

To dissect the transcriptional regulatory mechanism for TRV defense in petunia, 469 DEGs encoding putative TFs were identified (Supplementary Tables S7–S9). Compared to S0, there are 286 (204 up-regulated and 82 down-regulated) TFs belonging to 32 families that were differentially expressed at S3. When S6 and S0 were compared, 374 (225 up-regulated and 149 down-regulated) TF-encoding (33 families) DEGs were identified. For S3 and S6, 148 (58 up-regulated and 90 down-regulated) TF-encoding DEGs from 24 families were found. The highest number of TFs included those from bHLH, bZIP, ZFP, ERF, MYB, NAC, and WRKY families (Figure 5A).

### Screening of Antiviral RNA Silencing-Associated Transcription Factors Through Virus-Induced Gene Silencing

**FIGURE 6:**
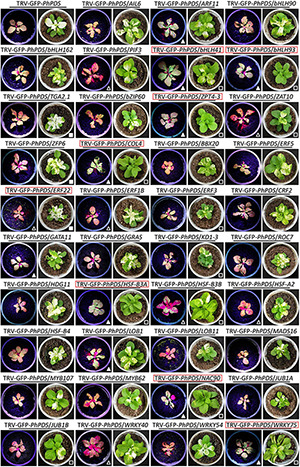
Development of GFP fluorescence and photobleaching phenotype in petunia leaves with VIGS silencing of various transcription factors. Representative GFP-expressed fluorescence signals and *PhPDS*-silenced photobleaching phenotypes in petunia leaves inoculated with *Agrobacterium* containing 39 transcription factors (TFs)-inserted TRV-GFP-*PhPDS* constructs. Three-week-old seedlings of petunia were used for the agro-inoculation. Photographs were taken at 4 and 14 days post inoculation for fluorescence and photobleaching phenotypes. The TRV-GFP-*PhPDS* construct containing no TF insert was used as the control. In comparison with the control, enhanced and suppressed GFP fluorescence signals are highlighted by solid and hollow triangles, while improved and impaired leaf photobleaching phenotypes are denoted by corresponding squares, respectively. The predicted TFs regulating antiviral RNA silencing are marked by red squares.

To identify critical transcriptional regulators involved in antiviral RNA silencing, 39 TRV-induced TFs were selected for TRV-GFP-*PhPDS*-based VIGS assay (Table 2). The fragments of these TFs were cloned into the multiple cloning sites of TRV-GFP-*PhPDS* vector to generate the recombinant constructs (Figure 5B). GFP and *PhPDS* were used as reporter genes for virus accumulation and gene silencing. Normal green fluorescence and leaf photobleaching were observed due to GFP expression and *PhPDS* silencing. Compared to TRV-GFP-*PhPDS* control, the inoculation with TRV-GFP-*PhPDS* constructs containing the fragments of *PhARF11*, *PhbHLH41*, *PhbHLH93*, *PhZPT4*-*3*, *PhCOL4*, *PhGATA11*, *PhHSF-B3A*, *PhLOB1*, *PhNAC90*, and *PhWRKY75* resulted in enhanced fluorescent signals at 4 dpi. In contrast, the fluorescent brightness was suppressed in the leaves infected with TRV-GFP-*PhPDS* constructs containing *PhPIF3*, *PhZAT10*, *PhERF22*, *PhERF1B*, *PhHSF-B3B*, *PhHSF-A2*, and *PhWRKY40* inserts (Figure 6). In addition, TRV-GFP-*PhPDS*/*TGA2.1*-, *ZFP6*-, and *ERF22*-infected systemic leaves showed promoted leaf photobleaching at 14 dpi, in comparison with the control. However, the plants inoculated with TRV-GFP-*PhPDS* vectors containing *PhbHLH41*, *PhbHLH93*, *PhZPT4-3*, *PhZAT10*, *PhCOL4*, *PhBBX20*, *PhERF3*, *PhKD1*-*3*, *PhHSF-B3A*, *PhHSF-B3B*, *PhNAC90*, *PhJUB1B*, and *PhWRKY75* fragments displayed impaired photobleaching phenotypes (Figure 6).

**TABLE 2:** Selected candidate transcription factors used for screening antiviral RNA silencing-related genes.

Gene ID	Annotation	Family	FPKM		
			S0	S3	S6
Peaxi162Scf01142g00028	*PhAIL6*	AP2	0.090	0.477	0.723
Peaxi162Scf00747g00018	*PhARF11*	ARF	0.553	15.250	9.473
Peaxi162Scf00710g00416	*PhbHLH90*	bHLH	0.017	0.267	0.887
Peaxi162Scf00017g02837	*PhbHLH162*	bHLH	0.033	1.050	0.147
Peaxi162Scf00201g00004	*PhPIF3*	bHLH	0.473	4.860	5.447
Peaxi162Scf00498g00042	*PhbHLH41*	bHLH	0.043	3.733	8.593
Peaxi162Scf01190g00006	*PhbHLH93*	bHLH	0.223	1.877	2.203
Peaxi162Scf00076g00531	*PhTGA2.1*	bZIP	0.690	6.707	7.357
Peaxi162Scf00385g00068	*PhbZIP60*	bZIP	8.607	18.870	24.847
Peaxi162Scf00026g00203	*PhZPT4-3*	C2H2-ZFP	0.017	0.373	0.547
Peaxi162Scf00016g00288	*PhZAT10*	C2H2-ZFP	0.517	11.867	5.960
Peaxi162Scf00753g00021	*PhZFP6*	C2H2-ZFP	0.073	2.513	1.773
Peaxi162Scf00045g01824	*PhCOL4*	CO-ZFP	0.040	0.867	0.537
Peaxi162Scf00016g00238	*PhBBX20*	DBB-ZFP	0.250	3.607	1.627
Peaxi162Scf00271g00062	*PhERF5*	ERF	0.630	2.617	0.850
Peaxi162Scf00078g00043	*PhERF22*	ERF	0.097	0.947	0.217
Peaxi162Scf00424g00042	*PhERF1B*	ERF	0.610	3.660	1.563
Peaxi162Scf01294g00031	*PhERF3*	ERF	0.040	0.333	0.663
Peaxi162Scf00390g10026	*PhCRF2*	ERF	8.487	97.417	34.483
Peaxi162Scf01286g00002	*PhGATA11*	GATA	3.360	22.087	11.230
Peaxi162Scf00520g00001	*PhGRAS*	GRAS	11.923	41.087	26.383
Peaxi162Scf01184g00135	*PhKD1-3*	HB-other	0.427	1.550	2.460
Peaxi162Scf00040g00326	*PhROC7*	HD-ZIP	0.797	2.073	2.107
Peaxi162Scf00063g02324	*PhHDG11*	HD-ZIP	0.300	2.317	1.350
Peaxi162Scf00002g02718	*PhHSF-B3A*	HSF	0.573	15.307	18.957
Peaxi162Scf00130g00326	*PhHSF-B3B*	HSF	0.187	21.843	30.043
Peaxi162Scf00327g00413	*PhHSF-A2*	HSF	3.277	4.723	24.003
Peaxi162Scf00450g00422	*PhHSF-B4*	HSF	6.753	27.653	27.030
Peaxi162Scf00389g00071	*PhLOB1*	LBD	0.970	10.050	21.957
Peaxi162Scf00809g00423	*PhLOB11*	LBD	0.610	1.897	3.957
Peaxi162Scf00083g01812	*PhMADS16*	MIKC_MADS	3.010	5.293	12.163
Peaxi162Scf00006g00098	*PhMYB107*	MYB	0.077	6.717	5.163
Peaxi162Scf00015g01036	*PhMYB62*	MYB	0.500	8.900	3.690
Peaxi162Scf00069g00510	*PhNAC90*	NAC	0.037	2.867	5.437
Peaxi162Scf00359g00815	*PhJUB1A*	NAC	0.193	14.713	27.340
Peaxi162Scf00452g00720	*PhJUB1B*	NAC	0.057	7.803	38.757
Peaxi162Scf00014g02915	*PhWRKY40*	WRKY	0.343	64.776	33.970
Peaxi162Scf00074g01125	*PhWRKY54*	WRKY	0.947	29.560	25.803
Peaxi162Scf00023g02225	*PhWRKY75*	WRKY	3.567	48.270	74.820

### Assessment of Viral Multiplication and Silencing Efficiency in Virus-Induced Gene Silencing Experiments

**FIGURE 7:**
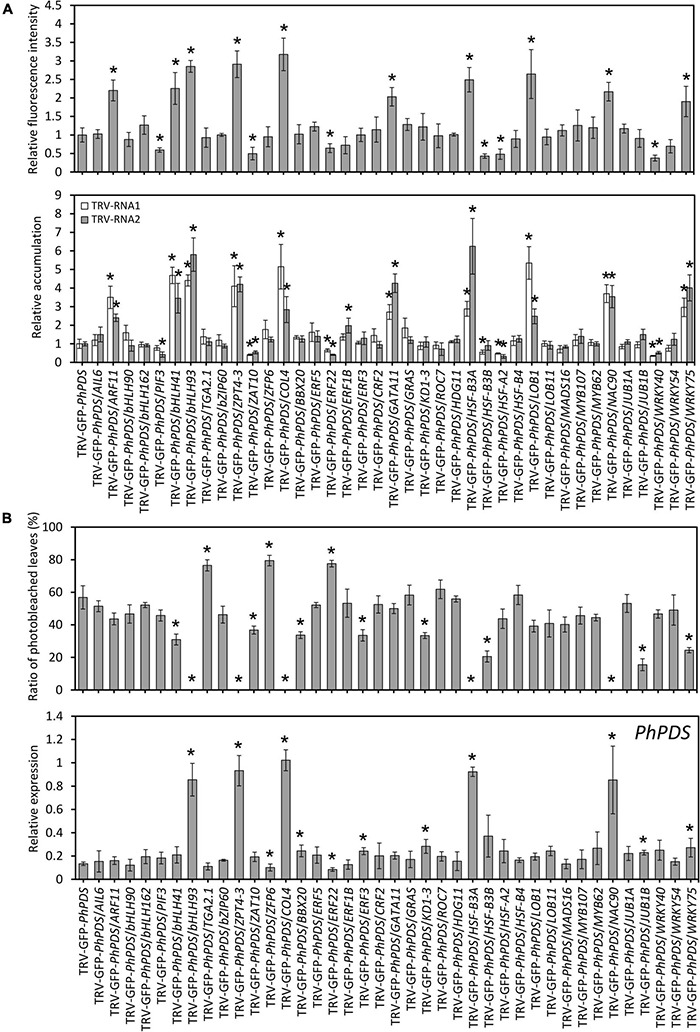
Virus accumulation and silencing efficiency in various transcription factors-silenced petunia leaves. **(A)** Relative GFP fluorescence intensities and TRV (RNA1 and RNA2) accumulation levels in the inoculated leaves at 4 days post inoculation (dpi) with *Agrobacterium* containing 39 transcription factors (TFs)-inserted TRV-GFP-*PhPDS* plasmids. **(B)** Percentage of photobleached leaves and expression of *PhPDS* in the uppermost leaves at 14 dpi with *Agrobacterium* containing various constructs. *26S rRNA* was used as an internal control. Error bars represent standard error of the mean from three biological replicates. Asterisks indicate statistical significance as determined by Student’s *t*-test at *P* < 0.05.

Transcript levels of 39 candidate TFs were examined in the uppermost leaves of petunia plants infected with TRV constructs. Results showed that all TF transcripts were significantly down-regulated in the corresponding TRV construct-infected petunia leaves (Supplementary Figure S4). Compared to TRV-GFP-*PhPDS* control, higher levels of GFP fluorescence intensity were observed with the down-regulation of *PhARF11*, *PhbHLH41*, *PhbHLH93*, *PhZPT4-3*, *PhCOL4*, *PhGATA11*, *PhHSF-B3A*, *PhLOB1*, *PhNAC90*, and *PhWRKY75*. On the contrary, silencing of *PhPIF3*, *PhZAT10*, *PhERF22*, *PhHSF-B3B*, *PhHSF-A2*, and *PhWRKY40* led to lower fluorescence levels. TRV-RNA1 and TRV-RNA2 accumulation levels exhibited highly similar patterns to GFP fluorescence intensities (Figure 7A).

At the phenotype level, TRV-GFP-*PhPDS*/*TGA2.1*-, *ZFP6*-, and *ERF22*-infected plants displayed remarkably higher rates of photobleached leaves than the control (TRV-GFP-*PhPDS*). In contrast, TRV-GFP-*PhPDS*/*bHLH41*-, *bHLH93*-, *ZPT4-3*-, *ZAT10*-, *COL4*-, *BBX20*-, *ERF3*-, *KD1-3*-, *HSF-B3A*-, *HSF-B3B*-, *NAC90*-, *JUB1B*-, and *WRKY75*-infiltrated plants demonstrated much lower rates of photobleached leaves. Compared to the control, the transcription of *PhPDS* was lower in the leaves with enhanced photobleaching, but higher in the leaves with reduced photobleaching (Figure 7B), which is consistent with the leaf phenotype.

### *PhCOL4* Modulates the Expression of RNA Silencing-Related Genes

**FIGURE 8:**
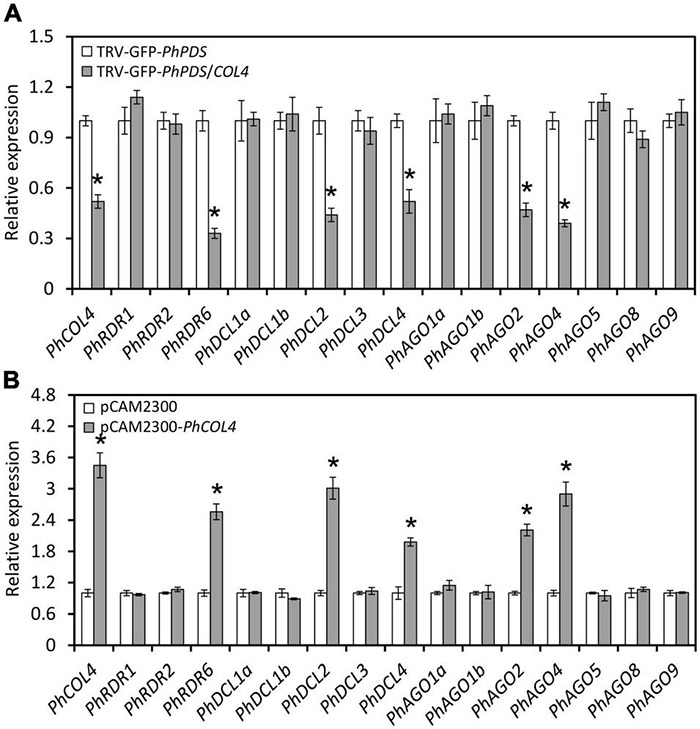
Impact of *PhCOL4* silencing and transient overexpression on RNA silencing-related genes. **(A)** RT-qPCR analysis of transcript levels of *PhCOL4* and RNA silencing-related genes in the inoculated leaves at 4 days post inoculation (dpi) with *Agrobacterium* containing TRV-GFP-*PhPDS* or TRV-GFP-*PhPDS*/*COL4*. **(B)** RT-qPCR analysis of transcript levels of *PhCOL4* and RNA silencing-related genes in the infiltrated leaves at 6 dpi with *Agrobacterium* containing pCAMBIA2300-*PhCOL4* (pCAM2300-*PhCOL4*). The empty vector pCAMBIA2300 (pCAM2300) was used as the control. Expression levels were standardized to *26S rRNA*. Error bars represent standard error of the mean from three biological replicates. Significance of difference was calculated by Student’s *t*-test at *P* < 0.05 and indicated by asterisks.

In view of the prominent impact of *PhCOL4* (SGN accession no. Peaxi162Scf00045g01824) silencing on GFP fluorescence and leaf photobleaching in the VIGS assay, we selected it for further functional characterization. Expression levels of *PhCOL4* were examined in its silenced and transiently overexpressed petunia leaves. At 4 dpi with TRV-GFP-*PhPDS*/*COL4*, petunia leaves exhibited decreased transcript levels of *PhCOL4* and several RNA silencing-related genes, including *PhRDR6*, *PhDCL2*, *PhDCL4*, *PhAGO2*, and *PhAGO4*, compared to TRV-GFP-*PhPDS* control (Figure 8A). Transient overexpression of *PhCOL4* in petunia leaves resulted in increased transcript abundances of *PhRDR6*, *PhDCL2*, *PhDCL4*, *PhAGO2*, and *PhAGO4*, compared to empty vector control (Figure 8B).

### DAP-Seq Analysis of PhCOL4 Target Genes

**FIGURE 9:**
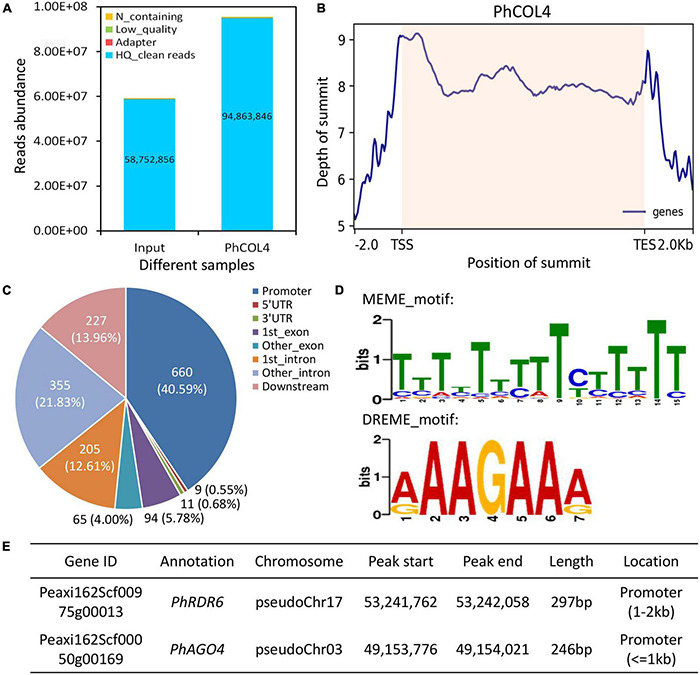
Binding peaks and motifs of PhCOL4 by DNA affinity purification sequencing in petunia. **(A)** Abundance of reads from PhCOL4-bound DNA affinity purification sequencing (DAP-Seq) database. **(B)** Distribution of summits in peaks around transcription start sites (TSS) and end sites (TES) of genes. Summit positions within transcribed regions are shaded. **(C)** Number of peaks in different genomic categories. The promoter indicates the 2 kb region upstream of TSS, and the downstream represents the 2 kb region downstream of TES. **(D)** Two top DNA motifs bound by PhCOL4 from MEME and DREME analyses of DAP-Seq database. **(E)** Two putative PhCOL4-bound peaks and corresponding RNA silencing-related genes.

To screen downstream target genes of PhCOL4, a DAP-Seq assay was performed. A total of 94,863,846 high-quality reads were generated from PhCOL4-bound products, compared to input control (Figure 9A). A number of peaks were located before transcription start sites of genes (Figure 9B). Of the 1,626 peaks, 660 (40.59%) were located within the 2kb upstream promoter regions. The remaining peaks were located within the regions of 5′UTR, 3′UTR, exon, intron, and downstream (2 kb after transcription end sites) (Figure 9C). Analysis of MEME and DREME revealed two top PhCOL4-bound motifs with the core element TTCTT (or AAGAA in the opposite strand) (Figure 9D). Through the functional annotation of peaks, two important genes, *PhRDR6* and *PhAGO4*, involved in the RNA silencing pathway were found (Figure 9E).

### PhCOL4 Binds to the *PhRDR6* and *PhAGO4* Promoters

**FIGURE 10:**
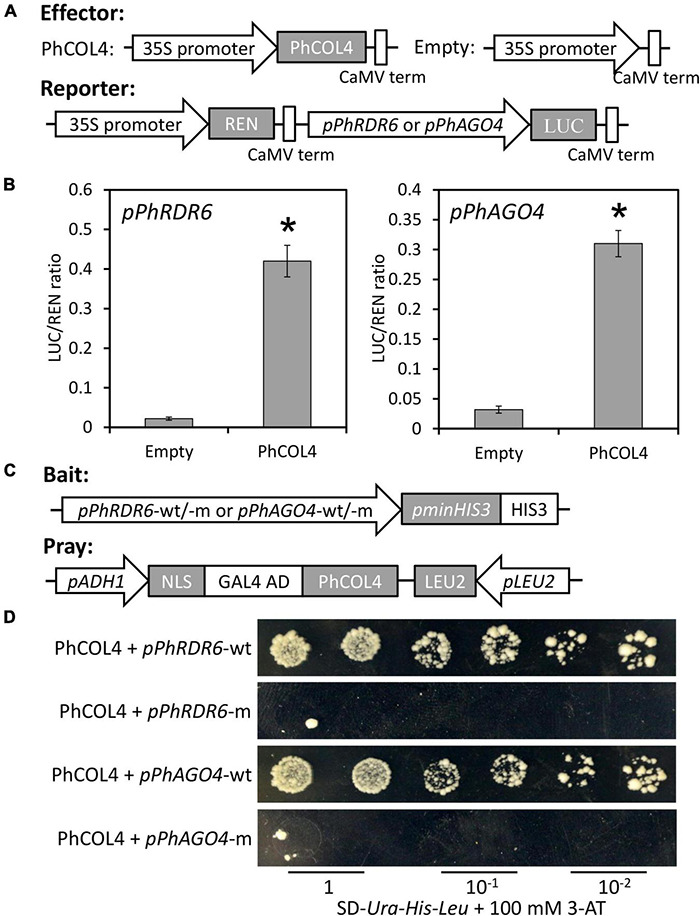
Transactivation of *PhRDR6* and *PhAGO4* promoters by PhCOL4. **(A)** Schematic diagrams of the effector and reporter constructs for dual luciferase assay. REN, *Renilla* luciferase; LUC, firefly luciferase. **(B)** Dual luciferase assay of the *pPhRDR6* and *pPhAGO4*. The activation was expressed as a ratio of LUC to REN. Error bars indicate standard error of the mean from three biological replicates. Statistical significance was determined using Student’s *t*-test (*P* < 0.05) and shown as asterisks. **(C)** Schematic diagrams of the bait and pray constructs for yeast one-hybrid assay. The fragments containing wild-type (wt) and mutant (m) PhCOL4-bound motifs were used for construction of bait vectors. **(D)** Growth of yeast cells transformed with the bait and pray plasmids on SD-*Ura-His-Leu* plates supplemented with 100 mM 3-aminotriazole (3-AT) at different dilutions.

To determine the transactivation of *PhRDR6* and *PhAGO4* promoters by PhCOL4, a dual luciferase assay based on the effector and reporter constructs was carried out (Figure 10A). Compared to empty vector control, the co-expression of *35S:PhCOL4* with *pPhRDR6:LUC* or *pPhAGO4:LUC* caused a dramatic increase in LUC activity (Figure 10B). To further confirm the interaction of PhCOL4 with predicted motifs by DAP-Seq, a 35-bp fragment containing motif sequence in the *PhRDR6* or *PhAGO4* promoter was used as a probe for yeast one-hybrid assay. The constructs of promoter and PhCOL4 were used as the bait and pray, respectively (Figure 10C). The yeast cells co-transformed with PhCOL4 and wild-type promoter fragments grew well on the selective medium in the presence of 100 mM 3-AT. However, the growth of yeast transformants with mutant promoter fragments were remarkably inhibited (Figure 10D). These results revealed the direct interaction between PhCOL4 and the promoters of *PhRDR6* and *PhAGO4*.

## Discussion

RNA silencing is an efficient antiviral mechanism in plants, which can suppress the infection of various viruses (Yang and Li, 2018; Guo et al., 2019). The lack of an effective screening system has limited our ability to identify the regulatory machinery that controls antiviral RNA silencing. In this study, we adopted an integrative approach of high-depth RNA-Seq and transient VIGS to screen important TFs regulating the RNA silencing process. This approach has the advantages of fast detection, high throughput, and excellent selectivity. It has been widely employed to characterize the biotic stress-defensive genes in *Zea mays* (Linde and Doehlemann, 2013), *Solanum lycopersicum* (Paz et al., 2011; Zuluaga et al., 2013), *Gossypium hirsutum* (Gao et al., 2016; Zhang et al., 2016), *N. benthamiana* (Luo et al., 2019), and *Rosa chinensis* (Gao et al., 2021). A recent publication reported that two functional genes, *GmSEOB* and *GmPAP27*, were identified to confer resistance to soybean mosaic virus through integrative method of transcriptome and VIGS (Sun et al., 2021).

Endogenous hormones have proven to be tuners of plant response to environmental stimuli and biotic stresses, such as virus infection. In this study, we found increased production of ET, ABA, JA, and SA in TRV (PPK20)-infected petunia leaves (Figure 3A), suggesting their potential roles in modulating antiviral defense. These hormones have been revealed to affect plant resistance to various viruses, including tobacco necrosis virus (Iriti and Faoro, 2008), PVX (Lee et al., 2011), tomato bushy stunt virus (TBSV) (Sansregret et al., 2013), TMV (Zhu et al., 2014), bamboo mosaic virus (BaMV) (Alazem et al., 2017), and plum pox virus (PPV) (Pasin et al., 2020). These studies suggest that ABA, JA, and SA may have positive contribution to viral defense, whereas the function of ET varies across species infected by different types of viruses. For example, the mutant of *ACS1*, a key ET biosynthetic gene, showed reduced susceptibility to TMVcg (Chen et al., 2013). However, a contrasting report showed that the accumulation of TBSV was dramatically disrupted in ET-sensitive but not in ET-insensitive plants (Sansregret et al., 2013). Our data showed that a rapid production of ABA and JA occurred at 1 and 2 dpi with TRV (PPK20) (Figure 3A). Previous studies have revealed that ABA- and JA-mediated plant defense against viral attack occurs at early stages of infection (Asselbergh et al., 2008; García-Marcos et al., 2013). Their defensive effects can be compromised at later phases, due to their antagonistic interactions with SA (Alazem and Lin, 2015). This may explain the divergent induction profiles between ABA/JA and other hormones during the initial stage of TRV (PPK20) infection.

It is worth mentioning that the plants respond differently to virus infection between local and systemic tissues, especially of endogenous hormones. For example, SA is crucial for the establishment of local and systemic resistance to viral attack. The infection with TMV leads to a remarkable elevation in SA levels in both inoculated and systemic leaves of tobacco (Vlot et al., 2009). ABA is active in the inoculated Arabidopsis leaves with BaMV, whereas this activeness is only found in the systemic leaves upon infection with TMVcg (Chen et al., 2013). Obuda pepper virus infection significantly increases the accumulation of some hormones, such as SA, ABA, and JA, in the inoculated leaves rather than the systemic leaves of pepper (Dziurka et al., 2016). These results suggest that the hormones may play variable roles in the defense against virus invasion in different plant tissues. This possibility requires further examination in the subsequent studies.

There is increasing evidence showing the participation of hormones in RNA silencing machinery. It has been reported that ABA modulates the transcription of *AGO*s, and ABA-elicited defense response against BaMV depends on AGO2 and AGO3. Mutation of the ABA biosynthesis pathway attenuates the expression of *AGO2* and accelerates the replication and systemic movement of PVX (Alazem et al., 2017). Expression of *NahG*, a gene contributing to the degradation of SA, decreases the abundances of PPV-derived small RNAs (Alamillo et al., 2006). SA treatment results in elevated transcript levels of *RDR1* in tobacco (Hunter et al., 2013) and *RDR1*, *RDR2*, *DCL1*, and *DCL2* in tomato (Campos et al., 2014). Some TFs associated with ABA and SA production share similar expression patterns with a few RNA silencing-related genes. These TF-bound *cis*-acting elements could be detected in the promoter regions of some *RDR*s, *DCL*s, or *AGO*s (Alazem et al., 2019). In addition, a rice TF JAMYB participates in the JA signaling pathway and specifically targets the downstream gene *AGO18*, a core RNA silencing component imparting resistance to rice stripe virus (Yang et al., 2020). This implies that ABA, SA, and JA may regulate different components of the RNA silencing machinery. Although the direct link between ET and antiviral RNA silencing has not been described, our previous work suggested that PhERF2, an ethylene-responsive element binding factor, regulated gene silencing efficiency and resistance to TRV and CMV infections (Sun et al., 2016). Another example is that ET-inducible TF RAV2 is essential for HC-Pro and P38 (two plant viral suppressors of silencing) to inhibit the silencing process (Endres et al., 2010). These observations suggest that the involvement of ET in RNA silencing-based antiviral defense is a complicated process. An extensive investigation of the impact of hormone treatment on expression of RNA silencing-related genes will be required in future work.

In the present study, we verified a number of TRV (PPK20)-induced TFs for their effects on GFP-labeled virus accumulation and *PhPDS*-silenced photobleaching phenotype. These TFs may serve as putative transcriptional regulators of antiviral RNA silencing (Figure 6). We found that these TFs belong to a wide range of families. Silencing of *PhARF11*, *PhbHLH41*, *PhbHLH93*, *PhZPT4-3*, *PhCOL4*, *PhGATA11*, *PhHSF-B3A*, *PhLOB1*, *PhNAC90*, and *PhWRKY75* led to increased fluorescence intensities and TRV accumulation. Consistently, *PhbHLH41*, *PhbHLH93*, *PhZPT4*-*3*, *PhCOL4*, *PhHSF-B3A*, *PhNAC90*, and *PhWRKY75* silencing greatly reduced photobleaching phenotypes (Figures 6, 7). Therefore, these 7 TFs can be identified as positive regulators of antiviral RNA silencing. Moreover, the other TFs, whose down-regulation increased virus accumulation, may play their antiviral roles beyond the RNA silencing machinery. In contrast to the above TFs, petunia leaves with down-regulation of *PhPIF3*, *PhZAT10*, *PhERF22*, *PhERF1B*, *PhHSF-B3B*, *PhHSF-A2*, and *PhWRKY40* displayed decreased GFP-monitored virus accumulation, implying their negative roles in modulating antiviral responses. Considering the enhanced photobleaching development in TRV-GFP-*PhPDS*/*ERF22*-infiltrated leaves, *PhERF22* may function as a suppressor of antiviral RNA silencing. Numerous studies have indicated that viruses usually hijack the host cellular machinery or components to evade plant’s immunity system and facilitate their infection (Patarroyo et al., 2013; Fang et al., 2019). A recent example showed that beet severe curly top virus can activate the *Arabidopsis* gene *VIM5*, encoding an E3 ubiquitin ligase, to repress the RNA silencing process (Benoit, 2020). It remains to be investigated whether a cross-talk between TRV proteins and petunia PhERF22 exists.

It is noteworthy that the down-regulation of a few TFs only led to altered photobleaching phenotype but no change to GFP intensity, i.e., virus accumulation. These TFs included *PhBBX20*, *PhERF3*, *PhKD1-3*, *PhTGA2*.*1*, and *PhZFP6* (Figure 6). We reasoned that this phenomenon may be attributed to the environment–plant interactions and intra-plant variation of RNA silencing-based traits. It has been reported that the VIGS efficiency probably changes under certain environmental factors. Low temperature and humidity can increase TRV-induced gene silencing efficiency in tomato (Fu et al., 2006). In previous studies, we have also observed variable silencing phenotypes in various TRV-*PhPDS*-infected petunia plants (Sun et al., 2017). These findings demonstrate that the spread of virus or virus-derived silencing signals can be influenced by external factors, leading to varied levels of virus infection. Further study is necessary to investigate the potential impact of these external factors on virus infection.

In this work, we found a zinc finger TF, PhCOL4, plays an essential role in antiviral RNA silencing by specifically activating *PhRDR6* and *PhAGO4*, two important components in the RNA silencing pathway (Figure 10). Some reports have revealed the important functions of PhRDR6 (Li et al., 2012) and PhAGO4 (Carbonell and Carrington, 2015; Kenesi et al., 2021) in RNA silencing-mediated antiviral defense. In *Arabidopsis*, AtCOL4, a homolog of PhCOL4, has been suggested to function as a modulator of flowering time (Steinbach, 2019) and abiotic stress tolerance (Min et al., 2015). Our results provided the direct evidence for the involvement of PhCOL4 in the antiviral RNA silencing process, thus corroborating the effectiveness of integrative approach of transcriptome and VIGS in identifying transcriptional regulators. However, how PhCOL4, PhRDR6, and PhAGO4 synergistically modulate plant defense against virus is still unknown. Future studies should include an investigation of antiviral roles of PhCOL4 and its target genes in petunia.

## Conclusion

We adopted an integrative approach of high-depth RNA-Seq and VIGS assay to investigate the regulatory mechanism of antiviral RNA silencing in petunia. Our results showed that TRV (PPK20) infection induced significant transcriptional changes in petunia, which involve various TFs and RNA silencing-related genes. A number of TFs possibly regulating antiviral RNA silencing were identified through the TRV-GFP-*PhPDS*-based VIGS screening system. One potential positive regulator, *PhCOL4*, was further validated for its crucial role in antiviral RNA silencing by specifically activating the *PhRDR6* and *PhAGO4* promoters. These transcriptional regulators may be used for future genetic engineering of virus resistance in this important ornamental plant.

## Data Availability Statement

The RNA-Seq and DAP-Seq data are available at GenBank (https://www.ncbi.nlm.nih.gov/genbank/) with accession BioProject PRJNA693880 and PRJNA693899, respectively.

## Author Contributions

YX and DS designed the research and wrote the manuscript. YX, XJ, ZX, YY, and XC carried out the experiments. DS and DK analyzed the RNA-Seq and DAP-Seq data. YX performed the graphic arrangement. DS and YZ revised the manuscript and improved the English. All the authors contributed to the article and approved the submitted version.

## Conflict of Interest

The authors declare that the research was conducted in the absence of any commercial or financial relationships that could be construed as a potential conflict of interest.

## Publisher’s Note

All claims expressed in this article are solely those of the authors and do not necessarily represent those of their affiliated organizations, or those of the publisher, the editors and the reviewers. Any product that may be evaluated in this article, or claim that may be made by its manufacturer, is not guaranteed or endorsed by the publisher.

## Footnotes

1
https://solgenomics.net/organism/Petunia_axillaris/genome

2
http://wego.genomics.org.cn/cgi-bin/wego/index.pl

3
http://www.genome.jp/kegg/

4
http://www.premierbiosoft.com/primerdesign/

5
https://solgenomics.net/organism/Petunia_axillaris/genome
