# Supplementary material for: Transcriptome Profiling Reveals a Petunia Transcription Factor, PhCOL4, Contributing to Antiviral RNA Silencing
Source: Front Plant Sci. 2022 Apr 14;13:876428. doi: 10.3389/fpls.2022.876428 (PMC9047179; doi:10.3389/fpls.2022.876428)
Supplement: Supplementary file 1 [file Table_1.DOCX]

**Supplementary Table S1** Primers used for real-time quantitative PCR and plasmid construct.

| **Gene ID** | **Forward primer (5’-3’)** | **Reverse primer (5’-3’)** | **Product size** |
| --- | --- | --- | --- |
| For gene expression analysis | | | |
| TRV-RNA1 | CAGTCTATACACAGAAACAGA | GACGTGTGTACTCAAGGGTT | 463bp |
| TRV-RNA2 | GGTTACTAGCGGCACTGAATAGA | TAGTACTCCCTTGGTTCGTCGTA | 225bp |
| *PhRDR1* | TCATGATGTCACAATTGAGGAAG | TTCGGAGTGATAGGTTGTCTTGT | 271bp |
| *PhRDR2* | CACAAGAATTGGAAATCAAGAGG | TTAACATTCTCCCAACTCTCCAA | 254bp |
| *PhRDR6* | AGTTAGCATTGAAGGTTGCAGAG | TGGTCGAACTCAAATTTCTTCAT | 169bp |
| *PhDCL1a* | AGCTCTATGAGACATGCCAGAAG | CCTTGTTAGCTGCTCTGTTCAAT | 159bp |
| *PhDCL1b* | AGAATTTGTTGATTCATGGGAGA | CTAGTGGCTTCCACACTCAAACT | 165bp |
| *PhDCL2* | GTTATCAGTTGGAAGCATTGGAG | AGTAGCAGCATCCCAATAATCAA | 266bp |
| *PhDCL3* | ATGGATGTTGCAAAGAAGAAGAA | TTCTGATCACCATTCCTCAAAGT | 182bp |
| *PhDCL4* | CAGCTCTTCTGTTTGGACAACTT | AAGGTTCATGGCATTTCTCTGTA | 292bp |
| *PhAGO1a* | TCTATCAAAGTATCCGCCCTACA | CTTGAGATGTTAAACCCGAGATG | 249bp |
| *PhAGO1b* | AAGAATCTCATCTGGGAAAGAGG | TGCATCAGCTTGTCTACCTTGTA | 230bp |
| *PhAGO2* | ACCCAAGTCTGATCTTCGTATCA | TATCACGAGGAATGGATGAGAGT | 266bp |
| *PhAGO4* | CTAATAGGAACAATGGCAACAGC | ATGCTGCCTCAAGATTATATCCA | 221bp |
| *PhAGO5* | GTGTAATGCTGACGAAATGATGA | TGAGAAGTTGACACAAGTCCAGA | 214bp |
| *PhAGO8* | AGACTTCATTGAGGGTGATCAAA | GTTCCTAGTCTCGTTGGGAAAGT | 238bp |
| *PhAGO9* | GAGTGAGTGAGTCCCAATTCAAC | CAGGAGGAACATTATCAGGTGAG | 171bp |
| *PhAIL6* | CCTCCTATGATGGCTCTTCTTCT | TGTTGTTGGTGAATTTGTGACTC | 157bp |
| *PhARF11* | TGCAGAGGCCATAGTCTACTCTC | ATGGTTACTATTTCCGCAGGATT | 174bp |
| *PhbHLH90* | CAACAAATGAACCAAAGTGGATT | TGAAACAGCCCTTGGAACTATTA | 228bp |
| *PhbHLH162* | GGAAAGGAGCAGTAAGTGCAATA | CCTTGTGATACCTCCTTCCTTCT | 255bp |
| *PhPIF3* | CTACAAATGGCAGAAGAAGCAAC | ATTATGACAAGCAGAATCGTCGT | 267bp |
| *PhbHLH41* | CATTGAAAGCTCAAGTGGAAGAA | ATTGGTTTGCACTGACTCTAAGC | 287bp |
| *PhbHLH93* | AAGGAGGAGAAGGAAACGACTTA | CGTTGCTCCTTTCTACATCAAAC | 263bp |
| *PhTGA2.1* | ACATGGATGGCAATGATAAGAAT | TGCTTGAACTGTGTATTCCCTTT | 264bp |
| *PhbZIP60* | AGGTCACGAGAGAGGAAGAAGTT | ACCATTAGCACCGCTATTCTGTA | 156bp |
| *PhZPT4-3* | CTTGGATATGGAATGGTGTCAAT | ATCAGCTTCCAACATGTCAACTT | 197bp |
| *PhZAT10* | CCGCCTTATTAGATGACAACAAC | TCACAACACTTCCACTATGACCA | 243bp |
| *PhZFP6* | AATCCTATTAGCTCTGCCTTTGC | CGCTCTTGATACCCTACTCATTG | 300bp |
| *PhCOL4* | TTAGCCAAAGTGTCTCTTCTTCG | CCCTTTGATTCTAGGTCGAGTTT | 248bp |
| *PhBBX20* | TCATCATGCCAATAAACTTGCTA | AGAAGAAAGCTGCACTCCAGTTA | 226bp |
| *PhERF5* | ACACCTCCTCGGAGACTTTACTT | AGCTGATGTAACTGGGCTTAGTG | 166bp |
| *PhERF22* | TCAAATTCAGGCGATTAATGAGT | AGAACCATGATGTTGTTCCATTT | 193bp |
| *PhERF1B* | TACTTGGTGAATCCTCAGACACA | TGTTGAATCCCTTATTTCTGCTG | 167bp |
| *PhERF3* | TATAAGGTGGCGAATTCAAGAAA | TACAACATGAGTCAAGGCAGAAA | 297bp |
| *PhCRF2* | GGGAATTCAGTCATGTCTCCTAT | GCCTCTTGATTCTCCGTACATTA | 196bp |
| *PhGATA11* | CAACTCTTGTCTCCAACATCCTC | TCTCAATTGGAGCTAGTGGAAAG | 184bp |
| *PhGRAS* | TTACGAGTCAGACAAGGAGAAGC | TATTGGTGTTCGATTCTTGTTCA | 169bp |
| *PhKD1-3* | GCACACTAACGCATCATCATCTA | TTGTATGCTAGATCCACCTTTGC | 294bp |
| *PhROC7* | GTGGTCGGAGAAGTATAGCAATG | GAATCGCTATTGGACAAGACATC | 283bp |
| *PhHDG11* | AGGATGGTGAAGAGTTTCTGTGA | CATTCACATGGTTCCCATTAGTT | 256bp |
| *PhHSF-B3A* | TAAGGAAGTCATCAACACCACCT | AGAGTGTTGGAAGCAAATCTCTG | 153bp |
| *PhHSF-B3B* | GAGAAAGTCATCACCATCACCAT | TTGCAACTTTACGGAAACCATAG | 218bp |
| *PhHSF-A2* | ATATGGGAACTGACATGGAAATG | GTTGAACTGCAGCCTAACAAGAT | 242bp |
| *PhHSF-B4* | GGATTGGGAATTTCTTACTGCTC | ACAAATCCTTAGCAAATTCAGCA | 299bp |
| *PhLOB1* | GGTGTATGAAGCCAATGCTAGAC | AATTGTCCAAAGATTGTTGTGGT | 224bp |
| *PhLOB11* | TCTCATCATCACGTAACTCTCCA | CAGCTGAACCATAGACAGGATCT | 279bp |
| *PhMADS16* | AGAGAACTCAACAAACAGGCAAG | CCAAGTGTCCTTTGGTACAAATC | 195bp |
| *PhMYB107* | GGCTTGAAGAAAGGTACTTGGAC | TGACCATTTATTCCCAATTTCTG | 240bp |
| *PhMYB62* | ATGATATTGGGTTGAGGAGAGGT | GAGTTGTTCCTGTGGAGTGAGAT | 206bp |
| *PhNAC90* | TTCAGCTTGTGCAGAGTTTACAA | AGATGATGAACTTTCCACTGAGG | 159bp |
| *PhJUB1A* | CCTACCGATGAAGAACTTGTCAG | CTCCCTCTCATGCAGAAGAAATA | 170bp |
| *PhJUB1B* | GATTCCATCCAACAGATGAAGAG | CAGAACATGTCACCCTGTTAGGT | 222bp |
| *PhWRKY40* | TGACCTTAACACGAATCCTTCAC | GTCAATTAGGTGAGTTTGCAAGG | 160bp |
| *PhWRKY54* | CACAAGTGGATGACAATTTGGTA | TGATGAAGTGTTGGCTGAGAATA | 176bp |
| *PhWRKY75* | TCCCAAGAAGCTACTACAAGTGC | TCTGACGCATCAAGATATTCTCA | 168bp |
| *PhPDS* | CGAATAAGAAAGATCGAGCTGAA | CAGGCAAAAGAAGCTTGAAAATA | 130bp |
| *26S rRNA* | AGCTCGTTTGATTCTGATTTCCAG | GATAGGAAGAGCCGACATCGAAGG | 185bp |
| For fragment amplification in VIGS assay | | | |
| *PhPDS* | GAGCTCCAGTGCTTCTTGATCGCTTTGAA | CTCGAGTCTGACTTGGCCACCTTTTGACT | 150bp |
| *PhAIL6* | CTCTAGAAGGCCTCCATGGGTCCCTGTTTCCAGTTATGCTAAA | GAAGCACTGGAGCTCGGTACCTATTCATAATGGCCTCGACATC | 362bp |
| *PhARF11* | CTCTAGAAGGCCTCCATGGGGGAACGTAAAGCTAGCACTTCAA | GAAGCACTGGAGCTCGGTACGGCCTTCATAAGGAATCTGACTT | 375bp |
| *PhbHLH90* | CTCTAGAAGGCCTCCATGGGGGACAAAGTTGCAATACTTGGAG | GAAGCACTGGAGCTCGGTACCTCCAGGTTTATGTGATCCAGAG | 330bp |
| *PhbHLH162* | CTCTAGAAGGCCTCCATGGGAGAAGGAAGGAGGTATCACAAGG | GAAGCACTGGAGCTCGGTACTTCTCACCTGAGCATGAACTGTA | 308bp |
| *PhPIF3* | CTCTAGAAGGCCTCCATGGGCTCATTCATCATCCAAATCCAAT | GAAGCACTGGAGCTCGGTACTATACAAAGCTGCCATGTTGTTG | 281bp |
| *PhbHLH41* | CTCTAGAAGGCCTCCATGGGTCACCTAGACAAATCCAAGGAAA | GAAGCACTGGAGCTCGGTACACCTGTGTGTTCGTAGGACAGTT | 264bp |
| *PhbHLH93* | CTCTAGAAGGCCTCCATGGGTCCAAGGATAGAAAGTTGGAACA | GAAGCACTGGAGCTCGGTACGATTGTGAAGGAGAGAGGAGTGA | 193bp |
| *PhTGA2.1* | CTCTAGAAGGCCTCCATGGGCAGAAGATGCTCTTTCACAAGGT | GAAGCACTGGAGCTCGGTACCTAGCAAGCCAGAGAGAACTGAG | 344bp |
| *PhbZIP60* | CTCTAGAAGGCCTCCATGGGTAATTTCATCAATTGGGAGGATG | GAAGCACTGGAGCTCGGTACAAGATTGTTCAGTTCCACAGCAT | 362bp |
| *PhZPT4-3* | CTCTAGAAGGCCTCCATGGGCATAAGAATTGGCAAGCATAAGG | GAAGCACTGGAGCTCGGTACTGACTGCTGAAGATTGATTGAGA | 270bp |
| *PhZAT10* | CTCTAGAAGGCCTCCATGGGCACCAACTCTACCACCACGTTAT | GAAGCACTGGAGCTCGGTACCGCCAAGAGCTTGATAAGAACTA | 361bp |
| *PhZFP6* | CTCTAGAAGGCCTCCATGGGAGTTGTTTGGTTTCAACGTGACT | GAAGCACTGGAGCTCGGTACGCAAAGGCAGAGCTAATAGGATT | 308bp |
| *PhCOL4* | CTCTAGAAGGCCTCCATGGGCATGTGACCAAGATATCCACTCA | GAAGCACTGGAGCTCGGTACCGGATCCACATCACTAAACAAAT | 297bp |
| *PhBBX20* | CTCTAGAAGGCCTCCATGGGCAAATACACCAAGCAAATGAACA | GAAGCACTGGAGCTCGGTACCCTCTTGGACATAATCAACTTGG | 263bp |
| *PhERF5* | CTCTAGAAGGCCTCCATGGGAACCCAATAATTTCGACCAAGAT | GAAGCACTGGAGCTCGGTACTCTTGAAAGCTGCACAGTCATAA | 337bp |
| *PhERF22* | CTCTAGAAGGCCTCCATGGGTTTGGCTAGGGAGTTATGAGACA | GAAGCACTGGAGCTCGGTACTCATTAATCGCCTGAATTTGACT | 329bp |
| *PhERF1B* | CTCTAGAAGGCCTCCATGGGTATGCAGCAGAAATAAGGGATTC | GAAGCACTGGAGCTCGGTACTCTAACAATTCAGCTCCCAAATC | 363bp |
| *PhERF3* | CTCTAGAAGGCCTCCATGGGCCACAACTTCAACAAACTTCACA | GAAGCACTGGAGCTCGGTACGAAGCTGCAGAGGTAGCTAATGA | 369bp |
| *PhCRF2* | CTCTAGAAGGCCTCCATGGGCACGTGACAGAGATCAACATCAT | GAAGCACTGGAGCTCGGTACTAACTCCTCCGTTGAAACTAACG | 319bp |
| *PhGATA11* | CTCTAGAAGGCCTCCATGGGATGAGTGTGGTGACCTTGACTCT | GAAGCACTGGAGCTCGGTACCTCTCAAGCACAGAAACAGGACT | 335bp |
| *PhGRAS* | CTCTAGAAGGCCTCCATGGGCAAGTATTGGACATAGCCTCCAG | GAAGCACTGGAGCTCGGTACCTCATTCCTCATCGCTTCTTCTA | 388bp |
| *PhKD1-3* | CTCTAGAAGGCCTCCATGGGAAGTCAACGGCCTATTGATGATA | GAAGCACTGGAGCTCGGTACACATGGTTGCACCTGTACTTTCT | 240bp |
| *PhROC7* | CTCTAGAAGGCCTCCATGGGAAAGGCATCTGCCATAGTGATTA | GAAGCACTGGAGCTCGGTACGTTGTGAACCTTCTTGGATTGAG | 342bp |
| *PhHDG11* | CTCTAGAAGGCCTCCATGGGCAGCAACAATTATTCCATCACAA | GAAGCACTGGAGCTCGGTACATGATTCAATTCGAGCAGTTGAT | 289bp |
| *PhHSF-B3A* | CTCTAGAAGGCCTCCATGGGTAACATTGAACAAACCAATGCAG | GAAGCACTGGAGCTCGGTACTTTCCTCTGAACTTTCTGCAAAC | 270bp |
| *PhHSF-B3B* | CTCTAGAAGGCCTCCATGGGAGCCAAATAACAGCAACAACAAT | GAAGCACTGGAGCTCGGTACTGAACTTCCAACCTTACTCCAAA | 341bp |
| *PhHSF-A2* | CTCTAGAAGGCCTCCATGGGTTTAGAAAGGTGGATCCTGACAA | GAAGCACTGGAGCTCGGTACCATCATCTGCTCTTGTTTCCTCT | 349bp |
| *PhHSF-B4* | CTCTAGAAGGCCTCCATGGGACTTCTGCATCTCCTGACAATTC | GAAGCACTGGAGCTCGGTACACATTCTCATCCAAGGAGCATTA | 344bp |
| *PhLOB1* | CTCTAGAAGGCCTCCATGGGTCATCATCGTCTTCTCCTCCTAA | GAAGCACTGGAGCTCGGTACGTCTAGCATTGGCTTCATACACC | 308bp |
| *PhLOB11* | CTCTAGAAGGCCTCCATGGGCACTACTGCTCATCGTGTCTTTG | GAAGCACTGGAGCTCGGTACGACCTTGTCCAAAGATTGTTCTG | 353bp |
| *PhMADS16* | CTCTAGAAGGCCTCCATGGGAGATCCAACGTATGGTGTAGTGG | GAAGCACTGGAGCTCGGTACTCAAATCCCGACTTAAAGCAATA | 375bp |
| *PhMYB107* | CTCTAGAAGGCCTCCATGGGCAGAAATTGGGAATAAATGGTCA | GAAGCACTGGAGCTCGGTACTTCCACATGTATTGAAGCACAAG | 330bp |
| *PhMYB62* | CTCTAGAAGGCCTCCATGGGACGTGCTGGACTAAAGAGAACAG | GAAGCACTGGAGCTCGGTACATGGGAAATGAGCCTTGATAAAT | 360bp |
| *PhNAC90* | CTCTAGAAGGCCTCCATGGGGTATGATGTCCAACCAGGGTTTA | GAAGCACTGGAGCTCGGTACGATGAATAAACATAACCGGGACA | 349bp |
| *PhJUB1A* | CTCTAGAAGGCCTCCATGGGTCAAGAAGCTGAAGTTTGGACTC | GAAGCACTGGAGCTCGGTACGAATGAAGATTGAGGCATATTGG | 341bp |
| *PhJUB1B* | CTCTAGAAGGCCTCCATGGGAATCTTAAAGCGAAATGGTTCAT | GAAGCACTGGAGCTCGGTACTTCATGTCCGGAGCTAATGTACT | 282bp |
| *PhWRKY40* | CTCTAGAAGGCCTCCATGGGAGCTCAATGGTGAAAGATGGATA | GAAGCACTGGAGCTCGGTACTAGTCAAGGAAGATGCCATTTGT | 371bp |
| *PhWRKY54* | CTCTAGAAGGCCTCCATGGGAATTCTCAAATGGACTGGTTCAA | GAAGCACTGGAGCTCGGTACGTTGCTTCATTCTTGTCCAAATC | 397bp |
| *PhWRKY75* | CTCTAGAAGGCCTCCATGGGTTCTTCTTCGTCATCGTCTTCTC | GAAGCACTGGAGCTCGGTACCTTTGAACTTGCTTCTTCACGTT | 406bp |
| For fragment amplification in transient overexpression assay | | | |
| *PhCOL4* | GAATTCATGGCACGGCGTTGTGACTC | GAGCTCTTAAAACGATGGAACGACAC | 1,023bp |
| For fragment amplification in DAP-seq assay | | | |
| *PhCOL4* | ACTTTCAGAGCGATAACGCGATGGCACGGCGTTGTGACTC | TACCGAGCCCGAATTCGTTTTTAAAACGATGGAACGACAC | 1,051bp |
| For fragment amplification in dual luciferase assay | | | |
| *PhCOL4* | CCCCCGGGCTGCAGGAATTCATGGCACGGCGTTGTGACTC | GATTTCAGCGAATTGGTACCTTAAAACGATGGAACGACAC | 1,051bp |
| *pPhRDR6* | GGCCCCCCCTCGAGGTCGACTGAGGGTTGTGCTTCAACAA | GCTCTAGAACTAGTGGATCCACCGATTCCATCAGAAAAGT | 1,526bp |
| *pPhAGO4* | GGCCCCCCCTCGAGGTCGACTCATGTCTTACTCAACAGAG | GCTCTAGAACTAGTGGATCCAAGGTATATACTACAAGTAA | 1,365bp |
| For fragment amplification in yeast one-hybrid assay | | | |
| PhCOL4 | GAATTCATGGCACGGCGTTGTGACTC | CTCGAGTTAAAACGATGGAACGACAC | 1,023bp |
| *pRDR6-*wt | GAATTCCATGAATTTTTTTCTTTCAATTAAACTTTTTTTGT | GAGCTCACAAAAAAAGTTTAATTGAAAGAAAAAAATTCATG | 47bp |
| *pRDR6-*m | GAATTCCATGAATTTTTTAGCTTCAATTAAACTTTTTTTGT | GAGCTCACAAAAAAAGTTTAATTGAAGCTAAAAAATTCATG | 47bp |
| *pAGO4-*wt | GAATTCATTTGTGTGTTTTGTTTCTTTCGACTTTTTTTGCT | GAGCTCAGCAAAAAAAGTCGAAAGAAACAAAACACACAAAT | 47bp |
| *pAGO4-*m | GAATTCATTTGTGTGTTTTGTTAAGTTCGACTTTTTTTGCT | GAGCTCAGCAAAAAAAGTCGAACTTAACAAAACACACAAAT | 47bp |
