# Supplementary material for: Transcriptome Profiling Reveals a Petunia Transcription Factor, PhCOL4, Contributing to Antiviral RNA Silencing
Source: Front Plant Sci. 2022 Apr 14;13:876428. doi: 10.3389/fpls.2022.876428 (PMC9047179; doi:10.3389/fpls.2022.876428)
Supplement: Supplementary file 10 [file Table_10.docx]

**Supplementary Figure S1** Sequences of *PhCOL4* coding region, *PhRDR6* and *PhAGO4* promoter regions. Start and stop codons are marked with boxes and underlines, respectively.

>*PhCOL4* coding region

ATGGCACGGCGTTGTGACTCGTGCAAAACAACGTCGGCGACGGTGTTTTGCAAAGCCGACATGGCGTTTTTATGTTTAGGTTGTGACTCCAAAATTCACGCGGCGAATAAGCTCGCATCGAGGCACGCGCGTGTTTTGGTTTGTGAAGTGTGTGAACACGCGCCGGCTAGTGTCACATGCAAGGCTGATGCAGCTGCTCTTTGCGTGACATGTGACCAAGATATCCACTCAGCTAATCCTTTAGCTCGTCGCCACGAGCGGCTTCCTATAGTGCCGTTTTACGACTCCGCCTCAGCTAAATCTCGGGCTGCTGATGAAACTCAGCTGCCCGAGACTGAAGAGGAAGCTGAGGCGGAGTCTTGGCTACTACAGGCCCCGAATATTAATATTAATAATAATAATATCGGGGTCGAGGGAAATGAGTACAATAAATCAGCTGAGTATTTGTTTAGTGATGTGGATCCGTATGTGGATATGGAAATGATAACAGATCAGAAACCATGTACTGATATTCAGCTTCATCAAGAGTATAAGGAAGCAGATTGTGTTGTGCCTCATGTACAAAATAAGAATAATGAGATGATCCATTTACAAGGTCCAGTTGTTGATGGGTATCCTACATATCATGAAATGGACTTCACTGGATCTAAACCTTTCATGTACAACTTCAGTACTCAGTCCATTAGCCAAAGTGTCTCTTCTTCGTCTATGGAAGTGGGAGTTGTGCCTGATCATAATTCCATGGCAGATGTATCAAACACATTTGTGAGGAACTCCTCCACTGATGGCCTACCTACCCCAGTTTCAAGCTTAGACAGAGAAGCAAGGGTGTTGAGGTACAGAGAGAAAAGGAAGAACAGAAAGTTTGAGAAGACTATTCGATATGCTTCTAGAAAGGCATACGCTGAAACTCGACCTAGAATCAAAGGGAGATTTGCCAAACGTAACGAGATTGAAGTTGACTCCCTCATTGCCTCTGATGCTTCATATGGTGTCGTTCCATCGTTTTAA

>*PhRDR6* promoter region

ATTTACTAGGAAGCCTAGTAAAGATGCAACATAATATATCACCTGGAAGATGTATGGATGTATCACCTTTCAAGGCTCTCCTCTTTTTTACTTCATTCATCAACATCATGATTTCTACTGCAGCTAGTTTAAGCGAAGGGTGCAGCCTCTCTTTTTGCCTATATAGGGTTTACTTCGTCTTAATTTAATTACGTGAAAGGATAGAAGAAGGAAAACAGGTTCTATTAGAGTTGTCTTTGTAAGGAAAATAGATTCTTTCTTTTAAATATTAAATATTTATATATCTCATATTGGCTTACAATATATTTTTACCAATCACTGCAATAATATAACTTTACCTTTAAAGTGTTCTAATTATGTAATTACGACAAAAAAAGTTTAATTGAAAGAAAAAAATTCATGTTGAGAAGGATTGAAAAAAAATACAGGTGAAGAAGAAGCAAACAAATATGGCTCAACGGAACAACTCTCCTCTAAAAGTTAAGGAGAAGTGGGTTAAAAAAAAAACAAATGTTGAGGGTTGTGCTTCAACAAGTAGAGTTGATAGGGCGTTGATTACAAGAGAATCAACGTTGAAGTTGGAGAGAAAGTGCTTCAACTTCCATAACATAATAGGGTGTTGGTGCTTCAACAAAAACAAAGAGATACTTTGAATTATGGGAGAATATTGAAAATAATTCAAAGTTGATTTCATTATTAAGTAGTTTATTAAATTCATGTCTAATTACGTTTTAGTAAAACCATACTAGAATAAGTTTTCTAGTTAAAGTAGTTTTTGTTTGTTATTATAAATAGAGTGCTACACTCAAAGAGAAAGGTAAAAATCAAGAGCATCAAGCTTTATCTTCAAGCTAAGGGACGCTTTATAAAATTTTAAGGCATAAAGTAGAGATTTAATAAAATGTCATCAACTTTTTATATAATTAATATCCATTTTTATTTAAAATTACTCTTGCTAATTTTGAAATGCAAATTCATAAATTAATATTCTTTTTAGAGTTTATTTCATCAAGGTTCAATGGACAAATAGAGGCAATCATGCAAAAGAATCGCAACAATAAAGTTAAAGTTGTATTATCATGACATTAGCAATGTAGTAAATTTTCTATCCAAAATCCTGAGCTAATTGATTATTAAGAAAATCAAATTTTCCATTGTTGTACTTTTCGTAATATCTCAGGACTTTGAATTTAACTACTCAATTTTTCACATAAGTCGATATAGTTTTTACAACAAAGATCAATGTAATGTATTGAATCAAATGGCTGACATTTTGCTAACTTTGAAATAACCATTACAAATATAAATAAATATAACTAATAGCATATGTTTGGCGGCTTTAACATCATTAATTTGATGGTTCTTCTGGGAAACACTCATACGTCCAGCAAATATATTATTAGTAACATATAAATGATTATTGATTAAAAAAATAAATAGGCTAGCTACTTATTTTCTCAGTTTGTTGATTCTATGTTGGTTTATAAGTTGGTCATTACACCAACTCGGGCTTACAGTCTCGCCCTAATACTAGAGATTTCAAACAGGGTAATAAGAAATTTTCGTCAACTATCTGATCGATTCTGGAAGGCCAGCTTTAGAGATGGATGCTTGGAATACCTTCATATTGGGACATTGAGTGCCTTTGGAGCTCCAAAGGGAATTTCATGTGATTTCCGGCGAACAACAGTATTTAAAAGGATAAATGACATTATAATAGAGGGATTTACATTTGTTGGTAGAGGATACTCTTTTCTAGCATTCTCAAATTCTCAATTAAAAGATCGGTCTTCATGGTTTTTCGTTGATACAGCTAAAATTAAAGTGAATGACATCATAATGTGGATGAGAAAGCTTCAGAAATATGAACGCTGCAAAATGCTCCGCGAGGATGGGCAATGATTTTCGGCGACCTATGTAACAGTTGAACTCGAGATGAGGTTGACCAGAACCTCCCTGACATTAAAAAGTATGGGTATAACTTTTCTGATGGAATCGGTATGATTTCATCA

>*PhAGO4* promoter region

CAATATCGCGAAGTGGCCGCCCCCTTTTTTTCCAGGCATGTTCGGACATACGGTTTCCGCGGTCTTTCAGCTGACCTTTCCAAACCATGCTTCGCAATCAAGCTAGAAAAAGACTAAGCAAGTGCTGTCCTAATGACCCCTGGAAAAAAAAAAGCTGAAATCAAAAAGAAAGAAGAGAAATTGGGCCATCTTTCCCGAGAGAGGCCGCCTTCTCTCAGGCCAACTTTCTACTAAAGATAAATACAAATTAATAGTGCACTACAAGTCTACTACAATATACTCCGTGTAATTTAGGGAGAGTAAAGTATACCACTTTCTTATGAGAGATAAAGGAATCATTTTTTCAAGATTATAAATCTTACTCTTTATGAAAGTAATATAAACTCGGCTTAAGTATATTTTACTGTTTACACTATTAAGTTGTATCGTCTTTCTTAAGCCGATAGTCAATTGAAAATATACTCCGTGTAATTTAGGGAGAGTAAAGTATACCACTTTCTTTTGAGAGATAAATGAGATGTTTTCAATTGACTATCGGCTTAAGAAAGACGATACAACTTAATAGTGTAAACAGTAAAATATACTTATATGGAGAGTCGAGTTTATATTACTTTCATAAAGAGTAAGATTTATAATCTTGAAAAAATGATTAATCAAATAGACCAATATTTATATTCATGTCTTACTCAACAGAGTTTTAGTGTTTGACTAAAACCCGGTGAAGATAGATGTGTCAGGATCACAAATTAATGTAATACACAATATAATTTTATAGGTGGAGTCTCAGTAAGGTGAGGTGTATGTAAACCCTGCCCCTACCTCAAATTAGATACAAAGGCTATTTCTGAAAAATTTTCGACTTAATAAAAATTATAAATTTCTAAACGAGTTAGATAAATAAGGTAAAATATGGGTAGTCTATGCATATAAGTTAAAATTTTTTTGCAACATACATTTACCATTATTATAACGTTAATTACACTTGCACTTTTTAAGGCAGTAGTTCTATGCGCATTAATATAGTACTAATATTTGTCATTATAGTGGTAGGACAGGTGGACCTCACCTAAAAGGAGAGAGAGTGTGTGTAACTGATACATATATACTCCTAAATACTCTCACCTTCTCAGTCCACTCTCTCACAAATTCTGTACTTTTTTTTAGGGTTTCTTGAATTCTCCTCCCTTTCATCTGCCTCTTTTCAAAACCCTTTAGGTATATTCCTTATTTCTAATCTTTTTTATAATTTTGTTGAAGTAATTTTTTGTGTTTTTTCTGTTAATAATTCAGTCAATGTTGTTATTTTAGTTTAATATTTGTGTGTTTTGTTTCTTTCGACTTTTTTTGCTGAAAAATTTGTGGGTTTTTAAAATATTTGTTTTTTTCTGTTCATGAATCAGTCAATGTTGTTATTTTAGTTAAATATTTTATGGGTTTTCTTTATTTTGTAGTTTTACTCTACTTTTGAGTGATTTTGACTAAAATAAATTATGGGTTTTTGCTGAAATATGGATTAAATGGTTTGTTGAAGTATAAAATGTATTTTTTTGCTATGAATCAGTCAATGCTGGTGTAGTTTGTATGATTTCTGTAAAATATTTTAAGGGTTTGGTCTTTTTTTTTGTTTTTTGGTGCTGTTTTCTGAAGAAAAATTTGGGTTTGGTTTGTTAAAAACCCTGAACTGAAAAAGAAAAAGAATAAATGAGAAAATAAAAGACTAAATTTTAGAGTGAAATATGAGGGTTTTGTTTTGTATTTTTATATCTTTTTAATTTTGTCTTGTGAGTTTTTTTTTGGTTTGCATGTGTGTTTTATGGCTTGATTTGTGAGATTTTGACCTTTGTTGACCAGAGTTCTGTGAAAATTTATGCTTCTTTTGTGTTTTTGCTGAAATATAGAATGTTTTCAAAGTAGAGGGGGAAAAAATACAAAAAAAAGTTAATAAACAGAATATATTTATGGGATTTTGACCTTTTATTTTTACTTGTAGTATATACCTTATGGTTGGTCTGATT
