# Supplementary material for: Transcriptome Profiling Reveals a Petunia Transcription Factor, PhCOL4, Contributing to Antiviral RNA Silencing
Source: Front Plant Sci. 2022 Apr 14;13:876428. doi: 10.3389/fpls.2022.876428 (PMC9047179; doi:10.3389/fpls.2022.876428)
Supplement: Supplementary file 11 [file Table_11.docx]

**Supplementary Figure S2** Sequences of novel unigenes in petunia leaves inoculated with TRV PPK20.

>XLOC_034819 transcript=TCONS_00057243

TACATTCTCTCTCTCTCTCCTCCCCTCACAGGATTTTGCGGGTTTTGATCTGCTCAGCTA

TGAAGAATAATTATTAGATTGAAGATCCTGAATTTAAGAAGCTTGCGCCTGGTTCCATCA

GCGTCCTTGCTTGTGTTGCAACTGCTGGAACAACACTAATTCTAGGTGTTGCATTTCCTA

CCTTATTCTTGTTTGTGAATTTCTTTGGTGTTACATTACCTTATTCTTATTTGAAACCAA

ATTCGTCTTTACTTTACTGTAAATGTTAGTGTTTCAGGCAAGTTTATGCTTACCTAGAAT

AACTGACAGCATCTTTAATTCCTTCAGTTTCTGTTCAGTAAAGATAAAGTTTGCAATTAA

TTTATTGTCTGATAGATGCTTTAGCAATTCCACATGACTTGATTTTAGTTTGTTAATTGT

TCAATTTCGCTGCTTTTTCATAACTTGGGATATGCTATACCTTTTGTGGAAAATGTTCTA

TGGTGTTGATATACCAAAAAAACTTAAATCAGTTTGGCTTTGCTCCCCTTGAGCCATTTT

TATTCACTCTCAAGGTTGTCCAGCATGATTGGGTGGAGTTCTTTTCTTTAATTTTTTTTA

AAAGAGTTTATCTTTGACCCTTTGTGAACATTAGGAACTTGTCTACACTTTTTCCTAATA

ATGTAGCCTAGATAATGCTGAGATGTTTTTGGTAGTCTAATGAGGTTGTAAATAAAAATA

GATACCTTATGCCCTGTTTGGAGTTATCCGTGTCCTTGTTCTATGTTGACTCCTATGGTT

GGTTCATACCACTTGAACTGTAAAGAGATTTCTCCCATGATATGTGCATACCTTTTTGCT

TTCTCTGTACTCTCTCCACAAAGTTAATTAATGATTTGTGAAGGACAAAAACCTCATGGC

CAAATCCTTAAACTTATTCTGAAACTTACTTGGAAAATACTTCAACGACTAAGTTTTCAA

GCCCTAGCTATTAAACTAAGTCTTTTAGCATAAGAATATTTGTTTGTGATATCAACCTAT

TCCAATATCTGTCAGTTGACAACATTCATTTGTTACTCAACTTAAGTGCTTGCTTTAGAT

GGGAAAAAGTGTAAATGTGTTACCTGCTTCAATCCTTGTTTAGGATAGTAAGTTGAAATC

ATTTCGTTGAGCCTTTTCTTTATGTTAAATTTACCAAACGCAGTGACATGTGATGAATAT

TATCACAACACCACACTTTTAAGGTGGCGCACATCTGTACTAACAATTTTTCTTTCTTCC

GAGGTGCTACTTTGCTAATGGACGCTATCCAGAGGAGCTCAAAACATGTCGTTTTTCTTG

AATGTACAAGGACACTAATTCAGAGCATTTAAAATAATTTAGTCTGGGAAGGACTTGGAC

TTGGAGGAGCATCTCAGTTGGAAACTAGAAGCAGAGGTCCCTACGACTAGTGAAATCCGA

AAGCAACAGAGGTCCCTGCCATTTTTGTTGTGAAAGTCATCTCCAACCTTACTTAAATGT

GGATTGTAATAGTCAAATAACTTTCTTCTTTAACAGATATTTGTTATTGTGTTTATTTGT

AATGGAATTTGATTGAAAT

>XLOC_013219 transcript=TCONS_00021669

AATTCTCTGGGTGCCTTTTGTTTTCCCTCAATTTTCCCCAATTATTACAAAACCCTTTTT

CTCATCTCCCTTATTTTCTCTCGAGATTACTAATGAATCTCCCTCTACTAATAGTTCCAC

GTTCTCCATCTTCGCCTCTCTCTTTGTTAAAACGAAGCGGAACTCAATTCGAAGTAATCG

AAGGCCTGAAATCTACAAAGGAGAACGCACAATAGAGCAACAAATTTATCATTGGAGCGA

GAAACTTTATTGTCCTTGTTGGCGGAGGCAGAGGAAGTTGTGGAAGCCCTATCCCTCTCC

TTCTCTGATGAACTTTGCAAATGGAGATATCATTTGACTACATTGACATTTTCAATTAGC

CAACATAGTATCATCCTGACTTAAAGGCGTTGGTTGTCAGGCTTGATTCAAGTTCGTTGA

AGGACAACTGAGGCATGGATACGTGGACCACAGGCATCAAATTTACTAATTTTCAGTGCA

AGCCTTATCCTAGTCAGGCCTTACTCCCTAGTGCTCATATATTAATATTTTAAATATGTG

TTGCCTTAATACAAATTTATAAGTGAATGAAAAGACACATCAAAATTTTAGTTAATTTCT

ACACTTCTTAAATGTTCTTTCTGGTTGAGTGATTAGCAAACTACATGACTTGCAATATTT

GAACTAGAAACATTTGCCGCTTTATGTGAGCAGCTTGGTTTCACATTTAACCAAAGTACT

TTTTGCGAAAATGGTTTAATTGGATGTTCATAGAGCCGTTTATCATTCATTGTATAGCAT

TTATGACTTAACCGGCTTGTTGCCTTTGTTTCATTTGTGGAGTAAGAAGAAATGAGCTTT

ATTTTCGTTCTTTTGTTAATTACATTCAGATGACAACTTTAGTATTTATAGATTTCTTCC

TGGATTCTCTAGCAATATCAATAAGCTTATTTTTCACACTTGTTGATGTAGAATAGGATA

CATCTCTTCTCCCAAATGGTACTCATCAAAGTGAAATATTTCTAGGATAAGATTAGTTTA

TAGAGTGAAGAAATTCAATTCATCTATTAGTGTAGAGGGTTTAGTGTATAGAAGACCTGC

ATAGCATGTAGCTACTCTAATTTTCCAAAGTAATAGTTTTTGCACTTGATTATGAGGCAT

TGATATATGTAAAAAAAATTCTTCAGTTATGCCTTAGCTTTTTTATCATAGTGACTGAAA

ATGATAGTTGTTAGAAAATTATGGTTATCAAAATAATCCTTTGCATTTTTTCTTAAATAG

CAGGGAGAAGATCTTTCAACAAGCTGAGAAATATTTTGGGTAATGAAGAGTCCGAAGATA

TTTTCTTTTTTTGTATTTATACACTATTACAATGTATTATGAAGTAGTATACATTTTATT

GTATAGACTATTTGGTAATGAAAAGCTCTTATACCATTGTTTTAACTGTAATTGGTGGTA

CTTATTGTAATTATAGTATTGTTGGGCAGTTACTAGACATGTAACTGATCTTTTTTTTTT

TTTTGCACCAAATAAAAGTATCAGGGGCAATAATAGTTGCCACAAAATATGTATACTAAA

CTAAGTACTATTAGTGGCAATCAAAATGTCGCTATTGAATGGCTTTTGTGGCGACAAGGA

CGTCACTGAAGGCATGTTTATTCGCAGCTACCCTTCTTGGATTGACTTTCTTGGTTGTTT

TTGGCGGCAACTGGGGTCGCCACAAAAACCTTATAATTTGTGGCGACTATTTAAGGTCGC

CACTAGTAACTTTCAGCCACTGATTATAGCCAACGACCCTTAGGTCGCAACAGATAACCT

TTTTGTGGCGATTTTTGTTGTTTCAGTGGCGACTTTAGTCGCCACAAAAGACCCGATTTC

TTGTAGTAAGCTGTCTTCGAAACGTTCCGATATCAACACAACATAATCGATCACACCGCC

AAATGAAGCTGCTTTGTGTCGATCTCACCAGGTATGTCTTCGAAATCGGTTATAGCTTTG

CCACATAATCGAACACGTCCTCTTCGTGATCGAAGAAAAATCGATCAATAAACTAGCAAA

ACCACCTAGCAAAGCCAAAATTCATGAAACTATGAATGGATCTCGAGCTTTACGTTAATC

ACAATTAGTAATTAGTAAATTAACTAATCAGAACATATATGTTTCTACCTTAAAGTAAGA

TAACGAAATAAAGCATTAAAGATGTAAAAGACGTGAACGAAAATGAAACTTGACATGTTT

AGATTTACAGAAGCCTATACAGATGTGGAGCTTCAAATGCTAGGAGATTCCATAAAACCT

CCTTGTCCATTCTTGGAGGATCTGCTCCTTCTTGGACAGCCA

>XLOC_023822 transcript=TCONS_00039169

TGAGCGGCATATGATTTGATTTGTTAATAATTTTTAAAATATAAATTTACCCTTTTCTTT

TTCTAGAAACATATGAAACTAAAATATTCAAGATACAACTGTAAACTTATTACTTTATAG

TTTTTAAAAATAAACATATATTTAAAATAATATCTCTTATTTCCAATTTTACAAGTACAA

TAAATCAAACACTTATCTTGATAATACAAGTACTACTAATCCCTGCATAACTTGGATTTC

GAACTTAGCATCTCTATAAAAAGAACCCATAGCAGCTGCAAAGTAAGAGAGAGAGACCCT

AGAAATTTCCGAAATAGAAAAAGAATGACACAAAAGTTGAACAAGTTCAAGGGCGATGTG

GGAAAATTATTTAGTGAAGCCCCTAAGATACACAAGAAGGGGAGATCCATCGCAAACAGA

AAAAGGGTAAATCGCAATGGAGGAGGAGGAGAAAAGTTTGATCTTTCCATATGCTATCCC

TACAACTTGAATCAAATCTCTAAAGGCAAGTGCGTCTCAAATAACACTTCACAGCACCAG

ATGCAAATGGATATTGATCAGGAGGCCATGGAATTTTACTTCGAAGAGGAAATATTTATG

GACATCTTCAGCAGGTTACCCGTGCAGTCTCTTCTTCGATTCAAATGTGTTTCAAATTTT

TGGAATGCATTAATCTCTGATCCTGGCTTTAAGATGAAGCATCTCAACTATGCCAAGAAT

GATGAAACTTCCCAAAAACTTCTTGTTGCCCAATACTGCAATGGCAAGAATGATAATTTG

TTTGCCTTCTATTCTTCTTCTTTATCGTCGGTTCAAGTGATTGACGATGAAGAAAAACTT

GATAGCCCTTCAAACTGCAAACCAGTGTCTTGCATTTTGCATTGTTGTTGTGATGGGTTG

GTTCTTCTAACTGTTACTGATAGACTCGATCGTCAGCTTTTGCTGTGGAACCCCTCCACA

AGAGAATCAATACTACTTCCCCTACCAGAGTGTACGAATTCACAATCTACACTGCCGGAT

GGTGTATATGGATTGGAATATGACGTGACTAGTGATGACTATAAGATCCTTGTGGTTAAC

TTGAATGCGGTTTCTGCTGCTGTGGGAATATGCATTGAAATTCTTGCGCTAAAAAGTGGT

TCCTGGAGAAAAATTGGTAACCATCGTAAGGACATTTGCCGTCTGGATGGTTTTACAGAT

TGTGGTATGGATTCTTTGGCATTTGTAAACGGGGCATTTCATTGGCTTGGGTTGTCACAA

ATCCAGATTATCATGTTTTCATTTAATATGTCAAATGAGGTTATCGTAGAGATACCGTTG

CTAAAGCAAATGTTGGATATATGCAGTGGTTCTCCCTCATATGACCATGGTGTTTCAGCA

TTGAGAGGAACTCTTTGCTTTTATACCACATACCGTGGTCCGATGGTGACCACTTTTAAG

TTATGGGTAATGAAAGACTATGGTGTTGAGGAATCTTGGACTCAGTTGCTTACAATAAAA

GGTGCTAACATTTTTCATACAGCCAAACCAAAATATCTGTTTGTGGATGATGAAGTGCTA

CTACGCTGCAAATATATGTCTTCTGGCTCTCAATTTTTGACATCTAGAGGACCATTTGAT

GCATACCATCGGGCTCATGTGCTTGAGCAAGGACTCGTTTATACAGAGAGCTTGATATCT

CCAAGATCACTTATTTGATATTTACTTGTGTATGTAAATAAAGTGATTAATCTAATTTTA

ATCTAACTAAACTGTAATTAATCTAATTTTAATAGTTGAAAATAAAGTGATACTTTAATT

ATTTATGTATCATTAAGTGATAACTGAACAAAAAC

>XLOC_020919 transcript=TCONS_00034349

GTTAGGTAAAATTTTAATATCAAAATCCATTCTTGCATAAAAAGCAAATTATCCTATTGT

CTTTACTCCAATACACCCATCACTTTAAATTTGAATGATCATTAGTATTCTTTTTCTCTT

CATCTCTCTGATCCAAACACTTTGTTCCGTCACAAAATTGGGACATATGCTTAATTTGTC

TCTCATAATTAATATCAATTCCTTATCCTTTTGGTCTGAACCAGTACAATTCACATATTA

GTCTTTCAAATTTATTCTCTTCAACATATCATAGCTGGTTCTAGTTCATATATATCACTT

TTAATACTCACCCACCACAACACATTATTTGTTCAAATCATGTCGAAACAACTTGGAGAA

TTTCTTCAAGAACAACAAGAACCATTTACTTTAGAACTTTACCTTCTTGAAAGAGGCCAT

CTTAGAAACAGGCTTAGTTTAGATGGCGATTTTCGGTGTTGTTCTGCAAATTCTACTAGA

TTTTTTAACAGATCAGCTAGTTGTGGTACAAAGAAGAGAAGAAAAGTCATTCCTAATTGT

TCAAAAATTGTAAAAGCTGTGTTTAACAAGCTTGTTACAATTAGCCATGACCAGAAAATC

AAGAACTTGGCAAGTGAAGAGCAGAACAATGCTACAAGAAACAATTGCCAAGGCAATATT

ATTAATGCAGATGATGATAATTTTTCTTCAGCAAGTAGTACTACTGTGTTCAATTCTTGT

TCAAATACTAGTGATGGAGAAGAAGCTGATAATTTATCCGAAAAGGGTGATACATCACCA

CATGCTGCTGATGATAACTTTTTACATGACAAACAGGTTTTAGAAGACAGAAAGCTTAGA

TTGGAATATCTTGAAGAAAGTAAGCAACTCAGCCCTGTGTCAGTGCTAGAAGAAACTGAA

TCTTCTGATGAAGACTCCCCAGAACTTGATTCTCAAGAATGTCACAAGGCAAAGAAACAA

GAGGAATTTTCAACCCCATCAGTTTCTAAAAGAAGGGAAACAAGTCCTGAGTCTCAGTAC

ATTATAAACAAGAAGGCCATACAACAATCAGGACAGCTACTTTTTGAATGTTCAAAGGAA

GTGGTGGAGAACCAAAGGAAGAAAGATCCTAAAGAAGAATTTCAGAGGATATTGGGAGCT

GAGCAATTATGGGAGCTTTTATTAGAGAACATCTGGTTATGGAGCCAAGATTCCATAAAT

GAAACTAATATTAACCTCTTAATGCATTTTGATCTGCTGAATTCTTTTGAACAGTCAAGT

GGACTTGAGGAGCAAAAGGAAATAGGTAGGGTGATAAGTGACTTAATTTTTGCAGATATT

AGCAATGAGATTGTAAAAGACATGTTCAATCATTCTTCATCTTGAAGAGTTTAGAAAAAG

GTTATAATCACAGACTTTGCTTTCTTAAGCGTCATTTTCTTGTTTTAATTAGTTCTTCCA

ATCTTAGTAATGAAT

>XLOC_005312 transcript=TCONS_00008652

TAAGTTTGGGATACATGTTTCGTGTGCTAGTGCAATAGAAGAACCCAAATTGATAAAGGG

CTGTCATAAGCAACGCCATCATTCCCTGATTCACCAGAAAAGGCCTGCCACTTTCCATTG

CGATGCTTGTGGTAAGAAAGAAGCTGACACTGAGCCTTCTTATGTATGTACTGAATGTCA

ACTCTGGATCCATGAGACCTGTGCATCTTTGGACGGTGAAATCAAGCATAACGACCATGA

GCACGACCTCTCCCTGGAATTTTTCCTTCCAGAAAGATTTCAGTCTTTCAAACTCTCCTG

CGAAGTGTGTGGCGAAAAGCTCCATTTTGCTTACTGGTTTTATCATTGTGGGCGCTGCAG

TTTCTTTGTACACGTCAAGTGTGCCGGAAAAAATAGAAGGGTAGCTGAAGACAACATCGA

AACTTACCCCAAATTGAACTTGGCTCTGCTGCCGGAGGCTAATGAATCCGTGCAACTTAT

AGAACGATTTCTTGAGAAAAATGGCATCCTAGAAAATCACATGCCACCAAAGCAGATTGA

CAACTCTCAGCACAAGATGCATCCAT

>XLOC_018114 transcript=TCONS_00029660

GAGGTTTTGAGTCCTAAGTTTCTCTTTTACCATGAGTATAAACATTCCTTGCAAATGATA

TAATCATATATGGTAGTACTGAGTTTGAAGTTACTCCTTATCTTTGTGAATGTCTTTTAC

AATGAAAGTATAGAAGTATTTTTATGAGCCTTAGATAAACTGATCGTGGGCATTAATAAG

TCCATACCCCTACGATATGATCATAGGTGACCCACGATCTCCCTCTCATTTAATGTGGGA

TCATGGGCTATGGCAGTTACAAACTTGGAGGGTCTAGCTGTGAGCAACACCGACGCTCCA

TAAAACTTATACACGGGTAGATGTTCACGGTCATTCCACGGAGTTAATCTACCAGTCGTG

GCTTGGCCCTCTATATATGACATGGTGGAACCAAGCTTCGTCCTTAAGAAGGTTTATATT

TGCATGCCACGTGTCCGCGACGAAATTAAACTAATACAAATACTCATAAGTAAATATAGT

AAATAATAAACTATCAAAGACCTCTCCTAAGTAAAATAGTAGTGAGGGTAAGGAAAAAGA

TATTCCTACCATAAGTGAATAACACAAAATATGCACTCCTTAATTAGTTGGGAACTAAAT

ATTTTAGGGATAATTACTAAAACAATTAGTCAATTCTCTCTTCTTACTTTTCAACAGCTT

TTACTTTTCGTAGCTAATATATACCAAACTAATAATATACAAACGATGTCATAGTGTATA

CAAACAGTCTCCTCACATATACATAATATTTACATTCAATTTTACTGTGTATGTAGTCAT

TTTTCTCATTTATACATAATGTATACACTTATTTTTTTCAGTTACATTCAGTGTTACACA

TTTATAAAAAATTGTGTTTATATTTACAAATAACACCATCTGCCGGAGAGCTTAGCTGGA

GCTGCATCGCCAGAGTAAGGAATGTATCAAATCTGGCATGCGAGAGAGAAGTTGGCAAGT

TCTTCTCCTTTTTTGGCGAGATTGACCACATTGAGATTCACTGAGAACACGGACAATCAA

ATATGCCTTCGTTGGAGCTGCAGGAGTAGTAACTGCTGCTTCCCCAAAGCAGCAATGGAC

TAACAGCGCACCAGATTCAGAACTTCCTTCGCAAACGGTGGCCAGATGGCTGGAGGGAAG

GGTGGCGGTGCCGGTGAGAGGAGAAGAAGAATGGGAAGACTAAAAAACCTTAGAAAAAGA

GAAGCTAAAAAGATAGGAGGTGTGGTTGAATTTTTATTTAGGGATATTCTTTAAAAATAA

TGACTAAAAAATATGTAGCTTAGGCTTTTT

>XLOC_030569 transcript=TCONS_00050284

GAAGAAGGCAAGTACTATTATGGAGTTCATAGCTGGCTAGCTGCAAAACAGATGTGTCTT

CAAACTTTCAGGTAAAGTTGGGAGAAAATGGATATACGTGGACATTAATCAAGTCAAAGA

AACTCCAATTTATCATCGTACAAACATTCGATCTCTGGCTTGTGCAATAAACATGGCAAA

ATCAACTGTTTTCCGGCATGTCAAAGACGGCCATTTTAGGGCTCATACCAATGCTAGAAA

GCCTCAGTTAATCGAAAACAACCAGAAGGCGCGACTCCGATTCTACCTCTCAATGATTCA

TCAGGGTACACTTCACTCAAATCCCCATGTTTAGAGATATGTTTAACTATGTTCACACTG

ATGAAAAATGGTTTTACTTATCCAAAAGTTCTGAGAGACTACCTGCTTCCTGAAGAACAA

GAACCTAATCCATATCGCTCTTAAAATTTTTATCGCAAAGGTTATGTTTATGGCTGCAGT

TGCGCGTCCTAGATTTTATGAAAATGGACTTGAGATTTTTTCAGGAAAAAATGGAATTTT

TCCCTTTGTAGTTAAGGAACCAGCTAACCGATGTAGCAAAAATCGGGCATCCGGAACTCT

CGAAACAAAGTCTATTTTGTCTGTTACTAAGGATGTCACTAGAGCTTGCTTGATAGAGAA

AGTTCTTCCCGCCATTAGATCCAAGTGGCCAGCCTCAGAGTTTAATACTCTTATTTTTAT

CCAACAAGATAATGAAAGACCACATATCGGAGTTGATGATTTAGAATTAAATGAAGCCGC

TCAATAAGGTGGATTTGTCATTAGGCTATGTTTTCAACCTAGCAGACCTGATCTTAATGT

ATTGGAAGATTTTTTTTAGGGCAATTCAACCTCTTCAACATCAAAAAGCCCCTTCAAGTA

TTGATGAACTAGTGGATGCAGTTGAAAAATCTTTTGATGAAATGCCAGCTAAACAACTTA

ACAATGTATTTTTAACGTTACAATCTGGCATAGTTGAGGTGATGAAAGACAATAGTGACA

ACAACTACAAAGTGCCTCATACGGCAAAAGATAGACTAGAAAGAGAGGGGAGTCTTCCTG

TTCAAGTGTCTTGTGATATTAATATTGTCAATCAAGCTTTAGCTCTACTTCAACAATAAT

ATGGGTTATTACTATGTCAAGGCTATTTACTTTTAATATATTTTGGCGTTTCTACTGGAC

CTTGCAGATTTGACATGTTGAAGATGGAATGATATTTTGTCAACTTTGTTAGAAGTGGAG

TAACCTTTTTATGTTTAGCCTATGTTATCATCATGTAATTTTGAGGATGCATATTCCTAA

CAATGAG

>XLOC_021169 transcript=TCONS_00034794

ATTGATACTCTTTCAGTTTATGTGAACCTTTTGCTTTTCGAGAGTCAATTTGACTAATTT

TTAAAGCTAAATTGGATTAGGTTAATTTAATATTTTAAAATTAAAATTTAAATATTCAAA

AACTACACGAAAAATACTATAAGTTGCAACTCTTCTTATATCAATAGGATGAAAAACTTC

ATCTTAGAATGTTGGTCAAAGTTCAAGCTTTTTGACTCTCGAAAAGGGAAAAAGGTTCAC

ATAAACTGAAACAAAAGTAGTATGATTTTGTTTCCTTTATCAGGGATATATACGTCCATT

TTTTGCTATGAAGGGGTGGAGAACATTTCCCACATTGAGTCCTCCATTGTACGTGTTTTA

ACCTTATTTGATGACAACCATTATTGCCTGATGGCCGCATTGTCAGGCAGCAAATTATTC

ACAAAAGCTCTGATTAGCAGGAAACAGTCGTAGCAGTTGCCAATCATATTTGAGAAAACT

CTAGTGTAGAGCTACCAACTTCCTTGGCTGAGCTTTTGGATATTATTGAAGCTATTACAT

TTATGGAGGATCAAAGTTCCAACCTGAACTTTTCAGCCTTAAATCTGAATTTATGAAGTT

CCAGTTTGTTCATAGCTAGAATGGCTCGCGTCTGCGGGCATATCTCCCTCGGGTGGAAAC

ATCACCTGTCTTTGTAGCATGTTTGGCTATTCTTAAGCATATAACTAGTGGGGAATCATA

ATTAATTGTTATTGGAGCACAAATCAATCTTGAGATATGGACATTAAGCGACGTCGTTTG

TAGATGGATAATAGAAATTTTTTCGAGCGGCGGGGTTGAGGCCTTCGTTTGTAGCGGGTG

TTAAGGAATATCTTGATAATGTCCGTACAACCGGCGATTATTTAAGGCATGGCAAAATTA

AGTGTCCATGTGAAAAATGCAAATACGTAAAGACACAAGATTTGACGAAGGTAGAGATAC

ATCTTTATAAGAAAGGGTTTATCGTTTATGCCAAATTACTATGTTTGGTATAATCATGGA

GAGCCGTGGGATTTGCAGGAGCACCTTCAAAATCTAGAAAATATTCATGGCGGTAGGAGT

TCTCCGGTTGTGGAACCAGACCTTAATAATTATAGAATGGAGGACGTGGTACATGATGCT

TTTGGGATTATAAATATCGACCGTTATTCGAGGTCGCAAAAGGCGGTTTTTCTAGTAGTG

ACTCAACAGCGGGCAGCTTGTACTTCACTGTAGTTGTTAGTCAAGAATTGAGTTGGTTTG

ATGAATGCCAGGACCACCTCCCCCTAACAACACCTCCAGCTCTAGCGTTACCCTCCTTGA

TCATGCAACTCCCATCAGTGTTTATTTTCATGACATCTTGAGTTGGTGCAGACCATGTTA

CTTAAAATTTTCAGATATCCAGCACAGCTTTTCCCAGTTCCCTGCCTCCATCTTTTTTAC

TTTCTTTCTCATAGCCAATTTAACCAGAGAGAGGATCTGATGATCAGTTATTCTCCTAAT

TGTTGTTGCTTTTTGTTCGTATTTCTTGGCACATCTTGCCTTCCGTAACTCCCACTAAAT

TATGCTTGGAGTTACTTGTAATATACTTGGAGTTGCTTGTAATAGGAACTGATGGACTTC

AAGTTATTGACAACAGTATCTCCTCTTCTCATTTCCAGTACTTAAAACTTATCAGTGACA

AAAAAGTTCTCCTACTTGTCAACATGTTGTCACTTTTTCCCTTTGTTATCTCCATATTCC

CTTTATATCTCTTACTATGATCTTTATTTGTTCTTTTAACCTTAGGTTCAAGCGACGCAT

GAGTGTGCTAAATAGACTACAGAGTAGAGTTTTCACAAGATAAGTTATCTGCTAGTATGT

TGTTTATTTACCAGTTTGTCTGCAAATTGTTTTATCTTATGCTGTCATTCACTAGTTCTG

GAGAAAAGGGTACGGTGAAAGATTGGTGCTGTGTGCACATAACACTAATAATACATATCT

AGCATTAATTTCCACACCATTCTTATCCTATTCCAGGCCTTATGTATGTATGTATGTATG

TATGTATGTATGTATGTATGTATGTATGTATGTATGTATGTATGTATGTATGGTGATTGG

TTTCTTAAATCTGGGTCCAAATTCGTATTTCAGTAATGTCTTGTCATATGATTAATTGAA

CTTTTAAGAGTGACTGTGATCTTGCCATGTTAGACGGTGAGGAAAGTAGCAGAGGACGGC

GCTACAATTAGGCGTAGTGATAGAGACTCGTGATGAACTTCCGCTGTTCATTGTGCAAAA

GGCACAATTTTAGTACCAGTTCAGTATTGAGGTAATTCTCGACAACGACAATGGTTCTTT

GGTTTGTATCCTGAACAAGTTGATGACCTAGCCAAGGGCTCAATTTTAGATGGTTGTACA

CCAAGTGGAAAGCATGAAATGTTGTGCCAAGAACTTGAAAGGCCAGAGTTTGATACTTTA

GAAACCTTTATAAAGGTTGCTAAGTGCAGATCAGACCAATTATAAGATTTTAAACGGTTC

TATTACTAGTAGTACTAATTAATGAAGTAGCTAGAATTTAACAGTAAGAGTTGGAC

>XLOC_035724 transcript=TCONS_00058719

CTCTTACCTAAAATATCTGGTTCTTAGACTAAGTTTCGGAGTTAATTATAAAAGTAGCTA

GAAAAAAAAATTGGTGCCCTAATTCGCCCAGTTAAATAATATGTTAGGTGGTGACTCCAA

CTCATTACCCGTGAGTTACCCACTAAGTCGATCCCTATTTTGAACTTTAACCAGTTCGGG

TAAAAAATAGGATGCGACACTCGTTTATTACAATTACCTGATTTTAACAAGGCTTTTGAA

ATTAAATTTGACTAATCTGGTAGTGAAAAAGTGACTTGAGCTACTTTGAATTATTCAACT

TACGATAAGAAGTGATATACTTTTGTTAGAGCTTTAGCTACCTAGCTGCACTATTTATAG

CCTAAAGAATTTGTAACTCCTTATTACGAATCTTTGAGGCATCTAAAGTGTAAATGTAAG

CTTAGGAGAAGGCATGCTAAATGGGTTAAGTTCATAGTAACTTTTGCTTATGTGATTTCT

TACAAGCAAGGAGAAGACAATGTGGCAGCCGATGCACTTTCAAAAAGCCCGTGTTAGTGC

TGATCAAGCCGCCGAGCCAGCGTTCAAGCGATCGAGACCGCTAACACGAAGCTAAGCCAA

GGCTCTGCATGATGTGCATAGTCATTTAGTGTGGCCGGGAGAAAGCAACTGATAAGAATC

AAAACGCGGAAAGTAAAAAGGATAAAGAACGAGACGTAACTCGATCTCTAATCCCAAGTA

GCAACGGAGAACTTGTTCCGCTACACCCCGGGAGAAGGACAAGAACACCTACGCTATGCA

ACTACCCTCGAGTCAACTAGAACTCGCAAGTAGAATGGTCGGATAGGGGTTGTTAGGCAA

CTAGAATCCACTAGAAGTAACAAGTCCCAACTAAGGTTGGTTTTCATAAAAC

>XLOC_024062 transcript=TCONS_00039549

CGCACAGTAGAAGCACCAACACAGGTATGTGAAATTTTATTCTCCCAAATACATCCCCTG

CCTCTTCTTATTTCACGCTAACACACACACACACCCCGTTCTCTCTCCCCGTCGAACCCC

CTGCACCGGTGAGTCGGCGCCGCACACGCAACCCACATCGCCGCCGGAATCAAGTACTTT

CCATGGCTCCTAAGGCGACATCTTCATCATCTAGACCATCTAAGGCCTCCTCTCAGGCTG

CACCTAATGAAAAAAGTAAGAAAAAAGTTGCCCTGAGGGAGAGGGTTGCTGCCCCGCAAC

AGAATAAGAAACAGAAAGTGGCACCCCCCAAGTTTACAAGTGATGCTTCCAAGGAAAGGT

TTGAGATGGTAAAGAAGAAGAAGTATCTTCCAGAACATGCCATAGATGCTGACAAGATAA

GGAAGAAATACTGGGAGGTTTGGACAGATTTGGTAGAAAAACGTGAATGGAGTCCTTTCT

TTGCACCCACTGGCCCTGCGAACCTCACTAAGATTCATGAATTGTATGCCAATTGGGGTG

ACACATTTAGCGGCTCATGCACCGTGAGAGAGAAGGTAGTGGCAGTAACGACGCATGCCC

TTAATGATTTTCTGGGATTGCCTCAAGTGCCTGAGGGCATGGAGTGGAAGCTTCAATGTG

ATTACACTAATGTTCGTGAGGTGGTCTGTGGCAACAACAAGAGAGGTCCCAAGGGCAGGA

GCAAGCATCATGAGCTCCCGTATAGGTACTTTACCAAAGAAGCCCGAGTTTACTTGAATT

TTACCTTGCAAACACTTTACTAATGTGAGGCGTAGTCGGGTATTCCTCACTTATGCTTTG

ATGATGCACCTCCCTGTTGATGTTGGTAGTCTCATGGTTCAAGAGATGAAACGGACCCTG

AAGTGGCCCAGATGCCGACTCTTCTTTGCCAACACACTCACTAGCTATATGCAGAGGTTA

GGTGTCCCTAACCAAACACTTGATAAGAAGATGCCGGCTGAGACGAAGGTTGATAATTAT

GCCATTGTTAGGGGCCCTGACGTTTCTATGAAATTGCATATTGAGCAAAAATTTGATGTG

CTTGTTGAGTCCGACAAGAGAAAATGGGCGTTTTTCAAGTTCGTGGCAAGAAAGTTGGGT

GTTACTCAGGCTGATATAGATGAGTTTGACTCTTCCATGCCATTTAGTGCTGAGACGAGC

TCTGCCTTCGGCCTTGATGATACTGAGGAGTTCCACCCCTCTGATGATGAGCTCACTCTG

CTGGAGTTGGCAAAGGAGGATGACAAGAGTAATGATGGCAGTGATATTGATGAGCCAGTT

CCCGCCACAGCTATTGTTTCTACTGCTACTACTTCTATTCTAGCTGCCCCACCTGTGAGT

ATTGGTGATGATGCTGAGGATGAGCACGGCTATGAGGCCGATGGTGATGCTGATTTTGCT

CCAGCCACGTAGAGGGAGTATCTCTTTCACCCCTTGTTTTCTATTTTGTTTTGAAGCACT

GGGGACACTGCTATTTCTTAAGTGTGGGGTGGGAGACTTATTTGTTGGATGACTGTACTT

ATGTGGATATTTACTGCTATTTCTATTTGGTCAGTTGACTGTTTTTGATTTGTTTCTTGC

TTTAATTTTTGATGGGTCGGTAACATTCTGAATGTTATGGCCTGTAATAGTTTTAGTAAA

AGTAATATTCAGTAGTTATTAAAGTTTAGTCTCTTTAGTACTTTTATTATTAATAACTTA

TTGTAGTAATAACCTCTTGGGTTTTCTTGGCTTTGGTTCTTTCCCAAAGAGTTGTCCTTT

TTGAACCAAGCAAATTTAAAAAAAAAAAAAAATTTGTAAAATAATTGGAAAATACAAAAA

GAAGAAAGGACGATTAAGAGAAACAGTGGCAAGAGAGAGAGAGAGAGTTCAACCCTGAAA

AGCAGTATCTTGTGAGAATCACCTATTCTGTTCTCGGACCCCTTTTCTCAACTCATAATC

GTTTATTGAGGATTAAAATTAAGTGAGTCACGGATTCCATGTACATGGTGCGGTGAGGTT

CTTTTATGTTTCCGTGTATGCATTGCAGTTTAGAACTTGCCCGGCTTGTCCTTGAAGCGA

AATCTTAGGATATTCTCGAGTTAGGAGATTGTAGGCCTTGTTTGTTAGTCACTTTATCCT

AAATAGACCTACCAATGATTGTGAATTATCCTTAGACAGGCCCTTTGAGCCTGAAGTATA

CCGTTTCTTTGATAACTCAGAAAGAAGCACTTAGCCTCTGAAATTGATTAGCCAGTTTGT

CACCCTATTCCCAAATGGCACTCGCTACTTGAACTTGGTAAGAATAACTAGTGTATTGAT

GAAAATGTGTTGGAAGTTGAAAGGAAAGAGGCAAGAATCATGTCTTAATTTGTGAAGCTT

TGCTAAAAAAAATGAAATAAAATTAGGTGGTTTGTGAAGTGTGTGCACAATAAAAAGAGG

ACGGAAAATGTTTGATGTTGGGGCGTGCAGTAACAAGAAAAAGAAAGTGAAAGAAAAAAA

AAAGAAAAGAAAAGAGAAAAAAATTCAGTGTCTTTGGGAAGTTACAAGAGAAGTGGAAAT

GTGGGAGAAGAAATTAATCTGATGGTTGTTGAGCTTGATTGTTTTGTAGTGGTAGTGCCG

TAGGAAATATAGTCACTTTTAGCCATATTATTACTTCGTCCCTAAGCCTACATTACATCC

AAATGACAAGTACTACTTGATCTTGACTTGAGATGCCTACATTAGTGGAGAGTGAAAAAA

AGGGCAAGCATATGGTACTATCTTGTGTGCATGTGAAAATCTTTTCTGAGTGTGAGAGTT

TCTCTTGATGTTATGCCCTGTTTCGTTCATTGAATACATTACATGCGTGATAGCAGGCAA

TTCTTTGATTGTGAGGTACATTGGAGTCTGTGCAGTGCAAGGTTTTACTTCTCAGTACTT

GATTATGAGCGAAAGAATATGTGTTTGAGTCTTCTGGTTGATATCTATACAGAACTGCCT

GACAGGATATGAATGATCTTGCTTGTTATCTGTGACTCGTGTTGAAGTTTTGTGTTTGCT

CAAGGACGAGCAAAAGCTCAAGTGTGGGGTGGAGATAATACGCCAAATTTATGCATAATT

GAGTATATTTTACCCATACTTTTATGTGTTCTAATTACGTTTGATGTTATGTATGACTCG

AATTAAGCTAATATGTGTATTTTTATGTCTAGGAGGCTAAGGATGTCAAAGGTAGATGAT

TTGAGCGAAAATGGAGTCAAAATGCAAAATCTAGAGGAATTTAAGGAGTTGGTGCGTCGT

ATGACCATAGTGCAGTCCCGAGACGAAATTTGGATGAAGAAAAATGCTTTTGAGGCAGTG

AGCTAACCCCTTATCGCATAGTCATCGCACGGGAGAAGAGAAAAGTGATCTTGAGGTATT

TTGGAGTAGCTGTGCGTCGCACAGAATAAAGAGTGAAGTTCCAGTATCAAGGTGCTATAG

TTGTGCTTCGCACAGCTATAGCGCCAAGGCAAAAATTCCTGCAGTTGTGTTAAGCGGGGC

AAATTAGTCTTTTGGCTCAAGGACGTGATATAAAAGCAAAGAAAGACGATTTTGAGAAGG

AGGCTGAACCTAGAGAGAACAAGAACAAGGCTGAAGCAGTGGAGAACGAAGCTCAAATGA

GTTTTTCTTAATCCTTTCAATCCTTTCTTGTAATTTGCTTATTATGGTTGCTTATGTCAT

GAACCTTATGAACATGAGTGGCTAAGTTACCCTATCTAGGGCATGATGAAACCATTGTAG

GATGATGTATTGAGTATTCGTTAATTCAATCGAGCGTGACATAATTATTCTTGATTATTC

AACTATGTTTTGAATGCTAACAATTATTCTTGACA

>XLOC_000843 transcript=TCONS_00001370

AACATACTAACATGATTATCTGATAGGTACCTTCAAAAATACTTCTTCTAAACATCTTAT

AAATTCCCCTCTCCAAACCTCTCTATCCAAACAACAGAATTCTTATTCCTTTCCTTTTAT

TTCCTTTTATTTTCTCCTCTCCATTGCTAAGATGGATGGGAAAATTGTTGCTTTGTTTGG

ATTTATCTGCATTATCATTGCTGGAGTTGGTGGTCAAGCACCACCCACCACTCCTACCAC

TAGTACAGCACCTCCTACACCCACCACCCAAGCTCCTCCCACACCCACTACCACCCCAGC

TCCTCCCACACCCACCATCCAAGCACCTCCTACACCAACCACCCAAGCTCCTCCCACACC

CACAACCAGTCCAGCACCTCCAACACCCACCAACACCCCAGCACCTCCAACACCAACTGC

CCAAGCACCACCAACACCTACTGGTTCTCCTCCTCCAGCTAACACCCAAGCGCCTCCAGT

TCAGCCACCACCAACTGCTTCACCACCCCCCACAGTTTCATCACCACCACCTACCGTAAC

TTCACCTCCTCCAGCTGTAACTTCTCCACCACCAGCTACGACACCTCCACCCGTTGCTGC

AGCTCCTCCTCCGCCAGTGAACTCTCCACCACCAGCTAGTCCACCACCTCCAGCTACCCC

ACCACCTTCACCTGCACCCCTTACATCTCCTACGTCAGCTCCTGCACCAGCCAAGAAACC

ATCATCTCCAGCACCATCTCCTTTGGGTTTGAGTCCTCCATTGCCTCCCACTGGTGCTCC

AACTCCAAGTCTTAGTGCTTTATCTCCTGCTCCATCAGCCACTGATCAGAGTGGAGTAGA

GGGCCTGAGGTCGTCAAGAATGATTATGGAAAGCATGGTCTTAGGATGGGCACTACTTTG

CTTTCTAATGTAGAAAGAACTCTCTTTTACACTGCATATCATTTGCTTTTTTGCCTCTCT

CTATATATGAATTGTCATGATGTCTGTAGGAGATGATGGCCCATTTCTCTACTTGTACTA

GGAATATTATACTACATTCACTTGGTCGATTTTTTTTTTTTTTTTTTTTTTTGATCTTGT

ATTTTGAGGGGATTGTTTTTGATTGAAATTTTTATAATTCTATTATTTCATCTATTCGTG

TATCTCTCTATTCTCTCAAGCACCCAACCCCTTTAAATTCACAAGTTGCCTCGTGCTAGA

TTTCTGGC

>XLOC_014071 transcript=TCONS_00023043

TTTTATCGAGCAATTGAAAAGTGTGCAAATCATATCACGATTCACGTCTTTGTTTTTGGT

TCATGGAGAAAAAATGGACATGGATTCGAAGGGATAATGCTTGATAAGTCTATGGTACTG

TACTAATTTCACATGAACATTGATATGTTAGTCTTGATGAAACGATTGCAGGAACACATC

TTGATTGACATTAAGGTTGTTGATGTTTCTTGCCATGGGAAAATGGATGCTAGAGAAAGG

AAGAGTGAGCATTTAACAACTGACAATACAGGAAGTGGTGTAGTTTCTGGAGCAAAACCA

ACAGTAACTGAAGCCAAGGCATGCATCTCGACTTTGATCCTCTTGAAGCTCTCTGTGATT

CATACAGGAATATGGAGCTAGTTCATTGCCATCAAGATTACACCTTCATTATACGGTAGG

TATAGCGTCGAATACATGCCTAATACTTACTCGGCAAAGGTATGACAAGCAACAGTTGCT

TCTGCTCAAGTATTTAATTTACCAGTTATAAGCTAGCTAACCAAAGAAAATTTATGGTGA

CATCTTACCTTGTCATGCTTGGACAGATTGGTTCGGCAACTGCAATAAGATACTCGCTGT

CCAGGAGCTACTCCTAATGGGCCAGAAGTTCTTATACTTGATTATCTAAGTCATCAACGG

AGACTTCTTCCCCATCTTGCGAAAACGGAGCATAAATGGTAGAGAAATATCATCGTAAAA

CTCTCATCATTTCAAGAAAAGGTCTTTGTGGATTTCACGACATAGTGACGGATGAAGGAT

GTTGTTACTGATATTGCTGTTTTTGAAATGCGTCGGGCTTTAAAGATGGA

>XLOC_022483 transcript=TCONS_00036954

TCTCTATCTATTTCTGTCTCTCCTTTTTCTTGACTTGTTCTTTTCAATCTTTCTGTTTTC

CCCTCAAGAAAAAAAAAAGAGTTGAAAAAGCAGATAATAATATTGATAAATCATTCCTTT

CTGCTACTCTTCCATCACTCTTTGGTTTGTTTGCATATCTCCTCTCTCAAACCTTAAGGC

ACTCTTCCTAGCTACTCATTCTTTTATTTTTAAAAAGAACTAGGATTCTCCTCTATTTTT

TCTCAACTTTATAACTTCAAAATGCTACCATGGTTTTGAGTAACACTTCAGTTTTCTCAT

TATTATATTTTTCAATCAAACCCACAATTATTCCCCTTTTAGTCCTCCTCCTTCTCTCTC

CTTCCTATTGTTTAGTATATCATACACATAAATCTCTAGACTCTACTCGGGGTTTGCTAG

TGGAAGAGAAAACAAGATTAGGTTCAACACCACCAAGTTGTTACAACAAGTGTAACCAGT

GCCACCCATGCATGGCGGTTCAAGTGCCAACAATTCCGAGTCACCACCGAGTTGAACCGA

ATCAAAACCAACGTATTACGACTATGGACTATTTTGAGTCATCACCAGCTGGGGCCAACA

GGTACTCCAACTACAAGCCACTTGGATGGAAATGCCGATGTGGTGATCACTTGTACAATC

CTTAGCCACGCAACGTAGAGACAAAGCCAAAATTTGAACTAGCTCGGAATGCACTAAAGG

TTATTTGAGTTTTAAGGTCCGGAAGTTGATACTTTTTGGATTGGTAGATTTTTTATCAAA

CAAGGTTTGGATTCGCTAAACTCATAGCTCATATGCTACCTTCACCCCTGACTGCTCGTG

AATCTTCTTCTTTATTCTAACAGTGGTGTTCAAACCAATTTGTTGGCCATCTTGACCACT

ATTTTATCGAGTATCTTATTAGTATGCTTTCACCAAATAGCTTTAGCTCGTGAATTTTGA

AATTATTAAGATTTGTCTGAGTTATATATAGTAATTAACATGTGGAGGATGCTAGTATTT

TTTGTTTTAATTTTAATAGATTTTGCGCACAATAAATTGATCCATGTTGAGATTCTTGTA

GAGACAAGACATGTTTAATGAGGCTGTTAGTAGCATTTACTATGGTGTACACATTGATAT

CAATACTGATGTTGTACAGATCCCTTTTATGAGTGCTGACTTTAATGTAGTATAGCTTTT

CACAGAGTAGTATTAAATTTCTTTATATCGTATTTACAATTGTTTTGTGTTCGTTCTTTG

GATTTATTTCAGTACTAGGCTTGAGCACTCCAA

>XLOC_006961 transcript=TCONS_00011328

ACAAATAAAGCAAAAAAGAAATCTTTCATTACTTACAAATTACAATATCATCAACCTATG

GGTATAAAAGAAGCAATGATATGGTCACTAATGGTTCTAGGGATTATACGATTTGTGACT

ATGCAAAATACTAATATCCCTGTGAAATCATTGATTTGCTATCAAGCCCAAGATTTATTG

TCACCATGTCAACCATTCTTGTTAGGTTGTATAAGTAATATTACAACAGAGTGTTGTCAA

GGTTCCCAAACTTTGGCTAGTCTGGTTAAGAATAGTACAATTGATGATATTAGAGATTTA

TGTAAATGTCTTCAGCAAACTGGGAATTCTTCTAGTATTAATGTTAAGAAAGGAAGAAAA

CTTTTCAAGGTTTGTAAGATCAAGCGAGTCCCTATTGGCCCTCAAGTGGATTGTGACGGT

ATTTGACATGGACCAGCCGCAAGAATCGATACCTCACAGCTTGAGATTTGAGAAGAATGT

CCTACTACTTGCAATCTATTATGATGTAGTATACATATATATATATATCGGAAGTTTGGT

ATCTCAATTGACATAAGGTCAAGAAAGACTCTTATTATCGGTGCTATGAAATAAAATAAA

TATTACTGTACAACTCTTTGTCCACCATATTACTTCAACCAGCATGTCATTAAAAAGTTT

AGATTGTAAAGATGGATTTCTTTTATTTATCTACTCAAGTTTGAAATATCTCCTCATGTG

TAGACCCAAAGTCACCTAAAAAGTATAACATGTTAGAGAGGACATGAAATATTTTTATTT

ATATATTTTAGGTCTACTACATGCGCCTCCGCATACTTTGATAGAACATGAAAAAATTTG

GTTGACAAACAAGAAGGTTGTGTACGTAGGATTTGCCTATGTTAAAGTCGTATTGGAA

>XLOC_012961 transcript=TCONS_00021225

GTCCCAAACTCCGGCAACCCTGCACGGTGGACTGCGGCGACACTACACTAGGGATGGAGT

GGGCAACCAAAGTCGGGGATGCAGTGAACAAGGCGTTAAACAGCAACAAGGTGTTGAACG

TGGTGCTATTGGGTGGCTTTGGGCTGCTATGTGTGAGATCAGTGAACCAACAAAGAGACA

TTGGAATTCTGGAGGCAGAAAAGGACTCTTTGCTCAATTCTAATAAAGCTATGAAGAAGT

CCATGTGGGAATGGAAGCAGCAGCTTTTTGCCGAAGCTGCTTTACCCAACGCCATTTTTC

CATTGTCCAAGCTTAAAGCCATCTATGGAGAAGCCCAGACCACCTCACCTTCTGGTGGAG

ATGCTCAAAAAGGAGATGGAAAGTCACCTGCATCAACATTTGTTGTCTAAAAGATATTAT

AGGTTGTTCATGAAGAAGCAAGTGGTTTGTGAGGTGT

>XLOC_018239 transcript=TCONS_00029888

TAAAGAAACCCTTTTGTCCCTTTCCTAAAAACCTAAATTCAACTCAATTGCAAAAGCTCC

ACCTTCAGCCACCATACGAAGATCCAACAACCATTCGAAGATCCACCATCAGGCTTCATG

CAAGAGCAGTGACTTGTACTACTCATGCCTGAAAATTCACAAAATGATATAGTGGTTGAT

GAAAGTAAGGCTTCTAACTCAAATGCGACTAGTTAACCAATTCAATCAAAGGTAATTAAA

GAAAGGAAAGGTAGGTATATTGGTTGGAATCATTTTGAAAAAAAAAAATTATGATGCCGA

AGGGAAAGCTACTTTAGCAAAATGTATTCATTGACATCATATGTATTGTTTCAAGTCAAA

AGACGGTATGGCTTCTCTTCTTGGACGTATAACAAAGTGTCCAAAAATTCCTTGTAACGT

TGAAACGAAACAACAAAAATTAGCATTTCAACCTACTGCGGGAGAAAATAAAGGTGACAT

AACTCTTGTCAATTGGAAATTGGATCAAGAAGAGTAGGAAGACATTATGTCGTATGGTTA

TAGTTGATGAACTTCCTTTTAGTTTTGTTGAGAAAGAAGGTTTTAGAAACTTTATAATTG

TGGCACAGCCTCATTTTCGGATTTCTTCCCGTAGCACAATCACTAGAGACTGCTTTGTCC

TTTTTAATGAAGAGAAGCAAAATTTGAAAAAGTTTTTTATTGAAACAAAACAGAGAATTT

GTATTATAACCGATACTTGGACCTCTTTACAGAGAATCAATTACATGTGTATTACCACAC

ATTGGATTGATAGCGAATGGACAATGCATAAAAGAATGCTCAATTTTTGTCCAATTACTA

GCCATAAAAGTGAAGACATGGCAAATGGAACTAGTAGGTGCTTACGTGAGTTGGGGCTAA

CTAAGATTTTTACTGTCACTGTGGATAATGCAGGCTCAAATGATGTGACAGTAAGAGAGT

TCTCCAAGCAAATAACAAAGTGGGGAACAAATTTAATGAATAGCAGTTACTTTCACATGA

GGTGTATGGCTCACATCATGAATCTTGTTGTTCAAGATGGGTTAAAAAAATCTTCTTTGT

CAATAGAGCATGTTAGGCTAGCAGTGAGATACATTAGGTAATCTCCTGCGAGATGGAAGA

GGTTTCAAGAAAGTTGTGAACTCAATTGCAAAAAATCGTTATGCTTGGATGTTATGACCA

GGTGGAATTCCACCTACTTAATGTTGAGAAGGGCTGTTGAATTCGAAAATGCATTTGCTA

ATTATGTTGCTCGTGATATTGGCCTGAAACTTTATCTTGAACATTCTTATATTGAAGAAG

GAGTGCCTACTGGTGAACTTTTGAGTAATGATTGGGAACATGTTAAAAGAATTACAAAAT

TTCTTGAAATATTTTATATTCTTACTTTGAAAATTTCTGGATCACGTTATGTTACATATA

ATATTCATTTTCTTGAAATTTGTGCTATTGGTGTTTGTTTGAAACAATTGATGTCCAGTT

TGTTACATCTAACATGAATAAAATGATTTTTGTTTCATGTATTTTGGATCCTCGTTACAA

GCTTGAATTTGTTGTTTATGCACTTGACAAGATGTTTGGAGATACATCAGGTGCATCTGT

ATTGGCAGCAGTGAAGACACACATGACTTCAGTATTTAAAGAGTATGCAAAGTCCAATTC

AATGGGTAGCATGGCTGATGTATCTTCATCTTTCTCTTCATTGGACAATTCTAGTTCTGG

ACTTTCAAACAGTCAACTAACTCTCCAAAGTGCAGGACTTTTAGAAGCATTTATGCAAGA

ATTAAAGAAACATAAAACTGAGAGCGGAAGTGTGGATGCTAGAACAGAGTTAGATAAGTA

TCTTTGTGAAGAAACAGAGGATGAAACTGTTGATTTTGATATCTTGCAATGGTGGAAAAT

GAACTCTCCACGATTTCCTATTCTTGCTGAGATGGCTCGGGATATATTAGCTATTTCGGT

TTCAAGTGTCGCATCTGAATATGCATTTAGTATGGGAGGACGTATTCTTGATCCTTTTAG

GAGTGTTCATTAACTCCTAAATTGGTGCAAGCTCTAGTGTGCCTTCAAAATTGGCTTCGA

AGTGAATCATTACCAGTTTGTGTTGATGAAGATCTAGGATTTCTTGAGCAACCTGAAAAA

GGTTTAATATTATTGGTTTGCTGCCTGGTGTTGAAGCTTTCTCTTACATTAGAGGTGCTT

AAAGCTGTTAGATTAAAAGCTATTGTTTGCTGCTTCAAAGTACCACTTGACATTTATTGC

TCTTTACTAGCTACTGGATTTTGAAGCCATTTTTTAGTGCTCTTTTTCTGTTGTTTCAAC

TTCTTAAGCTAGTGTTTGCTGCACTTTGGAAGCTACTGATTTGCTGTGTATAAAGCTAAT

GAAATCTTTTTTTTTTTTGGATCTAGTATGTTGCACTTTGGAAACTACTGATATGCTATG

TATAAAGCTAATGAAATTTGTAGGCTCATTTACTATGTATAAAGCTAAGTTTTATTATTG

CTTGCTTGATTCTATCTAGACAGCAAGTCTGTTAGCAGGTTGATTAAATATTTAACAACA

ATGGTTGGTCGGTGCTTGATGTTCTCTGTGAAAAAAAACCATATGAACTAGTCATTGTTG

AAGAATTATGTGACTGTATATGTTTGCTACTGTTGTTCAATCGTATATCAGTAAAGGCGA

TATAGACTGAGAAAACCATAGATCTCAGCTTCTTTTGACAATAAAAAATTCTGGGTAAAA

ATTTATTTAAAGATGTTATAACTTAACAATAAACCGCTCA

>XLOC_018344 transcript=TCONS_00030066

TATTTACCACTCATGGAAGAAACACAAGCTTTGGTATTATCTCCTCTTCCACCTAAATCT

AAACACACTGCTTCCTTTGACCAAAACAAAACGAAAAAGAAAAAACATTTCTATGGAGTT

CACAGACAGAGTGAATTTTCCGTGCGGATGATTCTGCATATATAAAGGAGCTACAAATAA

AATATTCTATTCTTTTAAGCCTTTCTAGATAAAGTAATTTGATAGACTTGAAAATTTAAT

TTTGTAATTGCCCACATTCAAAGCACTTCGATCTTAACGGAATGCAAATTATTCTTAAAT

TCCTGTTGAAGACGCAGTTACTAAGACAGGTATGTTTTGGCAGTCATCCTAGACTTCACA

TTTTTGTTGGCATAAACAAATAAGCAGCTGTCAGAATGTTCTCCAACTAAAGTTTTTACA

ACAATTGTGGGGTCATCTGGTTCCTTTCGAGAGGCATAGATTTTCCATGGATATCCATCA

GAGCACTTTGCAGAAACTCTACCAGGTTCATTTTTTCTAAAGCGAATTGCTTGATTATTT

TTAATGGAATATGCTCTCAAAGCTGCTCTAAATTCTGCTGCACCACTAAATTCCATTCCC

AATCTAAATGTTGGATCAGTCATGTCTGTTGCTGCCCTAAAGATAACTTTTTCTCTCTTC

ACATCTTCCTGATCCACATCATCCTCTCCATTAAGACTTCTCATGTCGTCTGATTCATCA

TATTCAGATTGAATGTCACTGTTCTCATATTCTGAAGATGAAGGTGAATGCTCCAATAGT

AATCCTGCAAAACTGGTGCTTTTATTTTTCTCCAGCACTTTATCTCTCTTGTTTTGTTTT

CTTTTTTTCTTCTCTTTTGCACTTTATCCTCATTGCTTTCTGCTCCTTCCAAATGTGATT

CATCCAAGTTAATATTTGTATCACTGGATTTGCCAATTCGATTGTTTTCATTATTTTTAT

CCAGCTCAGTAAATTGGACCATGCTATCTTTACCAAACTCAGTAAAATTCACTTGGTTTT

GTACTTTTGGCATACTTTCAGGATGTGTGGTGAATACGTCTGCGACCCTATTGGCTGGAA

GTGTTTTCACCATTAGATTGCAATCTTCATCAGTCAAGCATTGGATGTAACCATTATCAA

TATCTGTCCCTAGTAACTTGAAGTGCCAACCTAGTAACCCAATGTACCCTAATTTTATTG

TTCCAATTGAGTCCAAGGTATGTAAAGACCATTGTTCAGGATCACAATTTTCAATGTAGT

CAACCTTACCTCCTGTATAAATCTTTTGTGGTGTATCAACAAATTTACC

>XLOC_010518 transcript=TCONS_00017114

GTAGTATTAAAAAAGGAGAAAAGAAAAAGAAAAACATAAACATAGTTTGGTGTGATGGAG

GGGAAGAAAGTGGCTAGGCCGAGAGGTGTGCGGGTTGCGGTGCCCGTTATGAGTTCAGTT

GCTCGTTCGCAACAGGAGGAGGATTGGAAGTCAAATTGGAAAGGAGGAACCACCGCCACC

ACCACCACCACCACCGTTCCAGTTGATAAGCGTCCTGATTTAATCAAAATTTCGGAGGCT

CTCTATGTTGCACAAGAGAAAGCCAGACAAGCGTTTGCTATGCGATGCGCTCAAAAGAAG

CGGCGAGATTGTTCAATCACCTTTGCCTGTTTTGACAATTATTAGTATTTAGTATAATTC

ACCTTTCTTGATAGTGTGTGCTACAAAGTTTAGTGGTTAACTTTGGCATGTATGGTCTGC

TAATTCTGTATCTTAATCTGTCTTAAACACTAATTCTCATTTCTTTCTCAACCAACTAGC

TTTAAATATTCATCATCTTCTGTTATCGAGTCTTCCTTGTTTGCTTTCTATGATTATGTA

AATACAGAATATGTTTACAACTTAATATGTTTAATCAAACTCATGACAAGACTACTTCTT

TCGAGAATCAAAACGGGCGGGCGGGCGCTTTGGCACATTGGCAGGTATCTCTTTGTGTAT

TTCAACATAAAAGGAGGAATCGTTTTGAGACAAATGCGAGTTTTTCTTGTAAGGGGCCTG

GTAGCATATGCTTTCAATAGGACCAACTTCGGTTTAGTGGACCGGAGGTTCACAAATAAT

GGGAGAGGAATCATGGTTTCTTTGTCTACTTAATTGTTTATATATACTTGTGTTTGTAAC

AGTCAGGATAACAAAATCCGTAAATTTGGACAACTCCTTCTACATTCCTTTTCTACTTTC

CAACTAGGTGTGTATCCATCAAGTTACTGACAACTCCTTCTACATTCCTTTTCTACTTTC

CAACTAGGTGTGTATCCATCAAGTTACTGCACACAGCTGTATTTATGTTACAATTACGTT

AGAAGAGACAGTTTGAGCTGGTCTCTTATCATGGTCAGGCGTATATTCGACTTTTCATTA

ACTTTGTGCTTCATTGTGGCTGTGCAATTGGGCTTCTACAGTTGTAATTTCCTGAAGATG

GTCAGGAAAACCAAGTGCTTTGTGCTAAATTCCCCGTCTACTCTTATTGTTACAGCTGCT

TTAAGGATATATAAGTATTTATCGTTTTTACGTGCTGCAGCTTATTGTACATCATGAATA

GGAGATATAGCGCCCTTTTTATTCCTTGAACTCCTCACTGTTTTGAACCATTGTCCTTCG

CGTCCTGTAGTTGGCATAGCCAGACCCTCGAGGGTTTCATTTCCATTTGTTTGTTAGAGA

ATCATGTGGAGGATTTTCCAAAGAAACCCTAAGAGGCGAAGTAATAGGCAAATCACTTGC

TTCGTCTTCCTTTTATTCTTCATCTGATCCATTTTCCACTTTGCAAATAGAAGTCATTTG

CCTTGGTGCTCTTCGTATTAATGTTTCAGTGAGTTCCAGGCTTCGGAGTTGAAAGTCTGA

CTTTCTGAAAAAAGGAACCGTACTTTGTTGACTCTGCATATCAATTAGTTGTTCAAACTA

CTGGCGATTGGAGACTCAGAGATACTGAGAGTAGTCCAACTCAGATATACCGAGAGTAGT

CTATTGCTCGGTTTGCTTCGGAGTTGTTTGATGATCTTTTACCAACCACTGGTGTGTTTA

TCATGTCTAACACTTTTCGACAAAAGGCAGTTCAACATACTTAGAAAATCATGCTGCTGC

ACAGATTAATATCTACCAGTTGAATTAAGCTAAGAAAGCCTGTGTAAGTTGGTCCATCTA

AGAGGGCTGACGATCTGGTTAAAAAAGATGGAGTTCTTTTGTGAGGTTTGCTAGAAATTT

TGTATTTCCACATGTTCTTTGAAGCTTCTTTTGTGTTCACCACAAAAAACCAAGTTAAAT

ATTCTACTTAGGAATTGTGCTCAGTGGCTCAATCTTGAGGAAACATTGTAAAATTCCGTG

ATTGGAACAACACTGTTGCAAGCAGAAATGCACAAGTCCAGAACAAATAAATAGAACACA

AAATGAGAAGAAAACAGATATGACCAAGGATGTATCTGGGTGCAGTTGCTTATGAGATCT

GAAAATACTGCAACATTTGAGAAGTAAATAGCGAGACGTATCAATGTTGTTGGCATAGGG

AACACAAACTGAAGCATAAATCATCAATAATATAGAAAGAACTTGAGCAAAAGCCTAACA

ACAATACTGTCAAGGATCCAGCTGTCCTAAAGCCATAAGCTTTACTGGTTGAGAAGAGCA

GGAATTAGGCTTGATTGAAAGACTATGGTAGAGTTCTCCACAGTAAGTTGATAATCGGAA

AGTGATGCAACAATCTACTCATCACTAACTTTAGTCGAGAAAGGAAGCGGAAAGCAAACA

AGTCTACCAAGGTTTTTGTGTTTGATGATTACATGAAGTCAGAAGCTGAGTCAGGCTAAA

GAGCGAAGGTGTGAAGGAATGAAGGACATGCAAGTTGATGGAGGTTTAAAAGAATATGAT

TTGTGCATGAAGTTTTACAGTAGAGCTAAGTATATTAATTGCTTTCCTTTCGTGTGATAA

AATTAGGCTATCCACATATATTGTGTCTCTGTTCTGCGTATTTACTATTTAGTAGTCTCC

TAAAGTTAGAGATGAATCTGTTACTATCTTGAAGCCCTCTAATGTTCTAAAATTACATTT

CCTGTGTAAAACCTCCATTTTGTGAATAAATAATTGGTTGGGTCCTTGTTGTTTACGCTG

GAAGATATCAGTATCAGCATTGCGTTGAAAGAGTGATCAAACACCTCCATATATGTGAAT

AGCCCAAATCTCAGCATAGGCATACTATCACTAGTTATCTATTACTACTATCTAATGAGG

CGCTGTTCAAGCAGGTCTCCGTCAACGTGCTTACCGACCAGAGGGCAGTTTTGGGATTTC

ACGCTTCACACTACACGAGTCTCATCTCTGTGTATACCAGATGGATTCTAATGATTTCCT

CAAAATCTCCGGCGCTCACCGCTGTTCTTCATTACTGTTCTTCATTACTCTCTCCGTCTT

CTTTCTAATTTTCTTACATCCGTTCTCCGCTTTGCAAAATCTGATTTCCTTTTACTGTTC

TCTGATTGCTTTGGTTTCTTGGGGTTTGGATTATAAATTGTTGTTGCTTCTGGTGAGTTT

TGCCCCTTATCCTACCATTGCTCTTCAGTTAGCTGTTCCTTTATTGTTTGTATTGCTTTC

TGCATGTTCATTTTTTTGATGAAGTGATATCATTGAAGGCATCAAGAAGATGCAAATAGT

ACAGAGGATACAACATTCAAGCTTACAAAAAAAAGAGTTTGCTTGCCATTAGGCATTGCA

TGCTATACTAGCACTAGAGAACTAAGGAAATTCATAAATTGATCTACATTATTTACAGGG

GATAATTCGGTCCAACTGAAAAGAGTGGCAATACATCTAGCTTTTAGACAGTGACTTGGA

GTTGATTCACCATCAAAACAACTTTTGTTTCTTTCTGTCCATAAACACCAGAATATACAT

GCTGGGATCATTAACCAGATTTGCTTGATGGTTTTATCAACTCTTCTTTTGCACCAACTC

TCATAGGCATCTTTTAGCTTGAAAGGCATAACCCAGTTGAGACCAAAAAGGCTATAGAAA

ATGCTCCAAAGATCAAAAGCAGCAGGACAATGAAGTAAAAGGTGGCTTATAGTTTCAGAG

TCCTGCTGGCACATATAGCATTTATTGGCAAATTGATAATTCCTCTTGATAAGGTTATCT

TGTGTCAAAGATGCTCCATTTAGAGCTGTCCAGGTGAAACAAATTACCTTAGGAGGCAAT

TTAGTTCTCCAAATGAGCTTCCAGGGCCAGTCATCAATCATGTTGTTAGAGCATAGGTGC

CGGTACCCTGCTTTTACAGAGTAACACCCTTTAACACCCTTCTGCATGTTCATACTATTC

GAACTAGACCTTTATTCCTTGAAATCAAATAAAAATTCTGGGTTAAATTTAAACTTTGCC

GACGCATAGGAGTCTTTAGGCAGACGAATAGTTCTGTGGTCGATTGCTATGGAGAACCAT

CTTGCTGTGACTTAAAATTTTCAGTTGAATCACTTTTAGAGATGCATAATTTGAGATGAG

ACATGTACAAATCACACCTAAGCCAAGAAACCTTAGACCAACAATGTTCAACAGTTTATC

AGCTAAAGTTGTAGGGTGAAAGTAATTGCTAAGTGGATACAAAACAAACCCAATTGCCCA

AAAAGGCAACCAAAGGAAGCATAACAGTAGAAAAAAGAGCCTGCTTTGACAACACATTAG

CCAACTTCGTATACAAAAGCATAAAACTTATAACCATTGGCAGAAGGTGCTTGGGGAACA

GAGATTTGGTTGGAAAAAATAGAAGAACGAAATCTTGAAGCTCTAGAGCTTAACCCCATC

AATCACCGGACAAGAAAAAGTCAACCTTTTTGCTAACCATTCAACATGAGGGGGTTGCTG

ACTATTTATTTGCTGCAAGATTATTGTAATTGTTTTGGACAACCCCAGATGAAGATTTGA

GATCTACTTGCCATAAAGTATATTGAATATTCAAATAAAACATAATACATACTTTAATAA

CTGTAGATATGTGTGATAATTATGTAAACAATCCAGAGAAAATGACAGGGTA

>XLOC_020772 transcript=TCONS_00034102

TTGTAAGCCTAAGTGTCTTTTTTGTTTTGAATAGAGAATTTGGGATAATTTGGAAAAATT

ATTATAAAGTTAAAAACAAATAAGTAAATAGCTTAATTAGGGGGAAATAAAAGATAATGA

AGCTTCAAGTGTGTCATAAGAAAAGAAGTAAACGTGAGGTTTGAGAAATTTCGGAAGAAC

CAATGGCTTCTTCTTCTCTCATGGCTTCCCAAACCCTTATCCTTCAAAACCCTAATCTTT

TTGCTCCCAATCACAACACCTTCTTTGCTCTCAAATACTCCAAATCCATTTCACTTTCAA

CTCCTAGCCCCAAATTCAAGAAACTCACAACTTCAGCTTCTTCTTCAACTATTCTCCAAA

CCCCATCTCCAAATTTAGCCACAAAATCAAATTCAATTGACACTTTAACCACTAGCTCCA

CCCGTACAATAACAACCCTCATAGCAATTACCTTAACAGCCTCAAAATTATTCTTGCACA

AATTGTCCAACTTGGGCTTTCATGGGGTTGGAAAATCAATTGTATACTCAGCTGGGCCTA

TGTTTTTTGCAGCCCTTAAAAACCAACCCACAACAGGGGGACTTAACACTCCTTTTACTG

TTGTTGCTGCTGGAATGGCGAAATGGCTTGATATATATAGTGGGGTTTTGATGGTTAGGG

TTTTGCTTAGTTGGTTCCCGAATATACCGTGGGATCGACAGCCGTTATCTGCTATTAGAG

ACCTTTGTGATCCTTATTTGAATCTGTTTAGGAATATAATTCCACCAATTTTTGATACTT

TGGATGTTAGTCCACTTTTGGCGTTTGCGGTGTTGGGCACCCTCGGGTCCATTCTTAATA

GCAGCAGGGGAGCATACTGAGGTGATTTAAAGCTCAAGATTATACCCTTCTGTTTGGAGC

TGCTGATCATTCTCGTGGTTGAAAATGGGGATATATAATTCTATTATGGCAATGACTTGT

GGAAGGCTTAGCAAGGACAAACAAGGAACCAAGCAATGACTTCTGTGAAGGCATAGCAAG

GACAAACGAGGAACCAAGAAGTTGACCATGCATCTTCTTTTCTTCTTTCATTTTTGATAC

TTTTGTTAAGCATGATTATAGATCTTTGAGGAATGTCATTTGATGCTTTTGTCTAGCATG

ATGATAGATATAGTATTACAAGTGTTGCCACTTAAATTTGAATTACTTATTGGTAAACAC

AGGAATCCTCCACTTATTCTCCAATGTTCATAATTTTGCTTTATGTCCAACTACGTGTAA

GAGCCTGT

>XLOC_001258 transcript=TCONS_00002038

AAAAACAGTACAACCATGATGTCTCCACGGTATATTGCCGGAAAGGCCAGAGCTTTCTCT

GAGATGGGGTTGTCTAAATACCACCATAAAGTTCTCACAGAAAAATTTCCAGTTATTAGC

ACCCAAGCAACAAGTGGAGAAATGGAGAAGAGCATATATACTATTCTGACTTTAGACCGT

TGGGGTTCGCTCAATCATATGAAGTATAAAGTGACTTCATTAAGGCCAATTCATGGCAAG

TTAGCTTTGAAATTCTTGAATTGGTTCATTAATCAACCTGGTTTAGAGTTTAGTCACATT

ATTCATATGTATGGTATTACAACACATATATTAGTTAGAGCTAGAATGCATGACTGTGTT

AAGTCAATTTTGAGGCATTTATGTGACATGGGTATTGGGCCAAGTTCTGTTTTTGATGCT

CTTATGAATACCTATAGTCTTTGCAGTTCGAACCCTTCTGTTTTTGACATCTTAATTAGG

GTTTATGTAAGAAAAGGGGCAGTTAAAGATGCTCAAAAGATATTCCATTTGATGAGTTCC

CGAGTGTTTAGACCGTCGGTTTATACTTGCAATATGCTCTTAGCTGCATTAGGGAAGCAG

CAGAATGCTGAATCCGTGTGGTTGTTTTTTAGAGAGATGGTTGATAGACGTGTTTGCCCA

AATGTTGGTACGTTCAATATACTTTTACAAGTTCTTTGTGCTAAAGGAAAGGTTGAGAGA

GCAAATTATTTGATAGCAAAGATGGTAGAAAGTGGTTATAGCCCTGACGTGGTTACTTAT

AATACGTTGCTTAATTGGTATTGCAAAAAGGGGAGGTATAAAGCGGCTCTAGAATTAGTT

GATTGCATGAATTCGAAGGGTCTTGAGGCTGATGTTTGTACATACAACATGTTTATAGAT

GACTTATGCAGGAAGAATAGGAGTGCTAAAGGATATTTAGTATTGAGGAAGATGAGGAAG

AGATTGATTGTTCCAAATCATGTGACGTACAATACTCTTATTAACGGGTTTGTGAAAGAA

GGGAAAATTGAGGCTGCAATGAAGATTTTTCATGAGATGTTGAAGTTCAATCTTTCACCA

AATTGTATCACTTTCAATGCTTTGATTGATGGGCATTGTCGAGCAGGCAATCTTAAAGAG

GCTCAAGAAATTCTGAATGAAATGGAAGCAAGAGGTCTACGGCCCAATGAAGTTAGTTAT

GGAGCTCTCTTGAATGGTTTCTGCAAGCATGGGATATTGGATTCTGCAAGAAATATCCTC

AAGAAGATGAAGCTAGATGGATTATCTCCTAATCGTATTGCATATACAATGTTAATAGAA

GGGATATGCAAAACGAGGTCACTTGGAGAAGTTGTACCTTTACTCGAGGAAATGTTTGAG

AGTGGTATATCTCTTGATGTTGTTGCATACTCGGTACTTCTAAATGGATTTTGTAAAGCT

GGAATGCTAAATACAGCAGTGGAGATATTGTGTAAGATGTACAGGTTTGTAGTTTTTCCA

AACGATGTAGTATACTCTACTTTGATCTACAACTTCTGCAAGCAACAAGAGGTCCTAAAA

GCAATGAGAGTCTATGCAATGATGCACAAAACAGGCCATAGTCCTGATACTTTCATATGC

AACACACTAGTCTCTTCTCTTTGTACATGTGGAAGAGTAAGAGAGGCAGAGGATTTCATG

CATCACATGCATAAGATTGGTCTTGTTCCAAATTCTGTTGCTTTTACTTCTGTTATTGAT

TGCTATGCAAGTGTAGGTGAAGGCTTAAAAGCATTATCATGGTTTGAAGAAATGATTAAC

TTGGGTAGTCAGCCTAGCTTTTACACCTATGCAAGCCTACTAAAGGGAATATGCAGAGCA

GGAAACCTTACAGAGGCTCTTAGATTTTTTGATAGACTCCGTGGTATTTTCTGTTCGGCA

GATGTTGTTGTCTACAACACGTTGCTGGCGGAGATATGCAAGTTAGGACACTTCCACATG

GCATTATTCCTGATTGACGAGATGGTTCAGAATAATGTGCTCCCTGATAGTTATACATAC

ACCAATCTCCTAGCTGGCTTGTGTGCGAAGGATAAGTTGGTTGCTGCAATTCTTCTGCTG

GGAAGAGCATTGGACAGAGGAGACTCCTCTTCAAACCGGGTCATGTATACGTGTATCATT

GATGGACTTTTCAAGAGTGGCTTGCCTAAGGTTGCCAATTACTTTTATGATGAGATGACA

ATGAAAGGGCTTAACCCAGATACTGTGGCACTGAATGTGATGATGAACGGTTACTCAAAA

CATGGGCAAATGGATAAGATGCGTAGTTTCTTGGTTACAATGAGACAGGGATGTCCAATG

CCTTCCTTGGCTACATATAATATTCTATTACAAGGCTACTCGAGACAGAAAAACATATCT

GAGTGCTCTAAGTTATATCAGTCACTTAGAGAAAAAGGTCTGGCTCCAGATAAATTAACA

TGCCATTATGTGATTCTTGGACTCTGTGAGTCGAATCTGCTAGATATTGGGGTTAAATTT

ATGATTAAGATGATCTTGAGGGGAACAGTGGCTGATAAATTCACATTCAACATGATTATC

AGTAAATACAGTGAAAGGGGTGAGATGAAGAAGGCCCTTGATCTGCTTACTTTAATGGGT

ACAATCGGTGTTTCTCCTAATGGAGACACTTACAATTTGATATTTAATGGACTGAAACGA

ACATTGGACTTCCAAAGTTCACGCCGCCTCTTGCATAAAATGATCGAAGACGGTTTCATC

CCAACTGACAGACAATATAGTAATTTAATTACTAGTATGTGTAAAGTTGGAGATGTAAAA

GGAGCGTTCAAACTAAAAGATGAAATGGAGTTGTTAGGTGTTAGTTCCCGCAATATAGCT

GAGAGTGCAATTGTAAGGGGGCTTGTGCACCGTGGGAAGATGGAGGAAGCAATCCTGGTA

CTTGATTGTATGTTACGTGTGCATCTAATTCCAACTACTGCAACATTCACAACTGTTATG

CATAGACTTTGCAAAAGTTCTAAGCTCTGTGAGGCCTTAAAATTGAAAGCTACGATGGAG

CTTCATGGTGTAAAGCCAGATGTTATTGTTTACAATGTCCTGATAACAGCCCTTTGTGCT

GATGGCAATATGGATCATGCATTTGATCTCTATGAGGAGCTGAAAGAGAGGGGTCTGTGT

CCCCATGTTACAACTTTCATGGTTCTTGTCAATGCCTTTTGTTCTGAAAACGATCTTGCG

AAGGGCGAAATTATTCTAAGGGATCTGCAAGAGAGAGGATTGACAAGTGAATATTCCAGT

ACCCAACCGTTATGTGAAAGATTGACAGTCGTGAGGGAGAAGCTAAATGCCTTAAGAAAG

AAGAAAAAGAAAAGAAGATAAGTATTGCTGTAGAAAGATGAAAAACTGATCAATTTAACA

CTGGGAACATTTATCTAGCTTGCTGGAGGTCTTCATGGTGGTCTCATGGCTTAAAACAGC

TTCGATAGTTAAAATTAGGTCTATTTGATAAGTCTGCAGCTGGAAATTTGATCACTTCTA

CCTTCTGAAGTCTATTTCTATGCTCGTATCCTTCAAGATATCCATTCACAAGGTGGATAT

AAGGATTATTCTGCAGCTCACGCAGCGGTCTACCATTTTACTGGAACTGATGTCAGGATA

GCTTATGTGCTCAACATTGGAGTGCATTTCATTTCTCCAGCAAGACGGCTAGAGTTGAAG

TCTTGCACAAATCACAGCGAATCTTGTAGCTTCAATATGTAAGTGTATATCTGCCTTGCA

ATTTTTTCACTTCAATTTTGATAAATAGGTGATGGTTAAGAGTAGTATGTCTCTCAATTT

TTCTAGCTTGGCCAAGTGATATAATACTTTTAACCTTGACGTGCAAGGATGCATTATCGC

TAATAGTGATTAGGAATGAGATAGCAGCGAGCGAAGCTAGTGGTAAGGGCAGAAAGAGAT

TTCCCACTATAGGTTTCATCTTGCAAACTCTCAGGTGCATATGATTTCTACTATCCGTTG

TAGTACCTGGATTAGCACCTTTATACTTTTACTCCTGGATTCTTTAAATGTGAGATTTAT

GGAAAGGAAAATACTCTAATGATCTCTGATTTTTAATGATCCCTATGGTGGAGAGATTCT

TTGCACATATGCCTTTAATCTTGCCTTTCTTCGTGAAATATGTCATATGATGTTTCTTGC

CATGATTTAGATTCAATTAATATGGGAAATGGATTTCTTGCCCTACCTTGCATATATTTG

CACAGTTTATACCAAACTACTGTCAGGACCATTAAAATAACTATTAACCTGGGTGCAGAT

ATCTTGGCTGTTCAAAGGGATACGCCGTTTTTGATAAAGTAATGGATTCATTGATAAAAA

TGATGGGGGAAAACCCCATATACATGTGGTATGTAAAAAGTGGTGAACCAGCATGGATAT

ATGGTTCTCCACAAATCGGACCCAATCGTATATACTAACTGGGATCTCGTGTGTGCACAA

AAAAAAAAATTCACTATAAACGGAAGTGTTTCCTAAGCTTTACACTTGTATTTTCCACTA

CCTCAAATGCAGTCGTACGCTCTCTTATTTCAATACTCCAGACTTTTCGAAGCCAATTTG

TAACGTATCTCCCTTTTAAGTCAACTCTTTTCTTGGTAAGCAACATATTTAAAGAATTGT

GAATTTGCCCTGTTCTCAAGTGTTGTCGTTCTGTTTCATCTAGGACTGGTTGAAGTCTCG

GAGTTTTGGTTGCTGAGTTGTCCTGATCCCTAAGGTGATGATTTGCATTCATCAGCCCCA

GCTTGTACTCCATTTGCTGGAAGATAACTCAGTAGCCATGACAGATGCACTTCTTCTCAG

ACAGTTCTATGAGACCTCATCATATCCGTGAACTCACAATAAGGCAGCTCCTTATATATG

GTGTACCACGACATTTTTAACATATATGTAGACCTAATAATGAAGCAGCTGGATGAAGTG

TGGGTCATCAAATTACAGCCAGTCTGGCAATTGTGGAGCCAGGCAGAGAGATGGAATATG

CAATGGTCATTTGATTTAGCCCATTTTCTTTCTGTAGCATAAGATTGTCTTCTTGTTCCC

ATGGCAGATACAAAATGTTAGTACTTGTGC

>XLOC_036784 transcript=TCONS_00060337

TTTCACTTTCAAAAAATGTCTCTTCAGATTCTGCTCTGAGTGTTACAATTATAGATTGTA

TTGGAAGCTCAAGATAGGATTGCGTCGCTCTCTTTCTTTATATCTGTCATTTCTTTGTCG

CCGGCAGCTCTATTCAGTAAGGAGGGGACGAACATGGCTAAAAGATTGGTACTATGTGCT

CCATCAATTGAACCCAAGAATTGTTTGCACAAGGCTAGAGGTCTATGTTGTCTTGTAACA

TAGAGGGAGTTCATATAGTTAGAGCTAATATAGTCAGTGCGCTGTTTAATATTGGTGAAA

TCAGTGGCAACCTTAACCACTTGTCAATTTTCTTTATGTTGTAAGATGTTATTGACTCTC

AATTGATCGAAAACAACTCATATGAGTAAAAATATCAAGAAAAGATTTCAACTTAATGCA

TGTCTTTTTAATCTAATAAAAATCTCAATTGGACAATACAATATCAATTTTGCATAATAG

TTATACTTGATGAGAGAAAGCTGAAAAAGAATGTCCACAATAAAAAGCCAACAAGCAAGC

CATCTGCCAAAAAAATAACAAGTTTACTCAAG

>XLOC_003138 transcript=TCONS_00005105

ATGTCATTGTTATGTGCCTTGGAAAGGGAATCCCATGCAAGATCAGGCCTCTCCAATGCA

TTCTCCATCTCCACAAAGGCCTTCTCAAGGTCTAAGCTTCCAGAATAAAATTCATAAAAG

AGACTATAGCTAGTCTCACAAGCAATCCAATCCTTTTACATTACGACCTTATGCTCGAAG

CCTTGAAGCTAGGTAGAAATATCATCTCAGGAAACTTGCAGCGCTACCTTGTCTGATTTC

CTAGCATAGAGGGTTGCTTTTAGCTCTTGTACGAGGGCCTTTGAAGCCTCATTCTCATAA

GTATCCAGAGTAAAATTCTCCACAAACTTTGCCTCCGCGTACAGGTTTTGCTCATCATAT

AAATAAGCTTGTGATTCTCTTCGTTCAACTTAGACTGCTCTTCAACATGGCCCATTTTAG

CCTTGGCCAACTTTGCCCTCAATGTTATCACTGACTGCTCAGTCTGATTTGCTTCTCCAG

AAGATACACGCATCCTCTTCGTTTTCTTATAATCTTTGGATAACTCATTCCTTTCCTTCA

TAAGCACATCCATTTCCTTTGAAAGGTTGCTAACTCTTACAGTAAGACCGTGTTCCCTTT

CCAACATTCTGATAGATATCACGTTGCTCTAAAATGAGGCAAACTAAAATTGGAGAGTAT

TAGATAAAACATCAAGGAATGGAGATAAAACTTACCCGGAGAATCCAAAAATCAATTTGT

GACATCTGGCCCTCATCCAATTAGAGGCCCATCTGGGCATATTCCATCAGCTCCATTAAG

TCACTGAAGTACCAGGCAGAATTATACGGAACCCCTAGAAAATTGACATCCCGAAGGATA

GAGAAAATAAAGACACACTCAACTCTCTCGAAAGGCTCCCCATCCTCCATAAGCTCCCAA

GAATTAACAGCAACTGGTTCTTCGCAAGGGGGAAGGGGGATGAGCAATGCACTCGGCATG

CTTCTCTGAGAAAAGTGACAAACCAGTAGCCTCAACCCTCGAAAACTTTGACGGCCTTCC

ATTTTTTCATTATTAATCACAATAGTTTCGGCCACACGGCTACCTTCCTACTCATGTAGC

AAAACTCTCTGAAGGCTCATTAACCAGATGGTGATAAGCACTTGCGGACTGTTGCTGGGT

GCTTCCTCTGCCATCCACACTCTTTTTCTCTTATTTGAACAACTTGCC

>XLOC_028564 transcript=TCONS_00046935

TAGGTAGTTAAAAATATTTTTATTCATGTATAAAAAGATGTACATTGCATAGTGATTCAC

CAAGAAATATAGAGAAAATTATTAGTTTTTAATTTCCTTTTCCTACATAGTATCAGAGCA

GTGAAGCATTTCATATCCAATCTTCAGTTTAATGGCTTCCAATACTTCTCAGGGCGCTTG

TGCATCGTCTACTGTCACTACTTTTCTCCAATCTGCTATTGTCATTGATTCTTCTCATCC

ATATTATCTGCATCCATCTGATTCTCCTGGAATGAATCTTGATATTTCCATTTTTGATGG

AAGAGGGTTTTGGAGGTTGAAGCAGTGCTATTGTGATTGCCTTGTCAGCCAAGGACAAGC

TTGGCTTCATTGATGGGTGTGAATCTGACTCAGATCTATTTTAAATCCTGGAGTGGATGC

AACGACGTGGTTCTCTCATGGTTGCTAAACTCCCTTTCAAAAAATATTGTTGAAAGTGTT

CTTTACTGCAGGAGCTCGAAGGATCTCTGGAATGACCTTCCAGAAAGATTTGGTCAGGCA

AATAGAACTCAGTTATTCCAGATTCAAAAGGAACTAAGTGGCTCCATCAAGGGGCCTTCA

ATGAAGAGGCATCTGGATCTTGGTGAAGAAAAATGGGGGCTCTACATCCTGCACTCAAGT

CACTCAACATCAAATATCAAGAAGTTCTCCAGTTTTAGCACTGGTTTTACCTCAAACAGG

AAATTAGGAAATACTAGATAAGTTCCCGATTTTTCTTCCAGTTTTGATTCCTTCCATGTA

AAAGAAAAGTTATGGCACTATATATTAGGTCATTTATTTTTATCTAATATAAAAAAATAT

CAGGTCTGAAGTTTGTACTAAGTTTTCATCTTCTTCTTCAACTTCCCCTCTTGCTAGGCA

GAATAAGCTCTTTTTTACTTTAAGTACGATATCTACCAAGGATTTTTTTTATCTTATTCA

TGTTGATACTTGGGGTCCTTATAAAAGCTTCTACCTATGAAGGTTTCAAATGCTTCCCTA

TTATAGTAGAGGAACTTGGACCTATCTTCTCTCTAATAAATCTAATGCTTTTCCTGTCCT

GATCTCTTTCTTAGCTATGGTACAGAGACAATTCAACAAAAAGATAAAGATTATAATAAC

ATACAATGATTTTAAACTTGGAATAGAAAGACAGAAGCTGAATATTTTCTTTTCCATGGA

ATTCTCCATCAGACTACTTATATTGGAACTCCACAACAAAATGAAATTGTGGAGAGAAAG

TACAAGTATCTTTTGGAGACAGCTAGAGCACTTTGATATAAGTCACATCTTCCTATTATT

TTTTGGGGAGATTGCATCTTAATTGCTACCTGTTTACTTAATAGATTCCTTTTAAGATAA

TTAAAAACAACACACCTTATGAAGTC

>XLOC_016266 transcript=TCONS_00026651

AAAACATTAGCCGCTACTGATACTATTTTACCAGCAAATAAAAGAAATGGCCATTATATC

TCTAATAAAAAGTTACTTTTATTAACTCCAAAATATCTCTGTCCCTACCCATACTAACTT

GGAAACCTAGGGAAAATATTGAAAGCAGTTGATCTCATCTCTGTTCAACACACCGCCGTT

CACCGGCTCCGTTCGACATCGCCGCCGTTCAACGTCGTCGCCGTTCACCGTATCCGTTCA

ACATCGTTCCGCTCCCGTCGCCATTCAACGTCTCCGCTGCTCACCGTCGCCTCACGCCAT

CTCCGTTCTCCATCGCCGGCCACAATCCCAGGGCATATGCAGCTCGGTGATACCGATTCG

ATGCTGGGTCTAGATCGCTCCGTGAGTCTTACAAGAAAGGCTTGAAAATACAGATTGAAT

CTTTAGGGGACACTGATCCAAGAGTTTCGGAGAGTTGCAGATTCCTGGGACTTAGAGATG

GATGCTTGGTGTGGTGGGAGTGCCTGCTGCTATTCAATTTTTCCTCATGCTATTTTTACC

CGAGTCCCCGCGATGGCTCTACATGAAGAAGAGAAAATCTGAAGCAGTCACTATTCTATC

TAAGATTTACGATCCTTATCGGTTGGAGGAAGAAATTGATCAGCTTGCTACTGCATTAGA

GGAAGAACGGTTTAGAAAGCAAGCTATCAGTTACATTGATGTTTTTAGGAAGAAAGAGAT

CCGACTTGCGTTCTTTGCTGGAGCTGGACTGCAGGTATGTTTAGACTTTGTCATAATATA

ATAATGTGTATCATTATTTTAGTTGAAGTTGGTGACTCTGGAATCTGGAACTGCAAATGG

GATAAGCCAGATTTCATTCAGTACTGAGGAAAAGATCTCTCCCAAGAAACCTACCACTCT

GCCAAAAGTGGCAAACCAGAGAGAATTAAGTGGAACACTAGAAAGTGACGCAGATAGCAA

AGCGAAGAAGCAGCTGTCAGATGCAAAGAGCAAGGAACTCAGTGGAAATGATATCTTTGG

CCCTCCCCAGAAATTGCTCCTAGATCTTCAAGGGAAATGTTCCTCCTGGATCTGCAGAAA

AGTGACCTAGCAGCGCAAAGCTGAAAGAAATGAGTGGCAATGATATTTTCTCCGATGGAA

AAGTTGAATCCAGAGATTACTTTGGTGGCGTGCGCAAACCACCAGGTGGAGAGAGCAGCA

TCGCACTAGTTTAGATAAATGCCATCAAACTGCTTTATTGTTAACTTATCGTGTTCCTAC

AACATCTCAGTTGATGACGCTAGTGATGCTTTTAGTGTTTCATGGGATTTAGAACTGAGG

GTTGTAGATTTTCTTGTGTTAACCATGTTGTACGACTACTTCCAAACTCTTAATTTATAT

CAACTTGGAGGATTGTTGTACTAAAAAAAAATTATGTATTTCGGTAGTGAATGATAGTAT

ATTTTATTAGAGATGATTGTATTTATAAATGAATGAG

>XLOC_002430 transcript=TCONS_00003911

CTCAGGTCTTGCTAGATTGTTGGCTAGCCATGCTGCATCACCTGTTAATTTGACTCAAAT

GCTATACACAACCATGCTGGGTTTCACTTCTAGAGCAGCATTTGGAAAAAAATACAAGAA

CCAAGAAAAGTTTATATCTATATTATGTGAACTTCTTAAAGTGGGAACTGGCTTTAATAT

AGCTGATTTTTATCCTTCCATCAACTGGCTTCAAACATAACTGGTCTTAAGACTAAACTG

GAGAAACTTCACCATGAAGTAGATATGTTATTTGAGGAGATTATAGTTGATCATATAACC

TCAAGCAATAATGCTGCTGACAACAAAAATATAATTATACATGAGGACCTGGTGGATGTC

CTTTTAAGAGTACAAAAGAATGAAAAACATGAAATAACTCGCAACAACATTAAAGCAGTG

ATATTGGACATGTTTGCCGCTGGAACAGATACCTCAGCTACCACTGTTCATTGGACAATG

GCAGAAATGCTCAAGAACCCAAGTACCTTAGAGAAAGCGCAACAAGAAGTGAGGCGAGTT

TTTAAGGATATCGGCTGTGTTGATGAATCACAGTTTGATCAATTGAAATACTTAAGAGCT

GTGATAAAAGAGTCCCTTAGGTTGCATCCACGCACCTGCACCTCTATTACTTCCAAGACT

AAGTAGAGAAAAATGTGAGATTGGTGGTTATGAAATACCGCAAAAAACTCAAGTCTTGGT

GAATGCTTGGACAATTGGAAGAGATCCAAGATATTGGGAGGATGCAGAGTGCTTTAAACC

AGAGAGATTTCTTGATAGTTCAATTGATTTCAATGGAAATAATTTTGAGTACATACCATT

TGATGCTGGAAGAAGGATGTGTCCTGGCATGTCATTTGGTCTAGCAAATGTTGAGCTTCC

ACTGGCTCTTTTCCTTTACCATTTTGATTGGAAACTTCCCAATAATGAAGCATGAAGAAT

TGGATATGACAGAGGTATACTTTGGTGTGACAGTGAGAAGAGAGCTTAATATGTATGTAA

TCCCTATTGTCTCCAGGCCTGTATAAGTGTAACAACAGTGCAAGCATCTCTCTCGCTCTT

TCTAATATACTTAATAATCCTGGCGATTTTGTACTAGTATTTGTTTAAAGTTAATAAAAC

TGATAATTTGCCAAGTTGCATTTTATCCCTACGCTGAAAGTTTTGATTGGTTCATATCTA

ACAAATGTAGTTACCAGGCTAGCTTCTGTTTTCAATTAGTAGGGTTTTTATTCTGCAAAC

TAACTTTTGAAGTTAACTCGTGATCAGAACACGGAAAGCCCATCATCTTAACTAAAGTTA

CTGGAAAGGAAGGCCCTGATGGCATCAGAAACAGGGGTGAAAAATCCAGTTTGTCATCAA

TTTCTATAAAAT

>XLOC_014003 transcript=TCONS_00022942

ACTCACCTTAGAAGAAAGGTACCAAATTAAATTTTGATAGATTGAAAATGACCAAAAACT

TTAAAGGCTAAATTAATTCGAAGAGAATTAATCAAAAGACAATGTCATAGACAGTTGCGC

AAAGCTCCTGTCCCTGAATTATGCTCTGTTCAAGGGACAACTTGCAGTTGAAGCGTTGCC

CAATTGGTCAGATAAAAAGGAATAATTTTTATTCTTCTTTCATAGAAATACGGCTCTCAA

GAGTTCTTTCAACTTCATGGGCCATCTCTTGTCAAAGTATTGCAACTTTACCTCAGAAGA

AGTGATAGCGGCATTTAGATGCTGCCAGGACAGTCATTCTGTAGCTGCTGCAATATACTC

TTTCGGTTTCAAGGGCATTATGTGTACCTATTACCTTTCAAAACAAAAGGTTAGCAGTCG

TGTTATGTCTTTTCATGTGATGAAGTTGATGAAGCAGATTGCGAACATATCCGCTTTGCT

TATGTCAAGGTAAAGTTCACCTTAATATGGTTGGGGCTGTTGCTTTCTTTGTGGCAATCA

GCTCTAAAAGAAAAATGCTTGTTGCACTATTACCTGCGCGATGAATCTTTACTTTAGGAA

CAATGGTCATAATCTTTTAGATTATCATCAAAATTTGTTGGTTCTGAAACCTTTCCATAC

TAGACACTTCTAATCTGGAATTATTTCTTTTCACATTTATGTAATATCATTAGGCTGACC

TATTTGGTACTGATTGTATTCTGTATCCCATTCAGATAATTCACTCTGTCACTTTGAGTT

ACCATATTTGACTGCAATTATATCAATACGCTGAGTTATGCTCTCAGCCCAAGGTTTCCC

AGCCTCAAAAGGATATGCCATCTCCTTGAAGGGGTCTTGTAAGGATGCTTTAGTGTGGGA

CTGTGAAAAGTTGTATACTATTTTTTGTTGGACAAAGTATGTAATTAATATTGACCATTT

CTTGGACTTCTTTAGCTCCCTGATTCTAGATTGACTCGTTGTAAAGGAGCTAAACAAAGT

CTTTTTTACTTTTGTATATTTGCATCTCCTTGATGCCTCTTTATAATGAAATACTTCATC

AAAAAAAATGCCTCTTTTGGTACCAATGAATGAG

>XLOC_035976 transcript=TCONS_00059117

TTAGGTACACATAATTTGAACAATTTTCAATACATAGGGGTATTTTTGGCCATTTTCCGT

TTATAACATAATCCAAGATAGATCTACTATTAACATTTCCCTTTTGAATTTCTTGATCTG

CTCTTCTCATTTGCATTAATGGCAAAGGTAGAATCAATGGAAGACACGTCAAAGAAGGTG

GCAAAGAGGAACCGTAGATCCACTAAATTATTTGAGATACTAGATTATGTTTACCTCTTC

ATCTTCTTCTGTTTCCTCTCCTTCTTTCTTGTCAAAATGGTTGCTCTTTGATCCCATTCA

GGATGATGGTAAGCTCGTATCCAGATCAGGAGAAGCTGAAGTTTGAGAACTTCAATTGGT

TAACTCCATTTCAGAGTTCATCTTCATTTTTTGGCCTGTGAGATTTGGAATGGAGCGAGT

TGCCGTGCTGATGACCTGTCATTTGTCTGTGTGCCACAAATTGCATATAAACGTTCTTCA

GATATGTATATTCAGTAGATTTTGTGTTACTAGACTACTCTTTAACACTGATATGACTGA

AAACTTTTGCAACTTAATTCTCAATTGATAGTATCGCCTTTGCTAGTG

>XLOC_014848 transcript=TCONS_00024286

TCGAGAGGTGTAATGATACACGGTGGTGAATCACACTATTTGGTTTGCAGATCTATTATC

TCTCCTCTTTCCTCTTCCCTTTCTCCTATGCTACAGACTATGCAAACACCAAGATTTGAG

AGACAATGTTAACATTCAAAAATAAGGCGGTGATTATATTGGTAGAGGAGCATGGAAGGA

GCTCTATTCCCACTTTTCTAGTAAAGCTGCAACCTCAACTGTAAGGAGTTATCCTTTCAT

CTTCAGATCATGGCTTCTCCAAAATGTTGAAAATTGTTTTTTTAAATCATTTTGATGGAT

TTATCGTATATGCTATTTAGGACCCTTATGATCACTTTTCATTGCTCATCTGCTGTCAAT

ATCTATGGATTCTATTATTTTGTAATTTGTGTAGCACAATATCCGAAATTCATATATTAC

TCTTTCACACGCACCTGTTTGCCATCCTCCTTCCATCCACTTTAGTTTGATTGCTTGGAG

TGTTGGACTTCATCATTTATCATTGTAGTTTCTATTTTGGTTCTATTGAGATGCAGACTC

TGATATGTGATGTCATAGCTATAAAATCCAG

>XLOC_019874 transcript=TCONS_00032573

AAAATACTCAATTTTACTCCACCCCCTTCTCTCTACCTAAATCCCTCAATGAAATCTCAC

CCCATCACTTAATTTTGCAACGATTTCTTCAGGTAGGAGTTCAACCTCTCTCGGCCTTAT

CGGTGTTAGCGAGGATTTCTTCTTTGGGAAAATCCGGTGTTAAGCTATTGGGTATTTGTT

TTTGTGGACTGTGGTATCGATTTCTTACAGGAAAGTATGGAGACTGAAATCGTTATCACA

TTATGCAAACTTGAAACTATTTTTCCTCTGGCGTTCTTTGCTGGACCAGTTCAATCTCGA

TTGATGTGCCCTTTTGAGAGAAATTTGAGAACCCTTACGGGATATGTTCGCAACAACACT

CGTCCAGAAGGTTCAATGTTGGTGGAGGAATACACAAGAGAGAATGAAAGTGAAGACGTG

TTACTTGATGAAAATAATGGAGAGGAAACTGATACATCCTCTTGAAAACTAAGAAGCAAA

GGACTTTAATTGCAGATTTTTTGAAGACCACAATAGAAATATAGCCTTACAATAGATGCA

TTATTTTGATAGCTTTTGGTGCATACAAACTGATCTGCAAAACTTGTAGTTTGCCTGAAG

CATTTTCCGGAAGCATTGGCCAAGAGCAGTTCGTTATTTTCCTTATCCGAATGCAACAGG

AACAATGTTCTTATGACGATTGTAGATGGCATCGAACAGGGAGCTTGAAGGCACATTGTG

TTTTGCTTTTTTGGAGATTTATATAATGTGTACAACCTTGAACAGCTTAAAGGCAAAGTG

TTTATATTTTTTGGTCCTTTATAAGCATACAATGGCGATGAACAATTACATATTGTGTAG

TATTTAAGTAGATCTATTGATAATGAAAGCA

>XLOC_024027 transcript=TCONS_00039487

CAAAAAGGGAGCTGAGACCTTGAGCAAGCTTCTCACAAACTTGTAACGGTTCGCCAAGAA

ACCAAGAAGCATATTCCTCTTCTTCTCACTCTTTTTTCACTCTTTCCCTTTTACAAATCA

AAATTATTATTCTTCAGAGAATCATTTGCTGCTGCTACTGCTGCACAGGGACAAATGGAA

GAAGTGGATCATGACTTGCACGGATGTCCTTCTGGGTTCTTTGGCAGTTGAAGGGGTTTG

AGACAGGGTGATCCGTTATCTCATGTGTTGTTTATCTTAGTGATGGAAGCATTGAGCAAA

ATGATGGACATAGTTATTGCAGGTGGTCCTTTGAAGGGTTTCTCTGCCTCAATCAGGGGA

CTTGGTAGTTTAGGAGAAGTTTCCTGTATTGAGAGATGTGTGAGTTTCAGAACTTGGTTG

AATTACTTTAGATGTATAATTCGGTTGCATGACACTTCTAACGTATGGAGATGGAAAGGG

AGCACAGTTGGAATCTTCTTTTGCCAGAATCTGTTGAAGAG

>XLOC_001099 transcript=TCONS_00001770

AGAATGGCATTGTAAATCATAGTAGGTAACACCTAACGCTAGACGATGACAAGCCTGTTG

ATTGTAGTGGTAGAATAACCAAATCAAGAGATAATGTTGGGCGAGACAAGGTATGCCTTA

ACCCTCATTCCAAAGGCTTCTTACTGGTCCAACAAATAATTAATAGATTCCTCTTTGACA

AGGAGGAGTTTCCTCTTTGGTAGCATTAGAAATATATAGTTGCCTTTGCTTTCGCCCTTT

ACAAAAAGGTACATCATCTACCTTTTCTCATCACGAAGGTTTGTCCCAGGATCTTGCCAA

ATAGTACTTAGTTTCATGTCAGGACAGGTATGCATGGTGATGCGGGTTAATCTGGATTGC

CCTTGTTGTTGTAGGAAAATGAGAAGAATCATTCTAAGAATGAAAGAAATAGAGATGCAC

CTGATAGAGAAGCAACAAAACAGAGTTAGCATCGTTGGAAGGTTTGATCCAGCAGATATA

GCCATAAGAATAAGGAAAAAAATGAACCGAAGAGTTGAGATTTTGGATATTCAAGTACCG

GGGAACGGTGATGGACAGGCTGAGGAGATACCTCATGCCCCTGATGGCCCTGGCCAAACC

ATTATGCAACAAGCCTAAATTTGAAGCTATTTCCGTTTCATGGAAGTTAAATGACCTGAG

ACACAGAATGGCATGCTCATAATATATTTGGCAGTTCTAATGATCAGCAGGTGAGCTCTG

CAGCTTGTACATATTTCCGCAGAGGAAGCTCATGCTATGTTGAATTATTACAAATTAAGT

TTACCTGCATGTTTTAGTCTAAAAAGAAAGAAAACAATTTGTTGGTTTATGCTTGTTTGG

ATGAATTAAGGCCTTCTTTGTATATAATACCAAATTAACTTATGCTGATGTACATAATTC

AAGCTGTCTTATGTTAGTCGAATTACAAGAGAAGAAAGTACATGCATCTTTGATCCAACA

TAATCTAAAGCAATCGACTCGTGGGAATTATAGTAATTTGGATAGAGGGTTACATGCAAT

CACTCAAAAGTCAAAAGAGCGAAAATTTCAACTCCAACCACTGAAGTGTACAAAATTTTT

GCTTACCTTAATATTGC

>XLOC_023579 transcript=TCONS_00038749

ATTTATCTGTAAAGAAAATTTGCTTTCCCACTTCCATTTTTCTCTTCTATTTCAATAAAA

TACTCTCCCGTCTCACCTCTGATTTCGCCGCCCTTAAAACCACTCATCTCTCACGTCTCT

TTCATTCTTTCTTTGCTTGGAGTTTTCAATTTTTTATGTTCCTCGAGATTCTTCAAAATC

AAATTATTTTGTATTTGTGATCAAGGAAGAACCTCTTTGAAGCTGTAAAGACAATGATTA

AGTGAAATTCTTGAAGGCATCAAAGTTCTTTCAAAATCATCTGAGTTGACAAAAGGTGAA

GATAGCTGTCGGCTGGGTTGGTCCGGCTGAGAAAGGCATAGGAATTTGGCATTGCGTGGG

ATGAGTGTTGCATGTCTACTTGCTTCCACTAGAAACTGTAGCTGATATGAAAATACTTTA

TTGCTATATGCCAGAATTGTGAGAACCAAAGAGTAAATGTGCAGGTGATATTTTGAACAT

GATGGCCAGATTGTCAACTGGTGAGCACTCACCTCTTTGCTGAGGAAATGCTAATGTGTG

GAATTGCAGTATAAGAAAGTCACAGTCGTGTTATGTGAACATTTTGGTCAGGAAGAACTG

TTTGTATTGATTATATTGAACAAAGGTGTATTCCAATAGACAGTCTTTCCGATGATTTTA

GTGTTGTATGAACTACTATATTTAGTGTCTGGAATATGTGCCATCAGTATTTCCAAATAG

CTTCCCCT

>XLOC_005903 transcript=TCONS_00009619

ATAATCCCATGTGGTTACTGGTTAATGTTCTCTTGTATAGTTCGAAAAGTAGTATCAAAT

ATTTTGAAATCAAAGTAACAGCTAGAAATTTTGGATTTTGAAATCCCATGTGGTTACTTG

GTTAATGTTGTTTGTATACAACATGTTGTTCTCTTGTATAGTTCGCAAACAATAATCAAG

TTTTTACGCGAAAAGACAGATTTGGTGATGAGTGGAAAAGTTATTATAAGCATGGATGTC

AAGTTCAATGAAACAGAATCTTGGGCTTGGAATGATAGCATTACTTCTGCACCAGTAAAG

TCTCTAGTCCCTGAAAGTGAATCCGAAACACCTGTCACTGAAGCTTTAGCACCAGCACCA

CCTGAAGAACTTGAAGAGACTTCAGCAGAGCCAATTCAACTAAGAAGATCACATACACTT

TGGCGAAGCAGCTGAAAAAGAAGAGTGGCAGCAAGCAATGGTGGAAGAGATGAATGCTAT

CCAAGAGAATGAAACCTGGGAATTGGTTGACTTGCCGGAGGGAAAGAATGTTATTGGCCT

GAAATGGGTGTATCGCACAAAGTACCAAGCTGATGGAACGGTTCAGAAGCACAAAGCTCG

GTTAGTTGCAAAAGGGTACGCTCAACAACAAGGAGTTGACTTTGATGAAACATTCTCTCC

TGTAGCACGCTTTGAAACGGTAAGAACTCTTAGCTCTTGCTGCTCAACTTTCTTGGCCTG

TGTACCAGTTTGATGTCAAGTCGGCCTTCTTGAATGGAGAGTTGGAAGAAGAAGTTTATG

TTGCTCAACCTGAAGGCTTTGTTGTGACTGAGAAGAAAGTAAAGTGTACAGGTTAAAAAA

GGCTTTATATGGCCTAAAGCAAGCCCCGCGAGCATGGTACAGTAAGATTGATTCTTATTT

TCTGGAAAATGGATTTGAAAGAAGCAAAAATGAGCCAACTCTACGTAAAGAAGCAAGGTA

AAACTGATTTCTTGGTGGTGTGCCTTTATGTAGATGATATGATTTATATGGGCCCTTGTG

AATCTCTTATCGCTGAATGAGAAGCTCATATCTAATGATGGTACAGGTTTGGCGAATGCC

CGATGCTTTAGAAGCATAGTTGGTGGTCTGAATTATTTATCTCACACTAGGCCTGACATA

CATTTTCTGTTAGTGTGGTTTCCAGATTCATGCATAACCCAACAAAGCATCATCTTGGGG

CAGTAAAGCGGATTATACGATATGTTGCTGGAACTGTTGATTATGGAATTTGGTACTCTA

AAGTGTCATATTTCAGATTGTTTGGTTTCACAGATAGTGATTGGGCAGGTTGTCTAGATG

ATAGAAAGAGTACATCTGGATATATTTTCTCTCTTGGCTCAGGTGCTATGTCCTGGAGTT

CGAAGAAGCAAGAAATTGTGGCCTTGTCGTCATCCGAGGCAGAATATGTTGCTGCCACTG

CATCAGCTTGTCAAGCTTGCTGCAACTGAAATTTTCTGTGACAACAAAGCATCTATTGCA

ATGACAAAGAATCCGGCATTTATTGCAAGGGGGAGTGTTGATTAATGCTTCAAAGTGACA

GCTTAGTTCCTGATTTCTAGCATCAGTAGATTTAGTGTTTGTTTAGTAACTTAGCATTAT

GTGTTAGTTGGCTATTTTGTAGTGGATCATGGTAGAATTAGTGGGAACATGTTTATCCAC

TTTTCTCTTAGTTATTTGCTTATTTTTACCTAAGGTTAGTTAGTTGTAAGCCTATTTAAA

AGGCCCCTGCTGCAGCTTTTCATAATGCAATTTAG

>XLOC_035273 transcript=TCONS_00058018

ATAAGCTTGATTTAACAGTTAAAAATAACCAGACAAATTATCAGGCCAATTGCTGGTAAA

GTCATAGAGAAATGAGCCGAAGAGAGTGCTGTCTGGATGGTTGCCTGAAGAAGATCAGCG

CGGTGCTTCAGACGCCCCTTGTTAAGTGATGTGGCTCCTGGAGAGGCATACTACTGCCTA

AACGGTCACTGGAAATAAGAATGGTGCCACTAGACTGGTAATCAGAAGGAGATGAGTAAT

AAAATTCCCAAAGAAACATTTCCTTAGGTCTCTAAAAATATCATAGAGGCCTGATACCAC

TGAAAGGAGGGAGAAAGCCGCCAGGGGTTTGGGTTTTGTCTACTGGTTAAAAGAGCAGCT

TGTGATGTGTGGGTTCGGCACATGTCATTGGTTCGGACCTTTGCCCTAGTTACCTGGTAT

TTAGATGGAGAAGGGTAGAGGGGTTGGCCCATTATCGACCAAGTTTCAAACCGTGCACTA

CGGGCCTTCGGGTTATGAGAAACTCTTCTTGAAACTAACATCATGAGAGCTCCTTGTACA

AGAGATTTGAGCTATATGAGAAAGAAACTTTTCATGCTTCTAAGGGTGGTGTTATGGGCC

TGCTCGCACCTTGACTATTTCACTGAACAACTTGCTGCAGCCAGCAGTACAGGTTTGGGG

TAATCAGCTCACCACGGTAGAGGAGATGAGGATGAGCTTATACAGAGTGTTATAGCTTAG

CTTTGAAGCTGAGATCTTAGTTGAACGGGCTTGGGAGTGATAGCACGCAAAACCTTTTAC

CAAGTGGTGGCTACTGCTTTTAAGGTGCTAAAAAGGATGTAGAAAATGCAAGTGTCTATG

TAGTGGTAGTGAATGATCAGCGGAGATTGCTTCAAGAATTTGCTTTCGAATTGAATGCAC

TGAGAAGCTGTTGTTGCTGCAATGAAGGCATTGGAGGTTGCATGACATTATGATTGGAAA

CGAGTTCATGTACAATCAGATGTTAAGGTGCTGGTTTAGATGTTCAAGGGTCATATAGGA

CTAACATGGGTGATGCAATGTATGCGAAGAGATTTGGCATCTAAAGGATTAGTTGAATGT

TGTACAGTTTTCTCGTCTTAAGAGGAATATTAATGGGTGCAGTCACTGTATTGCGAGTTT

CTCGATAAATTTGTTCAATGGGGTTTCTTGAGAATTTTATTTTTCAAGTTGGCTTTGTAT

TTGAAATTTGAAATTCTGTCGTCTTACTGGTCACATGAATAAGTCAATATTCCTATGGAA

AAATAAGTAACTTCAGGCAGGATACGCACTTCTATAACGGATGCACACCGGGAGCACATT

AATTGTGCTGGTATCTTATGTAACCAATTGTAGAGGAAAGCAATCTAGTTTGTAAATTTA

CTTGTGTTTGAATGTTTTCTGTGAAGTTGTAATGATTTGGTTAAATTGACAGATTCAATT

AAGATTTTTAAGTTACCC

>XLOC_030120 transcript=TCONS_00049581

TATTTCTATTTGTTGTTGTCTTATATTGTTTATTATTTTATACATATTATTTCTATATTA

ACTGGTTATTGTTAATATTAAGAAATAATTCTATGTGTTTAGTTGCATGTATAAATATTA

TTTGAATATTAACTTGGTATGTTTCAATATTAAGAGATATTATTTGTCTGTTTATATTTA

TGTTTTGCATTTGTCAGACATGGGAATATTTCACTCCAAAGGATAAGCAGGGAAATAGGC

CAATTAATGTTCATTGCAATAAGGAGATTAGTTCAGAGCTTAAGAAAAGGTTAAGTGGTG

GTTTATATGAGAGGTTTAAAGCTACATGTTTTGGTCATTTCCTGGAGTTGCCTGCATTTC

ATGTTCAGAATCAAGTTCTTGTTGCTCTTTTATTTAGAGAAATTGTACGCCCATCTGATG

ATGCGTTGTTTTTTATAATTAATAATCAAAAATTAAAATTTGGGTTGAGGGAATTTGCTT

TGGTTACGGGGTTGAAATGTGTAGGTGATTTCATGAAAGATTATAGCTCTAAGGAGGATA

ATTGTCTTGTTGTTAAATATTTTAGTGGGGTTGATAAGGTTAGCAAATCTGCATTGGAAG

ATTGTTTTAAAAATAAAATGTGGGACAATGATGATGATGCTATAAAAATTGCAGCGTTAT

ACCTTATTAATACTTTTCTTTATTCTACCAAGAATAGTGAAACTAACCCACATTATATAG

ATTCTGAGCATTTTGATATTATAGAGTCGGGTGAATGGGAGTCATATCCGTGGGGTTATA

AGATATTTCTTGCAACTATTGAATCGTTGCACAACAAGGTTTCTGTGTGGAAATCTTCTT

TCAGATTTGCAGGGATGCCATATGCTTTGCTTGTATGGTTTTTTGAATGCTGCCCCACTG

TTCATGGCAAGCTTGCTAATCGTGTTGGTAACAAGGTCCCGCGTATTCTTAATTGGGAGG

TTACGAGGCATCTTTCATTCAATGAAGTACAGAGGGGTTTTTTTTCACTTACTGGTGACA

AGGTATTTTTTGTGTTTTTATTCATTGATATTAAATAAATATTAAATTATAAATTTATTT

GTTGATATTAATTGGATATGTGTATTCTTGGTTGGTGAATTTAAGATGGTTGTGCATTGT

ATTAATGCTACTGATATTGAGATTCAGTCAATGAATTTGCCAGTGGAAAGTGAAACAAAT

ATTGATGTAGATATTGGTTGTCAAGTTGAAACAGAATATGTAGGATCTGATGCATCGGTT

CAGGTTGATGGGGATAGTGATATTCTTGGGAGGAGAGTAGGTTTTATGGAAAAAATGGAT

GTTTTTTTTACAGGAACAGGCTGTTATTAAGAAGGAAATTCTTGCTAACACAGTTGCGGT

TGCGGGGTTGAAAGAGTTTATGGTGTCCACAGCTGTTCGTTTATTCAATGAGTTGGAACA

GATTTCAAATAAGTTAGATGTTAAGGGAGTGGAGCAAGTTGTTAACATGATTTTAATATT

TATGCAATATTATTAAATATATTATTTGTCATATTTATTTGTGTATGTTATGTCAATGTA

GGATGTAGCAATGGATGGTGCATTTAGTTTTGGTGGATTTGGATCTGATAATGAAGCTGG

TTGTGATGTAGTTAATGTTGGTGGCGCTGATACTACGCCTCTGTGTACACCTCGGGACTT

AAATGTTCCTGGTTGTTCTGTTGTTAATTCTGAAGCATTTTTGAATCAGACGCCTAGTAT

GCAAGGTGCAGATGTTATTGAGAAGGACGATGTTAGTGATCCAAATGTCATTGATGTATC

CAAACACTGGAAGATATGGTGGATTGTGATAGTGTGTTGTTTTTTATTAATGCCTAGAAG

ACTGGACTTTGGTGAAATGGGGAAGCAAGATGTAAATGGTGATGGTATTGAATCATTGTC

TACCCAGGAACTAAATATCATTGATGAGAGTGTGGAGAAAAGACTTCAGGAA

>XLOC_025987 transcript=TCONS_00042738

AATTTATGTTGTTTATTGCGTCTAAGTCATTACTTTGGACAAGTTTATACGCACAAATCT

CCCCCCTAAAAATAGATTATACATGAATATACATAGATAAATGTGTAGCAATGAAAGAAA

TATAAAAATATACACAAAAAAGTAAAAATTCATATAAACATCCTTAATATACAGATTTAT

TCACTTTTATATATACACTTATACAATTTTATACACGTATATAAATTTGTATAAAATTGT

ATAAACTTGTATAAAGTTGCGGCATATTACTATCTCGGGAAAATGGTGACATGTCGGGTA

AGATTCAGAAATATGAGCATTTTTTTTTTGTAATTTTCGTCTTTTGCTTGTCTTCTTTTT

GTAATTATCCCAAAGAAAACAGTTTAGATATTATGTCTACGAATCATTAGATTGTAAGGT

GGTAGTTGCTGGTGGTACTTGACAGTGATGTTTGGTGCCGATGATTGTTGATAGTGGTAA

TTTGTGGTGGTTGTGTGATAATGGTGGTTTGATGATGGAGGTGGTTAGTGGTAATGACTA

ATGCCTAGTAATGTTGGCGGTGGTAGGTAATGGGGAAGGGGGTGGTAGCTAGTTGATGAT

AGAGGCAGTTGTGTTAGTAGAGGTTGTTGGTATTAGTGGTTGGTGGTGGTGGTGGTTGGT

AGAGAAGATGGTATTTGTGATGTGGAGGTTGTTATTGTTGTAGAGATTGTCAGTGATTTT

CAGTGGTAGTATTGGTTGTGATGGTAGAGGTAGTTGATAGTTGGTGGCAGTTGGTAGTGG

TGGTGGGGGGTGGTAGTTGGTGGTTGGTAGTGGTGGTTGATGGTTGTGAGTGGT

>XLOC_010751 transcript=TCONS_00017545

CAGGAAGCAAGGTATATGCCTATCAGGAATTGGGATGCTTATTTTAGTCTCTACTGTATG

AGGTTGTACAATGACTATAGGACAATTGCTCACCATTACAAAGTGGAGTTCAATGGGATA

ATGATAATAAGGTGATAGAAGGTGATGACAAACATGTTGTATATCTTGAAATGAATAAAA

TGCACATGTAGGGTGTGGGACATGTCTGGTATATCCTATCCCCATGCTATAAAGGCTATG

CTACATAAGAAGATGGACCCATTGACACAAATTCATTGGTGGCTTGGTAGGGAGGCATAT

CCACAGACGCACAAACACAAAATGCAGTCTGTTAAAGGGAAAAATTCTAGAAAGTTGATC

CTTCTTATGCTATTGGATCTTCTGATGTGTCAAGGGTGTGGGCAGGCCCAAGGTGAAGAG

AAACAGGGAACCAAACATGGCAAAATTGTAAAAAGAAGCTTGGGTTGCATCTAGAAAGGG

TACTACCATTTTTGTAGCAAATATGGTGAACCAAATCACAACGTTAGGGGTTGTTAGAAA

GTAAAATTTAGAGCTTTCAATTATTTACGGATGTTATTCTTTTTGACTGGCTTGAATTAT

ATACATGTCATGTGTAGTCAGTTGGACAAGCTTCAAGCTCAGGGCAAAATGTGAATCAAC

AGTATGTGAATCAACAAGCTTCGATCTCAAGGCAAAATGTGACACATTCCTCAGCTGCCG

AATCAAAAGTGGAAAGTCAGAGTATGGATGAAGAACAAGCTGCTACATAGTATTCTAGTT

ATGGTTGTAATGTGGGTTCTTCCTCAAACCCAAATTAAGACCCATGGTTATATCTGAATT

ACAAACTAGACTCGATGAAAGGAAAGGAGTGGAGGAAACCAACTGGAAGAAGAAGGATCT

CATTTACTGGAGATGCAAAGACATGAGTGTCAATACCAAGCAATTTTCCATTTCAACCAG

ATACTATCACTTGGAATGGTGGGGCAATTGTTACATCTAATCAAATGGTCCTTGAAGCTT

TAAGGACGAGACAAAATTTGAGGTCTGGGATGGGACCAGAACTTCAAGATGAAGACATGT

CTATGCCTTGAAAGACTGCAAACAACACCAAAGAATGAAGATGCCTCGAAGAGAAAGCCA

CCCAAGAGCACCAAAAAATAAG

>XLOC_027009 transcript=TCONS_00044454

ATGATCAGAAAATGCGATTTATTGTTTGTATTCAGCAACTTTGATCAACTCTTTCATTGA

GTATTGGGCCTATTAGGAAATTGTGATCAACTATAGTCAATTCTCTGCACAAGTAGCTAG

AGGTGTGTGTTCCACAATTTGTAGAATAATGGGTATGGCACTATCCAGGTTTGTAAAAAT

GCTATTTGCCAAGAAAGAAATGAGAATATTGATGGTAGGACTCGATGCAGCTGGTAAAAC

AACCATCTTGTACAAGTTAAAACTTGGGGAAATTGTCACTACTATTCCTACAATCGGATT

TAACGTGGAGACTGTAGAATACAAGAACTCAAGCTTCACAGTATGGGATGTGGGTGGACA

AGACAAGATTCGGCCACTATGGAGGCACTATTTTCAGAACACTCAAGGTCTAATATTTGT

GGTAGATAGTAATGATCGAGATAGAATTACAGAAGCCAGGGATGAGCTTCATCGAATGCT

TAACGAGGGAGAATTGCGAGGCGCCACAATACTTGTTTTCGCCAATAAACAAGACCTTCC

AAATGCTATGAGTGTTGCAGAAATTACTGACAAACTTGGCCTGCATTCACTCCGGCAAAG

ACGCTGGTACATCCAGAGTACTTGTGCAACATCTGGCCAGGGACTCTATGAAGGTCTTGA

TTGGTTGTCAAATAATATCACTGCCAAGGCATGAATTCATTTCTACATCCAGCACAAGAC

TATAAATCCAAACGCGTTGTTTGAATTTAATACTACATGCTATAGTTTCTATGGCTATAC

ATTGTGATGCAAGAGTTACGTTGAATAATTATAAACCTATATATAGTGTTAAATGGCGAT

GTCCATTTTCATTGCTCAACTGTTTCGTACCTTTTGTATATGCATGATAGGATTTATCTT

AAGAATGTCCATATATTATTGATAGCACCCTTTTATCACAAAAGCTTAT

>XLOC_015273 transcript=TCONS_00025004

GTTCGTTTGGAAAGCAAATGCAGGTGGACAACCTTGCAAGACTGTAGCAGATTGCCATAT

TAACTGTGGCAGCAGTGAAGCCCATCCAATTTGTGTCAACGACACCTGCTTGTGCCAACA

CTGCGAGAATTTCTTCCCCCCTAATCCACCCTCTACTACTGATGAAGAATCTGTTGTAAG

CCAAGAATGTGCAACTCGTCCTTTTAATTCCAAATGTAAGAAGTAGTGTAAGAATAGTGT

ACCCTCG

>XLOC_031357 transcript=TCONS_00051592

AAACAAAACCCTAAAAACATTTCCCCCTACCCCTTCCCCTAAGCAACGCCGCCCCTCTCC

CCTTCCTCTCTCCCGCTCGCCCAAAAAGCCGACCCAATACATCTCCATCCAAAAATACGT

CCTATATCCGACAACCCACACCATTTCCGCCATGAAAACCGTCCGTCACACCCCCATATC

TCTATCCTATCTCATCTCTTTCATGATTCAACAAGGAAAATAAAAGAATCACGCAAATCA

CTTCAAATTCGAAACAATCTAGACAAAATTGGAGGAGATACAATACATATAAGGACATGG

AATCGAAATTTCGGAATCAAAACCAAACAAAAAGGAACAAACTGAAATGGGCCAATTTGG

ACCCGACCCAGTTCAACCACTACGACCCGAGCCGGATAAAAAACGGGTTAACAAAGGATG

TTGTGGTTTAAAGAAAACCATACTTTGCACTAACCTCTCTTCTGCAGGAGGTTGGATTTC

TATGAAATAGTTGATAATTCAAACTGAAGGAGCCAAGGCCTAATCACTGACATCTCTCTT

TATTTTCTTAATGAAGCTAATGAATTATGTTTGAACGTTTGTTTAAATTCATTAGTTTGT

ACAAAGTACCGTTAGGTGCTAATACAATTTGAGACAGAAACAAGCGATGTAATGTTTCCA

AAAAGGCAATACAACTAAGGCTAAACAGGGGCCTCG

>XLOC_019152 transcript=TCONS_00031423

GAGAAAAGAAGTCATTTTCACTTCTCATCTTTCATCTTCATACATCTCTCTTCCTGGTCG

TAAGATATAAAGTACACAGGAAAGCACAAGAGATAGACCAACAACTGGAACAAAGAGTAT

GTGAAGAGACCTCTTCGAGCTGCACAAATAATAGTCCTTCATGTCATGAGCAGCGCCATA

CGTTCATCATTTTAAAATGGATAGGCAATGATAGTTGCTATCCTTAACTTGTGAAAAAGT

AATTTTATCTAATGGCATAGTCCATACGAATATCTTGTAAAGTTTGTATTTATAGACGAA

GAAGTACATTTATAAGTTATGGAGAACTCAAAAGTCTCCTTCCAGAAAGGTCCACAACAC

GACTGATGGCTTTCTTCATATATCAAAGGCTAATACATACAAGGAAGAACTGTTTAGTAT

TCATCTGCATTTATTGTTAGAAGCTGGAGCATGCAAATCTAATGCCACCAAATTTAAAAG

GTCAGGTACATTGCTCTAAGTTTATTGCATTTTTGTGATGCACTGTATAATGAAGAGCTT

TTCGTAGCCATCAATTGTGACTCACGTTTCTATAATAATAAGGGGAGCTTTTAGTTGTAA

ATTGTGATAGTAATTTTGACTTTCAGTGACTCATCATAAAATTAACAATGTATTTGTACA

ATAATGATAATATAAAAAAGGATACTATATAATGGAGGA

>XLOC_029300 transcript=TCONS_00048188

TTAAGGTTTGGTCTAGTTGGAAAGGCTAATATGGTTATTTTCCTTGCAGGAAAAGCCTGC

CCTCTCCCATTCCATGAGGCCAACCAAGTCGATCGAATCGATAGAGAGAGAGGGAACCCC

GAAAACTGTTCTGATAGTATCATAAAATTCTAAAACATGTAAATCCACCTTGGTGCAATT

CAAGAATTACTTGTCTACAGACTCTGCACCTTCTATCAAACTTGGGTTTAGTATAGCAGT

GCAGCTATGACATATAAGATGGCATATTGAAGTCTTGCATGTAGAATTCCTCTATATGAA

ACCAGCACGGCAATACTTGATATTAAGTATTCCTTCTATTACTGAGAGTAAGAGCTGCAA

CATTCTTTGTGACCGCGGCAGCTTCATTAGCTGCAAGGCTGGGATCGATATATATCCATG

GCAGCTTTTCAGGTCTGCTTGCGTTTGTTTGGCAACCTCTTTACTTTCTGATGGACTTTC

TGTGGCTGGGCAAGCAAGGATACTGGATAGCATTCTGTTTCTTTCCCCTCTCTAATTAAC

TTGTGATGAACCTTCTAGCAGCACTTGTTGTACAATGACAGATGGGAATTGTACATACTT

CACTTGCCAACTTAGAATGAATATTTTGAAAGTGCT

>XLOC_004173 transcript=TCONS_00006812

GTGAAATCGCAAAGGTGACACCAGAGAACAAACTCGACCAAACCAGACCACCAGCCAATT

ACAGAACTCCAATCCACCCTTGCTCTCATCTACTTCTCAGAGTTATACTCCAAAAGAAAA

CCTAAATATAAATCTAAAATTTTCTTAAACTTACTAAGTGGTGTTTTTTCTTTTCCTCTC

TCAAGTTTTCCTTTTTTTTCTAAACAAAATTCAGATCTGAAGTGATGGTTTTTCTCAGAT

TTCTGTGTGTTTTAGAA

>XLOC_013329 transcript=TCONS_00021851

CAAGAAAATACCCTTACTTCATTTATGTGTTTCTTTGTTCTTCTCACCCCTAAAGTCCCC

ATGACCCCAACTATATTCCCTTCATATTTTCATAGGAATCCCCCTATTTATCTCCTCAAT

CCTAACTTTGAAATTGTATAATTTCCTCTTTACACAGATCCATTCTCCATGAGTTCATAT

ATGCACTTTCACGGCCTGAAATTTTACTCTAACGGTCCCGATCTGGACTCACCAAGTCCT

TGTCTACTCTTTCTTGGATAGATCTATGCTTTTCCGGTATAGATACACAGTCTACTCTTT

CTCGGTATAGATCTAATAGTACTTTCTAGTCAGATTTTGCTCTCGATAAGTAGCGGGACT

AAATATTACATGGTCGGTTTCACGTTTGGAGTATCTTAAAGTATACAAAAAACTTCTTGA

TTTTAAAGGGAAACATATCCAATTTGGATGGAGTGATGCGCTCTGTTAGGCTTTCATTAC

TATTCCGTGTATGGACATGTTGAAAAGCTAGCACAAGAGATAAAGAAAGGAGTTACATCA

GTTGAAGGAGTAAAAGCTAAACTCTGGCAGGGAAGGCAGTCAAACCTTATTAGCAAGATC

TTGGAATCAAACATAAAACAAAATATTGTAAAACATGAATCTGAGCTGGCCATGAGGTTG

AACTGTGCAGCTCACGATGGAGATGTGTACATGCTAAGTCGTTTGATTGGCGCAGGAGCA

TATCTCAACCAAGCTGATTATATGATGGAAGATCACCTCAGGTATCTATTTTTTACTTTA

ATTTCTTCTACTATAAAGCTATAAGTGACAATCTCTGTTTACCTTGTTTACTTAATTTTC

TTTAGTTAAAATCACTGTACTTGGACTAAAGACCCGGTGTTTATACTACGCCAATGTGTG

TATGTCATGTTTTAACAAATACACAATTCCCACTTAACCGTGGTTAGTAAATACATAGAT

TTACCTTATTAAATTATTTAACCATGATTTATTGGTATTGGGCGTGGGTAAGTATTTACC

CTTCCTTTATTGAATAAGTAGTATTTCATTAGTTTCCTCCTATATAATACATTTCACATT

AATTGGTAATTAGTTGGGATTTAATTAGCCTACTAATTTAACTTAATTTAATTTCTTTTG

TTAATTAAAAAGGCATAAGTGGCGACTCTAGTTGCCACAAAAATAAACTTCAGAGAAGGT

GTGCTTGTTTTAGTGGCGGCTCAGTTGCCACTATAACCAATCTTTAGTGGCGATTTACCT

AAGGTTTAAACTTTTGGATATATTTTTGTGGCAATGGTGTCTAGTTTTTGTGGCGGTAAT

AGTTGCCACAAATGTAAACATTTCGTGGCGACAAAAGGAAGTCGCCACTCTAAATATTCA

GTATCAGCTTGTAGTGCAGCAACCTTATCGTCACCACTAAAAACTTTTTGTGGCGACTTT

TCAGGCTTTAGTGGCAATTTTAGTCGCCACAATATGTGAGATTTCTTGTAGTGGAAGCAT

GGACATTTCATAGTATTGTGAGTGTACTTCAATTATATTGGGAACTTAGGTGGTTTGTGC

ACTTAATATATACTTCAATTGTATAAGAACTAAGCCTTGTTCTTGGTGATTAGACCTCTG

TATTGAAAATTACTTCATCTAATATGTTGCTAGGAGCTTTTTGCATACGTGTACATATTA

CTGGCGCTGAATCTGATTCTGCAAAATTACAGTAGTTTTCCTTAAAAGCAAAAACCTGTC

TAGCTTTATTAATCTTCTGCTTATGTTTATTCTTTCCCTTAAGGTTGCATAAGCACGTCC

TCTGTATATATGTTACTGTATCAGTTTTCACTCCAGTTTAGCTAGAGTTTGAGAACGGAA

GGAGTTGAACAGTTCAATGTGGAGTTGCTTCTCTTTTCTTACTCCAATTCAGCTTCAAAA

CGTACTTGAATGTCAGCTAACACAGTTTAATTCCTTTTAAATCAATTGCCAATGGAAATT

GACTGAATATTGGAGATATCTTGTGGAGGTAAAATCATTATAGTTGCTTGTAGTTTTTGC

TCTAAGCTCTTTTAATGCTTCCTATACAGTGAAGTTTAATCATATGTTTTGAAGAAAATA

CATTTATAAGATTCCTGATAAATGTCTATATGGTAAATGTTTTGGTAATTGCCTCTCCTA

TGACTTTGGACCATTGCATTTAGATTTGACTGCAATTTTTGCTCTTTTAGTACTTACATG

TAAGTTCCTTGACACTCACTTTTGGTAATGCTATCACTAGTGGGCTTGGGCTGTTACATC

CAACTTGGTTGTACTGATAGGTTTGGATTCAATGATGGCAGTTATCTTCGCTTCCCCGGA

GTCAGGAAGTGTTCTAAGAACCTTTTCTACAATATCTTTCTCGGGGAGGTCCATACCCAA

GGACTTCATTTCATCCGTAATGATGGTTAACCTAGTCATCATGTCTTGAATTGACTCCTT

TTCATCTGTGGTGAAGAGTTCAAATTGCCTCTTGCCTCTTGAACATCTTCTTCTTCGAGT

TCTTGACTTGACTTGTTCCTTCATGAGTAACTTTCAAAACAGCCCAGATCTCTTATGCAG

CCTTATATGAAGAAACTCGATTGAAAACCTCAGCGCCAAGTCCACAGATGACAAGTCTTT

TGGCTTTGTGGTTTTTCTCAATCATCTTGTAATCAGCTTCATTGTACTCAAACTTATCAT

TTGGTTCAGCTCGAGCAGCAGCTCCTTCTCGAACAGTCTTAGTTGGAACCAAATGACCCT

TAGAGATTATCAGCCATAATTGATAATCTTCAGCATATATAAAGTTCTCTATTCTGGTCT

TCCACCAGGCATAACTTTGCCCATTGAATTGAGGGGGTTTGAGGATAGACTATCCTTCAG

TAATTGTTGGTGGTCCACTCATGGTTAGATCCTTCTCAAGGTGTTATCCTATTGATGAGA

AGGCATGCTCTGATATCACTTGTTGCATAAGGTACACCTATTTCGGACACTAGAAGAAAG

GGGTTGAATAGTGTCCTGCGATTTTTCTGAAGAACTTTGTTCGTCCCATCAAGAACCTAG

TTCTTTCCTAGTTTCCTTAATCACTTGAGTAAACACAGATCTAAAACAAAACTAAAGAAC

ACATAGATTTTAAATAGAAAACTCCTTGCTCAAAGGAGAAAAAGTACGACCCTAGCTCTG

GAATTTCTCAAACTCTTTCACTAACACAACGAGCAAGTTTTTAGGATACAAACTCAATAA

CCAAGTGGCTAAACTCTAGACCCTACAACTCTCCATGATATACTCTAGGTTTTACAAGTT

ATTTCTCACCAGAATCACCTATTACAATGCTTCTAAAAATGAGCAAATAGGTAACAGTTC

ATAACAAGTTCAATACAACAACAATATGTAAGAACTAATACTAAAACTGGATCACCACTA

AGTTCTTCAACAGTTCTCTGCTTTTGTCCACTCACTCACAGTCAAAACTGAATTGCCTTT

GATTGCTCTTAACTCTCGTAGTGAATGAATGAATAAGGCTAGCTTCAATTACCTGTTTAT

ATAGGGTTTAGAAAATACTAGAAGTTTACTACTAGGAATCTTCTCAATTATGAGAAGTGA

AGATCTCCTACGATATACTCCTTGTTTAACTCTGCTTCTCGTTCATACTTCATAGAGGCG

AAAAAGATACATTCATCTTTTTTATCAGTCATGCCTTCTCATAAGGACTAGGTCCTCCGC

AAGGTCCTTTGTTTGTCAATATTTAAAACTTAATCATCATGCAACCTATTATTTATAATT

TTTTATACATAAAAATATAGAGCAACTAACATAAAACACCTTAACAATATTTTTTTTCAT

TATGTAACCAACAATTTAAAAGATTCTTATTAATGTCATTTTGAAGAATTTAATATTATA

TTTCTATATTCATTTTCAAAAATACTACTTTCACAATCCCATAAAAATCACCTAAATCAA

TATATATTAGATATTCCTATTCAATAAGTGTTTATACCTAAATCGATTGTCTAGGTTAAA

TAATTAATTAAGAATGATGTTACAAGCAATACAGTAAATACAATGGAAAAGTAAATAAAT

AAATAAATAATTAAAATAAACAATGAGAAAAAAAAGTAAAGTAGTATTATTCCTTGAGAA

TGGACAGATTATGTGGTGAATCGCAATGATTGACAATACAATGTAAGGAGGGGGTATTTA

TAGTAGTGGATACCCCGTCCTAATTAAGGTAAAATACAAAGACTAATAAGGCTATGGGCC

TATAACTCAAGGCGGTGGTATATCCGAAACCCTAACTAGGAGACTATAACCCTAAGGCGG

CAGGATGTATACCCTTTGTCGTACGCCCGTACCGCTTGAACATTCGTAACTCGTAGAACA

GGCGTGCATCCTTTGTGCCCGGTGACTTGCAGGATAAGTCCAGTCTCTCCAGCTGGACTC

GTATCCCCGTAATAGGAGGTGGCGTATTTTTTTCCACACAGTCAGTCCGCCACTTACTGA

AGCCGACGCAAGCAGTCAGGGAGTGAAAGCGCCATACAAGATGGATATGAAAAAATATCT

CACTGACTCCTTGAGACTTGCAATAACTTTGACCAAGAATGATGCTGATTTCTTCGAGAA

CTCTGTGTGCTTTCATCTGCTTGGTTGATACCGGCTCCATGCACCTTTCCACGAACTGTC

ATTTAATGCTCCGAATCGCTTCATATCTCCAGCTTATGTACCATCTTTATCTCTTTTAGT

CTTATAAATAGAGACTTCATTACAGTAGAAAAACCTCAAATTCTTCTTCAGAAAATACTT

CACTTCTTTAATAATTTCTTATGATAGTGAAAAAATTTCTTCAAAAATATTTGCCTTCGA

TCCTGGTTTGATATTCTAGAAAACTTTCTTTGTTCTGAATGTTCCATTGCATGAGGAGAC

TATAGGATGAAATTCATCTGCAGTTACCTCATTTTCTTACTTCGTGTGCGAGGAAATAGA

TGAAGTTTCCCTAAGTCATGAAGTTTGATCTGGATTCTAACGACGCCCTTTGGGATGTCT

TCATTGTTCCATGTGCACTAATTACGGTGCGTTCATATTCTAACACATCGGTTGCTGTGA

TATTCTTCTTATCTGCGGGGTCAGATCGCTTAGCCCTTTCAGCTTCTGTGTGGCTTCTCC

GCAGGGATTTTTCTGGCGCTTAGATTGCCTGCTTGACGGAGTAGGATTGAAGCGTACTTG

TCTGACTCTGTTTCATGGTAGGCAGGAGGATACAGCTTCACGTTGTTGGGTGATTAAGGT

GTCACTGAGCTTTGGATCTCCTTGTGATTCATGGCTTCAACCTTGGTTATCTGCTTCAGC

TAAATAGTTATTGAAATGCTTCATCGGCTGATGGCTATGGGATACTCGAGGGAAGAGGCT

CCCTTGTGGTAGGTTCCCATCTCAAGCCTCTCTGTTACGGGCGAACTCCTGTTTGTGCTT

GAAGATCTGCAGGGCCATGTACTCTGGTTTGACTAGTTGATGTTGACTTGGATGGTCAAG

TTTTCTTTCTAAGCTCTGCGACGGGTCTTCAAGTTTCTCTTTAAGTTCAGCGGGCAAGAT

GGAGGAGGTTAGGCTTGTACGCAGATTCTTCCGAGGTCCCTTGCTTTAATCAACCAGACG

GGCTTTGGTTGCAGCAACGGCCGCGTGCCTTGAAGGAATGCTCGAAAAGACATATCTCAA

GCTATCAGAAGAGAAGAAGAACTCCGGACTATATACGACTTGTAGCTACAATCCTTATGG

AGGATTTTCTGCCATGTTAATCCTGAGGAGCTCTTTATTTTTAGTCTTCTCTTTCCTTTT

TTTATATAATTGTTATGAACGAATGTTGTATTTAGGAAGAACTAATTGTATATTATGAGC

CTACGGGATTTTTATTAATAAATCAGTCGTTCATCAATATTTTTATACGTAGTTGTGTAA

TTACATGTTGTGTAATGTGTAATCAAGCAATATATTTTGTGTGCCTTTCTAGGCTAGTAT

CTTCAACAGAGAGGTGTGCGCACTGAAGAGCGCTTCATTTGTGTCAATAGGTGACGGCTT

AAGTTGCCCCCACAGTGGGACGACTTTAGCCTATGTTGAAATGGGCACTTTTGTGTCCGA

CTTCGAACGTCTCTGCTTAGTGACTGGAGTATTTAAGACCTTCTGGCACTATGCGAACGG

GCGTCCCCATCGGCATGTAGGTGCATTTGTTGACTTAACGAGTTCAACGTACGAGTAATG

CCTGGTGGCTGAGTTAGACGTGGTCTAACTCTAAGCAAGTAGTATACCCTACTATTTGTA

GCGAAAAGTGAGTGTATTACTTCAACTTAGGTCTGACCGGGG

>XLOC_028868 transcript=TCONS_00047433

ATGGAGCTCTGAAGTATCTTAATCTGGTCTTTAGCCAAATATTATTTAATTTTCTCCACA

AGGTTCTTGCATTGCATCAGAACATGTCCTTAATCATGGCGATGTGTATTCTTCACACTT

TACCAGATTGGTGTCCTAGCCGATAGGTGCAACGGGAAAACATAGAAAGAGCACATGGTC

CCCTTTTATCGACGGGTAAAACTCTTGCTCTTCATATTATTTTTATAAGCATTTGAGTCC

TATTACTTATTATAAATCAACTCGAGAATATTGTTGGATGTATAGCATTTTAATCATACA

AAGAAAGATGTCCAAGTTAGCTGGAGAATGCTGTTGGATAGCTATAAAAAGAAGGATGAC

CAATCACAGTTTGACGGGAAAAACTCATGCTCTTCATGTTCTCCTGATAACCATTTGAGT

CCAATCTCACTTTCTCATACAATTTCTATGATTTCCATTTTATTAGTAGTGAATTGCTAG

ATGTTTAACTAAATAATGTTGTGTTCTCTCTGTTTGTTTTTGTGTTGATCCAAATGTCAA

TGAACTCTGAAGAAATTCAAAGGAGTCGAGCTAAAGGACTTTCTAATGACATGAGCAAGG

AATTTCTTATGATGTTTTTGATGAAGAAGAAGATGACGATGACATGGACCTAATGCAGCT

ACTGTGTAAGCATAAGGATGATAATTATGTGGAACTATTATCGATTGCAAAGTTCAACTC

TTGTGTAATTTCTATTATTTTAGCTGTAATGTGATGACAACAGGTCAAATACAAATTGCC

AGCAAAAGTATAGTTTACTTCTTTTCTGAACGTTGCCTACTGTATCATTCTCATGAAGGA

ATATTTTGGGGTGCCAACTCAACCATTGCTTCACGAGAGAATTGTTTGTTCTTATCTCAA

AAGTACACTAGGAAAGTTTGTATCTAATACTAGATTATTAATTTTATTTTTTGGTTAAGC

AAGGTAACAATGAGAATAATTTACATAAAATATAGTTAAATGGAGGG

>XLOC_009687 transcript=TCONS_00015774

GTAGCAGAATCACTCAAACCTATAAAGGAAAAACAACCACCCTTAGCCCATATAGTAAAA

CCAAAGCCGAACAAAAGCTCAATACTTTATAAGCTTCTCGATCTTTGCCTAACCATGCCT

AATTCTTTCAATATCTTCTTTTCTCTGAGGTAAAAACTCCACAGTAAAATAGTTCTTTGT

TTTCTCTTGTTCTTAATTACCTGAATAGGAAATAGCGAGACAAAGAAAAAGATTATATAT

ATGGATAGTAGTAGCAGGTTGATCATGTGCAGGGGATTTCCGAGCTCATCAGTGGTACTG

CTAGGAGTTATTTGGTTATTATTGGCTAATCAAGCGGCTGCAGCAAGCATGAAGAACAAC

AGATTTATGCGAACAGGCAAGGGAAATGTAGATCTCAACTTCATGAGCATTAAAAGAAGA

GTTCCTAATGGCCCTGACCCTATCCATAACAGAGGAGCGGGCAATTCTCGCCAGCCACAT

GATCATGTTTCCGAAGGAGAACTTGGCAAGCCATGAACTAAAGGCAGACGATCATTATTT

ATTAGGACTGTAGAAAGTTTCCTATAGTATGAGATGAAATTTCAGAACCACAACTTGTAA

GATTTTTTTCTGTTTTTTTACGCTACAAAATTCTTTAGAAGCTTTGGAGGTGGAATGCAA

AGATAGTTATTTTGTCTTACACATGAAGGAACATCAAGAAACAAAAGATC

>XLOC_004617 transcript=TCONS_00007503

CTAATGTTGGCTATTAATTAGGGAAGACTGAAGGAAGGCCGTTTCAGAAATTCTAATTCC

AAAAGCCATGGGTTTTCCATTTCCACAATTTTAGAGTCAAACATTTTCGTGTTATTATCA

ACTGCAAAGAAAATTCGCATTCAAAGATGACAAATAAGGGAAATTGCCCACAGAAGAAGA

AGAAGAAGAAGAAATCAGTAACTCTCAATCAAATTTCTCAAGGAAAGAGCATTATACACT

CGTCAAATAATACGGAGCAGGCCATGGGAAATCACTTCCAAGAGGAAATAGTTATGGAAA

TCCTCAGCAGGTTACCTGGGCGGTCTCTTCTTCGATTCAAGTGTGTTTCAAGATTTTGGA

AAACATTGATTTCTCAACCTTTCTTCACGATGAAGCATCTCAATCATGCCAAGAATGACC

ATAATTCCCAAAAATTTCTTATCAGCCAACGGTGTCATGAGGATCGTAAATTTTCCAGCT

ATTATTGTTCTTTATCGGTTGAGGAAGTGAGGGAACTTGATTTCCCTTTAGACTCTAAAC

CATGGCATTGCGTCATCTGTTGTTGTTGTGATGGCTTGGCTCTTATCGAGGTTTCTGATA

ATGTTGCTGATGAGCACCCCACACTTTTGCTATGGAACCCCTCCACGGGAGAATCAGTGG

TACTCCCTTATCCAAAATTTCCATCGCAGGAATATGCAACTATGGGATTGGGCTATGACT

CAAGTAGTGGAGACTATAAGATCCTCAAGATTTACGAGAACAAAGATGATGGTGACAAAG

TACCTGGTGAAATTCTTGCACTGAAATGTGGTTCCTGGAGAAAAATTGATAAACATCCTC

GTGGCATTTGCAATGTGTTGTCAGGTATGCACTCTCTGGCATCTGTACATGGGGCATTTC

ATTGGATTGGTACTCCACAGAATAATATCGGTACTTCACGAAATTATTCCGTGGTTTCAT

TTAGTATTTCAAATGAGGTGTACGGAGAGATACCATTACCAATATGCTTGACGACCTACC

ATTTTGACAATATTGACGTTTCAGTGTTGAAAGAAATGCTTTGTTTTTGCTCTACTTCTA

TTTATGGGATGGAGGACAATATTAAGTTATGGGTAATGAAAGAATATGGTGACAAGGAAT

CTTGGAGTCCATTGTTTACTATAGGGTACCACAGGATGCTTTCAGCCATACCAAAATATA

TGTTTCCAGATGATGAAGTGGTATTCTGGTGCTTACATACTGGATGTAGTGGGAATTCTT

TTGAGACATCTAGAGGACCATTTGCATTATGGCCTCAAAGTGATACCATCCAGAATGGAT

TCGCATTTACAGAAAGCTTGATCTCTCCAAAATTACTTACTTAGTATTTTGCTTATAAAT

GATTGAGGTATTTTTTTATCAACTGTCATGTTACAACCTCTTGTTCTCATTAGTTTATCT

ATTGCTCACCAGTTTGTTGGTTTCAAATTATATTCAAAACTTCCTGAGATGTACAATTGA

TGGATTTGTCGTGTGTGTGAATTTTCAAGAGGCATATGGGAGAATTGTTTGCTGTAATTG

TTTTGATCATCGATAGGTGTATTTAGTTATAAATTTGTTTTTCTTGTTTTTTTCTCACGT

GAGAAGTGGTGATATGGGAA

>XLOC_021232 transcript=TCONS_00034901

AATTTTCTTCCTCTCCCATATTTTCAAGTTTCTACAAATTTTTCTTTGGGGTTATCACTG

TATATTTTTCAAAGTTTATCCATCTAGAAGTTGTTGTGAAGGTTGTCCATTTTTGTTTGT

CACTGATCTAAGCAATTAGTTGTGAAAACTTGAAGTATCAAAATACCTGATACAAATTTA

GTCCACCGAAGATTATCTCTATCTCCGAGAGGAGACCAGTGACAAGGAAAGAATAATCAT

TGAAGTGGGTTTTTCACCTTGGCGCTTGATAATATGTCTGATTCATTATATTTTGGATGA

ACGAACGACAGCTCTTCCAGTTCCTCAATGGGCTTAATGCTAGCTAATTTGGAGTAATGA

ACTACAGTTCTTCCAATTCTCTACTTGCTGCAGAGTTTTATATGTTCCACAAAAACTTGG

CGAAGATCAAAACAGATGAGATCCTGAACAAGATAGAAATTCAAAGAGGAACATGGTGTT

TTTTTAATCTGAAAAATGATGGGTACAAGTTGCAAAATAAAATGGAGTGTTGCAGGTATG

TTGCAACTTAAGCTTGAAACACAAGTAGGTTGATATTTTCATGGGATTAGCACAAAGAAA

GAATCAAGCAAGGAAGATATGCAGTTCAACAACAGAGCAAACACCCCTCAACTCCAATGA

AGAAACTTTAAGTTATTCTCGATGAAAGATCACCTTTGAGAACTCTCAGCATTTAACTGC

ACACCTGATTCGGGAAGAAATGTTTGTCTATTTTTGTATTTTTCTTGACTTAATTTGATT

TTGTTATTGTCTCATCCATTAATATCTCTTACATGCTACCATTCGAGAACGTTGTTCATG

TTCATTTTGTTTAGAACAACAATTTACTAGAATGTAAAATCTG

>XLOC_022104 transcript=TCONS_00036304

CTTTGGCATTGTTGCCTCATTGGAGATTTATTAGTTTCAGGGGATTTTCAAATGCTTCTT

GATGTTTGCAAATCTTATGGATCTATCTTAAAATGACATTCTCGACTAATTTACTTGCAG

TGAGGTACCTTTAGTAGGTATTAAGTCTCTGTTGGGCCATTTATTCTGTTTTTCATCGTA

AATTTGCTAGTACTCAGTGCCTGATATCCAACTGGTGGTACTTTTTCTTCATCAAATATC

AAAGCTTTCTTCTTTGATTGTAAAAAAAACCGGAAGCTACTCCCACCATCCCATAATTGT

TGTCCTTATGTATTTCTTATATATTTGCTTCTTGACCTTCAGTTTCTTATTAAATATTTA

GTAGATTTCTCTTCTTATTAGTTTCGAACTTCAGTTTGAAACCTAATTCTTCATTTTTTG

TCCATATTATGCTACAACCATGTAATGACTCATATTATGCAGTTCAGTTTGTTTATTAAC

ATGTCAAATTTTACCAATTTTGAACGGTAATCATTGTGAAGAACATGGCCAAAAATACCG

CTTTGGCACAAAACAAGATTCTTCTTCTTTTTACTATTCCAAGCATTATTTTTACCAAAG

CCGGAAGATCTGACCTAGTTGGTAAGGTTCCTTGCATAATATTTTATGGGGTTCCCCTCT

CTGCATATTGGATTTCTGAAGAATACCTTGTGTTACGACGTATTCAACTTCTGCTGAGCT

GCCAAAGGAAACGGATGATGTCAACATGTTGACTTGTAATTCCAGGAAATGAGCAAGACC

TTCCCACTCTTTGGACTACCCTAGTTCATGAGCGAGATCTATAAAAATCCAGAATCATTT

GAATTAGTGGAGCATTACCTAGGATCTTTTTTCTACCCGTACTGCAAGAAACGAGAGCAG

ATATTGCCGAATTTATCTCATTTAATGAAATTGAGCCATATGGATCATTATTTTTTTGTG

TTAAAGTTGATTATTGGAAGAATGTATTTAGTGATGGTAAAGAGATAGAACATTGCCGGG

AGATATTGTTATTATCGCCGTTGCTAAACCAGAAACTGCTTCTGACCTGCTGCGTGTGGG

ATGGATTGGACATTTGCATCTGTCAATGATATCAGTGAAAATGAGAATGATAATCAAAGT

ACATCCAGTAATTTTAGGGTCAAAGCTGCTAGAGATATTGGAATCTCTGAGGGAACGCCA

AAATCATTTTATGTTGTGTATTTGGTAAATGTGATACCGTGTAAGAGAGTTCTAAATGCA

CTTGGAATGAGAGAATTTGAACATTATCGAGAAAGTTCTGTGCACTGGGCATGAGAAAGG

GTGAGGATAAATGTGATATTTGTTCTGCATCCGTTGATGACAGACCAGCTGGAGAAGTCG

TAAACTGTCTGTTTTCTACTTGTCTAAGTTGAATGATTCCCAGGCTGATGCAATCGTGAC

TTCTCTTGATACATTGAAGTGTCGCCACAAGCCTTCTGTTGAACTTATATGGGGTCCACC

TGGTACTGGAAAGACAAAGACTGTAAGTGTCATGCTCTTCATACTATATAGAATGAAGCA

TAGGACTCTTACTTGCGCCCCAACAAATGTAGCAATAACAGTAGCTTCACGGTTGGTTAA

ACTGGTTAGTGATTCATTTAAAAATGATTCTGTCGAAATGGATTTTGCTTGTCCTTTGGG

AGATATTCTCTTGTTTGGGAACAGGGATCGGCTAAAAATTGGTCACAGTATTGAGGAGAT

TTACCTTGATTATCGTGTTGATAAGCTGGTGGAGTGCCTGGTACCTGTGACTGGCGGAAA

CACTATATAAGTTCCATGAACGGGTTTCTCAAAGATTGTTTTTCTCAGTACCGCATCTAT

GTGGAGAATGAGTTGATCAAACTGAAAGGTCTTGCTGACCAAGAGGAAGCTCAGAAAGAA

AAAGCAAAAATCAGCTCATTGATTGATTTGGTAGATCCTGGTTTAATTCTACAGCGTCAT

CTTTGAGAAGATGCATGCTTATATTCTGTACTCATCTACCAGTATGCTTTATTCAGGAGG

AGAACTTTGATAGAATGGTGCGTCTTATCTCCCTACTTGATTGTTTGGAGCGAAAGTTAT

ATCAAGAAAATATGGGATCTAAAGAACTGAGGAGCTCTTTTCGTGTAAGCAAACGAATGA

ATTCCCTGCGTCATCTTTGGACGAGTTGTCATTACCTTGTTTGAGAAGCCAGTGCTTCAT

TCTTCTGAAAGATCTTTTCCGATCTCTTGGAAAGCTGAGTTTTCCCATTGCAATGAGCAG

GGAGTTGATTAGGGAGTTCTGTATCCAAATGGCTTCATTGGTTCTGTGCACTGCTTCCAG

TTCATATAAGCTTCATTCACTGGATAATGAGCCATTTGACTTGTTGGTCGTTGATGAAGC

TGCCCAGTTGAAGGAATGTGAATCAGTCATACCCCCTTCAACTTCAAGATCTGCGGCATA

CCATTTTGGTGGGTGACTGGAGGAGCTCTTTAGATTTGACAAATTGAGCAAGGCAGCAAT

GAATCTGACTTGACCAAGCAAGAAAAGAAATTGGTTTAATTTTAACTGTTTGCAAACATG

AAATATTACAGATCATTCAACACTTGATATGTTCTGAAAATTTTAAAGTAGAAAAAAATG

GAACGTGTGCAACCTTTTCTTCGGAATTGTTAAATGGCCATATGCCAATCTTTTGCAAAG

TTGGTCGCCACATAATCATCTCTCAAAATAGCCACCATTTGGATAAAATATCCATCATTT

TTTATAAAATTATTTC

>XLOC_005901 transcript=TCONS_00009613

GTTCTAGTTGTTCTTGATGATGTGGATCATAGAAGGCAATTAGAATCCTTAGCAAGAGAA

AGAAGTTGGTTTGGTTCAGGTAGTGTAATAATTATTACAACCCGAGACCAGCATGTGCTA

TGTATGCTTAGAGAAAATGAGAGACACCAGGCCAAACTATTAGTTGATAATGAAGCTCAG

CAACTTTTTAGTTTTCATGCTTTTAAATGTCTCTCTCCACCACAAGAATATCTTGAGCTG

GCTCAAGGAGTGATTGAATATTCAGGTGGGCTGCCGTTAGCTCTTGCGGCATTGGGGTCA

CATTTGGAAGGGAAATCTGTAAGAGAATGGGGATGCGAATTAAAAAGACTGAGAGAAATT

CCTCATCGTGATATTCAAGAGATCCTCAAGATAAGCTTTGACGGACTTGATTGTTATATT

CAAAGTGTTTTCCTCGATATTGCATGCGCCTTTCATGGATTTAATAGACAACAAGTTATC

GAAATACTAAATGCATGTGGTTTTCATTCTGAAATTGCAATTGCAACTTTAGAACGAAAA

TGCTTGCTCCAAAGGGATAGGTATTGCTTGGTGATGCATGATCTAGTGCGAGATATGGGA

AGAGAAATTGTTCGCATGGAATCACCCCAATATCCTGGAAAACGGAGTAGATTGTTCAAC

CCTCAAGAAGTTTGTGATGTTCTACAAGGAAATAAAGTCAGTAAATTCTTTATTTGGTTA

AACTTTCGTCTTTTTAATTTGTTTTTATTTGCTTATAATTTCTGTTGTCACCACAAATAT

TCAATTCCTTTGTGCTTCTTTTGGTTGGGTAATTTTTTCTAGTTGTTTGCCAACCATTGT

GGACTAGAAATGAGTAGTATTAGCTAAAGTCGTAAATGTATACTATCAAAGCCAATATAT

TACTACTACTATAGAAGTTCACCTATTTAACAATCACTCAAAATGGTTTGGTGATGCACG

TATATTGTCATAGACTATATTCTTTCCAAGATTAGATTTTCCTGAATCTGTTTTTTTTTT

CTTTTCCAATTGATCATTTTATCTTTTGTGTATATTATATATTAGGTAGTATGAGGGTGT

TTAGCTAAGCTTATAAGCTGGTAATCATTTTTTGTCTTATCTTCGCGTTTGGTAAAATTA

AAAGTGCTTATATGTCAAGTGCTTTTAAGTAACTTTAACATCTTATTAAATTCTCTAATG

TGTAAATTCATTTTTCTTTGGTGCATTATTGTATTCTCTTTGTTTGACCTTGAATCTGCA

TAAAGCATTTTCTGATCTAAATTTCTGGGACTTAACATGGTCTTAGGGAACTTGTCTTTT

TACTGAATACAGGGTTCTGAAATGGTAGAAGTACTAATGGTAGATCCACGGGTGTTCAAG

GGTGTGAACTTGAGCATGAATGCATTTGAGAAAATGAAAAATCTAAGGGTGCTTAAAATC

GACAAAATACATTTTAGTGGAGATTTTGAGCTGTTGTCCAAGGAGCTTAGATGGTTGTCT

TGGCAAAACTGTCCTTTAGAAGATATACCATCACATTTTGCAGCCGAGAAACTTGTAATT

CTAGATTTGTGTGGGAGTAAGATCCAAGAATTTGGTGCGGATTTGCAGGTTTGTTACTTA

ATTTTACAACTTCATGCGTATTGTGATTGCAACTGAGTGAAAGAAAATCTTACTTGAGAC

AATATTTTGTTTTACTTTTAGTGCTGTAAAAGTTTGAAGAAGCTGGATCTCTCTGATTGC

AAGCGCCTCAGAAGAACTCCAAACTTTGATGGTTTGCTAAGTCTTGAGATTTTGTGGCTT

CGTGGTTGCGCAAGTCTGATGGAGATCCATCAATCAATAGGAAAATTGGGCAAACTAATT

GAGCTAGATTTGTCACGATGCGTAATGCTTAGGGAATTTCCGCGCAGCATGTCCCACCTA

AAATCCCTTGAATCCTTGGACATTAGTTGCTGCTCATCTTTACATAGACTGTCATTTGAC

CTTGGAGATTTGCAGAGTCTTAAAACTCTTAATGCACATCAGACGGGTATAAAGCAATGG

CCTACATTTGATGAATTGCTTAGAGATCCTGGGACTTTGAATGTGGGAACCGAAACGATT

TTATATGGAGGAGAAGTTCATAGCATACAATCCTGGCCAAATGTCCTTCAGCAATTAACA

CTTAATTTGTCCGAAGCTGATATTCCTAGAGATATTGGAAATTTATCCTCCTTAGAATTT

TTAGATTTGAGTGGAAATAGATTTCGCTTTCTAACCTTTGATTTTTCTAAGTTGCTACTG

CTGAGGGATCTGCGTTTAAATAACTGTCCGAATCTTCAAACACTCCCACCAGTCGCAAAT

TTAGAATATCTTAGAAATTTTGAAGTTAGGGATTGCAAGAGATTGGTCGAGATTACAGAG

TTGCACAACCTCCCTTCTATAAGCCGGATCGACATGCTCGATAGTAGTTCTCTGCAGAAT

CCGTTCAATAAAGGCTTTTTCAGTGCACATGCTCTAGCACTCCAATCTAGAGAAGATCAA

AATCTGGTCAGTCTCTCTCTTTCTCTCTTTATTTGTATATATGGACTTACCAATCTCCCC

ATTTTGGGAACATAATATTATGCATCTTCTTCAGATCATGAAGCCGGAGTTAAATATTTA

TCTCCATGGCAATGAGATCCCACATTGGTGCAGCAACACAGTAACAGATTCATCTATCTG

CTTCACTATGCTTACACATAATAAGAAGTATAAATTTTTAGGAATGGTTCTCTGGTTTGT

TCGCAAGTTCGACAAGCAACCCTTTTCTCCCCATAAGCCACTTGGCTTCAGCTTTAGTAT

TGACAACAAAATGTTTGCTAGTACAATCTGGCCAATTCATACTGTACCCGCTGAAGAGAT

GGAAATATCATATGTATTTTACAGATCTTACTTGGAGAAGCCTTTTGATGGGCAAATGAA

AGGCGGGGAAAGGATAGAAGTGGTCGAGAAGTATGATAAAGAAAATACAGTGAAGCAGAT

AGGGATCCATCTGTTATATTTAGACCAACAAGGTACAGTTATATCATTACCACCAGCAGT

TTTGGTCAACCAGACACCTCTGGATGTTGCTTGGCACTCAGTTGGAGTTGATTCCCGTGT

CAATGATATAGAGTTGTTATTGCAAAGAGGAAGTGAAGATGAAGTTCGCATGGTTGGTAT

ATATGGTGTTGGTGGAATAGGGAAAACAACTCTGGCAAAAGCTATCTACAACCGAACATT

TCGGGATTTCGATAGTAGTTGCTTCCTTTCAAATGTAAGATCAGAATCTGAGCAGTATAA

TGGTGTAGTCAAGCTACAAGAGAAACTTCTTCATCAAATTCTGAGACACGAATTTAAAGT

TAGCAGTGTTGATGAAGGCGTCAATCTAATCAAAGCAAGACTTGGGACAAAGAAGGCTCT

AATTGTTCTTGATGATGTGGATCATAGAAGTCAATTAGAATCCTTAGCAAGAGAAAGAAG

TTGGTTTGGTTCAGGTAGTGTAATAATTATTACAACCCGAGACCAGCGTGTGCTATGTAT

GCTTAGAGAAAATGAGAGACACCAGGCCAAACTATTAGTTGATAATGAAGCTCAGCAACT

TTTTAGTTTTCATGCTTTTAAATGTCTCTCTCCACCACAAGAATATCTTGAGCTGGCTCA

AGGAGTAATTGAATATTCAGGAGGGCTGCCGTTAGCTCTTGCTACATTGGGGTCACATTT

GCAAGGGAAATCGGTAAAAGAATGGGGATACGAATTCAAAAAACTAAGAGCAATTCCTCA

TTGTGATGTTCAAAAGATCCTCAAGATAAGCTTTGATGGACTTGATAGTGATACTCAAAG

TGTTTTCCTTGATATTGCATGCCCCTTTTATGAATTTTCAGAAGAAGAAGTTATCAAAAT

ACTAGATGCATGTGGTTTTCATTCTGAAATCGCAATTGCAACTTTAGAACGAAAATGCTT

GCTCGAAAGGAATAGAAACATCGACAGATATGGTTGCTTTGCGATGCATGATTTAGTGCG

AGATATGGGAAGAGAAATAGTTCGCATGGAATCAACCCAAGACCCTGGAAAACGGAGTAG

ATTGTTCGACCCTCAAGAAGTTCGTGATGTTCTACAAGGAAATAAAGTCAGTGAATTCTT

TATCTTTATTTCGTTATACTTTCGTCACTTTTTTTATTTGCTTATAATTTCTGTTGTCAA

CATAAAAATTCATATACTTTGTGCTTCTTTTAGTTAGTAAATTTTTTCTAGTCGTTAAAT

ATATCTATATTAAATATAATGATACCTCCACCGTGTTCAGATTCTTGGTCTGCATCTAGA

TATATATATAATTTGTCTTGAATTGGTACTGTTATCAGGTTTCTGGATGTTATTATCCTC

TTTCTTCTTCTTAAATAAAAATTTACTTTGATATTATTTCTTACTGAATGCTGTTAGCAT

TTAAAAGACTCAACGTAGCCTTTTAAACAACTTCAACATTTTTTTACATCCTCTAATGTG

TAAATTCATTTTTCTTGGTGAATTATTGTGTTTTATTTGTTTAACCATTAATTAGCATAA

GGCATTTTACTGAATACAGGGTTCTGAAGAGGTAGAAGTACTAATGGTAGATCCACGGGC

ATTAAAGGGCGTGAACTTGAGCACCAAGGCTTTTCAAAAAATGAAAAATCTTCGAGTGCT

TAAAATCAATGAACTAAAAATCAATGGAGATCTTGAGCTGTTGTCCAAGGAGCTTAGATG

GTTGTCTTGGCAAAACTGTCCTTTAGAAGATATACCATCACATTTTGCAGCCGAGAAACT

TGTAATTCTAGATTTGTGTGGGAGTAAGATCCAAGAATTTGGTGCGGATTTGCAGTGCTG

TAAAAGTTTGAAGAAGCTGGATCTCTCTGATTGCAAGCTCCTCAGAAGAACTCCAAACTT

TGATGGTTTGCTAACTCTTGAGATTTTGTGGCTTCGTGGTTGCGCTAGTCTGATGGAGAT

CCATCAATCATTAGGAAAATTGGGCAAACTAATTCAGCTGGATTTGTCCTACTGCAAAAG

GCTTAGGGATCTTCCCCGCAGCATGCGCCTCCTAAAATCCCTTGAAGTCTTGAACATTAG

TTGCTGCTCATCTTTACAAAGACTGTCATTTGACCTTGGAGATCTGCAAAGTCTGAAAAC

TCTTAATGCAGATTTCACGGGTATAAAACAATGGGGTACACCGCTAAGAGATCCTGTGAC

TTTGGAAGTGGGAGCCAAAAAGAGATTATCTCAAAGAGAAGTCTATTGCATACCATCCTG

GCCAACTCTTCTTCGGAAATTACAGCCTAATTTGTCCAAGACTAATATTCCCTTGGATAT

TGAAAATTTAAATTTGAGTGGCAGATTTTACTGTCTACCCTTTGATATTTCTAAGTTACT

AAGGTTGAGGGGTCTGCGTTTATATTACTGTCCGAATCTTCAAACACTCCCACCAGTTGC

AAATTTAGAATATCTTGAACTTTTTTTAGTTAGTAATTGCAAGAAATTGGTCGAGATTAA

AGAGTTGCACAATCTCCCTTCTATAAGGGAGATCGAGATGGTCGGTAGTAGTTCTCTGCA

GAGTCCGTTCAACGAAGGCTTTTTCAGTTCTCATGCTCTAGCACTCCAATCTAGAGAAGA

TCGAAATCTGGTCAGTCTCTCTCCCCCTACAGACGGAGTATATATTTTATGTCCTGCTAT

AATATGCTTCTTCTTCAGGTCATGAAGCCGGAGCTACATATTATTTTCCACTGCAATGAG

ATCCCAAATTGGTGCAGCAATAGAGTAAGAGCTTCGTCTATGTGCTTCACTATGCCCACA

TATAATAAGGAGTATAAATTTTTAGGAATGGTTCTTTGGTTTGTTTGCAAGTCCCTGGCC

AGTCCTTGGTTTTCAAGCAATAATAAGCGACATGGTTTCAGCTTTAGTATTGCAGACAAA

ATGTCGGCTGGTACAACCTGGCCATTTCATTTTGGAAGCGACGAAGACGCAGATATGGCA

TATGTATTTTACAGATCTTACTTAGAGACACCTTTTGATGGGCAGATGAAAGGCGGCGAA

AGGGTAGAAGTGGTCGAGAAATATGATGAAGAAAATATAGTGAAGCAGATAGGGATCCAT

CTGTTATATTTAGACCCACATGGTAAAATTATATCATTACCAGCAATTGTGGATCAGGGG

CGGGTTAACCTCCAAGCGCCTAAAGTTAACCAAGGAATGAAGAAATTCATAAGGAGACAT

ATATTATATGTTGATTAGTGTTAAATGGATATTATTTTTAGTGATAAGTGAATATTTAAT

CATTTGTTGTGAATTAACTCATTGCGCATTATTCCCAAAACTTTTACTACATTGTAAAAA

CTGTGTGCAATTAAAATTGCAGTGACTTACATAATGACGATCGAATATGGTAGTGTGCTT

>XLOC_003909 transcript=TCONS_00006367

TCCAAGCCCATTCTTTGTATATAATATCATTTCACCCAATTTTCCCCTTTTCTCTCAAAA

CCGAGCGCCCATTCAAAATTTCCCCTTTCATTGTCTCACTCAGTAAAACCTATAAATCCA

TCCTCCCTCTCGCAATTGAAAATCCAATTGGAGCCAAAAGTTCCATTTAAAGAAAAAATT

GGCCCTCCTTCCAAGAAGCCCTAACCAGCGGAATCATCATCTTCGTGTAAAATTTGCAGA

ATCAGACGTCATTCATGTGGATCTGAAGAGTAAGAAGTGATCTTTGAGTGGCAGAACCTC

TCTGCGAAGAGCTTACTTTATCTTATAAGTACCAAGTCTCGTACAGGTACAATATTTTAA

AGACTATCACAGATGGCGTCAACTAGAAATAAGCTTGAAGGCACATAATGTTTTAATTTT

TTTGTCATGTGCAAGTGTACAATGACAATGTAACAACTTGAAGGCACTTAGAGTTTTATT

TTTTTGTCATGTATAAGAGTACAATGTGTACAATGACAATGAATTATTGTATAATATAAT

TTTTTGGTATCGGCTCC

>XLOC_000253 transcript=TCONS_00000425

CTCCTTAATATCTCTACTCTCTCATAATGTTGAAGACACCAACTCTTCAAAGAGGCATTG

ACCAAAACAATTCTCTCATCTCTTGCACAGAAATTAGTAATTAAAAAAACACCAAACCTC

CTTAGTCCTTTGCCAAACTGTCCCCAAATTACATAAATATATCCATTACCAGAACATCAT

CAGACAAATTTCATCTGATAACATCTTTAAACAAAAATCTTAATCACATCAAACCACAAA

CATCAATGTCTAAAATCACCATTACACTCAGCATCCCTCTCTTAATCACCTTCCTAACAA

CTTTCCCGCCGTTAATCACCGCCGTCAACGGTGGCACTTCCCCCTCCACCACCACCACCG

CCACCGCATACGACATAATCCAATCCTATGACTTCCCTATAGGAATTCTCCCAAAGGGTG

TTACAGGATACGATCTTGATAAATCCACCGGAAAATTTAATTTATACCTTAATGGCTCTT

GTAGTTTTTCTCTAGAAGGTTCTTATCAACTCAAGTATAAATCAAGAATCAGTGGATACA

TAACTAAAGATAGACTTACAAGTTTATCTGGGGTTAGTGTAAAAATTCTGTTTTTATGGC

TTAATATAGTGGAAGTTGTGCGAAAAGGCGATAATCTTGAGTTTTCGGTTGGAATTGCTT

CAGCAAGTTTTACTCTAGATAATTTCTTGGTTTGTCCACAATGTGGTTGTGGATTGGATT

GCAATAGCATTAGCAATGGACGTATTGTAAAGATTGGAAATAGTCATCTTGTGTCTTCGG

TTTAGCTGCAGATGATTGGTTTTGCTGAAAAAGATACTTCCATATCATAGTCAAAGAAGT

GCTGTGAGAGCCTCAAGTGATCGTTGTTCTGCTTCATCCTCCCAAATGTGAATTACAATG

CAAAAGTGAAAGTCCAGAATTCCAGTGTTCATCTACATTGTTTTTATGTTTTTTACAACT

GTATCTAACTCTGACATCAATAGACAATACTCTTGAAATTGCTTTCTGCACGAGTTACTT

ATTCACGTTTTATGACTCTTGCCAAATTTGATTCTTCTCACCGATGGGAGTTGTGCATAG

GGTTGGCTCATTGTTTTCTTGAAAAGCTAGGTGTAGCTCGTGCCCGCACTCTGGTAAATT

GGAGGCCAAAAAGTCAAAAAGTGGGAAGCTAACAAGGATTAGGTGACGTGCACTGTAACT

CTTTTGTCATAGCATCTAAAAAAATATGCAATGCATCTTGACTCAACTTAGTGATCAGTG

ATA

>XLOC_027769 transcript=TCONS_00045675

TATCACGTAAGAAAACAGATCGACAAAATAGGACTTTCAGGTGAACAACGTAAACATCAA

AACTGAAAAAACTCAAACACGCTCTCAGCCTAATCCAAATCCCCCCAGTTCTGCAATTTT

AAGTTCCATCACATGGATATATATTCTCCGAACTCCAGGATTACTCTCTGCACCTAAATC

CTTTTAAACAGAATCTACAGTTCACTCTCAAGCACTTCAATTTATAAATTCTTGGAAGAA

AATCGGAGAAAAACTCAATTTTCATAGTCAAACAGTCTTTGATCCTAAGTTTACTTTTCA

AAGGATGCTCTCAAATTAAAGGATTTTCATCGAAGAAATATAAGTCGCGTTGAAGGTTCT

TCTTGTCGTTGCTTCGATTCGGAAAGAGACGCTTAATCTTCTGATTTAGACAGGCATGAG

TAAGAAGGGAAATTCACAACTGAAACAGAACGAGAAAATGAAAAGCTCGTCGTACATAAA

AGCTGGAACACTCAGATCTTTAGTTAGGAGGCTTTCTTCGGGTACGAGAGTACAAAAAAC

TGAAGAACGTATGCTACATTCACTTGAAAAGCAACAACCACAGGCACCACGTCGTCCACT

GGTAGAAAATTATACTCAAATGGAAACATGCCCTTTTGTTGAATAAGAAGCAGAACAAGT

GCAACCAGATTTGACCACACCTGAAACAACTAAGCAAGTTGAAGTACAAGGCCCTTCCAC

TCAAAAGAGAAAAAGAGGCAAGACAAGAATGCAAAATGTACATGGATGCTCTGAGCGTAA

ATTGATTACTTTGAATGAGTTCAATCAACCCGTTGGTCCTACTGAACCTGTCGTAAAAGA

GTTGTCTAGTTTCTTGGGCGCATTGTTAAGAAATCCGACACTTTGCCCTCTTGGCACATT

TGATTGGAGAAAGATGAACATAAAAGATGATCTCTGGAAAGTTACCAAGGTTTGAAGTTC

CTAAGACACACACACACACACACACACACACGTGCAAGCACACTCATACACACACACACA

TATGCAAGCACACTCATTCACGCACAAACACACAATCTGTACATGCAAATATAATTATAA

ATAGTGCAAATGGAATGTAACTTATTTGTAGGATGAATATGATATCCCGGATGCTGCAAA

AACATGGGCTTTGGAAGCAATTCACACTGCTTGGAGAAGGTACAAGAATCAATTGAATAA

AGAGCACTACGATGCATACGTCACAGATGAAATTCGAATGTCAAATAGGCCCATTGAAGT

TCCAAAGTCACAATTTAAGGATCTCCTTGAATATTGGAATTCCGATGCAGTCCAAGAAAA

AGAGAAAGGTTCTTCGGAGGCTGTATCGAAAAAGAATGTGTTTGTTCGTACTAGAGCAAG

GAAAGCTAACCGATCATACAAGCTCTCAAATGAAGACACAACCAGTAAAATTGATGAAAT

GGAGAAAATAGAATCGTAAAAAAGTGGAGACGAGACTGAACCTATTGACCCATTTGCGAC

TGTGATGGGACCTGAACATCCAGGGCGACTAAGGTTGTATGGGCGTGGGGTGACAAAAAC

TGTTTTGAAGGGTAAAATGGGACAGGCAGAACCTTCTTTAAATGCTAATGATGATTTCAT

GCAAAAAATAGATGAGATGTTGAACAAGAAATTAAAGGAACACAAGGCAACTATCCGACA

AGAACTTTTGGTAGATATGATTGAAAACTTCAGAATTCAGGGAAGCCAATTGATGCTGAT

ATCCTAGCAGCATTATGTGCTTGTTCTACTGAAGAAGCTTCAACTTCGTGTGAGAACAAT

ATAGGTGGTAGACCAAATGAAGGAGTAGGTGCAAGTGATGAAAGCACTACTTAGCATTAT

GGGAATGTAGTGATTGAAATTATAAGTTTCTTTAAATTTTTTAGGAATATCTGTTTTCGC

ATTTTTTCAGTACTTCGCAAAGTTAGATATGTTTCCACTTTGCATTGTATAGTTTGCTTA

ATGAATATTGTAGGATAATTCGAATTATGATTTGCTTTATTGATACTTCTTTTGTCATCT

ATACAGCTTTTGGTGCATTATATGTATTCTTATAATGTAAATGCCATATCCTGAAAACCA

T

>XLOC_019673 transcript=TCONS_00032238

CCCAAAGCAATTCACACGTCTATAAGATTTTCCCCTATATAACCAATTTCTTGTATTTGC

ATACTCATTTTTCTTTCTAGCTTTACATTGACAAATAAAGAGGGAAGTTCATTTCACAAG

GTCAAAACATATATTATATGGGTCATCACCACCATCATCATCACCACCATGAAGGTCCAT

ATGATGATCCATTCTTGGCATGTTGTTGCTGCCCTTGTTTCCTAGTTTCCTCTGTGTTGT

CTATGTTCACAAGGTGTATCTTTGTGGCTTGTTACCCTTTTCTTCAGTGTTTTGGATGGG

ATGAACACGG

>XLOC_020401 transcript=TCONS_00033475

TGGCTCTTTTTGTATAGAAATTTTATTTCTCTCTACCCCTCTTACATTTTTACTCTTATC

TGTTACTAAATTCTTGTGTTTGATGTTTTTTTAGCAGAAGAATATGGACTTTCCTTAAAA

AATAGATGCACGTTGTTTGCCCTAAATCATCTGTGCTTGTGGATCGATTATTGCTGCTAT

TGAGATGATCAAGTTCATACTTTTGTCATCCTTGTATGATTTTTCTCTGTAACTTGGTGC

ATCCACCCATTTACCCAAAAGAATTTTGCAAACACACGTGAATGGTATAGTCTCCACAGC

AGAAATCACGGAGTAGAGGTAGACACCAATATTTTGTATTCATTTATTCAATGCCAGGCA

ACCAGGAAGTTGACAATGACGACAATGCCAGATAAATAAAACAATTTGGCACAAATTGCT

TTCACTAAAGGTGTAAAATCTTTCCTATTATTCTCAAGTAGACTATGCACCTTTACTATT

TGATGATATGCCTGAATGATTTCCTGCTCTAGTTTTCACATGCAGCCAACTTCGTTAGTG

TTTGCCGAAAAATATGTATTTCATCTCCTCAGTCATGAAGCACTACTGACTACTGTCAAG

CCAAATATTAATTATGCAAGGTTTTGTTGTACTATTATTACACCAATGACTGAAGATAAA

ATTCAATTAACATCTGTATTAACATCAAACAACTCAAAGCTAATGACTGTGTACTAACAC

TGTCTCCTTTTTCATTTTTTCTGCCAAAGCTAATGACTGTGTACTCGAACATTGTCTCCT

TTTTCATTTTTTCTGCTAACTAGGTGGATTTCCACTGTTTGGTACCACTGGCTAGGGAAT

AATCTGAAAAGATCTTAAGGAGCTTGAAATTGTTACACAGGTGAATAAGAAGAAAGTACA

TCGGAAGTATCCAGCTGTCTTCTTTGAGGATTTGGTAACACAACTTCACAATTCAATTTC

ATGATATTTGTTGGAGGAGAGAAATATCGTTCTAGATTATATATCAGTGGAAGAGAAATT

ATAATTTTCTGCTACTGTGCTACCAAGTGCTCTAGGTGACATTAGTTTCAGCCTTTGATT

AATTTTAATGAAAGCCTCTTTATATTCTAGCTTCATGGTTCCTGCAACACTTAAAATGTA

GCAGTACATATGCAGCTCTATGGGTGTGTCTTTCTGTAGCAGTTTCGAAGTTGTTGTATT

TCTTTTTAAGCTATATGGAGAGTTGTGCTTGACAAGAATTATGTATCCACGTGAATGATA

AAGGAGATTCTCAAATTTTAGGAAATGTGCTCCATCGTTCAATTTTCACTTATATTAGTC

TTTGATGCAATCTTCAC

>XLOC_037400 transcript=TCONS_00061236

CTCATTTGGTAAATGTTACGCTAAATAATTCAGTCTATGTATCTCCAAAAGGAAGAAATT

AAATTGCAGTTTTAATACATCAATATAAAGAATATCCTCAACATACTACAGGAGGATTAC

ACTATTCTGTTTCCATGAATACTATAAAACTTTAATTTAAAAAAAAAAAAAGAAGCCTGA

TACATAAAGTATCCCGCGTTTATCCACCTTCCAAAATTGCAGCTTCTCTTGTGCTATACA

CAAACAAAATGTTAAATTTCCTGCCTCACTCTCACTTAGTAGAAAATTCAGAAGCATCAA

TTGAGAACTCTTCACTTCAGTGACCAACATTGTGGATAAGCTCCATGAGTTCACTTGCTA

CCTAATGAACCATTTAAACCATACATAATTCCATCTGAGAATTGATACACGTTCTTTGGG

TCATCTGCATCCGCCAATATTGAAAAATTCCTCTAGCAGACAAATTATCATTGACTTCTA

GAAATTATTGGGATAGGATGAGCTTCTGTGTTGAATACATACTGATCCAAGGGGATCGTG

GTGCTATTGCCGAAGAGCAAATAAAGGTATTTGAGCGTTTCACCCAAGAAAAAGGTCTCC

ATCTTGTCTCTTCTTCGTGGAGGAAGCATAGTAACATCGTCAAGAGAAGTGTAGCCTCCA

GAATCAACCTTTGTGTACTTCTCAAATGCCTCAAAAATTTGCCAGCCCCATTCACGATAC

TTTGCATCTTCAGTAATACGATACAGTACAAACAGTGATTCAACGGTTTCAGGACGCAAA

AGATTGTGACGATCAGCAGGCTTTATGATTATGTCATTCAAATATTTTGAAGTTCTGTTT

CCGCCGTCAGGACCACCTTCAGAATCTCCCTGCTAGAAGAAAACAAGGTACAAAGCCATT

AATAATGCAAGAACTTTCCAATTACGTCACAATAGAAGTACTCGAGTTAGGGTAGATATA

GGGCAGCCTGGTACACAAAGCATCCCGCGTTTACGCAGGGTCCTGGGAAAGGCCACACCC

ACCCCAAGGGTGCGCATCAGTGGCTATTTCCATAGCCCTTGACTGGTTGCTCCAAGGCTC

CCCTTCTTACCTTTCATCTCTATATTGATGAAATTAGATCATTCAAGTCAATATAGGCAC

ATAACTCTGATGTTTTCCAGATTTGTGTGATTAATTGGACTAACATTAAACAGTTATTAT

AACATCTTTTACAACTGCTGAATCCGTATTGCATCAAGAAATAAAGATGTTAAACATAGA

TCAGCATCTTAGAATTTGCAACAGGATTCTTTTAACAAACTGCTCCTAAAAAAATAAAAT

TAGAACAAAAGAGAAGATCAAGGAAACTGAAAGACACCACCTTGCATAATAGTTAGTAAA

AAGTGATGAAAGCAAATAAAAAATGTTCATCTCACCTCAATATTGAAATAAGCTATTTCT

GGAGCAAGTCCAGTAGTGGTTACTGAGTATATTTCTACACACGTCTTCGCCAGATCTTCA

GCAAGCTTTAGGTTTTCCATATCTTCAAATGTGAGCAAGTTCTCTCTCATAGCCCTTTGC

TTTGTGAACCCCTTTGTAGCACCAAGGGCCAGGGTACCAGGAAGGAAACACACCTGAGGA

TGAAGTGAAATGATATCA

>XLOC_013971 transcript=TCONS_00022878

GAGAAGGAACACAGAGTTGTGCAATTACTCAAGATCGGTGAAGTACCTCTTCAAATATCT

GCATAAAGGGTCAGATAGGGCTACTGCAACTATACAATGTGCACATGCTGGTGGGGAGAT

GGATGAGATCAAAAGATATCTCGACTGTAGATACATATCGGCTGCAGAAGCTTGCTCGAG

GATCTTTGAATTTGACATACATTACAGGAAACCATCAGTTGAACGATTGCCATTCCATTT

AGAGGGACAAAATACATTAGTATTTGAAGAAGCAAGGCGAGCTGAAAACGTGCTTAGCCA

GCCTGGAATAGAAATTACAAAATTCACTTAATGGTTTGCGACTAACAAAAAATTCCCTGA

TGCACGAATGCTGACATATTCGGAATTTCCAACACGTTGGGTCTGGAATGCAACTTTAAA

AATGTGGACAAGAAGAGAAAGTGGGAAAGCAGTAGGTAGAATCTACTTTGCACATCCTTC

AAGTGGAGAACGTTTTTATCTGAGAATGCTACTAAATTTTGTCAAAGGGTGCACCTCATT

CGAAAGCATTCGAACTATAAACGGTGTGGAGTATAAAAGTTACAGAGAGGCATGCTACGC

ATTAGGATTACTAGATGATGATAAAGAGTGGAATGATTGTTTGGATGAGGCGGCACACTG

GGAAAGTGGACATGAGTTAAGACATCTCTTTGTAACAATACTTATTCATTGTCGAGTATC

TAATACAACCAAACTTTGGGAGAGCAACCAAGGAATACTATCAGAAGATGTAACATCATT

ACAGAGAAAAAGCATTCAGA

>XLOC_004012 transcript=TCONS_00006534

TTGAAAAGCAAAAAAAAAAAAAAAAAAAAAAAAAAGAGAGGAATGAACATGTGTAAGAAT

GTTGCAAGACTAAAACCTGGAGAAACTATGCGGAATGAGCTATCAAAGAAAAATTGTTGC

GTGCCTCCTAGTTTCGATCAAATGGACAATGATGTTTCCCCCGGTCAAGAAGCTGATGTT

ACGCAAGATGATCATAGAATTGACCTAAGTCTATGTGAATCAGAAAAGGCCAAAAAAGTG

AGAGGAATGAATATGTGTAAGAATGTTGCAAGACTCAAACCTGGAAAAACTATCCGGAAT

GAGCTAGCAAAGAAAAATTGTCGTGTGCCTCCTAGTTTTGATCAAATGGACGATGATGTT

TCCCTCGGTCAAGAAGCTGAAGTTATGCGAGATGATCACAGAATTGGCCTAAGTCGATGT

GAATCAGAAAAGTAAAAAAAAGTGAGGAATGAACATGTGTAAGAATGTTGCAAGACTAAA

ACCTGGAGAAACTATCCGGAGTGAGCTAGCAAAGAAAAATTGTCGTGTACCTCCTAGTTT

TGATCAAATAGAAGATGATGTTTCCTCTGGTCAAGAAACTGAAGTGCTGCAAGATGATCA

GAGAATTGACCTAAGTCGATGTGAATCGGAAAAGGCAAAAAATGTGACAGAAAAGAACAT

GTGTAAGACTGTTGGAAAGCTGAAACCCGGGTAAAAGTTAAGAGTTACTTTTTACCACAA

TCGAGTCGTTGGAGAGAATCATGCTACTTTTACGAGGCACTTAGGTTTTTTAGTTCGTGA

TTCTAATATGTGTCTCTTCCGGGTACACTCATGGACGGACATCGAAGAGTATAAGCTGGA

GCACATATGGGCAGCTGTTATCGTAAGAATCTAAAGCATTACTTTTCTTCATTTTTTCAG

AGAATGAATAATCCTTTAAAAGTAGTTAATGTGTATTTAGTCAAGCTCTATATCAGCAAA

GAGAATCCAATGGATTAAAATTATTATCAGCTGATGAGCGAAACTGAGAGACTAAAACAG

GAAGATATTCCTCTTGCAATAAACCATGAAGCCATTTTGTTAGTAATAACATTTGTAAAA

AAATCATTTTTTTTGTTAATAAGTAGATAATATTATTATATGACATCAATAAGATAGAGC

TTACAAAATTATAACAGATCTAGAATAACTCCCTATTAGCTGCTAGAACACTATTGTGGT

AATAAATTCTATTAATTCAGTTTGCTCATGATATATTCTAGGAAAAATTTGACAGTGATG

ACATGAACGATCATAAAGATCATGTTCTGCAACATATGAGAAGGCTTTGGAACAATTAGA

GAGGATCATTGCACAAGAATGTGAAAGCTAAGCCATTGCACGAGGTTCTAAAAGATGTGG

CAGAGAGGGTAGACAAAAGTGATTGGGAATGGTTGGTCAAGGAGCACTTTTTATCTGATA

AATTTAAG

>XLOC_010823 transcript=TCONS_00017667

ATTCGGAACAAATGAGCATGGCACTATTAAAGTCAAAGACATTCATTGTGGCTACAACTT

CAAGATTGAATTCTTTATACAAGGGACATGGTAATCAATATGTATAACGGCATCGACTTG

CGATTAACAACACTAAACTACAATACGAGAACAAACTAAGGTGCAAGAGTCTTCTATATA

TTTCTTTCATATTGATACTAAACTAAGCACAATCTTAACACTACAGTATCTAAACACATG

GACAAATAGGTAAAGAAAAATAAAGGGGGTTTCATCCTTTCTAACCCGTAGCCAAACCAA

AGCTGTTGCGTCGCTGCCCGAAGCCAACACCACTACTGAAAATTCATCATCGCAACCTTC

ACGCCTGCTGCATCATCACCACAGTGCCAAAACTACCTCTGCTTCATCACTGCCTCGCCG

AGCCACCACTACATCTATTATCACAACCACTACTAATAGAATCACTGCCTCAGGTCATCA

TCTCAATCACCATAATAGCAGCCTCCGAGCAATCACAAAATGCCTCGCCAAAAAACATGT

TGAGCGATAACCCCAATCCCTGTCTCACCGTCCATACATCTCCACCACTGTGTCGACTAG

CTGCTACGTCGTCATCATCTCCATAAAGCAGAACAGCCTCCACTATAGCATAAAGAAAAT

CACAGCCCCATCATGCCATTTTCATAACTCAAAGCCAGTGGTCCATTTTCAGGGGAATCA

AAGTTCGAAAAATGAGTAGAGGAGAAAAAGACGAACCCGATGATGTTTGATTTGACCGGA

AAAAGTTGGTGTCATCAGAGTAAGGGTTTGCCCAAATTTAAAACCTACTATATACGTAAA

ATCACCGAGTATTTGGGAAAATTGACCCCATAAACGGAGAGAGGGGGTCACATCGAGTTC

AATGGTGTAGTTGGTCGATCGGAATATGGTGGCCGGAGTTGAGCTGCTGTGGAGGCGGGT

GGAATCCGGCGGATTGTGTGAAATTGGGCAGAGGTTCCTGTAGTGAACGCTTGATACTAC

GACAAATAGGGAAACAAGTTCCTTCAATTAACAATAAGAAGAGCAACTTATA

>XLOC_000624 transcript=TCONS_00001016

GGTTTTCAAACTTTTTGGAATATTTTTCACTTTCTCTAATTATAAAATCTGGGAAAACTA

CACAGTTGACTAATTGCCACCTCTTTTCCCAACTACCAACATGTTTATCTTAATTCTCTC

CTTAATTAACCTTTGTGTGTTGTTAACCTGATATGGTCTTTTTTCGTTTTCTAAGAGCCT

AAAATATTCTTGAATTTTGTGTCAAGAAAAAGGCATTGATTGATAACATTTGATCAAGTC

CAAAATGTTAGTAACTACTAGGATTGAGTTGTCATCAAATGAAACAATGGTGTAATATGA

GGAAGAGGAACAAGTGTTGATGGAGAAAATGCTTGGTGGAGTTGATCAGCTGAGCACTAT

CTCGATTGTTGGCATACCTGGAATTGGTAAGACAACTCTTGCCAGAAAGTTATATAACCA

CGAAAATATAGTCAATCGCATTGATGTTCGTTCATGGTGTTGTGTCTCTGAAATCTATGA

TAAGAAAAACTTATTGCTTGAATTGTTGGGCTATGCATTTGATCAATACCATGTTGATCA

AATTGAGAGGAGTGAAGATGAGTTAACTCTACAAGTACAAAAGTGTCTGGAGAAAAGGAG

ATATTTAATAGTTATAGATGATGTATGTAAGAGCACTTCTATATGGGATGACTTAGTGAA

GTTTTTTCCAGATGACAATAAAAGAAGTAGAATCATTGTAACTACTACGAGGCTTGACGA

TGAATTGCCTGTAAATTTGTTTTGCAATAGTACTATTTATCGTCTTTCTTTCCTGGCTGA

AGATAAAGGTTGGTTGTTATTACAGAAGACGATATTTAAGGAAGATGTTAGCTGTCCAAA

AGAACTTGAGGAAATAGGGAAACAAATAGTAGCAAAAGGTTGTGCAGGACTACCTCTTGC

AATTATGTTGATAGGTGGACTTCTTGCAAGATTGAATATGAAAAAAAGCTATTGGACTAA

AGTTGCTGAATGGTTAATTAGTACTACAAAAGTTGCTGGTGAGGCAGAGTGGTATATGGA

CATAATAGAGTTGAATTACAAGAATTCACCACATTATTTAAAGCCATGCTTTCTTTACTT

TGGTATGTTTCTCAAGGATGAGGAAGTTTCAGTTAAGAAGTTGATACGATTATGGGTTGC

TGAAGGTTTCGTAGAGAGTAATGAGATGAAGATGCCAGAGCATGTCGCGATGGACTATTT

GATTGATCTAGTTACAAGCAATCTTGTCACTGTTACGCAAAAATTTCCCCTTGGTGACAT

AAAAACAGTTCGTCTTCATCGTTTGGTACTAGATTTTTGCTTGGCTAAAGCGAAAAAAGA

AAACTTTCTGGGCACAGTTGATAGCTCTCATGAATTTGATCCTACTACTAATTCAGCTAG

TGATAATATACATCAGTTCCTGATTAGCTCTAAAATGCATCACTTCATGATCAAGTGGCT

TCATCCGACTCAACGTATACATACTTTGAGGTTATATGCCCACAGCAGCAATGAATTTTC

CTTACCAGATGGAGCATTCCATTCAGACTTGTTTAAATCAGTTACAGTAATGGACTTAGA

GAACATTGTGATCGAGGCTTCAGCACTCGAGGACATTATAACATTCTTGATACATTTGAA

GTACTTGGCAATTTTTGGAAATTTTAGTGAAATCCCACCAGCCATATCGAACCTTACAAA

CCTGGAAACCTTAGTTGCTCGACCAAAAAGTGGTACCTTAAGCCTTCCAGGGTCTCTATG

GGATATGATAAACTTAAAGCATGTCGATACAAGTGAAGGGATGATACATTTCAAAGAAAA

TGCTCATTCGGAGAGATTAGAGAAGTTAGAAAGCTTGTCAAAACTAGTTGTAGTAAATGG

AAATGATATAAGTGAATTGTGCAAGAGAGCACCAAACCTGATCCAACTTGAACTTATAAC

AGTGGAACATTGTGACTCATTGTTTAGCTTTCTCAACTCTCTAATATATCTTGAAATACT

AGTTGTCTATCATAGCAGTGACTTACCAATGAGTACTGGAATTAGCAATATGGTGTTCCC

TCAAAGCCTAAAGGAGTTAACTTTGTCTAGTTGTGGCTTCTCGTGGGATGAAATTTCGAC

TGTTGGTAGATTGCCCAACCTGGAGGTACTCAATATACTTGTTTTTGCCTTCAGTGGAAG

AAAGTGGACAGTTGGAGTTGGAGGGTTCCTCAACCTGAGGCTGTTGAAGATAGAGCATAA

TAGTATTAAACATTGGAACATCTCATCCGTCGATACATTTCCCTGTCTCGAGCAATTAGT

CTTGCGTTGGTGTGATCAACTTGAGGAGATCCCTTCGAGTTTTGGTTACATGCCTTCGCT

GCAGAAGATCGAGGTGCACTCTCGTTGCCACCCTGCCATGAAATCTGCTGTGAAAATTAG

GAATATGCAGAGAGATGTTATGGGAAATTCAGACTTCAAAGTCATTAT

>XLOC_010439 transcript=TCONS_00016982

CCTAAAGCCTCTTGGCTTGGGGAAACTCTGCCCATTTTTACACAGACCACCGGAAGTTCT

CCCGCCACAACTGTCCCGCCGGAGAACCACCACGGTTCCGCCGCTCCTCCGCCGCCGGCC

ACTACCTCCGGTGGCCGGAAAAATACAAAAAATACACCATTGGAAAGATCTCAACCCCCC

GTTCCATTTCTCAACTTAGTTTTTACAAAATCCCAACGGTTTGGAGTAGATCGACGGTCA

AAGTTCCGGTCAAACCCAAAGTCCGGCCATTTTTATTTTTTCCGGTGAGTTTTCATTTCT

TTTCTTCTCTTTACTCCTTTCTTTCACCTTCCCATACCCCATTCCCCAAAATTCCCCTCT

CCCCTACCCGTTTCTTGATCCCGTTCTGATTCTTTTTTTGTTTTTTTTGTTTGTTTTGTT

TTGTTTTCTTCTTCCCTTTTCTTCTTCTCTTTTCTTGGTTGTGGTATATGTTTCCAGTGT

GATGATTTTGTTTATGGTGATGGGGCTGTGATACGGACCGGAGTGAGGCAGTGATGATCA

TTTGTTGATGTTTTTGGCCGTGTATTTGGTGTTGTAATCGTCGTGCCGTGGAGGGTTCGT

TTTTGATTTCTGCAATGATTGGCTATGATGAGGGTAATGGTGATAAGGTGCTGAATTTTA

GCTTTGAAAGGGTTTGTGGTGATGGCGTTCGTTTGTTCGTAACAGTGAAGGCGAGGTGAC

GGCGCGAAACTGATTTGATGATGATGTTGTTGAATTAAGGCAGCGGCAGTGATGCTGTAT

TCGCGTTGATGAGACTGCAGCGAGTGAGTTTTTCTTGCTTGGCATTGGTGACGGGTTAGC

TAGACCATGCCCTCTTTCTCTATTTTTTCCTTAACGTTTGATCAGTGTATGGTTTGGCTC

AAGAATGTGAATATTAGTTTTTATGATCAATGAAAATTTTGCTTTTGTTTCTAAAGTATA

AGCTATGTGTATGAATGCTACTCACCAATGAATATTCTATCGTTTATTGAAGC

>XLOC_009683 transcript=TCONS_00015764

TGTACACTTCTCTTTTCCTTTTTTACATGAAAATTGAATTCTCTATTACCTTAAAAGATT

GTATTTTTTTTCTTATGTACCACCTTAGGTTATTTCACACTATAAATACTTCCTTATTTT

GTATCAAAAGGACACACGAAAAAGGCACAAGACAAATTTTCTCCTCTCTTTCACTAGATA

CCTTCATCACCTCTTCTCTCTACTTTTCACACACTGAAAGGTGCTCCGAATACTCATTCC

TATACACGCCGAAAGGCACCTTCTTTTGCGCCCCTTCTTCAGCTACTGCTCATTACCGAA

AAGTACTAGAAACACTACGTCTTCCCTCTCAAGTTCGTCTCCGACATTGCTACTTTGCGG

TCTACAAACCAAGTTTTACTCTCAGTGCATTTTGGCCGAGCATCAGTACCAGAGGAGAGC

TAAAGGCCCAATTACAACTCCTCCCCTCTAACAAACTAGAAGAAAAGGAAGACGAAACAT

TATGCTTAAGTTGAATAATGCTGCTCTTAGTGACGATAATATTGGTACTAGTGGAACACC

ATACAAGCATGTTGCGGTTGGATATGATGAACATGTTGCTACCTTAGAGCAAAGAATTAT

TGAACTAGAAGAGAAACTGACAATGGCCAGTTTACCCATTGTTAACGCCTCCCATTCAGG

AGTGAAACGACCTTAGGTAGTCGTTTTGGGAGTTAGGACTCCAATTGGTGTGCCGACAGT

TTTAAAAACTCCGTAATTCATTAGGACTTGTTTGTAAATGTTGAACTAAATCGGACTTCG

TTTGCAAGGTATCGGAAAAAGGTTTCAGTTTCCATTGTTGGTAAATCTTAAAAGAGGTTT

TCAAGCTCGATTTTGGCAAATGAGCTGCGATTCAAAATCCGAAAGCTTCGTTAGGTTTGT

ATGATTATTTTTTACCTATGTGAACTTTCGAAAGTGAATTCTGAGGCCATTCGGATAGTT

TCCGCTAAAATACCGA

>XLOC_000443 transcript=TCONS_00000753

ACTCCATTTTTCATTATAAATAAAGTTGCATCCATAACAAGTTAAACTAAAAATCCACTA

CAATCAAAATAATATTTTCGCAAAGATCTTAGACAGATCATTTTTTCAAATGAGAAATTA

ACTTTCAACATTATGACTAGTTGAAGATGACGGTCGGACACCTGGAGACATTGGTCTGCT

TTCTTCATCTGAAGCCTGAAAATTATGAATATCTTTACTGTGAGTAACATTCTTAGGATA

TTTCCATACGTCCCAACAACCAGTAAAACAAAAGAAAAATAACACATCTGATATGTCTTA

TTTTGTTCTCTCTTTACCAAAGCTATTTTGGTGTCTTAACCAAATAATATAACACATCTA

ATGCAGAATACCTACAACAAAAAATACACCATGCATCAGAAGAGAAGACATATCTACATA

TCAACCGGAGAATCTATGATAGTACCAATATATTACATAAATTTGGGAGGTAACAAAAAT

ATACAAAAATACAAGCACAAAAGTTTGAAGGACTTGTCCACAGCTCCCTGATTTGTGTAG

CCAATGCTCTGACTTTATAGCATTTAACGGTGACAATTACAAATGCAGTTCAATACAACA

TTTAGTCTATGACTCCAGTCTGAGGGCCTATGAATACATATACGTGTTCAGATTTAGCAG

GTTTTATGTCTAATATGAAGGGTCTACTATCTTGAACACCTTGAAATTAATTACAGACAT

CCATGGAGGAGAACTCTAACAATATAATACACTCGATATTACTCTCCCATCAGTCCAGTT

TCAACCATATCCACATTTTTGCCGTGATCTTTGAAACGAAAAAGCAGTTAAGAAAAAATT

CCAACTGCACTGGATAGATTTACTTCCCCCTTCACAACTCACACACATTCAACTTCAACC

ATTTTTTTTTTTTTTAAGTTTAACTTGGATAGTTCTAGTTAATACAATCAACTAATAATG

CTTGAAGCATCAGTTCAAAGTATAATCACAAAAAAAGAAGTCAACTTTATTTAAGGATAG

ATTGCAAAGGAAACGATCAGTTTAGTTTGGACAGAAGACGTATGTAAGCATAGGAAGATG

AGAGAGCAAGAAAAGTTCGTAACACATACCATTGCATAATTATAGTAGCAAACTCAGGCG

CCATATCTCCTCCTTGTTTTATGAAACCAGTTGATATGAAACCCACCACTGACTCGAGTT

GTCGCCTCTTCAACCTTTCAGCACCCACCACCGACTCAAGCTCTCGCATCTTAATTTTTC

AGCTTCTATCTCCATCTTTATCGCATTAATTTTGTTATCTTGAATCTGAGAAAACAAAGT

TCCACTGCATAGATGAGTTGTATGACCAAATACTTGAGTGTCTGTGTCTGGTTTTTGGCT

TTTCTGTATTCTTTTTCTTCTTTTGTTACTGTGAATTTGTGGCCAAGTCACATTTTTCTA

TTATAGCCAAGTTTTACCTTCTCGTATGGAGATTTTAAGGATGCACATATCAATTGCACC

AGTAATAGATAGAGTAGTAAGCTATAGAACTTTCAAGATGTGTCTAATAACAGTCATTTA

ATCACAAGAACAATAACAAAAATATAGAGCATTGTGCATTATACCTTTTTATGAGCCTTT

CCTTCTTATTTCTAACGACAGGCAAAAAAAGTCAGGACTTTTCATCCATTATTATCCTTT

CTTCTACTAAAATTCAACTTCCAAACAGTTCCCTCTCCACTTCACAAAAGTCAAGATTTT

AGACATAACTCTTCCATGAATTCGACATCTATTCATTAAAGCAAATATTTTCAAGAAAAT

GTAGAGGTAATAGTAGCTTCACTGTATCACACCAATAGACCAAAATACAGCATATTGAAT

CAATGATCAATTATTGAAACTACAAAAACATAAGGAAAAGCTGACCAAAAGATATATATA

TTTTTTCAAGTGAAATGTATGTCCAAACCCAATTTCAATTCCAACTTTGTCTTTGGGCTA

TGGATCGTATCATTTCAAACTCTTTAGTTACAGTAATGACTTTCATGGGATGATGCCTGT

TTGTGGTACATACTCTCTGCTTGCTCATAATAGAACAGTGGATTTCATAGTTTGATATCT

ACTATCCTGTTGCAACCAATGATTTTTATTAACCATATTCATATCACGATGTTTTCTTGC

ATATTAAGCTAAACAAGAAAGAATTGACGAAGAATCAACAACAAAACAGAAAATTTCCAA

GTTTGCTTCTGCTAACACAGTAAATTCATAATCTCAAATTAGTCAACTGCTAATGATAGT

CTAGCTATATGGATATATCAATAAGTAAATTACAGTAGAGAAGCATCCAAGCAAAGTGAC

ATAACTACCTTCTACATGTTCATCTAAATGACGAAGACACGCACACTCTTAAGTACAAGA

ATGATAATTCTTACCTATAAGACTAATACATTGTGACGGTTATTAATTGTCTCACCGGAT

GTAAGAGTTGCATCATCTTCAGACTCATCAAAAAAGCTGCCATTCTCAGGCTAAGAGTGA

GCCCCATTATAAGTTGATTATGCAGAAGTAGCTCTATGATGAGTTGATTGTAAAGGAGTA

CCTTTAGCATGAGTTGAATGAACTGGTGGTATATAAGAAGCTCTGCTATGAGATAACGGT

ATAGAACTAGCTCTCATATGAGATAGTATGCTCTTGAAGCATCTCGGCTGCCTCATTCAT

ACAAGTCTATGTCAAATAAATAAATATAAAGAATAGTAATTTGACATTAGAAAAATGAAA

GAGGATATAATGATTGCTTAATCTACAACATATTTTACTCAAGAAGACTAGGATGCATAG

CTTAGTTTATTCAGGAAGACAAGGATTCATCACTATCCTAAGAAGACTAAAACTAAGTAA

AGATAAATTCATAACAGAACCAATTAAACCATTTCATAACCATGCTTTCATTTTTACCAC

TCCTAATGAAAAGATTTTCTTATGCTTTGACATCTGATGCTTTTCTTGATAGTACTAACC

CACATTAAATTTTTCTTGCTCACTTGAAAAAATAACAATTGACGAGAAAGAATAATGCTT

GATAAAGATAGGAAGTATGTAATTGCACTATATCAGATGGTTGCATGACCGCTAATCTGG

TAGATTATCATCGTATAATTAACATATATAACTAATCCATGTCCAGAGGAAATAGGGAAA

GAAAATTACGAAAAGAAGTAAAATCATAACATAGAAAATAGCAAAACTAGGAATATAATA

AGACACCTGAATCTCTTTCTGCAAAACTTGCTGATACTTCAATGTGAATTTCAGAAACTC

TTTCTCATATTAAAAGGTGTGTTTCACAGTCTTCCGTTTGGTTTCAGATGCTTCCTGACC

CATGAATATAATCAAAAGAGTGAATGAAACAAAACTCTAAAATACAAAAGTACATATTCA

AACAAAAGCAGTCAAATTTACCTGACTGAATAAAGGTCGAAACTGTTTCGACACACAGAG

CACTGCCAATACACATAAACTCAAACAAAAATTTATTACATACAGAAGTCGATACACACA

TGCAGTACTCATAGTTCAAATCTAGGGGTAGGCTTCAGAAATTTGTGTATTCAGTAAAAT

AGACTTAAATACACACTAATTTGAAGGAACAAGTGCAGGAGCTTAAGAAATTATAAAAAG

TGTTTCGATACACGGAGTTCTGCAAATACACATAAATTCAAACACAAATTTATAACATAC

AGAAGTCGATACGCACATGCAGATGCCCATAGTTCAAATCTGGGGTTGAGCTTCAGAAAA

ATTACTAAAAAGGCCTATACACATAGAAAATACAAAAAAAAAAAATACTAAATAAAGCTT

AAATACACATAAGTACAACAAATTCAGAAAAAATTACGGTTAAGCTTAAATACTCACAAA

TTACGGTTTAGAAAACACAAATCGAGGGAAAAAATCAAATAACACGATGCTTTCAGTGAA

GAAATTAGGGTTTACCTTCGGTGAGAATTTGGGAGAAATTGGAGGAAATGAATGTGAAAT

GAGTGAACAGAACTGAATCGAGAATAAATTTGGGGGAGAAGTTTTAATTAATTATAAAAA

AAAGTTGAATGTAAAATGAATTTGGGGGGAGTGATGGCTTTTTCTCATTATTTTTTCTCC

CTTTTC

>XLOC_033428 transcript=TCONS_00054982

GCCGCCTAGGCTGGGCAAACCATGGCGAAATTTCGCCCTTCACCGGAAAACACCATTTTC

GCCACCTTTCGATGGCCGAAAATTATGAAATTATATACCTCCGGAAAGCTCTCGAGCCCC

TTTATCTCGTCCTACCATTGGTTTCCCAAAAAACATAGCCATTTGTCACAGATCGGGGCT

CAAAGTTTCGGCTGAACATTAACTTCAGCGTTTTTTCCTTTTTTTCGGAGTTGATGTTGG

TTTGTTCGGGGACTGATGCCGGTGCTATTTGTCGTGGTAGAGCGACGCGAGGGCTGTGTG

TGAAGATGGTGAGGGGGCTGTTCAACGGCAGTTGGTGTGTTTATTTTTGTTGGTTGTTTC

CGGGGCTGCGTGGTTGTTTGTTTGGAGGGGGCTGTGGGGGTTATGTTGAATGGAGGTGAT

GGACGGGCTGTTCGTGACCTTTTGGGGGGCTGTTTTGTCGAGGCCGTGGTGCGAGGCAAT

GAGGCAGTGATGGAGTTGGCATTGTGAGGCAGTGACGATTAGGTGATGGCGTTGAAATTG

ATTTGATGATGGTGCTGCTGAATGGAGGCCGAAGCAGCGGCTGTTTTGTTCGTTTGTTAT

GATGATTGATGATGGCATGATGAAACGGGCAGCTATGATGGCGAGGCAGTGGAGATGATT

GCGTAGGCAAGTGCGGAAATATGAAGGCAGTGCTGGCTGCGAATCGCTTTGTGAGGCCAG

GCAGTTTGCCTCGACGGGTTAGAAATTTTGGAACTCCAGTCCGCTGTTTTCCTTATCTAT

TTCTTATCGTCTTTGTCGTTTTGGATTTTGTCCTCGGAGATAGAAATCTGTTCAAGTATG

TTAGATTGAAGAATA

>XLOC_021644 transcript=TCONS_00035604

AATGAATGTGAAGGAAATGTGCGGGTGACGGTGGTAAGTGATGACCACTACTTATCACCA

CTCTCCCCCCACCTTGTTTATTACTGCAAAGTTTCTTCGTTTTTAGTTGGGGTTTCATAT

CATTTCTTTCCGAAAGCCACAGCTGAACAACATTTAGTTTCCTTTTCACCTAGACTAACA

CAGATACATAGATTCCACCATGAGCTCTCTTCTTCAAAGTTTTCAGAGCAAGGAAGCTCT

GCCCATGTCTCAACCAGCTGTAGCAGAAGAAGGACTTGGCGTCCGCAAAAGGCTGTCTTC

CCTCTCTCTAAGGATGCACAATATGAGGTCCAAACAACCCATCCTCTCATTCTACTCCAC

CTCCGCCAACACCATCTCTTCCTCCTCATCATGGGCATTTCGCCGATCCAAATCCTTGTC

GTCTTTATCGGCCGAACAGGCAGGCACTTCGATTAGGAAATGGTGGGAGTGGGGATGGGG

CTGGATCATGTCCAAGAAGCCCACGTTCGCCGAGGATCTGGAAATGAACGAAGAGGAGAA

GGCCGTGTTGGGGTGCCACAGCAGGGGTAGCTGGAGGCACGTCTTTTATAAAGTAAGATC

CGAGCTGCGAAGACTCGTCGGCTCCGACAACAACGTTGGTCTACCTCAAACTTTCCGCTA

TGACTCCTCCAATTACTCCAAGAACTTCGATCACGGCAACTAATCCCTGCATCAAGTTGT

TTCTCTCCACAGTAATAGTCAGATCAGCAGCTACACAGGCACATAATGCATGCTAGTTGG

GTGAAGGTGAAACCAATGCCAGCATGAGCCTGCCTGCCTACCTACCTGCCAG

>XLOC_030925 transcript=TCONS_00050874

TCTCAATTACAGGATTACATTGACTCAGATGGTTACAAGCTTTTCCTTTTTGAGAGGTAA

ACATGTGACTGACTTTCATAATGCTCTACTTGACAAACACGAAACAAAGTAAATCACTGG

CTTTTTATTGGAGTTGGATTTTCTTAGTTCATTTGAATTTCATCATCTCTTCATTTCATT

GGCATATAAATATAATTGGGGCTATAATACTTTGATATGCCTTGTGGGTTGTGACTGCTT

TTGTAGTGCAGTGCTTTGATTAATTATGTATTACTTATGACTAATTTTTTCTTTAGTCTT

GTTGCATGTCTGGTGATTTTTGAATCAAACATTTTCTTCTTCATTTTAAACTTTTTATTA

TTTAGTTGACAAAACTCTTTAGCATGTTTTCTGTCTAGGAATGACTAGAGGGACACCAGT

TCCAGTTAACGATCTTATTCTCCATTACTCGATCTTGAGATTATGAAGAGCCTTTTCCTA

CTTGTCATTGGTGATTAAACCATGAATATTATTGTTGTCCCATTACTAGGGCAAAGAGAG

GGGAGGTTTATTAAACAACTTCATGCTTATGGTTCATTTAACTTTATTTTCAGTATGATA

GTTAAATGTCAAGAATGAGTTTCACAATGCAACTCAAACAAGCAGGGGATCTAGTATAAA

AGACTTGATGGATGGCATGGGTCATCTTGGTTGCTTGCTGAAAAGGTAATGGAACATGTT

CAATTGATTGCCAAGATATGACTAGGCAGAGATGTCTAATTCACATTCCTACATTAGCCT

TGCTGCAAGTGCCAACATATATTTGGGAAATGTGAGCTATGCAAGATTAAAATATAGAGT

GGAGTTTGATATAAATATTTTGTCCGGAAGTATCATGGAGAAGAAACAGAGGCTCGACAT

TATTCACCAATTCACAAGTAGTGAATTCAGAGTTCTAGTTAATCTATGGGGTTTATTTAT

AGATAGCGTTTTTAGCTTGTTTTGAAGATGTACTTAAAAAAATTCTGATAGTCG

>XLOC_027159 transcript=TCONS_00044721

AATGAAGAAAAAGCGACACAAAAGCTTAATCAAATCTGTAAAGGGAAGTGCGTTGTGGAG

CCGTTAAATAACAATACAGATCAGATGGATGTTGATCAGGTGAGTTCAGAGTGTACCAAT

TGAGTTTATCGGGGTTTAGTTGTTGGTTGGCCTCAGACTTGAGGTGTGTTTGCTACGGCT

AAGAAGTGTATTTCCGTGTTTACTTCACTTAAATAGTTCAAAATGTTTTAGACCTAGGGG

GAAAACTTAGCTGATCCTTACTTTAAGATAAAGCATCTCAGGCATGCCAAGAATGACCAA

ATTTCCCAAAAACTTCTTCTTTCTCAATCTTGTCCTAAAGGGGGTGTGTATTCCGTGTAT

TGTTGTCCTCTATCAACTGTTCAACTGGTTGAGGATGTACAAAAACTTGATTTCCCTTCA

GGCCCTAGACCACGATATTGTATAATCCGTTGTTGTTATGATGGCTTGGTTATTATCGGG

TGGAGACATGATGAACGCTGCATACTTTTGCTGTGGAACCCCTCCACGGGAGAATCAACA

GTACTTCCTGATCCAGAGTTTCCACCGATTGGACGATCTTCTTGTTTGGGATTAGGTTAT

GACTCAACTAGCGGTGACTATAAGGTCCTTCAAGTTCAGCCCATAGGTCGTGGCCCTAGC

GAAGTTCTCGCGCTGAATAGTGGTTCCTGGAGAAAAATTGATAAACATCCTCGTGGCATT

TGCAATTATTTGAATTTCACACATTCTATGGCATTTGTACACGAGGCATTTCATTGGACC

GGTTGTTTTGGAAATATTTTTATGCCTCCAAGACATCTATGTGTGGCTACATTTAGTATT

TCAAATGAAGTGTACTCGGAGTTGCCCTTGCCAGAGCAAATAATGAGGGGTAACTTCGCT

ATTGGCATTTCGGTATTGGAAGGAATACTTTGTGTTCATTCTGATAGTATTTCTTCGTCG

AATCGATCTTATAAGTTATGGTTATTGAAAGACTATGTGTCATGGAATCTTGGACTGCAT

TATTTACTATAGAAGATCCCTACATCTGTCATGTCGTACCAAAATATAGGTTTGCGGATG

GTGAAGTGCTATTCTTCTATTTTCATGCTAAAGCTGGCGGGACGACATTTAGGACATCCA

GCGGACCATTTGCGTTATGGCCTCATGGTGGCAGCCAGAATTGTATCGCTTTTACAGAAA

GCTTGATTTCTCCAAGATCACTTATTAGTTTCTTATGTATGATCGAGGAACTTTTTTTAT

TCCTTCAGTTATCATGTTACAACCTTGTATTCTTATTAGTTTAGCTATTCCAAAGCTGCC

AGCTTGCTCATTTCATGTTCTGCGAGAAACAATTTAAGCCATTTCTTTGCTAAGAACGTG

GTTAACCTGTCATCTC

>XLOC_027367 transcript=TCONS_00045044

GGTTTCTCTAGCAAAGTTCTGTGCCTCTCTGCCGCTCTCCGATGATCTCTGCCGCTCTCC

GCTAGCTTAGCTACTGGTATTTCACACATCGCATACAAAGGTATTTTCCGACCTCAGCAA

TTTTGAACTTTTCATTGTATTTTCTAAGCTCTGTTTGTTCTTTCCTTTTTATGAACGTTG

ATATTGTTGCTTCTTAAAAAACCCTAGGTAAAAAATCTAGGTTTCAGTTTACTTTAACGA

GTTTATGTTTCCCTAATAGCAAAGCTACCAGTATGCCTTAAGTATTGCGAATTAAAGTTA

TAGATCTGCCGTGTGTTGAACTTTTACTAAAGTTCAATTTAAAACAGCTCTTTGAGGTAA

TACTCTGAAGAAGTAAGCATCTGCTATTACTGCTTATGGGGTATAGTTAGCAAAATAAGG

ATTTGTTGAATCAGCTAAGGGTATGTTCTCCATGGAAACCTCATTATCGATAGAGTAAAG

ATCTGTTTCTGTTTCTGTTTCATTTCTCGAAAATTCTCTTGAAATCAACATGTGAATGTC

CAGCTCTTTCTGTTCTGATGTCATAATTTTCATATCTTTCGAGAGAGACTTAGCTGCGAT

TTCAGTGATATCTCTGTTTACATATTGAAGCTGTTTTATGATAATATTTGGTTCACTTAA

CTGATTCTTCAGTCTATAGTGATCGAGCTCATTTTATGTATTAAATCAAGAAGGCTGACA

CTCCCTTAATTTTGAGGGTTACTATCTAATAGTCTATTAAAGGTGAAAACTCTTTTTGTA

GGTTTATTTCTTTGAGTAGTCATGACGAGTAGACGTATATTGGCACAGCAATATGGATGT

TTTCTGTTTTTTTATTTTCTTTCATTACCAGAACTGATTTGCTTGTTTCTTTCTTTCTTT

TAGTAAGGATGATGTGCTTGTTATTTTAAAAGAATTTCTCTGAAATAGTTGTTCGCATGA

TGAGGTGTGAAGACATCAGATTTTGCCCCTTGGTAATTTGGAAAAAAACCCAGAAGTGGC

AGAACAAGAGCTAATCCATTATGACATTTATAATCTGAAAGAACATATCTAAGGTCCTGA

GCTGCTAAAGTTATTTTGCATATAAATTGACTCTGGAATTCTAAGTCAGACTATTATTTA

CTCACAAGTGTACCTTCTCTATTAATGAGAAGTGGAAAAATCACTTTGTATAAACAATTT

CCTGGATAAACATATCGTGTTATTTCACTATTGTCTGCACCTTCCACTGCGACTTCCTTG

TTGCAACTTCACTATCATTAGCCTAGCGACTGTGTTCCTTGTAGGATCTATACTCTTGGC

TTTTGGATCAGTCCTAATGTTCAGAGTTTGGTGAAAATGATTGGTTTTTCAAAATCTGTT

CTTGTCGACTTCAGATTCAACTGCTTTAATTATTTTGAAACATATATTAGAAGGTGGAGT

ATGATGAGATCTAAAAGCAGTCTGTACATTGTACAAATGCTTCAAGAAAAAGATGCAAGT

GAAAAAGAGTACAATGAAGCACTCAACCTTGAGGGCGTTACAAATACTCTCCTCCACCAA

ATTAATTCATAGAAAATAATTATATCCTCCATGACTAAACAGAATATTAGCTCGATGAAA

ATGCCATTTATCCTCCATTAAAATAAAACTTGTCTACCAATAAACATGTTAGTAAAGGAT

ACAATGTTGGTAATTTTGAATTAAGTGACTAGCAGCACACATGCTAACAGTAAAAACTTA

CCAACGAAGCCATATTTGTATGAAGACCCATATTATGTATTTTGAGGATGGTGATAGACA

TCAAAGTAACAGATAGAAGTTATAGATACTGAAACAGTGACCCACTGTACTTATAACCAC

ATCCACTTGCTTTATAGCCTTCACCAAGCTCTCATGACCAATTGATGCACATAAGTTTTA

GTCATAGCTACACCCCGTACTCATTCAAGTGAGAAAATGATCTAATTGTTGTTTGCTTAA

AAGATAGTATTATGTAAAGATAGAACAACTCATTAGTAAATAAGGTGCTTTCCTCTTTAG

AGAAAGTGGATCCAATATAGCTGGAAAACCAGAATATTTCCTTGAGATACTTACTGAACA

ACCTCCACCGGGTAGAGTTCTAGTTCTGGTTTTAGTATTTTAGGTGGACAAAACCAATGG

AGTATTTAATATTGGAGATTCTAGCCGATGAAGTAAAGCAAGGGAATAAATCAACTACTC

AATTCAAGGCGATCTCATTTAATCGTGTTGCGGAAGCCATTAATCAACGACTTGGAGTGG

AGTGTTCACCTAAGCATGTGGAAAACCATCTTAAAACAATAAAAAATACATGGAATACGG

TGCAAACTCTACTAAACAAAAGTGGTCTTGGATGGAATGATAATTACAAGATGATTACCG

CTAGTCCTAGCGTCTATGCTGCCTATATTCAGGTAAAATGTTCCTTCTTTGGTTGTTCTG

GGCTGTATCCAAGAGAGAGTGTAGAGGTTTAAATGTTCCTTCTTTGGTTGTTTCTGGGTT

GTATCCAAGAGAGAGTGTAGAGGTTTAAATGTTCCTTCTTTGTGTTGTCCCCTACTTATA

CTTCTTAGACCAGAACTGCAGCTTCAACTCTGCTAGTATCACTCATTCATGAATGTACTG

TTGAGTTTAACTGAGCTTCTGCCTTCTGTGCCTTATGTTGGTGGAAAATATGAAGGCTCA

TGGTACTAGTTCATAAGCACAAAAGATGCTGACAGTATTGTATCGCGAACGTTTTGAATT

CAGCACTTCTTGGATGAAATTCTTTTGGATACAAGACTCTTGCAATTAAGTTGTTATATT

CTTTTCTCAATTGATTATGTTCTGCTCTGTTAATCAGACTATAAGACAATATTTTTAATT

GCCTCTGTAGGATATTGCAGAATGATAACTTTAGTAAACAAAATATGACTTCATTTTGGT

TAATAAGATCTGCAGAATTGCATTGATCTTACATTACATTAGAGCCCTATGATAACTGAT

AGGTGGAAGATCCACTGCTGTTATGCAAAAAAAATCTGTTGTTATGCTTGTTTCTAATAT

TATATTTGTCATGTGTTATAGGCCCATCCCGCACACGAGAAATTCATCAACAAGAAGATT

GATATGTTTGAGGAAATGTCTCTCGTTTATGGTAATGATCGAGCTAGGGGTGATTGTGCC

AAGTCTTTTGACGATATTAGCTTGGAATCTTGTTCCGAGAAGTAACAACGATGACATTCA

AGGACCATCAAAGGAAAATGAGGTGGAAGACGTAGTCAGTGAAACTCCTCAAGTCAAGAC

AAGTCGCAAACGAAGTCGTTCTGATATGCAAGACATGTTTGTTGATGTATCATCAAAGCT

CGGAGAAGTAGCAACTGCAATCAGTAGAATAGCTGACACTCGATTGGATGTGAAGAAGTT

GCATGAAGAAGTTATGTCAACCGAAGGTTATGAGGAAGCTTTCTTATCTGAAGCTGTTGA

TTTTTTTGTGGAAAGAGACCCGTTAGCTAAGGCATTCATGGTAAAAAGTCAGGCTCTCCG

CAAGTTGTGGTTGGATAAATTCAAGCGAGAACACTATGATACACAATAGATTTGCAGAAT

AGCTAGATAGAGTTTGTTGCTTGTCTAAGTAGTTTGTCATGTGTAGTTATTTTTGTGTTG

AAATGAACAATTTATTGCAGATTCCCTTTATTATACTGTAATATTTAGCAAGTTGGATAA

CTTTTGGTTTCTCACTCGATGTTATGCACTTACTGTGTACTTATTAGTTTTAGTACAAGT

ATTAGAAAAATAATTTTCTTTGTTTATATAGTTATAGTAGGG

>XLOC_020419 transcript=TCONS_00033509

GGAACTTTAATGTGAATACATTGTTCAAATCGAAACACAGAGAGCATGAGACAGATCTTA

CCAACAGTAAACTTTTTAGCTTTCTTGAATAAACCATTACTTCTCTTTGAGTTAGTCACT

TGCCTGTTTGTTGAGTTCTCTATTTTCTTGATCTTAATTTTTCCACAACCATTTTTTTGG

GACTAGGTAGGGATCTCTTGATCTTATTTTACTACCAAACCCTGCTGAAAGTAATGGAAC

TGATGATGAATATGAAACAGAAAGCTGCCTCTAATGTTCAGTGATGAAGTTAAAGAAGAA

ATAATAAGAAAAAAAAAAAGAGTTAGTAGGAGAACTATATCAGGAAAAAGAACTCTAAAA

GCAAGAACTAAAATAGAGAAAAGCTAAAGTCCATTTTTCTGAAAAAACGTATAAAATTCG

TGATTGGTAGTGTTGGTGTGTATTATCATTAGTGGCCATCTTTGAATTAGCATTACAAGT

GAGTTTCACATGCCTTTTCTGTTAACTTTCTTAAACTATTAGCTGAACCATGCCTTTTCT

GTGGTCTGTTAACTTTCTTATCTGAGTCCTGCACATAAGTTATATTTCTTGTTAACTATT

AGCTAAACCATGGCTCCAAACTCTTGTCAATTATCTTTCGTTGTTAATTATTAACTGCAC

AAGTTTTTTATTCATATAACTTCATACTATTAAACTTTTCCCATTGCAGGGTCATTGTTA

TTTTCCCTTCTAATTACTTTCTTTATTTGCTCTTGCTTTCCCTTTTGCTTCACTCTTGGA

GGGGCTGCGCCTCATTTTCCTGCATTCCAAGCACCACTCATTTCAGAAAAATACGAGTGA

TATAAATGAATTGTGGCCGC

>XLOC_015047 transcript=TCONS_00024609

TTATATGTTATATTCTTTTTCTTCGACTGCCTCCACCTCCTATGACCCCCTCCCCTTCTT

TAATTTTTGCCATCACCACCCACCATTAGCACCTATTATACTAAATCATCTAGCACCACC

ATCAAACTAACAATACCTCATCTTTCTTTAGATTCAGTCACCATCATCAACACTTTCTCC

TTTTCTTTCTATTTTCGTTGACCGCCACCAATATCACAACTAACTCCTTCTTTAGATCTC

ACTGCTATATATTATCATCCAAATCGCATTTTATATGCTTCAATATTCTCTTTCCCCTCC

CATTCTACTTCACATCTGACCAACAGTACTGCAACCTACTCATCCTTCTTCTTTAGATCT

GGCCATGACAACCACAATCTCTTCCTCATTCTTCAGATTTGGCTGTTATCTCCACCACTA

CAACTTTATCCTACTTCTCGATCAATTTTTCTATACTAGCACAAACACCACTCCTCATCT

TTAGATCTATATGGCGACCTTATCATTCTTCTTCTTTAGATCTGCCACCACCACCACCAC

CTATTTATCTTTTTTATATCTAACCATCACCACCACCTCCTCATTTAGAGTTTTATGTTT

TTCTAATTTTCTTACCATTAATAGAATAAGTCGACCAATGATCACCTTGAATTCGCTGCA

GGTAGGGATGGCTACAAAGCATGTAGTCGTAAGTAGGTTCAAAAGAAAGAGAGAGCATTT

AGAAGAGATACGAAAAAGGAAAACCATTGTTGAGTAGCTCGTGAGCTTC

>XLOC_023205 transcript=TCONS_00038155

AGAGCTGGAGATGGCAATGTTGGAGGAGTACATAACAATTCAAGTGACACCATCCTTCAA

GACTAATGCACCGTTGATGCGCACCTAGGCTTCAACAAGCATATCTAGAGTCAGGACAAT

CGAGCCACACTCGCTCAATCAAAGTACCCTCGCTCAATCAAAGTACTCTCGCTATGGGCC

TTTTGTTGAATCACAATCATCGAGGATTCTTGTCGAAAGTGTGCATCCAACTTCAGAGAA

TGAAAATTGTGATGCAGGTCATTGAGTCGCAAAGGAGTTTTGGCCACTGAGTTGCGAAGA

AAGCTAGAAAATGATTGTGCTGAGTTACAAAGGAAGCTAGAAGAAGGTCGTGTTGAGATA

CCACATATTCTTTCTGGTACTCTAAGACATGGCGCCTTTGTAGTTGTTTGCTATAAGTTT

CTTTCTAGTACATTGCTATAAGTTTCTTTCTGTTGTCATTTTTATGTGACTTTTTTAGTT

ATCCTTTTTGAGCAAAAACATTGTCCTAATTTCAAGCATCTGCTTGTATCCAGATTCTAT

TACCATGCTACTCGGTTGTTACTTTACTCTTTGTTGATCCTAACATAATAAAAACAATGT

CTTCCTCCATCTACGCACTGTTGCTGGCTTAAAGTGTTGTTAAAACTAACTTAATCCTCT

CAGCATCATGCTTAATTATTCAATTCAAAGGCCACCTCTGACCACTGTTGTTTATAGGCA

TAATCATTTGTTATCAGTACAGATTGTGTAGCCACAACTAATTATCATTCTGATGCTTCC

TATGTTCCCAATGAATAATCGAAACTTTATGAATCCTGAAGTTTATGACAGTTATGTTTG

TATAAGTATTCAAATATTTAGACCAAGTACTTTTTTCTCACCTGATGCATGTATTCTTTG

GGATCAATCTTCTTGGTGATAGTTGATTCATGTTTCAGGAAATGAATGTTCAATAAGATA

GAGTAGTCTTGGCTTCTAGTTATACTTGTAGTAATTCCTTCAGCAATTTACTAAGGATAT

GTATTATATTGTACACGCAAATACTAATGGTGTTCATCTTTGTTTTGTTAATACATAGCA

GGGATGAAGAATTTCAACTACAAGAAAATGAAGTTGCATTGGAGTAGTTTGAAGCTTTTG

GAATGT

>XLOC_034176 transcript=TCONS_00056223

TAAAAAATTCTCCCAAGTCACAACCCATCTCTACAAGGATTTGCTTCAAAAGGCACCATT

GAGGGTTTTGTTTTGCACAATAAGTTCTTTTTTCGGCTTCAAATATTTGATTTTCTGGTC

AATTTCGTTTGCGAATTTGTTGCTTATTTTTCTAGGTTTTCTTGTTTCAATTAGAGATTA

ATGTTGTGGCTATCTTCTTTTAGGCTCTCTGTTAGCAGTGATGTACACCGTTGGACTCAA

AGTGCCACTGGGAGCTTTTGAATTCATTTTTGGTAGATTATATAGTATATTTTTATAATG

AGTGTAATAATAGATGAATCATTTGCTTGCGTTCTTTTTCATTTGAGTGTAATCATAGAC

TACTCATATGTCATCTATTAATATTTTTATCAATCTTATTTGGGAGTTCTAACTTGTTTC

GGATATTTACTTTGGATTTTTGAGATGTTTGATATTCAGGTTGACTAATTCTTTTCACTC

TTTATTGCTTGTGGCCTGTATTTGGTCTAGGAGGTCTAGGAAAACCTATTCCTGCATAAC

TATGTGTTTTTAGTAGAAGAAAATTAAAACTTACAGATGATTATGAAAAGAAATTGAAAT

TGCGTTCATCCATTCACTTTCTTATAAGCATCTTGCGATTCAATAAATGATTAGTGCACT

ATTATAAATTCATAGTTCTGCATTTTGATTGTCTTGGTTATAATAATATCTTTTCATAGA

GCTAATTTGAATTTAAAGTAGTAAATGATTGTTACTGGTTTCATATAGCAGTAAAGCCTA

CGTGTGTGATTTTTTATATGAAAATATATAGTAGAAAAAAGGTGTCTTTGGACTGATAAT

AGGTACTAATGAATAAAGAACTGAAACAAGCTACTTATGTTTTCATATGATCTTTCTTTT

GGACTTTAATTTCTTCAAAAGCTAAAACCATAAGGTTGTTGATAGAAGATGGCAAGAGCA

ATGTTAGCATCATTGAGTAAGCCTTCTTGAGCAGTCTGTTTGTTCTCTTTGCTAAGGACA

CCTTACTTGTTTGATTATTAATCTCTTTAGTTTTCCCTTCTTCTCATCATAGCTTTATAT

TTTTTTTGTTTTTTACCGAAAGATCTTTTAACTGAGTTTTATCGTCTCAATAAATTCGTC

TTGTTTCAACTGATAGTCAATTTTTTTGGCTCAAACCTTTGTATATTCTTTTTCTACTTA

TTGTCATCTGAGAAATGATGTTTTCAGCTTAATTATCCAAGATTTTGCTTGGTGTATCCC

TACTTTGCTTTTTCAGAAGCTAGGAATTTTTCTCTCAGCCCTATGTCTATCTCCGATGGG

ATTAAGTATCTTTTGTTGCAAAACAACCCTGGAGTAAATGTTCAAGATGTATTATGATGG

ATCGAGGCAACCTTCGCCAGTAAATGCAAATAGTTCACGGATGCACAAGCTGTGAGAGGA

CAAAATCTTTTGCTGGTTCTACTCATGGTTCGATTCTTGAAAGGTAACTTTTTTTATTAC

GAATGGGAAGACTCTGCGATGAATCAGCTCCTCGACTAGCTACTTCATTTGCTTCACTGG

TTTTAACAAATCACAGGTCTCTTGCATTTTGTTTCACATGGAATGAGCTCCCAAGCTCCA

AAATAAAAGATGCATTGGATTTCATAAACTCCCACTGCACTGCCAAAAGCAGACAACTTG

GAGGTTCTTATCTATTGTTGATGAAAATCTACTGATTAAAGTAGAGCCTAAATAATGTCA

ATGGAAATTAAATTAGTGAATAAGAAACAATTATCTTCTTTTAGTGGATAGAAGACGATT

TTATTTTTTAAGCTTCATTACTAGTATGCATCTAAAT

>XLOC_005302 transcript=TCONS_00008637

AAAAAAATTTGAAAAGTTATATTAAAATTTTCCTCAAACATATTCTCCTAAATTTCAAGG

ATTAAATTCACATAGGTGCGAAATATATTTTCAGTGTCACTTTTCCCTCCTCTTCCTTTA

GGTTTAGTTCATGAAAAATAAGCTTCACCCTGTTTTCACAAGATCCACCGTAGGTTTTGA

AAAAATTTCAGGTCTTCTTTCCCTTTGATTTAGTTCATTATCTTGTGATTGCTCACAACT

CTACTGTGATAGTCCATCTTCTTCACTGCCGAATGAATCTTGGCAGCTCAAACACTAATA

TTTGAGCCTTAGAGTGAAAAGGTCCAATTGGAACAATGATAAAAATTGAGAATTGGTACA

GCTTTGCTTAGAATGATGCTCAATATATATGTCCAAAGAGAAAAACCTTCCACATTGGGG

ATGAAGACTTTTGGACAGTTATAATGTTTATTAGATACTAAATACATTTTCTTGTAACCT

TGAAATGATTTGTTTTATTAGTTGATGTAGTACTAAAAATATGTTATTATTTGAAGTTTA

TTGCAAAAAATTTCTTTTGAATTATTTTTTATTGTATGTTTAATTAATTTCATAATAATA

TTTCTGACG

>XLOC_000533 transcript=TCONS_00000891

AGAAATAAGAAGGAAAGGCTCATAAAAAGGTATAATGCACAATGCTCTATATTTTTGTTA

TTGTTCTTGTGATTAAATGACTGTTATTAGACACATCTTGAAAGTTCTATAGCTTACTAC

TCTATCTATTACTGGTGCAATTGATATGTGCATCCTTAAAATCTCCATACGAGAAGGTAA

AACTTGGCTATAATAGAAAAATGTGACTTGGCCACAAATTCACAGTAACAAAAGAAGAAA

AAGAATACAGAAAAGCCAAAAACCAGACACAGACACTCAAGTATTTGGTCATACAACTCA

TCTATGCAGTGGAACTTTGTTTTCTCAGATTCAAGATAACAAAATTAATGCGATAAAGAT

GGAGATAGAAGCTGAAAAATTAAGATGCGAGAGCTTGAGTCGGTGGTGGGTGCTGAAAGG

TTGAAGAGGCGACAACTCGAGTCAGTGGTGGGTTTCATATCAACTGGTTTCATAAAACAA

GGAGGAGATATGGCGCCTGAGTTTGCTACTATAATTATGCAATGGCTTCAGATGAAGAAA

GCAGACCAATGTCTCCAGGTGTCCGACCGTCATCTTCAACTAGTCATAATGTTGAAAGTT

AATTTCTCATTTGAAAAAATGATCTGTCTAAGATCTTTGCGAAAATATTATTTTGATTGT

AGTGGATTTTTAGTTTAACTTGTTATGGATGCAACTTTATTTATAATGAAAAATGGAGT

>XLOC_025051 transcript=TCONS_00041224

ATATACCATAACGAGTTAAACAATTTCACCTAACTTTAGCCACAATTTGCTGGAACCTGA

CATAAGAAGATTCTTGACAGCAAAGCTTTGAACCCCAATAGGATTGCAATTAAATTCCTT

TCTTACAATTGGTCCTTTATATCCTCTTTTCCCATATATCACATTTCCCATAAAAAGAAT

TAAAATATTCTCCCTATAGCACTGCTCCATCCATTGAATTCTTGCAACCCCCTTTAGTTC

CTTACATCATTAATCCCTGGTACCAAACATCGGATAGGAAAAAAGATTTGATCAATTATC

TTTATTGTGTCTGGATGCTTATTCAAACTTTATTGTGTTTGGTTGGTAAAGATTATAATT

ACCTTTTTTTGTGTGTTTTGAATGCTAAAATTCAAGATTTGCTGTATTTGGTTGCCAAAA

TTCAAACTATATTGTATTTGGTCAAAAAAGAATAAACATTCTAACCTTTTTGTGTTAAAG

GTTGATAATGATTGTAACTTTATTATATTTGGTTGCTAAAAGTTCTACTAACTCTATTGT

GTTCTTAAAAAACGACATGGGAGAAGAAGATAATGAGAGGCAGGAAACAAGCAGCGGCGG

TGGCAAGAAGTTGACGGCGGTGGTATCAGCATTCGAAAAGGCAAAGTCTGTGGCTGGCGC

GGGGGTTGACAAGGCAAAGTCAGCAGCAAAGAAGTTGAAAAGTAAGACTTATTTGGGGGT

TAATTGGGTTAAGGACAAGTGCCATAAAGGAAACTCTACAAATTAGTGAAATCAAGGATT

TTGTTTAGTTTCTGCTCATTTGAATTATATAGTACAGATGATGAGAGATTGTTTTGATGA

AATGATGTTTGTCCCTCTTCGCGAATGTTTGTATTCCATCCGTTTTAATTTACGTTATAA

CCAATTATTATTTGAAGAGCCATACAAGATTTTATTCAACTAAATGTGTACTGTTATTTC

AAATTAAATATTTTATATCTAAATTTACA

>XLOC_007074 transcript=TCONS_00011498

GTAATATTTAAAAACCAATTACCAAGTACTAATGATACTTTAGTGTGAGCTCTTTCTCCT

TACGCAGACAGGGATCAAAGATGGACTTTTGCATCCAAGTAACAGTTACTTTAAATCTCA

AGCAGTGACGTTGATGCAATGCCAAATGGATTGCACAGCTGCAGGTTAAGCATGAATGTG

TTAAATTTCGACCTTTCAAAGGTGAGATTTTGGGACCAATAGTTGCCACTCACAAGATGA

AATTTTTCTCTAACGCAATGCCTGT

>XLOC_018893 transcript=TCONS_00030982

ATATCTTCTTGTCCAATTTTTCAAGATACTCTTCGGACCTCCCAATTTTGATTCTCACCT

TCGCTTCTCTCTCTTCATTCTCTAATTCTTTTTATTCCGTGTATTTTCCTCATTACTTCT

ACGGTTAATTTCTCAGTTTTCAGAAAAAATCTCTATTTATTTGTTTCAGCATTTGCTTTT

GGAAACGGTGTCTCATTCTTGTAGTGTTTGCAGAATTACAGCGTTCAGGTTAGTGTGATT

TCTATTATACTCCTTATGTTTCTTCTATTTTGCCTTTTGTTTTCTTTTTTTAGTTGCTCT

TCCGTTGTGCTTCCATTAGCTTTCTTTAGTTTTTCTTTTGTATTTCCGTGCATTTCTTGT

ACAATAGATTTGTATTGGCTTTTGTGTTTACCTTGATGTTTTTTGCTGAACTTTGTACAG

GAATAACAATATTTTGTTCTCATTGGCAAGTTTACCATTGAGTCTATTTTTCCAATTTGT

GATTGTGATAAATGAAGCTAACTTCCTATGATGCTTTCCTTGATAAACCGAAGGAGTCCG

GATTCTATTTAGTTTGTTTTCCTAAATGAAAATGAGAGCGTCTCTCTTTATTGAGCAACA

AAAAAATCTTGCATTTTTCTTTACAGTTTCGTGAACAAAGTTATAAAAACATTGTTGTAT

ACATCTTGTAAGGTCAAATTTTCTTCGGGCATATTCTGGAACCGTTTTACAATAATTATG

CTAAGGCGCATATAGAGTTGTGCAGAAACGCTGGTGATGCTATCTTCAGGAATGCTGGTG

ATGCTTTTATTCTTTAAGTCACTTGTAGGCTTTGCAACTACTCATTGATCATAGCACTTG

CTTTCTCTTAACAAAGAAATTCCTAAAATTCAGGGGACTTCACTGCTGAAGGATCATTTT

CAGTGAAACAAATATTCATTTTCCTTGATTAAGTGGATAAGGCCATAAGGGGTGTTGAAA

AATAAGGATACATCAATGCTTGCTTGACGTGGTGTTGAGGTGGATCCACTGGCTAATGAT

ATTCTAAATAAAGCTTATGATCGACCAATTTCCTTCGAAGAGACTCATAAGACTGTAATT

GTTTCAAGACTCAATTTTTTGAAATCAAAGTATGTCACAACGATTCAGTCAGGCAGGGTG

AAAGATCAAACTCCTCAAACTTGGAGAAAAAAGAACAATAAGAAATTACTATCAGTTGAG

CTTAGGTATTTGATATGTGATACATCAGCTATTCAATTGTGCAAGCTGATAATTAAATGA

GTATTCATTTGTTGGTAAAACAACAAATGATGACAGCTGAATGGAAGTTGCTCTCTAGAA

TTTGAGAATGACCTTTGTATGTTCCATCCTTTGTTGATGAATATATATTGGGATTAGTCA

TTTGTCCCCCTTTGGCTACTTCCTACTGATTCTTTGATCATCCCAGTTGATCTAAATGAG

TTCTCCAAAGCAATATTAAAGAATGGCAGTCG

>XLOC_024365 transcript=TCONS_00040070

AAAATTTAATTAAAATAGAGGGAGGTAAAATGTAATTAGATTAGTGAAACAAAAGCCTAA

CTCACCTATAAATAGGTAATAATCTCATACTAGGGTTAGGACATAGTTAGGAAGCTGCTG

AGTTCACCTTCAGCCGCCGTCACCGGCGCTATGCTGGGGGCGGTGGCATTATAGCCACCC

CTCTTAGTGATACTCACCATTCATGTTAGTCTTTCATATATAGCCTTTCTTGCACTTTTA

GCCTTTATTTCACTTTTAGCCCCTCATTTCTTGATTATTTTCACAACTAATTGAGATTTT

GATTGATACCCAAATGAGGAAACATAGCAAAGATTTTTTCTTGATCAGACGTAACAGGAG

CTGTTTTCTAAGCTTGATCAGATGGGAAATGCAGAAGTTGGGTGAATTTTGTATGGAGGG

TTTTCATTGTCTAGCAAGGGAATGGACTTCGTTGCCACATATCAACACATGCTAAACATG

AGTTTTCTTAAGCTATTTGCGCTTAGATCATGGAGTATTATACTCATATGGACCTGATGG

TAGATAAATTGTGGACACTAGTTGCAGTTACTATTATGTAATCCATGCAAGTTCTATGAT

ATTGCAGACGCAAAATACATTGATTTGTATGACCACAGCATGAAAAGGAATGATGCTCTC

ATCTCACAACAACCAGTCTTAAGATGATATAGTGGTTTGGAAGGCGAGGCATAAAAAGCC

TATGAAGCAATTTAGTTGAATGTGCAAATCGATTGGCCGCTTGGACTTGGTAAGGCAACT

CCAACCAAGAAAATATAAAATTACAAGTACATAACTTTTCACTTGTTTGAGCATGGTGTT

TATTTCTCAAGTATACGAGAATAGAAATTTGTCAATTGAAAGTTTTGATCAATGCGCTCT

CACAATGTTGAAGCTTAAAACGAGCAATAAGGCTATAACTTTCTTATTTCATTGAAAAAA

GTAAGGAGTTAAAATTGCAAGCTTGTGTTATGCTTCAGGTTATTGGAGTGAATAGCTTGA

CTGAAATTTTCCTTTTGTAAGTTTCTTTTCTCGTATACTTGTGAAATAGAACACCATGCT

CAAATATTAGAGTGGATTGTTATGCTTTTGTGATTATGTATTTTCTTGGCTAGAGTTGTC

TTGCCAAAGTCAAGTATCCAATTGATTTGAACCTTCGGGTGAGTTGCTTCATGATCTTCT

TATGCCTTAGCTTCAGAACTTACCACTATATCATCAATTCTCGGAGAGTGTGACCTGCCT

GAATGATCTGGCTATACAATTCAAACATTTTTTCATTGGAGACCTGATGTTGAATTTGGG

TGTTGAATGACTTCTACTTGAAATTGAAGAAGAGGAAACACTTGTTATTGTAATTCTGCA

TTGTTGTAGCTCTACTTTTGTCCAGTGGCTGACAAGAAGGAAATGTTGCTTTTACATCTC

TCAGCGATGGCATCTGTTCGGCTGTAGTTATCTGTGTACCTTGATTTGTACTCTGATTTG

CAAAATGTAAACTGCTTCAGGTTGTAGGGCGTATGATAGGATAAGTTGTCGGAATCTGAT

TAGCATGACCTAGGTCGCGAACACTTGTATTCCAGTTACAGTCGTGTGAATTTGGATATT

ATATATCTTTGGCTTTGAAATTTGATCAAGTGCAA

>XLOC_029055 transcript=TCONS_00047746

GCTTAAACAAAATACGGAAAACCTCAAGAAAATTTCCTGGTGCTTTGAACTACTAGTAAT

ACTTTTGAAGTCCTAAAATTTTGACAGTTTGAAAATTTAGTTCAAAGTCTCAGAGAAATT

TTCTCATTTTTTCTATATTTCTTAAATATGGTTATTAAAAATTTTCAAATAATGACTGTT

TCTCCATCAGCACGCTACATACTGGCTATGAGGCCCAATGTTTACTGTTTAAGAAATCGA

ACCCGACATAAGAGTGAATGGCAGTTGAGAAGGTGTAAAGGAATTTGTGGGTTAGTGAAT

GGAGAGCAGGAAGTCGATAGCGAAGCAACTGTTGAAGGACAAGAAATTCTGGGTGGCCTC

AGCCCTCATAGTGTGGGCTGCGGCTCTGCAGGGGCACATGATGTGGTTGCAGAGGCAAGA

TTCTTTCAAGCACAAGTTCGGAAACTTGAACGATCAACCCACTAATCAACACGAACTAGC

CGATACCAAATAATTGTGGTTACTTGAAATGTTGGAGAACCAATGCACCACATCTTTAGG

GAAGCAAATACTGTGGCACATCTTTTGGCCAAAGAGAGTTCCAAAGAAGCTAATGGTTGT

AGGACTTGTCCTCTATCTGCTCCTCCTCTAACTGTGGTGGCAGCACTTTCTGCGGATATG

AAAGAAGCTAAACTATGTTAGAACTGTGTCTGAAAGTGTGTAATTAATTAGCTAGCTTGG

AAACCCTTGCACTCTGGCAGTTTCTTCTAGCTTTTGCAGTAGCTCTATGAACTCCTTTCC

TTATGGCAGTGATGGTCCACATGGGACACCTATGCCTTGTAGTGTTTTAATAAAATGAGT

CCC

>XLOC_012938 transcript=TCONS_00021184

CCGGCCAATGGCCAAATATCAAGATGTTACTCCAATGGAACCTACTAAGGAAAAGGCAAC

AGAGAGAGTAAACATGGATGATAGGTGTGAAAGTGTGGAAGAAGAAGAAGAATGCTTGAT

GAGAAGAACGCTTGAAGCTCACCTTGATTATATCTACACCCAAAAGCACAACCAGCCTTA

ATTCATTTCTTTATTGTAAAAGTATTTTAATTCTACATTCTTATGTAGCTAGGTATTGTT

TCTACTTTTTTATTTTTACTTCATG

>XLOC_016624 transcript=TCONS_00027245

GCTACAAGATCGTGAATTTCCTCTTCGAGATACAGTGGTCATTGATATTGGGCTTCTCGT

TTACTGGTTATATGAAGGTGAGGAGGAGAACGAAGAAATGAAGCTCGGAGAAATGAACAC

AACACTAGGCAATATGAGCTCAGTGATCTACCTCATCACTCGTAAGGCGTTTCAATCTAA

TTTGCCGAGGATTTGTGGAATAGGCTATGTCGATTTCCTTTTAAACAATCTAAAGGAGTT

C

>XLOC_024565 transcript=TCONS_00040405

GAAAGATATGTACAGACAAAAGATCTTTCCTTTTCCCACATCTACCAAGAGTCTACCCTT

TTAGATATACCCTTTTAAATATATCTTTCAAATCCCCCAAAAGGAATTCTTTCATAATAT

CCTATCATCTTTTTTTTTCTCAAACCAAAAAAGTTATCATGTTAGTTCTGTCTAAGCACA

ATTAAAAATATAACTAACAGATAAAAAGGAGTTTTTGTTATCTTCCCTGTTCGTCTTCAT

CACTCTTCAGGTAATCAAACTCCTTAATTGTTTTATTAATTCCTTCAACATTTTCAAAGT

GGAACCCCTACAAAACTGGCGAAGGAGCAGTAGATCTAGTAACAAGCAAGTTATCAAAAG

CGTCAAACACCATACCTAGATCACCAAACGTGTACTGACCAATACCACGAGAAGGTTCGT

CAATCTCAAAATTATTCATAGGCTGCCCAAAAGGTACTTTTTCACCTCCCTTAGCAATAG

TTCCCAGTCCACCTGTTTCAAAGTGTCTAATTGTTCCATCTAGCTCCTCGTCTAGTGGCA

TATGCGGTGGTGGCTGGTGAGAATTTGACTCTGCACACGGAAAAAAGGTAGGAACACATC

TTCTTATGACTCCATCTGCACAGGATTTAAACAAATAAGGATTTTCCCAAAAGTAATGCT

TTACCTCTGACATGAGCTTTCTTTTCTGTTGATATGAATATTCCTTCGGTATCCATCATC

CCACCAAATAATTTGCCAAATCGTCAAACCATGGTGGTTAATCAATGATTGAATTCAAAA

AGAATGTGCTCATCCGGGAATTCTTCAGAGATTTCAACCAATTCTTTTGGAGGGTTCTCT

AGTCGAGACAGGTGATCAGCAACTTGATTTTCCAAACCTTTTTTTTTCTATAATTTTCAA

ATCAAATTCTAGCAGTAGGAGTATCCACTTCAAGAGTCGTGGCTTGGCATCCTTTTTCGC

CAGAAGGTATTTTAATGTTGAATGGTCTGTGTATACAAGTTACCTTCGATCCTATTAGGT

AAGCCCTGAACTTGTCAAAAGCGAATACAACTGCCAAAAATTCTTTCTCAGTAGTAGCAT

ATTTTACCTGTGCATCATTGAGCGTTCGACTAGCATAATAAATTGCACGAAAAATTTATC

TTCTCGTTGTCTCAGAACTGCACCAACTGCAACATCACTAGCATCACACATTAATTCAAA

TGGTAGGTTCTAATATGGGGTAATAATAGTAGGTAGTTATCAATTGTTTTTTGACTTCTT

CAAAAGTCTTCCATGCAGTCATTAGTAAATTCACATTTCACATCTTATCCCAACAAATTT

GTCAAAGGTTTTGTGATTTTTGAAAAATCTTTGATAAACCTGTGATAGAATCCTACATGT

CATAAAAAACTTCTGATACCTTTGGCAGTTGTTGGAGGTGGTAGTCTTGCAATAACATCC

ACTTTGGCTCTATGTACCTCAATGCCATTTGATGTTACCTTGTGTCCCAGAACAATGCCT

TCTTTCACCATAAAATAACATTTTTC

>XLOC_033600 transcript=TCONS_00055269

CTACAAAGTTTCAGGATGCTTGGACTTCAATTGGATTGAATTTGAGAAGTTGGACTGACA

CATCAGTGTACAAAACTGCTTCAGATCACTCTCTGTATTCAATTTGGCAACCCGCCTCAA

AAAAAGGGCGCTCCGCCCATATTTTGGTTCAGCATACTTCTCTTATTTTGGTTAATAAGT

CACACACGCTCCCTCACAAGGCTCAAACCACAACCATCCGCACACCATCACCTCCGATCA

TAGCAGCCATCAGCTCCGATCATTGTCCACCCCACACCGGCGACTCAGGTAAGCTACGTC

TCTTATCTTTTCTCTCTCCGCTGTTCTTCATTTTGTTCATATCAATTTCCACCCGTCCCC

AATTCACAATTCCTTCACCGTCCCCGCCACTTTCCGCCACTGAGCAACCACCATCGATCA

CAACTGCTGAGACACCATCGGCGACCACAGGAAGTCAGGAGTCTGAGATCTGAGAATATG

TTGGTTCTGCTAAATTGATAATGCTGCTGATTTTGCAATTAAATATCATTCCATCTTGTG

CATCTGAATTCAAGAAGAGCTTGACAGGTCATAAAAAATATAGGGGATTTGATGAAGAAA

GGATTCAATTAAAGATTAAGGTTTGGAACTTTGAAGTTTCGCAAGCAAAAGGGGGAAGTT

ATGATAAGGGGCAAATCGAGCCAAGACCCCAACCATAAGGAGCTAAGCTTGGAGGATGCG

AGGATGCAAGGGCAAGAGCTTAGGAGATGATTTGAAGGCCATACATTTGAGCTCAATTAA

AGGAAGGAAACCTACGCATCAGGGCCAGGCGGAGCGCCTAGAAGCCAGGCGGAGCACCCA

GCTTCAAGGAAACCAGGCGGAGCGCCCACATCCAGTTAGAGTGCCCAAGCCTGGGGCCTC

TTTTGATCAGAAAATCATCCAGGCGGAGCGCCCACATCCAGGCAGAGCGCCTAGAACGCT

GAAGGTTTCTACTTTTGGCTTGAACTTCCTATTTTACCCTTAAGTGACTTGTAAGTCTTA

ACCCAACCCATGGGCATTTTTAGTCATTTCACATTGCCTTAGGAAAGAATATAAATAGGA

ACACTTAGAATTTCATTTCATTAGCCAGACATAGAGAAGAATTGGAAGAGAGGCTTTGCC

TTCTTTGTTGAAGCCCGAGGCTTCATGTATCTCGTTAACTTGGAATCTATGGGATCGTCC

TAGAATGATCCTTAAATAGTTCGAGTCTTTTTTCCCCCTTGAATCAACTAACGTTCGTG

>XLOC_008133 transcript=TCONS_00013260

GCTGAACTCAAGTTCATCAATGTAGCTTTGGTGAGTGAGAAAAGCGCAGAGGATGTGACA

CTGAGGTTTCCGAATGTGAAGTCTGTAAAAAAATACTTCAACAGTGCTTGCACTGTAGAT

AAGTATCCAGATTTTGATGAGGAATTTCTTATGGGAACGGCTCTGGCCAGTAGAGTGCTT

CGCCGTCAAGTTCCCTTTGATGATGTTTTGCAGATGAAGTCTCTTTGGTCCTTCTGGTTT

GTCAATTGGTTTATTAGATGGGGCATAGACCGACCATTGAATTCCGTAGGGGAACATGAA

CACAGCAATAGTACGAATTCTTCTTCCAGTTCCGTTCAAGGAGATGAATCTCCGAAGGTG

AAAATAGAGGAGGAGGAGGAAGGAGCTGGCGAAGAATCAGAAAATGAAATCAGTGATGAA

GCAATAAAGCAAGATGATGAAGCAGAAGAAAGCGGTGATGAGGAAACTGATGCTACAGAG

GAGGCCAATGTGAACCTAAAGAGAAAAAGATATACTACTAGCTTAAGATCTGCTATGAAA

AAGGCAAAGGTTGCATTGCCGTATAACCCTATTCCCGAACTAAAGCTTAAAGCAAAGTTG

CTCGAGGATATTGAAGATCCTGCCCTGGTTGATCAAGTGCTCGAGGAGCTGGATGGTGAG

CTTTTACCTGGAGGGACTCAACGTCTTTGCTGGCGGCGCAATGCTGAGGGAACACTAGAG

TATTGGCTGGAAGATGCTAATCTAGTTAACAACAGGAAGGAGGCTGGTGTGAATGATCCT

TATCAGGTTCCACCACCTGGTTGGAGGCTCGGAGACTGCTCCACGCAACAGATCTCTATT

CTTAAAAGGTTTAGTAGAGAATTCTGTTCAATATCTTAAAATGTTGGTATTATAACACTA

ACCTGAATTTGTAACTTTAATTTTGTTTGTGAAATATACACTCATGTATGTACTAGAGTT

AATGCATTTTTCTTTCTGTTGAGCAGAGAGGTTCAAGTAGTTTCTCAATCACTAGTAAGT

TACTTCCGAAGATAATATTTTTATCATTTACTATTTCTTTAGACATTATTATCTCTATTG

ATAATTGCATACATTTCATTGACAGGAAGAATTCAAGAAAGAAGTAAGGGCTGTTATGGA

GGAAAAATTTTCAATGTTCTCAACCTTGCTTGCTGAAATAAAGAAAAATCAGCTAGTTGA

AAAAGAAACTCGAACGATGTCTGTTTAAAGTTGGTGAAACCACAACTACCATCATGATTC

AATTTCAGGGGATGACTGCAGGGATAAGAAAGTGAAGGACTACAGTCAGCATACCAGCAA

ATGCTGCTCTACCTCAGATACATTGTCGAATGATGAGTTTGTAGTTGACTAGTTCTGCAA

ACTTAAAAAGCCTACTAACTCTGATAATGTGGTCGTTTGATGAGCATATTTACTGGAACT

TAATAGAGTATAGCCTAAATTATGAGGTCTTCTGTTACATTTGTATTTGCAATTGAAACT

TTTGTTTTCCAACTTTTGATATACTATCATAATCAGATTCGAAGCTGTATAGGATTGAGA

TTAGATCCTACTTTCTTGGGCTCATACTATAAATAATCGACAAGTAATCATGAAGCCTTG

GTGTGCC

>XLOC_006131 transcript=TCONS_00009969

CTCGAAGGATATTGTAGCGACATATAAAAAGTCGCTGCTAAAGAGTTTTTGTGGTGACTT

TGTCGCCGCTAATGCTCTAATTATTTTAATTTTAAATTAAAAGTTAACACAAAAAAAAAA

AAAGGAATAATAAAAGACTCCCTCATATCTACTCCTTCCCCAATTGAATTCAGAACTTAG

AAGGGCCAAGTATTCCTCGACTGTATCGTCAGACCACGAAACTTTCCCATTTCTGCTCAA

ATCTCTCAAATTTGTCTTCTCATCTAAGCAATTGTGACCATCAATTCTAGTCTCAAAATC

CCTAATCAAGTTTTTACACTCCATTAGGAAAGTATGGAAGCTGAATACTATCACATTATA

CAAACTTGAAAGTCTCTTTCCTTCGCCGTACTTAGGTGGACTAGTTCAATATCATTGAAT

GTACCCTGTTGAGAGGTATTTGCTAACAGTTAAGCCACATCTTCACAACAAAGCTCGTCC

AGAAGGTTTATATGTGCTTCAAAACTGTGAAGAGGTTTCACATCAATTGTAAAATTGCTT

TGATTTTTATCTTTTCTCCTTTTCACTTTTTAATCTTCTCAGTTTGGTTGTTTGTTATGT

GAAATCTGAAAAATATTGAATGTTTTGCCCTTTAATTTCAAATTTTCAGTAAAGAAGAAG

TAGAATAGAGCGATCTTTCTTAGATCTTCCTAGTAGAGAATAACCTACTCGATTAAAAAT

AGAGATGAAGGCAACACAACTTTAGTTTACTCAACTATTTAAGGCATTTTTCAAGAGTAA

AAATTGTAGATTCAGCTGAATTTTAATTGAAGTGTTCCATCCGAACTAATGCTCTCTCAT

AATTTTCTTCAGTTTACTATATGGAAATTGACACTATCCCACAAATCTATAAATTGTAAT

TGCCTTAACTTCTTCTACCACTTTTATCGTGCGAGTATGAAAAACAAATTTTCCATGTAG

AGAAACTTAAAATATTCTGTTTGTTACTTAAATGAGGACTGTTGTGTCTTGAAATTCCTA

GAACCGTATATGCAGTTGTTTAAAGATTACACCATATGGATCATGAAGTAGTTGAATCCT

TCAATTTTTTCCTCCTTATTACGACTGCTTAAATGTTTAAATGTGGAGTTGTTGCACCTG

GACCACAGTATTTTACGCAATGACTGATTGTGTCTCATAGATATTAGTATAAATAAGACT

GCAATTCTTACAATTTTGGCAAGTCATTAGTCTTAGAATAAATGGTGGCAAATTTCTGCT

CTGGCACATTTGAAGATGTTGTATCCAATATAGATATTGTCGATGTTTTGATTTTTCAAA

GTGTGTTTCTCCTTACTTTGAAGTAAAGCAATTAACTCATCTACCTCCTCATCATTAAGA

CGGCTTCTTAAATTCAAGTCTCAAGTGTCATCTTTATTTGCGTAATAGAAGAGTCCTGAA

AACTTGCAAATAAAGAGGATTAGAAAAGGCATTTGTTAGAGTAATATCTCCTATCCATTA

TTCATTCCACTAACTCTGAAACATTAATGTTTGTCTTCTTTCTCCTAGAACTTTAAAAGA

GGATCAAAAGTTGCTAGAAGAACATGAACAAATGACCGCTGAGTTGAAAAGGAATATGGA

AGAAGATCCAAGAAAAGTTTGTTGATATAATGATTAGAATGCAATAGGTACTTTGTCTAT

GTACTTATCATTCATTCTTGGGGATTCTTCTTTCTTTCTTGAGACTTCTTCCGTCCTTAT

ATTAATTCTTTCTCCTTTTCATATCTCAATCTTCTCATTTTTCTCTCCTTTGTGTTAAAG

TGAGTTTTTTAATGATACTTATGAACTGTTTGATAAAAGGTCCGAGAGAAATATTGACTC

ATCACCCTGAAGTTATAGGCAGTTTTCTTTTAGATTATTTTGCTCGAAGTTTGGTTTAAG

TTTTATTATGGTATTTTTAATTTTCTCATTTTTATCCTTGATTGTTCCTATTATGTTGCT

GTAGTAAAAATATGTTTACAAAGTTCTATTGAAAAGACAGACTATTAGCTTGGTCGAGTA

TTCTTTAAGTTGTATCAGTTTAATACTTAAGCTATGTCATGATATAATGCAGCTCCATTG

TCTTAGTGTCTATTGATTTTGAGCTACTTTGTTCATCCAGAAAATATTTTCCTTCTTTTT

ATTGGGTGTCCATAGTAAAAATATTTGGGTGCATTATAATTTTCTGTAGTTGTGCAAACA

AGTTTAGAAGTAAACCATAAGTTTATATAATTGAAAATCTAGTATAATTTTTTAAGTAGT

AACCTTAACATAAAGCATAAATTAGTTTGCTTTTGAATTCCTGTTAAATGGTGGAGATAC

AGCCATGCTTCCCACCCTTTCCTTTATTTTCTTTTTGAGTTGTTTCAATCCTTCATCTCT

ATTAGAGTTGTTAATAATATTTAGTACTTAGTTGTGATGCAAAGTCTTGAAAGTATTGAT

CAATTTTTCGTTGTATGTTGGATCACCTGAATGAGTTAAAGCTTCAGTTTAGTGGTGCAT

ATATATTTCCGAAGGTGGGACTTGGGGTGATCTTGTGTTTATACTGAGGTGCAATATTCT

ATAGCCTCTGACTGAAAGTTTTGATATTTGTGATTTTTTGGATGGATAAGACTTTATAAC

CGTCCTTAGTTTGTTGGAATTTTTGTTGTTTGGACCAAAGAAACACACATTGTGAATTAA

AAGAAGCTATATTACTTTAGTATGTATACAAATTGGATAATGTTATTGGTATGATTGACA

AAACTGAATGATTGTAAAGATTAGTTTCTTTCTCTTTGTGTTTGTTTTAACTTTTTTTTT

TTGGTTATAAAGACATTTTTTATATGTATATAAAAACACACACAGAGAGTCTCTCTCTCT

TTATGCTTCAGTAAAGAAGTAGGTGTATTTAGGGTCACCTTTCTTTGAACTATTAGCTGA

GATATATTCAGATCATTTTTCAAGAACATTCTATTTGAGTTAAGTGAGTACTTCCTCTTT

TTAATGATATGGTTAACTGAAAAGTGTAAGCTAATCTCATATTAGTTTGGCTAGTTGGTA

CACGAATGATTATCCAAGAAAAATGAAATGATTAAGCTCCTTCTTTCACAAGTGCGTTTT

TAAATGAATATTTGAACCTGCTGGACAAGTTTTTGAGGAGCACACTTGGCTTCAGCTAGT

TGTTGGTCTGGTTGACAAATCAAATGATTTTGTGTATATGGCAAATTGAACTATGATGTC

GTTTTTAAGTACCTTGGTTGCTCCATCCATCACTCACTTCAATTACATACTGTTGGGGTG

GTAATGTACCAAGTTTCTTATCTAGGTTTGTAATGTTCTTTTTGCTAGGCAATACAAATG

TTATTTTGCATATTGCCTGTATGTGCTTGGAAGTTGATAATCATGTGTCTGGCGTAATGT

TGAGGGAAAATGGTTGGACTATATACAGATCAAATCTTTGGATTTCAGTTAGATTAAGAA

GAATCATTTGTGTTGTTAGATTGAGCTTCTCCTTCACAACTTTAATTTTAGTGGTTGGAT

TTTTTAATATAAAGAGATCTGTTATATTATTGCCTCTAATATTCAATATCAATGTCAACT

CCTAAATTATAAGTTGTTTACTACCTGAAGCATTGCATTAAGGGCTGAAATTATTGGATT

GATACATTTTATATGTGCTCATTCTTTTTTAGGTGGTTCTACTCTTACCGAACAAAAGAC

TCTTAACTTCTTGTTATTAGTAGTATTGTTGTGCACTCATATCACTTTTGTGTTTAGTAG

TTTGCTTTTTTAAATAGTTTGATAGTCTCTACTATAATTTACAATGGTTATCTTTCTGCT

ACTTGATATCATGTGTTGATTTTTCTTAATAACATGGATAATCCTTCTAGAACGATCAAA

TCGTGTTGACCAAGGATGAGGTTGCATACAAAGACACTGAACAACTTGATGTCAGTTAGT

ATATTGTTTATTGGTCATGTATATGATACAATGATAAAGAAAGTATTGTGTAGTAGATTT

TTTTGGTAATGAAAGTATTTATGTTATTGACATTATAATTATTTTGGTATTCTGTTGGTT

ATATGCATTTAATATTGGCATATAATGATAGACATATATTGTATATTAATTTATGGGATT

ATG

>XLOC_016519 transcript=TCONS_00027085

TTAGTTTGCGCCTCCCGCGGAATTCAAAAATAAGAAAAAGAAAACGAAAATTAAGTGCCT

TCCTCTGGCTATAAATATATTCCGTTTTTTCGGTTTATATCCTCATTCTCTTCTACATCT

CTCCAGCAAGTCCACCTAAGTCCTCTCGATTTGGAATATACCAAGAATCTCAGAGCAATA

CAAGAATTATTTTTAAGAAACGTCAAAGCAGGAAGGCCTAGCTATACGTGTTTGCAATAT

GGCCACCTTTGCCATCGAGCTGAAAATTCACACCAATTATTTTAAACGCAAGCTGAAAAA

GATGCTGGGAAAAATTGAGGGAGTCAATACGGTAAAAATTGATCAGGACCGTGGAATGGC

AGTTATCTCAGGCACGATTGGCTCTGCAGATTTCCTAACCATGCTAGAAATGCGTGGGAT

AGCAGCAGAACTCTTGTGGGAATTGAAAGAACCTGTTTATCGGAAAGTGATTGAAGATCC

ACTGAATGATCCAGATATCATGGCTCAACTGATGAAGCAAGTGAATGGACAGCATGGTCA

AAGGCTCCCAGATCCCCCTTTTGCTACTAGAGGCAATGCTAGTCCCTCGGCCCCTCCTTG

GCCATCTGATTATTATAATGTTCAGGCACCACCGCCACCACCGGCGCCACCTCCACCACC

ACCTCCGCCACCTCCACCAGCACCACCATATGATTATTATCATCAGGCAATGATGCCTTA

CTGCGATTACTTTAGTGATGACAATGTAAATGGCTGCATAATCATGTGATCTGCTGCCAT

GTACGGTTATTCGTGTGAATCTGTTGTTAAGGTGCACTATGTTTCTTTTCCCTCGTTAAG

GTGCACTTACCAAGTTCATTCCTGGACACTGCAACAGGAATTGTAACGTGTGGATTGTTA

GTAGTCAATTGGTGGTGGATGTGGCTCTCTGTCTCATTGTATGGTGTTGGGTGATCTTCA

CTCCATTAGCGTTGAGTTAGACTAGTTTTGTTCCAAGATATGTTGTAGTCAACCTCACAC

TGTTCTCTAGGGTTCCCCATTTTCCGGGCTGGGC

>XLOC_000019 transcript=TCONS_00000032

ACTAAACTATTTATTTTTATCATTACAATTAAAAAAAGAAATTTTAACTTTTTTTTATCG

GCGTAAATAATTATATTTATCAACCAAGTGGCAATGTTACGAGGATTCCAGAACTAGCTA

GTCCTCATACTTACCATACAAGTATCAACCAAGTGCCAGTTCTATTGGACTCCAGAACTA

GTCCTTATACTTACCACTCCTACTTCTATCCTTCTTGATATTACAAAATCAGAGCAATTT

ATAAAAATATCTAGACAGGGAATCTATCAAGTGTCTCCAATATTTTCCTCCATTTGGGTG

TGGCTTGGCTTCTGATGTGCAATTGTAGAACAGCTTCTCTGATTCTGTGCTCTGCTCTGT

GTTCCTCTCTTGGAATCTTCTTGTATTCCTATCCATCCATGTGAGCTTTCTACTGGTCTG

TTCTCTCCCATCCAGTGCAACAGTGTGCCCCAAATTTGTTTGGAATAATCACACTTGAAG

AACAGGTGGTCCACACTTTCCTCTTCCTTCGATCTACGCAGCACGTTCAGCAGGTTCTCA

AACAACTCAAATATTCTTAACCTCCAGCGTCCAGAGTCCTGGCCATACTTACATACAATC

ATATGCAGATGCGAGCCCCTGTATCTTATAGTTTGTAATTTATTAAGGAGTACTCATATG

GTTCACAAACTAGTAATGCAAAGACTACAAAATGCAGTGACATGTTAATGATAATGCATG

GATTAGTTTAACCTATAACAAGAAAGAACTCTTAGACAGTGATACTATTATAATCTATGC

ACCTCCTAAGGACACTAG

>XLOC_032595 transcript=TCONS_00053568

CAAAATCTGGAACCCTAAAATCATTAACCCACCGCCCCCTCTTCAAAACTCTCTCAGAAA

CCCTAATCCTCCAACACGCTAAATTTCTTCCGTTGTTTAGTGGTACCTTTCCACCACCAC

AACATCGTCTTCGTCTTCCTTTTTCCTTATTCTTCCAATAAATGAGAAATGGTCGTAGCA

TTTCTACTTTTTGCTGGACAAATGAGAGGAAATTAAGTACGATCTGTAATCTCATCCGTC

CAAATAAAAGGAAAATTGATTCCCCATGGTTGGATCATCTAATTGAGGTCTGTTTCATTT

CAGTGCTCGCAAACAGGTATAACCTCATCCATGCATATGTCTTTTATAATGTTATAGTCA

CTCTTTTCGTAGCAAACTGCTGATTCAGGCAATTTGCACTCCACGACACCCTTAGGGGTA

TATAAATGAGTGTCTGTACGAAACAAATCTAAAGCAGAGGTAAATGGTGCATTAGAGACT

ATAGATCCATTTTCTTTATCATCAATGAAATCAACAATCATGCAGCCCTCGAATTCTGGA

ATATTTCAGCGGTGATCTCTATCGTCTGTATCACGTGCGAAAGGATTTCCATTTCTCTTG

TAAGTAGTTAAATTAGACAGACCTTCCAATTCATCCTTCAAAGGTGAAGCTATAATTTCA

TCTTTGTTACTTCTTGAAATATGCATCAAAAACTACGGTGCCATCAAGTTGCTGAACCTT

CCGGCGCTCATTCATTTCTTTTGTTTCAAACGCCAAAGAGTCTTTACTGCTTTCATAACC

ATTTGAATCAAGCAAGTCATTTGCTGGACAGACTAAAGACTTGGAGTCTTTGAAATCATT

TGAGTTATTTAATATCTATCCAGATTTTCCTTCATGGTAGCCTTCTCTTCAGCTGTTCAA

ATACATTGTTATTTCATTCAAATGTTTCAGAATCAT

>XLOC_015809 transcript=TCONS_00025873

GGCATCGGGAGTTAATATGTAACTTTAAGGAGCTTCATTTGGGATCACATTGAGATTCGA

CACCATCCACTAGTGGGCCTCGAGGTTGACCGAGTTAGAGTATCTTTTTATTAGCGGTGA

ACCGACCCTTCGTCCATGGTGGCCCTTTAAATAGTTTATTTTTTTCGCTTTAATGTATTC

CCGAAATTTCAAAATTGTATAACTTATTCCACACTCTTAGATGTTCATGATTCGGGGAGT

ACTTAGACATTCTATTGTTGGGA

>XLOC_034344 transcript=TCONS_00056509

AAAACTTCCTTCTCCATTGTCCCTCTTCCAAGATCCTTTCAATAATCAAAATATCTAGTG

CCAACACCCCCTCCCCCCTCAAATTGGATGGGTGAGTAGCCACGCCCAACTTGCCAACGA

TTGACCTATGCGACGACCCCAATAAAGGTTTTTTAAACGCATCAGCAAGTTGAGAAGCAG

ATGGAATATAGGAGAGAGAGATTATCCCGGCGAGAAATTGGAGCCTGACAAAGTGACAGT

CTAACTGCACATGCTTCATTCGTTCGTGGAAAACTGGATTTTTTACGATGTGAATGGCAG

CTTGACTCTGAGTGAAGAGACACCGGTAAATTGGGGGAAATGGACAGGTCCTCGAGCAAA

CTATCCATCCAGGTAATTTCGGCTACTACACGCCTCATAGATCGATACTCCGCATTGGGG

GATGATAAAGAAATTAAAGGTTGTTTCTTTGATTTCCATGAAACATGTGAAGCCCCAAGG

CTGATGTGAAACCCGCTAACAGATCGATGGGTGTCGGTATAAGTTCCCCAATCAGAGTCG

TAGAAAGCCATGAGCTGAAAAGATGGAGTCAGATTCATGAATAAACCAAGACCCGAATTT

GTTAATAGATAGTGCAAACAGTGATAAGCAGCATCGAGATGAGGACGATGAGAGGCCTGC

ATATATTGGCGCAAATGTTGAACCGCGAAAGAAAGGCCCGGTCTTGTATGAGTTAGGAAA

TTCAATTTTCCTAATAAACGATGCTAAAATGTTGGATCTGAAAGAAACTCGCCTTCAGCA

AGACTGAGTTTGATAGTAGGATCCAGAGGAGCAGAAATAGGCTGGAGATTGAGATAGTCA

AATTCAGCCAACAATTCCAAGGTAAATCGCCTCTGACTTACAATTAATCCTTGTGGTTCT

CATATAATTTACATGCCTAGGAAATAGTGAGCATGCCCCAAATCTTTAATTTTAAATTCA

GAATCCAAGAACAACTTTAAAATGCGTTGTTCAGTAGGATCGTCCCCAGTTATGAGAATA

TCATCTTTGTAAACAGCGACTATAGTGATCTTTCCCTCTAATTTCTTGAAGAATAATGAG

TAATTGTTGAGAGTGTGTGAATATCCCTTGAAATTCAAAGTTGCAGTGAGCCTTGCATAC

CATTGCCTTGAGGCTTGCCGCAGCCCGCATAGTGACTTTTTTAGGAGACAGACATGATTA

GGTGAGGGTGTTGTCATTCCTGCTAGAAACTTTATGTTTACCTCTTCCTGCAAATCTCCG

TGCAGGAAGGCATTATTAACATCGAGTTGAAAAAGTTCCTATCCTTTTTTTACAGCTACT

GCAAGGAGAATGGTTGTCATCTTGACTACTGTTGAAAAGGTCTCGATAAAATCAATACCT

TCCCTTGAGTATCCTCTCGAATTACCAAACGAACCTTGAACCTTTCCAAAGTACCACCGG

ACTTATATTTGAGCTTATATACCTATTTACAAGCCAAAGTTTTTTTCCCTTTTGGCAATG

GAACTACTTTCTATGTCTTGTTTGCTACCCAGGGCATCAAACTTTTGTGCCATAGCTTCC

TTCCAACTAGGATGATGGGCAGCTTGAGAATAGGAAGTAGGTTCAAAAAGGTAGGAGATA

TTCTTGATCAAAGACTGATATACATGAGATAGCGAAGCAAAAGAAAGAACTAAGGGCTAA

ACCGGTGCAGCAAAGCAAGAACTAGTTAATTGAGTGAGATAAATAGAGTTACACACATAA

TCATGCAAATATACAAGTGGATTAGACACCTCGCTAGATTTCCTTAACTCAACTTGAGGG

GAATGTTGCACCCTAGGAGTATAATGAGCGATGGATGGTGTAGGAGAATGGAAAGGTGAA

TGTTGATTTTGAGAAGAATGAGGTGTGGAGAATTTAGGATAATCTGATGGGAGACCAAGT

AAGGCTGATGCATCAGTGATGTGAAGAGAAGGTTCTGTGAGTATAATAGGTTGTGTAGGC

TATATAGTAGGAAATATAGAACTTTTTGATGAAGTGGAGGAGAAATGAAAAATAGTTTCA

AGAAACTTCACATCCCTAGACACAAAAATCTTGTTATTTTTTAAATTGAGCAGCTCGTAC

CCTTTTTTTGCCAAATGGATAACCCAAAAATACACAAGCAACAGACCTTGGATCTAGTTT

AGATCTGTTTTGTGTCAGTGTGCAAGCATATGCTAAACATCCAAAAAAATTTATAGATTG

ATAGTTTGGTGGACTTTCAAATAAAGGAGTTTTAAACTTGAGAACCTTAGAGGAAAACCT

ATTTATTAAAAAGGCAGCAGTAAGTAAGCATTCCCCCCAAAACTTGACGGGGAGATTTGA

CTGATAAAGTAAGGCTCTAGAGGTTTCTAGCAAATGCTTATGTTTTCGTTCAATAACTCC

ATTTTGTTGAGGGGTATACACACAAGTAGTTTGGTGAATAATTCCATAGGAAGAAAAAAA

AAGTAGATATATTCAAACTAGATCCTAATTTCAATGCATTGTCCGATCTAATGACTTTAA

CTTTTCTGTGAAATTGTCTTTCTGTCATAGCTAAAAATTGTTTAAGAATGGTGAAAGCAT

TTGATTTGGTTGATAAAAGAAATGTCCAAGTTCCCCTGCTAAAATCATCTACGAGTGTAA

GAAAGTACTTGTACCCATCATGAGTTGGTACCTTATGGGATCCCCATATGTCAATATGAA

TTAAATCAAATATTGCTTTAGAGGAAATCAGACTTGATTGATATGGTAATTTGACTTGTC

TTGCTTTAGCACAAATTTCGCAAGACGCAAATAGATTAGATGAACTTGAACAAGAAATGG

AAGAAATATCTCTCATATTAGAAAATGGCAAATGCCCAAGTCTTATATGCCATAGGCTTA

CATTTTTTGACATTGCTGATGAACATAAAATTGAAATTAATGACTGCTGGCTACTAGCTT

TCCTATTACATTGTAGATAAGGAGAACTAGTATCCTTATTTCTACTTGAAAAATCTGAAA

CTGGAATGAACGAATGAAGCAGGCCTTATTTCTGCTCACTAAGTGCCACTGGCCTATTCA

GAGAAGGGGCCTGCAAAACAGCACCAAATGAGGAAAAAGTAAGTGTGCAGCAACATTGAT

GACAAAGTTTATGAACAGAGAGTAAATTGAATTTTAATGACGGAACATATAACACATTTG

TCACAGTTAGATTAGGCAAGATGTTCACACTTCTTGCCTTGAAAACTTTGACTTTGTGTG

AGTTAAGAAGATTGACATATAGGGGTTTTAATAATGGCTAGACATCGCACAAAATTTCTA

GATCATAGGTCATATATGTTCAGATGCACCAGAATCTAAAATCCAAGAAATAGAATTGGA

AACAATTGAATATGAGATAGAAGTGGTTTTACAAGCACAATTAGCAGCAATATTTTCTTC

TGTGTTCTTTTCTCCTTGAGTCCCAAGCTTTACTCCTTGAAGAAGCTGACATAGAGTATG

ATATTGTTCTTGGGTAATAGATTGTTCTCCCAAATTGATTTATTGATTTCCATTCTCTTC

TGTCATGAGTGTTGCATTGCCATGAGGTCCTCCCTGGGGTTTCTTTCCTTTTGTGAATTT

GAAGTCAGGAGGAAATCCAATTTTTCTGTAGCTATTCTCGATAAAATGTCCAGATATCCC

ACAATAGCTGCATTCAACATAGAGCATTCTTTGATTAAAATTGCCTCCTTTTTCAGTAAA

AGTAGGTTTTTGAACAAATTTTTTACTCCCAAAGTTTTTCTGTGCAACCATGAAAGCAGA

ACCAAGAGGTTGTTGATTCAGTTGCACTTCTCTCTGTTTCTCATCTTGAATCAGAAGAGA

GTATGGTTGATTAATGATGGGTAAAGGTGAAGTCAGGAGGATGTCCATTCTAACTCCTCC

ATAAGCTTCATTCAAACCCTTTAGAAATTGGATAAGTCTGCCATCTTGATGAGCCTTAAG

TGTCCTTCCCTTTCCTCCGCAAGAACATCCACAAGTGCAATGAGCGCAGCTGGTATAGGA

TAGCACCATTGCTTTGTCTAAATCTATCCTCTAGTTCCTTGCATATTTCTCTTGTTGTCT

TGGAATATAGCACGCTTTTTGCTATCTCCTTAGAGAGTGAATTAAGCAACCAAGAGATGA

CCATATCGTTACAATGATTCCAGGCGTTGAAAAGTTTAGAATTTCTTTCTGGGGGTAGTG

ATGCTTCCATCAATAAAGCCAAGCTTATTTTTGGCTGACAAGGCTATGAAGATGGCTCTG

CGCCACCCTCCATAACTCTTGCCATCAAAGATTGAATTTACAAGAACAATTCCTGGTAAA

TCTGAAGGATGGAGATAAAAAAGCTAAGACGAGTCATTGACAACTGAAGAGGATTATCCT

GATGAAGATCCAGAAGATGGGATGGTTTTATCTGCCATTGATGCAATTAAAAAAACAATA

CAGGATTGAAAAAGATATTAGATTGAAAAATTTAATCTAAAGCTTTGATACCATGTTAGA

AATCAGAGAGATTGAGAAAAACTTGAATTCTTTCTTGTGTACTATGTACTGCAGATTACA

TGTATTTATATAAAAATAATAGAAGCTGCCTACCATATTCTATACTAGGCTTTCCACAAT

ATTAAAAATAAATCAGTTAAAC

>XLOC_024303 transcript=TCONS_00039987

TTTTTTTATATAATTTGTCCGGTATATGCTTTATCACAAAAATACTATACATCAGTAAGC

ATAGTTGAAGTTCAGACATTTGTTGAAAGTGCAAGTGATTGATTGATCTTGCATACTTGG

CTTCTCCGTTTAATTCAGTACATGAATGGACATGGATAATCAGACTTCTATGTGTTTTCA

ATAAATGGGAGGTATAATAGCATTTGCATATATGCTATCTATGTATTATGAACGTGCTTC

CATGATGCTATTTTCGACTTTATTTGGTTTTCTCTGTATGCAAAACAAAGCTAACAAACG

AATTTAAAGAAAATGTTAACTTGCTATGGTTGCTGAAGATAGAAAGCTCCTCTGTTGTTT

TAACTGAGATTATTTGTGAACTATTTATCTATGTAGCTCTTTACCCCAATCCCTTAGGAT

ACCATCCCTCGTATTTGTGTTTATCAATTATGATGGTAAGGCCTGATTCTTTTAACATAC

TTTCTAGAAACAACCAGTGGTGCTTATCTTGTGCTTTAATTTATTTATTTTATTGGCATT

AGGTTAATGTACCTGTGCACATGCTTCAATTGGTTCTAATCTGCCAGTCTAAATAGTTTA

TGTTGTATGTGTCAGGCAATTAATATCTTGACATCATGGAGGTTGAAATTGTTATCACAT

TATGCAAACTTGAAGCTATTTTTCCTCTGGTATTCTTTGGACCAATTCAATTTCAGTGGT

AGTACCTTATTCAGAGGTACTTGCGAACACTACAATCATATGTTTAGAACAATGCTCATC

CAAAAGGTTCAATTGCAGAAGGTTTTTTGACGGACGAATGCCTCACATTTAGCTCACAAG

GAATACAAGTTAAGGTGGATCAAAAGTTGCCAGAAGAATGTGAAAAAATGATTGTCGATT

TAAAAAGGAATGTGGAAGAGGATTTGCAAAGAGTTTGACGAGGAATGTGAACACGTGAAT

ATAGGAGTAGACAAGAGGATTCAGGTATAACTGGACAAGAAATTCAAGAACAACTAGCTG

CTTTCATGAGCACAACGTAACAGGTACTTTCTGTCTCTATGTTTTTTGTGAGCATATGTG

CCTATATATACTCTATATACATTCACAATCAATAATGATG

>XLOC_015669 transcript=TCONS_00025637

CCCCTATATCTTCTGGACCTTTCGTCAAACCCTTTTACTTTGCCAGAAAAGAAATCCCCA

AATAGCAAATTGCAGCGGTAAAATTTTTAAGAGCCCTAAAATCTCCAAAAGACTGCAATT

GTTCATTTTTCTAAGTCAGAGTGACGATTATTCACTCCTCATCGCGGGCTTACAACATTT

CTTATTATAAGGTGAAGTATTGCGAAAACTGGCGCTGGGATCGCTTTGCACCACCGATGT

CACAACCCTTCTTTTTTCTCTTTCGTATCTCGATCTTTCTGGCAACAATCCTCTTCCTGT

CCTTTGGTTGCCAGAAATGTAACCTGCAATGCTTCACCACCAACAGTTAAAGAGCCAGTC

GTCCCTTTTCCTGCTCTGCAATGCCATCAAGTGATTTCTGAACTTAAATCATCAGTGATT

GAAATAAGTCCTCAGCACACAAGCTTGCAAAGTTCTCCTAGTAGGGAAGATTCTATATCC

TTTAAGATTTTAATTACAGGATTTATTTTAACTGGCAAACTTTATAATCACATGTTATTA

TACACATGTTATTCTCTTCAGACATTATTGTATTACAATTATGTGTGATGTAATGTATCC

TTGCAGGTTGCTTTTAGGACTAAACATTTTCATCTAAATATACTGCGTGAACATATTTAA

AGAGCAACATACCCCTGCCAAGCTAGCTTCTTTCTGTAAGAAGTAATAGTGTAAAACAAA

TTTAAGATTAGAACTGTAAAAATAGTAATTCTCTCTCTCTCCA

>XLOC_033286 transcript=TCONS_00054747

ATAGAGAAATGGACACTTAATTACAAAATATTGCAAAAGAGAAAAAACCCTAGCCTCCTT

TTCTCATTTCACTTCTTCTCTCCCTGCCCTCTTGCCGCCTCCTTCTCCGTCGTTAGTTCA

GCTGCATTCTTAAGCTACTAATCTCATTGTCCAGATTTGTAGCATTTTCTGATATTTTTA

TTGTTTTGCTTCTTATAAATGAGACAGTAGGAGTAGACATGGCTGATGTATATCTGATCC

TCTGTGGACATGCCTCAGCTATTCGGCAATTTTTTTTTGTTTATTTCATTCAAGTATGGT

GTTTTCAATGAAGATGTAGTACTTTTCGATAAAGAACTTGCATTTTCCAGTTAGTTATGG

TGTTTCCAAATATTTCTGCAATTTTCCGACGACTTTTCCTACCGGACGGCGGTACTGGTG

GCGGTAGACGATAGGGGGCAGCCGGCGGGAGGAGAATGTGAGGTTGATGCTAGGTTTTGT

CCTGTTTAGTTGTTTGTAGCAACTTGTATATAAAAATAATGCTATATTTTGTAATATTAA

TAATGCTACTTTTTGTAATTAAAGCTTCAAAATTATATATAAGTATACAGAACCCTTTTA

TAGTTTTAACATTAATGTGTGTGTGTGTATATATAAGTTTTTGAATGAGATTTTTTAATG

GAGGG

>XLOC_022791 transcript=TCONS_00037474

AGCTGTTCAGTGTTCACACTCAACCATTCTCTTTTCTAGGCGTTACGTTTCTGATTTTTT

CTTATCAGGTAATTATCTGTTGAAAGAAGATGGCTAACAATGGAGAAGCTTTTGGGAGCC

ATGAATCTGAAAGCATTGGTACGGAGATTCACATAAAAGTAAAAACTATGGATCATCAAA

CTCACAATTTGTGCATTGGTAATCAGGAAGTCCTAAATCCTTACAGGCATTGTCACGGTA

CCTGGAAAGGTTGCGACGAGAACATAGTGTACAGATGAAACAGGAAGCGAGGTGCTCGGA

AGTGCATTTAGATCTTCCACTTCTTCAGTAGAAGGGATCCCAAGAGCAGAGCAATTAGCA

CACCTATTAAAGTTTTTGGCTTAATCACCTAGGAATTGGAAGGGGATCTGCGCCATAATC

TGAATGTGATTGATCCGAGTTTATGCCACAGAACTCAGTCTAATGCACAGAAAACCGAAG

AATATTGCACAATCTTGGTGCTTACTTTCTTGAGCTTGCTTGAACAATGATGCAAGTACA

AATGGGTGACTGAAGGGAATTGGAAGGGGATCTGCGCAATAATCAGAATGTGATTGATCC

GAGTTTACGCCACAGAACTCAGTCGAATGCGCAGAAAACCGGAAGAATATTCAACTTGGT

GCTTACTTTCTTGAGCTTGCTCGAACAATGATGCAAGTACAAATGGGTGACAGTAGGAGT

ACTCCCACGGTCAATGCTGGACCTCCATTTTATAAAACTGATCTGGGTCCCAATCCTCTC

GCAATCCAGGGAGAAGGTTCTAACTCTGATTTGGTGAATGATAATAGTACTGATCTGCAG

CATAGGCAAGTTGGTACAGAAACAATGGGAGAGGCAGGTGCTAGATCTCTGCAGATGCTT

AGGGAGGCAATTTCTTCTTCTAATGAATACACGACTGAGAGTCTCCACATAATTATGCTT

TCTCGCCGTCAACTTGAGCAAGCTATTGCAAACATTCGACAACAAAACAACACTGAAGTT

ATGGACATGCATGAAGAAGCTGCTATGAATGGAGCAACTACAGACAACACTGGCGCAACT

ATAGCCAATGTCACCGAGGATATTGCAACAGATGTTTCTTCAGGACAATCAACTGGAGAA

TTCAGAAGTACTGCTCATGAATCGTCCATTGAACCTTCCGATGGAAATATTGGGAATTCT

CATCAACAAGAAGATGTTGCACAAAGCTCACCACAAGCAAAACGACAAAAGATGGACTAA

TACTTCTATCGTGGGAGCAAATTTTGTTATTGTACATCATGCCTACTAGGGTGTATCTGC

TGGTGACAAGATGTTGCCAACTATTACTTTCATGAGATAAAAGAGGGGAAAAGACCCCCT

CAAGTCTTTAAATAAGGATTGGGGACAACCTTTTCTTTTTGCTTTTCTTTTATTCCCGGA

AAGTGTTAATTTGTCATTAGTGGGTTAAGTAGTGGGGCCGGGGATGCTTTCGTCAGTTTT

GAAAACTTTGGGGGAGAACTTACGGGGTCATAGTGTAAACTTTACACACATTATTCAATT

CGCACTTTCCCACATCTGCCGATGGTACACATTTTTCGAACAACAACAACAATCAAATCA

CCTTCAGAACCTTTCAAATCCACACTTCACACATTATTACACTTGCACTGATCATTTCTC

TCAAACCAACCACAACATAAACAATTTTCTCTCAAACCAAATACAAACTCAAACCAAAAA

CCTCTCTTCAAAATCTCTGATTCTCGTCTAAACTAATATCATTTGGTTTAATTTTAGCAG

AAATTGTGATCATCGTCGACATAACATCAAAATCAAG

>XLOC_022021 transcript=TCONS_00036184

GTCAACCCCAAGGTCTCATTTTTGATTTCTTTAAAAGAGGCCAAGGATTACCAGTGATGG

GGACGGTTCCCTGATCTCCTTCTCCTCTCTACACACCAAATACATATATACAGTACAATC

ACAATCAGCTAAGGACACACAGGCAGACCTTTTTCCCTATCATCTTCTTCACTTTTCTAT

CAACACAAACCAATACGCAGTAACATCTACACACATTCTACAGTGCACGCATATACAGTA

CCCGATCACCCCCATCTTCCTCACAAACACACCACCTTACACTCCTATTACCCGTGTCCT

ACCTCACCATCTCCAATAACTCACATCCCCACAAAAATCAGTATATACCCATTACAAAAC

ACACACACAACTAGTAAAAAAAACATACACGATATACAGTGAGATCGGGGGGTGGGGAGA

GGGGTACAGATCGACAACAGGAGAGCGCAGCAGCGGCGACACGTCGCCGGAGACGGCACT

GAAAAGGAGAGTAGCAACAATGCTGCGATGTTCGCTGGAGTTTTGTCGAGAAACCTTGCC

AGCGATTGTTCCTCTGCTTCTGCTTGTTGTTATGCATAATTTGATTAAGTCGACACCATT

TGCACTCGATCTGTTTGTGTATGTCTGTTCAATTTTGCAGATTGTGGGATTACGATCGGA

ATCGTCGTATACCATCGTTTAGTTCCACTGGGAAAAAACCGTCACCGACCACCTCTGCTC

GCGTCGCTCCTCCTTTGAACTGTTATCTGGGTCGCCGGCGTTGACTCTTGCTCGGCGATG

CTATGGTCCAATCGTTGGTTGTTTATGGATTGGAGATCATTATCCTTTTACTTCTTTGCC

TAAATCTTTCGTTTATTACTTCAGATTAACCCGTTCCCAGTTTGACCCATTTACTTTTGT

TGGATATCTTGTTATGTTACTTTGACCCGCATACTGGATATTGTTATTTCCTATATTAAC

>XLOC_016639 transcript=TCONS_00027268

GCCTTATTTCCTTAATCCGATGTTGACCATAACGATTTCTCCCTATTCCGCCAGACCTTG

TCCTCAAGATCGAATGTTAGCCATGAATTTATGGAAGTGAGTCTTCAAATCCTAAGACGC

CTCTTTCGGACTAAGATTAGCCCACTGAACTAGCCATTTCTCTACCACTGTATCATTTAC

CCTAATACGACGTCGCTGCAAGACCATCAAGGGTCTTATGAGAACTCAAGCGTCACGATC

GCAGAGAGAAGCTTCCAGTTGAGTTGAAACTAATGGCCTAATCCTCATCTTCAACTACGA

TATATGGAAGACCGGGTAGATTTGAGACCCCGGTGGTAACTCTAATCGGTTTGACTAGAT

AGTAGTGGTCATGCAGTCTGCTAGGAGGCAATGATTTGGGTTTTGCAAAAAGATCATAGA

ATTTATCCAGCACTTCTTTGATTGCCAGAGGTATGCTCCATTCTAGTGATTCTTCCCTGG

CTGTCACAGTGAACAAATGAGCCCAAAGCACCTTCCCCTTTTTGAGCAACTGTTTAACTT

ATTGGCCAGTCATGCTATGCAATTCTCCTTCCGTACTTAGTCCTTTTAGCTCAATTCTCT

TCCCATTTTTTGTTGCTTGTAGCTTGTTTTCAACAAAATCAAATTGAACTAGGTTGTGTC

TTCTCATCTAATCCACCCCTAGTATGATATCATAGCCCCCTAAATTAAGCAACCTCAACT

TGTCTTCAAACTCCACCCCCTTGTGTTTTCCATCTAAAGTTAGGGCATGATTGTACACTC

AGTAATTGACTACCATTAGCTACAATGACTCTCATAGATGTATCATCCCTAATCACACAA

ACCACTTTCTTAGTTGTCTCTATGGCCAAAAAAATAGTGGGTGCTTCCAGTATCCACAAG

TATAGTTAACTGCTTTTGATATGCCTTTAAGCCTGATGGTGCTGGAACTTGAGGTTCCTG

ATAATACATTGAAGCTTATGGATTCATTCAGTGCACTCCCCTGTATTTCATCTGTATCTT

CTAAAATTTCCTGCTCCATTTGGGGTTCCTCACCATTCTCCTCTGCCAGTAGGTCATTCA

GTTGTTTACTCTTACATAAAAGACCAAGAAAGAACCTTTCCCCACATTTATAGCACTCAT

TTCTTGCCCTTCTTGCTTCAAATAAATTCTGGGCTACAGTAGAGTTAGTATTGTTAGCTG

AATCTCTTCCCCATCTATTATTTGCTCTACTATTGTAATTTGAATTCCCATTAGACATAT

TAGAGTTCTGCATGCTTCTTGCTGGGGTTACTCTGTTTTTCTATTCTGTACCCCCATCAT

TCCTTCCTGGTGCCTAGCATTCTCCACTCCCTGGCTAAGGGAATATGGTGCTATGAATCT

AACCATATGTTTGATATCTTCTTTAAGCCCCCCTATAAATGTTTCCAAAAAGAAGTCCTC

TAGGATTGTGGGGTTCCTGCCCAAGACCCAAGTTCTTAGGGACTCAAATTCCTCTAAATA

TTCATTAACAGTACCGCTCTGAGTGATTTTATTGAACTTCTCTATTAAGGTGTCATTTCC

CACTTCAGCAAATCTTTTACGCATCTCCATTGTGAACTCCCCCCATTTCACTATACCCCT

ACTTCTTGGAAAGAGTAAAACCAGGCCTCTGCATTCCCATCAAGGTGTAGTGTAGCTGCT

TCTACTTGTTTTTCACTTATATTGTAGATGCTAAAATACTTTTCACACTTTCTTAACCAA

GAGAATTTGAACTTCGGAGGGAAGAAACCCACCTGATTCTTGGGAGCAGTTTCTTGATTT

TCTGGAATTCATGATCGAATAGCTAAACTTCCTGAAGATTCACCTCCTGAATCTTGTTGC

TTCATCAAGCGATCAAATAGAATATCCATTTTGTACCCACAGCACCAAGCTTTTTATTTG

TTTCTATCATTTTTGATTTGTATCCTCTAGTTTTTTATTTGTATTTGCCATGTATTGTTG

TAATTGATATTCCACTTTAAAGATATATGTTCTTCCGTACTTTTTGCGCGTAATTCCATA

ATATTTCACAGTGC

>XLOC_036054 transcript=TCONS_00059230

CCTATATCTTCACCTTCTCTGCGAGGCATCGAATGGATCCAAATTCATGATGTATATGGA

GTTACTCTTTTTTCGTTTCCTTACAGGAGACAACAGTATTAAATAGCAATAAAGCAACGA

CAAAAAATAACAGTATTTAACAGCTAAAGGGCAACGAAAGGTAAAGCCGCAGCTATTAAT

GCAAGTCCATATGATATAAGCAGGACTATGTCGACATTAACAGTGTCGACATTAACAGAT

AATAGTGATCAAATGAACATTTATCCATTTAAAAAGTCTTTAAGAAATTGAACATTATCT

CCTTAGTTAGCCCTTGCAGTTGGGCAACGAAAGGTAAAGCAGGAGCTATTAATGCAAGTT

TATATGATATAAGCAGGACTATGTCGACATT

>XLOC_027136 transcript=TCONS_00044685

AAATGAGCCGTCAAAATATTTGCCATTTCTCTTCTCGGTTCTCCAACTACTGTATACTGG

ATTTAGGGCCAGAATTTATTTTTAATTGATCCCAGTATCTCCACCCTAGACACTTCCCTG

CCGGACCCCAATGCCTATTTTCTACAGATCGTTGTGTCTCAATAACATGGAACCTGCCCT

TCATTTTGAGCACGACTCATGATGTTCCTGTTTCATCAACAAAAGGGTCTTAGTGACTAA

TGAATATCACTTGGTCAATTTCATATCCACGCTTGCTCCTTTTCCACAGATCGTGATTTT

TCATGTCATTATGCAACCCTTAACTGTTCTACTTTTAATCAGATATTACTAGAACAAGGA

ACATATGCAGGTATTACTGAACGTATGCAAGAATATCAAGTGATCGCGATGGTGATACTA

TTGGGGCCAAAGTTGAAGTATTTATATTCAAAACCAAATCCAGGAAGATGTAAAGAGAAT

AATATCTCAGAGTTTACACATGTCTGCAATTGGTTCAAGACAGAGCACTAGTAGTAGACA

TAATGATCATTGTTTGACCAAACCAAATTTTGTTGCTTCCTGCAGCGTATTTGGTCCGAA

GGTATCGTCCATCAACACCTCCTTCATATCTCTAGTTGTGCGCGGGCAGGATCTATTAAA

TTCAGGTGCTAGTGTTTGAGCATATTTTGTGCAAATAAGACCAATAAATTCTCCTTGTTA

ATGTACCATACCTATAAATTTGACACATACAGTTTCTATCTGTTAAATTCTTTTAGAATC

TAGTCAACTCCAAAACACTTCATTTCGGGTTGTCATGTGCTTAAAAGTCATTTCGGGTTG

TCATGTGCTTCTTTTAATGTCATTTCATCATTTTCTTTCACTTTTTGGCCCCTCTCTAAG

TTGATCTCCAGACTTTAAAAGTTTCTACCCTATGTTGACCTTTAATTACTACTACTATTA

TTTTCTTTAGGCAAAAGAAAGTACTTTCCACTTCCTGCAATTATGTTGTACAGAGCTGTT

TTAGTCACAGAAGTACGATGTCGTTAATAGTACCAACAGTGAACTGTTAGCTCTGCAAGA

CTTCCTGTTATGGATTTTTTGTCAACCGCAGTTTCTTTTCAGAGTTTACCTTTAACAAAG

TACCGTGGCACTTGATGGAAGTAAGTTTTAATACCAGTAAGTTTTAATACCACGAAGA

>XLOC_030823 transcript=TCONS_00050721

GAAAAATAAACCCCACAAAAACTTCTGCTTTGTTTCCCCTCTTCTCTCTCAAGAAGAAAA

AAAGGGAAACCCTACAAATTAAGAACAACAAGAACAAGAACACTTCATTCTTGTGTATTT

GAGACACTGCTTGTTTTGTGCTATTACAAAGAAACGCTTGTTTCATAGTTTTGGTTAACA

TCATCCTTCCTTTGTACATGAGTTAATGAAACAAGAAACAGCTAGCTAAGCTTGTGTCTT

GTGGTCGTGTATATATGAAACAAATCTCTTTTTTTCTCCTTAAATTAAACCATCTTGCCT

CTTCTTTCTTTCTCAATCTCAATCTCCTTCTCCCCTCATCAGCCTTCCATTATCTTTTGA

TTTTGTTTGCATTCATAGTCTTAGAATGCGAGGGAAGATTAGGACGAAGTGATTAGTGAA

GTCGTAATTTCTTTCTTTTCTCGCTTCCATCGCGAAGTTTGACTTCTTTAACTGAATTCT

TTTGATATATCTTCATCTCCCCATTCTTCGAGACAACCACGCTTAATTTCTTTGTTTTAC

TTATACAAAACTATGGTACTGTTAGTTTAGGTTGTTTAATGAGTCATATATATAAGAAGT

TTAAACATAAGTAGGAGTATTTTATGGAGTATGTGTTATGTATATGCCTGGCTCCCTGTA

TGCCATTTGCAGAGTTCATCGGAACATCGGTGGGTCTCCGTGAATGGCGTATGAGGAGCC

AAGCATATCCTAAATTTGGTACATATTTAGTAATCTTACTATCTAAAGCTTCATCATTTT

ATTTCTTTTCCCCTTTTTGTTGTGAGAGAGCTGGTTTTGTAGGCAAGGTATTGAAGCTGA

AGCTATGGAACGGGCTACTTAACATTATCTACTACCTCAATTACATTCCAAAAGGGCATA

GGGGAATCTGACACTACAAAGGAAAATGTTTGGGTTGATCATGGACCACACCACACACTT

CTCTTCTTCCTGCTTGGCTTGGCTTTCCACAGTGCCTATATGCTATGTCATCTCTTGCTC

GGGTTGGTTACTAGCGAATAAGATTATATAATCAATGCAGGTCAAAATTTTGACCCAATA

AAGTGAGTAAAAAGGACAAGTCCTTCTTGGAAGAAAAGAAAGACAATAAAGGAAAAGAGA

AGGAATGGGACCACTATTTTCTTAAGATGGATCTCCTGAAACCAATTGGATTTGGATGAT

TCAAATCAGTTTCAAGTTCCAAACACCCCTTAACCTCTTTACCCCCCCGCCCCCCGCCCC

CAACAACTCCTTGACGGTTCTCACCAACCCAAACTTGAGGCTTTTCCCTAACCAAACTCT

TATTATAGAGAACATTCTCCTGGATGACCTGAAAAGTAAGGTAAAATAAACAAAAAGAAA

AGAGGTAAAAAGTAGTATAAGAAAGAAAACATGGGTGGTCCCTTAACACCCTTTTCCATT

TCCAAAAAAGAAATGTCACTTTGCAAAAATGTACTTTAATGTAGATTCTGTTCGACTTTA

ATCAGTAAAG

>XLOC_023023 transcript=TCONS_00037851

GTTGACCCGCGAACTTCAAACTTAAAACACAATTCCCTTTCCCTCTTTCCTTGGGTTTAC

AGACACAAAACAGAGAAAAACACCTAAATCAGTTCAAACAACAATCTTCACAACAACCAT

CTTCTTCACTGCTTATTAACGGCTCTGGTACAATGGTGAAAACTACACAGAAATCCTCCT

CCTCTTCTGTTTTACCTGCAGCCAAAAGGCCAAAACTAGGAGTTGCCTCGACTAGTCAAG

CGCTAAAGGGCAAAGCAAAGGCCAAGGCAAAGAGCGAAGCAAAACCAAAAATGCCGAAGG

GCGCAGTCTCTATTCCACTGCGGGATGGAAAAACCAAGTATGGCTTACCATTTGTGAAAA

AATGGGAAAGCCAAGCTTGGTACAAGAATTTCAAATTAGCCGGCTACATCCCCAAGTTTT

CCATAGATGAAGGTAAATTAATGAGGCGGTTTCGACATATAAGGGATGTTATTCACAAAA

TGAAATGGGGGTCCGTCTTTGAAAGTCCGGGGGATGTCAACCTTGATCTTGTGATGGAGT

TATATGCTAATTGGGACTTGGAGCAACTAGATGACTTGGTGAAGGTTCGAGGGCAGTCCG

TAAGGTTAGATGCTTCAGCTCTTTGCACTTTTTTAGGTGCGGAAGATCGTGATCTTGATG

AATTGAAAGATTTCATTCGCAAGCCGAACTACAAAGCAATGAGGCAGACTTTATGTGGTG

ATGATGTTAGTCATAGGTGGGGTAGAAAGAAGAAAACGGGTTGGCACAAGTTTATGCAGT

TTTGCAAGTTTCAAAAAGAAGCCCGAATTTGGCTCAGGTTAATCAATGCAAAAATTATTT

CCATCTGACCACTTTACTGATGTGATTAAGATTAAGGCTTGCATAGTATACTTTTGCATG

ACGGGTAAACCCGTCAACCTTGGGTACTATATGATCAAAGCAATGAAGGAAACACACGTC

AACAAGTCTAAGAGGTTATCTTTTGGCCGTGTTCTCACTCAGTACCTAGTGAAAGAAGGT

GTACTAGTTGATACCATTGGTGATCGCATTGTTCCAGCCATGAAAAGAAAATTTGATATC

TTGGCAGTGCAAGGTCCGAATGCAAATGCTTATAAACTATTCAATGATGAGCGGCGTGAT

ATGCAAGACAATTTGGAAGGCCGTAAATACTTGATAAATAATTTTTGGCAAGACAAGCAG

GGATGAATATGGCAGTACTTGATCAGCAACTTCCATTGTTGGATTCGTCTAAACGGTTAT

TGGGAATGATTCAGACAGTAGAGGTGCCTCCTGAAGAGGATGAAAATACTGATTCTGACG

TGATTGATGCGAGCAGCAGTAGTGAAGAGGAAGAAGAGGAAGACGATGAAGAAGAGGAAG

AAGATGAAGAAGAGGAAGAGGAGGAGGAAACACGCACTCCACCGCATCATCCTGGGGGGT

CTGTGAGAAGCAAGGCTGTTGTGCCTACTGTTGATGAGCGAGATGATGACACTCCCGCAT

ATGCAGAGCGGGAGGATTCTGATGCTGGTGTTGATGCTGATGATGAGACTTAGGTCGGTG

GGCCTTCAGAGAAATTCATTTAACTCTTGCACTTTTTTTATTTTTATCCCATTGAGGACA

TTGCGATGTTTAAGTGTGTGGTGAGGGAATTTCCGAAATTTTTGAGGTTGTGGATGACTG

TACTTTTAGGAATTTCTTTTAGAAATTTTTACTTTTGTCACTTGACTTTATTTTTATTTA

TTTCTTGTTTTAATATATAAAAATGAAAAAAAAGGAAATTAATGAAAAATTCCAAAAAGA

ATTTCTTTTTAGTTTTAGTTAGTGGTACATGTCATTCGCCCCGTGATTTTTCTTATGGCC

TCGGTTCTTTCCATGGATAGATCGTTGAACCGGGTAATTTTTATTTTTAGGAGTAGTATA

ATATTAGTTAGTAGAATATTTTCA

>XLOC_031898 transcript=TCONS_00052446

GGGAAAGTTAATTATCTACCTTGCCAGTTTTCATCTTTCAAAAGACCTAGAAGTGAAGCC

ATGGTTAAAATAATTGAAGAATTAGCAGTTACAGCAGTCTTGTTTATTGGCGTTGCTGCC

TTCATGAGCAATCTTGATATGAGAAAGGCTTATGATAAAACCATGGGAAGGGCTCACAGA

GAGTTTACCACGTGGATCAATGGTGTGGTGGCTAAGGACGAGGCTAGAGGAACACAAACT

GCGGATAATTAATTAGCACTAATTACCTTAATTCCAATTAGAGTAGACGGTATATATATG

CTTTGTATGTTGCGATCAACAAAATAAATATATTTTCTGATTGCATGGAATTGGTACAGT

ATATTATACTCATCTAGTTTTGTGAAATTATCACATATATATGAATGGTTTTGCCTCGGA

>XLOC_029164 transcript=TCONS_00047945

AAAAATATGAAAAATATATCACTCGATTCCTTTCGGCCTTCCCATTCCATTTCTCAACTC

GATTTTCTCAAATTCATAGCGGATTCTCGTAGATCGGGTTTCAAAGTTCCGATTGAACTT

GACTCCGGCGACTTGTTGCATTTTTCCGGTAAGTTTTTGGCTCTTTCCTTTCTCCTCACC

GTCCTCTCTCACCCTCTCATCCCCATTTCCCAAAAACGCGTCCCCTTTCTCCCGTTTTTT

CCCTCCCGTTTTCTCTATTTTCTTTTCTATTTTTTGTTGTTTTGTTTGTCTCATTTTTCT

TCTTCTCTTTCTTGATGGTGGGTATATGTTGCCACTGTTTTTGGCGGTGATGAAGTGTCG

CGGGGGCTGTTGTGATTTGTTGCAGTTGGAGTCGAGGTGGCGTACCCAGGCAGTAACGAG

GCAGTGACGGGGGATGGAGGGGCTGTGACGTTTGGACAGTGGTGCGATGGTGTTGTGGGC

AGTTTGGTGTGGCTGTTGTAGTAGTGTTTTA

>XLOC_022823 transcript=TCONS_00037518

TAATACCTTCATAAGTTTAATGTATAGTGGCAAATAAAACATTAGCCGCTACTGATACTA

TTTTACCGGCAAATAAAAGAAATGGCCATTATATCTCTAATAAAAAGTTACTTTTATTAA

CTCCAAAATATCTCTGTCCCTACCCATACTAACTTGGAAACCTAGGGAAAATATTGAAAG

CAGTTGATCTCATCTCTGTTCAACATCACCGCCGTTCACCGGCTCCGTTCGACGTCGCCG

CCGTTCAACGTCGTCGCCGTTTACCGTATCCGTTCAACATGCGCCGTTCCCGTCGTCATT

CAACGTCTCCGCTGCTCACCGTCGCCTCACGCCATCTCCGTTCTCCGTCGCCGGCCACAA

TCCCAGGTGTATGAGGCTTACCAAGTGCTGTCTGCTTTCCAAAATGCTACGCCATAAAAG

TATCTTGATGCACTGGCTGAGGCAAGAAGCTGAGGGCATATGCAGCTCGGTGATACTGAT

TCGATGTTGGGTCTAGATTGCTCCATGAGTCTTACAAGAAAGGCTTGAAAATACAGATTT

AATCTTTAGGGGACACTGATCCAAGAGTTTCGGAGAGTTGCAGATTCCTGGGACTTAGAG

ATGGATGCTTGGTGTGGCGGGAGTGCCTGCTGCTATTCAATTTTTCCTCATGCTATTTTT

ACCCGAGTCCCTGCGATGGCTCTACATGAAGAAGAGAAAATCTGAAGCAGTCACTATTCT

ATCTAAGATTTATGATCCTTATCGGTTGGAGGAAGAAATTGATCAGCTTGCTACTGCATT

AGAGGAAGAACGGTTTAGAAAGCAGGCTATCAGTTACATTGATGTTTTTAGGAAGAATGA

GATCCGACTTGCGTTCTTTGCTGGAGCTGGACTGCAGCAAGCTGCAAATGGGATAGGCCA

GATTTCATTCAGTACTGAGGAAAAGATCTCTCCCAAGAAACCTACCACTCTGCCAAAAGT

GGCAAAGCAGATTAAGTGGAACACTAGAAAGTGACGCAGATAGCAAAGCGAAGAAGCAGC

TGTCAGATGCAAAGAGCAAGGAACTCAGTGGAAATGATATCTTTGGCCTCCCCAGAAATT

GCTCCTAGATCTTCAAGGGAAATGTTCCTCCTGGATCTGTAGAAAAGTCACTGAGCAGCG

CAAAGCTGAAAGAAATGAGTGGCAATGATATTTTCTCCGATGGAAAAGTTGAATCCAGAG

ATTACTTTGGTGGCGTGCGCAAACCACCAGGTGGAGAGAGCAGCATCGCACTAGTTTAGA

TAAATGTCATCAAACTGCTTTATTGTTAACTTGTCGTGTTCCTGCAACATCTCAGTTGAT

GACACTAGTGATGCTTTTAGTGTTTCATGGGATTTAGAACTGAGGGTTGTAGATTTTCTT

GTGTTAACCATGTTGTACGACAACTTCCAAACTCTTAATTTATATCAACTTGGAGGATTG

TTGTATTAAAAAAAATTATGTATTTCGGTAGTGAATGATAGTATATTTTATTAGAGATGA

TTGTATTTATAAATGAATGAGAATAGTATGCTT

>XLOC_024124 transcript=TCONS_00039663

ATTTTCCACGGCCCGCAAGATTCTCCCAAGAACCACTGCTAGATTCCGCCCTCCGCCACT

TTTCGGTCAAATAATTACATGTCATAAGGTCACACCCCCTTGGCCCCCCTCACTTCAAGC

CCTGTTTAGCTTGTGCCCAAAGTGAAGGTGACCAAGAGGTCTGCCTATTGGTTTTCATTT

CTAATGGTGACGCTGTGAAGTATCGACGCAATGCAGTAGTGGCGTGGCGATAGCAGCTTT

TGGCTTCACGGCTTTTAGCCCTTTCTTTCCCTTCCTTGACTTGAAAGCGCCATTAACTTG

ACTATTTTAGTCAAGGAATGGAAAGGTGGTGGTTGAGGCAAAGGTGAAGGTGACACAGCT

ATGACGGTGTGGTGATGGGGCTGTTTGGGAAGCAAAATAGAGACAGTAGTGAAAGGAATA

GCAAGCTGAGGTTCCCGTTTGGTTGTTGAAGCTATGGTGGCTCGTGGTATTGGAAATGAC

AAGTGATGGTGGCGTTATGGAAAAGCAGCAGCGGCAGTAGTAAAGACAGGCAGCAGTGAA

GGTGCATCGTCGAGGCAGACACAACTCGGGACAGTGGAATGGAGAAATGGTGACAAAGCA

ATTGCATTTTTGGTGCATTTTGCTCGCTGACGATCAGATAAAAAAAAACTCCAGTTTCCT

TTGCTCTTTGTTTGTAATCTAATCCACGTATTTAGATTTGGTTTGGTGATTGAACTAATG

TTTCTTTAGTTATAGCATGAATTCACCCATGTATGTTCGTTTCTATTAGTTAGTCATACT

AGTTTTGAGAATGTCTCATCTTGTTCCATTAGATATTTAGTATAGTAGAATCTTGTACTA

GTTTAGGATTGGTATAAGATATATCGTGTATGTGAGTGTCGTTTTACATGGTTGGTTCAC

TCGAATA

>XLOC_010373 transcript=TCONS_00016886

AATCCCAAGGCTGCTGTTGACGAATTAAATTCATGGTTTAACACTGGGGATATGGTGCTT

TGCCCTCCAACTAGCTGCTTCACTCCTACGACCATGAGTGACATATATGACCAGGAACTA

GGCAAATTCTTCAGAAATTATACTCGTCAAAAAGAAATACAAAGGATAATAGCTGCTACC

ATTCCATTGCGCTACTGAAGG

>XLOC_037501 transcript=TCONS_00061365

TTTTCTTTTTATATAAAGTGATACTCTCTTGCAAAGAAGGGACTCATAAAGCTATAGGAA

TGATATGGTTGCTGCCTTGGGCTTAACCAAAGGGCCCAGTTGAGTGGACAAAGACAACGA

AGAAGGAATAATAGATTGCGTATCTAACAAACAAAAGTATACCCACTGAAACAAAAACAC

ATAACATGACAGTTCAGCTAAGTTCCTAACCTTAATTGACAATGATTCTTTGTTCGTACT

CCCATCTTTTTTCACTAATTTAATTCCAATTAATCAACTTCCAAGCAGTAGTATTGACTC

ATCGAGTCCACACAAAAATCAAGTGTCTCGAGATTTTTTAGGAAAAGGCAACTAAGATCA

AGACACACACACCCTTTAGGTGTAGAAGAGCCTAATATATAATATGGTGGGTATATTTTC

AAGATTTTCTGTCAGCAGAGCTGGCCATCGTCGGGCTCAAAGTGCACTTGATGAAAGGGA

AGTGTTGCCCCCAAATACAGAGGCAATAGGTGCTGCTACTATAGCTGGAGCTCCCTCTCA

TGGTATTGAAATTGCAGTTGAGTTCAAGCCAATTGAACACCCAACTGAGCCTCTTGATAA

TGATCAACCAATTCAATGTCCGTTGCCAGAACCTTCGGTACTAAATGTAAGGTTTCCATT

TCTA

>XLOC_030067 transcript=TCONS_00049495

CTTGGATTTTCCTACTAAATCTTATCAATTTCACCGCCCATTATTCTCTACATAATTCCC

CTTTGATCAACTTTTTCCTCCCCATATTTATTCAAAAATCACCTTTTTTGTTTTGAAACA

ATCTTTTTATTTTCTTGATTGCGAATGGCTTCTTTCAGTGTCCATTGTCCCAAGATTTCT

TCCATATTAAGTGGTTCATTACAACACCAACCCCACCATGCTTCCATTTCCTTTTTATCT

GATTTTAGATCAACCACTTCATTTTCCTCTTATCTCTAGTTTTTTCTTTTCTTTTTTTAC

TAATGAATTAAATCTTGAAATGAAAATGTTGTAAATATAATTTGTTTTTAATAACGTGAG

GTGTCTTTTAGGGACCCACTCGCCTCCAATAAAAGGTGTTTAAGGTGTGTGCCAAGCTCA

ATGAGGTAATTTACTTACCTGTACACCTTTCGTCGAAAAATTATAGTAATGTGTATATAC

GTCAAATATTACTAGAATTTTTATACATACATATTATATCTTGAACACCCTTAGTGAAAT

TCCTGACTTTGCCATTATTTATTTACTTTTCCTTAATGATAGTTTATGTTTGTGAAAAAT

GTTGTGACTTCATCGATGAGATGGTCTTGGTTTAACCATTTTTCTTTTTTACTGTGGAGT

TTTTAAGGTTGTTATTGAGAAGTCTAACAATTCACTACATTAAAAGGAAAAGGAAGTGAG

CACAATTCCTGATGCTTCATTTATTCAAGAATTCTCGTCTCAAGCTGCCCATCTCGTTAA

GATTGAGACGTAGTTGTAGTAGTTGCTTTTGTATTGGTTTCTTGTGTTATGCTTGTGGAT

ACGAGGGAGGCCGTGGAGCTACAACTAAAGCAAAATGATTGTGAACTAGTTATAATGAAG

AAGGAAGCATTGCCCAACCAACCATTGCGACAACTCTTGTTAGTGTGGCTCCGCCTTTTG

TTTCATATGCACTACAATTGCCTCTGGGAAGTTCTCCTGCCCCAGCAGCTCCAACATCTT

TCGCACTTACACCTCCTACATCTTCAAAGCCTAAGTCATTGGATTGGTAGGTACAGTCTT

TCTTCTAGGCTTCTGATTGAACTTTAAGGTAATGGGAGAGAAGGCAATCGGCTTAACATC

AAGTGGAAAGCCGGGTGCTACTGATGCTGCTGCTGCGCGGCTGTTGGGTATCACATCCTC

CATTTAAGTGACCCCTGGCTGGAACATTTTATCACTCCCCGGCCCCAAGGGCACCACTTT

TTGTGAAGGTAAATACACAATGCTTTCACCTATCTTTTATGAGCATTACTGTCAGCTATA

ACTTAGTTGTCATTGACCAAGAGGATGAAATTTCAGCGGTCATGTTGAGTATGTTATAGA

TATCTTCTCGACGTGTTTAATGAAGTAGCATGTAAATAAAGAATTTACAACTCTAAAGCC

TGTAAACTTACGTTATGTTCATCAAATTTGGATCTATGGTAGATTTGTTAAAGCAGGGGC

CTTTTTTCTTCTTGTGTGAATGGATATTGCATATTTTGGTGGTTGAAAATTAGAAGAACA

GGAGGTTCGAATCACTGTTACTTCCATTTCTTGATTCTTTTCATGTCTGCTTAGTTGAAT

GTATATGACTCACTTTCAAAATAATATTACCCCTTTTCATGTCCCTTTTTGCAATTTTGG

GGGGGTGGGGGGGTGGGGGGAGTCCATTGTTGGTTTCTGATGCATATGTAGTGTTCCATA

ATTAGTAGGCGACCGAACCTCCTTGTAATTGTCAGGAAATAAAAATGAAAATAAGAAACT

AGAAGAAAAGAATAATTTGTTTCCCTCTTTGCACTATAGCATTTCACTGAATTTGTTTGT

TTTCACACAAAATTCTTTGCATTAGAATATTTTGTGTTTGAATGCATCATGAAACCAAGC

AACATCCAATTAAGGTAAAATCCACAATAATACTCTCAAAGTTTTGAGCTAGCCTACTTT

TTTCCCTTTTACTTTCTATATTCTTGGTCATGCAGTTGTCTCATAAACATGATTAAACTG

ATAATTTCCAAGGTAGGAGACAAGGTCCAGAAAGGACAAGTAATTTGCATCATTGAGACC

ATGAAATTGATGAGTGAGATCGAGGTAAGTTCATGCTCAATATCCACCTAGATGGACTCC

TAGAAGATGCACATGGTAGGATAACCATATGTGCAATCAGATAATTCATGCCAAGCATTT

TAAACATTCGATTCTGGTTTCCTTTCCTCTTCCATATGTTTTTTATTGTATTAAGGGACC

TACTTGGAAGATGTACTTGGGCGTGTCATGTGCATGAGAGGAGTTCCATTACCTTAATAC

TTTCCATGAGATATTGCTTATAAAGGCTCCAACAACAATATATCCAGTGTAATCCCGCAA

GTGGGGTAAAGGCGCCCTTAAAAATATTTTAGCATTTTTATGTTGATCCACAAGAAAAGT

TGACAATCATAATTTGTTGATTATAATGTCCAGAAATGAATGTCATATTGAATTGTATTA

TGGAATTGATGAGTTATACATAGTGAAGTTGCTGGGGTCGCTCTGTTCTAACTGACTGAT

CAATTCGGAACCGTAGTTGAGATACTAGCACAAGATGGTAAGCTTGTTAGTGTGGACATG

GAACCATGAGGAAGAAGAAGCAGCGCTGTAATGGGCTACGTAGGTGGAATCGAATGATGA

TATCAATTATACCATCATATTTATAGATTGTATTCGGAATTTATAAATTCTTTTGAAACA

TTGCAACAATCTTACAGAGTTGTAGGTTCTTGTCTCTAGTATATTGTCACCTCGCTCTCT

CTCTTTTTGAGTGTACCTACATGAGTTTCTTGAAGTCCTTTAATGTCAGTATATGGCGCA

CGCTAGCTCTAGCGGCCAATTAAATTTAAAGTAGTCAAGACTTAAGATCTATTACTAAGA

GCTTTTTGTTTACCTTTAAGCTATCAAAGATATGCTAGAAATACAACATTCATATTCTAT

>XLOC_005185 transcript=TCONS_00008439

ATTAGCATTTTTTTCTCCAGTTCTTTCAAGTTTAAAAGATTGTATCTTATTGAACCAAGA

TATTTCATTACATTGTTTTCGACTTTTAGTACTTATTTATATATTTGAACTTCCCTTGTT

GTTATGTTGCTGTTAAACGCTGCTGCTGGTGCAGCAGCTGCCCACCCCTCGCTACAGATA

TGTTAGGTTCCATTTGTGTGAAGATATTGAAACATAAAAAGTTTTTAGATTACTTTAATT

ATAATCTTTTATTTACTTTTTTATGCAAATGACATAAGAAAATAACGATAGACGTCGAGG

TCAGGATAAAAAGAAAAAAAATAAGGTGAAGAATCGTATCGTTAACGATGATAAATTCAC

TCCTATCTCCGTCTACTGCTACACATGTATTAGATTCCAATTTGTGTGAAGATACTGAAA

CATAAAAAGTATTTAGGTCACTTTAATTCTAATCTTTTATTTACTTTTTTATGTAGATGA

CATCAAACAATAATGATAGAAGTCGAGGTTAGGGTAAGCAGGGAAAAAATAAGGTGAAGA

ATCATGATGATACATCCACTCCATCTCCGCATACTACTACACATGTGTTCCGTCCGCAGC

ACCCACATTTTCCATCATCTAGCCACACTACTATGTCTCCTCAGTATGCTCCCGGGATTC

CCCCGCCTAACCATGTCTTTTCTTCTATGCCATCACAAGGTGATAGCTTGATGCCTCCAC

CTGGCTATGGTAATGGTCATCAGTATAGGCCTCCACCTGGTTTTCATCGATCACCTTCTG

CTGGTTACCATATTAGGGGATCTTGTAGACCCATGTCTACCATGGCTTCACCATCTCCAT

CTTCAACGCGTCGATCTCTATCAGCGACGCCGTCTCCACAGGTATCCCCTAATATTTCAG

GACTTCACCTTCGAGACAGTAGTAGCTCTCCAGTGGAGGGGGGAATCCTCCCATTATGGT

TTCTGGCACAGATAGAGAGATCCGTAATAGGATTACCATCAAACCTGCAGGGATTGGGTT

TGATCCAAAGGCTACTTCAGCAATTACAAAAGCAATTC

>XLOC_037527 transcript=TCONS_00061395

TTTTTTCATTCCACACTTGAGAGGACCTTTTTGTGTCATTTTCTTCTGCCTACTACTCCT

TCCTCCGCCACTTTCCTCCGCCATATCTCCACTTTCCTCTGCTACTTTCCTCCGCCTACT

ACGACTCCTTTCTCGACATGCTGCTCCTACTTCTCTGCTAATTTTTGGATCTTAAAAGAA

ACCTTCTATTCTTACAGGTGTAGATTCAAGTTTTTGAAGATTTGTCAGTTTTGGAATTTT

CTACGGCAGAGAGGCTGTCCGTGCGGGAACTAAAGCAGCTGCAATTGCATGTGTTACTAG

TGCAATTCCTACGGTTTGTGAATTCAGCTCTTCTTGATCTGATTCTTTTCCAAGAATTTT

TTGTTTTAGACTATACTCTTGCTATTATGGCAGACAATGCTGTCTTCGAACTTCATTTTG

GCTCTTTAGTTTCTCCAGAGTTCCTTTTAACGGGTATAGTCTAAAACAAAAAATTTATGC

GACAATTAACTAAACAACATAATAAACCCTCAAGAAAACAGCCTTCACTTCAAGCAGTTT

TTTTTAAATTTATGCGACGATTTCATCAACACTTTCACGGAACCATTGTGGACTAGTTGG

AGGAGACTTTGGGATAATCCTAAGTGTCGGGAAAAGTCAGAAACAGCTTCACGGAACCAT

TGTGGTGGTGAGAACGGAGTTGCACATGGGACTCACACACGCGGCTCTATCTCTATTGGG

GAGCATCGTAAGAGACTTGTTGTTGAAAGAGGTAGAGATGTAACACCAAGTGAGATACAT

TTGCACGTCCATACACATGATCATGATGGAATCTTTTGTTGGAGAGGTACGAAGAAATAT

TACATGAACAAATAGAGACTCAATCTGATATAGATAAGTGACAAGCATATTACCAAGCTG

CGGGAGGAAAAAAGAAGAGAAGAGTGTATGGTCTTGGATTTGCAGCACAAAGCTACTACG

GGTCGCATCTTCATCCCTCTACTAGATCCGATGCTTCATCCTCTGTGCCTTCTCCAAATG

TTCACTGATAGGGAATATGGATGAGTTCATGACACAATTGTTTCCTGCACTAACAAACCA

CATGCTTCCTATATATGTTGATCGAGTGCGGGAAATGATTTCTTTACCCTTAAATCAATC

AAATAATGTTGCCGACTCTCCATCATCGATAGCACCCATAGTCGATGCTCCTACTGATGG

AAAGATTGATGAAATCTATGCATCGGGTTCGGATGATGACCGTAACCCTTAGGCTTGTAT

TAGTAGACTTTTCCTTTCTAGTTTTTTTTTTTTGGTGTCGTGGATGTTCTAAACTTGACC

ATACAGTTATTATATGCATTTCTATTTTGGACCTCTTAGCATTTTAATTTAATGAGAT

>XLOC_006432 transcript=TCONS_00010455

GTTGTGCTTTATTTTGGTTAATCTGTTTCTTATTCCTATTGCCCGAAGAACTCATAGTTG

TGTTTTATGTGTCTTTCTATTACAACCAGCATCGTATACTTCCTATTATATGGATTAAAT

ATCATGTTGTGTATACAATATCATTCACTCTTTCCACTTGTAGTTTTTCCTGCTTAACTT

TGTGAATATTTTTATGTACCTTGTTGACTCCTTTAAAATCTATTTGTTTAACTATTGTAT

GGAAGGTTGACTAAGCCAACTTGAGATGGCTGCACCAGGGAGATATTGGAGATACGTTAG

CATAGTACTCCATAAATATATGATTTTGTTCAATTACAATTGAGCTGATCTTGTTAGATT

GGGTGCCAAACCCAGCCCTTCTGAATACTAACTAACTAATGTTGTCTAGAGGCATTTCAC

ATTACAAGGGGGAGGTGTGGTGAAGAAAGTAATTTGCTTATGTGCTGAGAATAAGGTTTG

TTCTCCTTATGAAAAAATAAGGTCTGTTCTCATCATGAAAAAATAAGGTTTGTTCTGTCC

AAAATTCGCACTATTTATATAGCCAACCCTTACTTTGCTCTGTTCTGGTTTTATTTTGTT

GATGGAGGTCAAGAACAAATGAAGGTACACATTTGTCGATGGTGTAGTTTATCAAGATCA

TTTCTGGTTGTTCACAAAATTACTTTGATTATGCTAATCCTATTGCTAATGTGAGCGGAT

ATTTTGGGGTTGTATACTGATCTAATAAAGTTACCTGGGAGCCTACTAGTTTATCTTTTC

ATTTACCTCCTCATTCTGATGTTTTGCAGGTCATCCATGAAACTGGTCGTTAGTTAATAG

TATTTGCACCCATGAGACAAGACTTGTAAACTTCAAAGGGCAATTTGGATTTCTGTTGAT

CTTCAATTCCTTAGTACTTGTTCTTCTTAATTGAGGTTTGAACATCTCTCTTCTCTGATG

TCTTCTAAAAACTTTCTACTTAGTGATTTTAATTCTTTACTAGTTGATGCAAATTATGCT

GAAAGGAATGCAACAACAAATAACTTATTCTTTCGTGTTATCGGTTCGAATTCAAGTAAA

TTCTATGTTTGTCTGAGATATTATATATATAGAAGATCCTACCAGATTAGCTTAGCTAGT

CAACTTCCTCTAGTCTTCTTGTTTACAATATGAGGACTTTAGCCCTTTGTCTTATTATGA

TGTAACCAGCATTGTATATTTCTTGTTATGGTTAACTTTTCTGCTGTTCATCCACATATT

CATCACATGACCCGTGTTCTCAACACAAACTAACCTGTTACAGTTCAAGATTTATAGCTT

ATGCTCATAGATCATTAAGTAATTATGTTTCAAAATTCATAAAATTATGTGAAGTCATGA

AGGTCATGAAGCACTTTTTCCATTCACAAAGGTCATGGCACGGGAGCCGACCCATTTGGT

GCGCCTACAAAGTTGTGGTCGTGGGAGGATGGCACAAATTCCCTGGAGATGGCTAAGACG

AGGGTGAGATCCAACTTTGAGTTCTTGGACAAAGAAAGATGATGCTTCCATAACAGGGGC

ATTGCTCCTCATGGTAAGACACTGGAGGAACAAATGCTAACTCGGGTGAAGTGGTGGCTC

TCGCGGAAGAGCTTCAAGTAATTTTAGGTATTTAAATCAGAAATAGTTTGGTTCTATCCT

TGTCATGGCATTTGCTAAGTTTTGTACTTGAATAGAATTTGTCTTGTGTTAGAGACCTCT

TTGTCAAGGGAACAGTTAGAATCACGAAGTTATAATTTTCTAATCTTTCTTTTTTATTAC

TCCTACTTCCTTACTTATAAACTTTGATGCATTTTAGCATGTAAACTTTAATTCTCATTT

ATTTTAAGCATTTGCTTTGTTTGTAATGATTAATTTATAGTTCTATGTGACTTATTATTA

GTAAATGTTGGGAATTTTTGGAATCTCATTGTAATATAACATTTGGTGG

>XLOC_014615 transcript=TCONS_00023924

GGGGTGTCAAGAAAACCTGAATGTCGCACGTTCGATTCCTTCTGTCAACAAATTTTATTT

TTCAATTTTAGGCGTTTTGCTTAACTAGCTATTTTTAAAAAACGACGTCGTTTTTAGGCC

CCTTATTATTATTTTTTATTTTTAAAACGTGATACTTGATACTTAAAACATTGAATAAAA

CAATTGGGATTAAACAAATCCCTAATTTTAGAAAGAGAAAATCTCAAACTTTTGATACTG

TATTCCTCTTCTCTGAGTTGCCACCATCTTCAACTGGTCTTCGCCGGTGGGACGGTGACT

GATTTCTCGCCGTCCGTCTCTGATCTCATCTCTCGATCCTTTGATGTGAATGATTCTCAA

AAATCAATGAAGAAGTACTTTGCCAAAGTACCTAAATCAAGTTTGGCGTCTCAAAATATA

CCTGTCCAAATCCAAGAAGAAAATGCAAATATCCCGGAAGCATCTTTGCATTCTTCTCAA

GAATTTGATTTAGCTTCTTTGAAGGCCGATCCAGCAGAAAGAACTGAAATCTTGAAGTAC

CATCCAAATCACCGTGATATAATTAGAAGAGCATACCTTCAGAAAGGGCCATGTCAACCT

CGGAAGCACAAGTTTCCAATCACAAATTTTTCTGGATATTTGCGTCGTTTTAGTCCTGAT

TGGTTTGATGAATTTGATTGGTTGGAGTATAGTATAAGTACAGATAGAGCATATTGTTTC

AACTGCTATCTATTTAGAGATGATAGCCTTCATCAAGGTGGTGGCGAGGTATTTTCAACT

AAAGGGTTTAAGAGTTGGCAAAAAAAGAAAAGCCTTGACATACATGTTGGTGGGCCGAAT

AGCATTCATAATCGGAACAAAAAGAAATGTGCTGATCTTATGCTTCAACAACAGTCTATT

CAAAGTGCATTTGAGAGGCAATCTAATCAAGTTAAGCACGACTATCGAGTCCGTTTGACC

GCTTCAATTGATGTAGTAAGGCTTCTTTTGAATCAAGGATTGGCATTTCGGGGTCACGAT

GAATCTAAGTCATCACTTAATAGAGGTAATTTTCTTGAATTACTTTCATGGTATGCAACA

AAGTGTGACAACATTCGTGATTTTGTATTGGAGAATGCTCCACAAAATGATCAAATGACT

TCTCCAATGATTCAAAAAGATATTGTGGCCGCTTGTAAGTTTGAAACAATTAAGGGTATT

ATGGAAGAATTAAATGGTGACTATTTTTCTTTGTTAGTTGATGAATCATTTGATGTGTCA

CGAAAGGAGCAAATGGCTATTGTTATAAGGTATGTAGATAGAATGGGATTTGTGATGGAA

CGACTTATTGATATTGTTCATGTTAAAGATACTAGTGCTTCGTCTTTAAAGGGAGCAATT

GTTGATTCACTTGCTCAACATTCCTTGAGTCTATCATATGTGCGTGGACAATGTTATGAT

GGGGCAAGCAATATGCAAGGTGATATCAAGGGTCTTAAAAGGTTGATTATGCAAAAAAGT

AGATCGGCTCATTCAATACATTGTTTTGCTCATCAACTTCAACTTTCTCTTGTTGGAGTT

TGTAAAAAATGTGTTCAAGTAAGTTCACTTGTACAATTGGTTTCAAATATTTTGAATGTG

TTAGGAGCTTCTTTTAAACGTATGGATGGATATCGAGAATCTCAAAGAAGAAAAATTCAG

GAGGCACTAGATATAGGTGAGCTTACAACTGGCCGGGGTTTAAATCAAGAACGTGGTCTT

TCTAGAGCTTGTGATACTCGTTGGGGATCTCACCATAGTTCATTTTGCAACTTTATTCTT

AATTTTGGCACTATTATTGAGATACTTGATGAGCTTGTTGTAGATGCACGTTCTACTGAT

GATAGAGCCAAGGCATCAGGGTATCTCGAAAAATGTCAATCATTTGAGGTTGTATTTACA

GTGCATTTGATGAAAGATGTTTTAGGAAAAACAGCTGAACTCAATACATCCTTACAGAAA

AAAGAGCAGGATATTGCAAATGCCATGCTACTTGTTCAAGTAGCAAAGAGAGGGTTGCAA

ATATTAAGGGATAATGGATGGGATGAGCTTCTGAACAAGGTATTAGCTTTTTGTGTCAAG

TATAATATTTTGGTACCTAATTTTGATGAGCCATATGTTAACCTTGGAAGATCAAATCGT

AATCCTACTGATTATACTTCCTTACATCATTATCGTGTTAAATTATTTTGTCAAATTATT

GATTGGCAACTTCAAGAACTTGATGATCGATTTGGCGAAGTGACAACAGATTTGCTTCAT

GGAATTGCTTGTTTGAATCCAATTAACTCATTTTTTAGTTTTGACGTCGGGAAGATAATG

AGAATGGCTGAATTATATCCTGATGATTTTGATGAATATAGTATGGTTAAGCTTGAGAGT

CAGCTTGCGAATTATATTGTTGATGTTCGTGATGTTGATGAAAGGTTCTCCAATTTAAAT

GGACTTTCTGATCTTTTAAGAACAATGATTGAGACAAAGAAAGGCTCAAGTTATCCTCTT

GTGTTTCGTTTAGTGAAACTTGCTTTACTTTTGCCAGTTGCCACTGCATCAGTTGAAAGA

GCTTTTTCGGCCATGAAGTTTATCAAGAATGACTTACGGAGTCGGATGAATGATGAATTA

TTAGGTGGTTGTTTGGTGCCTTATTTAGAAAAAGATATATTTTGTACTATTTCTAATGAT

GATATTATAAAAACATTTCAAGAAATGAAAGCTCGGCGAA

>XLOC_027282 transcript=TCONS_00044925

AAACTAGATGGCTTCTCCTACGCATATTTGCTGCGAAACAGGTATTTCGAAATTTGGAAA

CACGGTTCAACCAGATCCTGCTGCTGCTGCCCAGAAAGGTATGCGTACCGTTCGACTAGG

TCCTGGTGTTCCTGGTGATGAACTGGATATGGGTAAAGTTATGGCAAAGGCTCTTTTTTC

GGAATATGAAAAGACGCATCAACTAGATCCCGCTCGTCTTTGTGCCCATAAAGCTGTGGC

TGTGCTTGCCGCATATGTTGCTTCTCCCAGATATGAAATCGTGGTTCCATCAGATCCGGA

TGCTGCCCGAGAAGCTAAGGCTAGGGCTTTTGCAGAATATGCTCTTTCGTATTTTGTAAA

ATCTCTTCAACGACAACCTCCTACTGCTTTTCCTAAATCTCCACCACAGCCGGATCCTGC

TGCTGCTGCTGCTGCCCGTACTCAATGGGAAGACAAAATCCGAGGCTTTGAAAACCTTAT

TGTCATTGTCCAGTGCCCTGATTGTGAAAGGTTCTTGGACGAGGACGCTCGGCGTTGGAT

CAGAGCTGAATATAGATCTCTGTCGCTACTCGCTTTTTGTATGAGGAGGTTTAAGCTGAA

AAATAACTTGGATGGTCTGTTGTATCTGGGTCGTCCAGCCTTTACTCACGATTCCCGTGA

GTTGGGAGTTACCTTTGGCCTGCTAAAAACGTTCTCACCGGGAATTACTCTTCGACACAC

ACACATTGTTGACTTTGAAGTCCACTACCAATTCTGTGAGCACTGCTCTTCCATGTATGG

GCGCTCAGCTTGTTCAAGTGATGACAACTTGAAATCAGAGCCGCTTGACTATCCCCCCTT

CCCACCATTGCTACCCGCGCCCTCCTCTTCACTTGATATGTCAAGAAGAGTACAACCTAT

AGTGGATGATCACCTTTAGCACCTTCTTTATGTCGTTATCCGTTTGAAGACTATCTACCT

AATAGTATATATCTACTGATGTAGAGTGGATGAGGGAGTGAACTGAAGTTTTCCAAATGA

CTCATGAGTTGCTTGTCTTTAACTTTTAGTTCCAGTAATCAGTATATAAGTTGTCTTACG

TACAACGATCTAATGATGCATGCTTGAATCTGCGCCAAGTTGCCCTTGTTTGGTTGTTTT

TGAGATATTAACTGCAACCTCTGTGGATCCTCAATATCGTCCTCAATATATTGATCTGAT

AGCGACTTGGATTATTGATGAGAATATATTTTTCTTATGCTGATGTTTTTTAATTGGAAC

TGGACTTTGCATTCGAAGCTTGTCTTCATGATAAGTGACTCAGTTCTTGATGGTTTGGAA

AGAGGGCCGGGACGAACACCCGAGGGGTCGCATAAGGAGGATCCCTTGTTCCAGGTACAG

AAAGAGTACGGTAAATAGGAGAGTGGTCTTTCTCAAGCGCTTGTGGTCATTTGGGGGCTT

AGATCATCTACTTCTATGTGCTTCCAATTTGG

>XLOC_026844 transcript=TCONS_00044193

CTGCAACAAATAAGGCCTTTAGCGACAATTTATTAGCGCGCGGCAATAAGTTAAATGCCA

GTGATAACTTTCACAAGTTCAAGAATAGGCGGCAAATGTATAATTGCTGGTACTAAATTC

CCTTATAACGACAAATAAGAAGAAATGCTTATTAAATACCTCTAATTAAAGTTATTTTCT

CCTTCCCCTACCAAAATATCTCATTCTTACCCAAACTAATTTGAAAACGTTGGGAAAAAT

CACCTCTCATCTCTGAAATTGCCGCTGCCAAACCACCGCTCACCGCCGACGTTCACCGTC

TCTTCTCACTGACGCCAATCACTGCCGCCTCTCATGGTCGCTTATCACTGCTGCATCTCA

CCGTTTCCGTTCACTACCACATCTCACCACCGCATCTCACCATCACCGATCACCATTGCC

TCTCACCGTCGCCGGCCACAATCACTGAATAAGATTGGCGCAACAAGTACCTAATTGCAA

TTACATCGTGCTAGTCGATTACCTAAACGAAATTTTCTAAATCAGATGTTAAAGTCGCAG

GTTACAACTGCACAGTATATGGGAGCGCGAAAGAGCGTGTCACATTGTTAGTACATGAAA

TATGGTAAATAATATTTCAATTATTTCTATGCATTTGATAAAAAATATGAAGTTAGTATT

TTAGCTTCTTGGAATCATCATCTCCTAGATCTGCTTTTGTATTAATGCCTGCAAGCTGTA

AACCTTCGAATCTGTTTGATGTGGTTTAAAATCATATAATTTTTGGGCTATTTGCTCTTC

AATTTTGTTTGTGAGCTTAACTTCCTTCTATCTGCAATATGTTTTAAAGGACGCAGTGGA

GCATGATTAGTTGATAAGTTACTTGATAAACAAAGAAGATTTTATTCTTCATGTAATTTT

TCTTTCATTTATTTTTCAAACTTTGCTTCTCTAAAAACCCCAGTTGATCGATGGAAAATT

ATTTACTAACAAGAATCAGCGTGCTTCTTATATGATAATGAGGTGGAAACAGAGGTTATG

AGGCAGCAAGAATCCTGTTGGAGTACATACATATCAGCTCAAGTCTGAAGACAAGCAAAA

GGCCCACATGATATACAAACCTCCTTGACAGACCCAACTACGACTGGGCTGACCCGGAAG

ACTTATTTATTTTAGTTTCATTAGCTATTTTAGATGTAAAAATATATATAGGAATATTTC

AGTTTTTTTGTAAATAACAATACATATTTTTCCATTAATGAAGGAAGAAAGACCGACATG

GTCTAGTTCTCTCTCTGTCGAACTCTCTAGAATCTGGGATCATCCTTCACTTTCTTCCGC

TGAATTCATGGATTTTCTCCATGCTAACATGGTATCAGAGCCCGGAATCTGAACAATCCG

AGTCGGCTCTTCACTCTCACGGTGGAGTTTTCTTCATATCTCCAGCGATCGAGTAGTTCT

GGCATTTTTCTCGATCTGGTGACTGCAAAGTGAATTTTTCTCCGGCTGAGATTATCCGAC

GTTGTTCCGAAGGCTATTCCGACGATTTTCTCCGGTCAGTTTTCTGAAGATGATGACGAA

TCGTTGCTTCATCTCCGGTGACATCTGAACCCTAATTTTTTTTCTGATTTCTCTGCCTGT

ATTTGCATCGCTATACAAGTTATAGTTTTGCTGTTCCGCTTCACTGTTTGTTGCGAATCT

GCTAAGAAATTGATAAAGCCCTAAATTTTTGAATATTGATTTTTTGAACCCTCGTTTTGT

GAAATGGCTACTGCACCTAATATTACTAGTGCGTCAACCAATGCCTCTACGTCTTCATCT

AGATCCACAGCCATTAATACCATAGACATTTCTGATCCTCTCTATTTGCATGCGTCCGAG

TCCCCTGGTTTGATGTTAGTGTCAAAGGTTTTCAATGGAACTGGTTACGGAGGATGGAGA

CGGGGAATGCTCATAGGCTTATCTGCCAAGAACAAATTGGCAATGATTGATGGCTCCATT

CCGAAACCAGAGTCAAATTCTCCTTTGTATGGTAATTGGATTCGCTGCAATGATATAATA

ACAGCGTGGATTCTGAATGCTTTGGATGGGGATATCGCATACAGTGTTATGCACACTAGT

ACTGCCAAAGGGATATGGGGAGCTTGAGAAGCGCTATGGACAAGCAAATGGGACTAGAAT

TTATCAGGTTAAAAAGGAATTGGCCTCCGTTTCTCAGTGTGCCTCTAGCATTGCTTCTTA

TTTCAACAGAATTAAGAAGTTATGGGATAATTTGGCTGGAATGATCACGTATCCAAGTTG

TACTTGTGAATGTAAAGAGGCTTTCCATAAACTGGAAAAGGACCAAAGGGTTCATCAATT

CTTGATAGGCTTAAATGACTCCTACTCTACCATTCGATGCAATATTCTAATGATGAAGCC

ACTTCCAGATGTTGACACAACTTATTCTATGCTTATAAATGATGAGGACCAGTCTGAAAG

CCAAATGTCACCAGTCTCTTTTAATTCTGAATCTGCTTCTTTTTCTACTGGGGTTCAAAA

ACCTTATAGACAAAGATATCCTTCAAATCAAAACTCTTCTCCAAATCAGAATTACTCATC

AAATCAGAATTACTCATCAAATCAGAATTATGCCTCCAATCAGAAGTATCCCCAACACAA

CAACTACACTCAAAAGTATATGTATGGTACATCTAAGAAGTCTGACATGGTGTGTCGATA

TTGTAAGAGGCATGGTCACCTAATTGATGACTGTCGTAAACTCAAGAGAATTACAACTTG

TGCCCAAGTCCCTAAATCACAAGATAGAGGTATGTTAATGAAGGGGTTTCAACCAAGAAT

GTGCAGCCTAATATTGTCGCTCCTGGCTTTAGCAAAGAACAGATTGATACTCTCTTGACA

CTGATCCAGAAATGTCCAATGCCCTCTTCTCCAGAAATTGCTTCAGGACCTGCCAATTTT

GCAGGTTTGGTTGGTTGTCCTGATTATAATTTTAGTTCTATTGCTTGTAATTTATCAGAA

GCTGTAAGTAGTTCTTGGATAATTGACTCAGGTGCAACTAGTCATATGACACCTTACAAA

TCTTTACTTTTTGACATTAAACCACTTATTGTTCCTTATCTCATCAAACTTCCAAATGAC

TATAGGGTTAAGGTCACATGCACATGCTCTATATCCTTTTCCCCTACCATGTTGCTTCAA

AATGTTTTATTAGTTCCTTCTTTTCAGTATAATCTCATTTCTCTCCATCAGTTACTTAAG

CAATTACAATGTGATGCATATTTCACTGTCAATTCCTGTGTTTTGTAGGGCCATTCATTG

AAGAAGCCACTGGTTCTTGGTAAACTTCAGAATGGTCTGTACTTTTTCTACAATGAATAC

GGTCCTTCCCATCTGTCTCTGTACGTTTTGCCTGCAATCATGTAAATAACGCAAGTACCT

CTTTTGTTGATTCTCTCCCACAATTTGTAACCACTGTTAATACCACTTCAAATAATAAAA

CTGTGAGAGATGTTTTACCTGATGATTGTCTTGATTGTTCTTCTATGTCTGTGGATTATT

CTTGGCATCAAAGGTTAGGTCATATGCCTTTTGCAAAAATGCTTACTATTCCCTCCATCA

AAACTAAATTGCCTGCTAAGCAAACTTTTGTTTGCAATATTTGCCCAATGGCACGACAAC

AGAGACTTGCATTTCCAGAAAAGTCTCATCATTCTACTCACCCTTTTCAGCTAGTTCATG

TCGATCTTTGGGGTCCTTACCATACTTGCACTTACAATGGTTTCAGGTACTTCATTACTT

TGGTAGATGATTACACTAGAAGTAGCTGGACCCATTTACTTTCCACTAAGAGCAATGCTT

TCACTGTTCTCAAATCCTTTATTATTATGATTAAAACACAATTCAACACTACTATTAAAA

CAATCAGATTTGATAATGCTTTTAAACTAGGCTCTAGTTTAGAAGCTATATCTTTTTTCT

CCTCCCATGGCATTTTGCATCAGACTTCCTGTTCACACACACCACAACAAAATGGGGTTG

TGGAGAGAAAGCACAAGCACCTCTTAGAAACTTCTAGGGCCCTTTTATTTCAGTCAAAAC

TCCCACTCAAGTACTGGGGAGAATGTGTGCTCACTGCCACCTATCTTATCAATAGGTTTC

CTTCACCTTTATTGCAACATAAATCCCCGTATGAGTTGCTGCATGGTTCTGCTCCATCAT

ACACTCATCTGATATCATTTGGCTGCTTGTGTTATGCAGCAGTACTCAAACCTTATAGAG

ATAAATTCAAATCTAGGTCTATCTCTGCTGTTTTCATTGCGTATAGTCCTGGTAAGAAGG

CCTATAAACTTTTGAATCTCTCTACACAAAGTATTTTTCATTCTAGAGATGTCACTTTCC

ATGAGCACAAATTTCCTTATGCATCTAGCTCTACTACACCTGCCCTCTTTCCTTCCCTTT

CTTATCCACCTGATGATCCTATTTCTTCTCCAATCAACTCTCAACCTCCTAATACTTCCC

TTCCTCCCCTTCCTACTAGAAGATCCCTTAGGACTCCTAATCTTCCAGTCTAC

>XLOC_000525 transcript=TCONS_00000879

TTGGTATCAGAAGCACCATGTCCTCCTCTTCTCCTTTATCTTCCTCCATTCAAAATACCC

CCACCTCAATCATCTCTTGGTTTCCCAATTCCTCTTCCAACAGTGTCGAACATCAAAGGT

TTCATTTCAATTGAGTTAAACTATCTCAATTACCTAACATAGAGAAATGTCTTTTTAACT

ATTTTAAAAAGTCACAATCTTGTAAGCCTGATTGATGGCTCAATTCCATGTCCATCATCG

ACACATGAGGAATACAAATTGTGGATTCAATGTGACACTATTACTTTGAGTTGGATTAAT

GTTACTTTATCTCCTTCAGTTCTTGATACTCTTCTTAACTATGGTTGTGAGACATCCAAA

GAAGCTTGGGATACTCTTGCTTCACTCTACCTTGACCAAGTTTCTTCTTCTTCAATCAAC

TTGAAGTCCAAATTTCAGAATTTCAAGAAAGGTTCCCTATCCATGGAGGATTATTTACAG

CAACTTCATTCAATTGCTTGCTCTCTTCGAGCAATTGGAAAACCTGCAACTGAAGATGAT

CTAGTTACTCAAGCATTACAAGGTTTGCCTTCCTCTTACTGTATCTTTGTTTCTGGATTA

AATGCAATAGGTGCACTTCTTTTATTTATAGCATTGCGACCACTTTTGCTTACTGAAGAA

GCCCATATCAAGGAAGCCACTATAGATGACTCCACCTCACAAACAGCATTAACAGCATCA

ACTAACGCTCATGCTTCACACAATGCTCCTTCTTCACAGAATAATTCCTATTCTAGAGGA

CAAAATAATGGCCGAGGATGAGGCACGAACAATAAAGGTTACAATGGCAGGGGTTGTAGC

CAGCATAACCAGCATTCAAATCGCTCTTTTTCTCCTCAATGGGCTAGTTCTCCAGTATCT

TTTCGCCCCCTTCACCTATGGTTGGAGTATTAGGACGTCCGCCTTTGAATCCTTCTACTC

CATGCCAAATTTGTTTGCATTATAATCATACTGCCATTTACTGTCAAAATAGATTTAATC

ATTGTTATGCTCCTAATTTGCCACCGAAGTCCTTTGCAGCTATGAGTTTAGAAAAAGTCT

CTTCTACTGTTTGGTATCCCGACTCCGGAGCTTCAGCACATATGACTAATGATCCTACTA

TTCTTTCTTCCTCTACTTCCTACGGGGGGTCATCTTAAGTCATGATTGGTAATGGTAATC

TTCTCCCTATTTTTTCTACTGGTACTTCAACACTTTCCACTTTTTCTCGACCCCTAGTTT

TACGTAATGTCCTATATGTCCCCTCTCTGAAGAAAAATCTCATTTTTATTCAACCCCCTC

TGAAGAAAAATCTCCTTTCTATTCAACGACTTTGTACAGATAATGATTGTATTATTGAGT

TTACTAATACTAGTTTTTTTGTTAAGTACAAGAAGACCAGTCAAGTGTTGCTCCACTGTG

ATAATCTGGCCCCTTTATCCTCTTCAAGTAGCTATGCCTTCGTCCTCTCACATTGCTTTT

TCCGCTGATATTTCATCTTACAGTTTGGCATCGACGGCTGGGTCATCTTGGTCAATGATC

GCTTTTTGCTTTAGTTCATAGGCAGCTGATTTCTTATGTTTCGTCTTTTATAATAACTCT

TGTACTGTTTGTCATTTAGGAAAACAAACAAAATTGCAGTTTCCTCATTCATTTAGTCGT

GCAAATAAACTTTTTTTTCTAGTACATTCTGATGTATGGCAAGCTCCCCTACCCTCAAAT

TCTGGTTATCGTTATTACATTATTTTTCTTGATGATTACACTAAATTTACTTGGGTGTAT

TTGACGCATGAAAAGTCTGAGGCATTTGACAAGTTCAAGGAATTCAATATTATGATCACT

AATATTTTCTGCTCATCTATATCATATTTTCAATCAGACGGAGGAAAGGAATATGGCAAT

CATAAATTTCAGAGTTTCTTTAAGGACCATGGCATCCTTCATTGTTTTTCTTGTCCTTAT

ACCTCCGCCCAAAATGGACGAGCGGAAAGAAAACATCATCACAGTAGTAATTTTTTCTGA

TGTCTTCTATTTCAAGCGTCTATGCCATTTTCTTTTTAGGATGATGCTTGTCTTTTTGCT

ACTCAGTTAATTAACATTTCTCCTCTTCCAGTTCTTAACTTTATTTCTCCTTTTGAAATG

CTTTTTGGTCGTTCACCAGACTATAATCTTGTTAAGGTGTTCAGTTGTTTGTGTTTTCTT

TTTACTGCTCTTGGTCATACAAATAAATTTGAACCTCGTTCAATTCCACGTTTGTTTCTT

AGATGTTCGACCAAGAAGAAAGATTTCAAATATTTGGATCCTAATAGAAAGATTTCAAAT

ATTTGGATCCTAATACTAATAAAGTTATCATTTCACGACATATCAAATTTGTCGAAGATA

AATTTCCATTCTGTCAATGTTTCCTAGTAACTCCCAAACTCCTTTTTTGTTTAACGACTT

TGACTTCAAATAACCTAATTAAGATAATTTGAATTATAGCTTAAATAAGAATTCTTCCTC

TAGTCTTTTAAGTGATAAAGTACAGAGAGTTTCTTTGAACTCAAAGTTCACAGACTTTAA

TTCTTCTTCAACTCCTATTTTGCCTAGGACAGTAAATGAACAAGACTTATCCTCTTCCAA

TACTCAACACTTATCCAGAGCTTCCCCTGATTCTTTCATAAATACAACTTCAAATTCCAT

TAGGACTTCTTCCAAAATTCCTGTCTCGTGAAAATATTATCAGAAATATTCAGCGTCCTC

TCTCATCTAACATCTCTATATCTCCTGGCCGTATCTTAGGACAATCTCCTATATTATCTT

CACCATCACCTATTGCAGTATTCCCCCACACTCCTCTCCTATATTATCTTCACCATCACC

TACTGCAGTATCCCCTCACACTCCTAACTCAGATCCTCTTCCAGATTTATTTCCTCAACC

ACTTCACAGTCCCCCGCGTACTCACAAGATGATCACTAGAGCTATGAAGTGTTTACTTAA

ACCTCGTATACTTCCACCTCTAGCTGCTTCTTTTTCCTCTTCTCTATCTGAACCTACTTC

TTATGCCCAAGCTGCTAAGGATCCACGCTAGTAGCGTGCAATGAGCGATGAGTATAATGC

CCTTATTCAAAATAATACCTGGGCATTGGTTCCTCCTCATTCTAAAGCAAATATTATTGG

TAAATGGGTTTTTAGAATTGAATATTGTGCTAATGGAGCTATTGACAGGTTCAAAGCTAG

ATTAGTCGCTCTAAGGTTTTTTGCAGCAACAAAGGTTGGACTACAGCCAGACTTTTGCCC

CGGTGATCACAGTCCATCTTGTCATATCTCTAGCACTACAACAAGGCTGGTTCATGCATC

AACTTGATGTTTCAAATTCCTTTCTCCATGTTGAGCTTAACGAGGATAATTTCATGCGAC

AACCTAAAGGTTTTGAGAATCCAGATTATCCTACTCCTACATGTAAATTGAAGCGAGCTT

TATATGGTTTGAAACAATCTCCCAGAGCTTGGTTCCATCGGCTAGCTATATTTTTGTTAG

ATATTGGCTTTATACAGTCTACTTCTAATTATAGTATGTTCATCTTCCGGACTTACCATG

ATATCTTAGTTTTATTAATTTATGTGGATGACATTGTTGTTTCTGAATCTTCAAAGCAGC

TTATACATCAGTTTATCTATTCCATGCACTCTGAGTTCTCCATGAAGGATTTGGGTCCTT

TGAGATACTTTCTCGGTGTCGAATTATACCGAATCCTGATTGTCTTCTTTTGTTACAGCG

CAAGTATATTAATGATATTCTTGTGCGCTTTGATTTAGCTCAATCAAAAGTTGTGCTTAC

CCCGCTTCATAGTAAAATGGCTTGGAATTCAGTTGAGAGTCCTTTTCTGGATGATCCTTC

TCTTTATCGATATATGGATGGGAGTCTTGAATACTTATCTTTTACAAGGCCAGACATCTA

ATTTGCAGTTAATCTTGCTTCACAGTTTCTTCACAAGCCGTGCCAGATTCATCTCCAAGC

AGTCAAGCGTATTATTCGCACTAGCGAATATGGACTAAACCTTTATTCTGCTTCTTCTTT

GTCACTCATTGTCTACTCGGATTCTGACTGGGCTGGATGCACAGCTACTAGGCGATCTAC

CTCCGGGTTTTGTAATTTTCTTGGTGACAACATAATCTCTTGGGCTACAAAGAAACAACC

AACTGTAGCTAGATCAAGTACTGAAGCTGAGTATAGAACTCTGGCAACTACAACTGCAGA

GGCTACTTGGTTGCAGTTTCTTCTTCATGATCTCGGAGTTTATCTTAAAACTCCTGTATT

GGCGAGGTGATAATATTGGTGCTATACACTTGGCGCACAATCCTGTTTTTCATTCTCGTT

CTCGTTCTAAACATGTGGCTTTTGACTATCATTTCATTCGAGAGAAAGTGAAACAAGACA

ATCTCACTGTTTCTCATGTTCACACATCTCAGCAACTAGCTGACTTGTTCACCAAGGTCC

TTTCTTCTACAAGATTTAAAGACCTTCGCAGCAATCTTCATGTTCATTTCCCCAATCATA

TTGAGGGAGGGTGATAGAGATAGCTTTCAGTTATTTGTATTTATCCATTTACAGTGTTTT

AATGTACCACAATTACTATAGCATTTGTACTCTTATCTGTATAAAGACATGAGTTCTGTG

GCCTTAGCAGTTATGCTAGCCATCAAACATTCAATCTCATCTGCAAATCCAGCGGAACAA

AAATGGATTGTGGGGGTCATTTTCATGGTCCCTGTGTATGCAACTGCATCAGTAAGGACA

TAACCAGTGAAATGCACATTTATTGATTCACTCTCCCATATTTCTTAGGCATCATTGTCC

TCTTTGTTTAATTTACTGATGAGGTTTACATAATATTTTGGCCCGGTAATAAATATTACC

CCTTCTGCCCCACTTTGTTTGTCATATTTTACTTATTAGTCTGTTACAAAAAGATTGTCA

TATTTCTGAATTTAGAAACAATTTAACTCCAAACTTACTTTTTGACTCATTTATAGCCAC

ACAAATCTCTATGGCTCATTTTAGACCACAAGTTTCAAAAGTCTTCCTTTTTTTATTAAA

CTTTGTGTCAAGTCAAACTACGACAAACAAAATGGGATGGATGGAGTATTATTTTAATTT

CAGTTAATTTTCATGCTTATTGTAGTTATCTTTAGCACCTATAAATATCGATTTTGTAAT

GTTAACAC

>XLOC_021238 transcript=TCONS_00034914

AAATCACTAAACAATTGATTAAAAACAACTCAACTTGCTCTCAAGCCCCAAGTCTACCAT

TGGCCTGTTCAAAGTCTTCCTTGTGTCAAATGCAAGTGCTTAGCATGAGCCAAGAAAATA

CCTAGTAGGAATCTTGACAGGATCAAATAAACTGGTAATAAGGGGTGAAGAATAGATCAT

TGCTTTCTTGATCTGCCAAAGTTGTCAAAGGTACATGATCATTGTTGTAGCTTTCTGGTC

ATAATAGGAAGTACAAACCAATCATCCAGAGCTGTCAACTCCCCTCCATCTTGTGGCAAT

TCTGAAGAAAACAAATGTCTGAAATAGAGAGGAGATATCTTGATACATTTTTGTTTTGGG

CAACCCAAAAAGGAAGGATCAAGGATGGGAAGTTGCGCGGACATGGAATGGAAGTTGGAG

ATACTGTTTCAGGGCCAAGGGAAGGCTTATCTTCTGCTGAAGTCAAAGAAGGTTATGAGT

TTCTCCTTTCTCTAGATCCGGGAAGGTGGCCTTTACCAAATTTATGCAGTCCACTGTTTC

AGAAGTTCATTGATCACATACTTCAGAATGTGAAGGATCTTTTCAAATACAAGAAAGAGC

TGGACAAGTTTGATCTTCTAAAGAAAGAGTTTCTAGTCCTTGAAGGAAGTCTAAAATATT

TCAAGACCTTCCTCGCGTTCATAAAAGATGCAAGCATGGAACTAGAGAAATCCAGAAATC

TCTTTAATCATATGGAGGTTATCACCCGTAATGCTGCTTACATCTACTACCTATGTTATG

TGGACGAACTGAATGAAGAACTGGCATCTCGCTTGAGACTTAAGGTCTCTTCCGTTTTGA

GTGATATCAAGCCCATTGAGCCCCGAGTTGCAGCAATTTATACCTCACCTCTAAGATGTT

TGGTTAAATCACAAACCCATTACAACGGAGATGTGAAAGAATTTGTTAAAGGGTTCCTTC

ATTTTCTCTTGAAGGATCTGTCGGAGTTATTAGATGGTGAGGCGTGTTCTTTGATTTTTC

CTCTTACAAAGGATCAAATCCTTCAAGCATCAAAGCAGTTGGTAACTTTGCTCATTTACC

CGCCTGAAGATCTATCTGTCGACCAGGGGAAATTGAGTGATATACTTACTTGCACTGAAG

GTCTTATTGCTGAGATAGCATCTGCTGCTTACTCATTCTCTGTTGAAAAAGCAGCAGAAG

TTAATTTGGTTTTCTCTGACTTGTTAAAAAGGATTGGCCAAGTCATGAAAGACCTAGAAG

AAGTAGTTTTGAAAGCTCCACTGTCATTAACATGTAATTTCCCTAGGACAAATGGATTAG

GCTCTATTGACTCCATCACAAGCTATCTGAAAGAGCTGCAAGATGGAAAGTGTGATTCTG

CTGTCAAGCATCTTATTAAGGAAGCTCAGGAGGATTTAGCTCTCTTAAGATCCCTCTTGC

AGAAAGTTGTGAAGCGACAGCATGAGCTTGACGAGGTGAAGAAACCTGAGATTTGGACAA

AAGTCATATCTCTAGCACACGAGGCGGAACGCATAATCGATTGTCTGGTTATGGGAGATT

ATCCTGTTTGGTATCTGCATTTGTTATGTAAGATCACAACAGAAATAAAGCTTGTCAAGG

TTCAGGACGAGTTCCGCCAAATTATGAGTGTGCATGAAGTTGACCCGATCGAAGAACAAG

CACCAGATCCTGTTGAAAAAGAAGATGAACCTACAAAACTTGATGAGGAGGTAGTGGTGG

GTCTTGATGAAGAGGCAAACACGCTAATTGATCAGCTCACTAGAGGATCAAGGAATTTAG

ATATTGTGTCTATTGTAGGGATGCCTGGTCTTGGAAAGACTACACTGGCCAAAAAAGTTT

ATCGTGATAAAAAAATAAAGGATTATTTCGATGTCCATGCTACGTTTTGTGTTTCCCAAA

TGTATAACAGGAGAAAGTTGTTGCTCGATATTTTAAATCAAGTTAAAGGTTCTGAACAAA

AAACTGAGAAGAATCCAGCAGATGCTTTGCGGAAATTTTTGATGAGGAAGAGGTACCTTC

TCTACATTGATGATATATGGAGTGTTAATTCATGGGATGACCTGAGGGGATGTTTCCCAG

ATGATGGCAATGGAAGCAGGATATTATTAACCAGCCGTCTCATTGATGTGGCTATAGAAA

TTAACCCTGATAGAGTACCTTTTGAACTTCGTTTTCTTACTGAAAAGGAAAGTTGGGAAC

TGATGAGAATCAAGGTGTTCAAGGAACAACCTTGCCCAACAGACCTAACTAACATAGGAG

AAACCATAGCGAAAAAGTGTCAAGGACTGCCACTTCTGGTAATTTTGGTAGCTGGAGTGT

TGTCTGGGTTGAGCGAAACTGAGGAAAGTTGGAAAAAAATTGCAGACAGTATTAATTTAG

ATACTGTCACTAGTGCAAAAGAGTGCTTGGATGTCATAGAGCTGAGCTACAGTCACTTGC

CAGATCGTCTCAAACCCTGCCTTCTATATTTTGCATCATTTCGCGAGGACGAAGAAATTC

CACTCTCAAACTTGGCGTGGTTATGGACTATTGAGGGACTGGCTCCAAAATCAGAGGCAG

AGAGTGAAGAGCTTGTTGCAGAACGTTTACTGAAAGATCTCATTGGTAGAAGCTTGATAA

TGGTGAACAAGAAAAGGTCCACTGGAGGAGTTAGAACATGCCACATTCATGATATGCTGC

ATGATTTTTGTGTGACAAAGTCGAGGGAAGAGAAGTTTATGCAGATATCAGATACTGGGA

AAAAAGAATTACCTAGTTCCTTTTATGAGCACAGAAGGCTGTGCATTCAACATAGCCTTT

ATTGGGCTGGGAGGATCGGAAACTTGCAAGTTCGGTCTGCATTCTTCAGACCCCCACAAT

CTGGATGCCAAGAATTTTTTGATCTGGACAACTTTAAACTTCTAAGAGTTCTACAAATGG

AATGTCCTGTGTCAGATAGTTCATTCCAGAGATCAAAAGAGCTGATTCTGTTGAGGTACT

TAGGAATCAAGGGCTATATGGAATCTATGCCGTCATGGATATCAAACCTTTCAGACCTTG

AGGTATTGCTTGTAACAACAGTAGGCAGTGGTACAATATTGCCGATGACCATCTGGAGTA

TGCCAAGGTTAAAGCATGTGCATGTAGAACCTGTTGCATCTTTTGATGGACGCTTTCCTC

GCAAATCGACAGACATATGTCGCATAGAGACCTTAGCCACAGTTTCTCTCACTAATAAGC

TAGCAGACAACATGATCAAGAAGGCAACCGGACTGCGAAAGCTCAAGTGCCACTGTAAAG

AACCTCTCAGGCTCAGATTGGATGCTCTTCTTAAACTCGAGTCACTCAGTGTGAATGGTA

AGATCGTGAAATTCAGCTTGCCCTCGACGCTGAAAAAGCTTACTCTATCAGATGTTTTGT

TGGCACAAGATGAAATGTCTAAAATCGGGAAATTACCAAACTTAGTGGTTCTCAAATTGG

AGCATAGAGCATTTCAGGGGACACAATGGGTCATAGACAATGAAGATTTTCCGGAACTCA

AGGTTCTCAAATTGAGGTCCCTCAAAATTACAAAATGGATTGCCACTGGTGAATACTTGC

AGAAACTCGAAAGCCTTCTGGTGGAGAGCTGCTTTCATCTTCAAGAAATTCCTCCACCTG

TGGGAGAAATTCCTACGCTAAAGATGATTGAAGTGAAACGATGTGGCGAGTCTCTTGAAA

AGTCAGTCAATGCAATTAAGGAAGAGCAAGAAGACTATGGAAACGAGCAGCTCAAGTTCG

AAATTTATCACTTAGATACATCAGCTTGATTCAATGCTGTGGCTGATCTTAAGGAACCAG

TGATTCTCTTAATTTTTTTTCTTCTTTCATTATGCATAATAACATTTCCACCTAGATGGA

AGTTTTTTGTTTTTTTTTTTAAATGTGTGCCCTACTTCAAAGTAATTTCTTTGGATGTAT

CCATTGAATTGACATATGAAGTTCATTTCTTTAAATGAAATTTTGTACGATTGCACCTTA

TAATAGTTTGCGAAGAAATTACTCTCGTCACAGGAAGGGTGAGATAACTTCAGGCTGTTC

TTGCAATGTTTGTTCGATTTGCAGTAGCCATCAAATTTCAAGAAAGAACTCTGCAGAACT

ATTATTAGATCAGTGAGTGAAAAATTGCTCAAGAAAAATAGTACCTAGTTGACA

>XLOC_008468 transcript=TCONS_00013802

ACCATAATGGAAGCTTCTTCCAGTTCATTTGTTGGGTTAAAACCTAAATACGACGTGTTT

TTAAGTTTTAGAGGAGAAGACACCCGCAAAAACTTTGTGAGTCACCTCTATAGTGCTTTG

ACAAAAAAAGGAGTTCACACATTTAGAGATGATAGGAGTTTAGAGAAGGGAAAAACAATT

AAAGATGAACTCTTTGAAGCTATTGAAGAATCACAATTGTCCGTGATTGTCCTGTCCAAG

AACTATGCGTCGTCTGGATGGTGTTTGGAGGAACTCGTGAAGATCATTGAATGCAAGATC

CATAGAAAGCAAATTGTTATACCCATCTTGTATGGTGTGGATGAGACAGTAAAGTGCAGC

ATAATTGATAGGTTTGCTGAGGCCTTTGCCAAACGTGAAGAAGCAGATTCTAAGGATGGT

TACTATGTTGATCAAGAGGAGAGGGTGAAAAGTTGGAGGAAAGCTCTCACTGAAGCAATA

AATGAATCAGGGTGGGATATCAGCCAGGGTGTCCAGCAGTACGGGAATGAAGCGGATTGT

ATAGAGCAGATAGTGAAGGAGATTGTTGGCAAGCTAAGAGACCA

>XLOC_001800 transcript=TCONS_00002889

CGCAAATTTCATTTCCCCCGATCTCCTTTTCCCCAAAATTCAACCAGTCTCTATGTACCA

ACTCTGATTGGCAAATCGCCTCTTTGACCTTAAACCACTAACCCATGGTCCACAACTCCT

TTCCTATTACTAATACCTTCCCTTATGGTGACAAGACTGGGATTTAATGGATAAGAAGAT

CGAAAATTCTATGTTTGTAGATAACATGCATTGGCTCGATTCCACTATAGTCGTAAGCTT

CAATAGCCAGCAAGTTCAAACTTAGAGGGGATATCAAGAGCAACTCAATTACCACTTAGT

TCATGAAGTATTACAAGACTACATTCGAGGAGAAGGGATGTCATTGATGAGAAGCCAGAA

GTTATCTTACCAGATCCCAACTTTTCAAAAACTGGTTTTTCCTTTATGGAAAGGAAAATC

TTTTAGCGTATTGAACATTCTGATGTTAGAGATATAGCTCAATTTATCTAATAGAATGCA

AGACTCGATACAGGGAGAAAGCTCTTCAACAAAGGAAATAAGTTGGCCA

>XLOC_022811 transcript=TCONS_00037505

AGTTGATTTAAGAACTCTTGAAAACGTGACTGGATCATCTATGTCGTGATTCTCACCACT

GAGCTGTTGTAGAATAACATTAGTACAAGAACCTGTATCCACACATGCATTTTTTATGGC

ATATTACTGCCATGCTTTTCTTCCTAAAATTTCGTGACAACATGGAAGTATATGCCTCTA

AGTGATACATATTTATCATTTCCTATAGTTGCATTATTAATGTCAAAGCTGTGGATCACC

TCCTTCTCACCAACATCTTAAAGTTACATTCTATGTCTACCACTTCATGAATGTTAATTT

TGCAGGACTTCAAGTTGATTGTGCTATTTGCGGACTGCTATGCTCTGTGTTTGCTGGATA

GAATTCTTCAAGTCCTGTAGGATAAACTTGGGTTTTTCCTGAAGATGGTGGGATATTAGC

AAGCTTTCTTAGATTATTGATATATTGACAATCAGATAGCAGTTGTGAACTCAAATATTG

GTGCATGCTACCTTTCTCATGATTGAGCGTATCGTTTGTGGTCTCCGAAGGTTTTCTGAG

TGCACATTGTACTTGCAAGGAGTAGTATCCACATAAAAAGCAACATTACTTGCTCTGGAA

TTTCCTTTGAGCTAATCGCACGCTGAAACTTTTGTGAAGGACTGTCGGAACGTAGGTTGT

CAGCTGAAATGACAACTTTGAAAATAAATGGAAGTGGTGACATTATTACTGTACCTGAGA

AAGAGTGCCCAAAGAATGAAACATCTTCCAGGAACTCTTCCAAACGAGATTTGCCAAACA

GTACAAGGGGACAGTCATCTTGTATTTTAACTTCCAAAAAAGGTGCTAAAAATGTCTATA

CTGCAACATGTATCATTAGTGATGAGTCTTCAGCTGTTGTCTTTGATGGAAGTTTGAGGA

AATTTGAGGGCTCTAACTCTAGGAAACCTTTAGGTTGTAATCCTTCAGAACAGCACACAT

CGGCAAAGAGTGATATCAGCTCAGAGGTGGAACTGCCCTTTGTTGGAGACAAAAAAGAAC

TCGATACCAGGAGGGCGCTTGAGAAGAACCTAAATTCTAGCGAAAATGGATTAAGTTCCA

TAACACATGATGCACAAACAGATTCTTCATGTTCTAACTCATATGTAACTCATCCAGTTT

GCCAGGAAACATCAGCAGAAGCCAAGACATTCATGCAACAGAATCTCAGTTCAAGTGCTT

CTGCACCGATTAGTTTCATTTCTAGTATGGAGTCATCAGTTATGGAAAAATCTACATGCT

CCAAACGACAACAGAACATCTTTTACACGGCTGGATCATGCACTAGGGGCACCTAATAAT

GGTGCAAGAGAAGTCAGGCAACATCTTAATAATCGTCCTGATAAAGTATGTGGAGTTCTT

GATTTTTCTGGTAACCGTAGTGAAAACAACTGCGCAGCTGCGAAAAAGAAAGAAAGCCTT

TCATGCATTCAATCTGACAGCTTCGATAATTATTTTACCAAGGGGGAAGAAGTTTTGCAT

CGCCAATCCTCAACAAGCGAAAATAGATCCTTGTTGACAAGTCAGCAGGAATCTGAAGAG

ATGGATCCTGGAAAAACACTTAATGGCAGCCTGAAATCACCTGCTTGTTCAGCAAAGTGT

GAAATTGAGCAAAAGACTGCTGTAGCCACTATACTCAATCTGAATGAGGATATCCGCCAA

TATGATGATCCGAAGAAATCTGATGGTGAAAGTATACCTTCTATCAAGGCGATACGTGTG

GTGGCTAAATCTGGAGTTCCTCTACCCTTGTCTATGACTATGAGTTCATTGAAATTCGAA

GGCAAGCTAGGTTGGAGGGGTGCTGCTGCTACAAGTGCTTTTCGCCCTGCTTCTTTTCCC

AGGGGCTCAGATTGGAATACTTCTTCCATGTGTAAGGATGACCTGATCTCAAATGACACG

AGAAGAGTCGTGGAATTTGACCTTAATGTTGCTGCTACAGAAGAATTTCCTCCAGTTGAA

TTGCCAAGTGAAAATTGGATAACATCGGGAGGTTCAACTTTTAAAAGTTCTTCAAATTAT

AGTTCAAAACAAGCAGAAAAGTTTAACTTTGACCTCAACTTGCTTGGGGATAATGCTGAT

GAGTGTCCTCAACCTTCTTTTCCCATGAAATCAGAAAGGTTCTCATCACTTAGCCTGAAT

TTGAATGAAGATCCATGGCTTGGAGGAGCAAAGAGTGGTGTTCATCCACATATTCAGAAT

GGGGAACTCATCGGGAAGAAGAACGTAGAATCTGCAGCCTGTCCAGTCTTTTCCAAAGAC

CAAGACCTCAGTTTTGTTAGATTAGGTAGCAGGGGAGGCATGAATTCGTCCCATAACTTC

AGCCATTATCTTGGCCAGCCATTTCTAGTGGCTACACCTAATATGCAGCACCCAGTGGAA

CACATCCAGAGAATGGTGCCACTGCAGTCAAAGTTACCATACGCGGTCCAGATGTTACCA

TCTCAGAGTTATCCCTCCAATGGTCCCTTCTATATTGGCCCTGTCAGTTCCATGCCCTCC

AGCAACTACTATGCTAGTATTGTTCCATGCTCAAGGGATACACGTGAAGGGGTCATTTTC

CCTCAGGTACTCAATAGCAGTGCAGTATCAGCCTTTCCTGGGGCTCCATATCTTGTAAAT

GTTCCCCAAGGTCAAAGCCCAAGTGGTGTTATGGAAACTCGAACCAGATTTGATGTTGAT

GGTGGAGAAGTTTATTTGATGAATGGTATCAGGAAGGATAATGCCCGGCAATTTTTTCCT

GTACAAAGTACAGAAGAGCAAATGAAAGTTCATCAGCAACAACCTTTACGTCCCATGCCC

ATGAAAAGTCACCAGCTACAACCTTTACATCCTATGCCCATGAAAAGGAGGGAACCAGAG

GGGGGATGTGATTCTTTCCAGCCTGGTTATAAACATACAACTTCAACCCGCTAAAGTTCA

GTGGCTAGAGCAAAGCTGTCGTATCTGTAACTCTCATATGTCAATCTGAGTCACTCATAG

GTTGGATTTCTCAGTAGGTCTATATTGCCCCAGTTTTTTGTAAAGGCACTACAAAGTAGG

TATCACTTATGCTTTGATAAACTTCTCAGATACGTTTGCAATTGGATTTTCTTAGTTTTA

TTGTACATGAGACAGTATCATTCTTGAAGCTATGGAATTGATGGTGATACAAGCAACATA

ATGTAAAATCTTTACTGCCATCCTGATCTCCAGACCGTTTATGTGCTATTTAGTTTCATG

GTCTTCTTGTTGATGGCATTCAAAAGCAGTTCTTTAAATACTTTAGTTAGCATTCCTTTC

TGTTACCTTGTAGTAAGGCAATCATTGTTGCTGGTTATGTTACTATGTATATTCAAGAAA

TCATATCTTTTTATCGGACATACTGGATATGTTGTTGTTGC

>XLOC_034984 transcript=TCONS_00057512

GTTCTTTTCCTAAGACTTCAGCTACTCCAGGTATGTTGTGCTTATAAAGCTTTGAAATTG

AAATAAGATGTTTGACATCATACTTTGAATCCCATTTTATGATCCTGGAAAGTTGAAATG

AAGAGGTCTTTTCCTCTTAGTTGTATGGAGGCTGTTATGGTTGCTTTCAGGCAGGGGTTT

GCTTAGGGGAAATGTGAGAAGTTGAGGTGTGTGTGCAGGATGAGAAGTGTAGG

>XLOC_000500 transcript=TCONS_00000844

TTTTGACACAACTACGTTCATCTCCCTACAAAACCTGCATTTAATGCTTTCATTGCCCTT

TTCCTACTACATATATGCCCAAAAAATCCATTGCCAATCCCACAACTCTAGCTATACCTT

TTCTATTTACAAGTATTCTTTGAAGAAAACTGATAATACCCTATTGATCATAAGCTTGCA

AAAGCAAAAATTCTCAATATAATGTCTAAGTTTCTTGTTTTTCTTTTGGTTTGTCTTTCT

CTCCATGCATGCAGCACTGCCAGACCACTTGCAACCACTGACGAAGAAAATAGAATACTA

ATCTCTGCCAAGGATGTACTAACATTTTTGACAAGGGACTTACTTAAAACACAAGGAAAA

ATTACGTTAGAAACAGATGGAGGTAGTAGAAATGGTGATGATGCAAACTTTATGCGACAA

AAGAGATCAAATAGTGTCTTACAAAAGGAAGTTGAAGATGAAAATAAAGGACAAGAGGTC

AATATATCGTTTGTAAAGGAAGCTCAAATGAAGATGTTGAGGAGACAATCAAGGTTGATT

CTTGAATCTGCACCATCTGCACAACATGAGGAAGAAGCAGTGAGCTCAATTGAAAAAGGC

CCTGTGGAAGACGTGGTGGTTATGGACTATGCACAACCCCATAGAAAACCACCCATCCAC

AACAGAGAACACTAGCTAGATATGTATATCATAGTAGCTAGAATATGAAAATATACTTAT

GTATTTGATTCCTCATTTCCATGTGAGACTCTTTGTTTTCAGTTTTATTTTCTTCGTTTA

TTGTTTTCACAAATGAAGGGAGAATCCTAGATAAGAATACTCCTATACGATATATGTACA

AATAGCAAAAACCCTAGCAAGAAAATATCAATTGTGTTATTGTCACGTGTTATACATTAA

TGCAGTGATTGAATGGGCAAGAAGAGGCTGAGGTTTAATTTAGGGAATGATGTGGATGGA

TATGGTCCCTCATATTATTGTGTTGTTTTTGTCAATTGTGCTTGATATAAAAAGTGCATG

TAAATATATTAGTATTAGTAGAAAAAGAGTAGCAGTGGAGTGGAGTGGTGGT

>XLOC_017113 transcript=TCONS_00028078

GGAAAGTCGATAGATAGTGTCATATCTTATGTCAAGAAATCTTGAAATATGTTCTTTACT

AGGATCCTTTTAAATTTATTTACTTATTAAGAAAGGTACACGCGTCAAACGCGTACCGAG

AACTAATACTAGTGTATTAAAGAAGAAACCATAATTTTTATAGCAATTAAAAAGAAAGCA

GAGGGAGGTGGGGTGGGTGGGGGTATACCAATTACTCTATGTAAAATTGCAGTAAGAGAC

GGCTTTTCAGTTGTTCACTTTGCAATAATAATTGGGTTGCTTGGAACCGATAATGGCAAA

AGAGAGTAAACCAGTTGATTTCCTAGGCAAAGAAAAAGCATCTGATATAGATGAAGACTA

TGAGTTTTTCCTGGAGACTCTCAAATGTAAGAATAGGGGCATGAAAGGCTCGTTAAGAGA

TGATTACAGTTTTGAATATGAAGCAGCCGAAGATGACGTTGAAATCCTTGACAACAATAG

CGATACACTCGAGAATGTAGGAAGTTTTGACCTTACAGCTTCTCCAGCTAAACACAGCGA

GAGAGTCGAGGATAGATATCCCCAGATGAGTGTGCAGTCTGAGTTCAGGCAGAAAGTTAT

GGATCTTCTGAAGAAGCCTTACAATCAAAATGAGTATGAAGAACTATGGGGGGCAGTCAA

ATGTAAAAAACGAATGCTTAGCAATATGGAGACGCGTAATGGGGGAGCAAAGTTTTACGA

GTCACAGAAATTGGGAAAATCATATCTGGATCACCATGATGAGCTTAGAAAGAAACTTAA

AAAAGTCGGTAATGACAAGGGGAAAAAGTTGAAGATCTTGCGTGGCTTTTTCTTTTGGCT

ACAGCATCTCACCCAGGCAGGTGCATTCAAACCTTGGATAGACACTGAGTTTCTTGCACT

GGTGGCTGAATCTAGCTAGAAAGTGATGAATTTCCAGTCCTACCTTTTCCGATTGATCAT

ATGATCTACCCACAAAGGCATGTCTATCAGTGAAGTATGTCTTGGTGAAGATTGAAATCA

TGCAGTGTAATTGGTAGGTAGTCCATATATCATACAGGGAAGCAAACCTAGCAGTGGATC

ATATTTGAATGCATGGCTTTGGTACATTCTTGTGATCTTCGTCCTAGCAAACCCCTGAAA

ATTTGAGGATTTGATGTCAATGTAAACAATTTTTTGCATCTTCATCCATTTTTATAGATT

ATGTTAAATAGATTTACCGCCATGCTGTTCTTGGCCTTTATTGGATCCCTATTTCTTTTG

TAAAGTGCAAAGGAGGGATGGTAGGACCAGGTTTGTATGGTCAAGTTTCATTCAAATTAG

TTGCTCCATAGTCGTTTTGACTGGCTGCATTTACTTAAATGATAAGTAG

>XLOC_007097 transcript=TCONS_00011543

ACTTATTCAAATGTATCACTAGTGCCATGAAGGCACCAACAAATAATGAGGCCTAGTTCT

AATCTCTAACAGGACATTAAACTTATTAGTGAACTGCTTAGTAACATGATTCAAGAAATA

GTTTAAATAGGTGGTGCACCAGAAGCCTGAAGCAAATACAACCGACAACATGGTGTTCCA

TCATCTGGATGAGTTTGTAGTGACCGTGGCAAGCTACGCAACCTGTTAAACAAATTTAGT

GGATCGTGCAATAGCCCAAAATGGGCATCTGGATCACCAATTTGAGAAAGTTTAACTTGA

GAAAATCCTAGTGATGGCAATAACTGGTCAGGCCAATCACTATAGAAATGGAAAATAGAG

CTGCACATAGTGGTTGATTGTTTGTTCATGAAATCTGCTAATAGTACTGTATGTGCTGAG

GCACAGTTACTAGCAATAACATTGAGTACTTCCATTGCATGAGAATGTGATAGATAGTAG

AGAATGCCCTCTAATACCCATACTGTATTCTTCTTTAACTCTAAGCCTGATAGTTGAAGC

TTTTCAAGCCAATCATTTTCTCTTAAATCAGCTGCTACTCTCCTCAAGGATTTTGCAATC

ATCATTGGTTGTTTTTGTTTATTTGTTGCCTCTGTTGCTTCCTTAAGAACAGTAGCTTTC

ATCTGCAAGACCTCCGGGAAATCAACCTCGAAAACATCACTTTCTTTCAAGCAACTCAAG

CGGTATGCCCTTGTATCCATACCTGCAGCAAGAAGGACAACTTGTGTGTCTCCACCATCA

AAGGAACTAAGAGCTGCTTCAAGTTGTTTATCAAACCATAATGTACGAATTGCAATCATA

ACACCAGAAACCTCTCTTGCATTATTGACACAATCCCTCTTAATTTTTTCATACAAAC

>XLOC_003391 transcript=TCONS_00005501

CTTACAGAGTGAAGGCGACCAGCCTTAGTTCATCTCACAGGGATTCTATTACATCAAAAA

GGGAGTCTATATCCTCTAGAGTTTCCTGCTTGCACCAAAAGTAAAACAACAATAAACAGT

TCATCTTGATTTTCTGGATAGAGTCGCATAGATTTTCGAAGCATCTTGAGATCCTCTCTT

TCCAAATTGTCCACCAGATACTAGCCGGGACAATTTTCCATCTCTCTTTATGGCTTGACT

GTCTTCCATCACTGTTCCATCATTCAAGGACTTCAGTTACTTTCCTTGGCATAACCCAAC

TAATACCCCTCAAATTAATGAATATCCTCCATAACTGAACAGTCACGTCGCAGTGCAAAA

AAAGATGGTTGATTGTCTCAGCTTCCTTCCCACAAAGATGCAATCTTAAGGATGACATAT

TACTGGTGCAATTTCATGCCGCTTTCCAGAGGAACTTCTGTTGTCAGGTTTATAGTTTTG

CTTGGACTACTCCTTGCTGCTAATATGGAGTTCACAGGAAGCATTCCTAAAGGTCTGCAG

GTGGATTGGGATCCATCCTGGAGTTTGACCCCAATTCTTTTGTAGATTCTGTAAAGAGAT

GGTTGTACCCATCTCTCAAAGTCGGCACATCCTGCAAAAATTATCCAGATGTAACATCAA

CATTTGAGACGACTGGATCTGCTGTTGCTTCTCTGAGCTCCTATTCAGACTGAAGGAGAT

ATGTCATTTATTGTTCTGATTACAGTAATACAAGGATCCTGGGTTCAGTGTTTAAGTGTG

CTTCATGGCGTGTGTTACATGCACTCTTGTATGTGGCAATCTTGGCTGTTTGGAGCTCTG

CAGCAAGAGTGCTTTCTCCTAAAGGTGTCAATTTTGAAGGTGTAATGGCCATATAAATTA

AAAGCTGCTTTGAAGGATCCTTGTGAGGTTCTTGATAATTGGGATGACACCTCTGATGAT

CCATGTAGTTGGGTTATGGTCACTTGCTCTGCTGAGAGTTCTGTTATTGGCCTGGTGTCT

TCTGCAGAGGGTCTCCCAGCCAGAATCTATCTGGTTCACTTTCTCCAAGCAATGACAATT

TAACAGATCTTCTGATTATATTGCTTCAGAATAACAACATAACAGGATCAATACCAAAAC

AGACTGGTTCAGACTCTTGATCTTTCAGATCACTTCTTCACTGGTGATATGCCTCCTTGG

GACACTTGAATAGCCTCAAGTAAATGAGGCTCAATAATAAGAGTCTACCAGGACAAATTC

CAGTCTCATTGGCCAACACGCCACAGCCCACTCTTGTGTAAATCTTCCAGGCCACAGACC

AAAATTGTCTGAAATCGTCTAAATGCTTGAAGTTGATAGAACTTGCAGACCGGTGGCAAA

CATCCCAAAAAGTAGATGGTGATAAGTAAAAAACACGAACAACAGATCATCATCATTTGA

TTGCTTGTACAAGCCATAATGCTATCCGGTCCTAGGTGAGCTCATTGTATTCTTCAATAC

GTACCAGTACAGGTTCTCCATAAGGTGAAATACACACTTAGAACTGTCAAGTAGGGGTGG

GCATTCGGAATTTCGGTTCGGGTTCGGTTTTTATAAATTCAGAATTTCGGTTTTTGGTAC

CTAATTGTCGATACCAAATACCAAACCAAAATTTATTCGGTTCGGTTCGGAATTATGTAA

TTCGGTTCGGTTTTTTCGGTACGGTTTCGGTTTTATAATTCGGTTTTTATTTCAATTTTA

TGTCGTTCTATTAATAGCATTTTTGCAAAACCAAATTTTGTAGTAAGTGGCGTAGTGTCA

CTGTACCAACTCCTAATGTTGTTCAACCTTAATAAGCTTTCAACGTGCTTTTAAGGACCA

CAACCCACAACATGATAG

>XLOC_016496 transcript=TCONS_00027043

ATCCCACTTACCAAAAACCCTAAAACACCACACCATCTTCCCAAATACATCACACCAGCT

GCCACCCTTGTCTCTTCTCTCCCCCTTTCCCCTAATTTCAATCCAAATACACCCCCCCTC

TCCGAAACCACCCCTTCACCGCCACTAAATACGGTGACCCTACATCTCTCTCGCTTTCCT

CTATCACGTTCACACAAGAAAAATGAAAAGGAGAATCTTCTCTCCTCATTGTTACATGAA

CCCGAAAAGGGAAGCAAGAAATTAGGCACAAACCTTTGCAAATCTGAAGCAGATCGAAAT

GATTTTGATACAAAGCAAAACAGAAAATCCGGGTGTGAATCCTCACAAATCAAGCAAAAA

CGGGACGGACCCAACCCGAATTTCACGTCTCAGACCCGACCCGACACGAGAAACGGGGAA

TATATGGAGACATGACAAAGTGAATAAATATTGATTTAAGTCTTAAGGACTTGAAATAGA

AGCAGGCTGTTCGTTTAAGGACTTGAAATAGAAGCAGGCTGTTCACAAAGTGTATACAGG

GAACATTTCTTCCTTTTATTCTAAGTATACATGTATCATCCCTTAGACCCGTTATTGTAT

TTGTTAGGATCCTCCTTTGGTTTGTATGCTTAAGCACCATAAGAAGCATTAGTTGCTTAG

GATAGATTGTTATGAGTATGAGTTGCTGCTGCATGCAGCGAATTGGGCTAACAGGGGCCA

TTTCTGACCCATTCTTTATTTGTTGTATGTCGGAAAGGTTGACATCACGAGACAGGTTGA

AAATATGACTATATATGCAAGGTTAAACATCAAACTGCTGGTCTGTTTATGTGTAGCAGA

ATCTGAAATTTTAGGTGGAAAAGGACAGGTCTTAAAATTGGATTATAGTAGGGG

>XLOC_032290 transcript=TCONS_00053066

CTATACTTCTAAGCAAATATGGTCTGGACACACTCCAAGGTACGGGATCTAAAAAAAATG

GACAGAGTAGTGGTTCTGGAGAAAATTTACGCAAAAGAAGGGCAGGTGCAGTGGAGTCTC

GAGCAGCTCAATTGCCCAACATAATCCCATCACGACACCTCATGTCTCAAATCAGTCCAC

TTCGTGCAAATGAATGTTCCTTGCCTCAACGTAGTTCTGAAGTCACTCGGCCTGAGCTTC

ATAACATCAACTCTCAACCTAATCAGTCACATACCTTAAATGAAGTCTCTGAACCTCTAA

TTACTCCACCAGCCAATATATTTGTAACGTATGAATTGCACAATTAAACTCACTGTTGTT

ACGATAGAAACAATATAAATTAATAAAATGAGCTCTTAGAGGAACTAGAAAGTGTATTCT

TTTTCAAGTCATTAACTGTTTCTCTAATATTTAATTAACATTTCATTGATTGCAGATGTC

ACGTAATGGATCCAAAAAAAAGGGCAGACTAATGGTTCAGGAGAAAATTCACGCAAAAGA

AAGGCAGCTGCAATGGAGTCTCGAGCAGCTCAATTGCCCAACATAATCCCATCACAACAC

CTCATGTCTCAAATCAGTCCACTTCATGCAAATGAGTCTTCCTTGCCTCAACGTAGTTCT

GAAGTCACTCGACCTGAGCTTCATAACATTAATACTCAACCTCAATATAATCATTCCCAG

ACCATAAATGAAGACTCTGGACCACAAGTTACTCCACCGGCAACTATGACTCCAGCCACC

TCGGCTAGTGCAATTGGCGATGGTGATGGCGATAAACGATTATGGATTGAACCTGACGAG

GATGGGTTTAAGCCTCACAAACCAGCTATTGAATGCATCGCTAGTTGCATCCGTAGCAAA

TTTGAGTTGGCTCGACCTTCTTGGAAGAAGTTTCCAGATTCTGTTCGTCTTTTCTGGTTT

GAAGAATTCAAGAAGAAATTTAGGTAGTTGCCTTGCTATGGTGACGCTATATGGCGTAAT

TTTCAGAAAAGAGGATCTGCAAGAATGAGTCAGCTATTCCTAAATGTTAGAAAAGATTTG

ACCATCAGACCAGAATGGATGGGAGATGCTGTATTCAAGGAGATGAAGGAGATTTGGGAG

TCTCCTGAGTTTAAGTCAAAATCTGAGCAAAACAAAAAGAATCGCGATTCAAAGGCAGGT

GCCTCACTTCATACCGGTGG

>XLOC_021023 transcript=TCONS_00034516

AAAATCCCCAAAACAGCTCAAGACCTAAATCATTTCAACCCATTTTCTTCCCCAAAATTA

ACCTGATTATCCGCGCCTTACCCCAATTCCACAAAATTTAGAACCCGAAATCCAAACCTC

ATTCAAAATCGATCGATCCCACAACCCTCTCCAGCTAACAACCAGTACGAAGATCGAATC

ACCAGCGTTCATTAGTATTTTTTCCACTCTTGAACGACTTGGATAAATCCAACGGTCCTT

TTCAAGAACGTTTAAAAGAGATTGAAGTTTTGACTGAAAATCAGCGTTGAAAATAGATCT

TCAGGTAATTTTACCTGATTTTCCCTTCTTCTTTTTTGTTGATTTTTTCTCAAAAGCTTT

CATAAATTCAAATGGAACCTTATCCAAAAAGATCTGCTAAGGGTATTTTAGTATTTTGAG

GCATGGGTTAGAACCCTAATTTCACTATATAAATACTCATTTATACGCAGCTTTGATAGA

GGGAGACATACAAGGAAACTGGCATAAGATAGAGATATACGCATAAGTTCTTTTTTTTAG

TCTTTTATTTCTCATAACACAGCGTGTTACTGTTTTCTGTGGATTGTTTTCATGTTATCG

CTTCATCATTCGTGTCGTCGACCTCGTTCGAGTCGTCAACCTTATTTGAGTCGATTACTT

CGGATCCAACGCCTCGTTCACTGCCTTAAGAAAGGTTTGGGATTGAACGCTGGAGTATTG

AGATTAAAGATATTTTCTAGGCTGTAAACTTCCCAGGTGGTCTAGTTTCTTCTCTGTCTA

AGTTCTTTTTTAACTCTTTTAGCTTCTCTTCATGTCCAGCTTTTGCTTATTCTGCCTTGA

AGTATCTGACAAATAGTATTCCCTTTTAGCTCTGCTTTAAATGTCTGCTTAGTTCTTATC

TTGGTATTTGTTTCATGTTTTTTTTCTCTTTCTAAAATGGTTGGTCGGGCTAAATTGTGT

GTTGTCCAGATTTGAAAACTCTCAAAGATAGGCCAAATTAACTCGTTTTCAATCTGTATA

ATGGCCTTTACTGGGGAGTTAAGGCTTGATTCTAAATTGGTGAAGTAGCCTGGTGGTATT

TTAAAGAAAGCAGCATTTTGTAGTATTTCTTAAGAATTTTTAATTAAGTCATTAGTTTCA

GCTTTTCTTCTTTTGAATTATATTCAGTCCATTAGCGTAAGTTCTGCCTAGAATCTGCTC

TAATCGTGCATGTTCTTCCCTAGGTGATCCGCCAAGCTTTGGGTTATTGTATCCAATCAT

TTATGGTCCAATAGGTTGAAGTGGAATTGCTGCTTTAAGTTTGAGAATGAGTCTGGTTAA

ATTGATGTGTGAAGCTTTGGCAGTAGGCCTATTAATTTTAGATGATTGCTGTCTAGAACT

TCCCTTAAGTGTAGTCTAGTTTTGGGCCTGGTGATTGTATTTGGCCTTGGTTTAAGATCT

AGAATAGAGAAGAATATAGTCATGATGTTGAGTTTTGCCTTAGAGCTTTGGCAAGCTGTT

AAGAGATGTGATGTTTGCTTCAGTTTTCTTTTTAGTTTTGGTCCTATATCTTCTTGAGTT

GTTTATGGCTTCTTACTGCTTTGTCTTGGGCATTAGCCATCTTCTGGATCTTCTCTGGTT

GTCCTCTGTTTAGTTTATATCATGTGTCTTGGTGTGTTCCATTTAGATCCGTTAGTCAAA

GTATTAGTAGTAGTAGTAGTAGTAGTAGTATCTTTGGTTAAATGTAGAGATTTGTATGAA

TGTCTCTGTTACCTTATACCTCTGCATTTTCCATGTTAAATTGGTCTTAAAATCATATGT

CGTTTTGCATTCAAGTGCTTGTTCATGTGAATTTTTCTTTCACTACTTGTTTAGGGATCT

ATTATAGAATGTGGGCTAATTCATTTTTGTTTTTTTTATTTTTTTGGCATGAAAACTTGG

GACGAGTTTGGTTAAAACTTGACATTGGAATCAGCTAATTGGCATTGGAGTTGTTCGCGT

CGGCATTGCATTCCGGGCTCAAGTTTTTTAAGAAATTGAAACTTTAGAAAATCCAAGGCT

CGTTTACGAGTTTGCAAATCCTGGGTTCTAGTTTCTTAACCCTCTTTTTCCCCATTCCTT

AGAAGACGCATGTTATAGAACTTTCTCGTAGACGGGACTAGCAGCCATGTAGGATAGAAT

TATAATTGAACTAATTAGAACTGTTTTGTGATTTCTTTTATTCACTTTCTTTGGAATCTG

TTAGTTAGTTTGTGAATTTTAAGAACCATTTCTTAAATAATGAGTGGAAGAAAAATACAA

TTAAATTGTCCCTCT

>XLOC_013090 transcript=TCONS_00021449

GTATGAAAACATATTTGTTTTCTAAAGAAAGTTTTCTAACACACCATTTATCTTTCCCTC

CAATTCTATTCTAGATAATTTATTATTTCACCCAATTAATAAGAAAGGAATAAAGAAAAA

ACCTACTCCCACGCAGTCGACTTGGGTTTAAACCTCCCTTACGAACACCCTTCTCAATTT

AAGGGTTTACGGGCTATTGCATTAGAAACATAGTTTACCCGTGGATTTCCTTATTTATAA

AAAAAAAACATATTCCCTCCAAAATAACGCCGATTGTCAAAAAATAAGCGCCACTGCTCG

ACGTTGCTACCACCATCATCGTTTCCCACAATTCTCAGGTAACCTAACGCATAAGTGTCC

GATGGCAAGGCTACATTGAGGGTCTCACTTAAGCATTCTGGATCTATTTGTATGTATGCT

GGCCCTGCAGGTGGAGCATATGCCAAGAATAGCTTTGGGAATATATTGAGAAGATGTGCA

GAAGAACAAAAGCATCTGCTATTCCAGTTATACCTGATTCAGAAGATCTTCATAGTTCCT

CTTCAGTTTCTGTTAAGCTGCTTCTCGTGCAGGGTAAATGCTGGGGAAATTTGTGTTATT

GACAATTCTTGTTTCTTGCTAAGCGAAAAATCATGTTCAATTCAGGTGGAAGTATGAGGC

AAATGCTTCCCACCTATGACACTGACTTTGCAGTTATCTTGTCTGTTTAATTTAGAGTGG

CGAACTCTCTACTATCTTGTTATATGTTTTTCTTTCATGGAGATGAGGCAGATATTTCCC

ACCTGCACTCCTGACTTCGCAGTTGCGTCAGCTATTTAACTTAGACTGGTGAAGCTCTGT

ATCATCTTGTTTTATGCTTTTGTTTCTTTGAGAAAAATTGTAGACTAATATGCTTCATGG

TGCTTGCACAAAATGATGTTCTGCATGCCTTTATTTTTGGTCAGTGTATTTGTAATGGTA

GTAAGAGAATGCTTTTTTGTGAAGTTCAATTGTTTCTCTAGGGTGATTCTCATCAGCGTG

TGCACAAAATGGGTCTTAGTTACCGATTGGCTAATCATGCAGGAATGGAAAGCAATCCAT

TCTCTGTTGATGGACTTGCGGTTTTTATATTCCGAGTACTTTACCGAGTTGATCATCCGG

TATTTAATCTTCACGTTACTCCATCCTTGTAAGCTGCTCTTCCCCATTTACCCCATTCAA

CTCGGATATTGATGCCTTAGATCTCCTTGCCGGCTTGGTCCTGAAGGGAAAACTTGACAA

GTCATCTCCAAATGCTGGATTTGTGCTACTAAAGTTTTATTACTTTTACGAGGGAAAGGG

TCGTCAAGAGTTCGAGGGTGAGCTCATTGAACGTTTTGGCTCACTTGTCAGAATGCCTCT

ACTGAGACTTGAAAGGTTAGTAAGATCTCTTTCTTCTGATCCCCTTTAATTGTGATTAAC

CTCTTCAGCTTGAAGTGAGTGTGCGCTCATTATGGATTCAGGTGGACTTTTTCCTACCCC

TTTGTTAAATTAGCTGGTTATGTTAGATCGGAGGCTGTTACAGCAGTCACATGTGTTGTA

TTAGTGTACCACGCAGGACAAATATCATAAATATCGCGTTTGACATCTGTAATACATGTT

TTTTAATGCTTCAAGTCATTACTATATCTCTCCTTTCCACTTCTTTTACACTATTGCGAT

CTCATATGCTATGTTTCTCCTTATATCTATCTCCTGTACCTAAGGTTGGTGATTTCTTGA

AGGATAAAAGCATAGTGAGCTGCATTCAGAAGGCTCATCTGACCCTGGCTCATAAGAAGT

CACAGTGACACTGCAGTTACCAATTATCGCGATAAAGTAGGTTTTAGAGCCGTAGCCCCG

GTGGCTATTTTGAAACCGCGACTTAAGCAGCAGTACTCAGAATAAAATAAAGTGTTTACT

ACCAGTGCCTCCATATATATTAGTTCTGCCATTCCTGTCGGGTGTTTGGCTTTTGACTAA

ACTGTATATACCCAATGTTTCTGTTTTTGCTACACTGTTAAGTTTATCAATTATGCGTGT

TCCAGTTGGTGCAAAAACTGTATGTGATGATTGTTTCTCCTAACAGTATTGTGATGAGTT

GATATTGTAGTTCCAGATTCATTTTTCAGTGTCCTGCACTGCTATCTGTGGTAAGGACAA

GTCATAGTAGTGTCTGATAAGGATAGACGTCCCTAGTCTATCCAGGTTCCAAGTTTGGAT

TATAGATTATAAGATGTATTTATCTTTGTTAGAGTCCATGTACAACTTATACTCTATCAC

TTAGCATCCTTGTACTTGTATATAAACAGCAGAATGTACTCAATAAAGAACAAGCCTTTT

TACAAATATTCCTCTACTCTTATATGGTATCAAGAGCCCAAAATTAACTCACACGAGTTA

TTATTCCTTTTTTTTCTTCTCTCCGTCCAAAAATGTCTGACTCCTTCTCGCCCATTGTTC

CAGCCACCCACGGCAGCATCGTCCAACCCTCTCAACTCATCACTTTTAATCCTGCCTCCC

AATTGTCTTTCAAACTACAGGGGAGTTCGAACTACTCTACTTGGAAATCCCAAGTCACTA

CTCTTCTCTTTGGCTATGATCTTCTTGGCTATGTCGATGGCACATCCTCTCCTCCACCAA

CTCACATTCAAGATCAATCCAAGAACGACATCCCTAACCCTACATATCGACTTTGGCAAA

GGCAAGACAGCCTAGTTCGCAATGCTATCATGGCATCGGTTGATCCCACTATAGCGCCTC

TAATCGCACACGCATCTACAGCAAAACATGCCTGGGAGATACTTCAAACAACCTATGCTA

GTAAATCTCACTCCCGCATATTTAGCCTTCGTGATACACTTGCCAATATCAAGAGAGATT

CACGCTCCATCAGTGATTACATGAAGGAAATCAAATCGATCTCCGATGATCTTGCATCCA

GTGGTTCCCCACTTTCTAATGAAGAACTTGTTATCAAAGTTCTAAGTGGTCTAGGACCTG

ATTACAAGGAACTTTCAGCAGCTATTCGGGCCAGAGATAATCCTATTTCTTTTGAAGAAC

TTTATGACAAATTATTGGCACATGAAATATTTATCCAACATTTCGAGCCAAAAGTTGAAA

CCCCGATAGTCACTGCCCAGTTTCATCAAAAATCCAACAACTCTAACTCGAAGTCTCGAC

ACTCTAATACTTTCAATCGTCGGGGTCCTTCACAATCCAATCCCACACCCTACCGTAACA

ATTCTTTCACAAGCCCAAACTTCTTCACTCAATCTGCCAACAGCCGACACACTCAGCACA

GGGTTCAATGCCAGTTGTGTGACAAATTTGGCCACATTGCGAAAGTTTGACGCTCAAGAT

CCCACAATGCACTTGAAGCTCAAGCGAACTTTGCCAATCGCACATCTTATGCTTCTCCCC

ATTCCAACAATTGGGTTGTTGACTCTGGAGCTTCGCACCATATTACCAACAACTCGCAAT

CTCTACAATCTTCCACCGAGTTTCCGGGCACTGATGAAATAATTGTTGGTGATGGTAAAT

CAATTCCTATTACACACATAGGGCACACAACACTTTCCTCACCCCACAACTCATTTAAAT

TACATAATGTTCTCTGCTCTCCTCACATTAAGAAAAGATTAATTTCTGTTGCTCAATTTT

GTCGCCAAAATCTAACCTCTATTGAATTTTTTCCTCATTCTTTTCTTGTGAAGGATTTGA

ACACAGGAACATGTCTTCTGCAAGGTCGGAGTAAGGGTGATCTATATGAGTGGCCGACTA

CACCAACCAGTTCATCAACTACTTCACCACAGGCTCAGTTTGTGACACAAACACCTCCAT

CTCTCCTTCTTTGGCATGCTCGTCTTGGTCATCCTCAACCTCGTATTACCAAAGCTTGTG

TGTCTTCATTTCAGCTTCCAGTGTCGAATAAAGAGTCATTTAGTTTTTGCAATTCGTGTC

TATGTAATAAAAGTCACCGTTTGCCATTTGGTGACAATTCTATATCTAGTGCCAAGCCGT

TTGATGTTGTCTATTCTGATGTTTGGGGTCCATCCCCTGTTACTTCCTTTGATAATTTTC

GCTTCTATGTGGTCTTTGTTGATCACTTTACTAAGTATACATGG

>XLOC_017635 transcript=TCONS_00028871

GACTAATAGTTGTACGTAATAGTGATAAATACTTGTATAGTTGTTACGGTTGTGTTTGAT

CGGAATTGGGGCCGTGGGTTGAGAAGTATTTTCTTCTAATGCAAATAAATCCTGAAGCTA

GTCTTGGGATTTCAGACGAGAACTCTGTGGCAGCTCCTCTACCAATTAACAAGAAACGTA

AGAGAATGAATAGACGTGGTGCAAATGCTGATAAATAGTCGGATGATGAAACAATGATCT

TCATACATGTCCTAAATGATGATGCGATGAGGCACGGGTTTAATGGTTCTACTGTCAGTG

GATCACGCCAGAAGGAGGTTATTAAAGATTTTTGGACGAGAACCAACAAAAAAGATCAGT

TTAGTGAAGAGCGCATTAAGTCCAAATATCAAAGATTAATGGCAGACACTAAAGCCTTCA

AGGATCTCCTTAACTCTTCCTCAGGGTACGGGTGGGACCCAGACATAGACACCGTTACCT

GCCCACCACAAGTATGGGATGCTCATATCAAGCAAAAGAAAGGTATTAACAAGTTCAGAC

ACCGTGAGCTTCCACATAATTGAATGAGATATTCGGAAAGTCATTTGCCACTGGGAACCA

TATTGTATACAGTACAAATCCAGTTTCCCCACGACGATCAAATGACCCTACTACTATGGA

GAATGAAGCTACTGATCGGGATGATGAAAATGTATATCGTCCTATGCCTGTGAATACATG

TAGTGGTAGTGGAAAGCGGACAATGTGTGATTTATCTATGGGGGATGGCAGTGGCACATC

GACACGCCGCAAAAAAAAAAAAAAAAAAAAACAGTCTACTGCTCTAGTTGGCTCACATGC

TTATCTAGAAAAACAGTTCCAGTGGAAGAGAGAGAGGGAGGAAGCAAGAGATAGAAAGAT

GGAGGAACAAGCACTAGCTCAAGCTACTGAAAGTGATATCTACTCTGAGGCAGCCGTGAT

TGTTCAAGTGCAAAATATTGTGAAAAATTATGGCCTGCCAAAAAATAAAGTTTCAATGGC

AATGTCTCACGTTAAAGAAGAAAGTGTTTGCAAGTTGTTCATTAGTTTGACAGAAGAATT

TCAAGTCCAATGGGTCGAGGACTTGCAACCTATTTGAGCTAATATTTATTTTTATTTGGT

TATGTTGTACTTTATTGAGTGAAACACTTACGCTATTGTTCATTTGGTTATGTTGTACTC

TATTTAGTGAATGTTTACTGTATTTTCATTAATTATGTTGGAGTTTATTTAGTGAGATGC

ATTAGTTATTTGCATAATGATCTTATATGTATCAATTTATATTATGCATTTCTTTTTAGT

GAAAGCTTTTTGCATCTTCATCCTATAATGCTTCATTTGTTCTTTATCTTTTTATTGTAG

AACTAACACTTTATTATTTTTTCTTATAGGTTGTAATGAACATATTCAGTAGTGGCAGTG

ATTCAGACAGTGATATTTAGTTGTACCTCATAGCTTGTGTAATGGATGAGGATGATGAAG

AGGTCAATCAGCAACGTAGGTTATTATCTCANTAATGAACATATTCAGTAGTGGCAGTGA

TTCAGACAGTGATATTTAGTTGTACCTCATAGCTTGTGTAATGGATGAGGATGATGAAGA

GGTTAATCAGCAACGTAGGTTATTATCTCGCCAACCGCAACATACATCTAGGCCTGGAAT

GACATGGGTTTGTGAGCTTATCAGTGGGCATCGCTTACGTATGTATGAAGCTATGCGTTT

TTATCCTGAAACATTTAACCGGCTTATTGAAGTGATAAGACAAAATAATCTGTTACCAGC

CGAGGGTCAAACCACACATGTTCCAATCGAGGAATCTGTGGCTATTTTCTTAACTGTTGT

TGCACGTAATGATAGTCAGCGCTCAACAAGTGAAACTTTTCAATATTCACTCGAGACAAT

TAACCGACATGTTAGAAGAGTTGCCAAGGCAATTAGTCAGATGGAACCAACAGTTATATG

TCCAAAAAATATGACCGGCGTACATCCAAAGATTCGACACAATACACGCTTCTGGCCTTG

GTTTAAGGACTGCATTGGTGCAATTGATGGAACATATATACAAGGGGAAGTCTCAAGTGA

TAAACAAGCATATCGTAACCGCAAGGGGAATACTTCACAGAATATATTATGTGCATGTGA

TTTTGATATGCGGTTCACCTTCATTGCAGCTGGGTGGGAAGACACTGCGCATGACAGTAG

AGTGCTTGACAGTGCACTTGCTGACCCGGCGGCAAATTTTCCAGTTCCACTACAGGGTAT

AATTAATATAAATTCGAATTCTTGTCGATTTGGTATGTTTGGACATTTTAATGAGTTAAA

TTTATTTTATTTTGGTAGATATGTATTACATTGTTGATTTTATTTTATTTTATTTTAATG

AGTTAGATATGTATTACATTGTATTACATTGTTGAAACACAAAAGGATTTTTAGCTCCTT

ATAAAGGAATACGATATCATTTGCAAGAATATAGAGGAAGTGAAAGTGAGCCTAGGAAAG

CCAAAGAGCTGTTCAACTATAGGCACTCCTCTCTGCGCAATGTTATTGAAAGAACATTTG

GATGCTGGAAAAGTAGATTCAGAATACTAAAGCAAGGGATGAACAATATGAATTGGGGAC

ACAGGTGAAAATTGTGATAGCTTGTGCGGTGTTACATAATTTCTTGCGTGAGCATCAAAG

CAGTGATGGTATTTTCACAGAATATGAAGATGATGATATGGTTGTTGATGGATCAGACGA

ACAACCAACTCAAAGTAGCAATGTTGCTTCATCGTCAAGGTCGACTGATCGGGAAATGCA

TGATCGACGTGAAGGATTAGTTCATACAATGTGGGAAGATTATGTTAAAGATTAGCTAGT

TATCATTGTAATGAATTTGGTTTGGACTAATGCATCTTTGTAATATTTTTAAATTTTAAT

ATTACATTAGCTTAATTATATTTAAAATTGGATACTTGCACAAGCATGTTGTACGGTAGT

GGCTTTCCTTTTTCTTCTGTTTTATTTTTGTTGTTTTGTTTTAAACGAGCCGAAGTGTTT

GAGGTGAGAGGAAATGTAGCAAGGTGTAATTCAAGTGTGTATTCAAGG

>XLOC_018058 transcript=TCONS_00029567

ATGGCATCTCAGGCCCTGTAGACAATTTCGAGGCACCTCTTGAGAGGTCTGAAATTTTGA

TTGGGAGATTGTCAAAGCACTTACGGAAAGAATTATTAAAGATCGTTTGTTAACAGCTTG

CGCCAAGACGCCCATGAAGACAAAAGCACTTCTACTGGATTATGCGATGCTATTAAAGGA

GCAGACAACTGTATATGATCAAACAAGAAAAATTGCCAGAATGTTGCCTAAGCCATTGAT

TGACTATGTCTGTGAAGTGTTGACACCTCAAGATGCTTTGCAGCAATCATACCTAGACAA

AATGATCAGGAAGTTCAAGGTGCCAAATCCAAAGACAAAAGGGCCATGTCAATAATCAAG

TGACTCACAACGTGGAAATTGATACTCTTAAGGTGATCGCGGACATATCGACTGAATGTC

TTGCCTCGAACCTTGAGGACAAGGTTCTCATTAAGGATGGGGGAATTGTTATGAACTCGT

TGGAGGACACAGATGGAACATTACTGATTTATGTGTGGGACCCGGGCGGAGGTGAATTAT

GTAAATTAGGCACACTAGGTTATTGGGGAACTAGTTATTATATATATATAGTACTTTGTC

AGTTGAAGGATTAGGCTATTGTTGTTAAGACTCTTATCTTCCTATCTCAGCTCCTAATCC

CCCTTCCTCTGTATCTGTTCTTCTTTATCATTTCTGTCTGTCTCCTCACCCCTTTCTCTT

ATCTGTATCTGCTCCTTATTCCCTTCTACTTCCTCTATTACACTTGCTTAACTTCTGTAA

TTACCTATCCCTTTTATCAAT

>XLOC_027928 transcript=TCONS_00045934

TTCCAGACTTAAAAAAGACTTCGGGGCCCAACCTTGACAAGAAAGCTGTAAGAAATTTCG

CATATGAGTTTAATCAGATAACAGCGTTGACGATGATGAAGTGAGTTATGTTTGATTGTA

GCTGCTTGACGGAGTTATGACTGAGAAGAAGCTTTGCTACTAGCTGAAGCACAGTAAAAG

TACTCCGGTTACCTGGTCGGCCTCGATCGAATAATTATTCATTTCAGCTCTTTGATTATC

ATTTTTGATGAAGATGTACTTCTTTAGTGGAGTCTGCGATTGGATTCAGCAAGTTGTGAT

GGTAAGAACCAAACTCGCCCAGTAAGGCAATCATATTTTTATACTTAGTTTCTTTGCTAA

ATTACTATGGCGTCTACTTCCTACCCACCGCCACCAGCTACGTCCATACCTCAGATCCGC

AATTATCAAGAACCGGGATGCCCCAACCTTGGGAAGCTTCATCGCTTTGGACCTTTATAC

TGCAATGGTGAGGATCAGGACCTTTTGAGCTTGAGAGATAATGCTTGGCAACACGTTCAA

TTGATTACAGGAGGCTGTTCATTGGAAACAGAGGAGAATTATCTGACCGCGGTGGGTGGA

CGACTTGAGCAAGCAGCAAAAAATGCTTATCCTAGAAACACTCCTGAGGTTACCAATCTA

GAGGGTGGAGTTTTCCGCCGTATGATGGTAAGACAGGGTTGCTTCTTTCTCATTGCAATA

TTGTTTATTCTAGGGGCTGATGCTGAGCAGCTTAAATATTCTGGCGATAACTCCATATTA

GGCACCAATCTCAATGACACTGACTTGAAAAAGAGGCGCAAGTTATTCTTCAAGTCCATG

ATTATTCCTGGCAATCAAATACCCCTTATCGTGCTACATAAGCTGATGGAACAAAGTTTC

TTCAGAGATATAGTAGCAGCAAAGACAAAATGGGAGAAGCCTGACCAAGATTTTCTCCTG

TCTGTCTTGTATGATTTTGTTTTGGTCCCTGCATTGAGGAGGACACGTGACACCCAACAA

AAAGGTTGGATGAATTGGTTGTTTTTTGTTCAACAGTTTTCTTCTAGCAATATTTCAAGA

ACTACAACTTTGGAGCAACCTGTTGATATACTTCAGTGCCTCTATCAACTGGTAGTAGGG

CCTGGAGGAGGAGGAGGAGCTGAGGATGACACAGTTGGACATGTGTTGGAAGATCTTGAA

GATGATCTGGAATATGGTACTAATGATATTGGTGAGGAGGATCAAGAAACCCAAAGGATC

CCAAGTGCCACTAGTTTGAGTCAAGCAGGTATCCGGTTTAAGAGTATAAAGGGTCTAGGA

ATTAGACAAATTCACCTAAAACGTTACATGTGTGGTGCTACTCTTTGCTTGCCCACCTTT

AATGAGAATACTCTTTTCATGTTGCGGTTTCTAGAAGATTATGAGAACATCCAAGGACTC

GCGAAACGCGAGATAAGTGCTTATCTGCGATTTCTTAGCGAAATTGTTCGTACGATACAT

GATGCTACCCTCCTTGATTCTGGAGGTGTGATTCGAATTGAACCTCGGCAGCTCAATAGG

TTGCCGTGCCTTTTGAAAGGACTTGTACGCCGCACTGCGTCCAATGGAAATTTTCGACAT

ATTACTAGTGCCAACCTTCAAATTCTTAAAGTCCATATCACACAATACTCCCAGAGGCCA

AGATACCGCAAATATTTTGGTTTAATTGGCATTCTAACTTTGATTGGGGTTATTTTGACT

TTGATTGGGGTTATTTTTGCTGTACTTCAGTACTTTAAGAAGGATAGTCATTAAAGTGCT

GACATAGGGAATGCAAGGTTGAAAAGGAAATGCAATCCATGAATTCAGGATTTTAACCAT

CTTGAGGTCTTGAGAGAAATTGAATTTCAGGTTTATCTGGATGCGATGAAGCTAGCTCCT

TTACACGAAGTTCCTGGACAACTTTGCAGGTCTTATACTCCATTACATTTAATATTTTCA

CAAGTATTAGGAAAGATGTGGAAGCTAATAGCAATAGTCGAAGAACAGCTAGAACTTTTC

TCTAGAAAAATGTAATTGGAAGAGCTTTCTAGACAAGGCTGCAATTACATTTAGCTTCTT

TTTTTTTTTTCCTCCTAGACTCCTAGTAAACATTGCAGAATTTGAGTTACGTTTTTGAGC

CAGTGCTGATTTGCTCAGAGTTAGCCCGATGTAGAGTTATAAACTGTTGTACATTACTTG

ACATGTTCGAAGTCGTTGTTAATAAGTTCGATTTGTTGATGAAACATTAATTACCGTAA

>XLOC_023112 transcript=TCONS_00037997

TAGGTATTCCGCGACACACCAAGTTATTGAGATCTTGCATCGCATGAAGGAAGTTCTTGG

ATGATTTGACTTTTGGCTCTTTGTAGTTTAATCTAGACATGTTCTTTTCTCGTTTATATA

CTTTTCTTGGTGAAATAGGAGGTGGGAATCTGATCAGATTAGTTTACTTATATATCAAGA

ATTCCAAATTCAAGTACCCGTGTCTTTCACGACTTTAGAGTTGTCCCCTGTCCCCACCTT

GTTTATCTTTCAAACTACATCCTGAATATTGACGGGTGTAGTTCTTAAACATTTTTCTCA

TTTATGAAGGATTTGTAATACTAAAAAGTTAATCATGTTGAAAAGTAGCTCAAACAGTGT

ATGTATTGTATTGTATTTGATACACTTCAGCTTTTCTGTGCGATATATTTCGTCCTTCCA

ACTCCACCTCACTAAACTTGTTCCTATCATACTTCACTTCTCATTCTGATACTTTCTGCT

AACCTGTACATCATCTTCTTGTTAGCTAGACATGATTGTAGCTAAATGTATGCTGGTTCT

CTCATTAATAACAACTCTCTTTTCTTCTCAATCCGCTAGGTGTACAGCTTCTAATGAGAT

ATTTCCACCCACTTCCACTCCAAGCCAAACTCCGGCGTTGGAGAATAACCCCTGGAAGGA

GGGGCAAAAGTGCTGCCCTAAAACTCAAGATGTGAACGTCCAGATTAAGAGGAGATCACG

CTATATACCAGCTGTTATTGGTGGGGGTGCTCGTCGAAGATCAAGATCATCATCCGTTAT

GGGAAAACAATTATCTTCAGTTCATGTTTTTTCACCTCTGTTTTTTCTCCTTTCACTTTG

CTTCTTGTTTTAGCAGCCATGATTTAGCATGGATATCATTAACACCGATGTTGCTTGCGA

CCCAATAAAAGCAAACAAAAAAAAAAAGGAAAAAGGAAAAGAGAAAATAGTCAAAAGAGG

CAGTTAGTGCTATCTTATTAGGACGATATAAAATATGTTTTCTTTTGGTGTTTCCTTTTT

AATCTACTATTGTATCTTTGGCCTTGAGCTTGAGGATTGTTCCTGTACCTTTCCTTTTGC

TTGAGGATAAAAGGTGGCTTTATTCTAGGCTTTATGTTGCTTTTTTACGTGTAAGTTGCA

TCACTTGCTAGAACTTTTCTCTGTGTTAGCAAATGTTATTATAAGATGCTTCCTTAAATT

GCCTCTGCTTTTAGTTTTCGGTAGCACCACCAGGTTTTCAACAAGGTGAAAATACTCCAA

TGTAAATTGTTCCTGTGGTTTGGGAAACATGCATAAGTAGATGTTATTTCTAAGTTGTTT

ATCGAATTAGGGGCTTTTGTGGAAGACCTATGTGGGGCATCTTTTGGGTAGCACACAGAC

CTCGCAGGTCCACGTTTTGAAATATTTGACCACTGGGGGGAGCCAAGAAGGGA

>XLOC_019493 transcript=TCONS_00031951

ATTTAGGAAACTTGCAGCAATTACAACTGGATCACAACTACATCGTGGGTTCAATACCGC

ACACCATATCCAACTTATCATCTCTATGGAAACTTAATTTCAACACCAACAATTTAACAG

GTGCTATACCCACAGAGATTGGGAAGCTTCACAAATTGGAGATACTTGCTTTGCAGCGTA

ATAAATTAAGTGGTTCCATACCGGAAGGGCTCTTCAATATATCTACGCTAAAAGGGATGT

CACTTGCTTACAATGACCTTTCGGGTAGTCTTCCATCTGCTTCAAACTGCTGGCGAACAA

ACCTACAATTTTTGTATCTTCTTGTAAACAATATTGGTGGAATTATACCCAGCTCAATCT

CCAATTCTTCAAATCTAAATGAATTATATCTTAGTGATAACAAATTCATTGGCTCAATTC

CTACCTCGTTGGGGGATTTGAGACAGCTTGAAATTTTGGACTTGTCCGACAATAACTTAT

TGTCTCCACATCTAAGTATCTTCGCTTCCTTGGAAAACTGCAGATCTTTGAGAATTGTAG

CATTATATAATAATCCTCTGAATGGTGGCCTTACAGATTCCATTGGTAATCTCTCCACTT

CTCTTGAACAGTTTTATTTATATGGTTCTGAAATTAGGGGCAAGATACCATTAGGAATTG

GGAATTTAAGCAACTTAAACACTTTGTCCCTATTCGGCAATGACCTGACTGGATCAGTGC

CAAGAACATTATGTGATTTGCAGAATCTTCAAGTTTTTGCTCTTCAAAAGAATAGGCTAA

GTGGACACTTGCTGGAGTGCCTTTGCAGATTGTCGGAGTTGGGAGGGGTTGATTTGTCAT

ATAATCAAATTTCGGGTCCCATACCGTATTGTATTGGCAATGTTACCTCTTTGAGATATT

TATATCTAAATTCAAATAGGATTGTCACCAACATACCTATGAGTCTATGGAGCCTCAAAT

ACCTATTGGAGCTTTCCTTATCGAATAACTCTTTGGTTGGCTCTTTACCTCTTGATTTCG

GAAATCTGAAGGTCATAACAGCCATAGATCTGTCAAGGAATAAACTTTCAGGAAATATTC

CAGCCACTGCTGGAGATTTGCAGAATTTGCTTTATCTTTCTTTGGCTTATAATGAGTTAC

AAGGATCTATTCCTGAGTCACTTGGGAAAATGATAGGTTTGGAAACATTGGATCTATCTA

ATAACATTCTTTCCGGTGTGATTCCAAAATCATTAGAGGCACTTATATATCTGAAGGATT

TTAATGTGTCGTTCAATAGATTAGAAGGTGAAATCCCAAGTAAAGGACCGTTTTTCATTT

TCACCTCTCAATCTTTTATTGGAAATGAAGAGTTATGTGGCGGTTCACTCTTCCTACCTT

GTGAGGCTATTCATCAGTCAAGGAGAAGCAAAGTTCTTCTGATTATACTAGTTACATTGG

CGATATCATTGATGACACTTGGTTCAATCGTTGTGTTCATGTTAAAGAGACGGTGGAATA

GAAATGTTCCAACTCAAGTTGAATCCTTACCCGCAACAATAACGCCAGCCAGAGTTTCAT

ACATTGAAATCGAAAGGGCAACTCAAGGGTTCAACCAATGCAACTTGCTAGGCTCTGGAG

GTTTTGGTTCTGTATACAGGGGCAGGTTTGCAGATGGGGTGGTTTTGGCCATCAAAGTAT

TTAGCTTACAGTTTGAAGGTGCATTATTGAGTTTTGATCGCGAATGTGAGATTTTGCGCA

ACCTTCGCCATAGAAATCTCAATAAAGTTATCAGCAGTTGTTCAAATATGGATTTCAAAG

CGTTACTACTAGAGTACATGCCCAATGGAAGCTTGGAGAAATGGTTGTACTCTGATAACT

TTTTCTTGGATATAATCCAGAGATTGGACGTAATGATTGATGTTGCGTCTGCTTTGGAAT

ATTTCCACTATGGTTACTCGACAGTTGTTGTTCATTGTGACTTGAAGCCCAGTAATGTCT

TGCTAGATGAAAGGTTGGTTGGACATGTGAGTGACTTTGGTCTAGCAAAGCTATTGGGAG

AAGGGGTAGCTATAGCTCACACTAAAACTCTTGCGACAATGGGTTACATTGCACCAGAGT

ATGGATCAGCAGGATTAGTTTCAACAAGTTGTGATGTGTACAGCTATGGCATTATGCTCA

TGGAAACTTTCACAAGAAAAAGACCACTGGATGACATGTTTCAAGAAAATTTTAGTATGA

GGAGTTGGGTCTGTATTTCACTTGCATCGGCAGTGGAGGATATTGTTGATTCCACCTTAT

TAGAACCAGAAGAGACTCGTTTTAACAAAATTTTGCATTGTGCAACCTCTATTTTAGAGT

TGGCATTGAATTGCACATTTGAATCTCCTAATGAGAGGTTGAACATTAAAGATGTCTTGG

CAAATATCAAGAAGATCAAACTGGAATTTCTTTGCAAATGATATTGCAAGGTTTAAGTAA

GGGATAGCGGGTACGAAGTTGACCCACCAATTGGCCAGATATGCTTCATATCTTTATGTT

ACCACAGTTGGGCAGATGTGCTTGTTTTAATATGTTTTTGTTACTTCAGATAGGCAAATA

TCTTTGTTTCGTATGTTTCTAGTGATCATTCTTGTGATGTACCGCTATGTTTTATTTATG

ACTACTTTGATCAATGTGTTGTTCCGAAAAAGATATACTAACATATACGCATTGTACTTT

TGGAC

>XLOC_018312 transcript=TCONS_00030022

GCCGATTTAACAAAATTGTGCATATTCGACTCTAGGGTTTTCGAATTGCCCCTCAAAATT

CATAAAATTTACGCTCTTGTACTGTTCATCTGATCTTTTCAAGAATTGCAGCAGCATAGC

ATATCTCAGATATTGAAGCTGGAGTGATTTGTGGACCCCCAGTAACCTATTCCGAAAAGT

TGGTTAATTTAAGAAAAAGGAGTGATCGGTTGGGTTTATTGACTAGGTAGAAGCTATTTC

GATGTGTCTAGAGTTATGGGGGATCAAGTATTGGAATCTGTACGCGCGATTATAGGACCA

GGACCTGATTCAAATGATCAGCAGTTAACAGCAGTGCCCAGTTTGGATGGAGTATTGATT

GACCAACCTTTAGCAATTGGGCAAGAGTTCCCGGATGTTGATACTTGTAGAAGGACTTTG

AAAGAGATAGCAGCGCCCAGTTCAGATGGAGTATTGATTGACCAACCTTTAGCAATTGGG

CAAGAGTTCCCGGATGTTGATACTTGCAGAAGGACATTGAAAGAGATAGCAATTGCATTG

CATTTTGAAATTCGAATAGTGAAATCAGATAGGAGTAGATTCATAGCTAAGTGTTCCAAA

GAGGGTTGTCCGTGGCGGATTCATGTGGCCAAATGTCCAGGAGTTCCTACATTTACGATT

AGGACTCTTCATGGTGAGCATACATGTGAGGGAGTTCACGACCTTCACCATCAGCAAGCA

TCAGTGGGTTGGGTTGCAAGGTCAGTGGAATCACGGGTGAGGGATAACCCTCAGTATAAA

CCAAAGGAGATTTTGCAGGATATTCGAAGTCAACATGGAGTTGCAGTGTCTTATATGCAA

GCATGGCGAGGAAAAGAGCGTAGCATGGCTGCTGTACATGGAACTTTTGAAGAAGGTTAT

CGGCTTCTTCCTGCATATTGTGAACAGATAAGGAAGACTAATCCAGGGAGCATTGCATCA

GTTGCCACGGGACAGGAGAATTGTTTCCAGCGATTGTTTGTCTCATATCGTGCTGCAATA

TATGGATTTTTAAATGCTTGCCGGCCACTTTTGGAACTTGATAGAGTCCAGTTAAAAGGA

AAGTACTTGGGTATGTTGTTATGTGCTGCAGCAGTTGATGCTAATGATACACTGTTTCCT

TTGGCAATAGCTGTTGTTGATGTGGAAAGTGATGAGAATTGGATGTGGTTTATGTCAGAG

CTGCGCAAACTTCTGGGTGTAAATACCGATAGTATGCCGAGACTTACTATTCTGTCTGAG

AGATCAGCGGGTATGGTCGAGGCAGTCGATACGCATTTTCCAAATGCATTTCATGGTTTT

TGCCTACGTTATATCAGTGAGAATTTTCGAGATACATTTAAGAACGCGAAATTAGTAAAT

ATATTTTGGAATGCAGTGTATGCACTTACCATAGCCGAATTTGGGAGCAAAGTCTCCGAG

ATGGCAGAGATTTCACAAGATGTGATACAATGGTTTGACCATTTCAATCCGCAGCATTGG

GCTGTTGCATATTTTGAAGGATTACGATACGGTCATTTTTCGTTGGGGATCACAGAAGTG

TTGTATAACTGGGCACTGGAGTGTCACGAGCTTCCTATTGTGCAGATGATGGAGCACATC

CGCCAACAGATGACATCGTGGTCTAATGACCGCAGAAATATGGGCATTAGGTGGACGTCA

ATACTTGTGCCATCTGCTGAAAAGAGGATTTCGGAAGCAATTGCTGATGCTCGCTGCTAT

AAAGTTCTACGAGCAAATGAAATTGAATTTGAGATTGTATCAACTGAGAGGACAAATATT

GTGGACATACGAAGTCGTGTGTGCTCGTGTCGCCGTTGGCAACTCTATGGTGTGCCATGT

GCACATGCTGCTGCTGCACTTATATCTTGTGGGCAGAATGCGCAGTTATTTGCTGAGCCT

TGTTTCACCGTCCACAGTTATCGCGAAACCTACTCGCATATGATATATCCAGTTCCTGAT

AGGAGCATGTGGAGAGAGGCTGGTGAGGGTACAGAAGGTGGAGGAACAAGAGTTGATTTT

ATAATCCGACCACCAAAAACGAGGCGACCTCCTGGCAGGCCCAAAAAGAAGGTTCTTCGC

ATAGAGAGTTTAAAACGTCCAAAGAGAGTCGTTCAATGTGGCCGCTGCCATATGTTGGGA

CATTCTCAGAAAAAATGTTCTTTGCCTAGTTAATCCTTGGTTAGTTGTTTGTTTTGTTTG

TTCATTCAGCAATTTTCATAGAAGAGTCTTAAGCTGTATGTATTTAGTAACGGCTTAGTC

CAGATATTGTTACTTGGTCTCTGCCTTTTCAGTTCACTAGTAGCCCTTGAATATAATCAG

GAACATGTTGTGTACCTTGTATCGTATAACTATATAAGTAGTTGGGTTTCTGACGAATTG

TACCAAAGGAAGCCTATTTTCCTTTTTTGTGATTGTTTTATCTAAGCTCGGGTGTCGGAC

AACAGATGCTTAAAGTAAATGGTAAAATATTGCCTATCTTGTATGACTAGGGGATTTTTA

CTGGAACTTCCCCCGTCAGTTTCTGATGTGAATTTGACAGAGT

>XLOC_033385 transcript=TCONS_00054908

ATTTGTTGTGAAGAACATTGCTTGTCACTTGCGATCTCTCTGCTCATCATTTGTGAAGCA

AATAACTGCAAAGTAATTCATTATATTTTGATTATCCTTTTCTTAGTGTTTCTGGCTTTG

TTCATATGGGTGTTTGAAACTCTTTGAAGTCATTTCAGTTTTAATCCGTTAACATGATTG

AATTTTCTGATGTAGTTCTATTTAATTTCTGAATTCCTATGACTGAATTTGATCCATTCT

ATTAAGGTGTAAGACATAAAAGATTTTTTCCTTCTGATGATTATAATATATGTCCAGAAA

CTGTTTGATAGTATTACTGAATGAGATCATTGTCAAGAATTATATATAATTTTCACTTAG

TTTAGCGTGTAATTCTCTTTCTCCAGTGCGAGCAAATTTGGGAAGTTATGACTACACCAA

TAACACAAAATATTTGGATTAAGTTAAATTTTATGAATTTTCAAAAACATCTGTTGCACT

TATTGTGTGGTCGACTCCTGTATATTGCACCACTTTGCCATAAACATCATACTACATAGT

TGTAATACCTCCAAGTCATGAGTGGCCAGATAGTTCTAAAATTACCCAATTCTTGTTAGT

TTGGGTCCTGATACTACATTCTGTAGTTAGACCTGAGGATTTATAGAATTTAATCTTCTA

CGGTGCCATCATTGTAATATGTAATCACTTCAATTACCTTTTATTTTAAATGGTAATAGT

TATTGTGTATATTGAGATAATGTTACTTGAACATAAGCATGTGTGTGTTGAGTGATGGTC

TGGGTTGAGACCATTCTCGTGTCACTTGAGTATGACCTACAGCCTTTTTTCGGCATGTTC

TATTTATCATAAAGTAATATACTTTTTTTTTATAATTCATTCTTAATGCTTTAGGCTGAG

TTCAATTTATTTGATTACGCCTTTTTATTTGTCCACTTTCTTATTCTGTGCCAATTTCTT

TATTATGCATGTGCGCACCATTTCCTTTTCTTTTCTCTACTAAAGTCGAGAACCTACATA

CACATATTGCTATCAGATTTAAGAAACAGAGAAACCTTTTCGATTCCAACAATAATTAAT

GTCTTGAGGGAGATAGCAACAGGGGATTGACATTACAAATGTCGCTGCAAGTGGCAATAA

TAACTGCACTACTCATGAAAATTTTGCCAGAAACAAGAAATAGGGAGCCTACATATTGAG

AATAAGAACGTGAAGTGGAGGCACTAGCAGAGTTGATGAGGATCAGTAACATGTGAAGGT

CATAATAACAAGGAATTTATGGAGCATCTCTGCTTTGTTGAGTAATGAGTCATTTAGTTG

GATTTTGATTGTGGAATAACTGCTAAGGAGTTGAAACGTGGTAACGCTTTAAGGTTGGAT

TGTTGGATGAGCTGAATTGAAGTAGAAAAAGAATAGAATCACTAGAAAGACGTGTGGATG

AGGTAGAGAGTAAATATGAATGGCTTGCTACCCATGCACTACTTTGCCAGCTCTCACCAC

CACCTCTTCCAGTGAACAATTGATTGAGCATTTAACAGGAACTTGTTCTTTTTACTGATA

AAGAGATTGATGTCTGAAGACCGTATCCGTGAAGAAAGTGCAGCAAAAGTTGAAGCAGAA

GATGTTTTTGGAATTGTTAGAAAGTCTCCTTTGTATATAGCCTACATAATTTCTTTTTTT

ATTTTTTTAGTTAGAACGTCATACAATTTGGATGGATATGTTGTTTTAGTAATGTGGATG

ATAATTCTCTTTGTAATTAATAATGAAATTTTATGACTATTGATTATTTAGATTTGTGTA

CAGATCTGTCGAAGTTTAATAATTATTACATTCGATAACCG

>XLOC_025947 transcript=TCONS_00042682

GTAATAATTACATTGTCTCTATAATTCGTAACAGAAATCTCCTCTACACGATGAAGAATG

AGAAAGGGGGTCTGGCCGGCCATCTCAATAATACACTTCCATGGGTAGTTTTGATAATTC

ACTTGTTATTTCCAATTTCGGAGATGCCCGTGGGAACCCCATCAACTCATTTGAGATGCT

CGAGTGTTGGCTTTCATCAACACTTTCTGGATTTAGCTTAATGATGGCCATCAGGAGTAA

AGGTGTAGAGGCTGGTCCTTCAAAATGATTCATTGCGTCGACTTCCATGCCACCTTTCTT

TTTCAAGCTCGATGTAAATCGAAGCACCTCCACATTGGGACAACTGCTAAGCAATCTTAG

AAGGGAATATGTTAGCTCTTCATCATCGAAATCGAAATCAAAAGGAGCTAGACGGATCAA

GCTGTTGAGGCATGTGGGA

>XLOC_025565 transcript=TCONS_00042066

GCAAAAGTATATACACCCTAAACATACAAAAATCTAGCCTCCTTCTCCCACTTTTTCCTT

CCCCTCTTCTCACCCTCTCACAACTGCTCCTCCCCCACACCAGAAATATGTGTTGCCGCT

GCTCTGTTGCTGGAAAACGAAAAAAATACATTTTTGAATTTCTGTCTACCCCATCTTTCC

ATTATCGCAATTAGTTTTCTTTAATTCTCGTGGAATCAGCGGTAGATCTAATTTCAAAGT

TTCGACCAACACCTAACTCCGGCCCTTTTCCGTTTCTGACATTTTTGCACTAGTTCATCA

CTCTGGCGGTGGCAGCGGCAGTGAGAGTAGCAGGTGACAAGGGAAGTGGCTAGGGGATGT

GAGGTGGAGCATTAGGTGTCCCCTTTTATTGGCAAATGTCTAGGTTTTTTTTTTCCTTAT

GAGAAATGTTGCAAAATTAGACATGACGACAGACGATGTTTCCATCTCTAACTCTAAGCT

CCTCTATTTCTCGTTCTTTCTCCTACTTATGCTTCCTCTTCGTTCTCCTTTTCAAAATAA

AGACAAAGTAGACCGATTGATTTGTGCATTCATTTGTTCCACTTAAGAAAGGCA

>XLOC_023600 transcript=TCONS_00038785

CACCCCCACAATGAAGCTATCTCACCGTGCAGAAAAACTGATACCCTATCAAAGACACAT

AAAAAGTCGAATTTTGCACTAACAACAACAAATGAGTACTTGATGAGCTAGAAAAAGATT

GGACCACCCTTCCTCTTAGTTGACCACAAGTGGTGGATAAAGGAAATAAGTGGATACGAA

TACCACAACTACTATAGCTGTATGTAATAGTCACGTGTTCAGTAGAACTATATGTTAACT

AAACTTTACATACACATAATATATATCGTTTAATCTTGAAGTAGCAGTTGTGCCCGCATT

TGTTACCAAAGAGCTACATCGCTTTTGATTCTGGGATAAGTCCAGACTTGAGGTAGTGGA

TGTGTCTGCACCTATTACCAAAAAAGTAGTGGATGTGTATTTGATTGTGGAATACAATAG

TTCAGTCTTATAGTAGTGGATGTGTATGCACATGTTAAAAATAAATTACCCTTTCCCTTG

ATGGTTCGGTCTTAAAGTAGTGAATGTGCCTGCACCTGGTAATAAAAAACTATGCCCGCC

CTTGATGGTGGGTCCACCATCTTAAGTTGATTTCCGAAATTGTAAACATTGCAACTTGTT

AAAGAGACAGTTATGAGGGGGAAAGGAATCGAAAATAGCACGCAAAGGAATACAAATTGA

TGGTAGTCGTGGCCTGGTTCATTGCCTTCCTTTACTTGACACAAAATATAGCTGGATATG

ATTGAGCTATTTTTCTCAGACGTTGGTTCTGTGTGGTTCTCTTTCCTTATCTCTACTAAC

AACTTTTTCCCTCTCAACAGTACACTTTGTTTCTATTATATATATTGCATGGCTTATCTC

TTTAGTTCTTCCCTTTCCATTAGTCACTAGAAATCAGCATAATAATGTCTAATCATGAAA

ATGGTACTAGCTTGCCAGCAGACCAGCCATGGCCAGATTTTATATTACCTGACCCATTGT

ACACTGAAACCATCAGAGAAATTCATTCGACTGTAGAGCGTAATTGGGATTCTTTGCGAC

AATCAGCATGTCAAACTGCGGCAGGAAGGGCACTATGGAAGCATGTGATCAATGATCCAT

TAGCAGAGTTACTTGCAGGAGAGACATACCTGATAAAATTGTATGAGAAGATTAAGAAAG

ACATCCTCAACAATGCTAGAGAGGTTTCTGGAGTTATCATTGCTGTAAGAACACTATGGT

TTGATAAAAGAATTGAAGCAGCTCTTAATTCTTTTGATGGTGGAGGATCACAAATTGTCA

TCCTTGGGGCAGGTATGGATACAAGGGCATACCGTTTGAGTTGCTTGAGAGACAGTAACA

TTTTTGAGGTTGATTTTGCTGAGGTCCTGCAGATGAAAACCACTATTCTAGAGGCAGCAA

CAGAAACAACGAATGAACAAAAGCACCAAATGATGATGGCAAAATCACTGAACAGAGTGG

CAGCTGACCTGAAAGAAACGGACTGGCTTGAAAAGCTTCAGGAATCAGGCTTAAAGCTAA

ATAAGAATACAGTATGGGTATTAGAGGGCATTCTCTACTATCTCTCGCACTCAAATGCAA

TGGAAGTACTGAGAATTATCGCAAGAAACTGTACCATTGCTCATACGGTACTCTTAGCGG

ACTTCATGAACAAGCAATCAACCACACTGTCCAGCTCAAACTTCCATTTCTATAGTGACT

ATCCTGATCAGTTATTGCCATCGCTAGGATTTTCTGATGTTAAACTTTCTCAAATTGGTG

ACCCAGATGCCCATTTTGGGCTGTTACATGACCCACTAAATTTGTTCAACAAGTTGCGCA

ACTTACCGAGGTCACTTCAAACTCACCCAGATGATGGAACACCATGTTGTAGATTGTATT

TGCTTCAGGCTATTGGAGAACCACCA

>XLOC_007427 transcript=TCONS_00012090

TCCATATCATCACTATCTCCAATTTTTATACAAAAGGTATCCAGCATTAAAAAAGGGATC

TCTTCAACAGATTTTAGTCATTCCCCTAATCTCTTCAATTAATCCCAATTTTCCCGTGAT

CCCTTCAATTCCGTTTCTTTCCCCTAACGTTCTCAGAATCCCCCAAATTCCTCCTTTTGA

CCGTTGGCATTGTCTGCTTAAATAGACTAGTGAAAAATCAAGATAGGGGAGACCAAGAAA

GAACCAACCCCAGCTCCTTTCTCCAAAAAATCGTTGCAGACACAATTTATTGAAATACAG

AGGGACATACGCGCACACACACAAGTGGAAACAGGGGAAGGGGGAACACCACCATCAACG

GTGGTGGAAACCACAATAAACAATACACACCCACACATGATATACCCACACACTCCGGGG

AAAAGAGAGAGACGGAGGAGGCAATTGAAACAAATTGGGGTAGTAGGGGAAGACCCATGA

CTGGAAACCTCCAAGATTCTGGCCGGAAAACGCCATTTAAGATGGTAACAGAAGCAGCGA

AGCAGTGAGGGTAATGGAGATCGGAGTAGAACGACACTGACACCATTGACGAGTAGCAGC

AACGCCGCTATCGGAAAGCAGCTGCCAGCTAGGGATGCTTGGTGGAGATGGATTAATTTT

TGTCTCTATGGCCTTGTTAATGATGCTATTAATTAACAAGTGCTAGTAAGTGTGACTATT

GCCCTACCCTGTTAATGTTATTGATATGTTCTTACTGATTAATGTTTTGTTTGGTTGGTA

ATTACAAACAGTGTTATGCAAGGCTGCACCACTCTTACTGGCCTATAAAAAATATTAATT

TTGAGACTGATGGACTAATTTGAATAAGATACTACACGACCAATGATATTTTGGAGGCCA

ATGCAAGTCTCGTCTAGTTATCCCGTTATGCATCCAGAAAATAATTAAAAAATAGGCAAT

AACGATACGTAATACTTTACAAAGTAAGCAATAGTTGAGTAGTGGGATTGGTGGTACTTG

GCATGGCAAACCGGCCTGGCCACCGTGTTTGCAGCCGTACCGATATCCCTAACCTCTACT

TGATGCATTACACCAATCTGAGCATAAAATTTTTAAACATTTATTTTTTCCTTTTTCTTA

ATTATTATTATTATTATTATCATTATTATTATTCATTACTTATTCATACTAATTCATTTT

CACATTATTGCGAGGTACATTCAGACGAAATTCGTTAAAGACAACCCAATAGGAGAGGAC

AAAATATTTCAAAACTATTACATTCTTACTCAATTTTATTTTATTTACGATTCTTACTAA

TTTGTTGTTTTATTTGCAAGGTACATTTAGATGAATAATATGAAGATGACGCTCCAGAAA

ACGCTCCTAATCAAATTTACTATTTCATTCTTGCAATTTTCATTTTATTGTTGTTAATTT

ATTTTTGATACACTAACTGACTTATTTTTTAATTTTTGCAAGGTCTATTTGGTTGAAATT

CCTAAGGAAGAAAGCAATTAGAGAAGATGAGCCTTTCATTTAATTTTAGAATACAATTAT

TTATATTTAAACCGAACCAGTATAGTACTCGCGCGATGCGCGGTTGATATCAACAGAAAT

TGATCGTACTAAACTATTATCAAAGTACCAAGTGTGAAATTATGATATGATTTTTGGTAT

TTATCACCGTGGTTTGCTAATATTTATTTTTTTTGGTTTAAACCAAACCAAAATGATGCC

ACAACTAAAAAAACAAGTGGAACTAACTAATGATGCATTTATTCAAAAGAAAAAAAAAAT

GAAATCACAAAATAATAATATTGTTTGTGATGCAAATAATTTAATTATTATATAGTTTGT

GGTGCAAACAACATGAATTGATAAATTAAATCATATAATGACATGTAATAAATGGATGAA

AATTAATTTCAATGACATCTTAATCAAATTATTAGAATCTACCTATACACAAAATTTAAG

CTTTATTATTTATTTGTTACAAAATAACTCCTAAGTAAAAAAAATATTTAGCGTTGGTGG

TGTTTCTTATGGAGCCATTTTGAAATAATAAACAGTAATTGATCATCAACATGACCCATT

TAAGGAAAAATAAGCATAAAAATTTTAAGTTTAACCTCAAAAAAATATTTGGAGTATTTT

GGTCTTTCTTTACGCTTTCCAGTACATGAAAATGGATCAACTGAACGTTAGTGGCTATTG

ATTTTGTTACAACTTACATTGCATATCGATTATCATAATTTGAAGATCTATTTATTGAGC

TAGTCTATCATGTATTTTTAGTAATTTATGGATGTGCATGTTCCTATATGATACTGGTTT

TTGAACTTTGGAAAAGTTCACACATACAATCTTAATAACCTATAATTCACACCATCCCAC

ATTAAAGAAAAAAACAGACATGAATGGTTCAATAAAGAACAAACTTGTGTACTTCAGCTA

GCAATCAACAATCAAAGGAAGGGAAGAAATCCTAACATTAATTTATTTAGGGGTTTATAG

AGATTACAAATCTGATATTTAATTCATTCAACGCAAATATCTTTTAAATATATTCAAAAC

TTGTACCAGTTTCTGAGGATATTTCTTTTCTAAATAAATATAACGTATATTTCGAAAGAT

AGGATTGTAAGGCTTATTTATCCTTATATTGTATGATAAAGATATAAACTACCTGTAGAA

TATAATACCACAAAAAAAAAAAATTTGCATAAAATTTTGACGATAGTATCAGGTTAAGTA

AAATTCAATGCTATTATCTCATTCACTCTTTCACATAGTATAGCTAAAAAAAAGTATCTA

AAGATGAAAATATTTAATAAGGGTAATGACAAAACTATGTAAAGAATTTAGGATAACTCA

AATACTAACTTAGACACATGGGAGTATCAGTGTTCTGTTTTTATGCTTTTTCTTTATGTA

ATTCTTATTTGTAATTAATTTTTAGTTTTCTGTATAATGAAATATAAAAGAAAAAGGTAA

AGCAAAACCAATTCCGCGAAAGATCATTACTCTTTCATCTATAAGCAAATCTAAAATAGT

ATCTAATATTGGTCGGCTTAATCTGAACTTTATCTGGATACAAAAATGTAACCTTTTGCA

TTTCGGCTAATGGTAGGTTTAATTATGACAAATAACACACGCATGCATCCAACCAAAAAG

TTATTTAGTCATAATCTAAATATAAGTTGCAAAACTCACCATAATGCTAGAGCTTAAGTA

GAGTACCTCCTTCATACTTAAAGTGTGAAACAAATACATCACTATAATCACAAACTATGA

AATCAAAAGGCAATAATTCTAGTTCTAGAATAAAATGCAAAAAATGGTTCCAACTCTTCC

TTGCTGGTAAGTAGTGCCGTTGGAACGGACTTCGAAGTAGAAGCATGGAAAGAGCACCCT

TAACAGAGCCAATGCACCTCTATACGTGGACATTTCATATTTGCAATTCTGCTATGTAAA

TCATAAAAGACATTAACATGAGTTGCAGCTTCAATCTTCACAATTTCTATATAGTCTATT

TCCCATCTTTAGTGAATGCTTCTCATGCTTTTGAATCAAA

>XLOC_033240 transcript=TCONS_00054661

GTAATGTTTATTGTTTTACAAAATTTCTTGTAGTCTTGAGACTCGAAGCTATGCTTTTAG

GGGTCGCTCGGTACGAAAGATTGGATTACTTATTCCGGAATTAAATTCGGGATTATATTT

ATCTCATATTTGGTATAAGGTATTAGCTAATACTAGGACATATTTTATGCATAAATTGTA

GTATTATTAATCCCACATAAGAGGCGGGATTAACAATCTCGGGATTGAACCGAACGACCC

TTAAGTTCTTCAATTCCTTGCGTTAATCCGTTGAGGCTTGACCGCTTGAATAGAGTTGTT

TCACATCTACGTTACATTGATTTTCCTAGTAAAAGTACGAAGGGGGACCAACACTTTATT

AAACTTTAGGTCTCTTTATTGTTATTTTACTAAAGTTGATTTCTCAAAGTTCTATTAGGA

AAACTAACTGTAATCTTTTTCACCACCTGAGGGGATCCTAGAGGAAATAGTAGGGGGAAA

ACATAAAACACCGCGTTCAATTCTAAATATACAAAAAATTTGGTAATTTCTTGTTATTAG

TCTTATTTCTAGTGATATGTGTTGTCCTGTACCTGTACTAAATGTAAGAGATACTTAGTG

GAATAGAGGAGGCAAATGTACATATTTAATGTCAAAATTAGTCATCTAAATACTCGAGGT

GTGTGTTTACATGTCTAGTCAAATTTATTACGTGTCAGTTAGAGGCGGAGCTATATAGAG

CGTAGGGGTTCAATAGAATCATGTTTCTTAGAAAATTATATTGCACAAAATTTTGGTAAC

ATATGAATTAATGAATCCCCTTGCTATGAACGAAGGTTTCGGTCTAATGGTTAAGTGGTT

TAAAAGTTGATTTAGCTCCCGTTTGTTTATATTTTTTTGAGAGAATTTTTTGGAAAAAAA

ATTGTTTATTTATAGTATTTAACCAAAATTTGGAACAAAAAATCCCAAAAACCAAAAAGT

ATTCACTTTTTAGGATTTTTGGAGAAATTTTGTATCTTTTTGGGATTTTTGGAGAAATTT

TGTATATTTCACCCACAAAACTTCAAATATTTTTTCAAGTGAAATGTATGTTCAAACACA

ATTTCAAGTTTCAAAATAACTTTTTCAACTAAATTTCAAAAACTCAAATTCTCAAATTTC

AATCAGATCTATGTCCAAACATTAGGTTATATATGTTCAAATCTTCAATGAGATATTTTA

GTAATCTTTTGAGGTCCCAATGATTGTGTTATTACACACTGATTTTTTAAGTTATACACA

TTACCCAACAGATATTAATGTACTCCATCCGTCTCATTTTAGTTGTCGCTTTAGGTTTTT

TCACGCTCATTAATAAAGATATTAAACAGGGTGTTTATTTTACTATGTTATCCCTATTTA

ATGTTTCACCATTAACTTTTTTTGAGATTTTTTTTAATTATTAGGAGTATAGTTGGAAAG

AATTAATCAATTTAGCCTTGAAATCCTAAAGCGACGACTAATTTGGGACAAACACATTTT

GCTAAAGTGACAGCTAATTTGGGACGGAGGGAGTATTTACTTTAGGCCAATTTAATTGCA

CTACCTGGTCCCCAATAAGAAGATAGCACTTCATGTCTTTTTGAATTCAGTTTGGTAGTG

AGTTAAAACTGTTAATGACGTTCACATTATAGTAGTGGCGATGGAGGAAAAAAGTTACTA

AAGTAATGATTGAAAAACTTAGAGAAATACCACGTCAAAAATTAAAATTTGTATAATCCT

ATGAATTTGAGTGCTTGAAAAGTTTTTCAGAAATTGAGCAATAATTAGCGGGGATTTATT

TTTTTGAAAATGCACAAATGTATGGTTATCTTAAAGAAAATTTTACAGTTCCAATATTTG

CATTTGGGAGAAAAATTAAGCTTTATTTATAGCTGCTGATATGCATGGAATAGTTATTGC

TATTTGCTAATATTGATAGCCTGACATCTTTAACCTGCACAGTTATGTAAAAGGTTGGAT

CCCATTAAGAGCCCTTCTTTTGCCTTCTGTTGATGATACGACATTTCCATGTGAATGATG

GATGAGCAATAACACAGCTTTAACAGCTATCCTTTACTTGTTTGTGTTAGAGAATGCTAA

GTTTTGTTCCTCATATGTTGGGTAAAAGTTTCTAAAGAGGTATATATAGTTATGGCAAAG

GGGTCAACTAGCCCCTTATACTTGTCTGGTTTCGCCATAATCGCACTTGTATTTAGTTAA

GTATCAAGTAGCCTTTTATACTCATCCAGAACCTGCTGTCTTTATAAACCATTGTGCTTA

AGTGTGGTGCACAAACGCCACCCACGTGGCCGCCACGTCAGATGATTGGCCATATCATTT

GCCACATAATATTTTATGGATTAAACAAAAAGGAGAATGAAAAGGAGATGGTAAAAACAA

AGAGACACAAAATCACACCCTATTCTTTACCCGTTGATTAACCAAAACCACATGCACCCC

TTTGTTTAACCCTTAGATTAACTAACCAAAAAAATCGATCATCTTTACCGATTAACAATA

TATTATGGCTTCGTTCTTCAAATTTAACTCAAATTGTTAATGGTTTTAATCATCTTTCCC

GATTTGTAAGGTAAGAGTTTTAACCCATTCTCTTTAATCAATTGTTAATTTTGTGATTTG

TTCTTGTAGGATTTATAATTGATTAAAAGAACACATAGTCAGTCTTTGTTTAGAAATTAC

GGATTTTAATTTAAATGTTTATTATTAGAAATTAGGCTTTTTAATCGATTAACAAGAATA

TTGAAATATTTTGGGTGAATCAATTAGAGTAAGGAATAGAGTAATGAAGACTAAGAAAAA

AGTAAGGAATAAGGAGAAAGAAAACTAAGGAAGAAGATGCAAAAAACAAAGAAAAAATGA

CCCCTTTTATTTCGGTAATGAAAGCGCTTAAAAAGATCAAAAAATAGCTTTTGGAAGGCT

TTTTTCTGATCCACTCTCTTAAAAGCCATGTATTACACGCCCATTTGCCACATAGGCAAG

CTACTTGACACATCAGCGAAGAAATGTTTAAAAACATATTTTGGAAGAGTATAAAGGGTT

ACTTGACACTTAGCTAAGTACAAGTATGATTATGACGAAATCGGACAAGTATAAAGGGCT

AGTTCGCCGTTCTGCCTATAGGTATTGATAACTTCATTCATTTACGTGAGGTTTTGAAGA

TGCTCAAATTTTCACAAGGTATCATACTCAAATTATGGTAAAAACCCATTTTCACGACTA

TTCCATACTTTAGCCCGTCAGAGTTGCTAACTTAATTCTATACTTATTCATTAGCATGTT

GAGTCTAATTTATCTTTATCTTTTTCTACTTTGTCCTCTCCACGCTAAGCCTCCCTAATA

ACTATTTCAAGAAAGTCACCGTAATGAACTTTTGAAAGTTTTTACGTAATTTCATCCCTT

TTATATGATTTATGCCTTTTAAAATTATTTATTTTAAATAATATTTATTATAAAATATTA

AATACTACAGATTTGTGGGGCTTCCGTCCAGTGCATTGGGCTTACGTCTCGTCCCATGTC

AGGTAAAAACGCCTCGTGCCCACGCCTTTTATAACTTTGCGGTGAATAATGAGTTTATCC

CTTTATCCTTAAATGCAACACACGTCGTGCGTCTACCTACACGTCTAAGGCAGCTCTTAC

TACTAGAGTAAAGCCCTGTGCTATTGATTTCATATTTTTATAAGGCAGAAGGATCAAATG

GACACCTATACTTCTCTGGTTTAACCATAATCACATTTATATTTTGCAAAGTGTCAACCA

GCCCCTTCTACTCGTCCAAAATATGACTTTTAAACCCTCCCGTCTAAGTGTGGTGCACAA

ACGCGACCACATGGCTTCCACGTCAGATTGGTGGCCCATATCACATGCCACTTTTTTTTT

TTACCCTAAAAAACAAAAGAAAAATCCAAACGACTTTTTTTTACCCTAAAAAAGGAAAAA

AAAAATCCAAACGACTACACGGTAAACAAAACTAAAAATTACTTTAACTCAAATAATACT

TACCTAAATCCAAACTTACCTCATCGATTAACCCAAAAAAAAAAAAAACCCCCCGTCGAT

TAACAACAAAAAAATATCCTTATTTGATTAACAATATTTAACTCGTTTGATTAACAACAT

TTGAAGGTTTCCCCATATATGAGGTAAGGGTTTAAATCCGTTTCCTTTCTAATTTTTGTA

ATGATTTGTTATTTGCGATTTGTTATAAAAATTAGGGCTTTAATTAATTAAGAATTTAAC

TAATTAGGACCGTTAAAGGATTAGTTCTCTAACTAATTAGGGCTTTAATTAATTAAGAAT

TTAACTAATTAGAGCTTTAATTAATTAAAAATTTAACTAATTAGGACCTTCATTCCATGT

CTTCCAACATTAAATCTGAATTTTGTAAAAAAATAGACTAAGAAAATAATAAGAAATAAA

GGAGAAGGAAAGAAACTAAGGGAAAAAAAAAAAAAAAAGCAAAAGACAGGAAAAATGAAG

GTGAAGGATTTGGTATTTAAAGGCCTTAAAAAGACCAAAAAATAGCTGTTGGTAGTGCTT

TTTTTACTGATCCACGCACTGAAAATTTGTGTAGCTCACGCTCACTTGCCACATAAGCAA

CTTATTAGACACATCAGCGCAGGATGGTTTAAAAGTCATGTTTTGGACGAGTAGAAGGAG

CTGGTTGACACTTTGCAAAATATAAGCGTGATTATGATTAAACCGGACAAGTACGAGTGT

CTATTTGGGCTTTCTGCCTTTTTATAAAGAATTTCTATGTTTTTGGGACAGATTACTGCA

TTTGCTTCCTATGTACACTACAGTGTCATACTCGTGAACATACAGAAGGTGTCGGGTGCA

ACCCAAAATAGTTGATTGAATTCTTTATCAACAGTTGTTTTTTACTTTGGATGAATTTGA

ATCTTATTGCTAATTTACTTCTTTCGGTGAAGTTTTATATTAGACCTTTGGTATTGGATA

GGCATTAGTGGATCTAAGTTTTTAGATAATGTTAGCGTCTAAGTCAATTCATGTGCACAT

GACTAATTAATTGAATATCTACTATTTCCCACTAGCACGATTATCAACATAATTCACTCA

ATATCTTATATTGTTTCTTTAATCATATAGTCTACCCTTGGTTATAAGCTGTACCATTTT

TCTGTTACTGCTGTTGCTCCTTGTAAAAGACTCAGGAGCCATGCCACTGGCAGGTCCAAG

TAGCATTAGAAATTAAATAGAAACTGTTTGGGAGAAAAGCAGCAATACAGGAAAGAAAAA

GGGAAGGGAGAGGTATGGAGCATTTCTGTCTTTTACCAGGCTGTGTGTGTATTGGCTTTC

TTAAAGGTCCAAGTTCTCTGTTTGAAGAACAGACACTAGTCATATCAACAATTTCAACAT

AATTATTGGATTTTATTACACAGATATTATTTGCAATTTTCATTATGCTAACATACAAAT

TGTTTATGCAAAAGAAATGAGGTTACAAGAGTATGGAGATTCTCCTGTCAGTTTCTGTAG

CCTTCTGCTGACGTTTTGATTCTGGTCCCATGGTACCAACTTACCAGCCTCGCCCTGCGG

ACGAATTGCTTGAAAACCATCAGAAGTTATATGCCTAAAATGCCCTCTTGTTCAATCAGT

TGAAGGCCATAACAAATACTTCTAGCAAAAGTCTTCAACAACAAAAACAATTACAGCTCA

ATCACATATAAGTATAAGATGCGTGATAAAACCAGCTTTGAATTGCCGTTCTTGAGCATT

CAAGGTCTTCCTGCAGGCCTTAATTATACAGGAAGGCTAGTGGACTACCTTTACGTTTCC

CTTCCAATTGTGTTTAAATCTATGTTGTTCAGATCCTTCAAAAATTTCACCATACCTGTA

TCGAATGCAGGGTGTAGATAGAGACCTAAAACATAGGTTCGGCTGAACCCAATAGCTTTG

GTCCAAATCTTGTATTTATCCTAACTAATTCATTTAATATGTTCAAATTATTAGTTTAGA

ACCAGGTA

>XLOC_029895 transcript=TCONS_00049222

ATCGCAACCCGCAATTCCCAGTTCAAAAGGAGATAAGTCCAAATTCCAGCTGGAAATGGG

TACAAACCTTATCCGTAAGAGATCCGTTGGAGGTCATTTTTGGGTATTTCTTACACCTTA

AAGAGCCCTAATGGTTTGGATATAAAAGCGAGTCTAGAGGTCTTGGTCCAAGGAGTTGAT

GGAAGCAGCCATTAATGGGGTAGGAGAGAGCAACGACAGTAGTAGAGAGTTCATAGAGGA

GCAGCCGTGAAGGGGAGAGTGGATAGAGCAGCGGCGAAAACAGAGAGAGGGAGTTGATAG

AGTAGAGGCAGTGTAGGCACTACATATGGAAAGGTTTATTTTCTCAGTTGTGGAGTTCAA

AATTGAGTGCTATTGTTTCCGTTGATTTTATTGTGTTCGAGTCGTCGCATCGCTTTTGTT

CGAGTCATTTTTTTGGATCCTCGCCCCTATTCACTGCCCTGCAAAAGGTAATCTTCTCAA

CTAATGTGTTTACTTATTTTAGTTGCTTAATCTGCTCATTGTTTGTCTGACCCTGCATAT

TACTTTGTTTTAAGAAAAATGAATGAAGCTGTAGTAATAATAGCCTTTTCCTCTTCGTTT

TCAGTTTCGCATTTCTTTGGTGAAAGTGAAAATCTGTTCATGTCAAGTTCATGAAATTGG

ATTCAAAAGAATGTAGTCTCGTGTGGTTAAATCCATTTAGTAGCATGTTCTTTCTAAGTT

TGATGTCGATTTTAGGCTTCATTTTGTTCGATATCAATGTATATGTAGTTAGCTTGATTA

ACTAATTGCACTACGGAGTAGCTGACCTTTTGAATCTGCCTAGTCTAGTCTATGATCGTT

CGAATCTGCGTTTGAGGTTAAAACTAGATCATTAGGAAAGTTTTCATACATGTTGTAGGA

TTAAGGAATTAAAATCTAGGTAGGACAGGAAGTTAATCAGTTTATGAGTTTAAAATAAAA

ATTTTAAGGTAAAATGAAGGTCAGTCTCTTTCCCCACTTTCGTTTTGGCTCGTTTACTAA

TTTTGAATGAGTTTCTCAGTCAATATAAGCCCATTGCGCTTCTGCGTTTAAAAAGCAAGT

AGTATTCTTTTCATCTGGTTAAGTTTCCTGAATGCTTTTCTGCCTGTGTTTATTCTTGTC

TGGTTATCTCTTATGCTTCTGATTCTTAGTGAGTTTGTCTCTAGTTCTGGTTGTTTGAAT

GGAATTCTGTTTGTAGATAGTTGGTTTGTCTGGTTGTTGTGAGGAGTCTGTGTGTATGTT

CTGGTGCGGTTATAATGTCTCATGAGGGATGTTCAGGTGTAATGTTTGTTCTAGTAGGCT

GCAGGTGTTTGCATATGGTCTGGTTGGTTTGCAGGTGATTAGAGAAGTTAAAGAGGCAGG

GTAGTAGCAGTAGTCCTGCTATTTCTGGTGCTGCAAAGGGTGAGGGAGTGTATGTTTTGC

TGCAATTTATTGGAGTCAGTAAAGGGGCAGGTTAGAGGAGATTAGAGCAGGGTGTAGGCT

GTTTTAGAGGTCAGGTGCAGCAGGGTATGTTCCTGGTAATTAGTTTTGTCCTGGTTGTTT

CTGGAGGAGGGATGTCAAGGAGTCTGTGTGTGTAGGTGGTAAGTATGTGTGTGCAGGTCT

CTGTGTGCGTGCTAGTGAATGTGTGGAGTTGGTTTTGGATGTGAATTTGGGGTCTGTAGT

AGGTGTTCTGCACAGTTTAGGATGTTGTGAGCATGAGGGAGTCTTGGAGAGGTTGTTCTA

TCCTGAAGTAAGTAGAAGTGTGGTTCTTTTCTAGATAAAATGTCTAGGATAGTATATGTA

GAAGGGTTGTCTGTCCTAGTTGCTGTGAGTAGAATTAGGGTCCGTAGGTTTCAGGTCAAG

TGCTTCTTTTGTTCATGATTGCATTAAAGTGCATTTTCAGTTTTCCTTTAGGATCGTGTC

TTGAATAAGTGAAGTTTTGTTTTCAGAACCTTTTGTTTGCATTCCAACAAGTTTGATTTG

GTTTCACTATGCAGACTCTCTGTTTTTTTAATCAGTGCACTAGTAGAAAATCTTTTTATA

TGAAGCCAAGTTCGTCTTAATGACTTGTTCATGAAGTATTTCGCATATTTTCTTTTATCT

ATAATCTGCTATATATGTTTCAGGTTGGCTTAGCCTTTCTTAAACAAGTTTTAATTTCAT

AAGTAATTTCATGTAATGCTTTCTTTGGAAATAGGATGAATATTGTAAGCTGCTAATATA

ATCTATGGATTGGATTTACATAACAATGAATTTGATTGGCCACTATGTTTTAGTAGGTAT

AATCTTAGTTTTGCTTAAATGATTAGGGTGATGGTCTGTGACAGTTTTTCTTGAATTAAG

TACCTGTTAATTTCTTCTTGCCTGCTTAAGTCTCATCCAGTTTTATTTGGAATATGAATG

AACAATGCATTCGCTTTACAATTTTTTTGATTATTAACAATGACATATGCAATATTCAGT

TTGAAATTTGGAGAAAGTATGTTTAAGCTCCTTGTTCATTTTATAAAGTGGAAAAAGTAG

TTTTCTTATCCTTCAGTTAAGATAAGCTTAATTTATGTATTTTTGTTTAAAATCATTTTT

TCTAGAACTTGTATCTGCTAGCATCTTAAGATTCATCATGAATTGGTCTTCTTGATAGTT

TGAGTATAAGTTAAGATTGAGGCCATAGATGATGCATAAATTTGAAGTTCATAGCAGGTT

TTATCTCTTAGGATTATTCTGATGTCTATANGATTATTCTGATGTCTATCTCTCTTTGGT

TAATGATAAATTAAATTATTCATTAATTTGAGCATCTCGTTTTTTAGATAATGCTCACTT

CATTTGGATTGTAGATTACTTTAATATCAAATATAAGTTGTGAACTTTAGTTAGTTCAAT

CCATATCTTCGAAGTTTCTTTTTATGAGGAGTAATTCTTTATCTTGAGATCATGTTGCAT

ATTTTGGAAGAATAAAGTATTTTTATGCTGACTTTGCCTAATAATGTTGAGCTCTGCATC

TTGTCTTGACTCATGCCTAGAAGGTTTAATTTAAAGGTTTGATTAATTGCTTTATTTGTT

GTTTTAAAGTAGTAGTTTTTGTATTAGGAATTTTCAAGTAGCTAGTTTTGATTCAAAAGA

TTGTGCTTTTAGAACCTGAGAAAGCAGGTTGCATTTTTCTACCCATTAGACACTATTGTA

TTGTAGTATAAGTTGGTGTTTATTTCTTGATCCTTCCCTACTTGTTCTATGAGTGAATGT

GTGTGTTTATTTATTCAACTGAAGATAGATTAACTTTTATGATTAGTATCTAGTAATTTT

TTTCTTTAATGTATTAATTTTTATCTTCCATTTTCTTTGCATAATACACTCTTGGGAGCG

AGTTTGGCTAATACTCAATTGGGATCGACACATTGGTTTGAAGTCACTTGGGGTCGAGTT

CGGGCTCAAGCATTCAGGATATGTGACCTTTAGAAAATCTTAGAAATTCAAGGCTCCCGC

GAGTTAGTCATTTTCCGGGTTCTTCTAAATATCTTATTCCTTTCCCTTTCCTCTTTAGGA

CAGTTATGTAAACTTTGTCGAATAATTTATTGTTAATTCATTTATGAATAATATTAGAGT

TATTTGATCCACTTTATGTTCATTACCTTTAATTTTGGAGTTGTCTTTTATTTTTACGCA

AATT

>XLOC_028033 transcript=TCONS_00046103

TACAAAACAAGAATTTTCTTCTCTTTTAAACTTTCTCTGGTTCTTTCCTTTTTCTCTCTT

GCTTTAATTTTCCTCCCGTTTGTCTATAAAACTTGGGAGCTTTGGGAGCACTTTTTAGGG

ATTTTTGGAAAAAATTTTATTTGGACATAATTTTTTATCAATTTTTGGTGAAGTTTTGAA

AAAGAGTTTTGAAAAACCAAAAAGTTTTGAGAATTTTTGGAAAAGCTATCTTATCTTACC

CACAAAATTTCAACTATTTTTCAAGTAAGTTTTATGTCCAAACACAAAATTATTTTTCAA

CTCAAATCCAAAAACTTAAATCCCCTAAGTATCAAACAAATCTAGGTCCAAACGCTAGTT

TTAGGATAAAGCTCTTCTCTTGATGAAATAACGGACTAATGGTTAGAGAAGTGGACCAAT

GATGAGTTGTTCGTTTGAAATCTCAGCCACACTATCCAAAGTAAGGCTGCATCCAAGTGC

TTCTATTGTTAATCATATGATATGCGAAATTTACTGATTTGTCTAACCGATATCTTATTA

GCTCCGGATAGGACTAATGAAAAGCATTGGCAAATTCAAAAATGAAAATCTTGGAGAAAG

GTTCAGTATCCACTATCCAATCTATAGGCAATGTCCTCTTCATCTTCTGTCTTTTGCTTA

CATAAAAATGAATGTCAACTATAGGCTAAATTAAATGGCTTTCATATTCGTGTCCAGAGA

AGATGATAGATTTATAGTTACCATCATGACAACAACAGTGAAAGCATATGTCTAATGACG

TTACTATCCTATTTGAGAGTTAGGTGCACAGCTCATATTTAATAGGTTCAATCGAATTCA

ATATTTTTAAACAAAAATTTTATTAAAATAAACTTTAAATACTTAATTTAAAGAGCACAA

TAACTTTGATATTCAAAATACTGAATATTGATATCACAATCCTCAATTTACCCCTGATTC

GTCCATTTACTGTTTTTGTTTACGAGTGCCATACTTGAGATACTCAGGGTCGTTTGGTAC

AAAAGTGGTTAATCCCGCGATTACCAATTCGGGGATTAAAATTGGGACTAGAAGTGGGAT

AAATAAATCCATCTCCTATATGGGATTAGGTAATTCCATAATTTTGGGATAAAAGCTAAT

CCCAACTTAGCTAATCCCAACATTATCTCAAATCTTATCCAAGGATTATAATCCTAATCA

CTTATGAAAACTTCTGGCGACCCGTTACAGATTTTGCCTCTATAAGAATTTTTTTACAAT

TTTAGCCCCATAAAAAGTTTTGGCGATCGTCATACCTTTCTACTAGCAAGAATTGACAAT

AGTTAGATCTGTCTATTTTGCACAATTTACAAAAATTGTATTTGAAAAGATGAGTTGGGT

CACAATAATCTAAACAGCGTCATAGTCCACCTGTCAAATTCTTAATAAATTTTAACCTTT

TTATTTGTGTTCATATAATTTGTTTAATTACTAAACAAAGTCAATAAAAAGGTTCTCTTT

ATTTTGGTTATATAAAATAAAATACGGAGAAAGGCCAAATATAACCCTCTACTTTCATTT

ATTCGTTAAAGCCATCCCCCGTCATACTATCCGAACAAATATACCCCTACCGTCATACTA

TGGCCCAAAATTGCCCCTCTAGCTAACGGAGGGGACACGTGGCTGCCTATTACGCCTAAA

CCCCATACCCAACATAAAAAACCCATCCCCAACTAAAACCCGACCCATAAACTAAATAAC

CCCCATAAACATTACCCCACACGTTTCATCTCACACGATTATACACTAAAATTTAAGGGT

TGACTTATTATTTCTTCTCCCTCTTGCAATTCGGATTAAAATCTCAATTAGATCCGAAAA

CTTGTTGAATAATGTCCCAAACAAGTTGTTCTTCTAAGAGGAAGTGCTATTGTGGTAAAA

TGGCCAAACAATTCACCTCAAACACAGAATCTAATCCGGGTAGGGTATTTTACAAGTGTT

CTCAACCTAAAAGAATTTCTTGTGGATATTGGGAGTGAGATGATGAATTGTCACCGAATG

AGTCTATGATTGAAATAAATAATTTACAAGCTCAATTGGATGATGCAAAGTCTAAAATCA

TAAACTTAGCCATGGTAGTGGATGCGCTTAAGGGTGAGTCGGATGAGATGAAACAAAAAG

TGAACAAGGTCATGAATTTGGTAGATGGAGTATTGAAGCTTAGGATGTTTTTTTTCATTT

TCTGTGCAATGTGCATAGGTTATTTGGTTGCTTCAATTATGAAGTGAAAGCAACCTTTTC

ATAATGAAATCTTAGAGTTTTATGTTAATGTCTACTGATTATGAGATT

>XLOC_005902 transcript=TCONS_00009618

AAGTGCTGATGGTAGATCCACGGGCATTAAAGGGTGTGAACTTGAGCACCAAGGCATTTA

AAAAAATGAAAAATCTTAGGGTCCTTAAAATCGATGAATTAGCAATCAGTGGAGATCTTG

AGCTGTTGTCCAAGGAGCTCAGATGGCTGTCGTGGAAAAAATGTCCTTTAAAATGTATAC

CATCAGGTTTTCCAACTGAGAAACTTGTATTTCTAGATATGAGAGGGAGCAATATCCAAG

AATTTGGTTTGAATTTGCAGTGTTGTACCAATTTGAGGAAGCTGGATCTCTCTGATTGCA

AGCACCTCAGAAAAACCCATAACTTCAATGGTTCGCGAAGTCTTGAGACTTTACAGTTTT

CCGGTTGCTCAAGTCTGACGGAGATCCATCAATCAATTGGAAATTTGGACAGACTAATTA

GTCTACAGTTGGGAGGTTGTAAAAAGCTTACGGATCTTCCTAGCAGCATATGCCATCTCA

AATCCCTTAAAAAGTTGGACATTAGTTGGTGCTCATCTATACAAACACTGCCAGTTGATT

TAGGTGATATGCAAAGTCTAGAAGGTCTTTATGCAGGTAATACAGGTATAAAAGAATTGC

CTGGATCTGTTGAAATGCTAAAAAATCTTGCAGTTTTGATAGTGGGAAGTCGAAATTTTG

AGGCCAAAAGGACTACTTTTTCTCAAAGAAGTTTCCATCGGACACAATCCTTGTCAAGAA

GAGTCCATCCTGTACAATCCTTGTCGACTTCTGTTTTTCAGTTAAACCTTTCTTATTGTG

GTTTGTCCGAGGCTGATATTCCCAGGAATATTGAGAGTTTATCCTCTTTACGATATTTAG

ATTTGAGTGGCAACAGTTTCCGCTGTCTACCCTTTGGTTTTACTAAGTTACACTTTTTGA

GGTCCTTGTATTTTAAAGACTGTGTGTATCTGCAAACACTCCCACCGGTATCAAATTTAG

AATATCTTCAAAGGCTTGAACTTGCAAATTGCAAAAGATTGGTGAAGATTGCAGAGTTGG

ACAACCTCCCTTCTATAGATTATATCGACATGGTTAATTGTAGCTCTCTGCAGAATCCAA

TCAACGAGGGCTTCTTTAATGCACCTGCTCTAGCATTTCCATCTAGAAAAGATGGTTATC

GGTGGTCTGAAATTCATCATCAAAGCAATGAGATTCCAGGTTGGTGCAGCAATCAAGTAA

CAGCTTCATCTATCTGCTTCACTATGCCGACAAACAATGAGGAGTATAAGTTTATAGGAA

TGATTCTCTGGTTTGTTCGCAAGTTTGTTAACGTACCCTTTTCTAGCAGTGCACGTGGCT

TCAGCTTTAGTATTGCAGAAAAAATGTTTCCAAGTTTCTCATGGCCTTTTGATTATCATA

GGTTACCTGCTGGATACGTAGAACGATCATGTGTACTTTACATACCTTACTTACAGTCAC

TCTTTGAAGGCTGTCAGATGAAATGCGGAGAGAAGATTGAAGTCCTCGAGTGTGGTAAAG

AAGATACCGTCAAGAAGATAGGGATCCATCTGCTATATTTAGACCAACATGGTAAAGTCA

CTTCATTGCCAGCAGTCTTGGATAATTCTGCTAACATGAAGAGAAGCCAGGAGATGAATT

GATTCTCAATGGACTGGAGTTTCAACAGGGCATATCAAGTACTCATTCAATCCTTGATCA

GAACGCAAACTTTT

>XLOC_037374 transcript=TCONS_00061201

AAACTCATCAGTCATCACCCTTTCTCTCCCTCTCTAGTCTGCAGCCGCCACACCTCCTCC

TCCCCTTCACAATTTCCTTCTTCGCTGGGTGCATTATCAAAAAGTGAACCTACTTCATCA

TTGAATGCAGAAGATATTGGATGGCAGCAACACAAACACATTTTTAGTTTATGTATTTCA

AACTGTATTATATGTATCTGTAGAAGAGTTATGTAATGCGGATGCTATGATATGTAATGT

TATGATTGTGTATTTGAAATTTACTTTTAGAAATATTTAGATGTCAATTTGTCTGGAAG

>XLOC_035827 transcript=TCONS_00058878

GTGGAAATGAGTTACTTAATTTAAACAAATTAGATTTATAACGTAAATTACTAGGAAGAC

TTTTTGACTTTTTTTAGTGGCTCCGTAATTTTCTCCTTCTTGGGTGGGAGTTGAGCTCCT

TAATTTTCTCCTTTTCTTAACTACTTTTTAGAAATAAAATCAACTTAGACATATTTATAT

TGGGTAATTTTTGGCTAGCATATGTGGAGATGTTCTATGGCTATTTGAATATAGGGTCAT

GGTATATATTTGGTTAGATATGAAATTTTCCGACTAACATTTGATTTTTGTTTTCATTGT

TTGCAGGTTTTATTAGGACATTTTGCAGGTCGAAGTACAAGAATACAATAAGAAAAGATG

AAAAATGCAAAAGCCATTGCCAGATCTTCTCAAGGAGTATGATATTCCAGTAGGTATTTT

CCCCCGAGATGCCACCAATTATGAGTTTAATGAAGAAACGAAGGAAGCTCACTGTCTATA

TGGGCAAAATTTACTGTTATCTCAACTGAAAAATCCAAGGTTCAAAGTATTAGTTAGATT

TCAAATTGTATTCCTTGCCTTTGCTTGTTGA

>XLOC_003284 transcript=TCONS_00005319

TGGGGATGTTATGAAAAAAGTGAGGAAAAAAGGCGTCGCTTGGAGGTCTGTTAGGCGCAT

AATATCGGTTATTGCTTGCACTAATCAAATAAAAACCAACTCTAATACAAATAGACTAAC

AGAGCATGGTCTTCAATTTTTTCATCTAGGTGGTTTATGTTTGTTTGATGCTTGCAACTG

CCTAAATTTGCTCGTTAAACTGGTAGTCATAATTGGTCCTAGGACCCCTTGGTCTCTGGT

AATGTGAATTCTCTTGAATTTAGCAGAATAAGGGAGAAGAAAAGGATATGCTCACTGAAC

TTCCAAATTGCTTGCTTGTCTCCATCCTTTCTCGGTTAAGCTTAAAAGATGCAGTTAGAA

CCAGCATATTGTCCCGCAGATGGAGGCACCTTTTCCTGCTGAACCCCAACCTAGTAATTG

ACGGTGCACATATCTTTGGTGATGATCAAGAACATCAGAGGTTTAAACTTTCTTCTTATG

AGGATTTCGAGAACGAAAGGCGTGAGTTTTTTAGGCGTGTGGATCAGTTCATGCAGCAAA

AGCATGGAAGACATATACGTTCTTTGACGGTGGGTTTCTTCATGGATAGTGAATGCTCAT

CTCATTTAGATGAATGGTTGAAAATTGCAATTGCACCTGGACTGACCAGACTTGAACTGT

TGCTTTCATCGGAGCAGAATATCTTGACTAAATTTTATGTTGACAAAGACCGCCTATACA

ACTTCCCCTACTGGCTCTTTTCAGGAACAAATACACCAGCTTTGCGCCATTTACGGTTAC

ACAGTTGTGTTATGAGGGCACCAGGCGACTTCAAGGGCTTCAGGAATTTGAGAAATCTTG

AATTGAACAATGTGATTTTGGCTGAAGACTTTGCCAAAGAATTACTCCCTAGTTGTTTGC

AACTTGAACAGCTGACATTGGTTTGGTGTTCGATAAAATCAACCTTATGCTTTGATCATC

CATATCTGCCACTAAAGCATCTAAAATTGCTTCATTGTCTTGGTAGACCAATGATTAAAA

TATCTTCTACGACTCTCACAACCTTTGAGTATGATCGGGATATGATGAAGATTGAATGTC

TTTATGCTCCTCAGCTGAAAGAAATGTGTTCAATAAACACTTGTTGGAGGAGTCCTCTTT

CACTCCATTTCTCTAGACTTCCTAAGCTAGAAACCTTACTTCTGTTTGTGGACCATCACC

GAATGCAAATTCCAAGTCCCATCTCTCTCACAAATCTCAAGCAACTGAGTTTGTATGCGA

TGACTCCAGGTGGCGATTTTGACCTATCATGGGTTATATCTTTTTTGAAGGCTGCACCAC

TTCTCCAGAAGCTTGCGTTGACGTTATTTGGAGATGTGCCGTGTGCAGCTCAAAAAGATA

TACGAAGACTGGACGGGATCCATCATGGTCAACTGAAAGAATTTGAGATGCATGGATGTG

CTGGTAATTGGTATGAAATTGAGCTTCAACTGTTTGTATTGAGTATGGCAAGCAGATTGA

AACGATTAGTTATCAGTCCCTCACGGCTGTTGTATGCTGGAAATGGACAATGGACCGATT

TTCCTGTACCCAAAAAAGCTTGCTGGGATCAATCTGGCCAAGAAATGGTCAGGAAAGGAC

TTCAAAATCACATTCCTTGTGGTGTTCAGTTGGTCTTTCTCTAATCAGCCTTGGTTTAGA

AGATGTTTGTTGAGCCTCGGAGGAGGATTTACGGTTCTTGTCCTCTTTTTGCTAACTGGG

TTTTGGATAGATAATTATGTAAAGTTTGGGTGGGGTAGTCACTTTTTAACGGCTCAATTC

AATAAATAACCGGCGTTTGGTATGTTTAAGAATTATCACCTCAAACTTTAGGCAAATTTC

ATGTAGTGCTGAACAGAACTCCAGGTTAAATTCAGGAGTTTAATGAACTCCATGAATTGT

TCCTGAAGTTAGAGCTGATGTAACTATTATTCTAATACTCAACAAACGGCAGTTGTTCGT

GGA

>XLOC_011162 transcript=TCONS_00018207

CTCTAATCCTAAATCCCTAAAGCACACACCGCCTCTCTCAGATTCTCCTCTCACGAACCC

TAGCCACCGAAACAGTCCAAGACACCTAACGTCATAGAGGCCCCGCAACATATCCACCCC

ACATCGCCGGTTTTTTGTATTCTCTCTTTTTATTTCTCAAGTACAATTTACTTTTGTGCT

TGACAAATAAGAAGAGAGGATGTACAAGTCCTTAAATCTCATTCGTCCGTTCAAAAAGAG

ATCGATTCTACGCTTTGGATCGTGAAGTATTGGCGAGTCCAAGTTTTGGCGTCATTTCAC

TGCCACACATAAGGTCACAATGAGTAAAAAATTTCACAATTTCGGAATGACTATGGGCTG

GTGGAGTAACTTGTCACTAGAAGAGCAAACCGAAGTACGTCAACACTTGGGTCATTTGGT

ATCACTATTAGAAATCGAACCGTGTAGGGAGATGATTGAAGCATCAGTGACATTTTGGGA

TGAAGAATTGAATGTGTTTAGATTTGGGGATATTGAATTAACACCTGTGTTGGAAGAAGT

AGGAGGGTTCGTATCACACTTGGGTTTAGACAAACATCGTTGGCAAGGGGGCCGCATACT

AGCACCTAGCATCCCCACAGCTGAAGAGTTTCGTAAAAAGTTAGGAATGGTGGCAAGTGT

GAATGTGGAATTTTTACAACAAAAGAAGATATCCTTTTCTTACTTCTACAATCGGTTTGG

GTATTTGAGCGCGTTCAACAACCATAAGAAAGAGTTTGAGTCCTACAGTGATTGGAGGAG

AGTTAGACCATTCGCATTTGTTATTTATTTCTTGGGAGTAATGGTGTTCCCCGAAAGTTC

GAGTCACGACATATGTTCAAGGCTCGTCATGGTCACCGATGCAGTATTCAATGGAATTGA

TGGTTACCATTACACCATAGTTTCTATGATAGTAGTAGACATTTATCGGGCTTTAGGTTG

TTGCAAGAGGGGAGCAAAATATTTCCAAGGTTGCAATCTTCTACTACAAGCATGGTTCAA

AGAGCATTTGGGGAAAGTAAAGGGAGTACAAGAACTGTCTAAACCAACCGATTCCAATTA

TTTGGTTGGAGAGCGATATGGGGTTCCGAAATCGAGGCAGCCAAGCCGCACGGAGAAATG

GGTAAAATTGTTTGAAAACATAACGGAGGACTCAATTCAGTGGATGTTCACTTGGTTTCC

TTCATTAGATTTCATCATTAGCTCTGAGAAGGCATCCTTTGTGATGCTTATGGGATTAAG

AGATCTCCAACCTTACCAACCATTGAGAGTGATGAGGCAAGCAGGGAGGAAGCAAATCAT

ACCATTGATACCAGACACAAAAGACTATCAGTTTGATTACACTGGAGATGTTGTTCCAAG

GGTCAAGAGCATTAGAAAATCCTAGGTGGGGAAACAAGTCATGGGAGAAGCATCGCTAGC

GCCCGATAGATACAACGATGGATGCGGACCGAACTACAAAAGCTGGTTACGCTCTAGTAT

AAAGGGAGATGTGGTAGTAGGTCCAAGTCATGGGAATGACATAAGAGATCCTGAGACGGA

AGCTCAAATTAGATACCGAGAATTCATGTTGAAAGCTGTCAAAATGGATAAGGAAAACAA

AGAAGAGATCGAAGTATGGAAGAAACGTACATTAAAGCTAGAGGAAAAGATAGAGGTGTT

GGAGGCAAAACTTGAACAATGGAACTCAGAGTGTGCAGAGATAGACTTTTTACACTTAGT

GGCGGCTAAGGGATATTTAACCAAAGCTATGGAAGAAGTTAAGGAGATTACGCGAGAAGC

AAAGAGAGCCAAGATGGAAAAGGAGCATGCTGCCTAGTCAAAGTGTCATTTTTATCTTAT

CTTTTGTATTTTACTTTTTCGAAAGACATCATGTCCTGATTTTCAAATGTTGGGAAATTT

ATGATAATAATTGGCGACAAAATGTGTATTTATTTTCTTTTCATAATGAAAAACTTAGGA

AGGCTATACGCCCTTGGTGCATAATAGTCCATTAGTAAGCCTACTTCCAAGTGTCAGAAG

CG

>XLOC_013067 transcript=TCONS_00021412

GCGTATACTTGTGTGTGCGTTTGGAGCGACAACAGTTCCCTGAGCATGTGTACAAGTTGG

ACAAGGCTTTGTATGGGCTCATACAGGCACCAAGAGCTTGATATGAATGTTGTCTGAATT

CCTACTTTAGCATGGATACACAAGAGGTAAAATAGATAGCCCATTATTTTTGAAGAAAAA

GGTAAGCATCTCTTAATTGTTCAATCTATATAGATGGTATGATCTTTGGTGCTACTTGTG

AAAACTTAAGTAAGGAATTTGCAAGCTTGATGGGGGGTAAGTTTGAGATGAGCATGATGG

GTGAACTCATTTTCTTTAAGTTTACAAATCAAGCAAACCTCAAATGGAACTATGATCCAC

CAACAAAAATATGTAAGGGAACGGCTTAAAAGATTTGACATGGATCATGCTAACGAAATT

GATACACCAATTGCCACTGCAACTAAATTAGATCTTGATGAACATGGTAATTCAGTTGAT

CAAAAATTGTATAGAGGCATGATTAGTTCCTTGCTTTATCTAACTGCTAGTAGACCTGAT

ATTTTTTTCAGTGTTTGTTTATGTGCTAGGTTTCAGTTAGATCCTAAGGAATCACATTTG

AAAGCTGTTAAAAGAATTTTAAGATATTTGAAACGAACAGGTTCCTTGGGTTTTTTGTAT

CCAAAAGGTGGAAATTTTAACCTTGTAGGATATATTGATGCTGATTATGCAGGTTTTCTT

GTTGATAGGAAAAGTACCTCAGGTATGACACATTTCCTTGGTGAGTGTTTGGTTTCATGG

GGAACCAAGAAGCAAAATTCAGTTTCTCTTTCCACTGCAGAAGCTGAGTATGTGGCAGTT

GCCTTATGTTGTTCACAATTGTTGTGGATTAAACAACAGTTGGAGGATTTTGGTGCTGAT

GTAGGAACTATTCCTATCTATTGTGATAACACTAGTGCAATTAACATGACTAAGAATCCA

GTGCATCACAGGAGAACAAAACACATTGACATAAGACACCATTTCTTAAATGATAATTAT

GAGAAGGATTTGATCACTATTCACTGATAAGCAAATAGTTGACATTTCATAAAGGCCTTA

ACTAGGGATCATTTTGAAAGGAAAAAGTTAGAACTAGGATGATTAAATTAACCTACGTCC

ACTTAGCTTCTATTCTAAAATTGGCTTAAAAAGAAATTTATTAAAGTCAATTAGAAGTTC

AGCACAGTCTCATACTTAAATGTATACTCTATGCCATGATTTCTACTTAAGTTTGCTAAT

TTTTAATGATGTTACCCTTATTTTCTTAGAATTTCAGATTGTGCAAAGTGATTAATATTG

GGTGTGGAAATTGTTCCTTTGATACATTCTCAGGTATGTACTATTATTTTATTTAAAATT

TATAAGAGCTAAAAGGCTATAGGTAACTAGACTAAGTAGAATCCCACTTGAGTTTCACGT

TCAGAAAAACAGTTCCATCAGAGAACCAGTTCCGAATTTCACTTGAACTCTCAAATTATA

ACGACTATTTATTGGGAGACGTCCAAGTGTGAAATTCAATCAATGAGGTAAGTGAATAAG

TTTTCTACTTAAACTCTACCACTAAATTCACTCCTGCATAATTGCACACACACCTTCTCA

TCTCACATTATTTTCTTCATGTTTTTCAAAATTTCAGAACGAATTTTGAATTATCTTCTC

AAACATATAATTGTCATGTCTCTTGCACCTGAAACTTCTTCTTCTACCCCTACCACTAAA

CAAAACCCAGTCACTAATCCAACTTCACCTATGGACACTACTTCACCACCACTCCTAGAA

AATCCAAACCCTCAAAATCCTAAATCTGAATTTCAGGGGGAAGGATTAGTTCCCTCAATT

GCTAGCACTTTAGAACATATTGAGGATTTAACTAGAGCTATGGTGGTTTATGAACTCCCT

ATGAATTATGTTGAACCACTCAACACTCTATATGAGAGTGAGAGAGATAAGGAGGAAGTG

AGAGGTTTAGGGGCTCCAATTCCAAGTTCTCCAGGTTCTCCCATGACTGCTCCTAGAGAA

CCCATGGAGGATGTAGATATGGTGGATGTTCATGACAATAATAGTGAGAGTGATGTGCCT

ATCATGGATCTTATAAATTTGAGGAGTATGAAGAGAAAACTTGAGAAATATGGTCCTGTT

GATCCTAAGAAACTTGCTATAATTAATGCATGGCTTAAGAAAACCAGTGAAAACTATAAA

TCTGGGCTTAAGAAGAAAAACCAAGCTGAAAGTTTTTCACAAGCTCAAGGAACTGTTCTT

TTGTACAAACTCCTACTAGTAGTGTTAGGAGAACTAGGATTAGTGTAAGGGTTGAAGAGG

AAAGAATAGCAGTAGTAGTGTTAGGATAACTAGGATTAGTACAAGGGTTGAAGAGGAAAG

AATGGCTGCAGCTCTTGAGAAGAGTAGGAAGGCAAAGCAAAAATAGGTACCTGAACTTAT

TATAGAAGCTGAGGAGGAGTCTCAGGAAGAACTGGAAAAATTGTTCCTTTGACTAAAAAG

AGGAAAACTTCTAAGAGGAAGTCTGCAAGGACTTATGGGAAGTGTTTTAGTGTTGGTCCT

GTTTCTAAAATAACCAGATCCTCTATGAGGCATTTGATAGATGAGGATACAAAATCTGAG

GAGATTGAGGAGGAACATAGTTAAAATGAGGCTGATAAATCTTTACATGATGACTTTGAA

AAGAGAAAATTGTCGAAGGGAAGGGTGTTTGCTGATTTACAGGAGGAAGGAATGATTTGG

TTGCTGCCACTACAGGAAATTAGACCTTTAACAACCACTTCTTAACGAAGGGATATGAAT

CCAGTCGTTAAAAGCATACTTAAGACGACCAATAATTTTTCCGGACCATAAAGTCAAATT

TTTTGTGTACTTTAGCGAAAGGATTTTATCCGGTCCATAATGACCATTATAACCGGTCGT

TAATAAAGAATAATTAGGCGGCAACATAAAAAATATTTCAATCCAACCAAAATTTTCAAG

AGCGAAACCGCCAAGTTATTATTAGAAAAAAGAAAAGAAAGTAACGCTAAAATTTTACTC

CACCACTAGGGTTAGCAAAACTTTTTATCCCATCTTTCAGCAAACAACCCACCGTGAAAA

TAGTGGCTATTTGGCTAGGGCTGGAAAATTTTTTTTGAATCTAGATCCTGACATCCACAA

AAAAAAAAAAAATGGTGTCGTTCAATTTCTTGAATATGGTGGTACTCAAGTCAAATTAAG

CGAGGAAACAGCTACAAATTGGGTGAGTTTATTTTTACAATGAAGATTTTTTGGGCATAG

TCGACAACTATTTACAGATTTTATCTTTCATTAAAGGTTTTACATATTTTCCCCACAACT

GTTTCCTAATTTTTTGCTTTACAATTTTGTTACTCAATTTGATCTTTTAGGCAATACTAT

TTCAATAAACTTTGCCCGTTTTCTCCTTTTTTTTTTTTTTTTTGAAACCCCATTGGGTAC

GTTTCAAAATTAAGAAATATTTGTCAAAACATCTTATTGCAAATTCACAAGGAATTTATT

GATATGTGAAAAAGAAAATTTAGAATCCAATGGAAAAATGGGGACTTCAATGACTATTTT

TCCAATATAAAATAACGTTCTTCATAACCAAATACCATATATATTCTGTCATACTATTTA

AAACAAGGTTAATAATCCAAATATTGAAGACATAATGTCTTTCTGCTGCAAAGAAATAGC

TATCTTTCTCCTGTTTAAACGACATGTAAGTTTTTCTTATTTTCTGTTGCGCAATGATAA

ACCCTGCATTTTGTAGTTCTTGATGATTTTACAGAACTTACAATATTTGCTTCCTATACA

TATCGTTTTACATTGTAAATCTGTCCTAGTATTCCTTTGGGTAATATAATACATGTTTTG

ACTTTTCTATTTCAATGAAAAGACAGTAATGAAGCAAAATTGAAATTTTTATGATCATAT

ACTGAGTTTTGGTTCTCGAAATGAAACAAGAAGCTTCAAGTTATCTACCAGACGGTCTAA

TTTCCTAAACTATTGCTTCAGTTTCAACTAATTACTTTTGTTTCTGCAGTACAGACCAGG

GAGGAGTACACAACAGAAAAAAGAAGTAAATGCAGTTAATAGGAAGATGGTGGTAATTCA

TTATTGTTTGCCTTAATGAGAAATATCTCATTCCTTTACTTTTGAGGTACTGCTTTGTTT

ACGGAAGCTGAAAAATGGATGATTTTGATGCTCAGTTTGCATTAGATTGTTTGAGAGCTG

AGGTTAGTTTTCTTGCAATACTTATTTTCTATAAGAAGAATGACTTCTTTACCTCTGAAA

GTTATATGATGTTAAAGATTCAACTTAGTGTCAAATCATGTAAAGTCTGAGGGCTCATGA

GAAATATTTAATGTCCTAAATATCAGATGCAGCTAATGTTTCTTTGGCATCTAATGAACA

TGTATAGTCGCTTTTTTGTCTAATGGGATAATGCAATTTCATGCAGAAGATTGTTTCTTA

ACCGAATCAATTACTGTAGATTGAAGAATTCAGTATAAAATTTGTTATAGATCAGCATAT

CAGAGTGGAACAGAATGGCATGCCAGATGGTTTGTATTATCAGCTTGTTCTCAGGTTAAG

TCATGTTGTTAAGTGATAAAGTTAAGAGTAAGTTGACTTGATTTTGTGTTGTCATATTAG

AAGAAGAGTAAATGCTGTTGCCTTTGGTTATAAATTAAAGTTTCATAACTATGGGAAGTG

AGTTGAATTCCTTTTTGATCATTTAAGAAGAAAGAACAAATTCCTATGAGGATTATGAAC

TTGAGTTTCTTCCTTCTCAACTATATGCAGAAGGTAATGTCTTTTATTTCTTTTCTCCTG

ACAAAAAATATTGACCTAAATATGATAGACTAAAGCAGTTCTACATATGGTACAAATTTT

ATTTGTAATGTGTTATTTCATGGAACTATAATTATATGCACAAATATGTATTCAATCATT

CATCAATATTTCTCTAAAATGAGCTTTACATATAGTTTTTGAAGTGCAGTGTCTCTTTTC

ATTAAGTAGTGTACTGATGTCTACTTGATGTTTTGAAATACTAGTAAATGGTGAAACTAT

TTACCTTATATCGGCTGTTGCACTTTTCAGATTGAGCATTCTAAATAGGAATTGACTCTT

GTTCATGCTTTGTAGCTGCGGATCAAGAGTATGAACTTGTTATTTTTACTGATGAAGAAT

TTGAAGTCGGAAGAATGTTTCCATGAAGAAAGTGCATGGAAAGTTGGAGTAGATGTTTTG

GAATTGCTAAAGGGGCCTTCTTTGTATATAGCTTACATCATTTTCTCTTTTTAGGAATCA

GAACGTCATACAATTTGGGTAGATATATTGTTTTAGTAATGTGGCTAACTTTTTCTTTGT

>XLOC_036372 transcript=TCONS_00059721

AAGGAAATAACTTTTAAAACACAAAAATAAAAAATTGATCAATTACCAATTGGGTGACCA

TGTTGTTATCCGACTCAAATGAAATGCTATTACTACTATTTCCACTCTCTTGTCAACCCT

CTCCACCACGAAATTCTTGCCACTAATATCAGTTCATTGAACCTTCCAAGTTCGTCGTCG

ATAGCTGCATACCCTTTCTTCATTCCTCATGAGTTTGACTTACAATCTTCATTTCCACAA

TACATTCTCAAGTTCTTGCTGGAGAATACCAACTTTAGAAAACAATCAAAGAGCACCTCG

AAGACTGCAAATTGTCAATATTGCCCATACGAAGAAATGCTTGAGGTGCAATATTATTTA

TGAAGACAAAGAAAACTCCCCTATAGCCTGCTGTTTTCACGGCCACACCAATGGAGATAA

AGGACTATTTTCCCTGGCGCCACCTCACCAAGGAATTGATGGAGAATGGAGTGATCGCTC

TGGAGTAATCGTCTATAAATGGAATGAGAAGGATAAGAGGCCAAATACAGGTAGAAACAA

CTGGAAAAAAAGATGGAGCTGTTGTGCCGAGTATGATGAAAATGCTCCACCTTGTAGGCG

AGGATGGCATGTCTCATACGATGATGGTTTTACATTATACTAGTCATATGTGATCACTAT

ATGTAAATTTGTTTCTGTTGCCTGGGGAATGCTATGACAGTTACAAAGCGAACTCATTCT

ACTGGAAATACGCCTCCTGATCACTGTTCCATTCAGGCGTCGGTGCATTCACAGAACGAG

CATACAGCTTGTTCTTAGCACAAGTAAGAGTGCCTCGACTCCTTCAATCTTGTAATCATA

ATTCCATTGTCCATCCTCCTCTTTCTTTTCTTCTGAGGTCACTTTATCAGCATTCTTGCA

AATGACATCTGATAAAGCAAGTTGATCAAGACTACATCTATAGATGTATTACCTGAACCC

TGCAAGGAAAGTCAGATATTCTTTCTCAAGGAAATTGAATGGGGCCGTTGACAGTTTGGA

AAAATATGATGCAGTATTCATATGAGATATAGTAGTCCAATGGTTTTTGAAATTTCTCCT

TTGTTTGCCTCAATGGCTTTAGAGAGACAAATCGGGTACTACTTTTATGAGAAGAAATAT

ATGTATTAATGCTGCTAGAAGCACTAAAGTATGCAATGATGCTACTACTTGTACTACTTC

AGTTTTAGTATCTACAGATTTCCATATCTGTACCAAAAAGAGGCTGACAGGAAGAGTTGA

G

>XLOC_032637 transcript=TCONS_00053640

TTTGTGTGTAATTACAGTAATAGATCTAGATGTTAAATATCTCTAATTAATCGAAAGATC

TAGAAGAAGTTTGATCTAATTAATTGTAGTTAATGTAATTTTGTATGTGTAAGTTTAATT

AGTAAGTAAGAAATTTGCAACTTAGAATATGTTGTAAGCCTTTGTAAATTAGTTCCTTAT

TTAGATAGGTTAGGACGTAGGAGATTTCTTGTGCTGAGGGTCTATCAGAAACAGCCAGCC

TCTCTACTAAGTCTGATGTAGGGGTAAAGTTTGCGTACATCTCACCCTTTGCAGACCTCA

CTCGTGGGATTATACTTGGTATGATGTTGTTGTTGAGGACTTAGGAGATTAGAGTAACTT

AAAGGTTCGGTTTTTTCAATCTTGAGTTTGTTGTTTTGGACAGAGAATATGAATAGCGCA

AAAAATTAGTTGAAAAGAAGAATATTTATTGACTTTTATCGTTCTTTTGATGCTTTAAAG

CTGGAAAAATATTGTTGACTTGGGATGTAGCTGTATACCTATTTTTATTATTTGTAACGT

GGTGTCTAGGTCAGCTTGTGCACGCCTTGATAAATTTACTACTCATCAGTGCATTAGTGC

AAGGTGACCCTTGCTTACTAAGGTTAGAGCAGATGTGCTCACGCTAAATGTTGTAATTGA

GATTGGGATTAGGCTCTAGGTTGTTACTTCTGTGTTATGCTTCAACCACTAGGCCATTTC

TTGAAGTCATATACTTGTATTTTCTGAATAATCTACGACTTTGATAGAATTCTTTAAATG

CATGTTTTCTGTTGGTATATGTTGATATCGAATCTTAGTTTTGAGTTATTTAATTTCAAA

CCTAATTAATTGGTAATTGTTGCAGGCTAGGCTTCATTTTTAATTTATGCTACACAAAGG

AAAACCAAAAAGTATAATTAACTAAAAAGGAAATTTCAGTCGGGGAATTCATATTTAGGT

TTGGAGCTAACTGGCAAGTTAATGGCCCATGTTTTGTGGTATGGCATTTTTTTATTTTGT

ATACTGTAGTCTGATTAAGAGCTACTATTTTCTTTTCCTCTGTATCTTGTGGCCGCTTTC

TTTTGTGTATCGGGAAACAGAAAATTGACAATTTCTGTTGAGTGTCTGAAACTATGTTTT

AAATTGTAATGCTGTTATTCTTCAAAGTGCAGCGCCAGTGTTGTCCCAGCAAACATGTTT

GGAAGTAGTTACTTTTCTTATTCAGTTAGCTGATGCAGAGTTGATTGTCTCTACATATTT

TTTAGATAGTATGCTCTACTACAAGCTGAGAACAAATTGTGTCTGGAAAAAATGATCTAC

TATTATATTTGTTTCAGTTTAGGTGTAACCCAAATGGTCTCTAATCAAATCTATTGTAAG

CAAATGCCATATGTGACTTTAGTCTGTTTCATTTTGTATCCATTCAGTGTGTTCAGTGTT

CGTATGCCAGAATATTCAGGTTGTCGTGGAATAAGCTGCTACAGTTGATGTGATTGATGC

TGCCCTTTCCTGTGGTTGATATACCAGCTGCATATTTGTCTCCTTTATGAATTTGATATT

TACTATTTCTCTTTTTCATCTGGTAACCTCTTTTCTTTATGCCAACTATGTACTTATCAT

TCTTTCCAATATTAGCAAAAAGTATGAAAAATAGTGTTTATGGCTGGACATATCAAGCAA

GTTGCATGGTTGATAGAGATTGACCATACAGGAGAGACACTTGAATATTAATTATAAGGA

TAGGTATTCTAATATGAATAACATAGGTTGCTTACAGTTCATGTCAAAATTCATGCCTCA

CAAATAAAAGTTAATGGTTACTGTTCCAGAAACATACACATAATTTTTTTCCAGTTTAAA

CAATTTACTGTTTCAGCTGGACGAAGATATATTCTGTCCTTGGTATTCTCATGCACAAGG

GTGAGAGCATATTATTTGCTTTAGAGTTTACTTGCTAGGTGTTAACCAAACTTTCCCTTT

ATGTTCTCACAGTCAGAAATGAATCATTCCTTCCAACCCTTCTAACATCTGTTATGCTTT

TACTTTTATCTGAACTGCATGTGAAGCTGAACTGCTTTAGTTGTTAGAAGGGAATTTAGC

TTGTGCAATGACATATACAGGTAACAACTGTCTTCTAGACCGACTCTTAACCTGAAGCAA

GAATGAAATGCGTGGTTGAAGGCTACTATGACCACACTAGCAAAGGATGGTATACTAGCC

TGGTTACCATGGGAAAACTGATATGGCGATACTACAAGTTAGACTGCCTACCTGGGGCAA

TGTAAAATGTAGTTTCATTTTACGTAGTTATGGTAAGCCATTTTATGTCGTTCCGTTTTG

TTGATAGTTGTTAAGTACCCTCTTTTTATGGACAAATATAGGTTTCTTAATTTTGTGAAA

TATGAAATCATTATGTATAGCACATATGATGCTGGGTAAATAGAACACGGAAAGTATTTA

TCTTGTTTAGCTAATGTTATTATTAAGGGCTTTACTGATTTCCTTATTTGCTTTCTTTCT

ATTAGTAGCAATGGGTAAATATTTTACTGTCAGTGTCAAGTGGAAAGAATATCAGTTGCT

TTAGTTATATCTTCTGCTCTTCCGATTTACATTGAATATATCATGAGTTGATTTACGAGT

AATTGGCCAGTGTTTATGCTTCTTTGGTGTTTTATGCAGCCTTGTATTTTCAAAAGTTAT

TTGGTATCCACATATTCAGTTTCTCATCAATTTAGCTGAAGAAAAAAATGTTATTAGGTA

TCCACATTTTTCATTCTCTCTTCAATTTAGCTGAAGAAACTCATCTAGTGATTTTAGGAG

AAACTCCAAAGTACTTCCTGACAAATATAGATCAACAGGATGAGGTTGATGAGTGATATT

GATACAACTTCATAGAGGAGCGAACTTTGAAAACCTAACTGAAGAAATTGCTCAATTTGA

TCCATTCGCTCCTGCAGTCTGGCAAGGTTAGAGGGAGCTCTTACATGGAAAGAAAGAATT

ACAGAACAAACCAGCGGTAAATCTCCTGTTCGAAGGTCTTTTTTCATAAAAGATCCAATG

TCTAAGCTAGAAATAACATGGAGGTCTTATTGAACCAAGCAAAAGCTCTTCTGCAGCAGA

CCTTCCAGATTCCTCAATGTCACAGATCCAGTATGGCAAGGTGAGAATTTTATCAAGGAT

AAAACTTCTGCAAGTAATCCCAATTCACCAGAAGCTGCTTGCTATGTTCTGGTACAAGAA

TCCCAGGATTATCAGACAATTCTGTATGTAGTCGAGTGGAGGTACTCACTGAGATGAGAT

AAATAGAGAGCTGATGGACGTTTTAGTGACCCCTGTCAAGTGAGTCGTACTCCATAAATA

TGATATCTATCTATCGATCGATCTAATGTGATGTATGGAAAATATGACATAGGTGGATCT

ATTGAGCGGCCAAATCATATAGGCCAGAGATGGACATCCAATTGCTTCGATTTCAATTAT

CCAGAGATGATATATTACCCGTGCCTCATTATTATGGACACTGGGTTTCCCGAACCTAAC

ATATGTCTTGTAAATGCAACCAACAACCCGAAAATGAGTTTGGTTCATTGGCAGGGAAGC

TTGTATTAGCATGAAGTCTTGTTACTGTGCATTAACTACATTTGTGCAAAAAGTGCTGCA

GGGGTCTATTACCCACTCATATTAAGAGGAATTTCCCCATATAAATATGAAGCTGATAAA

CTAATTATAATATTTAACTTAAATGTGTTTTACATGTTTGTTTTGCAATAACG

>XLOC_027768 transcript=TCONS_00045674

GATAAACGTGTTATTTTAGCCTTTCATTAGGTTGGGACTTCTATTATATTTCTCATTTCG

TCACTCTATGGTTACATTTTCTTCTTGCTTTTGAAATTTATGTCCAAACATTTACAATAA

AGATATATTTTTCCCTTTCTTAGATAATGAAAGAGGTTGTGATCAGTCCTCTATTTGTAT

TTCTTCCCTTAGTCAAAACCTTATTGCGTCCTGACTTCTCAATTACTGCACAAAACTGTT

GGGTAAGGAAAGGAGGTGCATTTACTGGTGAGATTAGTTTGTGGGTTATAAAGTTGCATA

TGCACTTTCTCAAGGCTTAAAAGTGACTGCCTGTGTTGGGGAGACTCTTGAGCAACGAGA

ATCAGAAGCTACGGTGGTTGTAGTTGCAGCGCAAACAAAAGCAATTGCAGGTCTATCTTT

GATAGTCCAGTGAATGGGGAACTAAAAGCTTTCACTTATGTATTTTTGTAGAAATGTTGC

TTTTTTGGAATCAGATGCCAATGCCTCCCTGATCTGACTCTTACCTGAACCCACTTTGCT

ATCTTAAATGAGACTTGGATAAGTATGATTGGACTTGGGACACATAGTGCTAGATGGATT

TCTATACAGACATTGTTATTATTAGTATTTACTTCTTGATAAGTGACGACTGTTTGGTTG

GAGCATTATCATTAAGTTATGTTGTGTCTACTGTTTATTCTACCTACTTTACGAGCTGAA

GAAACTCTTGCTTTGCTTATCTCCTCCTGAAATGAGATTATAAATTAAGTTGGTTGTTTC

CTGAGGGAATGACGTGGTTGAGGCATAGGAGTAACAACATAGAGATCTCTGGTTCAAATG

TCAGTTATGACACTCAGTATAAGCCTATTTGCAGCCTTGGCGGGCAAGTTTATCCCACTC

CTATTCTTGTAGGCTGTAGTAGGCCGCCCGATGGATTTGTTGATGTGCAAGAAAGCTGGC

CCAGATATCTAAAATATGCCAAAGTTTTTCCTGGTCATGAGTTTTAAAGTAGATGATGCT

ACTGCCACCATAGTTTTGATACTTCCACCAGCCGCAAGCAAGGCCATCCACTGCATCCCA

CTACTGCTGCTGTGGCAACATCTCGCTC

>XLOC_020349 transcript=TCONS_00033401

GGAGTAACTTAAACACAGCTGCAAATAGAAGTATAAATTGCTATATTCATCATGAGCAAC

AAAATCTTTCTAGCCCTCCTCTTTTGTTTCCTCCTCATTGCCTCCAATGAGATGCAAGGA

GGAGAGGCAAAAGTTTGCCAAAGGCGTAGCAAGACCTGGTCTGGGCCTTGTATTAATACA

GGCAACTGCAGCCGTCAATGCAAGAATCAAGAGGATGGTCGCTTCGGAGCTTGTCACAGA

AGTGGAATTGGATTTGCTTGCTTCTGCTATTTCAACTGCTAAATGAAAAGGAGAACACCA

CATGTTTATCTCTATTTCAATGAGTGTATTATCCACTATTTATGTAACCGTGTTGTGTGT

TGTTTGAATAAGGGCGTCCAATTTACTAAAATTGGAAGTAGCTCGGCTTGTTGTGTTGGT

GTGTGATTGTTAAACTTTGTACTATCTGGCCAGCTTTTGTTGGTCATTTACTAAACGTTG

AGTGTGTCTATTTGTTGTCTTCTTTGTGTTTCCGG

>XLOC_002537 transcript=TCONS_00004089

ATAGTGTGTGTGTTTAGGGATTGGCATGGCACAGGACGAATTACTTTCTCCGACCCGCGG

GTCGCTGAGACTTGCTCAAAGGCACCCGGCGACAGAAGATCCTCCGCCAGTAAGGATGGC

CCGACGGTCCCCGAGGCTTGCTCTGAAGACGAAAAGAACAGAAGATCCTCCGCCAGTTAG

GAAGCCCCGAAGGAAGACCCATCAATGGAAGACGAAAAGGCCATGGGCACCACCTCCTAA

GCCATATTATGAACGACCTCCTCCACCACCGGGACCGATGCCGCCAGACATAAAGGAGCT

AAACGATCGTTTGTATTCCAAGTATGGGATAACAATAAGCACAGGTTTCTTCGAGCCCGG

CCCCGAATTCGATCGCGATATGTGGGTTAAATACTATAAAGAGCTCCGTGAATCTGATGG

ATTTGATTTTACACCTTTTCCTGTTGCTCAACGCTCCATCAGGGTAACCGTGTTTGACAT

GGATTATATAGCCACAACGCGTGATGGGCATAAAGAATTGAAAGGTTATGCAACAGAGGC

ACTCGAAGAATACAACAAAGACAATAAAACCACTTGGGAGTTTGACGACATTGACTACTT

GAATGGAGGTGTCTGTGGTACCTTAAATTACTATATTACCTTTACCGCCAAGACAGCTTC

CCGCAAGCAAACTTTTCAACTTAAGGTCGTCATAACTCCTGCTCGTCGTAGTATCACCTA

TCCTATCTGCAGGGAAAAGAAGAATACCACAGCAGCATCAGAAAGCACCTCTCTACCATT

AAAGGCTAACAACTTTCTGTGAGGTTCATTCGACATGGTCATTAGCCCATCACTTTATGC

TATTTAAGTTCTCTTGTAATTTAAAGTGGCAGTAAGATCGGAGGGTATGTGACAATTGGA

ATATGCATAATTTAGTATGTACCAGATTTTACGTCAACATGTTTTATTTTGGAAGTAAAA

TGCTCATACTTAAGTCTTGTTTATGTTTCACAAATGGAACGTGTCAGTTATGTGAGACAC

AACTAATATTATGAATGTTTTTTTCTTTTGTTCTAAAT

>XLOC_027142 transcript=TCONS_00044692

TCCTCTTGTGCATCTTCCACATTCAGCTAGAAGCAAGTATTAAAAATGGCGACTTTCAGG

CATTGACTGGAAAGCGTCTACCTTCAATTGATAGGAAGAAAGATGGTAAAGTCTCAGAAG

AGGAGGAAATGGATGATCATAAGAACTTGCCTTCATCACAAGAAGAATCAAGAGACGAGA

TAAGGAGGTGGAAAATTTCTAGGAGGCCTTCCAAAAATCCTGAGCACTTGAATGAAGAGG

GAGAAGATCTTAGTGGAAGGAAACACTAGAATTGCCTGTGTAAGGCTAAGTTTTGACGAG

CCCAAAGCCAATCCTTGGATGGACACAATCAGAATTTG

>XLOC_015487 transcript=TCONS_00025349

TCTGAATTCCTTATCATCTCTTCAGGAAAATTAATTATAAAAGGGTCAACATATAACATA

TTGATTAAGCACCACATCTTTGTAGAATTCCCTTTTCAACAACTTGATCTTCTTACTCTC

TTTCTAGCTTTGCAAGCCATGAATCATAAAGCATTCTCTTTGTTCTTCATAGTAATTTTT

GTCTTAAGCTTTTGCCTATCCTCAACAACCTCTCGAAGAATATTGTCATCAGGTAGCAGC

GGCAGTAAAGTTGCAGCGCATGGCCGTTCTTCTACTACGGCTCCTGGAGCATATGAAAAT

CCGCCTGGACGTGGAAGTGGCTCTGCAGGAGGTCATTCGTCTCTTTCCTATAAATCATTA

CAAAGATCAGTATCAGTATGCAACCAGAAAATATATGGCAGTTGTATTGGCTCTCAGCCA

ATGGCCAAAAATAAAATAAACCCATAGAGGCACATCAGGTAGTGTGCTAAAAATGATTCT

TTAGGCACGACTTTGCTATCATGATTTGCACTAAATTGTGTAGTTTTTATCGTTACTATT

ATTTTATTTTATTCTAGAATTACTGAATGCGATGTGTAAACTTTATTTTCCCAAGCAAGG

AAAAGAGTTCTGAAGATTTGTTTAAATAAACAATGATGTTTGGCTTCTTTAGTTTTACTA

CAGCTTATTTAATCAAATTTTCATGAAACTCTGTTCGAAGAAAAAAGTTATGTTAGGGGT

TGAATTTGGGGAAGAAAAAAATACTAATAGAGTATTCTAAGGAATTGAGCTAAGAACAAC

TCCCGAACAAAAACTCAAACTGGAATGGAATTAATATGGAACAGTTCCATCAGTTATTTC

AAAGATTAATGTTTTTACCATCCCCTATTATCCAATGCATTCCTTTGTGACATATTTTAT

CCCATTTACTGATGTATATTCTTCATATGAGAGATATTGCTTGTAATTTTAATAGAATCC

TTATATTTATGCTAGCTAGAGAGGACTCTATTGCAACTGTTAAACTTGCCATACTCCTGA

CCAGGAAAAGTGAAGGCTTTGCCCTTTCATTTACCTTGCCTTCCCACTTTTCTATGATTT

GCACTGAATTGTGTAGTTTTTTTATCGTTACTTGTTATGCATACGGAC

>XLOC_004016 transcript=TCONS_00006538

TGTTAGTGTAAAAACGGTGTCGTTGCTACTGGTTCTGTAGAGGGTTCCGAGGTAGGCTAG

TAGTTCAAAACCTAAATCTAATGGAAGAAAAATTGTATTCCTTGTGGGATTAAAATGGTA

CGACTATAAGAAGAGCGCAAAGCTTGTGTGTTACAGGTTGGCAGTCAAAGGTTGAGAATG

AGAGTCGCTGGACAGATTTGTGGATTAAGTTGGGAAGCAACCAAACCATCAGCTTTCCTC

TTGGTTTTCAACTTCATGTTGATGTTTCTGTTAAGCGTTGTTCCTCATCTTTCACTTCTC

CTCTTCCTTCTACTGCAGACAGCATCTATGCTTCTGACGGCTGCATCGTAGATTACCGAA

CTGTCAAGGTTTTTTGGATTCCTTACGATGATAATATATTTGATGCATCTTCCTTTCTCT

TTAAATCACAACAACCTAAAGTGGACGACCACTACCGCCATGTTGGAGAATCATCATCAT

CATCCCGGATCTATAGTGGAGTTTGCGAGGAGCATGAAAAATTGTCTATCTTTGCCTTCT

CATCACTGGTCCATGACTTTGGAGATTTCAAAGGTTACTTATCGAATTCAACGAGACGCG

CCACAATTTGCATAAAACCCACAATGTTGTGGCATGGGAAAACGCTCAAGCATTGTTGGA

TTCACGAATTGACAGGTTTCAAACTTTCGAGCAGGGAGTATGGATGAAGAAGATGGAGTT

TCCGGCATCTTCTAATTCCTGGGATATGTGTGTGATTTGCAGAGAAGATTTTTTGCCAGG

TTCTATCGTTTCTTGCATTAACCATTGCTCTCATGTCTACCACGAAGCTTGCTTGCTAGA

GTGGATATTTAGAAATCCGTCTTGTCCATATTGTCGGTCTGAGCTGTCAAAGGAAGTCTA

ATCTAATAGTACTAGTACTTGTGCAGCCAGATGCACCATTGTTGTATGCCCTTCTTGGAT

GTTTCTATTAATGTTTATTGACAACTTATAAAATATTAAAATGGCAAAAGAAGAAAGTCT

AGTATGTCTTGATGTAATATTAATCTTACTCTGCACGTTTATCCTTTTCTAATGATGAGA

AGGCTTCACGGCCAACTAAACCACTTATCCCTATATATACAGAAGACTTGTATGGATGTA

GACCAAAATGCATAATTGATCTTTAGCAAGTCAATTCCTAAATCATGACTTCAAACCCTG

GTAGAGGTACTCCGCATAAGCAAAGAGTCATTCGGCTATAACGTGGGAACAAACTATTAA

GATAGCGTAGTCCTATTCAAAAGCTCAATTTTGTTTTCTACTTTTTCTACACATCATGTA

TGTAAGATCCAATAATGGAGTATTCATCTAATTAACTTATATTGTGGTTTCTGTAGAACT

CTGCACTTATGCAGGATTTGCCCTATGTAGCAATGTTAGCTAAAAAGCAAAACAATTGGA

TGCCTTTGGACATGGCCATCTGAACTGATATTGGTCTAAAAAATAGTTTACTACTCAAGT

TGATGGATATAAGTGCACAGTTCTAATGAGTTAGTCTAACATATACTCGTGGAATTACTG

CAAAATATTGTTTATTCAAATTCCCTATATACATGAACTACGGACCTTGTGAAGAACTAC

ATGTAGAGGCAAAAAGGGGATAACAATCTGCCCAATCTCTACTATGGCAGCAGTAGATGA

AGGTAGGACTCCTTATTAGTTATGTTAACTTCAGCCGATCAAATGGACAAGGAAAGAATG

ATGCTCACAAGTTTGGCGTTATAAGTTATAAGTAGCTACATGAAAAGGAAGCATGATGGG

GAGGATCTCAATGACGTTCACAAACACAAGAGAGGCAAGCACAAGAGCTCAAAGATTGAT

GAGATTGGTGCAATAACGGTAGCAGGCTGAAATGGTTCTATCATTTGTTTTATCCTCAGA

ATTTACAGTTGACAATTTGGAGGATCCTTTACTTAAGCTTCCGTTTGGACATAGTTTTTG

GGGGGAGTTTTTGGTTGAAAAAGTGTTTGTTTATGTTATTTGAGCAAAATTTCTTCCAAA

AACTCTCAAAAACTCAAAAAATTGTCTTGAGCTTCAAAAACAGTACAAACTAGCTTTGCA

TAAGATCTCAAAGGCTAGGGCAATGCCAAAAAATTTGTTCCCAAAAAGTATGTCCAAACA

CTTCTCATTTTCATATCAAATTTCACCCAAAAAGTGTTTCCCAAAAATTATTTGGGAATC

TATGTCCAAACGCTAGCTTAGTATTGTGAAAAATCTGAGGTACAGAAGTTATTGTCTTGC

TATATCCATTAACTGAAGAACTTTATTGGCATGCCCTATTGTTTCAATCTGTATTTGTCA

CATGGGCAAGACTGTTCCTGATGCCGTCCAAATTGGCTTAGTTACCAACCATCAAGAGCC

AAGCTTGAAGAGCACAAGGAATACAAGTCAAGGGCTCCGGAAGCGACTTGAAGATCATGC

ATTGGAGTGCCAAGGAAGGAGGACCACCTAGGCTACAAAGGAGGAGCGTCCGAAGCACAT

TTTGAGGCATTTCAAGGAGCTTTAGGAAGAGGCGGAGCGCCTACATTTCAGGATCTCAAG

GGGCGGACCGTCTAGGTGGCTGCTCACTCTCTGGACATTGAAGAGGGGTGGACTGCCTAG

TGGGGGTGGGCGGACCGCCTACCCCTAATAGTTGCACACGGGATTTTGACCTAAATGTCC

ATTTTGGCCCTACGTGGCTTGTATTGTTATAACCCACTTTGGGGGTGTTTTAGACATTTC

ATTAAGCGTAAAGGAAGACTATAAAAGTCTTACTTAGTGTCACGACCCAATTTAGGGCCG

TGATCGGCGCTAGACAAGACTAAGCC

>XLOC_006651 transcript=TCONS_00010807

ATCTATAGCTGATGAAAATAAAACTGAAGCGGGCTCCTTTAGAGCTAAATGAAAAAATTG

AAAAAAAGGAGGGAAAGTACAATGTTTAAAAGAATTTGAGAATAGTTGTGGGTTAAAGAA

AAATCAAAAGTTCTCTGTATACACAAAAGCACAGATCCAAAACCCAAAATTCTTCACCCT

CAACATCGTCAATTCTAAATATAAAGAAAAACCTCCACAGAAACACAATCCTCTCTCCTA

TACCTCTTCATCTTCCCCTTCAGAGCATCGAGCGGTACTACTCAATGATACACCTCATCA

ACAAGTAACTCAAATGCTTAGATTGTTCGTACACACATATTGCTATAATCAGATTTATGC

AGGGAATCTGTTTGATGAAATGCTTGAACGAAAAGATGTTCAAACGGTTGATGTTGTGCT

TGATCGTGGTGCCTTTTGGATATGGGTTCCCTACTAAAATTAGTATTGCATCTTTATTTA

CAAGGTGGTAAGATGTGATAAACTATAAGGATTATTAGTGTTTCTACTTATCTTGCTCGC

AGCCAACAAGCAATTTATGCATTCAACAATGAATTCAGCTACCAAGGGGACATCGAGAGG

AAGCTAGCATGGAGTCCAGAGTCAAAAGCTGCCTTTAACTAAATGGACCACTACACATCT

TGTACTTGTGTTGGAAGCCAGTTTTGGAAGATTGCTACCTTTGGTGGTGGATATACGGCA

>XLOC_000448 transcript=TCONS_00000764

GTTCAAGGAGGAATGCAATACTGAGAAGAAAATAGCATCTGCGCTGAAGTCATTCTTGGG

AACATCCATGTTGGTAGAGAGTGAAGAGAAGCTTCGACGGAAACTCTTTGATCTTCTGCA

GGTGTAGTTGTACATTGCATTTGTGTCAAGCAATCTTTTTTTTTTCTTTCTAATTCTTCT

TTTTAAAATTACTGTTTTTCTGCGTTGATGCTTATTCTACTAACACTTGTCTGTTATGTT

GATCCCCCCAACTTTATCTCAGAATATCACACTTATTAATGATTCAGAAGATCTGAGAAA

ATTTTATCCTCGCTTCAATATTGAAGATACGACAAGCTTCAAGGATTTGGATCAGCACAG

CCAAAATGTCCTAAAAAGATTATACTATGACTACTACTTCCACCGACAAGAAGGTCTTTG

GCGTCAAAATGCTTTGAAGACTTTGCCTGTTCTTTTGAACTCATCAAATATGCTCGCTTG

CGGGGAAGACCTAGGCCTAATTCCCTCCTGCGTTCATCCTGTATGCCGCTCAGTACCAAC

GTTCCTCTAAGAATCATGTCTCTTTGTTACATCACTTCTTGCATCGTAATTTACAATT

>XLOC_012353 transcript=TCONS_00020198

CCCATCCTTAATTATACCTACGTGAGTCTGTATCCAATCCACATTCAATGAGAAAAAAAG

GTCTGAAAGTTTACAGCGACTCAACTTACTCAACTTCTGTAAGTATTTTTTTCGACATCA

ATTTTAGGAGTATGTTTCCTATCTTTTTTCTTACTTTGATTCATTAACACGAATATTGTG

GTGTTTTCTTGATTTTGTAGAGCACTCTATATCATGTTCTGCTCCAATCTTGATTAAAAA

AAAATATAAATATATAATACTCTCACAATTTTATTTTGATTTTGCAGTGGATATGGGTAA

CTTAAGCAAGAATTACATGAAAAGTTAGATTCATATACTTCAATGGCAAAGACTTAAAAA

AGTATTTGATTGTTAAGCTTATGTGCTCTTTATCAGGCGAGTTACTCTTCATGATGTCTT

TTTTTTTGTTCCAGTATCTCTTCTTTTTATTTTATCTTAGTTAAACTTTCGATCATTTTG

ATATTGGTTTAATTTCTTATGATCATATTCAGCTATTTGAGTCTTATTTCTTTGATCTTC

TTTGGGATTAATCATTTATAGGTTGGCTTAAATATAGAGAATCTCAATTTTATATACCTT

TTCTAATTCCAAAAAACTTTCACTCTTTTCTGACCTCTCGAGCAATTAGGTTACGGATTA

CATAAATCTCTACTTCATCAGATATACAACGTAGTGGAGGTTTCTCAAAATGTGAGACTA

CTTCCAATTATGGTTGATGAAAACAGTAACAATGCTTACTCAAAGTAGCTCCAATAGAAG

AAAGAGTCCTGGAGGAAAATAGCGTTAATGAAAATGCCAAATGAAGAATCAATTCACTTT

TTTAATCCAAAGCATGAAAGGATCTTTCACTAAAGTTGGGGAAAAGACGTACACGAATAT

AGCACCACTTTCACTGTGAAAGTCATGCTCAAGGTAACTATTTTTATCATAATATAGCAC

TATAAAACGTCATTGCAGTGCTGAAATAAAGTCCA

>XLOC_007096 transcript=TCONS_00011542

CTTCCCCCATCAGTAAAACCTAAACTTCCCCCATTATTTCACGACTCTCCCCATTACCTC

CTTCCAATGACCCAAGCAAGAAACGTACACCATTATCAATTTTTATGAGAGTTGGACAGA

GCCCCAGCACGGCAATTTCGTTAGAGGTCTTCAAGACTTCAATTGTTATACTGACACTAT

GCAGCTTCGGTCAGGTTCTTATCCTACAAAACTGCTGCAATTC

>XLOC_032914 transcript=TCONS_00054103

AACCACTCCTCAAGATAATCTCTAAAAAATTAATATACTCTTTTCTCTCAAAATTATCCT

TATCTTGATCTCTGTCCATCGCCTCAATTCCGGACGCTGGTAATGAAGATTCAGATCTGA

AACTTACAATCACGAATCTCAATGGTTCGCCTTAATGAGTTAGTGTTTTGGACACTGCCC

TTTTTGTTATGTGCTCCTCTCGTCCCAGGTCAGATCTAAACTCAAAATTAGGGAGAAAAA

TTAAGAAGAAGATAACAATGAGCTGTTTTTTGTATTATTATGTAATTTCAGGTATTTGCG

GAATTTTGCGATTGCCTTTAGGTCTTCGATTTTATGATTTAGTATCCTCTTCAATTGATT

GCAACTTGCAAGTTGGTTAGAACTGCTCACGGGAATTGTACTTGAACAGTTGACTCATGT

GCTGATGTTTTGCAGAAATTGGTAGAATTTTGAGGCAATTTTCACTTTCATCTTTGCCTT

ATTGTGGTGTTTAGTGGTAATTGGAATTATAGACATGTAAAGCTGATATTTTTGGGTACG

GATTGTAGGATATAACTAATTAAGAAATGGCTTGTAACTTGAGGGTTTCCAAATCATCAT

TAAAATTATAAAAATATATATATGAAAATGCCACGCTCTTTAGAATTTACTATCTATAAC

TTTGTCTCGTAGTATTTGCTAATTCTAATCGAACGTACGAATGAAATGTTTGGCATATCT

TTTTGAACTGATACTTTTGGGAGAATGTTTCAGAAAGTGGTTGTTATCAAATTTTGAAGT

TTTCTTACAATTTTACCTCTTAGCTGTTTTGCATTTGAAACTTCTTATATTGTGAAAAGG

TGAGGAAAATGAAGAGTTAGAGATTTGGCATATCTTTTGATGAGTCCTAGTTGTCAAGAT

CTACAATTTTTAGTTCTACGTAGTTCTAATATTTTAATTCTTTATTTAAGTAAATTGTCT

AAGCCTATCATCTATACTTTTTGAACTGATACTTTTGGGAGAATGTTTCAGAAAGTGGTT

GTTATCAAATTTTGAATTTTTCTTACAATTTTACCTCTTAGCTGTTTTGCATTTGAAACT

TCTTATATTGTGAAAAGGTGAGGAAAATGAAAAGCTAGAGACTTGGTTTCTTGTTGAAGG

CCAATTATAAAGTAACAAGAAGATCATTATATTATATTCCACTATTTATCAGTGACGTGT

AGAATCTTATCAAGGCCTATGTACATAAATAGTTGTTAGCAACATAGTAGGAAGGTTGAC

ACTTTTTCCTTGAATGTGAAAAAACAAGCTAGGATTAGTGTTTATTACTAATTTTTTCTT

GCATTGAACTACAACACATTAGAATTGGAGCTTTTACATGAATAAGTACTATAATCTGAT

AGAAAAATAATAAGTACTATAATTTGATAGTGCTCTTTGTTGGTGTGTAGCATTGTTTCT

ATTACATACAGGGATAAGTTGTGTAATTTTATAATGCTTTGTTGGTGTATCGCATTGCTT

TTATTAAATGCAAGGAAAACACGTGATAATACTTTCACTTGGTTTTACAGTCATTCTTCT

TGATGATCTTATGTCTTGAAATTTAAAATCAAGAACGTAATTATAGAAGAATGAAAAGAA

GTGAAAAAGGTATAACTTGGAGTTGGTATTAGCTTATGAATGGAACACAATTGTATTCAT

GTATTAAATAGTTTGACGATAAAAATTCCATCATCTCTTTTAGAAGTTAAGCAAAGTTTT

CTGTTGTTCGTTGTTTCAGAATTTTTTTTTGTTTGTTGTTTTATTACAGATTGGTGATGG

TTAAACTATTGATCATGAGGCTTATTCTATTGTAAGCTTCATGCTAATTGGTTTCTCTAT

TGCTTGTGCACATTAGAAGTTATTCTGCCTCCGAGTTAACTTCGCTAATATTACATCATA

AATTTTGGTTTTGGCGCTAATAAATTTGGATTGCTGATGGTATTTATATTCCAAATTTCC

AAGTTATGGATTTTAAATCAAAAGAAGATTGATTATGCCATTACATTTATGTAAATTGTT

ATCCTAAATCTAATGGTGGAAGAGTTTAAAGGCTTGAGCAGCTTCTAGGAAGTTGAGCCT

AATGTTTGTTAATACTATATACTACTCTTACTCAAATGTGTTGCAACTTTGTGTATTCTA

ATGGATTTATCTTCTATAATAGTATGAGATAGAATAAAAATTGGTCCATATGTATTTTTC

AGGTCATTTGGGATATACTGTGACTAGATAGCTTCCTGGTTGGGAACATAGCAGATGAAC

ATAACTATATAGGTGGTTTTAGTTCCCGTAGGACTTCTTTTCTGACAGGTTTAGCAATTG

CCTCTTTGTCGAACCAAATTTTGAATGGTCATTGATGCATCAACTCACTATGCTTCTAAT

TCAGCTCAAGCTTTTTGATGGTTTCTGTGAGACAAACAAACTCTGTTGAGCAGGGAGACG

TGTCTGTGAAGACTTCATACAATTTTACAAGTTGGTGGGTTATTTCCCACGATTACACCA

CTTTACTGTTTTAAGAACCAGTTCCTTAAAATGTGTTCCTTTACTAAACTATATGCGGAA

TAAAAACTGTAAATAAATGAACATCAGATATTAGCGTGGAAAAACCCCTTGACTCGGGAT

CAAAAAATCACGACCTACTTCTCAAGTAGGATTTCAACTTCACTATGTAAAAAGACCCAC

TTGGTTACAAGACTCCCTCTTACTAACCTAGGACTAACTCTAATCCTTTAGTGATCTTTC

TCACGATCAGTGAAGTTGTGTCAAGTCCACGACTTGAACCAGAAATTTTACAACTCGATT

ATTGCTTACTACAG

>XLOC_019192 transcript=TCONS_00031485

CTCGAATGGCTTCTCTGGGACTTGGGGCTTTTCCTCTCTTCCATGGGAATTTTCCTTCTC

TCCGTCTCTTTTGAATTTATATCCGTCCTATTCTCCTTTTTGTAAGGAGCTCTTGGCACA

GGCTAGGATGTATGAGTAGGGAGAAGACCATGGAAGATATGCAGCTCTGCTCTCAAATAG

AATTGCAGACTGGAAGATAGATAAGAAAGATGTACAAATTAATTTTGTTGACAGCTCACT

CTTATGGATGTTAACAAACTTCATGAGGAGTGATATTTTCTTCGTGATTAAGTGGTAAAA

GGTAATTATAACTGATTTCTGAAGACGTCATAACTACTCCCTCTGAAAATCCTGTGTTCT

AATTGCTAAATTGATAGTTGAACTATAAACAGATTTCTCTAGACATAAAATCTCAGTATA

GCAGAACCTTGCGCATAAGTTACTTGACAAAGACGAAAAGTACACTTTATCCAAATCACC

GTTATGACTTAAACAACGTTAAAACCACTCTTTTTGAGTTTCAGCATGTTACCTATATAT

GTAGAAGAGTAGAACCTAGTTTCTGCAGTAAATAGTGGTAGGTCTGCTTTGCTTGCAGTG

GTTGTCCTTGGTCTGCACAAAAGTTGCACCCTCAAATGACACTATTTGCTTTAAAAAGAC

AATACAAACTGTGTTTCTCTTCTTAATCCACTTTCCTCCCCTTCCTGGACAAGCAATATT

TGTTTGCTGATATTCACAAAGTTGATTTTGATTCGTGCTCCAGTGTGAATATGTGATTTA

ATTACTTTAATTTTTCCATCTATGGAAGATTTTGGACCACTCCATGAATGTCTTTGTAAC

TGGCATCTACCTTGAAGTTGATGAAGCTAACTTGTATTTGGAGGAACTAACATCAGTGGT

GACTCTTTTTTACTGCGTCTACCAATTTCTCATTTGTTTTAAACATTTTCTAGATTTCTG

AATTCATGTCCATCTCTAGCCTCTGCCTTTTAATAAGAGAACTGTCTCTTACTGTGTGAC

TAGTGTAAAGAAAGTAAGGAACTTATTTTGGGCTTTGTCTGCAAAGAAGAATTTCGCAAG

TGCTTGGAGGCTACTTTGATTTAGGTATAATACTCTTCACATGATGGATTATATGACTCA

TTTCTATTTCAATCTATCTCTATTTATTCCTAAAGACGTTTAGTTGAACTGCACCTATCT

ACATTTAATCCAAAGATACAAATTGTTTCACCATGTAATTAAAATATGCATCTGTACTCG

CATCAAAAGGTCACTATGTTTC

>XLOC_012695 transcript=TCONS_00020780

ATTTCCAACATATCCTATCTTCTCTTGAACTCTACTAGCATAAGGAGACTAGCTCATTAC

GCCAGAGTATCTCTATAAAAGCAGCTCTATACATCTCACATTTTGCAAGGGAGCTGCTTC

TAGAAAATTGGAGTCAGCTAAGGATAACGCCCCGTCCCTGACCAATCCTCCCGCTTATGT

CACTGTGATGAAACCATGAAGCAGTAGCGGCCAATGTGCTCTCCTTTATCCCTTAGAGAA

ATATGTCCATTGATGATGACGATGACGGAAAAAATGTAGAGCTTTAATGCAGACTTTGTA

TTAAAAGAAGGTTGTTGTCTGGCTTTGAACGCTTTCTAGGATACAACGTTGTAAGCTATG

AAGCTAGTGATGTCGCCATCGTCCTTGCTTTGCAGTTCCGACACCAAGACTACTAGGAGC

ATCTCCCTCTTTTGCAGATTTGAAACTTCAGCAATATGTCACAATAGTGCTGATTTGAAG

CATCGCGCCACTGCCACTATGGTCTTCCTTCATTTCTTTTTCTTTGCAGGCTTTAGAAGG

AGCACAAACAGTCTTGATATCAGACTGAGGAGATGGCTTGTACACTTAATTGATGAGGAG

CTATTCGTAGCATGTTGTGCACGATCATGGCAAAGCATTTCTGGCGACTCACATTGTTAT

GCTGATAACACCAAGAAAAATTGAAGACTCATGCCTTCGCCATTGACATTCATCATGCCG

ACTTGAAACTTCTTTCCACCATCTTGAGTTGAAGCCCAAAGCAATGTCCACCATGGAGTT

GGAGCATCGTGTCATCAACACTATGGGCTCCTTTTTTTTCTTTTTCTTTTTTGCAAGTAC

AACAGATTCCCTCATCCTTTCGACTAGGCGAGATGGACACTTGGAGTAGCCTCTTTTCAT

CCTTGGCAAACAATAGTATCTAATCATCCTTTTAATATAGCGAGGACACTTGGTAGTACC

CCTTTATGCACCTGGTGGAGAGGTTTGCAGCTCTGCAACCATGGAGACTAACTAGTGTCC

CATTTTCGTGAAGCTTTACAATTTTGGAGAGACTTGAGCTGGCGTGGCGTTCTTGCTGTT

TAGTGGCTTTGGTGAGAACCCACTAAAAGGCATCCTCCTCCTACACCTTAACGAAGAATA

CATGGTGGTTTCCTCTGTCCGAGCTTTGTTCAAGCAGCTAAGCAAAGAAGTACATTATTC

TTTCCGGCTTGGTGAGGAATTCTTGGAGTAACTCTTTTCCATCCTTGGCGAGGCTAGTGC

ATCTGATCTCCTACGGCCTGGCAGAGAATCGTGTTTCATCCTTCAACATTTGGTGGAAAG

GAGCGTGGTTACTAAACCTTCAAACTTTGGTCATAGAACAACCTGATCTTGAAACTTGAC

AGAGAGGCTCATGGTGAATTGGATTCTTCAAGCTTCGTTGAATTCTCACCTTGCATACGC

AACTCACAGATTCAGCTTGGTGCATTCTTCTGGATCAACGGGAATTAATTTTTGAATTTG

AGGAAACGGTCGGCCACACTGACTTGACGGCTTGGAATGATCTTGCCAAAAAATCATATT

TCGCTCTAGGAGTCTCCGAATCACATGTCCTTTTGTATGTAGAGGACTACATGAACTATA

ACATATCAGTAAATCAGAAATTGATTCCAACCCAATTGGTACAGTTTATTATTTGAAGCC

AACCAAGATGTATATCAAGCTGATTTCATAGATTTGCGCAAGCTTACGGAAAAACTCATA

TCTCAAGATACAAGTGTCCAAATCAAAATCCACTTGATTCTGTAGAGAGGAGACTTCAAG

GGCTACGACTTTCAAAGAAATCAGTGTAACAAAAATTCTGGATTAGTGGGAATGAACTTT

TTTTGAAACTGGCGAAGCTGTTTGCCGTGCAAACTTGACAACTCCAAACGATCTTTCCAA

AAAACTTTATATATCAAGATAGGAACGCCCAAATGACTAGCCGTTTAATCAACTGGAAAC

TAGACTCTGAGTACTACATCCATGCTTAATATCGAAGTCTAGTTCATCCTGAGTTGACAC

AGAAAAATCTTGGAATTTAGCGCTGTGATTTGGACAATTTGACTTTGGGCAGCTATTTGA

TGAGCTCTTCTTTCCTTTTGTTTATATTTTATTATGTAGGCTTGTAATGGGAGAATTAAT

GGTCGCAATGTTAGACCAAGATAGTGCATGAGACGTCGCACATATATTTTGCTGTTTTGG

GCAACAAGCAAAATTTCCACATAAAAGAAGAGCCTTAAGTATTCACCATTGTCGATTGTT

GAAGAATAACTTAGAGCATGCCATCCTTCAAGCTTCAGCATGGAACGTCTGCTTGGAACA

TAGCAAATGAGTTCATGGAGCATCACCTTCTTGTAGCTTTTATGTGTAGCAACCTCTTTC

GAAACTTAGTGAAGAAGTGTGTGGGGTGTCTCAACCTTCAACTTTTTTGGAAGAACACAT

AGAGTGTCAACTCCTTTTGGCCGCGATGGATGACTTGAGTGAATATATGATACCCCCCAT

TGAAGAAAATATATATTTGATCCATCTTCAGCACAAGTTGTGAATACTTGAAGGAGGCTT

CAAGACAAGTCCAACAACAACCTTGGCATCAAACTATTTTCAGGACTTAGGCTCTTTGTA

TAGGATGATATCTTGTTGCTTATTTCTTTCCTTTGAATCACTTTGTATCAAATTTCTTCA

ATATTGAAGCAAGAAACATCTTTTCATTTTGATGCAAATTTTCTTCTTTTTTATTTTTGA

CTTGCATTCATCTGCAATGAAAGAATCAATATAGAACCGCC

>XLOC_013026 transcript=TCONS_00021332

ATAGGCATGAATGAATTGATGTGCAATATTCAATAGGGTTTTTTACACATATATACAAAT

ATACACTCCTAATTACACAAAAATAGCAATATTTTTTAAATTACAACATATGACAATATT

TTTACTAACACATTTTACAACTATCAAAGACAGAGAAACAATCACAGAAATTATAGTCAA

ATCCCTCTACCCTGCATCTTCACCCTTCTTTCTCCGCCCCCTTCCACCACCATATCAGCT

GCCACCGTATCAGCCGCTGCCCTCCGCTAGTGAAATTATCGAGAAGATGAAGAAGAATCA

TACTCAATTAACACATATTTCAGAAACAGTTGGAGACAGTTGAAACGGTGCAGAAACAGT

TAGATACGGTGCTGCAGAAACAGTTGCAACGGTGCTGAAACAGTTAGATACGGTGCTGCA

GAAACAGTTGCAACGGTGCTGAAACAGTTAGATACGGTGCTGAAACAGTTAGATACGGTG

CTGCAGAAACAGTTGAAACGGTGCAGCAAAAACAGTTAGAAACGGTGCTGCAGAAACAGT

TAGATACAGTGCTGCAGAAACAATTGAAACGGTGCTGAAACAGTTAGATACGGTGCTGCA

GAAACAGTTGAAACGGTGCTGCAGAAACAGTTAGATACGTGCTGCAGAAACAGTTAGATA

CGGTGCTGCAGAAACAGTTGAAACGGTGCTGAAACAGTTAGATACGGTGCTGTAGAAACA

GTTGAAACGGTGCAGAAACAGTTGAGATACGGTGAGATACAACGAGATACATCAAGGATG

GGATGAGGAAAAGTGAAATTAGAGGAAATAATAGGAATAAAAGTGAAATTGGGGGAAATA

GTGGGAGGAAAATTGAAATAGGAAGAAATGGGAGGAGCAAAAGTGATAGTGGCGGAAATA

GGAGGAAGAATGTAAGGATTTGGTATGTTATGGGAAA

>XLOC_012321 transcript=TCONS_00020165

CGATACACTACAGTACTACACTATATATACATATATTGCACTTGCCCACATGGCTACATC

TGGTCTATCCATCTCCTTAATCCTACGTTCCACATTATTTCCTTCCTTTTAACGATACAC

CAAACCACCCATAACAAAAAACCTAATTTTATTTCCCCTCTTTCCTTCCATTCGTATCAG

CCGCCCTACTCACTCACACACTCTCCCCTTTGCTCAAATACGGTCAGATACACCACCCCT

CCGCTAGTTTCACCGGCAACACCCTCCTCCTTTCCCTCTCTCTTTTATCCTCTTCGCACC

AGTAGTGGAAAAGCAATCAATCCCTCAATTTTTACCCGAACCCGAAAAAGAAATAGGGGA

TTCAAGCACGAAACTTCTTAAATCGAGATGAATTCAAAAAATACTTTGCAAAACGGATGA

TTCGAGCACCAATCTTTGAAATCAAGCAAAACGAGTACAATCCCGTCAGTTTCTTTTGGC

CCGACCCAATTCGATACGGGAGATTAGTGTTCATGTACAAAGTGAACAAAAGCACGAAAT

TAACCCCACTTTGACAGAAAAGAAAGTGGATCCTTCGAAGTCTGGAAATAAGTATTCAAG

AATGAACTAGGCAGTTCATTCACATGCATACAAGGCACCTTTCTTTCTTCTTTTATCTTC

CCTCTTAGACCCTTCACAAGATGTATTCGTTAGGGCTCCTGCATGTACTTATTATGTAGA

CGAAATATGATGAAAAGAGGCAAATAACTTGCCTTGAAGCTAGGGGTGGCAAT

>XLOC_001927 transcript=TCONS_00003079

AATTATTCTAACACTCCGGACTTGTTATCTTGTTGTGATACTGTCATGTCATAATTTTTT

ATATTTGGTGTCCTGTCCTCATGAGTGTTCCATTTTGGATTAATAGACCAAACAGGAAAT

TTTAGAAGAGTTGTATTGGTACTAATTTTTTTTATTCTGAGGTACAATGTGTTCATTTTT

AATGGCACTCATTAGCTAATACGTCATATTATTGAAAATGTCTTTCTGTGCAGTAAATTT

AAGGGCTGCTTCATTTGATAAGTTGTTACCAAAGATAGATGTAGTGTAGATGATCACTTG

GAGATTATAGATGATCAAATCTTTGCTGCTAAGGCACGAGTAACTAACCATGGATATGTA

GCCAGCAGAAATCAACAGAGGAGCTTTTTATTCTTTAGCTTAAGGAGCTCAGTTTTTGAC

ATTCTCATGGCTGTAAACATATAGTATGTTCTTGAGGATGGAGATGGAGTCTCTGAGGTG

CTTTATACCTTAGACGACTCACATATGGAATGTGATGATTCTGATAAGGGGTGCTTTCCT

GGAAGTGAAGCAAACTGAAAGACAAATAAATGGAGAGCACTAAATGCATTGATATGTTTG

TTTGCTCTGTAAGCAAGAACTGCAAGTCTTAGCAAGATATGCCAGAAATACATTCACCGA

AAATGTATGTGACAGCTAAGAATTTTCCATTTTTGCCTATTATCGTAGAAAGGTAATTAG

TTTCAGTGCTCTAATTTAGTAGGATGCTCTCTTGCTTCCTTGACACCTT

>XLOC_023596 transcript=TCONS_00038776

TGTCAACCACTTTGCCGCCTTATACGGTTACAACTCATCTAAGCGTCAAACATCTTATCT

CAAATTAATCTCAACCGTCCAATCCTTCCAAATCCTCAATAAATTACCACCTTTTATTTC

ATTCATGTACATGCACACATACCTCCTCTATTTTCTTTAGATCTATTCTATGTAATTGAT

CATCTTCATTTTTCATATTCATATTTATGTAGATCGGTCTTCGATCTACATTTTGAATAT

GAATTTGTTATATATCAGCTTTGGCCGGTTAATTGACCGGCCGAATGCTGTAATAATTCG

ATCTCCGTCAATTCTGACCGTTGATTGACGGAGATCCGGTTTGATCTAATGCTTTGAGCT

GGATGTGCATCAGATCTGGGTATCGTGGAGGTGACACGTGGATTGGCGTCAGCTAAATGA

ACATTTATTGTTCTCAAAAGAGAGCTGACGAGTCTGGTGTCTGATCCTGATCCGTGTGAA

TCAGACGGTTTGGATTGGTGAGCTTTGGATGATGGAACGATTTGGCGAGCTTGTTGGTTA

TATATATCTGAGGTTATAGTAGTTTATTTGATGTTTATAATTATTTGAATGGATCATTTG

CATTAGTTTTTATTTATGCATGCATATGTACGAGAGAATATGAAAAGTAGTGTGTTATGG

AGAGAGAGAGAATGAAAAGTAGTGTGTTATGGAGAGAGAGAGAGGAGATGGAAAAAAGTG

AGATTTATTGTTATGTTACCAGGCCTTTGCCATGTTGGGTGCGCTCGCATGGCAGGTCAG

AAATACCTAATAAAAGCATTTTTTTTGGTGTCTTGAGGGGAGATGGCACGGGGATTTGCC

CGGCTCTCCTCTCGCGCGTGTCTCGTGTCCCTTTTTATTTATTTCTGCTTGTCTTGCCTA

CTCCTACGCCCTACAATTTTGCATCGATCGGGTGGTTACTCGTCCTTGAATGAGTCTTTT

GATCTCGTATTTTGAGAGGGGAGAGACCTATTCAAGGATGAGTAACTCATGTTGATTGCG

ATGGACAAGCAATTGATTCTACTGCAGCGCTCCTCTTGTTTAAAGGTACAATCACCAGTT

CTCTTGCTAAAATTACATCAAATATTGTTCTTCACATTGTCAATATCATAATTAGCTAGC

TAGCTCGGCTTCATTTCACATTAATTTGTTATTATCAATAATTTGCTAAGCCGCACTTAG

GTTGATTGTTATGTTCTAGTAAGGTTGATAACTAATTGGTTGCCTATATATATATACACA

CTACAACACACTGATTGTGCATTGCCCTTCTAATTTTTTGAAAATCTGTTTTCAAAAAAG

AAAATGTTTTACAGATACTTTGGTTAGCCTATTTAGATCTATAAAACCCTGTAACATACT

GTGTTTCTGGGTAGATTGTTGTGTTGGTAGAACTGGTAATTAAACCAGCTGACGTTTGAA

GAAGTGTGCTTATTCTTATAAAACTAGTAATGGCATATTTCAATAAATAAAAAATAGCAT

TGGAGCCAATCACTGCCAATTACTTTGACTCTTCGTGATCAGTATGAAATTATGATTCAA

CTTATATATAATTATCACATTGCTGATAATAATTACATGCTGCTAACAGACTATCATCCT

ATTCCATTTTATTAACTCAAGAGGGGAAAAATATTGACTTATCCAAACAAGAGTTGGTGA

ACGAGAATGCGTTAATGTGTTAATCCTTCATTACAAAGCTTTCATTACGAGTATTCATTT

TGCATTTGTGAATCAATAGTTTGTGAAGTTCTAATTTTATTCTAAAGTACTCGGTGATTT

TTGATTATCCACCCGTGGAGTTTGCTCACTAGTTGTGATTCTTGGTAAAATCATTGCGGG

GTGCTATTTGTGTTCCCCATGTAATTGCTTTGTTGTAATCGTGCTATTTCAATGGAGATG

GAGAAGTACTTGGGACGATGCTTTTTGTAGAAAGGTTTCAAACTTTGTCCTTCGACAGTT

AAAGCTTATTTCAAGGTCAAGTATGAAGTTTTACAAGTGATGATGATTGAGTGCCTCTCA

ACAAGTATGAATAATAGAATGTGCGGAGTCCCAGCTAATGTTCTAGGCCATGTTGATGCA

TGTTGCTGCCTTGATGTACTGACTGATCCTGCATATGTGCTGAATGTATGCTAAGTTATA

TGAAGAGATGCCTGAGGATTGCCGCTGCTGATGTGTTGCCGATGCTGCTGCGTTTTGATT

TTGCTGGTATTGTACTGCTTCTTTGAAGAGAGGATGCTCATTACACGACCGTAATATGAG

GTTTTGGGGAGGATCAGCTCTTTGTGCTGTTATTGTTCTGATTTGAAGCTCTAGCTTATA

TTTTGATTTATGGTGATTTCGGGATCCATGAGGGTTCAACACCAGTTGGTTCTTGTTAGT

GCTGCACCATGACCAAGCTGATCATCCGAAGAGCTTTCAGTTGCGTATTACTGTGTTAGC

TATTCCTATGTTGGAACACAAGTCATATCATATTGTGAAGTTTCTATTTTATGTTAACAG

AAGTCTGTTGTTACTGAGATGTCTCTGTGGAACTGCCAGTGTGGTAATTTTGGAAGTATT

TGAGGTATTTACCTGGGGTCTGTGTCTGTCTCATTTTATTTCTAATACCTTGAGGTAATT

CAACTGTACACTGGTTCATTACAACTCTTACACGCTCTTGTACTTGAAGGCTTTTTCTCT

GGAATTTCTTGTGCTTTATTCTTAGCTGTCTTTTAATCTTTGTTGCTCTGAATAGATGAT

ACTCAGGTTCTCTGTGGTTGCAGGGGTAACTGTAGAACCTTCATGTTCTAATGTGTTCTT

TTTTTAGCTTTCAGCTATTTCTCTGTTAAAGGTGGACATCTAAGCAGGCTTTCTTCAAGG

TTCCAAACCACTCGACTGTATTGTGTTTGTTGGTAGGTTGTTTTGGACTTGTGGTAATCT

CTTCCAAGTAGTCATTTTTGTTAATTATAGCACCTTTGTTATTGCTGGAGGTTACTAGTG

GGTCTGGCACAGGTTTTCCGGTCATGTGTATATGTATACATTACAGTGTGTATATGTATA

CACTACAGGGTATCAGACACCATGTAAAGTGACCATTATTATGGTGTAATGCCCTTGAAA

TCTTTCTAGTTATTGTGGCACAGATTCAAACTTACTGGTGGATTGCTGGTGATAGTCTTT

GGGATGCTTTGATATCGTTTTGATGAGGATTATAGAGTGAAAATAGCACCAAATACACAT

CACAGATCCTTTATACTTGTGGTCTCCGTATAATCAGTTCATTCAAGTTCCATTATTAAA

GGTTGATATTATTTTTGGTGGTTTTTGTATATCCAGTCTGATATGTTTCAGTTCACTCTT

AAATGGTTTAAGAAGTTAAATGTTTTTCGTTGATATGTAACATGAGTTAAAAAATCATTT

AACAGCATGTACTTTAATGATGTTCTGGAATTAACCTCTATTTTTCCACCCAAAGTCGCT

GTATAACTTGAGCTTATTGGGTAAATTGTTAAAGATATAAGATGTTTCTTTTCAATTGAT

TTTTAGTATCAAATTAATTGCCAAATGTTGCGGTTAATTTGTTGAAAGAATCGATTATTG

ATGGATCCAGGAGCTCGGTTTTGGGGGAGCGCTGGAGTGCCTGTGCACTCGAGAGGTCGG

TGGTTCGAATCCACCTGGATTCCTAATATACTGTGTTGACAAAATATGAAATGAATTGTT

AAGCTGTGTATCATCAATGTTAAAAGTGACATTTTCTAATGATGGTTCGTCTGCTGAGTA

GTATATTATTTTTTTGTTCTGTGAAAGTGAAAAAATATTTGAGAAAAACAGAAGTATAAG

CAGGTGGCGGTGTTGGCGGTGTTAAATTCTATTTATTTTTCCCGTTTAATGCCGCTAGTA

GCATCACTGACATTTAATTCTTGTCTTTTCAAGGGGCTGTTAGGTTGGCGTATCATTGAC

GTAACAAGCTAGTATTACTCAAACAGTTGAACAGAGTATATCGGGTGATA

>XLOC_037639 transcript=TCONS_00061517

TCGGATCCACCTTCTTCAACTTCTCAATCTCTCTCTCCATAACTAGAATAACGAGGCATC

TGAGAGATCCAAAGCAATAATGGGGTTCTTGGTGACAACTCTGATCTTTGTTGTAGTTGG

TGTTATTGCATCTCTGTGTGCCAGAATCTGCTGCAATCGAGGCCCTTCTACTAATCTGTT

ACACCTGACATTGATTATCACGGCGACTGTCTGCTGTTGGATGATGTAAGTTTGATATAT

TGTTCAAATTTCCGTTTAAGCTGCAAAACATGATAAAAAGTTGAGAAAGTATTATTCAAT

GAAATTTTTAAACTTGAAGATGTCTGGATGCCTTTAAGTAGATTATGATATTTTTGCATT

TTGTCCTTTTAAAACTCTG

>XLOC_020583 transcript=TCONS_00033792

GACTCTTTTACACAGATAAATCCAACAGTATTGTATTTTGAGACATGAGTAAATTATAAA

TTGATCTTGCATTGGAATCTCACAGGCAGAAAGATCTGGCACTACTTTGAAGATATGAGC

ACTTAAAGATCAACAAGGACCGTTCTCTAACCTTCTTCATCTTTCTCTTTTCTGCTTTAT

CCCGGAATTTCTCTATTCCTTTTTCCTCTGTTAAGAACTTAAGGTCGACTTTTTGGTTTG

GCTTGGTTTCGGTCGGTTCAGGTTGTAACTGCTTCTGGTACTTGAGAGAGTTGTTCAAAT

TGGATGCTGCAGAGTACTGATCCTGGAAAAATTGGCAGCATCTTCTGTCATTCTCGTGAT

GATAAATTTGTGATCAAGACTTTAAAGAAGCCTGCACTCAAAGTTTGGACAGTAGAAAAG

TTTACCTACTAAACTATTCCAGCTCGCTTTTGCTCGCTTACATGTTCATAATGCCACTAA

GTTCATACTGAACCCAATTCAGCTAGAAGTACATCTGCCGTTGTATTTTGGGATTAGAGA

TGGAACCATTTATGAGGAGTGAAGTGATGTTTTAGCTTAAATCTAGTAATGCTCCCTCCC

TTTCCTATCCTAATCTCCTCTTTTAATATCCATTGTAGGTTATGTTCTAGCCTTATAAGC

GTAGAGCAAGAACTTTATGTAGAACTTATGTTTTTTTTTTCCTTTGATAGAGTCAACTTT

GATCTCACTCCTCTAATGTTGTATGAAGGATTCATTCTGGAGTTGG

>XLOC_012549 transcript=TCONS_00020534

TCGAGTTAAGCTTGGCACAAACTTTACATGCCTCTTGGAAAGAAGCTTTGGCTTCAGATT

CTCTCTCCTTCGATTACAGAGAGAATCCTGGCTTGGGGACCTTTGGTGAGGCTATTGAAT

TGTTCCGGACTTGAACCTTGAGGGCAAGGTTCTAACCACTATAGAAGACTCATTTGGGAA

GTTGTCAAATTATATCTGGGACCTTGGTTAAATGGAGCATGCGCATAGTGTGTGACAGGA

TTCCTGATGTAACGGCGCAAAGAAAAGCAATTTGTCAAGGTGCATAGCATATGGGAGAAT

GCTTTTTAATGAGCAAGCAAATTTCCCATTACTGGATGCAAGGAAAATCAAAAACTGCTG

TCAGAGGAAACAATCCTTGATTGCGCGACAGCGTATGGAAACTCTTGGTAGGGATCATAA

TACTCCTCTAGTAGGCTTGTCCGGCTCGAATCTGGTTAGTTGGCACGGTGAGTTTCTGAT

ACTGGATGACTAAAGAAAAGAATGGAAGCTACTTTTCCCAAAATCATATGTGGCAATGAA

CTGTACCAATTCTTCAGATGTACCTTTTTCTCCCTTGTAGGTTGGTAGTTGGCTTGTAGG

TTTTCTCATCTGATCCTAAATCATTCAACAACCTGAGTGTTGTAGCTCTCTTGTTCTTGG

CTGCTAGACGAGGAGCTGGGTAGAGAGAGAATAGATCCTCCCCCTAGGATGGTAGCTTCT

ATTCCTTGATTGCCCCGCAAGATTCATTTTGAAGAGAAAAAGATACAGAATGAAGGTAAA

AGGTGCCTTTTTGTCCTCTAAACATGTCAGTTTGAAGAATAAATAAAGATATGAAAATGT

GTGAGAACTAAATTCCCATTTTTCCTTCGTATATCGGTTTCATATATGATTTTCTACACA

TGAAATAGATCGAAGGACAAGTGGATTAATTCGCTATAATCTTATTTAATAAACATACAT

GCAGCAACAAAATAAACACAAAGGCCTAGTCACTAAAAGAAAAGGAAATAGAGAAAATAA

TAGAGGAAAAAGACACTTTTGTTTTTCATTTTTTGTTGGGATATTCACAGATGTGGATTC

AAGATTTTTGAAATTCTACATAGCCCATTGTTTTCTAGGTTCGACATCTATTATTTGTAT

TTTTTAGTCAATTTTTTTAACACAGTTTTGAGTTAAAACTATTGGATTCGGAGAACTCAT

AACTTGTACACTACATTCATCCTTGGATATATATATATATGGGGAGCTTTGGAGTAATTG

GTAAAGTTGTCTTCGTGTGATGTATAGGTCATAAAGTTCAAGCCTTGAAAACAGTCACTT

ATGCTTGCATAAGGTTAGACTGCGTACATCACACCCTTTGGGTGGAGCCCTTCCTCGAAC

TCTGTGAACGCGGAATGCTTTGTACACCAAACTGCCCTTTATTACGTGTTTCTTTGGCTG

AGGTTTGGCTAGCGGTATCTTTCTACGATTTGAAGTAGGTACAATTCACTTACTAGTACA

GAACAATTTATAAGAACTTTATATAAATATCATATTTTGAAAATTTTCAAATATGTATGT

ATGATTCCGCA

>XLOC_023528 transcript=TCONS_00038665

CACCACACTTTCGGGAATAACTGAAGAGAGGTCTCATATGAAGATGAGAGCATTGTTTAT

TAGCCAAGGATGACTTGCCTACTCCATTTGACAGTTCCGAAAAAATAAAAAAATAACAAA

CTAAAGAAGGTTACAATAATGATCCTTTTCATTTGGTTGCAGGCGAGTTATCCTGGCTAT

ATTGACAAGCTCTTCTCTTTCATCAGTTCATTCCAAGAAGTAGTCCTTTCCGCTTCGTTG

CCTTTCTCAACTCAACTCTCGCGGCACTGGCCGACGTGGTCTCCAAGAATCTTCTTCCGG

CCTCAGAGGTGGTCCGTCAAGCCAAGGAACTCAAAACCACAACACAAGCACCGTATATAA

CTGATTGTCTAACTAAAGAGGTTAGGAAAGTTGCGTCGGATCAACATCGGAATTACAATA

AACTACAAAAGCAACTGGAAAAGGGCATGGTGTACATCATTAAACTTTTGAGTAGTCGGC

GTCGGCCCTTGCCGCCTTCCTTAACAAGCCCTCGAGCCTCCTTTGCGCCGCCTTCTTCAA

CAATGTAAAGATTCCTAAGATTAGCCCCTGTTCTTACATGTTTAGACATCGTTAAAAAAA

GCTTATGGCTATCACCGAATCTTACTAGTAGCAGAACTTGGGTATTTTCAAGTGTTTCAT

GCTGTTTGACTTCTGGCGATCGGTAGCAGTTTCCGCTGCTCGCCTTCTAGCGAGCTTTTC

CTGACAGGCGCTAAAAATATAATAGTTCTCTTATGGGGTAAACTGTGATAGTGGTGCCAA

ATATGTTCATTCCGCGCAAAATTTAAAGTGATTTGGTCTTGGCCCTTTCCAAACACTAGA

GCCAAACCAAAGTGATGGCCAATAATCCATCTTACATTGATTGAAATAGTCAAATAGCTT

TTTTTGAGGTCACTATT

>XLOC_004570 transcript=TCONS_00007427

GTGAATGATAGGGGCAATTGGCAATGTTTGATTCTGTACGCCCTTGCCTTCCTTGGACCT

GGCTCCATCTTCCATCGACTCAATGTCAAGATGGGCTTACTTGGACAACTATCAAGGCAT

ATTTAGGCGGCATATCTGCTTTAACTTTGTCGGACATGGAAATACTTGTTTCTATTGGAC

ATCTTCATCATTGGGTATGGAGTAAGTGCTGGAATATAGAATGTTAAAGCTACACTAGTC

ATACCTAGTGTTGAACTTCATGTGGTATCAAGTTTTTTCTGCTTCGGCGCGACTTCAGAT

TCTGGTCACTGGTCAATGGTATCGAGGCCCCTGTGAATACGCCAAGGTGTCTAAATAATT

GCTACAACTTGAAAAGTTTTTGGAAAAACTGGCATGCTTCTCTTAACAAATGGCTAGTTA

GATATCTGTACATTCCTCGTGGAGGCACTCAGACAAAACTGCTAAATGTGGGTTATCTTC

ACATTTGTTGCTGTATGGCATGATCTGGAGTGGTACCGTTGCTACATTCATCAACTTTCC

TTTGTGTTATGGTACTAGGCTTAGAGTTCATCGCAACTTGCTTGTAATTGGCTTCAGGAA

GCTTCTTTTTGGGCATGGTTGACATGCATCTTCTTTGTACCAGAAATGATAGTAAAATCA

GCTGCAAAAGCCCTCAAGGGTGGAGAGCTCTTCTGGCAAGTTCCTTTACCGTGAACTTAG

TGCTGTTGCTGGGGCTATCACCATCACTTGTCTCATGGTGGCCAATCTTGCTGGCTTTGT

CATTGGACCATCCAGGATCAATTGGCTTCTTTCTAAATTTCTCCAGAAAGAAGGACTCCC

GACACTTTGTGGCATGTGTATTACATTTTACGTTGGCACAAAGCTGATGTTCCATGTATC

TGATGCAAAGAAAGGAGTCTTCGCTAGATGATACAAAGGTTCTTTCCTGTCCCACTGAAC

AGAGACAATATAATGATGCAAATACCATTAAGATCGCCAGGCTTCTCGTGAGGAAGCTTT

CAATAAATAATGGTATCAGGAGAGGAATTGTATGGTATGTTTCAGGAAATTATTCTGGTC

TTCGGACAGTCAGATGACCTTTTGGCTAAATATTTCAATATCCATCTATTGTATTGGTCA

ACATGAGCTCGCCAGGTTGCAGTTTTTTGGGACCACTCAGGTCTTACTGGTGCATGAATG

AGGCTAAGTGGGGCACAGAAATCTGACCACAAAATTGGAGTTTGCTGTAGGACTACATTG

GGTGAACGTGTGCGCGGTCGTCGTCCAACTGAATTTCTGTGATATATTTGACAACTGGGG

CCAAATGGTAAATGACAGAAAATATCAAGTCGTTTCTTTATAGGCATTTGTATTGTGGTT

CTTGGCTTCATATCAAATGGGCCATCCCTCTCAAGTCTTAGGGGTATCCGATAGTTGCTT

CTATTCATTTCAAGCTACAATGGCATTGTCTTTCACTTAAATTTTGTGTAATCATGAAGT

CCAGACCATGCTGTAAGGCAGAAATTTTCAGCAAATGTATGTAAATTTGTAGGGCCTGTA

ATTAAATTGGATACTCATTCCATTCTGTGGTTAAAAGCAGGGCAAGCTTGAAAAAGGAAA

TATTTACTACACTACGTGAGGAGTATCGCTACCAGGCTGGTCTATATACTACAGAAGGCA

AATGGCCCTTTCATAACATGCAATAGATTTGAGCAAAGTGGTGAAGTGATATGAATATTT

CTCTCTTTTAGTTCTCAAAAGTAAGTTGGGATGCTTTCCTTTCTATCCAAGCATATGCTT

TGGTGGAATCCATGTTGCAGATTCCATGCCTTCACTTCATACGAAGTACCCTACATTTTG

GACAGATGTTTGCATCCCAATTTGTGTATAGACAGAGTCTACAGCAAGATAGAACAAACA

GAACAACAGAAACATAAGTCTAAGTGAACATTTGCCTGCATCTCTTGGGGACATAAGTCT

TTTTACTTATTCACTTTCTATTGCCAAAAATGTACTGAGCTATAAGATTTGCAGTTAATT

TTTAGTTTTAATGTTTTTAGCCTTTCCTATTTTTCCTGGCAGATATTAGGTTCCTAGTAT

TTGACAAGCTTACCTAATTATTGTCACATCAACGTGATAAGGATTTCCTATATTGACAGG

CATGATATTCTGGAG

>XLOC_012014 transcript=TCONS_00019702

TGGAGCAAGGCACGGCAGCGTGCTGTAATTTGCAATCCCAGTACAAGAGAAAAAGTATTT

GTTCCTTCTCCAGGAGATGGACAACGTGGAGGATGGGAGAGGCATATCATAACTGGCTTT

CCAATTGTTTGTAAGGAGATGGAGGGTCATACAGTTCATTTTTGGTCCTGGACGTCGCAC

AATAAGGAGATTACATTCGTTGCTAATGAGGATTGCGATAAATATGTTTGTTATGATGTC

ACGAGAAAAAGTTGGAGAAAATATGAAATCCAGAGATTTTGTGAAGAGCATGCGTTCGGA

GGCATTTACTGTGGCATAGAAAGCCTTTTCCCATTGAAAAGAATTTGTTGTAAAGCTCAT

CAACAACAACACCTTCAGGACGAAGACTACAATCACGAGGGCGAGGGATTTTGTGACGTG

CATACGTTTGGAGGCATTTACAGTTGCGCAGAAAGCCTTTTTCCATTGAAAAATTTTTGT

AGAGCTCAGCAACAACAAAACCTTCAGGACGAGGACGAGGACGAGGACGAGGACGAGGAC

GAGGACGAGGACGAGGACGAGGACGATGACGATGACGATGACGAGGACGTGGACGAGGAG

AAGAGAAGGAAGAGGAGGAGGAGGAGGAGTTGTTCTTACTTTACTTTCTTTTAACTTTGA

ACATGTTATGTTACTGTTGAACTTTTAGTTAGTTGATACTTCTTTGAGATTTTGAGCCTT

TATAATTAGTTGGA

>XLOC_012553 transcript=TCONS_00020551

TCTTTCTCTCTCTCTCTCTCTTTCAAGTCTCAATTATATTATTTTCTCAAATCTGTTATA

TATTTTACACAATAATAAAGTCGTAGCGAAAATAATAATAAGTTCAATTTCAAGGCCAAA

TTGGAGATATACTCAAGCATTTGTGACATTTTGGGAGACTACAAGTGGTGGTGTCAATTT

TAACCTCAGTTCATAACTACTAGGGAACAATTTTCATTCTACCCCAAAGTAAAGTAAAGG

AAGTGGACAAGAAATGCAAGGATTTTTTGTGGGGGAGCACATAAGAGAGGAAAAAAGTAC

CACTAGTTGCTTGGGAGCAGGTATGTGTACCCAAAAGATATGGGGGCCTAAACATTAAGA

GCTGCAAATTCTAGAATATAGCATCAGTAGGAAAACTGATCTGGCAAGTTTCAAAGAAAA

AGGATGTGCTTTGGGTGAAGTGGGTACATGGATTATACATGAAGAGAACCACTAATTTCT

GGGAACACAACCCACCAGCAGACAGCAGCTGGTACTGGAGAAAGCTAAACAAGGTAAAGG

AAAGTATGGCTGGGTGGTTCACATAGAGGGTATGCAATATCACAAGAAATGGGGTGTATA

GAGTCACTGAAGGCTACAACAAGCTCATAGGTGAGAGACAAGAATTGAAGACCTATGACC

TCATGTGGACATCAGTGTCTATGCCTATGATCAGGTTCATCACTTGGCTTGCAGAACGAG

GACATAATGCAGAGAATGAACATTCACTGTCTAGATGGCACCTGTTGCTTGTGCAATGAT

AGAGTGCAAAAAACACACCAGCATTATTCAAGGATTGTACTTGGTTTAAAAGAATCAGGA

ATGCAGTTCAAAGCTGACTGAAGCAGAATACCAGAAATTATGACATTGCAGACACATTAC

TATGGATTAAAGAAATAAGATGGAAGAGATTTAGAAAGGAGATAGTAGCAGCAGGACTGG

GTGCAGGTATATATTACACATGGCAAGCAAGGAACTGGAAAATATATAGAGAAGGAAATG

TACCACAAGACTTCATCATCATGCAGATAAAGGAACGCATAAAGGAGAGACTGGAGATGC

TGCAATGTTACAGGAGAACAAGAAGCTGCAGTAACTATATTAGGCTAATAAGTAGTTAAG

AATCTGTAAACAAATAGGATGATGCAATGTAGGTAGAAAGTAGTATAGGTTATCAACTTT

CATTGTAAGATTTCATTTTTATTATTATCAGCATATACTCGGATGTACTGTAGTTGGTTG

GAGGCCTTTAATGACAGGAGTCATTGTTGCTCAAGAGGACTCTTTAGGTGTA

>XLOC_018935 transcript=TCONS_00031053

CGCACTTTGCTTTCCTTGAAAAGCAAAGTGATGAAGAAGTCAAGAGTTTCGTCTGGAGAG

ATCATCGGCAGCAATGAAAGTATGTTAAGCTTAATTTTTCTCCGTTTACCTGTCAAATCC

CTCATAAGATTCAAAGCTGTATGCAAACAATGGCTTTCTCTTGTTTCCGACCCCTATTTT

GCTAATAGTCATACCATAACTAGTGTCGAAAATAATCACAGTTTCCTTACCCCTTCTGGC

TTATTCATCTTTAATTCATTTGATAATCACATTAGTTCTGTTTCTTTGTCCAAAACAAGA

AAAAGAATAAAACCTTCTTTGCCTTCTCTTTCATTTCTTGGTCACAACTTTAAGATTTTA

CAGTCATGTAATGGCCTTTTACTCGGCCGTTTAAGCTGTGGAACCACTTGTGTTTGCAAT

CCCACAACTCGGAAATTCAAGATTCTTCCATCTGCTACGGTGGATGGCGAATTCTCATGG

ATTAGTCCATTCACTATAGCCTTTGATCCATCTCGATCGCCTAATTACAGAGTCGTGTGT

TTCAAGAGGTTCAAGAAAGAGTTACCTGAATCAACTGGAAGTTACTTTTTTCAGATATTT

ATGTATTTATCTGAGACGGGTTTTTGGAAAGCGAACACTTGTTTTGAAGTGAACTTGTGC

TCGGACATGGCTGCGGGGATCTTCTCAAATGGTTCCATTCATTGGCAATGCCATGAACAG

TACATTCGTTTCGATATGAATGAGGAGAAAGTGAGACTAATGACATTGCCATTTGTATAC

TGGAATCATCGACTTTCAAGTCCAATTGGTTACCTTGGGCAATGGTGCGACAATGTCTAT

TCTTGTATAAAAGATGACTGGACTTGGAATTCGCATGAGCTAGATCAAGGTACTTTGGAG

TGGACTAAGAAAAACTATGACATTGCTGCCTATCGTATTTCAAGATTAGCGTTTAATGTT

GTTACCAAGGAAGGTAAAAAAGAGCAACGTCAATATGATCCAGGAGAGCGCAGGTGGTCT

GTATTGGCTATTGCTAAGGGAGAGAATGAAACGGACACTATAGTACTAGTAGCCATTTTT

GACGTTCCGAAATCATGAAGTGCTATAGCGTTGTAGAGGAACCCCCGTCCCTAACCTGTC

TCAAGTCTCAACCATTGATTTGGTTAGAGTAACTTAGATTGCAATGCTGAATTACACATG

GAGCTTGGGCAATTCTCTGGTATGTGGAATGACTAGCGTAGCTAAGGCGAATGAATATTA

AATCTAATGAAGTGCGTGATTAAGAAGTGTGCAACAACCGCGGTGAAACTTGTGATCCAA

TTAATGTAAAGTAGTTATGTACTACGTTTACTTTTATCCATGGATTCGCTGAGTTCAAAC

GTTGATAGAAGAAGGTAAGGTATTTCTAGAGCAGACTCCTAATAAGCATTAGTTCTCCCT

AAAAAGAAGGTCATCCAGCCACACCTTCCAATACGGCTAACTCTTGCCACTAGCCCTGCC

TTCAACGTCCCCCTCCTTGCTGAGTTGCGGTTAAGGTAACTACTCCAGGCATAGCCAGCT

CCGATAGTTTGATGGGCAGTCCATGTTTGGCTTGCCTTGATTGGGACACTGAGTAAAAGA

GATCAAGCAAGTGCTTTGCAATAATAGGTGACTTTGACAAATTCGTAAAGGCGCTGAAAT

AAATTTGAGTAAACTTTTCCATGGCTCAAGGCTTTTCATTAACATGAAACGGTTATCAGA

GACTTGAAGAAAGCCCTTCTGTAACTGCTAATCTACACTTGAGTATAAGATTTAGCTTCT

CTTTTAGTGGAGCTCTACTCGCTGATCTTCCTAAATTGAGAAACTAAAAAGCCTGTCTTT

TCAATACATCTAATGTAAGACTTACCTTTAACAAGTTGATAATCCTCATTTTGTAACTGA

ACGATGGACAATTGATATGATCAGCA

>XLOC_011444 transcript=TCONS_00018683

ATTTCCTCATGTTTGTCCAACCGTTCCCCTCCACTCCTCACCCCACCACCCAAAACAAAA

TGAAAAAATTAGAACCAAGAACCCTTTAGACCAAAAAGGGTACTATTGGACTCCCTCTCG

TAATAGCGATTTCCCGTCTTCTGCGAATGCGTTTTAATTTCCTTTGTTTCCTTTCATGGA

GGTAAAATTGGAATATACTGTCAAGATCTTTCAATGAAAAAGAAGTGAAGTTTTTACAGT

AATCCATCATGTTTTGGGAAGTCTGGAATGAGATTCAGTTTGAAGACGGTTAAGTATGCA

CACATTCAACCTGGTGTTCTAGCAAGGGGATGAGTGCAAAGCTTCAAAGGTCTGACTTTT

TAAAAGTAAGTGCTGAACAGAGAATGGCAAAAAAAAAAAAAAATGTATTGCAGCTGACAT

CTGGGGAATTTTCCTATAAGATTTTAACGAGCAAGTTTAAGAAGCTGAAATTGTTGAACA

AGTCAAAGCAGAAGAGGAATTTGCTTTTTTTTTTTCGTAGGGGGTATCCGGGGTAATTTG

AAGGTGTTGTCGTTTGTACATGCTACAATTCTATTTTTTTCTGTATTAGGATTTGAAAAT

AGTAATATCAACAATTCAGATGGTATACTTTTTGTAAGGTGATACTTTAGTTCTTTTTAA

TTTACAATGATATTTTATATGATTATCGACCA

>XLOC_023015 transcript=TCONS_00037828

GAACCGTTGCATTAGAAAATAAACCGCCGCGACAGAGTTACAAAACCAGGACGGATAGCA

TTACTTAAGCAACGACGACGGTTTTTTGAAAAAAAAGATTATCGCGATGCTTATAAGTTC

CCTCCAACTTTTCCCTCTACTATTTTATTGTGTCCCCACACCCTTTATTTTAAGTACTTT

TTCATTTTTTAATGTTTCAATAAGAGTTAGGGTTAAAGGCTAAAAGCCCTGGTCTTCCTC

AGTCTCTACTTCACGTTCACAAAGCCCGTTCACAAAGTGACCTCCGACGTCGGTCTAACC

TCACTGACCCATCCTCCGACGGCTCCATGCGACCTCCATCGTTGGCTTTTCTCCGCAGAG

CTTAATAGTCATGAATCAAGAAGTGCCTTCCAAAGGACCTGAAAAATGTAGAAGCTGAAC

ATACATTAAAGCTGAAACAATTCGCTCTTTGGAAGAAAAAACAGAGGAGTTTATTTTAAT

ATTTAATTAAAATTATTTTATGGTTTTGCACTTACCCATTGAAACTAATTGTAGGAAAAA

TATGATATCCCCGATGTTGCAAAAAAAAGGACTTTGCAAGCTATTCAAAGTGCTTGGAGA

AGTTATAAGAGTCGACTGAAAGAAAGATCACTTTGACGTTTATGAAAATGATGCAATGCG

AATGGAAAACAGGCCAGAAGATGTATCGGAATCAGTATTTAAAGAGCTCCTTAAATATTG

AAAATCAAAATAAGTACAAAAACTTTCCAAGATTAGTATTGGGAATCGAAAAAGCTGAAA

AATCCTCATACTGATGGCAAGAAAAATTTTGCTATAGTTCGCAGTGAATTGGCTGAAATG

GAGAAAATTGAAATACAGCAAAGTCAAGATGGCAATGAGCTTGTTAATGTGTTTGAATCG

GTTATGAGGTTTGAACATCCTCGACGTTTGAGAACGTATGGACGGGCGGTTACAGAAAGT

TCTTTAAAAAAAAGTGGGACACTATGAACCATCTTTAAATGGTACAAGTGATCAAGTACC

AAACATGGAAGAGATGATGCTAAAAATAGAGGAAAAACTTGCGGAACAAAAGGCCCAAAT

GCGACAGAAAATTCTTGGAGAAGTCTATTCTCAACTTCAGCTTTAAAGAGTTCAAATTGA

TCTTAACATGCTAGCAACGCCACAAGGCACTACCCGACAAACATCGCCCATCTGTTAGTA

GCAACAATCAAGGTGAAGCAAATGAAACGGAGGTGGACGAAAGATGTGAAGAGCTAGACC

GTACTTAAGAGTGTTGGAATTGATAGTTGCTAGACTACTTTTACATATCTTGAAAATCTT

TTGTTCTAGAAGTACCTTTTTTTTTTGTTGATAAATTTAACGCAATTGTAAAAAAGTCAT

ACAACAATATTTTGCTCTAATATATGGATGAACTTGT

>XLOC_031660 transcript=TCONS_00052077

AATAACTCAAACATCATTTTTCCTAAAGAAGAATGATTTGGTATTAACTGAATACACAGG

TGCTGACTTTGCTGGCGATTTGAATGACTGAAGATTTATATCAAGCTATAATTTTCTCTA

TGGTGGTACAACCGTTTCTTGGTGTAGCAAGAAACAAGACAATATCTTTATCTACCATGG

AGGAGGAGGAGGATAAAACAGCTGCTCTTCAGCTTGAAAGAAGAACTTCGTTGCTTTTGG

AATCGAGAGATTTAGGACTTTTTTCTTCCTTTTCCAAGGTGTGGCTCCTAATGTAGGTGC

TGACTGACCAATTTCTATGGCCACACTTTTGAAGTGTGGCATTTTCTGTGCCAG

>XLOC_034233 transcript=TCONS_00056318

GCCCATGTGTCCTTCGTTTTAGCTAATAGACTCACAAACCCACCTCTCCCTCTGATCCAG

CCCTGCTCTCTCTATTTTTGTGTACTGTTTTCCGATTCATTGTCATTTTTTAGTTCCCTT

TGCTAAATTTCCAGGTGCTTCTTTTATTTTGTAGGTTGGTGGAAGGTGAGGTTTGTTGCA

ACGGAGCATTTCAATTTTTAGATACGATTGATTGAAGAAGTGTTGGATTGCTCGTGGGAG

ATATGTTTGAGCTACACGCGTCTGTACTGTGAGTTATTACCTTACCACAATTTGTGTTTC

CATAACCTGCATGGTTTCTACTTGATTTTGAAGTAAAATATATTACTGAATGATTTAAAT

TGCATGTGGGACAAGTCCTTTTACAAGATATGTACTGAAATAATTATTTGAAGTATTTTA

CCGTTACAAGATTGGATTTTTACTGCTACTTGACATGTTTTACCGCTATGTGAAATATTG

AATTATTACTCGAAATGCCCTGTACTTGGTATAGGGAATAAATATATTTTTGGGATGAGC

CTTGACAGACCCCGTAGCTAACGACGGGTACGTTAGACCTGGTGCACCTTGTATACTCCG

GATTACCGTTACAGTCCTTGCTAGTGGGAAGGTAGAACTAGCTTACCGTTACTGATTCCT

TCAGGGAGGAATTTATACTGATTACATGGTTTGTTCTTGGAAATCTCCCTAACCTATATA

CTTGATTTAATATAGCGATTACTGAATTTATTACTGGTTTGATATTGGTTACTGTTTACA

AGAAGTACTTAAAAAGACTGATTTCTGATTGTTATTCTGAAAGTGACAATTAAATTATAT

GTATATTCTGCATAAGATTTACTGGCATGTTTTTGACTTGTCCCCACTTAAAACGGTTGT

GGATAAGCGTTGTTACTCACTGAGGTGCGACTCACCCCTCGCTATATTTTTTTCAGAGAC

CGCAGGTGTTGTAGCCGAGGATTTCCATTCTTAGAGCCGTGAGTTGATATTCCTGGTGAG

CCCCGCATTAGTTCGCGTGGGCAAGAATTTCATTTATTTATTATTGTCCTTCTTTGAAAT

TCTTAGAGTCGCTCCATTTAGTCATTTGTCAGTCATTCTTTTAGTCAGTTGGATAACCAG

TTGGATTATTTATTTATAAGCTAGTCATGAGAGTATTTGGTCTTTATGAACTTATCACAA

CCCTATTAGACGTTCTCGTAGTATTTGGAGATAAATGTAGTATTTTTATTGTTAATGCGT

AAGAATATTGGTTGTTTTTGCTGGGTTTACTGTTTGTTTAAAAGCAGGTAAATTTTGGAT

AGGCCTAAAATAGGGGAAACTCTGCCCGATTTTCTGTAGAATTTTGATAAGGCTTGTTTA

GGGACTTAGATCCTAAGCGCCGGTCACGGCCCCAAAACTGGGTCGTGACACATTAATTAT

TTCTTTGATTACTGCACTTGTGTGCCTGAATTTTATTTTATCTTTACATGCATGTTTAGA

AACAGATACTTAGGTGCATTCGGTATTTCTTTTTGGGTAATTGATGAAACTACTTTGGTT

TTCCAAGGTCATTAAGCCTGTATTTGATCTGTTCCTCTTTAATTGTGAAACTGGAAGTAG

GTTACAGTAAATACTTTTGTTTGGCTTTAGTTTTATCTAAACGAAAACTTTGTTCGAACC

TTTTCTATACCAATAAACTCTTAAGTAGTAATATTTTTACGAATTAAATAATGGACCTTC

CTACTCCTATCATTCTTCATGATGCAGAGTTCCTAGCGAAAAATAATTTTTTTTAGGTAA

CATGCAGAATTCGAATTAAGCTTACCAATGTCAAAACATTAAATTCAAGAATCTGCTTTC

GTCATTCTTGATGTTGTTCAACAATTAAGAAACCCTTTTTATAGAAAAGGGGCGATTATT

AGGTAGTAACTAATACTTTACCATGGACTATTGTCTGAACTACTCCTTCCCTATCCTGAG

TAGGTGACTCCCCGTTTGGTGTAATAGTCACGAAGTCTTAATGTTTAGTTCTATTTTGCT

TTATTTTCGCTCGAACGAGCCTCTATAAGAATAGAAACTCACAAGAAAGGAGAGAGAACA

AAAGAAAAACATGAAACAAAGTTGCTTCCAGATTGTAGAGAAGAAGCTCTGACCTCGGAT

GGTATTGGCACGTACACTGTTTTGGTAATAGGATTGGCACGTACACTGTTTCGGTGCTTT

GAAGAATAGGATTTTCACTATTTATTATGAAGCCCCTACTTTAGTTTGTAATCACAAACA

GAACAGGTGTCATCAAAAGGGCGCTTTGGACCGGAGTTGTTGGACTGATGACACGGAGGC

TGCGAAGCAACTCAAGGTATGCATTTAGGTTGTGACTTGTGTGCTTTTCATGCTTATTGT

GAAGTCACCGCCACTGGCATTGACTCACAGAGTGAGACTAAAAAGGTGGAACTTCATCGT

ATATATTGAATTTTAGAACCTTAATAAACTAAAACAAACATTACGAGAATAACCATAGTG

AAGCTTTTTTAGCCTGCTTTGTATGTTAGAAGCATTGGGTTTGCAATGGCACTTCAACTG

TGTGTATTAGAATTTGGAGCTATGCATTAGACTGAGCCATAAATGATTATTGGTTGATTT

ATACAGTCGAATGTTTCAAAATCTGTATTCTCTACTCTAGAACTTTCTGCATCGATGTTT

TTTGTACTTTTATGTTGTTTTTTTCCAATGTTAATATGTCATTGTAGTTTTAAATGTTAC

ATAAAATCTCGAGGAGATTAAACAAATGAGATCCTGAATGAAATACCGAGAGGGACGTGG

TGATGTTTGAACTGGAAAATGACTTGTGCAAGTTGCAAAAGAAAGTTGAAGCACTTTGAG

GACGAGTTCTCAAGTTATGTTGGAAAAAGGACGGCTATAAGTTGAGCTCAAAAAGAAAAA

GACCAAATACAACCTATACTCATGTTGCTGACTTGAACTTGAAAGACAAGCAAATAGTTC

AGACAAATGACAAGTTGCTTGATACTTTTATGGGATTATCAGAAATGAAGAATCAACCTA

TTATGAAAGTAATATATGCAGTTAACTGAGTTTGGAAAGGATGCTTTAAAATGTGAAAAC

ACTAGGTTGGGAACAAGAGCAGAGCAGACATCCCCTCGACTTCAATGAAGAACTTGATGA

AGATATCAAGATTCCAACGGAGAAGATTATTTATTCTATTTATTGTATTTTTCTTTAAGA

AAAAAAGAAACTTGATATAATTGTTGTACCAGAATGTAGTGTGAATGTAACTTCTCATTT

AGCTTTTAAAACTTTGTTAATATTCATTTTAATCGGAGAATAATGTTCAAATAATGTACC

CAAATATAATATGAATATCTGAAATAGCAATACCCAGGACCCCGTGCTCACGGGCAGTTC

ATCTAGTA

>XLOC_036625 transcript=TCONS_00060098

AAGCACGATCTATCAACAAAAAATCTACAGATTTAAGCATCAAAGCTGAAGGGATCAAAC

GTATGTTAATAGCTATTCCATCTTCGGCACTACCATATTGACGGCTCATCAAAGCATTAA

AAATGTCATATAAAAAGGGGCATAAAAATAAAAAAGAGAGAAGAATAATTAAAAGCATTG

TGATTCCTTGGAACCATCAAAGTGGAATAGACCTCGAAATCATTCAGGTTATACAAGATC

TGTTATGAGTTGCACTAAAATTTCAGCCATTTCTTTTCCTTTTCCTTGCTATGAACTGCA

TTCGATGCAGATGAATGAGCAGCTTCAGCACTGGCTATAGCTTTATGAGTTTCATCTAGC

TTCCTCATAGCAGCTTCATAAACCAGTTTCTCATTCCTGTTCCGCTGCATCATTTCTTCA

AGTTTCTGAGCCCGTATCCTCAACTCTTTTATCTCGTCATCATACGCAACCAGAGCCCTC

CGATCTTGTTTCTTCTCTTCCCTGTTTGCCGTGCTTTTCTGCCCCTTTACTGCGCTGTTG

TTCTTGGTTTCTCCATGCACTGCAAGATGAGTATTGATAAGAGTGAACACATCTGGTCTT

GCTTCCTCTTCTTTTTTTGCAGCCCGAGCTGCTGCTGCAAATTTCTTCTCACGTTTCCTC

TTGCCACCCCTACTTCTTTTCTTACCGTTTCTGTTTTCTTTATTTTCAGGTTGCTGGTTT

TTAACAGCATTGTCAAGAGATTGTTTTGGTGGAAGAACTTTCACAGGAATAGGATCCACC

ATTCCCTGTCCAGATGCGCCTAAACCCATTCCTTCACGGTATCCCATGTTTGCCATCATC

CTGGAAGCTATGCCGCGTGTGTGATTCTCCCACTTTGCAAAAAGTGTTGTTTCGGTCTGG

ACACCATGCTGGAGAGCTGTGCTTTCCACAAATCCCAACCCTTCTGGACTATCATCTTCA

TAGTCACTAGAATCAGATTCTTCGGAACCTGAGTAACTCTCTTCATCACTCGCCTCTGCA

TGTTTGGAGAGTTCAATATTTTCAGCTCCAAGCATAGCAGAACTTCCATCATCACGGAAA

ACAACGTGACCTAGATTAAGCTTTTCATCCCATGACTCAAGTTCAGCTTCTTTCCAAAGG

CCAGCTCTGCAATCAGGGAGAGCCCAGATCCTGGAACCTGCAAGTGATGGATCCCATTTC

ATAGGGACATACTTCCTTAATGAGGATATTGGAATGTCGATACCATGTGATAAGCGGCAA

TTACTACCAAATCGACATCGCTGCTGTAGAAAGAACTTGCACATCTGCACTAGGGTGGTC

TATCGTTAGTAAGTGATATGACTAGAAGCTAAGCACTCTGTGACTAGGAAAGTTACAAGA

AGAGGCAAGAAATGAGGACTTGCACAATCTGAGACATAGTATAAAAGAGATATAAAAAGC

AACTTCACTGTTTCCTTGATCGAAAAACAACTTCATTGTTTC

>XLOC_030332 transcript=TCONS_00049896

CCTCGCCAGTCCATAAAATTTTAAATTTCTCTCTCCTACACTGCTTGGGAATTCCCTCAC

CAAAATTAAAAAAAAAAAAAAATAGCTTGGGAGTTCCCTCGCCAAGCAAAAGAAGAAAAT

ATTCTCTTTCCTGATTGGCTTGGGAATTCCTTCGCCAATCAAACACTGTCAGATTTGTCA

GGTGTTTGGCGAGGGAAGTCCTTAGCCATTCAGTAAATAAAAATTATACTCCTCCTTCAC

TGCAAGCCACAAAACAAATCGCACACATCCAGTTTCCATCCCAAATAAATCGCCTTCTCC

CTCATCACTGCCTCGTCCGTCTCCGGCAACTCCACTTACTCTGGCGACTCCATTTTTATT

TCTCGTCCAACCCATCGCAAGATTCTCCCCATTAAGGATGATGCTGAGTTTGAGCGACAA

TTTTCCACTGGGACCAAGACAATTGTTGTAATGACAGTTGAACCGCTGAGAGTTCTGCAA

TTTGTTTTATTATTATTTTTTTTTGGCAGACAATTGTTGTAATGACAGTTGAACCGCTGA

GAGTTCTGCAATTTGGTTCTACTAATAAGGTTAGTAGTAAAGTCTATGTTCAATAAGCTG

AAAATATCTCTCAATCGACTTGTTCAGCATAAAGTCCAATCTTCTTTAGGCTATGACAAA

TTCTTTCACTGATTCATTAGTCATTGAGAGGAAGCAATAAGAAAATTAAAATATGTTGTT

GTAG

>XLOC_016429 transcript=TCONS_00026948

ATCGTGAGCATTCTGAACAGAAATTGGTTCTTCTTCCTTAGGCCTCTAGCATCCGATCAA

GAATTTATTGAGCTCTTTAAGATAAGATAAATTAAGCTTTTTTGTGATGTTGATTGAGAA

TGAGCCGGCAAAGAAAGAGTTCCTCCTCCATCAAGTAGCAGAACTGATTCATTTGATCCT

GCGAAGCTGAAATAAGTGAGGAAAAACAGCTGTAAAGATATTAGTTCCTGACTGCAATAT

GTTCTTGCTCCGAGTGATGGTCGGACATTGGAGAACATAAATTGGAACACATAGAGAATT

TGAAATTTGAAGAATCCTACCTTGAAGACTACTCTGAGCACGTTGGTGTAGCTGAAAAAT

GTTAAAGACCAGTTGAAGCACAAGAACTTACTTTTTTTTCTATGGCTTATTTGAAGATGC

TACCTACAGTTCATGCTACTGTTTTAATTTATTTTCTTACGAGTTGAGAAAATAGTGTAA

TATTATTTGGATGGGAACAATAAAGTTGTACATAAATATATTGATTATATTTTATTTTTG

AATGTGTGTATACTGTATATATATTTGGAAGCA

>XLOC_008218 transcript=TCONS_00013384

ATCAACCAAATTGGTACGTGGGCTAAGGGCATAAACAGCCCACATTAGCGAAGCTCTGTT

TCTTTCATTCTTACGCACAGATTCTTACGCACAGAGACAGTAACATAGAGTTGTTCTTGA

GCACTTCCTAACCAATCCACCCTTCAAGTTAACATTAAGATTGAACCTACTCGGATATAC

TAGACTTGTGGAGCATACGGTTGGCTCAAAGGGAAGA

>XLOC_016232 transcript=TCONS_00026588

CTTAAGTTCGTCCATTTTCGGCATATAGAGGTGACCTTCAACTCTTCAAAAAGTTCTTGT

GTTCAAACTCTAGAAAATTCCAAAATAAAAAGGTCTACTCTTTATTCAAGTTTCCAGCAA

GACGAACCGATAAACCAAAGTATACTGGCTAACCGACCAACCATTAAAAATGGAATAAAT

AGAGGAGATTGAATTCTTCTCTCGTTTTTAAACCAGAGCATGATGACAAGAATTGGTTGG

GGAGATGTCCTTGAAAATCACGTACTCTAGTGTATACTCACTTCTTGTCCTCTTCCTGCA

AAAATTGTTAAAAATACATTGAAGAAAATACAAAATGTGCTATCTAATTGTCATTCCAAT

GAAATCTATGAGCAAATCCAACTGGTGCTCCTGGGCTTCGTTGAACTTTGAAATTTGTTG

CTCCTGGGCTTTACTGATCATCAAGAAATCACTGGACAATTGGATGGAACAGGTTCGACA

ACAATTTGTTGTTGCATTTGGAATATAATTGCACGCTACCCCCTGCTTGTTGCTGCTACA

TGAGGTTGTTGTTGTTGTTGTTGCTACATGAGGTTGTTGTTGCTGCTACTCGCGCATAGT

ATCTGCCTTTTGTGCTTGTTGCATTCTTACTCTTTAAATACCTGGGCTAAGTTGTCCTAG

CGTGAATGTAACAGTATCAAAAATTATGCTTCTTCATTATGCGCACTTGAATAGAGGTGT

AATCCTTTAGATTCTGCTGCAGCTTGCTGGATTCTACTTTGGATTTCCATGCTGCTCCAC

TTGACTTGAAAAATTGTACATATTATATGTATGAATATGACTTCTAGCTG

>XLOC_028910 transcript=TCONS_00047507

ATTATGTCTCGCCGTCCCGTGATTCTGGTACATTTGTGCTTCCTCTTTCTTTTCCTTTTC

TTTTCTTCTTTCCCAAAATAATAAAATCGTAACCCAATTTTGTTTTCCCTAAATGGTAGG

GCAGCAACCCGTCCTCACTAGATCTCCGCCCTCACTGGTCCAACAGCCGTTACCGGATCT

ACCAAATATTTTGAAGAATGACAAACAAAGGATTCGGTGACTTAGAGAAGAACTTAATCC

TTGATGAGAAGGACATGATGAAAAGATTTTGTAGAATGCAAATCCATGCAGAACTAGGGG

TAGTGGCGCGTAGGCTTATGCGCATCCTAGTCAGGTAAAAGAAGTTGTGCCGGGGCTGTC

TTATTGAAGTGTGAACAAGAGTTAGTGCTGAACAAAAGAAGTATCACAGGGAAATCTGGA

CTTGTCATGTTTACAAGGTTTCCTAGTGAAATACTGCTAGGGTTACAAAGCCCATATAAA

AGCAAGTAATTGAGGCAGCCGATCTCAGTTATTCACCACAAGAGTTCAGAGCAAACACAA

GGCGAATTCAATAACTCTTAAAGTGACGTAACTGTTGAAGACAACCAGTGAAGAACTAAG

AGCAAAAGTAGTGAATCGGTTTAGGTTCTTTATTGGAGTTGTCAGAGTTTTTATGCTTTG

ATTGTATTACCTATCTGCTTATGTTAGAAGTCATTGTAATAGGTAAACTTTGAGTGATTA

TTTTGTAAAGCTAGCGTTTAGCTTTGTTGAGTGATTAGGGTACAGAGTGTAGCCCCTTCG

TTACAGTGTTTGTAGTTTAAAACTTGCTCTTCATGTTAGTGAAGCGGTTTCAAAATCCCA

GAGTGTAGGTCGTGGTTTTTCTTCCTTGAGCAAGGAGTTTTCCACGTAAAAATACTGTGT

CTTTTGGATTGCTATTTACTTTTATATTTGTGTTTTTCCGTGGAGGCAACAACTCAAGAA

TAGAGTTCTTGCAATTAAGAGTCAGGTTCTTCGGTGGAAATCGGAGGATACTATTAAAC

>XLOC_002344 transcript=TCONS_00003767

TAGACTTGGTAACTATTTCTGTAATATTGGGTGGTAATGGTCGGCCTGGTATTTTCGTAA

ATCCGTTCTCTTTTAAGGGTATTTTAGTAATTATGTACCCATTTATTCTTCGTCAGTATA

CGTGTACCTCATTCCAAAAAGGCAAACATCTGATCTTCTATGCCAAAATGCCAACCAGTA

TCTAGAAAAACCTTACCACCACTTCACAACTCCTGCTCTTCTAATTAGTTTCACGTTAGC

TCCTCTTCCAAGTTTAAATCCTGCTCTTCTAATGCTTAGTACATGTTTGTGATGCTCTTT

CATTGAGCTGTTCTATTTGTTGATATACTCTCGCCATTCTCCTACGCTTTTGGTTTCTCA

TTTTTGCTTCATCTTATTTATTTCAATTTTTTTCAGTGTTGTGATAGTTGTATGTTTTTG

TTCAATGGAGTTCGGTAAGCACGATTCTTCTAATTAGCTTTTTCTGTTTTCCTTCTTAAT

ATCAGCGTTGACATCCCAAAGTCTATTATCATCTTTTGTTTTAATTTATTCATTTCAGGT

CTTCTCAGTCATATTTGGACACACTTTTCTATTTAAAAGGAAGTACTAATTGAAGCGACA

AGCCGACAATTGTACCAGGCCCACTAGAGGTACCTCATGGAATGGAACTTATTTGCATCA

ACCAGTTGGTCTCAAGATTCTCTTATGTGGAGTAGAGGCGTCCTAGCCTTCTCATCCAAG

GATGTTCCTCCTGGTCGTGATGAAGGCTATTTGCTACTTTAAATTCATTTAACTCGGACA

ATATTAATCATTAGCTACTCCTATATGGTAGTAGAACTCTATTTTAGAAGTTTTTCTTTT

TGGAGTGGTAACATTTTTAATTTGTTTCTTCATTGTTGAAATCTATCATTAGACTTGGTG

TTTTATTAGCTTTTCGAAATGTGTGCATATTTGATCTAATATGTTGTTGCATCCATTATC

AGTGAAGTAAGTTCATTTTTTCATTCACCTCATTTTCTTGATTAACATGTACAAAATATT

GAGCCGC

>XLOC_011505 transcript=TCONS_00018783

ACCATTTTTTCAGATCTAACCTCTCATCCTTGTTATTTCTTTAAATAATATCATTTTTTC

AAAAATCCAATTGCACACATCTCATCGCTCTTTTACCCTTCAATAAAGCTATTATTTTTA

CATAAGTCTTTGCTAACAACATATCAAAAACACACAAAAAAGACATGTTTTTGCTAAATC

AAAACCAAAGATTCGATTTTTAATTTGATGGTGGTGGTGTAATATGCTTGGATTATAAAG

GGGCCAAAAGTGCAGGCAGGCTTATGAGATCTCAAAGGATGGACCTTTATATACAACAAA

AAGAAAAACAAGAAAAACAGTCGTGAAAAGGAGTTTGTTATCCTAGTTAAGTCAACCACG

ATCGATGTGGCGGTGGATGAGACTCATTTGCGAACCGGAAACGTGGTATTTAGATTTGAA

ATTGGGAGAGGAGTTGTTGGAGGATGATAAGTTGATGAAGGAAGAAAAAGAAGAAGA

>XLOC_021949 transcript=TCONS_00036085

GCAGGAAGAGCCTCCCCTTCCTCAGGAATTAGTGCTGGAAATTCTGTTGCGGGTACCCGT

AAAAGCACTAATTAAATGCATTTGTGTATGCAGAGCATGGCAATCTCTCATCAAATCCCC

CTCTTTCATCACTTCCCATTCCAAATATCAGTTGATGATAAACAAGCACTACGACAGCAA

GCTTGTTTTCCTCATTGAAAGAGACAAAGGTTCATATTGTTTGCTCTCCCATAATGGTGC

TGCATTTACTAAAGTAGAAAATACAAAATTCCCATTTCTTTTTGACTACTATAATTGCTT

CATTACATCCTCTAATGGGTTGCTCTGTCTTGACGTTAAAGTTGACTTTGGCCGACATAT

TTTTCTCTGGAATCCTTCTGTTAGAAGATTCAAGTCCATAGAAACTACTACATGCTCTTA

CAAAATTACTACACATTATGCATTGGGTCTGGATGAATTTCATAATGAGTACAAAGTTGT

CAAGATTTATGGGCAACCAACTAAAGCTGATGTTTACTCTTTGACCACAAACTCTTGGCA

TACCACACTAGTTGGGAATAATAAGGATATTAATGCTACTATTCTAAATAGTCGGACACA

TGCTCGTGCCAATGGAGCTATCCACTGGTTGGCATTTTTTGGGAATAGCACAAACTATCG

GTTCTTGAAGAGTGATCTTGCTATTTTATCTTTTGATTTTACCAAGGAGGTGTTGGGAGA

TTTTTCCTTGCCAGCTGAGTGGTGTAATGACCAAATTAAAGAGGCTCTTGAAGAGGATCT

TAAAGTTCTCAACGGCATGCTTGCTTTCATTGTTAGCTGTAACTCGTTTGTTGTAGAGCA

ATCAGTGTGTTATTTGTGGTTGATGAAGGAATATGGTGTTGCCGAATCTTGGACTAAAGC

ATACACTATTGTCCTTGGGACGGAATGTTATTGGCCATTGGGATTTACAAACACAGGGAA

GTTTTTGTATTACTTGTACGATGAAAGTAGAGTCATGTCTTATGACCCTGCTAGTAAGAG

ATCTACCAATGTTGGGGTTGAGAATTCATTAATAACTCAAGCATTTTTTGACTTCACCGA

AAGCCTCATTTTACTTGACCAAGGTAGTGTGTCGATGGAGCAGGCACTGAAAGATTATCA

AAGTAACAGATCTACCAATGTTGGGGTTGAGAATTCATTAATAACTCAAGCATTTTTGAC

TTCAAGGAGAACCTCATTTTACTTGACCAGGTAGTGCGTCGATGGAGCAGGAACTGAAAG

ATTATCAAAGAACAATATAATCAAGAATAACAGAAATCCTCATTTTACTTGATCAAGGAA

GTGCATCATGGATTATCAAAGAACAATACAAAGGGACTTGAAGCTGTGTTTTCTCGAGGT

GTTTTCGCAGATTGATCAACATTTGTATCCGTCAATACCTGAGGTTTTGAATTGAATTAG

CTTTCAGTTACCTTCTGCATAGTCAAGGCTGCATAATCTTAAGCAATGA

>XLOC_034054 transcript=TCONS_00056023

TTTACGTATTTGCCCTCATTTTGTTTTCATTATTCTGTCACGCCCCTCCTATATAATCTC

TTTAATCCGAAACCCTACTCACTTGTCTGCATTCATCTTTCTCACTCACGGGCTCTTACC

CCTCGGAAACCCAGGTGAAGACATAAGCAAAGATATATCTGCAGTGTTACATTAACGAAA

ATGGTGACAAAGTTTACACCACTAAGAAAGAATCACCTCTGGGGTTGGCGACACAATCTG

CTCACCCAGCTCGCTTTTCACCAGATGATAAATTTTCAAGGCAGAGGGTGCTTCTGAAGA

AACGTTTTGGCTTGCTTCCAACCCAGAAGCCAGCTCCAAAATATTAGCAAATTTTAGTGC

AACCTATTGCTCACTCCTTAGTTATGTTATACAATTGCTGGTCGAGTGCATGTCAGTGAT

TCAGACACTTCTCTTCTGATATCTTTCAGCTCTTACTAAAATTGCATATGTTCTTACAGA

TGTTTGTATTATGAATCTTCATTGGTATTTTGTTGAAATAGTGGACGAATGTGCGGTCTG

AAGCT

>XLOC_026813 transcript=TCONS_00044120

GTCATAGCTCTGTCATTTACCTCATGCATCCTATCCTAACGGCAAAATAAACCAATTCCG

TTAATTAACATACAAATGTCTTAAACCCTAATTCTCAGTCTACCACCTTACAAAATCTTT

ACAAGCAAAGCAGCAAAAAAAACCTTCAAAAAAATGGGAATCCCCGCATTTTACCGATGG

TTACTGGAACGGTATCCAAAATCCGTTGTCGAATGTATCGAAGAAACTCCAGCAACCGTT

AACGGAATCACTGTCCCAATTGACACCAGTACTCCAAACCCTAACGGCATTGAATTTGAT

AATTTATACTTAGATATGAACGGAATTATTCACCCTTGTTTTCACCCCGAAGGCTTGCCT

GCTCCCGAGACGTATGATCAAGTGTTTCAAGCTGTATTTAAGTACATTGATAGGATTTTT

TCAATTGTTAGGCCACGAAAGCTTTTATTCATGGCGATTGATGGTGTTGCGCCACGGGCT

AAAATGAATCAACAGCGGGCAAGACGTTTCAAAGCAGCTAAAGATGCGGCTGATGAAGCC

TCTAATACAGGGAGAGTACGAGGGAATGATGAAGCAGAACGGGAAGACTTAAGTACTGAG

AAGTTGGATTCAAATATTATTACACCTGGAACGAAATTTATGGAACTGCTGTCATCTGCA

CTTCAATACTATGTACGTTTAAAAATGAATGCAGACTCTGGTTGGCAAGGAATCAAGGTT

ATTCTCTCTGATGCTACTGTGCCCGGTGAAGGGGAGCATAAGATAATGTCTTATATTCGT

TTGCAAAGAAATCTTCCAGGATTTGATCCCAATACAAGGCATTGCTTATACGGTTTGGAT

GCAGACTTGATTATGCTGGCATTGGCAACACATGAAGTTCACTTTTCAATTCTAAGAGAG

GACGTGTACAAAGATAAATCAAAGGATAGAGGTCAAAATAGTAGGAATTATAAGCATAGA

CAACAAGCAGGTAAGCACTCAAAGAAAGGGGAAGGATGTGGAAACGAGTTGGAGAACTTA

ATATCAGAGCAGACGTTTCAGGTTAGAAATTTGATTTAGTATGACGAGGTAGTACCATTG

TGTTATTGAGCTCCACCACTGCAATAAGCTGTAGGAATTTGATATGGTTGTCTTGTTTTG

ATAGCTGAAACCTTAAAAGCTGTTCAATATAGTTCTATTATGCAACTATGAGAGGAATCT

TTCTAGTTTCAAGAAATGTGGAAAACCGTTCTGTGTTTTGTGAATTCAAAGAGCTGAAAT

CTATGTTGGTTCTGAATAAGAATTTTTCTCAGATAATTCAAACCAACTTTTTTTTTAGAA

ATGCTGAGGGT

>XLOC_013227 transcript=TCONS_00021681

GCTACACCAGTATCACTTTCTTAGCTCAAAATAAAACCAAAGGACATGGGTCCTTTCCTT

GAAGCCAACGTTCCAGAGAAGGAGATTTTGCTCCTATTCAATTTCAATGTATACATATAC

AATCTCACTCATATCTAGAGGATATCAAAGCTTTCTTCAGAGCTGTCATGGTGGATATAT

ATGGACCTTGCGTATGAATGAATTGGCCATATCTCTTCTGGTGTAAACTAAGTAGCTGGA

AAATGGTATGTTAAAATCCTTTTAATGCTTTCGTAGTTTCTCATTTTTACTCTGTAAGTT

CTCTAGATTGCATTTTTCTCTGACTATTTTCTTCCTCTACGATATGATAAAAGATTAATT

TTGATTTTTCTGCTTCAATGTCCAGTAATTTGAACAATTAGTAACATCTTTGTTGGCCAA

AGAAATATTTAAGCCATGTAAATCGTAAGTTATCACTACAGTAAGCATTTTGTGCTTCAT

GATCCTTAGCATCAAAAAATTTACTATCACATTTACCCACATAGCCTTAATTAGAAAACG

CAATCTTTAGCAGTGAGAATCTTGAGCTTTTAGCAAAATGATATAAAAGAGTTGTTCAAC

ACAATTTTAAAAAAAAAAAATAGAAGGAAAAACTTGGTTCCATTTTCTTCCATATTAAAG

TGCGATCTTTCTTTTGGACTTCATTGTAGCACTTGTACTTTGAAGATTCTGTAAACTAAT

ACAATTTCTTCCATTCCACGTGTACTTCTCAGCATTTATCACAACACCGCCAACTTGAGC

TTGTACAGTCTTGTGAGATAAATCATCCTCCATTGGCCTTCTTGGCTGTAAGTAAAGTAA

ATAACAGAAATGTAGCTGTCTCTCAATCCATTTAATAACCAATACTTCAAGGTTTCATAC

TTACATTGAGTTTGGAAGGTTCATGTATATGTCCAGTCCAATAAGTTGACTCAGAATGGA

CTTGGTTACTTGGGTAGTATTGATTTGAAGCATTTTGATAACAATCTTGAACTGTTTCAG

TCCTTAGATGCCTCCAAGTCTATTTGTACCCTATTACCCTCACAAAAATTATTAGCATTG

GAGCTCACTTGGGTTTGTATTTTTGGGACATTATCAGCATGTGTGATGTATACTTCAGCA

ACTCTGTTAGCGGGAAGTGTTTTTACCATTAGATCACAATCTTCATCAGTTAAGCATTGG

ATATAACCATTACCTGTTTCTGTTATCGGCAGTTTGAAGTGCCAACCAAGAAAACCATTG

TACACTAATTGTTTTGTTCCAATCAAGTCCAGAGTTTGCAATGAC

>XLOC_018057 transcript=TCONS_00029565

AGGAAGGACGTACGAACACGGCATAAGATTGTTGTACAAAGTGAAGCATAAATTGTACTA

GAATATTTTATTAGGCCAACTCTAAGATAAACACGTGTATCTCTCTAGACAAACAATAGC

TCTTAGCTTTACATTTCCCTGTTCTAATTCTCCTCGCTGCTCACCGAATCGTTTTAGCCT

CTCTTTCTTTGGGTGTTCTACAATATAATAAATACTCATATTATTCAGGACAAAAGACAT

ACTACTATTTATCTAAATGGTAAGGATCTTAAGAAAGAAAAGACAGCATCTTTAAGGTTA

AAACGTGATGCACAAAGCCTTCACTTTCTAATTATGGCTCTCAGTATTAGTAATATTCAA

ATTCTGTATACCTATTTTTAGTTTGGATAATAGTTACTTAAACTATATTGGTATCTTTGG

AATAAGACAAAGGGATTGATCTATTTAGGTTATATGTACCGTAAAGTAACAAGTAGAAGA

CTTTGGTAAGTGATTGCATTGATGCATTAAATTGTCTTTGCTGAATCAAACCGTTCTCTT

TTTGTTTTCATCTTTTATAGTTCATATTATGTGCTTGATGATATGCCCGAAAAATATTAG

TACGTTATTTGGTAACTAAAACTTTTAGAGTTATTGTAGAAGAGAGAAGTAAGGAAGAGA

ATATTTAACTATAACTAAGAAGTACTATTTTATAAAATATAAGTCTATTTTTGTCCTCTA

AATTGATCATGTGAGAAGGGAGTATATTGCTATCTTATGCACATGCATTTTCTTTTAGAA

TTATCTAATGCAGATTACAAAGTCATTTTTTGCTGGTGTGGTCAGTCAAGCATTTTGTTG

ATGTGATGACCAGTCAGTAAGAGTGTTTGTTGTTTACTAAGTGCTTGGACACTCTAATGG

TATAACCACACTGGTAAGTAATGCAACACTTTCTTTTAAATCATTTCAATTTTCAGTTGA

GTTTTACCATAATTGCGTGGAATGTGTTGCTCTTTCGTAGATATTTACACCTATGTAAGC

TTCCTGTTTCATGTTCCTATATTTGTCTTGGAACTCTTCCTCTGATGTTTGGGATATTAT

TTGTCCAGCATAAATGTGTCTATTTGGAATCTAAATTTGCAAAGAGCTTAGGATGCTCGG

TCACCTATGCCGTGACTCTGAGTTCTGGTGGTTATATTGAATAGGTGATTATATATTACT

CTTTCGCCTATAATATTCTATGATTTATTTGGTTAGCACTCAATTTCTCTTCCATGTCTA

CTTACATTCATTAGACTTTATGTGACCTTGATGACATTAGGGGCTGATTGCTTCATTCTC

TGATAGAATAGTACTATTCCCTTTCATTCGTCAACTTTCACACGAATCTAACCTGAAGGA

TATTTTTCAATGTCTTTGACATGATACGTTGGAAATATTTCCTTTCAAAATTTGGTTAGC

ACTTCCATCCTGATAGCTTGAATTTGGAATGAAATACGTAAAGCCAAAGCACAACTTGTC

ACAATGAATGAATGATGCCATTTGGTTACTAAGCTACGCTATTTACCAATGGCATCATTA

TTCAAATTGATAATGTATCAAATGTGTATATGGAAAAAGAATCACTTGCACGATTTGCTT

ATAAGATCAGGAAGTATGGAACTATAAGAGAAAGCTGAGATAATGGCTATCCTTTTTCAT

TCTTGATGTTGTTTTACTAAAGCAGAAGGGTAATTAATTGGTACGTAATTATTTAGTAGA

AGGTTAACTTTTCTGTGAGAGTGATGTATAGAGTTTGTTTTATAGTCACATGTATTCGTA

GATTTTTTTCTTATATATAAAGTAGTGAAGTACAACTCAAATTACTTAATTTCCCGCTAT

GTCTTTATTTTAGATCCCAAACAAACATTATACTTCTTTGTCTGACATATTATATGTTAG

TTAATATATACTTTTGATTCATTCTTCTTCCCATTCCTATCATTTTTAATCGTCCTTTTT

TCTTCATGTTCTCGGTTTTCACTTTAGAAACAAAGTACAAACACATGTTTGGCCCACTTT

ATTTTCCATTATCATGCTAGAATTTCATGCATCCCTTAGATTGTAATATATAAATTGTAA

TTTTATTTGTTTTTGAAATGTTTTCCAGAGCATTCCCCACATGGTTGTTTTTGGAATGTC

AAGGCACACTACGAACATGGAATTAAACTAACACATAGGAGCTGCTGAATAAAAGAGATG

CTTCTGATGTTATGCTTTACATTTCCAGCATTTTTGGGTTATTAGTATTATGTAAATTTC

CTTTGTATTGTACAAATATATGTATAATTATATTTGCTGCATTATCTATCAGAGACTTCT

CATAGAGATGGATTGCCTTAGTAATAATATGTTGAGCCCGTGTTTAACACAGGCTTTCAT

TTCTAGTACATATATAAATGAACAACAAAT

>XLOC_031986 transcript=TCONS_00052590

GACACCTTCATGATTTCATCAATTCACTTGCGGAACATCTATAAGTATACCTTTCATCAT

TGAAACAAACTGCACTTCTCAATCCTCAAGTTAATTCAACATAGTGAAACATGACTAGCA

AGTGCTCATCTCATACACTAGCTTTCTTCTTCACTATCTTCCTCATGATGGTTTCCGTTC

ACGACCAGGTTGCAGCTCTGCGAGACCTACGTCCAGAAATACAAATGCTTGAACAAGTGA

TACTCCCAAATCTTGGGAAAAAAGGTTGTGGGCACAGATGTAAGACAAACGATGGTTGCA

AACCTCTTACATTTTGTGTGTCATGCGAGTACGTCGAAAAACTTAACAGGACGGCTTGCG

TTCCAGAGAGCAAAATGGAATTAATGTAAGCAAAAGATATATATGAGGGAGTAGATTGAC

TCTCCTCATGATCCTCGGATATAGAGAAATGCTGGTGTGCTTAATAAATCTATCATTTCC

TATGGTGTTTATGTTTTTTCGTTCCTTATAATATCTAATTTTGTGCCACTGAATAAATTA

GTAGTACTATGATATTTGGTGTAACATTTGGTGCTGGGTTGAAGATACGTACAAATGTAT

TGCTCTTAATTTTTTTCAGAAC

>XLOC_010386 transcript=TCONS_00016909

GTGATAGAAGGTTTTTCTCGGTAATACTCGAATACCTAAAGAGTGCAGTCTGATTTGTAG

CTTATATCTTTGGCAGTTCAGCAATGGCAGTAGAAATATCATTTGGAAAGCTCAATTGCT

ATATTTACATGGTTGTGTAATTTATCAGTGAAATTGTATCTACACGATTGGGATTATTGA

CCTGACGACCAGTGATACTCAAAAGAGCCTCTTGATTGAAGC

>XLOC_025698 transcript=TCONS_00042263

CCTAAGTTAGTCTTCATATCTCTCGGGGACATATTGCAGAGATAGTTGATCTTGAGGATG

ACATGAATAGAGACAAAAAAGACTATGAAGGCATCCTTATAGACGGAGTCATCCTTCCTC

GTGAAGAAGAGATTCCACATGTTGTCGAACACGTTGACTTGTAGGTCAGCCCAGTAATTC

AATCATCTAAGAAGAACATCTTTCCAGAAAAGGGAGGCTTGTCCCACATGATTAATCCTT

TCGTCGCTCCCCCGAGGGTGAATGGTTCAAGAGGCAAATGACGAATGACTTGATGAACTT

CCTTACCGCTACTTCCTTACAGGAGTGAGTTGAGGTCACCGAGAAGAAGCTTGAGGATAT

TAAGACACAAGGCGTGGTGAACCTTAATTAGTTTGTCATGAAAACTTGCTATGACACCCT

TTTAAAGGTTAAGTCTGAAGGCTTTGACATAGATCAAGAGTTCGAAAAGATGCAGAAATT

GGTTCTATGTAAGATTGATGCCGAGTTTTCAAATGAAATAGTTGAGGAAGAAAATAAAGC

CCATGAAGATTCTTCCACACATTGACCATCAGAATTGTCACTTCCCCTGTAACTCGGGCA

ATTTGTGCTTCTCCTGTAACTCGACTGACCTTTCCCCCAAATATTTTTTTAGAAATAGTT

CTACTTTCCCTTTGGAGGCTTCGAGCATTATTTTGACTTAGTAAATTTTTGTCCAGAACA

TTATGTTCATATGCTTAGTTAAATTCTACGTTATGGACTTTGGGAAATTTT

>XLOC_030725 transcript=TCONS_00050554

CATATCTGTAAGTATATACTAACCTTTACGTACTAAAAACGTAAATACATACTAACATAA

CGTAAAATGTCGTTAGAGTGCAAGTGAATCAAACACCATTGAAACATTATGCTTCATCCA

TTTTGATGTCTGCTTCTGCATGGAGATCAAATAAGCAGTCTATGGTATATGTGTTATCGG

GGATAATGGATTTCCATCGAGAGTAAGGGGGGCTGAGCTTGAAGGTGAATTCGAGCTTGG

ACGCTACAAGTACTACTACTGCGGCACCGATTGGTCAGGGATTAAATTGATGATAAGAAG

CATATTTAATGGTAATTTAGGGTTTTGAAGTCTGGGTAGAAGCCTGAAGGTCGTGCGAAC

GAAGGCCAGATGAAAGAATGACAAAACGACATAAACAACCAAAGCTACAGCATGCAACTC

AGAACGTCGTGTGACCCTATCATACAGTGTTCCATTCCAAAAAAATACTGCAGCTTTTAA

TTTTTAGAACTCAGCAGAAATACAAAGGCACGATGTGCTACATGTCGTGCGGCACACCTG

AATCCCTTTTGGACAGGACTTTTGGTATATTTTACATCAAAAGGAGGCTAGGAATGAGGA

GATTCAAGGTTCTACTTCATCCATCCATCCATCTTGTTGTTATATGCTTATAGTTACGTC

ATGTCTTAGAATATGACTATAAGTGACTAAATTATCCGTTCTAGGATT

>XLOC_023452 transcript=TCONS_00038556

ATTTCGTTCTATTCATTGGTTACAGCAAATATAAGGTCAGGTTCAAACCTATTTGCTGGG

TTGTATGGAAATTGTTCGTTTTAAAGGATTAGGGTTTTGGTGGATATAGTTATGGCGGAG

CACAAGGAGATTGTTTTTTTGGTATCATAATTTTCAGATTTCTCCACTGCTTGACAACTT

TTTTTGTTTAATTTGTTATTAACTAGGGGTGAAGAGGGTGATGTTTAATGTTTTCAGCGA

CTTTGCTAATTAGGAATCTGAAGCAGAGTGCATACTCAGATGTGGTGATGATCGGCTGCC

CATGGGTTGATAGAGTTGACCAAATTGAGTAGAGCAAAAAATGCGGATTCATGTTGTTGA

TTGGAATATTATGGTGATATGCTCATTGTTCCTTGCCGCTACATTGAATTAAGATAAGAT

TTATCTGGAATTCAATTTCTAAGATTGTTATGCTAGATCGATCTGGGAAATGGATGAACC

AATGCCGTTGAATCGAAATATACAAAATATGATAAATTTATTTAAATCCAACCTTTACTA

GTTTATATAAATTTGGTAACTTCAAACAGAATAATTGTTTTCAGTCAAACTGTTGTATTC

TTTTTATGATAATCCTTAGATGTTACTTGTTTCTTGAATAAGTTTTTCACATTATGTTCT

TTTTTCATCTTAGATTCTTAGCTTAAGGATATGTCATAGATGTTTCCTGTCAAGTTGCAA

GAATTAAAGAGGCTTTTCCGAAGATGGAAACTTAGATTATTTCAGCTCCTTTCTAGTGCT

CACTTGAGTCTCTTAAAATTACTTTCGTTTGTAGTTAATGAGCATAAGTTCATGAGGCTA

ACACAGAGCTTGCTGGCATTACAGGTTGTCGGTGCTTAACCATAGGACAGTCTTAAAGCA

GCTTTCAGTTTTAATAATATCAAATTGTCTTTCTCTTTAATTGCCCAATAATTATCTCAC

TCTCTTTGTGTTAAAATGTTTAAGGAAGCTTTAGAGTAAGTTAAACTATTTTTGTATAAG

CTTTTCTAAAAAAGAGAAGAAGATTTGTTTAATAGTTATGAACTAAATGATTATCATAAG

TTTCAAACTATTCTTGTAAGACTTTTGCCGAATTAACAACTTGGTTCTATCAATGTTCAG

TAAAGGTTGAATACTTCACCGTGTATTTTTCTTTCAGGTTGTTGGTTTTGCATCCTCATG

AACAAGAAATGCACATTGACAAAATTTAGCTGAAAGGGCATAGCATAGAAATGCACTCTA

TAAGGAAAATTCGGGTGATGCAGATGAAAATGGTGCCCAAAAGTCTATTTGATCCACTTG

AAGCTAATGATGAAGACTTTGGACACTATGATATCTATTATTTGTGATGCTATATGGTTA

CATACGGTTTTACAAAGTTCGTTGCTAGAATGTGCATTTAAACAGTTGGTTTTATAAGTT

GACGATTGATGTACATGAACTATATATTTCATTTGAGG

>XLOC_025129 transcript=TCONS_00041366

TTAGAAGAGAACCCCTCTTTCTTTGACAGCCATTAAAATAACAGATCTCTTTTTTTCTTC

TTGGCCTCTTAAGTCCATGCCACTGAAGCACAAAGCTTCATCCTTGGAATGTCACCCACC

GCCGCAATTGTTGGATGGAGACAGCAAAAGTTCCGATAGCGGTGGTTCTTGGACCAATTC

AGGCATCAAGATTTATCGTTGATATGTGCTTTTGCAAGTAGCTTTCAAGGCGGAGGTCCT

GTGACTTCTTTCCTGAGAGGTAAACTTCCTGCTTATTTTCAACGGATCCTTTTTTGCTCT

GTTTATGAATAGACTTAGATATTATCTTCAACAGAAGCTATGATGCATGTAATTTGCTTG

CAGACCTTCAAACTTATTTACTTTAAGCGAATTTTGCTTGATCCCTTCCCCTTTTTAATG

AAAGCCACCCGCGGCTATTGCCATTTTTATTATGACTTGAATCCATGTCCATAATTTTGG

AGATGAAATTTTGTATCTTTTTCCATATGTTCGCTACTTCTCATCGGAAATTAACACCAC

CTATGCATGTGCTTGTAAAGTTTACATGCTATGGAATATTTAATTGCTCATGTGTGAAAG

AAAAAAAAACATAGCTTTGATGCCTTATGAGAGTACCCACGTAAAAACTAGCGAAACCAG

TTTTCTCTTACGTTTTGTTTTTTGTTTTTTTGACACGCACGTCTTAATCTTTGTGGTGTA

ATTTTCAGCAGGCTTCTTTTCTAGAAAAGGGATTAACAAACCCGATAGTGATTAACAACA

CAATGACTTCCATAGGACTGGTTCTAGGCAGGTAAACCCCTCTCCCTTTCTCGTGTTATA

TTGACTGCTCGTCGTCTTGCTCTTTTCGCAGGTCCGCATATTACTATAATGGCACATGGA

GATATATTCGTTCATGGTGCATGGTGATATACCCTTCAATGGCGTGAAAACATAGGCGCA

GAGACAGGATCTCCTATGATTTGCGTTATTATATTCCTCCACGGAGCGGAGACATAGTCT

TCTATGGCTGAATACAAACTCTTCATTACTGCATGGTGATTTAATCTTCAATGGAGTACG

GTGCTATACTCTTCTATAACACGCGGAGAGGCATACTCTTCTATGACGTACGAAAGACTA

CTCGGAGGATCTCCTTTGCAAATGATCTTTTCAGTTGCCAAGTTTAATTTTCAGATTCAA

CCGTCTTCATACAAAAATACATATATTAGTAGAGGATCGTTGGAATGAGGAGCTTCTTAA

TTCTGTAAAATCATGACTTCAAGAGATACAAATTATTAATAAATCAGATCACGATGCCTT

TAGGATAGACTGCAATTA

>XLOC_009455 transcript=TCONS_00015374

CCATTCTACAAATTATTTACATAATATCCTAAAAAAAGAAATAAAGTTAAAAGATAAAAC

CCGAAAAGTGGCTAGGAGTTGAAGGCATCTTGCACGAATAGAAATGAAGGAGAACCATAA

TGCCTTCTACAAACAGGTGTCAATGCGAGCAACTTGATTTCCAAGGAGAGAGCATTGAGT

TTCTAGGACTCGTTGCAACTCCGGCGTTCCAATGCAAATAAAGAGAAGTGAAGGAATTAG

GAAATGAAGCATTATTCATTCATGAAGAAATAACTAGAACAGCCACTAAGGCAAAGAAAC

TTAAAAAGCTGCAATACCAAGACCGGAGCAGAACTTAAGCAAAAATTATCAATCCCACAA

AAAGAGCCGCAATTAACAATTTTCATTCAAACCGTTTGACTCACACCATAGCAACTAGTA

AAAAAATCACTTCTTTAATTGTAAGCACACGAACAGGAGCATTATACTCATTCAAACATT

GGCTTCCAGGGTAGAATACATTTAACACATAGCCTAGTGTTACTAAAAAGAGAAACTACT

CCTCAACAAATTATGAGAGATATGCATGAGTTCAATACGCAGCAGATAGCTATAAACATT

ACCCTTAAACGCAGCAGATTTTTATAGGTTTTAAGAATACAAATATGAGAAAATATAACC

TTTTAGCCAAAGAAATCATTTGTTATCAACAATTTATTCAAACAGTCATTAAACCTTGTA

CCTATGAACGATCTAAGCAATCAAAATTATACATCCTTATAGATTCACTTCAAGATTACT

CCATCTGTTAAAGGAACTTATAACATGGTGGTTTTTTATGTTAAAGTGCACATCTCGAAC

CCAGATAGTATTCATGCCAAAAGAACAATGAGGTGGAAAATTAGTATATGTTCATAGGAT

ATCTTGAAATTGATCAACACATAATGCCAAAAAGGCCGAAACTTAGTTTTCTTAAAAAGA

AAAGAAACACCAAGAACTGATGCACTTGCTTTATCAAAATGGAAATGAAGCAGACTTAGA

AATTTAGCATACAGCCATATGAATCCTTTAGCCACTAAACGAAACTTCTTATGAAAATAT

AAGCCAACTGTAGCAAACTTATGCAGAATTCTTACAACTCAATTAGGCATAGATTGACAA

ATCCAAATATCCAAGTTATACCAAAGCTTCCAAACAGGGATCCTAAAGAACTTATGCATA

TAAATGAAAATGTACTACTTACTGAAGCTATAGGGAAAGGCCTTCCAGGACATAAAAAAG

ATCTATCACATGAGTTATTTATTTTAGTAGGGTCTTGACTGGATAGACATTAAAATAGTG

ACCTAATTTATTAAAATGAGGATAAGATTTTAAACAAAAACCAATAAAACTTCAGAGAAA

AAAAAATAAAAGATCCAAAGAAACACTTTTGCTACTTCATTCAATGAGACTTTCAAGTTC

CAGAACATGAGTTTAATATACAAAAACACATGCCAAACAAAGAAAGATTTGAGCTTCATT

CAGGCTTAACAAACCAGGAGTTTACTAAGTTTCAAACAATAGGAGAAGAACACAACTTAA

ACTTGAGATTTTAGTCATACCCCAATCCAACCGAAGCTCTAACAAAATAGATTAATTGAA

GTATGTTTTTTCTTAAATAACACCTGAAAATTTCTAGGAATATTAACTGCCATATACACT

CTAAAAAAAAAGGGTTTTTATACTATTTACACAAAAAGGAACGAAAATTTTAAAGTTCAT

AACCAACTGGGATACACTAAATTCAACATTATTTCATAAAGAATTACTATTTCTCATCAA

AAACTATGTCAAGACCGCATTTTATACCTTACGAAGATACTGGCCAGAGAGCTAAAGATG

CAGCCAAGGGAAGTAAATGTAAGAAACACTATGCTTTGTACTCGACATGTCAAAGTTAAG

CTAAGAACCATAACAACAACTACTTTATCTCATAAACATCATTCAGCAGTTTAGTTGGAA

GAAAAGACTCTATCTTTATTACTATAAACTAAAGAACAAACCTTCAAGATTTTTGCTTAA

TAACTACCAAACTAATCATGAAAAAACTAATGACATTAAGTTGCATTTTTCTTACTGCAA

AAAGGAATCATTCATGTAAATTACTTGCTAACTTATAAACACAAAAGAAAAATGAAAAAC

AAAAGTAAAAGAGGAATAAGAGTTCACCTCTTGCAGCGCAGCGACTAGGGCGATGAATCC

GAAAAATAGGCTCGAACAACTGCGAAGACTTGAACGAGGTCGACGACTTGAACAATGAAG

AAGCGACTCTAAAAATGAAGCGATAACACTCCAGAATGCAAAGGAACAGTAAAACTATGC

TTCAAGCATAAACTGGACAAAAGATCTTATTGTTTTGTTCTATATTCTGCTGTTTCTGTT

GTTAATCTCGCTCTGCTGGTCTTATTATTTTCCCCTGTGATTCCTCTCTATTCCTTGGTC

ACGGCTGCCTTTCTCCTCTAAAACTTGGACTCATAGAATCCAACTCGAACATCTTTTTCT

CTCCTCTATATACGGCTCAAAATTTTGGAGACCTCTCTCTTCTTCCTACTACTGCCGCTG

CCCATGAACTCCCTAAATAATCAACAAAAATCCCATTTTATACCTCAACATTAGGGTTTA

AGGGAATGGCCCCATATACCAAAAATGACCCCCAACGGATCTAAAGAGATAAGGTTGGTA

CCATTTAACAGCTGGAATTTTGGATCCTAGGTACAAAGGAAGAGAAATGGGTTTTCTGGT

CCGGTT

>XLOC_013417 transcript=TCONS_00021990

GTTCTCTCAATATTAGCTATGGTTGATCCATTTGAAACCTTACTTATCAGACTCATGGAG

AAGGAATCTGAGTCCGAGTCAGTATACCTTTCAATGAAGCAGGAGGATCCCAACTCGCTT

GCTGTTAACCTTGCAAATTTTCTCAACCAATTAACTCACGACGGCCATGAGTTAATTACA

TACACCAATATCTTACGCCGAATTTTGCTAGATATTTTCAACCATTCTTTCATTGATCTT

GATGTGAAGATTGTGGCCCTAAAAGCCATGGTTGAATTCATTAAATATGTGGGGTGTTCA

ACAAAAGATAGGGAGAAATTTCAGATACTGTCGCCATATATTATGAAAATGCTAACTGAC

GCCTTGATTGACAAAAAATTGGAGGCAGCAGCTCAGTCTCAGTTGGAAATTTTGATTGAT

TTGGCGAAGAATGAGCCTAGATTCTTCAGGACACAGCTTGTAGACGTGATAAATACAATG

TTTGAGATAGTCGAGTGTGAGAGTTATGAAAAGAAAACTAGGCATTTAGCGGTAGCGTTC

ATGTTGACTTTAGCTGAAGCAAAGGAAAAAGCACCAGGAATGATGAACAAGTATCTGTTA

TTTACTTACAAATTTTTTGCAGTTTTACTCAAGTTGTTAGAGGAAGTTGATCATGCCGAC

GATGCTTGGGACACTTTGGATCCGGTTTTTGAGAAAAGGTGTATAAAACGGTTTTCTGAT

GCGGTGGGTGGTAAGACAATGAGTCCTGTTGCACTTGAGCTGCTGCTTGCTTACTTGGAT

GATGATGATGCAGACTCATGGCCGAAGCTATACGCAGCCCTTGTTGCACTTGCTCAGATT

GTTGAAGCCTGCTCTGAGGCTATGATTAAGGATATGAAGGAAATGGTGGCAATGGTTGTG

AATTGTATCGGACATTCTCATCCCGGAGTCAGAGTCGCCGCTTTTCAAGCAATTGAGCAG

CTTTCGGCTGTCTTGAGTCCCAGTATTCAAGAACAATGCCATGACCAACTATTGCCGGCA

TTAACTGGTGCTATGGATGACGACAATGCACGAGTGCAGGACTGTGTCTTCCTCGCCTTG

GAGAAATTTTCTGCGTCCAGCACGGAACTACAGTTGATACCTTACATCGACCTAATACTT

GATA

>XLOC_021417 transcript=TCONS_00035239

GCCTATTCAATATAAAAGTACAATAAAGATACGGTGTCTATGATTGGATGGTAGAACACA

GCCACGCTCACACATAACATTAGTCATCTTTCCTTTTTTGGATTTTCTTGTTTTGTATCC

ACCCACCCCGCCTTTGCCCAATACTACTTATATATACTATTTCCCCTAAATCCCTTATTC

CCAATTGTTCTTTGAACCCATATCTATTCCCCCCTTCTTTTATGCAGATTCCAGGGTCTG

ATTCTTTTGCAAGTTTATCCCTTTTTGGAGTCCTAGATAACAATTTTGTCACATTTTTGA

ATACCCACTTTGCAATTTCATCTAAAGGTCATGACTGCGAAGCGAGTCATAGCCATATGT

CAGTCAGGTGGTGAATTCGAGACTGATAAAGATGGTTTCCTTTCGTATAAAGGCGGAGAT

GCTCATGCTATGGAAATTGATGACAAAATGAACTATAATGATTTCAAGATGGAGGTAGCT

GAGATGTTTAGTTGCAGCCTTGATACCTTGTCCGTTAAGTATTTTCTTCCTGGAAACAGG

AAGACGCTTATAACAATCTCCAATGACAAAGACCTTCAGCGCATGATCAAATTCCATGGT

GACTCTGATACTGCGGAGGTCTATGTGATGACCGAGGAAGCTGTTGATCCTGATTTATCA

AACATGCCTTGCAGTAGGTCAAGCCGAACGACTTTATCAGAAATGGCGGTCCCAGTTGAT

GCTCCTCTGAGTGTTGTGGAGGATATCGTGGAGGACCCTAACGAGACTGGCCTCTTGCTT

GATGCCGCTTTTGATGTTGTAGGTGATACGAACAACGTTGATGACACAATTGAGATAGCA

GCTGAAATGCCCATGCAGGTTTCATTTGCTGCTAATTATGACGAAAAAAATGCTAAAACT

GCTCTGCAGTGGCAGAATGATATAACGGGTGTCGGCCAGAGGTTTAATAGTGTACATGAA

TTTCGTGAGACACTGCGAAAGTATGCTATTGCGAATCAATTTGCTTTTAAGTACAAGAAG

AATGATAGTCATCGAGTGACTGTAAAATGCAAAGCAGAGGGCTGTCCATGGAGAATTCAT

GCATCAAGATTGTCAACTACCCAATTGATATGCATTAAAAAAATGAATCCCACCCACACA

TGTGAAGGGGCTGTTGTTACTAATGGCTATCAGGCAACAAGGAGTTGGGTGGCAAGTATT

ATAAAGGAGAAGTTGAAAATATTCCCGAATTACAAGCCAAAGGACATTGTCACTGACATA

CAAAAGGAATATGGCATCCAGCTGAATTATTTCCAGGCATGGCGTGGGAAAGAGATTGCT

AAGGAGCAGCTTCAAGGTTCATATAAGGAGGCATATAGTCAGCTGCCAATTTTTTGTGAA

AAAGTGATGGAGACAAATCCTGGAAGTCTTGCAACTTTCACTACAAAGGATGACTCAAGC

TTTCATAGGCTCTTTGTGTCATTTCATGCTTCACTATATGGCTTTGAACAAGGCTGCAGA

CCCCTGCTTTTCCTTGATAGTATATTTTTAAAGTCCAAATACCAAGGCACTCTTTTAGCT

GCAACAGCTGCTGATGGGGATGATGGTGTTTTTCCAGTTGCTTTTGCCATAGTAGACTCA

GAATCTGAGGATAACTGGCGGTGGTTCCTATTACAGCTTAGAACTGCATTGTCAATGTGT

CGCGGTATCACTTTCGTAGCAGATAGAGAGAAGGGGCTTAGAGATTCAATTGGTGAAATA

TTTCAGGGCGAGGATGTCTTTCATGGCTACTGTCTACGCTATCTTTCTGAACAACTTATC

AGAGATGTGAGAGGGCAATTTTCGCATGAAGTTAAGCGCCTTTTGGTAGAGGATTTTTAT

GGTGCAGCTTATGCACCAAAGCCTGAAGGCTTCCAAAGGTGTGTTGAAAGCATCAAAAGT

ATTTCACAAGAGGCTTACAACTGGGTAATGCAAAGTGAGCCCATTAATTGGGCAAATGCT

TTCTTCCGTGGGATGCGATATAACCACATGACGTCAAACTTCGGAGAGCTTTTCTATGGT

TGGGTATCAGATGCTCATGACTTGCCAATCACCCAGATGGTTGATGCAATAAGAGGTAAG

ATTATGGAGCTTATTTATACCCGGCGGAGTGAGTGCAATCAATGGGTGACTAGGCTTACA

CCATCTATGGAGGAAAAGCTAGAGAAGGAAAGCCTAAAAATTAGTTCTCTTCAAGCTTAT

ACTTCTACTGGTAGTAGCAAATATGAGGTACGGGGGGATACCACCATAGAATTAGTTGAC

ATCGACCATTTCGACTGTAGTTGCAGAGGTTGGCAGTTAACTGGCTTGCCTTGTTGCCAT

GCCATAGCTGTCATGGGTGGCCTTGGCCGTGATCCCTATGACTATTGCGCCAGGTTCTTT

ACTGCTGATAGTTACAGATCAGTATATTCAGAGTCGATACATCCAATTCCAAGCTTAGAA

AGGCCCAGAAGGAGGGAAGCTTCTCAAGCTGCTGTAACAGTAACCCCTCCTCCCACCCGT

CGACCACCAGGACGGCCGACTACGAAGAAGGTGGGTGCTCATGATGTAGCTAGGCGTCAA

CTCCAATGCAGTAGATGCAAGGGTACAGGCCACAATAAGTCAACTTGCAAAGAGGTGTTA

TTGGAAAGCTAAGGTTTACAGGTCAATAAACAAAAGTGGTCCAGCTCTGGAAGGAAGATT

CCTTTTCTTGATCGTTCTTCCAATCTGTACTTCTAACATAAGTTTCTGGGTAGCTATTTA

GATTTTTGGGAATAATGTAGCAGACTCTTAAACGAGGATACTTGATTCTCTGTTAGTCTG

AAGTGACCCCTCTTTTGTAAAGTAATGTGGTAAATTCTGCATTCAGGTTGTCACCTTGGG

ATGAAAACCGCAACTTCTTGTCTGTGAAAGTGAAAAGTAGCCCTTCAACTGATTATCTTT

CTGTTACTCCACTTGTTGAAGGGCAGATATAAATAAGCGGACCAGAAACATCATCTGGAA

ATTTTGCTGTTCCTTAGGAGAGCAGCCATTGTACTAACCTTCAGGCTATTGGCAATTGTT

TAAGGGCGGATCTTTTCATTTCTTCTCCAGTTTGAGGGAGTGTTAAAGCATTGGCAAAGG

AAGTGATTAGTAAGGTAAGATATGGTGTCCGAAATATCAATAGTGGAGACGATAGACAGC

GACGGGTTAGGGCATATTATTTCTATTTATGACTCGATACTGTCCAGTGTCCACAAACTC

GAAAAGGAAAAACGAATTGCTAACATAATGAAATGGCATAGTCAATGGCATTTTAAACTA

ATATGGACCATTATCATTG

>XLOC_037128 transcript=TCONS_00060858

TTTTAAGGTAAGAAAACATTATCTTTTGTCATACCAAACATCCAAGATATGTGATTACAC

GTTAAGTAAACCGGTCACTCGACATTGCCTTGTCGGAAAATTTTTATTAGTATATGCTTG

TAGATAAAATGCAAAACATGCATAAAGAAGATAAAATGATACCACTTGTTCCAAAGAGCA

AGCTTGTTCGATTGTGAATTGTAGTATCAAGTAAATATGATTTGAATTGTGTTTTAATTT

CTTAAAGATGATGTCATTGTAGTGATTTATTCATTTTTTACTTCTTTAAATATCGACACC

GCTTAGAAATTTTTTTTGGTACGCACCGGTTTGCGAATACCTGTCAAATATGCTATTACT

TTGATATAATTATAATTCATTATTTCAGTCCAGCACTAATTATTAGTCACATCTTGTTGA

TTCTTTACTTGAACCCATCCACAATCAAGTGCCGTAAATTTCACATTTGTGTAGAACCAT

CGTCATTTTTTGCATTTGGCTTGTTATGTGTTCATCCTGGATTTGGTATTATAGTTGATG

GCTGTTTAAGCTACAGCTTCAGCGTGCAAGTAAATTATTAATATTTGTATTTTTTTAGCC

TTAATAGCTTATCATTCTTATTTTTTGTCATACTTGTATCTATCAGGTTTGTTCGAGTTT

GAAAACTGCTTCCTTATTCTGATCTATGCGAATCCTAGACTGACTATGGAGGAGAACCAC

ATACATAAAAGACTTGCTGCTACTAGAGCGGTAGATGTCATTGCAGGAAATGCTGACTTT

TTATCTGAAGTTTTACTCCGTTTGCCTGCAAGATCTCTCATCAGGTTCAGTCTAGTATGC

AAACTATGGCGCAAACTCATTACCAGTACACGATTTAGGCTTAGTCATACTCTTGCATTT

TCTAGTGCCCTCTCCCCTTCTGGGATCTACTTTTATAATTCTTTGACTAATCTCCAAAAA

ATTGAATCCCTTCCGCTGACTGATAATGTAGCGAGCCTCCCTCCTCTTCCATTCCTTGAC

AAAAATATTGTAGAAATAGTAGATACAATCGAGGTTACCCAATCTTGCAATGGACTGTTA

ATGTGCGTGATCACCTCCAAAACTGAAAGACCCTATCTCAAACTTGGTTTTGTTTGCAAC

CCTGCAAAGAAAGGGTATCATCGATTGCCAAGCCCCTATAGGAGTGAGTCATGGAGATTT

TGTTGTCGTTTCTATGCACCCTGTGTTATGTGCTTTGACGGTGGGGTGTTTTGGAATGGT

GCAATTCACTGGGTTGCTGAATACTACTCCATTTACTTTGATGCTAAATCAGAGGTAGTG

GGAAAGAAAGAAATGCCACCAAGCCTTCAGGGACTTTTCTCTAGAAACAAGATTAGGTAT

TTTGGGGAATGGGATGGCCATTTGCATCTTATTCAGGTTCAGAGCGCATATGCCAAGAAA

TTCAATGTGCTTGAAGTAGACAAGGATACCTGGAAGTGGTCAATAAAGTATCGTGTTCAT

CTTGCTCGGTTGACGTCAGCATTTCCTGAGATGGTTCAGCAGACATCATCCGGCACACAA

TATGCATTTTCCATTTTGTCTGTGATTCGAGGAGAAAAGGAAGAAGATTCAATCCTTCTA

CTAACTATTCCCGGGAAGGTGGTCGCCTACAACTTAGTGCGTAAAACTGCTAAACTAGTC

CGCGAGTTGCCTGGTGAAGTAGCTGATACTGATACTATACGCTTTAATCATGTTTGTGCT

TATCGGTATATTGAAAGTTTGGCTCCAGTTCATGAGTGGTCTAATGGTCCAAAATAGTGC

TCTTTTCACTATCTTTCAAGGACTTCTTTTCGATCTTCTGAATTCTCTTCTGGAAAAGGT

TATCTCGATCTTCTGAATTTTCTTCTGGAAAAGGTTATCTCTCATGTATTTCATATGAAG

ACTTCATCAAAGATGCTTGAAAAATATATCCTAGCTTTTGTAGGTTTGTTTTTGGGAATT

CGGTATAAATGTTGTGAACCTTTCTGATTTCAATGTTTTATTTGTAGAAAATGATTGTGT

TAAACTCTTTGGTTATTTTTGTTTTATTATCATTTCATTAGTTTCAGTTCTTTCATATTT

CCAGCCAATGGCTGATTGTATATGTATAATATCATCCTTATCATTATATCACCATCTTAC

TACTCTACTAAAGTAGGCTTCTTAAGGTTAATAGCCTTTTTTTCAGTAAATGCAAATCAC

TTGGTCCTGGAGCTTGCTGAATTTTGAGGTGTAAAGTGGAATCTTTACCCGCAAGGTATC

TTGGACTACCTTTGGGGGAAATATTTGAGGATTGCAGTATATGGAAGGGGGGATGATTGA

AAAACGTGAGAGGTAGCTTAGGACATGGAAGAGGCGGTACCTCTCATTTGGGGGCTGAGC

AACACTGGTTAATAGTGTGTTGGATGGTATGCCGTTAATCCCGTTACCTGCTAGTGTGGA

AATGCAACTGGACAAGATTAGATGCAATTTTATTTTGGAAGGAAATGAAGAAAGTAGGAA

AATACACTTGATAAAATGGGAGAAGTTGTGCTATGCAAAAGGATGGAGGTTTAGGTATCA

GAAACCTCAATCTTCATATTAAAGTCTGCTCTTCAAGTGGTTATGGAGGTGGAGTAATAC

CAGGGAGGACTACTGGAAGGAGGTTATCAAATAAAAAGATAGGCTCCAAGATTATGGATG

CACTATCCAGATTACTTCCTCACATGGCACTGCATTTTTGGAAATTTATTGATAGCCTAT

GGGACGAATTTCAGGGATTTACCTCGCTTAAAATTGGTGATAGTAGGAAAATTCAGTTTT

GGTCAGACAAGTGGCATGGTCTTAAGCTGATACTTTTCCGGATTTGTATAACATGGTAGT

TAATAAAAGCTGTATGAGTGCTGATGTTTGGGATGGTACCTGTAATAATTTGCAATTTAG

AAGAAGTTTCGACTGGAAAATGAGACTTACTAGTTTGTTATGATCATCAGAGGAAATGTG

TTAGAAAGCCGAAAAAGGGATACTGTATTCTGGAAAGGGAGCATGATGGAAAATTCTTGG

TGAAATCTTTTGCTATCAGCTGCTGGTTAGGAGGGTGGGTGGGAATAGTAATGATTGGCC

TTGGAAGATGATGCGGAAGACCAAGGCTCCAAGTAAGGTTGCATGTTTTGGATGGATAGC

AACGGATCTAGGGGTTTCAATTTATGTAGCTGGTGTTACCTGTGTAAAAAGAATGGGGAG

ACAGCGAGTCACCTTTTGTTGCATTGCAAGTATGTATGGCAAATATGGTGGATGTTTTTG

AATCTTTTAGCAATACATTGGGTCATGCCTGCTAATGTTAAGCAAGTATTATGGAGCTGG

CAGGGCCAGACTGTGGGAG

>XLOC_014033 transcript=TCONS_00022983

ATTATATCGCCATCACTATGGATTGTGGGTTTTTTTTTTCCATAGCAGGTCTAAGAACCA

AAATATGAATGCCTTCTATTTGAATCTTTCAGAAAATGTTGATGTGCAGGGAACAAGGAT

TCTAGAAAAATTAAAAGGCTATTCATATTACAACATGGTCATAAGCGGCTATAATGTATA

TGATAGGTTTAATAAATATTAATGAGCAGAATGCATTCTAAAAGCTTATGAAGAGTTCGC

CAGATCTTTGAGAATGCAATCTTCCATATGGTCTTTTCTCCAAATCACTAACTCATGAAT

TCAAAGCAACTCTAACTGCATTCACCTTCTTTCCGCACAGCCTTGTGGGACTGCTTTATG

AATAGATGTATTCCAAAGGGTAGAATGTTATTCTAAAAGACATACTTATTGGCATTAAAA

GAAATAAATATTAAAGTTTTAGCATGAATGCAACTCCAAAATGTATCCATTGAAAGTAAA

GCGGGAT

>XLOC_014940 transcript=TCONS_00024428

CTGAAAGAGAAAAAGAACAAAGCAGAATTATGGAAAGAACTAGAAGAAAATCTGGGTTCG

GAGAATATTGGCAGCTTCAAAGAGAGCATTTCTATGATCGAGTTCAGTTACAAGAATTTA

CCTCTTCCATTGAAGCCTTGTTTCCTTTATTTTGGAGGACTTTCGAAGGGCAGGGATATT

CATGTCTCAAAATTGACCCGACTGTGGGTAGCAGAGGGGTTTGTACAAGCATATATGGGA

AAAGGGCTAGAAGATGCTGCACAAGGTTTCATGGAAGATCTTATTAGTAGAAATTTAGTG

ATGAACGTGGAGAAGAGACCCAATGGCAAGCTCAAAACGTGCCGTATTCATGATCTGTTG

CATAAATTTTGCTTGGAAAAGGCTAAACAAGAGAATTTCCTTCTGCGGATCAATGGATTC

AGTGGAGAGGATACATTTCCTGAAAAGCCTAAGGAATATCGATTATTTGTTCATTCTTCT

GAGGATCAGATTGATATGTGGCA

>XLOC_033128 transcript=TCONS_00054466

GCCCTCCCTCCGTCCCTCACCCCCTTCCCCATCTTCGTCGTCATTTCCTCTCCCTCCCTG

TTGATGTTGTGCTCCAATCTCCGCCATTACTGTGCCGCTGCTACTGCTGTTACGTCGATC

TGGCATCGGAGCACTGTGGAGTTCTAGACCTAGCAGCATAAGGCAGCTCGCCGGAGCTTT

ACATGAGGCAATGCTTTTTCGGCTATGTTATTTTCTTCTTTTTTTTTTTTCTTTTTGAGT

TTCTCCGGTGAATGTGGCAGATTTAAGGCGGTTGTGGCGTATGTTTGGTGATTGTTGAAT

GTAGGGGAATGTATAACATAGGTATACTCTTTGTATGTGTGGTGTATGCTTCAGAGTATT

ATTATTGGAGGCCATTAGTGGCTGAAGGAACGGAGTCTCTTATAATGGTGATTCCCCATC

AAATTCGATACGGCAGGCCTGGGAACTGTGGAAAGATGGTGAGGCCTTTAAGCTCATAGA

TCCAGCAATAGTTGATTCTTGTCCCAGTGAGGAAGCACTACATTGTATTCAAGTAGGACT

ATTGTGTTTGCAAGTCAATGCAGCATACAGACCAACTATGTCATCAATTTTGTTCATGCT

AAGTAATGAAGCAACGGTTCCCTCCTGAAGCAGCCATTGAGTACCCCTAACTTAGACTCA

AAGTACTACATATACAACATCATCTTCGATCAAGGAGGCGACTGTTACAGCACTTGATGT

TCGTTAATTGTCTGATGATCTGTTAATCAAATGTTTCAAGTGGTCGCCACTTCCTAAAGT

ATGAGGCAATATTTTTGATTCATGCACAAGATTATTGTAGGTAATTGGATTGATAAATTG

GCTGATTTCTTACTTTGACAACTGCTCATCAAAGATTAACTTATTAATTTTATTTCAT

>XLOC_031183 transcript=TCONS_00051297

GTTGCTTTGCGGCTGCTCCTCAACATCATCTACTTTTCTAATTCTTTTCTTATTCCAATC

TGGATAATGATGTCATAACATCACAACTGTTCCCTGATATATATCCAGTTGTGGGACTTC

AAAATATTTGTCTTAGCCAGCTTTTTGACCCCACTCCACTCCACGTATTTCCTTTTGTTG

AACCCACTCATTGCCCTTTAAATATTACTTACAAAAAGAGAGAAAACTAAGTACTAAACT

TTTTCAATTCTCTTAATAGTAGGTATGTTTGCCAGAAGCCTTGAAGGCTCAAATTAGCAG

GTTTGAGGAGAAGGATACATGGAAGCGGCTATGGAAGTAGCAGATGATTTGTCTTTTGCA

GAGTTAAGCAAGCAAATCTCCCTTCTAATCATGGATGATGAGGAGGAAGACCCTTCAACT

CACTGTTCTTCAGTTTCTCTTCAGGCCTTTTCACAACGGGTTGATCCGGCAACACCAGCC

CCGTCTATACATGAGCAAAGCTTTAAAAGGGAAAGCAAAGGAACAGGAGTTTTTATCCCT

CGTTCTTCCCATCCAAGAAGAAAGAATAGGCAAGGAAGATATACTTCTTCTAACACCAAG

CTCCAAACGCACACCTATGACCCGAGGGGACTTCTTCACGCCCAATATAATATTGATAAT

CCACCTCATGATTCGTTAAACTCTAGAAAAGCATAACGTAATTAACTAGTGTTAATTTTA

TGAATATATAACCAAATAATCTTTCTCTGTAAATCTTTATGTTTTTGTGTACAAAAGCCA

AGCCTCTTGATCTTGTACTATTAGGGTGGCTCAACTTAAGGCAGTGGGCTTTCTCAGCTC

ATT

>XLOC_022226 transcript=TCONS_00036510

TCAAATCCTGGAAAGACTTCATCAAGCAGCTTCGTCGCGGCTGGAAAACTCATTCTGAAA

GCTGGTGCCATGGTTACTGCTCTTTATTAAGGAAAATTTATGTGTTGCTTTGAGTTATTA

TCAAAGGCTTATATCTATGTTGCATCGGTTGAAGGCCAAATTGGTTTTAGCTCATGTGGT

GGCTCTTGGGGTTTTAACTTGAAGTATAGACCTTCTCGGTGAGGCCTGTTCAGGAAGGCC

GTTGTTTTCTCCAGAAAGGATCATTTATGACCTGTTAGTATCATTAGTTCGCGGTGAGAT

ATCTGGTAATTAAGTAAAAGTGCATCTTATAGCTGTACGACCATTTAGTAACCCTCTTTA

TAATTATTCAATGCTTTTAAAAGTTTTACTAGTAACATCTGGAGTATATCTTTGATTCAT

CATTTTCCATACCTATATCCTCTTTCCCCTATCTGCTATTGGCTCTTGTGACCATTTCGC

TCATTTTGCTAAGCCCCACAAAAGCTTGTATTTTATTTGAAATGAAGATTGTACATGCTG

TCGTAATCAGACATTTATGGAGGATTTGCAACTTCTTCAACCTGGCTTTTAGTGATCCTA

GCAGAAAGAATGGCTTTTTCTGAGCCATTAGTTGTGGCATAAACACCGCGCATATGTGGT

TGCTGGGATTCAAACTCTGTTCTATCAATAGTCTTCATTGTAGTCTCGTCGACCTCTATA

CCACATCGTTGGGTACTAGGAAGGTCATAACATTATGAGAAGTTACAGTCGTCAATTCTA

ATGATTTAATAGTTCATACAGTATTTTGATGCTGCTTCTGCTTCTTCGTCATAGGATGCC

TTCAAAATTTGCAGGTGTCAGCAGCAATATTATTTTAAGCATCACAAAATGAAAGGTAAT

TTGCAGGAAATTTTTCATACCGACTGCTTTACTAATTTTTTAAGAGTAATTTGATGGCGC

TGACTGCCACCGAATCCTTTCCTGACTGCCACACTAAGTGGTGTTCTTCATAAAAATTGG

TGTGCATTGTGGCGTAGAGTGTTGAGAGAAAGTTCATCAATCTATGGTCCATTTGTAGTA

GAATTGCTGCCGCGTTATATCTAAGTTCTTTCTATTGTTGTGCATCAGTATTTATGTAAA

CTTACAGAACTAAAGATACCTTCTTGTCTTTCATCAACCAAATCAACGAATTGTAAGAGG

GACCTGAATTGCAGATTTTTATAGCTTATCAAGGTTTAGTAATAGCTGCATGAATTGAAG

GTCTTTGTCCAGTTGGAAGAATGTAATTGAGCACGGCTATTCATCATTGTATTATCTGTG

ATTTTCCAAATACTGCAGAAGAGCAAGAAAAGTACCAGTGCTATCCGATCTATTATCTTG

CTCCTTGCGGTAGGTTAAACTTGGCTTTGCAAGGGATTACATTTAATTGTGATACCTGTA

CTAGTTTAACCAATTTTGCAGAATAGAGACCAGCTTGTGTGTTGGATTGAGTGGCCAGTG

GCCACCCTTAAGCCTGATCTATCATAATTGTTGTTTCTTCCAATTTGTGCATCACAATAT

ACTTTGTTAATCAAGTCAAGTGGAAGTGAGGGGCTG

>XLOC_029385 transcript=TCONS_00048347

AAAATTGCCACTGAAAACCTATGTGAGACGACAACAAGAGAAGAACTTTGATACAACCAA

GCGTTTTATGAAATTCTCGACCAAAAGGAAGATGTCCAGTAGAGTGCTGTTCAAAGTCAG

TGGAGAAGTTGTGCCAGGAGATCACCTGTAAAATATCGACCCAGAGGTAAAAAGAGTAAA

GTTGCTTGGAGATAAATGTTATTTTAGAACAAATCTCAACATGGCCATTGTATCGTTCCA

GAGGAGGAATTTCTTAGGCATACAGATCATTCGAAAATTGTTGCTATATTATTGCTCAGG

ATACAACTATAGGATTAAAGCATCATAGGAGAAGAGAATTTTACTAGACAAATTGTTTTC

TCTTTTATCTTAAGGTATCAAGCAATTTTATTTTCGAGCTTACAATTTAGTGATTTCTAT

GATGGAAGTTATGTGTCACCTTTTTGGTGCTGCACATTATTTTATATTGGTTTTTGTACA

TGTGTGCATATTTCCCTATCTTTTGGAGGAAAGATCTAATCTGAAGGACAAGAAGTCTGT

AACCCATAAGGACTCTTAATAAAGAAAAGGTCGTTGTACAAAATGGTATTTTGGGGCTTA

CCCCAGACTAGGGAGTTGATTGGTGAAAGATTTCTACTTTTTATTTTCCTATCAGAAATA

CTCTTGACTTGCTTTCTCAGTCAACAGGCATATGCAAAGGAGACCTCGAGTTTCTGGTTT

TGTGGGGTTAATTTGTTTGGAGACCAAAATGAATAATAGAGTGTTATGTTTGTTTGTAAG

ATAGGTGGAAAACTATAGTATAATTAAACTTGAATCTTCAAATAGTTTTCTTGGTTGTTG

AGCTCTACTCTGTCTTTCGTATCACAGATTTTTCGTTGTTGGATTTGTGTTTCCAAGTAA

AGCTGAAATCTTGCACTGTATTATTTTAACCAGTGTAATGTCTATTAACGTGTCGTTTTC

TATCTATGGTTCTTGGAGGATCTTGATTGCGAATTTCATTACATAGTGAAGGATCTTGAC

ACTAACTTTGTTGTTATTTGAATTGGGTCGGGTTTTGAAATGGATTCACGTCCAAAAGTT

GTTGCTGGATCAATGGATGCCTTCAGTAATGAAAGATGGGAGAGGAGCTTTAGAGTGCTG

GCATGATGAATTATGGCGAAACTATTAGCCATTCTAAGCAAGAAGTCAGCCTTGAAATAC

TTGGAATTTTCATTTCCCATTTGAGGCTTTGACCGTACAACTGCCCAATTATATTTCATT

GTTGTTAAAGAAAAGGTGAGTAGCAGTCTGACTAAGCTTCTATGGTGACTATTCGCAAGG

AGTGATGAAGCATAACATTTTATTGTATTTCTATTCTAACAACATTATATGGAACAAATA

ATGTGGCT

>XLOC_015184 transcript=TCONS_00024852

AGCAAATAAACTTTTTTTTGCTATAACTTTTTCCTAGCTAATGTGGTAAAAATCCTAGCC

TTCTCTTGGCTTCTGATGATTTCATAAATTGCACATTTACACAAAGATGACTCCGAGTTT

GCTTTCTGAACTACAATCCAAATTATATGGTTTGACATGTAAAAAAATAAGCAACTGAAA

TCCATTCAAATTCCACCAAAAACATTTATTTGCACAACCAATCAAATGATACAAAGCATC

CTTCGCGGATGAATTACATTTTTACAAGTTAGTTAAAACTTGTCTTAAGCTTAAAAGATC

AACATTGGCTCCATGGTACTCAAGCTCAAAATTATTCACATAAGCTCCCATTTGTTGACA

TCCATGTTTCTTCAAATGTCTCCTTCCTCTACTTCAGTACCATTTTCATCCCTTTGTGTA

GAATTCTCATCCACCTCCTTGATGTGTGCTTCATACATGTATCTGAAAAAAATAATTAGT

TTTTTTCAATAACAAAATCAATCAAATCACAAAGAAGTAAAGAGACTGACTAAGATAGCA

ACAGATTAAAGAGACAAACAAGCAAACTACATGATTTTTTAGGAATCAAGTATAAACATA

GCATAGGTCAATTAGTTTATCAGTGTCAAAAAATTTTAACTTATTGCAACTCCTACGGTG

AATTGATCTCATAATCTCAAGTCCTTGAAATGAGAAACATGAGTGAAATTCAGTTTCGGC

TTTCCAGATAAACGGAAAATAAAGATACAAGATTATCCCATATGTGGCAGAAATGATATT

TGTTACTAGGGAATATTTCTTGACGAATATCAAGTTGTGCTACCATTCAAGAATTCCTAC

TTGCAAGCATAACATAAGGCTATGAAGATCCAATAGAAACACATACTATAACTTAAAGAC

TAGATGGCTTCATAGTACTAACACATATAATAGCTATATGAATATTTGAAGATGAGACAG

AAAGGAACAAGTAATTAATTAAACAGAATACAAGTAGCCAAGTAAAATCATACACTCGAG

ATAAATCTTGTACCGAAAATATTGCAATTACTGGTACAAGGGATTAAATGATGTGCTATC

ACTATGTCAGGTTAAAAACATAGACATGGATATGCTGCAATCAGGAAGAACTCAAATTCT

TCTTACCTGTTAAATTACAGGATCATCCGGGCTTAACAATTCATGTAGTTGTTCTAACAT

AATATTTCTTTGGACTAGTAATCTTGACCTGTTAAAACAACCCCCAACATGACAATAATC

AACCTCTATCAAAATAAATATGATACCAGATAGTATATAATTGAAAAATACTGATAAAAG

CAAGTTGTTGGGACTTAATGATCAAACAAATAGTGCAAGAACTCTGCTTCTTGCATTCAA

CTATGAAGAAAAATCCAATACAAATAAATATTTTCAGTTATTAGTTATTACATACTTCCC

AGTATTGATAAAACAGAAGAACAGTAATAACAAACTCTTGTTAGAATACTTTAATCTGTC

TAAAATGCATACAAATGCTTTAATGCGGTTACATTGTATTATAATTGGGCTCCTCAAAGT

CATTGTATTTTTATCTTTTTAAAGTAAATTCTATACATTGGATATATTAAGGCGTACATA

TTAACTTGGGATACCGCGGTACCGATCTTACTTTAAATTCCACTAACAGTTAAATCAGAT

ATGAATTTGTGAATCTAAGATCTGAACTATGCAAGTAATTAACAATAAAAGAAATAAACA

TTATTTGTACTTGATATTTGAATTAAAATTTCGTTGTTGTGATACCTTCGTATTATCGAG

CTGAGAGACGGCGGCAATTCTGGGAATTGTTGAACCTGTAATGTATAAACCCTAAATCCA

AAGTATAGCCATACCAAAAAACATAAATATATTTGTAAATGAATTAGCTTACCGAAAATG

AGAGACAACATCGTAGCAGTTGCAAGAGAAACACTTTCAGAAGAAAGAAAATAAAGAGAG

GGGGAAATTGAGGATTAGGGTTTAATTACATTTGGAGGGAGATTGTTAAGTGAGAGAGGT

GGTTATAATTGAAAAGGAACAAAACATTTAACTTTTAAAAAATAAAAATGAAAGAGAGGG

AGTTTTAATTTGTTCTCCCTCCAAAAGATTTCGAATAAAATTTTTCAGATTATCAATCCA

ACTTAAATTTCACTACAGACTTAGCTAGAACATTTTCCTAGCTACATTTTAGCTTGACAC

TAATCCTAGCAAAGTCCTAGCTATAATATGTCAACATTTCGAAAAAAAAAAAATTAGTTT

GCTAAGGATTATGAATTTCTAGCTAAATTTATAGCTAACATTACTTATTAGCTAGGGATT

TGATTTTCCTAGCTAAAATCTCTAGCTAATCACATGTTTTCTTGTAGTGTTTGGTTCGGG

TGTCTTTGTAGATGTATAGTGATGTTTCATTTCCACAGTAGTAAGAAACGCTCATGTTTT

TCCTTTTAGGTATATACTTGATGACAGGGAAAAGCCAATCATTACAATGCTAGAATTGAT

TAAAAAAAAAAAAAAAGCTAATGAGGAGATTACACAATAAGAGAACTTGGATCGAAAAAG

TTTAAGCCAGGATTTGCCCAAAGATTGTAAAAAAGCTTGACAAAATTAACAATGATGTTG

TTATGTTTAAGTCTGACTATTCAGGAGGGCCAAAGGTGTTAGTTAGTGGTCCAGGTGGAC

ATATGTGGTGGATATGGTAGCTTGAACATACACTTGTAGGAGATTTGATTTAACTGGATT

GCCTTGTCCTCATGCCTATGTGAGTATTATTGGAAACCCTGAAAATATAGAGGACTATGT

GAATGCATACTATTTTGTTGAGACATTTAAGAATATGTATTCACATTATATCAACCCAAT

AAATCCTGAAAGTCATTGGCCAACTGTCGTAAATGGTGGAGAAGTGATTCCTCCTAAAGT

TGTGAAGAAAAAGAGGGGGAGGAAGCCTAAAAATAGGAGAAAAGAGGCTGAAGAAATAGA

AAAGAAAAAAAGCAGAAGAAGCTACTAAAAGGGCTGAAAAGAGAGCTGAAAAAAGTGTTG

AAAATGAGGATATGCAGAAGTTATCCAGAAAGGGCTTTGTAAGAGGAAGATGTTCAGTTT

GCAAGAAAGATGGTCGTAAAGCAAGAGGTCATAATAAGTGGGCCAAAAAATTTGCTCGTT

CATCTTCAAATCAACCTGAAAATGATGACTTATGGGACCAATCTAATTTTTATCATCCAG

AAATGTGGGCCAATTCTCATGAGTTGAACTTTATAAATCAGTCAACTATAATACAGGGAG

GAACTGAAACTGCTGAATTTGGTACTGTAATACATAATGTAGCTGCTGCTACTCAAGAGT

TTGATGAAATTGAATCTGCTGAAGTAGTTGGGGCAGAAACACCTTCTACTTCATCAAATA

GAGGAAGACCGTTCAAGAGAAAAGTCATCAAGGATTATGTAACAAATGTGCTGGAGAAGA

AGAGTAGAAGCTCTAGAAAAATGTAGGGTGTTTGTGTTTAGTGTCTTTTTTTGGTTTTAT

TAGACTGAAATACTTTATGGTATCCTATGTTGGGTTTTGTTAACATCCTCTCTTGCTAAA

CTTTGAGGGCTTCAAATGTTTCCTAATATCAGTTGTCAAATGAATGGCTATGAACCTGTT

TTTGCTATCAATGTTCAAAGAGCCTTTCATATACAATCTCTTTAACATACTTGAATTATA

TAGGCCTTCATATTGTTG

>XLOC_002393 transcript=TCONS_00003840

GTTATGCACTCCTCTGAATGTCTATACAATGTAAATTTCTCTTACAATTTCAAGATGAAT

GAGTGGCATCAGGCCCACTCTTTTGCTGTACAACGTGTATTGGCCTAGGTATCACATAGA

TAATGGAAATCCTCATTTTGTTGTTCATCAGCAAAGAATGGTGTTGCCACCGACAACTGT

AGCCATATCTAATTTTTGAATGGTTTCCATTGACACCGTTTATTTGAGGATCTTGTCTTT

GTGTAACGGAATGTACTGACAACTAGACTAATCAATGAAAAACAAGAATACACAAGAAGG

AGCTCCTATTTTCGGTATTTTGGTCACAACAAATCAAAGAAAATGAGTTTGAGAAGTCGA

GAGATTATTTAGGTGGTCATGTTGGACTCGTTCAGCTGTAGTGCTTCGTCGATTGAGTTA

TTGTAATATATTATTATCTATGCTTTAGTTGTTATGCTGTATTTGATTGTGATGGGCGCA

CACTTGAAAGGGAGCGTGGTGTGGCCTAAAGGCACACTTGATATGGGGTGTGGTGTTGCC

TAGGATGTGATATTCTAATTTGTGAAAGAGACACACAAGATGTGATATTTTAATTTGTGA

AAGAGGCACACTTGATCGGAGGTGTGATGTGGCGTGGTATGTAACCCAGTTGATGTGAGA

AAATATACTGGTTTGGCCTTTTATAGTTGTATTGGGTGAAATCCATCGTCACCAAAGTTG

ATGACTCGGCATCCACATAGGACGGCACACAAACGACACGTGGCTGCGAATGGATATGAA

GGCAATGGATAAGATCGAAGATAAACAGTTGCAACGGACAGATGAACAGTCACTACGGCC

AGTAAAGTCGCAGCCCATCAATCAAGACAATTCAAAGTAGGAACCTTATCCACAAACAAC

ACAACTGCTCGTCTAAGGAGTTACAACTGCTTGCGAACGGGCACATAAGCTGCCTATATA

TTGTAATTTAAGTCACTAGCATACAATCTAATGCTTCTCTGAAATCTACACTTGTATTCA

AAATATTTACTAGCATTCAATACTTTATCTCC

>XLOC_029169 transcript=TCONS_00047952

TGATCTCCAATAACAGTAAGCTCATGATATGAATATATGCATTTTTGTATTGACTCAAAA

TTATGAACATACATATCTCATTTTCACAAGTTTTAATGACTAAACTATCACTAGTTTTGC

TAAAGCTAAACATGCATTTTTTCTATCATCCAACATAAAGACAAGTATGGAAGGAAAAGA

GTGTAATGACATTATCTGACCCGTCGAAGCTGAAACAGATACAACCACGAGCTCACTGCC

ATTGCCTCGCTGCTATACCCGAGCACGACAAGATAGCCGCATGCCTAGTTCACGGCTGTT

TCGCAGCCATTGTTACAGCATCAAACAGCTGCGTTGCTGCCTCGTCCATGGCTGCCTCGC

TCACAACTCCATCACTGCCTCCCTAAAACTGAAACAGCCCCTCGACATCAAACATCACTA

CCCCGGAAACAGACAACAAAAACAACCACACCAACTGCCATAGCACACGAGAAAAATCAC

AAAATCTCCACCACAGCCCCGTCATTACAACGCCAGCCAAACTGCACCACATCCCAACCT

CCTGCCCTAGAACACTACTACAACAGCCACACCAAACTGCCCACAACACCATCGCACCAC

TGTCCAAACGTCACAGCCCCTCCATCCT

>XLOC_009849 transcript=TCONS_00016052

ATCATAGTTCTTGGTTCTCTTAGCTGGAGGAGTGCCGATTCAGAGGCAAAACTCCTCTGC

TTTTCTCTTCAATTATTCTTTTATAAAGAGTCCATTCCTATTTTGAATTCACAAAAATCG

TGAGAATCAAGTGATGGATTTAGAACCACAAGAGTCTTATATTCTATCTTCATTGAAGGG

CTGCACGAGAATGAAAAGATTGCATAAAGATTGGGTAAGAGCTAAAGGATTTCGAGTAAC

CAAGCAAAGAGTTCAATTAGGACTCAAACTCTTGGAAGCTTTGGAGTTCTAATTGCTCAA

TACTTGTTGGATTTTGAAGCTATAAAACAGGAGCATTTCAGCTGTTGAGGTGTGCAAAAT

ACAGAGAGAATCGAGCTATAGCATTGAAAGAGTCTTCATCTTATTTATGTAATCATTAAG

CTACAGGGAACTAAGTAAAGAACTTGTTCTTTAGATTGAGTCTTTTCAAAGTTCAGTCTT

AGTTCATCTGTAGTAGGTACTATCGAGTTGTATAATTTGGATTCAAGTCTTGTACTTGGA

GAAACTTCACTGATTGTAAAAAAAGTCACTATAGGATTAGAGTTAGTCCTAGGTTAGTAA

GAAAGTAGTCTTGTAACCAAGTGAGTCTTTCAAATAGTGAAGTTTAAATCCTACTTGGAA

GAGTAGGCCGTGGT

>XLOC_034634 transcript=TCONS_00056957

CTTGATGGAGAGGAAGAACCGGTTATCAGCATGGTAAAGGCTAAAATATATGAGAATTTT

GGCCTGGTCTTGATCTGAATCATCTTAGCAAATGGACCAGACCCTTTTAAAATTTTCTAT

GTGTTCATGCATATTCCCTTTCAAATACTACTGCTGCTACATTTCTGTCAAAAGTATTGA

AAAGACTTCAAAATGCTTGCCATGTTATATTGAATTTTCTTAGCCATTAATTTGGGCCTT

GAACTTAATTTGTATGTCAATAAGATTTAATACAGAAGCATGTGATAAATATTAGTTTAC

ACTTGTTCTCTCTTGAGCTAGATATGAATGGGTAGGATAATGCAAGTGTTCTGCAGAATA

ACAATGTCAACTGGGAAAG

>XLOC_016957 transcript=TCONS_00027780

AAATGGTTGAAGCTTCTAAGGCTCAACATGAAGAAATCGTGAATGATTGGCAGTTACTTA

AAAAAAAAAAAAAAACTGCTTCACAGTCTGCTCTCTGTAATTTCAAACGGGCGGACCACC

ACACACAAAGAGCGCCCCGCCTGCACCTAATTTTTTCCCAAAGGTTTTCTTAATTACATG

CCCAGGTAAGTGAAAACACCTCTCTCACACATTCGAACCCCATGGCTCTCTTTCACCGCT

CAAACACCTCCGATCGCCACCATCCACCGTTAGGCCTAGGTAAATTCTCTCTCCTGGTCT

CTCTTCCCCTGCTAATTCCCCCACAAATTCACTGGCTTCCGCTACTTCACATCCACTGTT

CCAAAGTCGATTACCGTCCACCATTCCACCTCCACTGTGTGCCATCACTTTGTACCACCT

CCGACATACAGCAGCCACCATCCTCCGTCCAGAGGCATAGTTCACACGCAAGTCATGTAT

GAAATTACTCTCTTCTACATCTCAATTTTCTTCCCATGTCTTCAATTTCCCAATTACCCG

CCATCCCTGTGCCGCTACTGTATTATCATCACTGTCGCAGCCCCACCAGTCGCGTCATCG

CTCAGTACTGCAGCTCCTATTAGAGCTGAATTGTTATTGATCCTGCTGATACATAAAAGG

TACTTAAATTGGTTCGGGTTATCAAAGTAGATGAGCAACAGACTCGAGCATTACAGGTTA

AGGTGAAAGAGAAGAAGCAGAGAGTTGACATGAGAGAATCAAAAGAAGTGAAAGGGTTGG

GACTTTAAAGTTTCACAAGCAAATGAAAAATGGGACAATAAACTTGAAGAATGAAAGGAG

GAGCCAAAGCAAGACAAATACTCAGAGACACATTTCAGGGAGCCCCTTAAACTTTATTTT

TCATTGTTTAGTTGCCTTGAGGGCAAGGCAACACTTTGAGTTAGGGGGAGGAATTTAATT

GTTTTGACATAATATTATGTCTTAATTGTGAATAATGTT

>XLOC_028772 transcript=TCONS_00047274

TATTCATAAATCAATATACTCCATTTGTTTCTTTTTTCTTTTTGAAAACCCACAATCAAA

AGCCGAAAAGAAAGCCACCGTCAAAAACCCTAAACCCCAACATTAATCGCCTCCCTGGAG

TTCAAGGGATTTCAGAGAATAAGTGTAAGATTCGAAGTCGAATCCATGGAGATTCGAAGT

AGCAGAATCGAAGGAAAACACAAAGCCCTAATAAGAGACCAAACTTGTCCAAAATCGTCT

TGAAGAATACACCCATTTAAGCCGATGATTCGAGTCATTCAGGTCAATGGGTGTCCACCG

GAGATTGAAAACCCTTGTTTACTACTCCGATTTAGGTGCAAGTTATGCTCAATCAGAGAT

GCAGAAGCATATACACCTTCATCATTATGGAGTTTGTAATAGGTGCTTATTATCCATAAA

AAAACACATATGATACTTGAAACTTTTGCTTTATTTGTGATGATTAGTATATGTATTTAT

TACTAGCAAGCATCGTGATTTATTGGGA

>XLOC_009212 transcript=TCONS_00015002

CTTTAACTTAATGGAATATCGGTTACCAGAGCATTATCAATACTGTATGTAAAAAGTTAA

AGGCAGTAGAGTGGCCTGTTTGTGCTTTCATTGGTGTTCACTGTAGACCGACTAAACCTA

GCAAAGAAGCACATGATGTGGTGACTTCTCATCAAGTTTGGGAGAAGACATTGGAAATGG

TTGGCCTTCCTGCTAATGGTGTTCCCATAATTCTTCAAGGAAATGAAATTCATTGCCGCT

ATGTGGCTAACATAGATCAGTAACTAGTTCCAGGTAGATCCATAGTATAATCTATTTATT

TTATGCATATGCTCTTTTGAATGTAGCCACTATGCAGTGTGGCAGAGAAAAGGGAGGGTC

GAACTATATACCTCTAATATGAAAGTTTGCAACTTCACCATAGACCTACAATTTTATAGT

TTACTCAGTTTCTTTTAGATTTATAAATTATAGCAGTGGCGAGATCTAAAGAATATTGGT

GGTTCAGTAC

>XLOC_023248 transcript=TCONS_00038224

TGCAACTTGAACTCTCTCCCTATTAAAAGCTAAAAATAATTTCAGGGAGAAAACAAAATC

CAATTTCATTACTTTTTCTCTACAAAGTTTTCTTTGTCTCTCTGAATTTGAATTTTTTGT

TTGAAGACATAGAGAATCTCTATTAGAGATGGTCAAGTTTATGAATTCATATTAAATGTG

TAAATAGATGGAGAATTGAATTGTTTTCCTCTCCCACCATGGCTCACTATTTTAGAGCTT

CAAATTTCTTCTAAAGCTTCTTCAAACTCCTAAACATTTTTTAGAATTTTTACTTTATCA

AGCTCCAACTGCAGGATAATTTTTTGGGGTTTTCACTTGTTCAAGTCCAAACTCCAGTAT

AATTTTCTGGGATCTTCACTTGTTCAAAGATCAAACGCCAGCATAATTTTCTGGGCTTTT

TACTTGTTTAAGTTCAAACTCCAGTATAATTTCTTTGGCCTTTCACTTATTCAATTCAAA

CTCCAGGATAATATGTTGGGATTTGATGATAAAGACTACAAGAAAATTGAAGTAAGTGTA

GTGCCTTCAAAAATGGCCATATGTGCATAATAACAGTCCAATATTCCAGCCTGTTAGCCC

ATTTTTCTTACCCGACCCATTCATCCCACATAAAGACCCCAAAACTCCCCATTTCTCTCC

CATTCCCTTCCCAACCGCCAAGGCTAGGGAAACTCTGCCCAAAAATCCACCACCCACCGG

AAATCGCCCTCCGCCGCACCATCGTCGCACCACCGCCATTGATCCGCCGCCGGACCACCA

CCTCCGGAGGTCGGAAAAAATACCAAAATACACCATTTGAAAGATCTCGGCCCCCTCTTT

CCATTTCCGCTATTGGTTTCTTCAAAAAGTGAATCGATTCGCGTAGATCGGAGGTCAAAG

TTTCGGTCAAAGCTTGAAATCCGGCCACCTTGATTTTTCTCCGGTAAGTTTTGTTCCCTT

ATCTTCCTCTCCACGCTCTCCTGCACCCTCTTATCCCATTTCCCAAAAAAACCCGTTCCC

CTTGCACCTCCCTGTCTGTTTTCGTTTCTTTCTTTCTTTCTTCTTATTTTTTATTTCTCC

TTTTCTGTTATGGCTGTGATGAGGATGATTTCGAGGCCGTGATGATTAGTTTGTTGGTGG

AAG

>XLOC_032593 transcript=TCONS_00053563

CGGCGATCTTTATAAAGTCGCATCTAAAGCACTGTTGTGGCGACCAAATCGCCATTAGTT

ATTTTTCATATCTCCTCATTACGCACTCATTACTTATTTTTCATATCTCCCATTCTCTCC

AACTCAACTTTTTGAAAGTTGAATACCTGCCTACCTCTTCTCTCCTCATAGGCCCTTTAA

TATTTTGAAGAAAATATAGTCCGAATCGTAACTCCAAAAATAGTCTGCTCCAATCAGATC

TGTATCCTCCCCTTCTTCGATCTTCTATTTGAAATGAGAAAAAATTGAAACCCTACTATC

CTCAGAAAATCCTAGTTTCTGAAGCACAAATTAGTGATGTATCCAGCCTCTAAAGCAATC

AATCAGTCAATATTGGGAGGTCCATTTGATTAGAGTTGAGACATCAGAGAAGATAAGTTG

GGGGTGAACTCTCGGAGAGACATTAGAGAAGATAAGTTGGGGGTGAACTCTTGGGGCAGG

CATTAGAGAAGATAATTGAGACACATGGACAATGTAATGATCATCACAGCAGACAAGTGG

GAGCTTGAAGACACATAATGTTACCTTTTCTGGTCATATGTGTATCTACATAACAACTGA

AAGCTTGAAGGATCATAGTGTTTTATTTTTTGTTTTAATTATGTATATGAATACAATGAC

CATAGATTATAAGTTGTGTAGTATATTATTCGTAATGAAAGAAGCAATAATATCGGCTT

>XLOC_018102 transcript=TCONS_00029635

AGGAAGGAGTCATTAGCAAATCATACCTATTTACTTAAATACCATAATATTTTACTAGTC

ATTTAAATTATTTGCTTAGTTCAATTGCTTATCCGCTTGTTTGAATCCCAATAATAAGTC

CGGTCGGTAACCACATTTGTGGAGCTCGAGAGGTGCCTAACCCCTTCCTTTCGAGTTAAC

TTGAACCTCTTACCTGAATCTTTGACTCGTAGACCAACGCGGAGTTAATTATTTAAATGG

AATTAGAAATTAATAGGTGACCTAATACGTCTAGGAAATTTATTAGGTGGCGACTCCAAA

ATCTTAAGTCAAACGAGTTCACCCAGTCAAATCCGAAACCCGACTCGCCCCGAGTTGAGT

CCAAAATCGGGTATGACAGTAGCCCAGGCCCGCGAATCGGCTAATCCTCCATTTCAAGTT

TCATTTTTGGAAAAAAGTAATTGGAGCTAGCTCTACCACTGAGGAGCACGGGGCCGAAGT

TGATGTCGTTGGCCCCGAGAAGGAATCTATTGGAGAGCCCCGTGAGATTGTCATTCCCGT

CACTGCGGGGGTTAATCTGTTCCAGGATCCGGATGTCGTGATTCATCATACTGTTGACCT

AGTCGGGCTATCTAAGCAACGTTTTCTTTCCCTCTGCAATCAACGGGACCTACTGAATGT

TGCTGCAGGATATTTTCTCATGGACTCATCGCCTGCTAGGAAGCTGTCTGTCGTAGGAAA

GCTGATGAGTGCCTAAAGAATGAGAACGATGCCTTTCGGGGTCAAGTTTCCAGTTTACAG

CCTCTTGTCGCCAAAATCGAGAGGTTGAAGGTGGAGGTCCGAACCACACGGAACTTTCAT

CACCTTTAACAGATGCAGGCTGATAAGCTTGATGCTTCGATGGACACTGTGATCCGGGAA

CATAAAGAAAAGGTGGAAAAAATGACATTGTCCCTTTCAGGAAGCTGGAACTTTGGGGCA

AATACATCGAGCTTGTGTGGTTCTTCCAAAGGCGAATTAGCTAGCTTTGAAGTATAAAAT

GTCTGCGGCGGATGAAAAGGCTAAGGCCACCGAACAGAAGGCTACAGAAGCTCGGAGTCT

CGCCACCTTTATTAAGGACGAGAATGATAAGTGGTGGAATTGTGTTAAATCTTTGGAGAA

GTACCGTGACAAGCTCTCTGCTGATACCCACACGCTGTTTGAGAAGATTGATGCGCAAAG

CGCCAGTCATGTGGCTGTGGTGGACCGCATGAACCTAATTGTCGTGCTTGTTACCCTCCG

GAGGATCCATGATAATCTCAACTTAGATTTGTCTACTGAGATCGAGAAGATGACCAGGCT

TGTAGCGGCAGCTGATAATCAGTTGGGCCCCGACCCAC

>XLOC_011197 transcript=TCONS_00018265

TGGTGTTAAAATTAAAACTCTTAAGAGTGAGGACACATGTAACACAACATATTATTATCC

ACCAGTGGATTATTATACCATATCTCAATATTTCAAAAAGAAGCTACAATCTGCACCTAA

GTATAAGGTAAGGGAGATGAGGATTGATTTGCATAATACCTTTGAACTGAATGCTAGTGA

ACAGAAATGTAAGAGAGCCAAGAAAGTTGCTTTGGAAATTTTAGAAGGGAGTTTCAATGA

TAGGTACAACAAACTAGAGGCATATGCTAATGAGCTAAGGGAGTCAAATCCTGGTAGTGA

TATTGTGATTAACATGTCTAAAGATGCACTGGCAGAGGGTAGAAAAAAATTCTTGAGAAT

GTACATTTTCTTGTATGCCTTGAAAATGGGGCTCAAGGAAGGGTTAAGGCCCTTTATTGG

GCTGGATGAGACATTTCTTAAGGGATAAGCTAAGGGGCAGCTGCTGGTGGTTGTTAGGCA

GGGCAACATAAACCATTTCTATCCCATTGCATGGGCAATTGTAGACAGAGAGACCAAGAC

TACATGGACCTGGTTCCTGGGGTTACTGAGGCATTCATTGGAGCTGAATGATGGTACATG

GATTACCTTCATTTGAGACATGCAGAAGGGTTTATGGTTGATAATAAAGGATGTTCTGCC

AGAGGCACACCACAGATTTTGTGTTAGACAACATTGAATCTAATTTCCTGAAGAGGTGGC

CTTCTAAAGAAATGAAGAAGTACTTATGGTGGTGTGGTTGGAGCTCTTATGAGGAGGATT

TTCATGACCAGTTGGACAAGATTGGTAAGCTGGATGAAGCTGCTGCTGTTGATTTGGCAA

GTAAAGGCAAACAACCTGCCAAGGGAAAGGGCAAGAAGAGCAAGACAAATCATGTTGAGG

AAGAGATAGAAGTTGAATCATATGCACCAGACATGAGCTTCACTACATCTCAAAGCACTC

AACAAACCAGCTTTGTTTTTATCCCTACACCAACATTTCAAAGCCAATTTGGAAGCAGCA

TCCCAACTTTCCAAAGTCATTCCTCAAGCAGCAACAATCCAGGCTTTGAATTACCAGAAT

TTCACATGAAGAGGAAGAGGATCCTGAGCTACAACCCAAAGTGATTTCTGAAACATTTAC

TAGGCTACAAAGGAGGCAGCAAGTTGATGTGCCAACTGGAAGGAAAAAAAATTGGCTTAA

GAGGTGACAACACTAGAGCAAGTGTTCCATCAAATCTGCCTTACAAACCAAGAAGCTTAA

CATTGATGGGAAAAGATGCCGTCAGTACAAACCAGTTGGAAAAACAAAGTCAAGCAAGGA

TTGAGAAGTTGAAGGCAAG

>XLOC_033329 transcript=TCONS_00054810

AAAGAAAAAATGAAAGAAAGAAACAGGAGAAACAACCTATGGGAAGTAACAAATGCAACT

GATGGTAGAAATCGAAAAGCAACCTGAAATGAAGAAATTGAAAATTAACCTTCACCCAGC

AAGATACAGCTCATCCAAAAGGCAGTCTCCGTATCTCTGGAGCAAGCACTTGTCCGAAGC

AGAAAAAATCAAGTCCTATTTCTTGAGATTTGTTTTGCCCCAACGCTCAGTTTTATGAGC

GGTGTTTCGACTAATTGGACTACTTGGGAGATGGCAGAAATAGAGGCTATCCAGGAGAAG

CAGCTGGATGAATCACACCTTAAAAGACCAAATAATTACAGGTAATTAGATACTGTTGAA

AAGTTTTTTGAGCGCGTTATTTTGACTATTCTATGTATTTTTCTATTTTGGATTCTAATA

TGACACGTCTGCTCATGCTGATTTATGTATGACTAACCTTTGAATGAGTACTATATTTTT

TAATTTGTTGCTCTGAATCCAGCTTTATAAGTGTTCTAATGCTATTTGGCACAAGCTCGT

CCACGTTATGGGCACACACTATGTCATGTTTATGGAACAACCATGAAGCCACAAGTGAAA

GGGACGACATTACAATTCCTGCTGAAGATTTAATATGTCAATTTATTAGTCATTCTGGAC

GTTAGATGAAAATCTTTTTTATTTCTTATGAACTCAGCTGATAGAGAGACATCAATAGGA

ACTATTTATTTATTCAATTTTGCTGGTAAACCCTTGATTTCTTGTCGTTTGAAAATTCTG

ATTCATTTTCTTGGAACATAATGGTTTTTTAGGGGACAAGTTGCTCAAAGTGGGGGAAAA

AAGGGGACTACATCGATAGTAAGAGCAATGTTTCTAAGGTGTGGCACTTTATATGCAGTG

AAGTGAGAGTTGGAAAGGTTGCTTCAAAACCGTGACATCACAAGGTTGGGAATAAACTTG

AAATAGAATGATAAACTCTTTGACCCTGTTAAAGTGAACAAACTAGAGAAAACATCCATC

TAACTCCAAAGAAGAAATTAACGTAATTCCAAGGACATTAAGATTCGGGCAGGGAGAGAA

GTTATTTTATTTTAGTACTTTTCTTGACAAACTTCATTAATTTTAATTTTTGTTCGAGAA

GGCCCAAATGAATGTGTATTAAAGATACCTTTGTTCATATTCATTGTCTCAAAAAGAATT

ATCGCAACGTGAAATTTGATATAGCTCGAATATTTGAAATAGCTTATGCGACTTTTAATG

TTAGATATCAAAAACACTTGGGTTATCTAGTATGA

>XLOC_010080 transcript=TCONS_00016447

GTTCAACCTTATAGAAAATTTTGTACATATTTTTTATCTTCCTTCTCCCTTGGACGAAAT

CCAATTCTAAAAACAAATGCATCTCTCTCTTTCACTTCTTTTCGAGGCCCATGCGAATCC

ACCAACCACTGACCACCGTCCTAAAGCTCAGTGGATAGGGACTCTCAGGCACCAAATACA

ATGACACCGATGATCTTAGAGATGGAAATTGCTTCAAACTTGCCAAATCGTGTGCATGAT

TCTACCCGTAAAGAAAGGGCTTTTCTCAAATTTTCATCAAAGTTAAATAGAGATGTCAGA

TTACAATAAAGGCACATGGATTGTTCTAAAAGGTGAATCTCAACGCTTTTATCATTATTT

AATGGCTATTGGATTTGCTCCTTTTATTTATTGACATCGTATTCCTATTCTGTAAATGCC

CGGCCATAGATTGTGAAGTACTCCAAAACCATGTGTAGTGAAAGTATTTGTTATTCTATT

CTTAACATGAATCTTGATTACTAAGTCTCTTCTTTTAAATTTCAAATATCTTAAACATAG

TTCAAATGTAAAATGATTGTTTCTCCTATATTTTCATTTTTACTTGATATTGTGGGATCA

TCAAAATGATCATATGCATTTGTTTTCTTAAAATAATTGGGAAAAGGCTCTTCAACGAGA

AAATTGATGTTTCAAGGAGA

>XLOC_037234 transcript=TCONS_00060993

TTCTTATCTACGTTTCTAACCATACAACGTAAAAGGAACAAAAAACAAGAAGTATAGAAC

AAATTATCCAAAAATCAAATTGCAGCAACCTTTCAATATGGCTGAAGCTGTAATGAGCTT

TACAGTCATCAAACCAACAATTCACTGCCCTTCCATCAAAACTATTCAATTGGATTTGCA

CCCTTTCACAAAATCACTTAGACATTGTACAAGTTCAGCTTTTGGAAATGGCAGGCACCA

GGTAAGTTCCATTTTATCCTACCATACTAATTCATAAGAAAGACGAATCCAGAATTTAAA

GATTGTTTGTGCACCATTTAATATAAGTATATACGTTAATTCATTTTGAATTTCTATATC

CATACATATACATCATCGGTCGTTTCATTTTTTGCGTCTTAAGACTCTTAATTTGAATAA

CGCCAAGTTAAAGAACGTAAATAATACATTTGAATGTTTGTTTATTAAACTAAATGTGCG

TATAATATATCATAAATTCCCAATTGAATATCCTTGTGATCTTAAACATGTATAGAAATG

TGGGTTTTTATTAGCAGACTAATAAGAAAAGTAAGTCACATAAATTGGAACTGATGAATA

CATAACACAAGCTCAAGTTCGAAGCATTATTTAATATTTATACTTCATTTTTTT

>XLOC_033007 transcript=TCONS_00054262

CATTTATTTGTTTGCTGTAGACTTGTAGAAATGGGGTGGCCGAGACGCTTCAAGAGCATA

CTAGACGAGGTTCATTCCAAGACACAAAATCACATGGGAGCTAGATCTGCACCATTAGCT

CATAGTAGAACTTCTTCTACACCGCCAGTACGTGATGGAGCTACATTTATACAATCAACT

CATGTTGAAGATAGCTTTGTACAATCTTCTCATCATGGAACAACTTCTGCTCAATCAAAT

CATAATGGGGCTCACTCTGAAGCTGAGAATACCAATTTTTTTATGAATTTGAAGATGAAA

TTACTCTTACATCTGGTAAGACAATTAATAACCATCGCAAAACTGATGGCTATTGGAAGG

TTAGTCTTATAGATGACAATGGTACCATCACACAAGAGAGATGAAGGGTACATGATATTT

GGACATTGCTGATAAGTATAAAGGTTATAGTTCCTGGCAACGAGTTTGGTAAATATATTG

AGGATGATGGTGGGCTTTTGGGTGGTTTCATAGGAGTTTTGGCATCGGATTTTTTTAAGC

TGCCTATGTGTTATGTCTTGTCGCTTAAGGTTCCAAAGAAATACAAGGATAGTGCATCAT

TTTGATTCCATGTATTGCAATCACTGAACTTCTTAGATCTTTTTCCTTTTAGGTAATTTT

TCTATTTCTTGTTTTCGCTCTTGAGTTTTGAAAACACCTGATGCTATCATATAGTTCTAA

AAAACTCAAAAATATTTTGGTTAAGTTCTTGTTGTTCTGGTGGGAGCAATACTGTTTTTA

TTACTGAGTATTCATCATGAATTTGATGTCTACTGAATGTCTGGTTTCAGCCAAAGGAAA

AGACTAACATGGATAAGTTACTGAAGATAAGTCTTATGCTTGATATTATTTGGAGGTGTG

GTTTTGAAATCTGCCAAGCTTATGAATGTTTCTCTTAATACCAAAGAATAGTTTCTTTTT

GGTTTTTCTTTCAATATTCAAGAGAAAAACATGGATTCCAACAAAGGAGCTGATGATAGG

AAAACAGTACAGAATTATGTCGCGACTGGGTTTTGCACAATTTGTTTCTTCATATTCAAT

GTTTCTCAGCAAGTTGATTATTTGTGTTTTTAAGAATATTTATTAACAGTTTGATCTTTT

GATTTCAGACTTCAGATGAAGAAAGCGGGCCATTTTCGGCAGGTGTCAGACCGTTGTCTT

CAGCTAGTCGTATTGTTTGAAGTTAAATTTCTCATTTAGAAAAATGATTCGAATGCTGTC

ATTGAGAAAATATATTTTGATGGATTTTTATTTAACTTTATTATGAATGTAAATTATGGG

TTAACATAATTTTATTACGTTATGTTATGTATGATTACCTTTGCGATTGGAA

>XLOC_022879 transcript=TCONS_00037618

AACAAACTAAGCTACTAGTATCTTAAGCAAGAGACAGAAGAGAAAAAGAAAGAGAAGATG

GCAGATAAGGAAGGCAAAGGAAAGGGAAAAGCAAAGATGGCAAGCAGTGGAGACAGTAAC

AATGGTATATTCCCCTTCACTCCAAAGAAGGGAAGTGTACTTCCTAAAGAGAAGAAGCAT

GTTTCCACCATGATGGGTGAGAAAATTGGTCAGTCTATTGGTTCCCTTGTCAAGAACAAG

AACAAGAAGATCAACCCCAGCAGTGATCGTGATTGATACTTAACTAGACATGTCAGAACA

GAGCCAAAGTAGTTCCAACTCCACTTGTAAACCTTGTCTTAATTTATTCTACAATCATTT

CTTGTACTTAGATGTGTAGGGGGTCACCAATGCATCACAATTGTTTTGTTTTGTGCTTTG

TGCTGTTGTATTTTTGAAATGCTTATCAGATTGTATTATGTTGGGAGCTCATTTTTCTTA

AAAAGAAAAAAAAAAAAAACTTCACCT

>XLOC_022659 transcript=TCONS_00037240

GGCACATCAGTTGCAAATGGTTGATATTCAATTCTCCACTCCAATTCTTGCGTTGAACTC

TACATTTCTTGTGTAGCCCTTAGCTATTCCCTTATCCATTGTACACTCGATCCCCTCAAC

ATTAGCTAACCTCTTCTGCACTTGAGATAAAAACACTTTGAAACTATCCACTTCCAAATT

GTTTTTCCATTACTTATCCATTAATTTTTCATATGTTTCTGTCCTGCTCCTTTATTTAGA

AAAGGTGAGAGGGAAAAAGTCGTTGTTGATCTAGTCATGGATTTCGCAAAGGATATTGAA

AAACAAAAAACAGTTATGGGGTATCCTTCAATGACTAGACACAACGATATTTCTTATCAA

CAATCTATCCCTTCAACATATGACCAAAATCCTCCAAGTTATATAATGCCAACCCAAGGC

TATCCAAATTACCCTAGTAACCCCAATCCATACCCTTACCCCGATGCTACATACTATGTC

AATTATTCCCAAAAGTACATACCATTGCAAGAACAAAATAAAATAAAAAATGGGTCATTT

GGTCGTTATATGTCTACCATGTTGCTAGTTTTGGTCGTTGGTATGATCATGTTTAGCTTG

GTAATTTGGCTTTTATTTGGCACAGAAGTTCCCGACTTTCATGTAGTATCGGTACAAGTT

CCTACTTTTATGATTACTAGCACATCATTATTAGGTAATTGGCAGGTCAATGTCACGATG

AAAAACTCGAATAATGACTTAGATATAAAGGTGAATAACGGAAGGACTTCCATTTTTTAC

GATACGAACCTTTTGGCTGAGACTCCAGTTGATCCTTTCAATATAGCGGCAGAAAGTTCG

GTTATTTTATTTTCTAATTTGACAACATCACCTGGACATTTCTTGGATAAAGGAGTTTTA

TCAGATGTTACAAGTGATAGAAATCATGGAGTTGTAAAATTTGCCCTGCAGCTCTCCTTA

GGGATTCAATATACTTCGAAATCAGAATCAAAAAGTCAGAGAATACGAGTTTACTGCAAT

GATGTGAAAGTTCAATTTGGTCATGAACCTCAAGATAAAGGAGAGTTGATAAAAGGTGAT

AACATTGATTGTCTAATAAATTCATAAAATTTGGGGATGGAGTTGCTTTCAGGCAGCTCA

ATGAAATTGGCTGGAAACTTGGAATGGAGTCATGGACTGAAGCTGACATTCAAGACATGC

ATCTGTATCTTTCTTTTTTGCTGTACAGAAACCTCATTTTTAATTGGTACCTATTGACTT

TGGAAAAGTGTAATTATATATAGTGCTAATTATAGGCAGGTGCGAGAGTTATTTTATTTG

ACCCCACCTGCTTTCTTCTATAGTAGATCGGGAGTATCCAACTCACTACTCACTA

>XLOC_000953 transcript=TCONS_00001535

ATGAAATTGTAGAACTATGTTATGACAAATATTTTAGAAGGAGCAGAGCTGATTTTTACC

ATGCAGTTTGCAAAACTGTCGAAGAAATTAATAAAAGGCAAGGATTTACACAATTTAAGA

TTCCAAGCACTGCCTCTCTAAAGAGAGCATTCTATAAGCATCACAGAGGCAAAGATATAT

TGACAAAGGATGAATTCAAAAGGATCCTGAATGATATAATTCATGAATCAGGAATCACTG

GCTTTGGAGCAAAAGATACATTCTTATATTTATTTGGGGTTCCTGCAACTGCATTATTTA

TCAAGCAAAGAATAAAGCCACAATCAATTTCTAATGATAAATTTATTCCTGGTGTTACTT

CTGCCACTGTATTTGTTCTTGCCCAAT

>XLOC_033172 transcript=TCONS_00054539

TATTGATGAGATTCAAGATATAAAGAGTGGATGTATGTTTTCTTTCTCTCATACACTTAG

AGAGAGAATTATTTGGCTAATTAAATGGCTAATTTGGTGTATGAAGAGTATGAAATTAGT

TTCTCTTCCTTTAACCAACTCCCATCAAAAAGTAAGAGGATTGTCAATTTGGACAAGTAT

GGATGTCCGTTTGTATGGGCTCACTGTGTACTATAATTGATGGTAGCTGGAAGCACTATA

GATTGGGTTAACGGGAGCGCACTGTGCGAAGATTGGGTGACTAGTCCTGGAGGAAAGGAG

CTATGTTGGAGCCTTCATTTTCAAATCCTTAGGGAGGAGAACATGAGGGAATATGTACAA

AGGAAGATACATCAAGGACTATGTGTCGATGCAAACAAAACTTGTTACAAGCAGAAAGTC

CATTGGCAAGTCAGTCGCTACAGCGCAGGCTCCCTATGGAAGATCTCATAGAGGACCTGG

ACCAAGTTTGACAGATTTTCAGAAAAACTATAGCGTGGGCGATGCCTCCAAAACACTTAG

ATTAAATAAAATGATCGAAGAATGGCAGGCATTTGGCATGCAACTTCTTTGCCATCGCCA

CCAGCTTACGCTAATGGTAGCGACTATTTGGATCAATTCTTGTCATGTCAAGATGAAATG

AAAAAATCAATGGAGCTAATAATTTTTGCATTCTTTGATTTAGGAAATCGTCATAATTTA

AAAAGGCAACTTGCCATGTTCAACAAGAAAGAGAACCAATTGCAAGCAAGGAAGAAACTT

ATTTGGAAGGCTACTTGGATTGTCTTTCATGCAGTTTTGTTATTACCGTGGTGTCGATTA

GTC

>XLOC_023440 transcript=TCONS_00038533

ATCGGTTACTCTCAATTCTCTCTCTACTTTTTCCTAACCCAATTCCTTTTTGTCTTTTCG

TTATTTATATTCAGTAAGAAGAATTCAATAATATTCGTTACAATTTCTATTTTCAATCCA

AGAGTATTACAAGTGGTATCAAGAGCCATTCCAGGCACCTACAATAATGGTGGAATCACG

AGCTAAGAGTACTGAAGAGCATATTGCTCGAGTAAAAACTCAATTACAACATCATATTTC

AACTGCGAATCAGAAACTTGAAGAAAACAACTCAAAAATTGATGCACTGAGCAATAAGAT

AGAAGAAATTTTAGGGAAGTTGCTGAATCAACAAAAACCAGTGGAGGAATCTTCAAATGG

AGTAGTGCACAAGCATAGACTTTTAGAAATCCAAGAAGTTACTCCGGCTCATTCACAGGA

ATGTTATCATGATAGAGATCGGTGGGAAGACGAGTGGGAGGAAGCACCGACGGAACTTCT

TCAGGGACAGTTTCAGTTGCGGCAGTTGTAATGAGGCTTGATTTGCTGGTAGCACAATCG

CCCACGACGACCACGGGGATGTTAGTATGGCCGCCGAAGATTGAACCGCCGAGCATTTGA

AATTGGTGATGATGATGATTGAATACAAACGTGAAAAAGAATGTGGAGATAGTAATATAT

ATGGTGGAGTTGACACACAATAAAAAAAAAGGGAATCGAATCTAAATGTAATAGTATTAC

TACTATTTGATTTTCTTATTTGGTTCACACTTTTCTGTTGTTTTCACCTGTTTGTTGAAA

G

>XLOC_017636 transcript=TCONS_00028872

CTCTATTTCAATGTTTATCCACTTGTTTCAAAGGTCTAGAACAAACTGCTCTACACTAAA

TAAGGTTCTTTTATCTTATGTATATAGGAGCAAATAGAGAATATCAAGCGCCAAAATAGC

CCATCGATTACAATTTCACCAGATGATACAGTTGGTCATGTACTAAAGAAGGATCATCCT

GGTCAAGTTATGTGTGTGGGCCAGGGCCCAACTCCAACTCAAATATTTGGCCACGCTACT

CACCAACGAAGTGGAACTTCTTTTACTCAACCGATTCAAGATAACAAAATGGATGAGATA

AAGTCGGAGTTGGAAGCTGAAAAATCTAAGATGTGAGAGCTTGAGTCGGTGGTGGATGCT

GAAAAGTTGAAGAGGCGAAAGCTTGGTGGGTTTCATATCAAAATGTTTCATAAATCAAGG

AGGAGATATGCTGCCTGAGTTAGCTGCTATAGTTGATGCAATGGTATGGCTTTCAACTTT

TCTTGCTCCCTAATCTCCCTTCTTTTCCTTATTTTTCGGATAAGGCTCCCTGATATTCCT

TTACTTGAGCTGAACTTCTGTTTTCTGCAAAGGAGGTCTGTTCACAATCTTTTAAGTGCA

AATATAATGAATATATGTTTCCAAATGAAGTATTTGTAGATTGTTGGTCTGCAATTTAAT

CAATGGTTTCACATAGCCGTCAAGTAACCTGAATCGCGTTGATGTTTGTTCTTTGCTGAA

TCAAATCTTGTTCACTGAATCCTGTCGATTTGGTCTTTGCTGAATCAAATCTTGTTCACT

GAATGCTGTTGATGTTTGTTCAGAGCCTATGCCATTTAACTATATATACTATGTCCTGCT

ATTTATAGTATTTAAGATAAAGATTCAACCTTTATAGTATTTATTATATACTATGTCCTG

CTATTTCTGTGGTTGTGATTTCCTTCGTAGGTTATGATCAAGATAGTTCTGTCTTCTGGA

CATCTTTGAACCTGTCAATGTAGCAAAATTCACTTTAAGTCTCGCAGGACTTCATGGAAA

TAAAAAAGATCACTTCCAGTCCATAGCATTTGCTTTTAATAATATTTTTGCTTGTCCTTA

ACAAAATAAATTAAATAGCTTTAATCATAAGAGTATTTTGTCTAAAGTCTCATTAAGAGT

CACTTGACTAACACTACATGAAAACAGTAAAGGTAAACCTAGAAAAACAATAATAATTGA

CCTTTTTAAAAAAAAATCAAATGATTTAGTTTGCCAAAATAACTAATTATGTTTAGGACA

AGTGTCGTACCAAACTGCAAAAGAAAAGGAAAGAATTCTTCGTATTATTTGATGTAATAT

ATTGTATATACCATCTCACGATCAATAAAAAAAATGTAAAAATCTGATAGTACTACCTAC

ACAGGATTCTTTCTTCGTTGGCTATAGGAGCTAGAAATCTTAGTGCTCTTTCCCTCTGTG

TTGTGTCTGCAATTTTTTGTGGCTTTGCTAATCCTGCTTTGTGGTCCAGTCTGTTAGCAT

GTTGTCTAATTTCTGCTTGGCTCCCTGATTGTTGAAATATAAGGCTGACTAGTTGGTTTT

CTTCCGGAAAGAAAAATCAATCCTGCAACCTCAGGTAGGGCCTTTCTTTTCTGCATTTAT

CCATAAGCTTTTGCTTTCTAGTGTATGCATAAGCTTTTGCTTTCTAGTGTCAGTAGAGTC

GGCTTCCTTGTTTCCTCTTAATTTTTCTGGATTGAATTGCATGGTTCGGTCTACATGCAT

ATGGATTCTGGCAATTTTGTTGTCTTCTGGTTCAAGTGTTGTTTTCTTTGCCATCTGATC

AGTCAGTTTTAGGCTTCGGATGAAGAAAGTGAGCGGATTTCTCCAGATGTCAGACCTTCA

TCTTCAACTAGTCATAATGCTGCAAGTTAATTTCTCATCGAGAAAAATCTAACTTGTCTT

TGAGAAGATATTAATGTGTTTGTAATGAATTTTAGTTCAACTTTGTTTTAGATGCCGGAT

ATTAATGAGTTATGTTATTTAAGATTTGCCTTTTCAATTGAAAGCTGGAGTGTTTTGTAA

TTCCATTTGAGTTATTTTAATAGTATTGTACAAAATTGAATAATTTAATTTAGG

>XLOC_021165 transcript=TCONS_00034781

CAGGCCCGTTGCAGATAAAAAACCCAACCAGCTATGTCTCCCACATCGACCATACAGAGA

CGCTTTCAACAGTTTATATAGTGAAAACACAACAGTAGAGTTCGTCCCTTCGGGGACATC

CGATAAAATTGGAACGATACAGAGAAGATTAGCATGGCCCCTGCGCAAGGATGACACGCA

CAAATCGAGAAATGGTCCAAATTTTTTTTGCCATCTTTGCACATCCATATAATGCTCTTA

ATCTGGATTTCTCCGTTTCTATTGTTCTCTTTTTTCCTTCTTGGGCTTTGCTCTATCAAG

TTTAAACCTTCAAGCTCAGGTTGCTGCTGCCTAAAGCCGGGATCCTCTGCTGCATCACAG

TACAGGGAACTGGGAAGCAAATGGAGTTGCTGATCAACTGGCTAAATATGGATGCAGTTT

GGACTTTTTTGAAAGCTCAGTGACTTATTCTATTCCCCCGTATTACGTAGTTTATGCTTT

TGAACATGACAATCCGAGTGCCATGTATCCCAGATTAGTATGTAATTGTATTAGTATATG

TAGTTTTTAAAAAAAAGTAACTTCATTACCAAAAAAGAAGAAAAAGACGCTAATTGGCTT

ATCTTTCAGGGGAATTATAATGATTCCCTAGCTGAGTTGCTCTGTATGGCTGATTCTTAC

AATACAAAGAGCTTTTATGTCACTAT

>XLOC_030091 transcript=TCONS_00049531

CACGATTACAAAAAGAAGGCAAAAAATAAAGGTCGTGTCGAGGGATCAATATGTGAAGCT

TATATCATTCAGGAAATTCCACATTTTGTTTTCATTATTTCGAGCCTAACGTTCCAACTA

GGCTGAATAAAGTTACCCGAAATGATGATGGAGATGAAGTTGATGCACCTGATGGTTGTT

TGTCCATCTTCTTGCATCCGAGACGTTCAAATGGGGAGACACACACTCGATATTTGTCAG

ATGAGGAATTTGATGCGGCACGGTTGTATGTTTTATTAAACTGTGAGGAGGTTCAACCAT

ATGTCCTTAACTTTGAAGCTGAATTAAGAAGAAATTCAGCAAATATTAGCCTCGAACAAA

TTGATAGGGAGACAACTAGTAGATTTGCCAAGTGGTTTGAAGCTTATGTTCTTAATCCAG

CCGACAGTTGATGAGCGACTGATAAACTTAGCCTGTGGTCCTTATAAGAAAGTGCAAACA

TGGCCGCAATATTTTACAAATGGGTATAGGTTCGACACTATTACTCATGGCTCTAACAAA

TCAACTATGAATAGTGGTGTATGCATCAAAGAAAAAAGTTGGAGTGACTATGAAAGTGAC

TACTATGGATTGCTCGTTGATGTGATAGAACTAGAGTATTCTAATCCAACGGATAAGAGA

ACTACTCTCGTGTTGTTCAAATGCGATTGGTTTGATCCAACAATGAACTGAGGTATGAAG

GTTCATAATCGATATGGTCTAATTGATATCAATCATAAGAAAAGTTTCTTGAGCTATACA

TATGAGCCGTTTGTGCTGGCTGAACAAGCACAACAAGTTTATTTTGCGGAATATTCTAGT

AAAAAGAATGATAAGGCTGATTGGTGGGCAGTATGTAAAGTAAAAGCTAGAAGCCGAATT

GATTCCCCAAATATACCTTTCCAAGAAGACGAGGGTTATCCTGCTTCACTCCCAAATGAA

ATTGATGATCTTAATTGTTTACTTCATGAAGATAATGAAGTGGAAGAACATGCCATTACT

GAAGACTCTGCTGAAATTGGAAGAGCGCATAACAGTGATGCCGTTGAAAGTGAGAAAGAG

ATGGAAGAATCTGATTTTGAATCCTCCAAAGATGAAGGAGATGAATATAGTGAAGATGAT

GACAATGAATAATCCTCTGTCTATTGACTTTTTTTTATGCTTTCTCTTTGTCGTGTTACT

TATATATGTATGCTTATTGGTGCATATTATTGGTGATAGTTTTACACAATTTTAAACTAT

TAATTTTCTAAAGATATGTCTTAACTTGATAATTTTATTATTTGTTCTATTGAATTTGGC

TTGTACTCTGTGCTTCTAATTTTTGTTTGCTCAACTTTGTTTAAATGCTTACAAACTAAT

GGTCTTTTATTTTAATTCTTCTTAGCCATTTGATATAGGGAATCTCATTACTTGGTGTAG

ACTTGATGAATTGACACTGGTTACTGTAGAATAACTCAGTGTCTTTTGTTATTGGATTCC

ATCATGCTTTGCTAGTTCATAGTCATCTGCCATCTTCTTATGATTCAAATGTACTACCTA

AGTAATTAGGTATTGGTTACACCATCAGATAGTTTTGGAAAGATCTTCATTAAAAAGTCA

GATAGGTCATAACTACACTGTGTGCATAAGAATTGGTTTCTAACTTTTGTGTGTTTGCTA

CTTTAACCTTCAAGGAAAAAAGCAGCTTAAGGGGAAAAGGCATTTTACTGCTCTTCAGCT

AATGCTTGGCTGTTTTTTCAGTCAATTTTATTAGTTAATTTACTTGTGCCCTTTAGCTAA

TGCTTACTGCTCTTGTTAGATTTTGCTTAGGAAATATCCCAAGGGGAAAAGGCATTAAAC

GTAGTTGGCGAGGTCGTGGGGGTATTGCTAACAGAGGTGGAGGAAGCTCACAACAGTTAC

TAAACACTTCTCATAACAATATTTCTGAACCATCAACCCCTACTTCTCAGCAAGTTGATT

CAATCCAACATGGTGTCTCATCTAACGAGAGTGGTTTCCGACAAGTTCATACTTCTCAAG

ATATTGGTGCTCAACAAACAACTCTTCTCCTCAAGATGTTGGCGCTTAACAAGAGCATTC

TTCTCAAGATGTTTCTAGCGAACAACCAACACTTGCATCTCATGAAAGCAATAATCCACA

GGCAAGGCGAGCTACTATTCACGGTAAAAGACCCTTGAAAGTAACTGGTGACACATTCTG

CCCACATGACGCTGTTCGGAGTGTCACATTAGCCTTTAAGAAAAACTTCACTGGTTCATG

GCATTGTTGGAGCAAAGTTCTAGAGTATAAACGAGATCAATGGTTCAATGATTTTGAGGT

AATTACAATTAATTGAAATATATGACAAATATATATATTTTTTAATTAACGTATATGTCT

TAACTTGATATTCGGACACTAAATATAATGTTTTCTGTTACAAGAAAAACTTTACTTTTC

CTGATGAGGATAGAGTTCTCATTCGGAAGACTTTTGACTGTGTGGGAAGTGATAGGCTGA

GCGACACATTGGGAAAGGCAAAAAGAAAATTTTTAGTAACAAAGACAATACCAAAGTGGA

TTCGTAAACCTGATTGGAATGCCTTGATGAATTATTGGAACTCGGACGAATTCAAAAAAA

TTTCACCAATCAATTCCAACAACAGGATGTCATCTAATAATGGAGAAGGTCCATCCTTGC

ATACTGGAGGATCTGTTTCATTTGCCGAGTATAGAAGAAGACATTTAATTTTTTTCTTTA

TTATTAAACTTTTTTAGCCTAATTTTGTTATATAATGTGTTAGTGCATTAACATTATCTT

TAATGGTTGTAAAAGGAACAAACAGGGCAAGATCTTCGAGATGATGAATTATTTTTGAAA

ACTCACAAGACCAAAGACAAAGAGTGGATCTGCGAAAAGTCACAGAGGGTATGGGTACGT

TTTTATCTAATTTTTATGTCACTGTCTCTTTTTTGTTTTCACCTATTTGACAATGTTACC

TCTAAGTAACAAAGTAAAAATTATACAGAGTGTTAATGTTACTAGTCTTTAATAACTATG

TCCAAGTTTAGAGTGTTACCGGCTTTTACTTTTAAGTCTAGAACTCTGCTATTTTACTTT

TTCATCATTCTCATCACTTAAATTGGAATTTGTTATTAAGTTTGTAAGCACTTTATTGTC

CAGATTTCATCCAAATTTAAGCCAAAATTATTACTTAGATAGGTTGATCATAACTGCCAA

ACAAACATCTAGAAACCAATAATTCAGAGACTTTAATGCCCATAAAAAAAAAAAAGAATC

AGACACGTCATTAGCCAAATGTTTCAGCTATGATTTTATCCAACAGGTTTTTTGTACTAG

ACAATAGCAACAAGGCATTGAAAATAAAGTTGAACAGGAGCTACTGCCATGATTGTGAGT

CTTGTGAGAATGAAATGATAGGAGTTGATGCATCCCGTCCTTGTTTTCCATTTATAGCAG

AATTTAGCACGTTGTACTGCTGGTTTACAGAGTCGTAAAATGCCTTTATACTGCTATCTT

AGGTTGCATTACGAATGGTATCCGCTCTAATATACTTTAGTTTATTTGACATGGACCTAG

TAGAAAATTTGTACCTATAAAAAGCAGCGATCAATTCATTTAGTATGGGAGATTTTTTAT

ATGGAAACCACAGATGTCCTTGTTGAAATCATGAAAAAAACTGCATTAATAGATGCATTA

TCATTAGCAGATGCATCCTCTCCTGCAAGATATGAAGATCTCTCAATTCACCTGTTACAT

ACCAATAATGATCCCCTGAGCACCTTTATGGTAGAAGCTGGTCAATGTTCTGAAACCTTT

CTTGTCCAGCTATAGGAAACATGATATGTACATTTACGACTTTCTGTTGGATCTAAGCAA

AATTAATTACTTTATGTTGGATTAGAACAGCAATAAAAGGTAAACTTATGATAAAACTGA

TTACAGTAGTTTTGGAAGAGGAAAAACTTAGCGGATGATCCGCTAGAAATTTAGGAAAAA

CTAATTAATTACTCCATGGACATAGCCAGATTATTTCTGATCTATTCTTCTTCTTCTTTT

TTTTTTTTTTTTTGGCCAGANAGGTCGGTGAAACTCCAACATCAAGGAATGAAGAAGATT

ACGAAGCAGATGATGGAGATATGGAAAGGATCATTGCAACAAATGAAGATGAGGATGGAG

AACAAGATAGTGAAGACAATCTGAAGAATTTATTGCAAAATATATCGGAGGAGCAACTAG

TTAATGCTTGGGTAGAAGCAGTTGGAGGAGTAAAAAAAGGAAGAGTTTATGGACTTGGTA

CTGGAAGTTGTCTTCAAGGAAACCAAGTGCATGTTAATTGCGAATCCTCTAGAGCTTCTA

CTTCTACAAGTGTTCCTATCCAACAATTATTTGAATCACCCGAATTTGAGCAACTGCTTG

ATCGTGTGTTGGAGAGACGGATGAGGAATGTGCAAGCAAATATACAGGAGAGTGTTCGAG

CTGCTATGAAAGAAGATGTTGGAGATGCAGTTCGTGCAGTCGTTGGCCGGATGCTTGGAC

TCTCTCCACATCCACCAACAAATGGTTCTGGTTGACCCCCAAGCCTACTTTAGAATAATA

GAGTTTATGTTGCTGCTTTTTAAGTACAAGACATGTTTTATTTTATTTACTGCTAGACTT

GTAGTTCATGTTTTAAGGATTAAGAGTACTATTTAAACTGGTAATATGCAACTATATATT

TTTTGGCGTCT

>XLOC_016794 transcript=TCONS_00027523

GGAGCATGCATTTCTGATGATTATTCAATCGACAAGATGATGAGTCTGAAGTCCTTAAAC

ATGCCATCAGGAGAACCTGTTCCTTTGCGCAAAAGGAAAAACCAGTTAACAAAAGGTGAT

CTGGTGGGAGACAACACAGTCACAGGGAGCACTCTTACATCTGCTTCTGCTGGGCCTGAC

AAATATGCTTTGAAAGACTCTTCACATTCTGCTTCTTGATTTGATTGTATTATCAGTACA

TTTAATGAGGGAAAGCATCAGAAGAACATATATAGTTCTCTGCGTCCTAAGGACTCAAAA

ATATCTAATACCAGCAAGAAAAGGGGAAGGTCTGGTGATAGTCATAGGCCCAGACCACGG

GATAGGCAATTAATCCAGGATCTGCTCAAGGAGTTGCGTCAACTTGTCCCTAATGGTGCC

AAATGTAGCATTGATGGTTTACTAGATAAAACCATAAAG

>XLOC_023960 transcript=TCONS_00039383

GGATCAACATCTGCCGCACCTAACAAAAAGAGCTATAACTCACTTGGAAGTGTTGTAATT

GAAGGGGCTTATTGACATTTTGAGGCTTGGTGAACGAGATAGTTCTAACATTGGAAAACA

GACTTTTCTACCTGCTTCCTTTATCGGTGGACCAAGAGATATGCGACAACGATACATGGA

TGCTATTTCATTGGTGCAACATTTCGAAAGCATTAGAGCTTCTCCAGCAGCCAAAGAAGC

CAAAGAAGTTCACTTTGAAAGGACTATAACAGTTATGGAAGAAGATTTGGTTTTACATAC

AAGATTGAATGTTGAACAACAAAGGGCATATAATGTGATTACTCACAGAATATTCACGAA

CAAGGCAGGAGCATTTTATGTTGATGGTCCGGGTGGAACAGGTAAAACTTTCTTATACCG

TGCTTTACTAGCGACAGTGCGTTCCAAGGGATTTGTTGCTTTGGCAACAGCGAGCTCTGC

TGTTGCTGCCTCAATTCTCCCTGGAGGCCGTACAGCACATTCACGTTTTAAGATACCTAT

AGATCTCGCTGAAAATTCAACCTGTAATATTACCAAACAGAGCGCACTAGCACAGTTAAT

ACAAGATGCAAGATTAATCGTATGGGATGAAGTATCAATGGCGCGAAAAAAAATGATTGA

AATGTTTGATTTACTTTTACGAGATCTTATGAATTCGAATACATTATTTGGTGGAAAAGT

AGTCGTATTTGGAGGTGATTTTAGACAAACTCTTCCCGTTGTACGAAATGGAAAGAAGGA

GGATTTTATCCATGAAAGTCTGTTGTACTCTGATATTTGGAGTAAATTAGAGAAAATGCA

TCTAACAGAGAATATGCGAGCACGTGCCGATCCGGCCTTCTGTGATTATCTTCTGAGAAT

TGGAAATGGAACTGAAAAGATCATAGACAACAAAATTGAGATTCCAAAGTCACTACTTAT

TCCTTTTTCTACTGAAAGTGAGTCTTTGAATGCATTGTTTGCAGCAACATATCCGGATAT

GCACACTTTTTTTTCAGATACATATT

>XLOC_028024 transcript=TCONS_00046085

ACCCTAAGCCTCTTGGCTTGGGGAAACTCTAGCAAAATTTCTCCCTCCCCGCCGTGAAGT

CTCCTCCGCCGCTACTCCACCATACAACCGCCGCTCCTCCGCCGGTCAAACCACCGTTGA

CCGCCACCAACCGCCACCGGAAAAATATCAAAAATACATGGATCGACTCCGTTCGGCCCC

CTCTTTTCATTTCTCAACTCGGTTTCTTTAAAATCATAGCCGATTTTCGTAGATCGGGCT

TCAAAGATTCGGTCAAACCTCAATTCCGGCTGTGGAGAGATTTCGTGGCTCAGATTGGAA

CTTGCTGGTGGGCAGTTTGTGTGGGATGAGTATGAGGCTGTTGTGGTGTCATTCGGAGCT

GCTGTGATGGTGATGATAATGGCGTTTGAGGGGCTGTTATGTTTTCTTTATTGGCCAGCC

TATTAATGGTGGCGAGGTGGCGTCAACGAGGAAGCGAGGGTATTTTGCTTGGCTACGGGT

TAGCAAGAATAACCCCCAATTTTCCCTTGTTCTTTTTCTCATTTTTCTTGGTCCTTGTTG

GTATCTAGATAATTAACAAATTTTAGATTATAGTACGAAGAACCATTGTGAAATGTCTTG

TTGTTTGTTTTTTGTTTAAGTACTTGTCTTAGTCTAGTTTTAATTTCTAAATGTATGAAA

TATAGGTTGTTCTTGTGTAAAATAGTGCAATTAGTTTTAGCCAAGGATCAATGATGTATA

TTTTTGTTTACATATATGAATTTATGACATAGAACTTGTGTTTAAGATATGTTTGATTCC

AG

>XLOC_019043 transcript=TCONS_00031241

GTGGAGATCTTGAGCTGTTGTCCAAGGAGCTCAGATGGCTGTCGTGGAAAAAATGTCCTT

TAAAATGTATACCATCAGGTTTTCCAACTGAGAAACTTGTATTTCTAGATATGAGAGGGA

GCAATATCCAAGAATTTGGTTTGAATTTGCAGTGTTGTACCAATTTGAGGAAGCTGGATC

TCTCTGATTGCAAGCACCTCAGAAAAACCCCTAACTTCAATGGTTCGCGAAGTCTTGAGA

CTTTACAGTTTTCCGGTTGCTCAAGTCTGACGGAGATCCATCAATCAATTGGAAATTTGG

ACAGACTAATTAGTCTACAGTTGGGATGTTGTAAAAAGCTTACGGATCTTCCTAGCAGCA

TATGCCATCTCAAATCCCTTAAAAAGTTGGACATTAGTTGGTGCTCATCTATACAAACAC

TGCCAGTTGATTTAGGTGATATGCAAAGTCTAGAAGGTCTTTATGCAGGTAATACAGGTA

TAAAAGAATTGCCTGGATCTGTTGAAATGCTAAAAAATCTTGCAGTTTTGATAGTGGGAA

GTCGAAATTTTGAGGCCAAAAGGACTACTTTTTCTCAAAGAAGTTTCCATCGGACACAAT

CCTTGTCAAGAAGAGTCCATCCTGTACAATCCTTGTCGACTTCTGTTTTTCAGTTAAACC

TTTCTTATTGTGGTTTGTCCGAGGCTGATATTCCCAGGAATATTGAGAGTTTATCCTCTT

TACGATATTTAGATTTGAGTGGCAACAGTTTCCGCTGTCTACCCTTTGGTTTTACTAAGT

TACACTTTTTGAGGTCCTTGTATTTTAAAGACTGTGTGTATCTGCAAACACTCCCACCGG

TATCAAATTTAGAATATCTTCAAAGGCTTGAACTTGCAAATTGCAAAAGATTGGTGAAGA

TTGCAGAGTTGGACAACCTCCCTTCTATAGATTATATCGACATGGTTAATTGTAGCTCTC

TGCAGAATCCAATCAACGAGGGCTTCTTTAATGCACCTGCTCTAGCATTTCCATCTAGAA

AAGATGGTTATCGGTGGTCTGAAATTCATCATCAAAGCAATGAGATTCCAGGTTGGTGCA

GCAATCAAGTAACAGCTTCATCTATCTGCTTCACTATGCCGACAGATAATGAGGAGTATA

AGTTTATAGGAATGATTCTCTGGTTTGTTCGCAAGTTTG

>XLOC_026590 transcript=TCONS_00043776

GGTATTTTCGATACACCTCAAGTTATAACAATTCTTTATTTTTCATAATTTCCAACAATA

TATAGATGCTCAAACAGCTTTAAAAAGATATAGAAGTCCTAATCAAAGATATTAAAGGAA

AAAAAAAAAAGGCGATCAGCTTGCCCTTTGAATCCAAAGCTTCTTCAATCTACGGTGAGA

CAATATTGCTGTCACGAAGACCCAATTGATGATACTAGTCAACAACTGAAAGTAAGAAAT

TCTACATGATTGATTGAGGATCAAAGTGAATATTATTCCCCAGAATACAAGAATGTATCC

AGTTGCCATAGATACATAAAATCCCAATGACACAAGTTTGTCGTCTTCATCAAGATTGTT

GCTCTCATTGTTACCTTTCCTGCAGCCATCACCGTTCCGGAAACCATCACCTTCAAAGCC

ATCTTTACATCTACATCGAAAACGCTTTCTATTCCCTGGAAATAAAATGTTTGTACAAAT

TCCATCATTATCACATGCACAGTCTCCTTCTAATCCCCAAGCCAATTCCAATGTCTGAAA

TTCTAAGTACATAACAGAACTCTTCTTTGTATTATTGTCCATACCAACTAGTATAGACGA

GACCACATTTCCACACCCTGTATTCCTTAATTTCAAATAATCCAAATAATCAGCACTCCC

TTCTGAATAACAACTTACGTTCTCTTTTTTTGAATCACACGATGGTATATTCAAATGTGT

ACTTAAAAGTTCCGATGGTATAGTACACTCACTTCTTGGGACAGTGCAGTTCTTTAACAA

AAGTCCATTTCTCCAAGTCATACCATAATTTGTTCGACTAAATTGCTTAAGATCTTCAAT

CGGACGGTTACAATTTACTGGAAATTTGACCATTAACGTATCTGATGTCACATTTTGTAC

TAGATATTCACCAAGTCTAAGTTCTCCAGTTTCAGTACAATTCAAGTGAATTTGACAACC

TTCTGTGAACCCAAATGGATAAGGAACACGCCGGGCATTAGAATTAGCTGCTCCACAGTA

CTGATTACACTGAATTGAAGTAGTAGCACCAACTGATATTGACATTGATGATGACCACAC

TAATAAAATTGCGAAAACGAATCTTATAATTATGTGATGAAAAACCATGGTCACAAAATT

GACAATGAGTATATATTGCT

>XLOC_013468 transcript=TCONS_00022069

CAACTTTCTCTTCTTTCTCTCAGTGAAATAAAACATTCTCTGTGTGGCTGTGGACGTAGG

CAAAAATTGCCGAACCACGTAAATCTTGTGTCATGTCTTGTCTTGTGAATTTTATTATTT

TTATCATTTAAATATTTTCCCTTTCTACTAGCCTAGCACGCTTCCCAACATGAACTTCTC

TTCAAATAAGGCAACAAATGTAAGGAAAAACAAAATGTACCAACTAAAGTCCATATGGCT

GTGAATTCATAGCCCCTCCAGGCTGCATCCCCACGAGTAACGACCGATGAGCAAATCATC

CTCTAGGACCATCAATTCAGCAGGAGTAGTCATCAGAAGCAAATAGTAACTGTCTAACAG

AGATATTAACAAAGGACAGATCGTATAAGCAAAGATGTAACTTAGTAAACTAGCAAAGAA

AGTACATTCTGCCAAAATAACCTTAAGAACCAGAAATGCAAGTTATCAATTTTTGTTGCT

CCAAGAAACTGTATGATTATTGGGTGTATCATTTTAAAATACCACTTCGAGTCCTCTACA

CATGGTTGAATGCCACTAGAAAGTGTATGGTAGAAGCACCAAAGTGATTACAATCTTTAC

AATGGTTATTATAAAACATTCCGAAGTTTTCACCGAAGTCACTAATCATTCCAACCAACA

GTCTGTGGTTAGTGCTCTTCTTCTTATGGCATAGTATTAATATTTTAATTTGTCTTCTTT

CGTAAATTATTTTGATCTAGACTTCTAATGTTTTAAATCCTACCCATTTTAACATAAATT

TTGCTATTGCAATCATAACATTTTACAGTTTAACAATTTATTACTACAATGCAGAAGGAG

ATGACCTTTTTCTTTTAACGAGTAGTGAGTATGTTTGTGATATTTCGTAGATATTAATAT

TGGGAGGTTTCGTGAGGCCTATATTCAATATATCTTGAGTCTATGAACCTTGAACAAAAT

GCCACTCCTATTTGAAGTATCCTACACCAATTTTGTTAGACGTGGCAATCATTTGGCCAT

AAACTATTGGCTTTGTTGGCCATTTGTGCTTCTCTTGTTTTGTAGCCTTGTTGGCATTTC

CTAAACCAACCAAAGTGTTTTGGTTTTGGTTATAAATTCAGCCGATTGTGAACTCTTCAA

ACACACCAAAAAAACATTGAGCTTCTTTACTCAGTGTTTTATCCCTTTCCTTTTATAGAG

AGTATTTTGTGAGAGAGTTATGTTTTGGGAAACACTTGTGTGAAATTCTTTCTGAGTGTT

TTTGTGAGGTTATTCTCTCGAGGGTGTTTGGGATTAATTAGAGTATTATCTCTAATTTTG

TACTCTCTATTTTGTATCCGTTGATATAGTGAATTTGCTCCGCTCCTCCGTGGATTAAGT

CACACTGACTGAACCACGTAATTCTTGTGCCTCAATTATTTACTTTAATTGTCGTTATTA

TCAACTAACATAATATTTGTTATTGTCATTGTTACGCTATTTTGTCTTATTCCGCACTAT

ACTCGGGTCACGATCCTAACAAATTGGTATCAGAGCCGGATCAAACCGGGTTAGTTGTGA

GTAGCCAAGATGACTATAACAAAGACATACGTTGAGAAATTTGATCGAAGTGCAAACTTC

AGAATGTGGCAATTAAAGATGGAGGCTATCCTAATTCAGGATGGCGTAGATGTGGCGCTA

CAAGGCAAGCAGGCGAAGCCGGAGAATATGACAGACGAGGAATTTGCTATTATTGACAAA

AAGGCAAAATCAGGTATTATTTTAAATCTCTCAAATGAGGTTTTGCGTGAAGTAGCTGCT

GAAAGCACTGCTAAAGGAATGTGGGATAAATTAAAGGCACTGTACTTGAAAAAGACAGTA

GAAAATAGACTCTACTTGAAGCAAAAGCTTTACATGCTTCGTATGGATGAAGGTACTTCT

TTACTCTCACATCTTGACACATTTGATTCCATTCTTATGGATTTGGCTAATATAGATGTT

AACATTGATGAAGAAGATCAAGCTGTTTTGTTAATATGTTCCCTACCCCACTCGTTTAAA

CACTTTCGAGACACAATTCTTTATGGG

>XLOC_008928 transcript=TCONS_00014545

GTAAGTGTTTTCCCTTTTCTTTTTCATAGACAATTTTTCTTGCATGCTATTTTATCTTTG

ATTTTCTTCCTATCTATGCTGTCTTTGTTTAGTAGAAATTTACAAATTTTCAATTAAAAG

AAAAAAAAATACTATATTAGTAGCTTAGAAAGCATGATTGTTAGTGGATTCTAGTAAAAA

AGAATTTTCTAGAGAGAAATCGAGTTTTGATATACTTTCTTATATTGGGTTTATCAAAAC

ACTTTTTTTTTTTTTTGGAAATTTTTTATTCTTGGATTGTATAGTTTGATGAGTGCAGAG

AAGCTCATGAATCATGTATTAATTAGTCTGCATTTGGTCTATTCAACGTTGATTTGAAGG

GAACTAAGTATTAAATGTCAGTTTAGACCCTACTTGGTTTAAAGGTTCAGTAACTTGGAG

TACCTTTCTATGTTGTTTTTGTGACAGAGTTGCGACATTATTTTCTTAATTAAGACAAAC

TTAAACGTACTGATTCATTTTTATATGGGTAAATTGGGATCTGTGAAAGTTTCTTGAATG

TATAATGAAACACAAACTCATATTAAGGATTGCTTTCAATTAATATTCTGTGAAAGTGTC

TTGAATGTATAATGAAACACAAAGTCATATTAAGGATTGCTTTCAATTAATATACACTCC

AGTGATGAACGATTAAGCTTCAAGTTTACAAGTACTACTGCACTACATTGTTTCTAGTTG

GATGAATTTTGTTGCTAATCAAGTATTAATGTACATGTACTTTAAGCAGAGGTTACAGCT

TCGTCCCAGATTTGTGAATCAAATTATTTTTGTGTTCACCAAGTAATAGTATTATCACAT

TTAATTATTGGAATATCCTTGCTACTATACTAATTAAACAGAGTCAGGTCCAATTCTCAT

GGAGAATTGGCTAGTGATAGCTAGTACCATGATGGATTCACACAAAGAAATATTATTCAT

TTCAGATTGTTGTTCACATCCATATATTCTATTAATAGCATATATTCTTTATTTGAATCA

AAGATTTAGAAGGCCTATTATATTGTTTTCTAGTATCAGTTTAGGTGATGATCATCATAA

GCTTGCTAAGATAGGCATACTGGTGATTTGTTTAGGGTCTTATTTTCAATTTTCTATTGT

TTATGAATAGTGGATATGTCAATATCATTTACAGTAGCCTAATTAAAAAAACCGTGTTTG

TTTCCATAAGAATTTATTGTGTAGTTGGACTACCTTAAATTTCATTAGTAAATTTTCTTT

TTTACTTTGATAGAATGGAGTTTTCTGAACATCATGTCATGTTCTTCATAATTTTTATTA

GTGCTTTGTCAAAATCATGCCTTTAGGACTTTTTTGATCTGCTTGTTAAATCTCTTCTAC

AATATTTTTTATCTCTCGTCTATAATATAATCATCTTTACTGTATCAAATATCATTCTTT

AAAGACTAATACAATATGGTTCTAGTTTCGAATGTTGTAATCATTCTTGTTGGAAATTTT

TGTTTTTTTTTAATTTTCTGTTGATTCTTTACTTTTTCAATTAAAGACCTTAAGTTTTAG

AAAAGATAATGTGATCCAGGTATTGACATGATAACTGATTCCGTTAAGAGATATTTATAT

TACTATTTATGTGAAATTCAATATGGTGAAGATTTCACTATTTAGAACCTGAACAATAAG

GCGATCTGCTTCATGTGAAGTTCTGAAATAAATTCTTTGAGAAGTTTTATGATGGTAGAG

AGCAGCAAAAGTCCTTCCTCTTCACCTGGAATGGATAAAAGCTGGTTGAATATAAGAAAT

AGAGGTAACAGAAGGTATATAGATGGGGTAGACGGCTTCCTTAAATGGGCATTCAATCAA

CCAAGAGTGAACACTTTGATTAGATGTCCTTGTAAAGGGTGCATGAACACAGATTACAAG

ATAAAGATCGAAGTAAGGAGAGACTTACTGAAGAAAGGTTTCTGGAATTCTTACAATGTT

TGGGACTTGCATGGAGAATTATTAGTAAGAGATGAAAATGCTAATGTTGCATCCAGTGAA

GACGTAGATGACGATAATGTTGAAGATGATGATATTACTGAAATGATGCATGATGCTTTT

GGACATACAAATATGGAAGATGATAACAGTTTATCCGAGAACAATGAAGTACCAAATGTG

CAGGCAGAGAAATTCTACAAATTATTGGAAGATGCTCAGACAGAAATTTATCCAGGCTGT

AAGAATGTCTCAAAGTTATCTTTTGTCGTTACACTACTTCACTTGAAGTGCCTTAACCAT

TGGAGCAACAAATCAGTGGATGCACTGTTGAGCTTATTTAAAGAAGTTCTTCCGGAGGGA

TCATTTGTTCCAAATTCTTACTATGAAGCGAAGAAAGTTCTTCGTGACCTAGGTTTGGGG

TACAATAAAATAGATGCATGTGAGAATGATTGTATTTTGTATTGGCGCAATTATGTCAAT

GAAGAATCATGTCCAAAGTGTGGTAAGTCTAGGTGGAAGTCCCAAGAACATGGAGGAAAG

AAAGTAGCTAATAAAGTTCTGCGACATTTTCCAATCAAACCAAGGCTTCAAAGATTGTAC

ATGGCAAGAGAAACAGCAAAAAAAATGAGGTGGCACAAGGAAGAAGGTATTGATGATGGT

GTTATGCGACATCCGTCTGACTCCATAGCCTGGAAATCCTTTGATGAGAAACATCCATCA

TTTTCAGCTGAATTAAGAAATGTTCGGTTAGGTTTAGCGAGTGATGGTTAACAGCCTTAT

GGTAATATGAGTTCTAATCATAGTATATGGCCAGTTGTATTAGTACCGTATAATTTGCCA

CCTTGGGATTGCATGAAGGATGTGTATTTCATGATGACTCTTCTTATTCCAGGTCCAAAG

TGTCCACGTAATGACATAGACGTATACTTGCAACCAATGATTGAAGAGTTGAAGGAATTA

TGGGGTGGAGTGGAAACTTATGATGCACATTCACAATCCAATTTTAAAATGCGTGCAGCT

CTCATGTGGACGATTAATGATTTTCTTGCATACGCTAACCTCTCAGGATGGTCAACCAAA

GGCAAGCTTGCATGCCCTTGTTGCCATAAAGACACACATTCAATTTCCTTACGAAGTAAA

CTGTGTTATATGGGCCATCGTCGCTTCCTTCCAATTAATCATCCATGGCGCCGAAATAAG

AAGTTGTTTGATGGGACAGTAGAAAAGGGGGTTGCACCTAATACTTTATCTGGTGAAGAA

GCACTTGCACAACTACAAGGCCTGGGCAATGTGAGTCATGGGAAAGGGCAAAAGAGAAAA

CGCAATTTTCCCAAATGTGCTTACAATTGGAAGAAGAAAAGTATTTTTTTCCAGTTGCCT

TATTGGAAAACTCTCTTGTTACGACATAACCTTGATGTGATGCACATTGAGAGAAATGTT

TCAGATAACATTGTATCAACAGTGATGAATATGGTTGGAAAGACAAAAGACACACCAAAA

AGTAGATATGATTTGGTGGACCTTGGTATTAGGCAAAATTTGCATCCAGTTGAGGATGGG

GTTGATGTATTATTACCCGCAGCTTGTTATGCATTGTCCCCTCCAGAGAAATTGAATTTA

TGCAAGTTCTTGGCTAATTTGAAGGTTCCTGATTCATTTTCGTCAAATTTTTCAAGATGT

GTTAACATACAGGACAAAAAACTACATGGATTGAAATGTCACGATCACCATGTATTATTG

CAAGACATTTTACCAATTGCAATACGTGGTTTCTTATCTAAGGAAGTGAGGGAACCTATT

ATAGAGCTTGGCAAATTTTTGAAGAATATTTGCTCTAAGTGCTTGACAATTGAAGATCTT

GATAGACTGGAAGCTGAAATTGCTATCACATTATGCAAGCTTGAAATGGTTTTTCCTCCG

GTATTTTTTGATGTCATGGTTCATTTGCCAATTCACTTGGCAAGAGAAGCAAAACTTGGT

GGACCAGTTCAATATCGGTGGATGTACCCTATTGAGAGGTAAATGTATTTTATAATTTCA

ATTTGCTACTTTTATGTGACATATTACATTAATGAATCTTTGCATACAGGTATTTGAGAA

CACTTAAGTCATATGTTCGCAACAAAGCTCGCCCAGAAGGATCAATTGCAGAAGGTTTTT

TGGCAGAGGAGTGCCTTACATTTTGCTCGCGATACTTAAAGAATATCCCTACAAAGTTCA

ACAAACCAGTAAGAAACGATGATGGATCCGTGTCAAATGATGAGATGTCTATTTTTAAAA

AAAGTGGCCAGACAAAAAACGCTTCAGACAGTATCAAACCACCTCATGATGAGTTCAAAA

AAGCATGTTGGTATGTGCTTCAAAATTGTGAAGAGGTTTCTCCATTCATAGAGGAATACA

ACAGAGAGATTGAAAGACAGAGTTCAATAGACAATGAAATGAATAAATATGGCTTTCTTG

ATTGGTTTCGTGCACGTGTCTTGCTACTATCTGCACAAGGGCGTGCAAATGATGAGCTAA

TTAGCTTAGCCGTCGGTCCTTATCCATTGGTGCATCGATATTCTACATATATGGTGAATG

GATTTAGATTCCATAGAAAAGAACTTGAGTTGAGAAGAAAAACACAGAATAGTGGAGTAC

TTGTGAAGGGAGATGATTCAGACTCTAACAAGGAGTATTATGGTGTATTAGAGGATATTT

ATGAGTTGTCGTATGTTGGAAATAGGAAAGTTTACTTATTCAAGTGTCATTGGTGGGATG

TAGCTCGCTTAGGAAGAGGATATAAGATTGACAAATATGGCGTGACAAGTGTGAATACTC

TTTGTGCTTTGAATACAGATGAGCCATTTGTTTTGGCATCTCAGTCTGAACAAGTCTTCT

ACTTGAATGACATGGTTGATAAAGATTGGCTTGTCGTTGTAAAGACAAATCCTCGTGACC

AATTCAATATGCCTGACCGTGATGATACTTGCGTAGAAGTGGAAGATGAAACATTACTGA

ATGTAGAAGCTTATCAACAAGAAGAAGTTGAATTTAATTTTTTGAGTACCAATGATCAAG

AAATTGATATTGAAGTGTGTTTACACAGAGATGATGTTGAACCACAAAATATTAGTTCTA

ATCATGCAAGTAAACAAGCACAAAACAATGTACATAATGATGAAGATGATTTTATTAATG

ACAATCCTATTGACGTATATGACAGTGAAGAAAGTGAAGAAGGGTATCTTGATGATAATG

ATGGAGGGGATACTGATACATCCCTCTAGCTTATTAGATCAAGTTACTGGTTGTGACTAG

TATATATTACTTGCTCTTTATGGTCTTCAGACTACCTTGTAAATGATTATATGAATAATC

TTGAAGTCGTAGAATCCCTTTATGTTTTTGTCTTGGGGGTCTGTAAATGATTATATGACT

AATCTTGAAGTCATAGAACCCCTTTATATTTTTGTCTTGAGGGATCTTTATCTTTTTATT

ATTTTACTTTTGAGGGTTGTATCTGGCACCGAACACAATTTTTGTGTTGTGGTTTTATAT

ATGTTTATATATATGTATCTCGTGACTATATTAGTAATGTTATTTCTCTTTACTGATTTT

TGTTATTGGCTTTAACTTATTTAAGATGTCTGTCAATTTATTTTTCATGTATAACAGTAA

GCTTGTATCATTTTAATATTCAATACTTGATACATTCTCTTTTATGTGATTTTACAGGAG

ATGACAGGAAGAAGTAAAGGTGGAC

>XLOC_035670 transcript=TCONS_00058642

GTTCTGGCTTCTAGTTTAATCTTTAGCACCCTTTGTCATCTACAATTTTAAATTTGTCTG

AAAGAGCTTAGCCATCTTGTCTAAAGGACTACACGCATAAGTAAGCCAATGAGAATAGAG

ATATTACAAGATAATTTTTGTTAGAAGAGATTGGTTGGTAAGTTATGCAAATCTTTATAG

AGATTAGGCTAAACATGTTGTTCTTTATGAAATAGGAACACCTACAGAAACAAGATTCAT

CAAGAAATTTTTGAAACTGGAACTTTTAAAGATTAGTTTAAACGTGTCATTTTTAACTAT

AATTTCTCATTGAACATTATTTTTTAATATTGTCTTCCTTGATTATTTTTTCATTATCTT

TTCCTTTGACTTTTTGTTAGAGTGCCAAATGGAGATGTGATCGACTTTATTGACTTATTC

AAGCAGCCAGCAGTGTATCGTCCTACCTTAAAAAACTACAAAGAGCTTCAGATGTCAGGT

GAGGCTTGAATCAAACTTGTTGAAGTACAAAGGCTCGGA

>XLOC_003249 transcript=TCONS_00005266

CCGTCCATCTTCCTAAATGATTTTCTACTACAATAAAAGCCCAAAATGAATCAATGATTC

TCAAACGATTCTTCATCATTTAGGGTAACAAATAAGACAATTGAAAATCTCTGGATTTGG

TTTTGGTTCAATTTTAAAGATAGTTTCTCGGGAAAATTTTGTTCGAAAACTTGTTGGAGT

CCTTTATATGAAATCCTTTATATTATTCTGAATTAAGTCAAATTAAGAGATGGGTCTAAA

ATCTAACAAAATTGATGTAGAGGATTTCACATAAATGATTCCAACTAGTGTACAAAAGAG

CAGATAGATTCATTCTTTAGAGCAATCTACCCAGAAATATTTTATATCAATAGTAGTGAC

AAACAATGAGGAGGTATTTGCAAGTCCAACGTATGAGTACATGGATACTAAAATATGGAA

TTAAAGGAAATGCAGTGGCTTTTCGAAATATAGAGTTCTGAACTGCAGTTAGAGGTGGTT

ATCTAGCATTTACCATTTTCTTGTCTGCTCTGTTACCTTTTCTTGGAGATTTCATGAGCT

TTACTGGTGCTATTAGTATAGTTTCACTCACATTTATTCTTCCAAACCACATGTACATTG

TTGCTATGAGAAATCAGCTTTTGTTGTTACAGAAAAGCTGGCACTAGTTCAATATTGTCT

GCTTGAAATAGTGACCAAAAACTGGGTTGGGCAATAGTTTTATTAAGATGGAAAACTGCT

TGAAACAGTGCATCAATTAAATCTGGAGATGCTAAAATCTAGTGCAAAGGATAGTACTCA

AGTTGATTGTATTCAAGAGGGAAAAGGGCACGCAGTAGGTTCAGATGTCTAGTATACCAG

TGTTGTTGAAACTGGAATACAGAATATTGTTGTATCAGGTTTCCCATCCAAATTGGTACA

TCCATTCGTGTGCAAGTTGAAGAGAAATGAGATGTAGCAGCTGTTATTAAACCTATTGTT

ACTGCTACTAACGTTGATAAAAAGGTTGGTGTAGTTAATACTCCAAAGCAACCTGGGCAC

AACAATATTGTTAATGGCATGAAGGGGCGGCTTCTACACCAAATAATCCAGAATAATTGT

AAAGATATGGGTTCTCATGTTGCGTTTTCTGGAGAGGGTGAAAAAGATATTATTGGTTCT

TTAATTGCAAGTCAAGAGGGAAAGGAAATATGATGAACTCTACAGCTCATACAACAACTG

GGAAAGAGTCGCCAGTTACATCTCCCAACCAACCAGGGAAACCTAAAGGTGCAGATTTTA

ATACAGCTCCCAAACCAACTTCGACTGGGAAGTCGAAAAAATTTCTGTTGTACATGGAAA

ATATTCTAAAGATGATGCTATTGTTGTTGAGTTGAATGCTGCAGTAGGTCATGATTCAGG

CAATGATAAGGACAAAATTCAGGCCGTGATTGGTCCAATAGCTCTGGCAGTGCCTTGGTG

GTGTTGCGATGATGTACCGATGATGGAAATGGTGTATTGACAGTGGCGATGGCAAGTGTG

GTGGCTAAACGATAGTGGAATTGGCTTGGTGGTGGTGATGAAGCAGATGACTTGCCGTGT

CGTGGGGCTGCTACGAGGTTGTGGGCTGTTATGAGCTTGTGGCGATAATGTATGGCAATA

ATGTGCAGCGATGGAGTGACGACTCTGCGAATCGGTAAGTAGTGAAACAATGGTGTTGCG

TCGAGGAAGCTTCAGTGGTGATACAACAGCTAGGTTTCACTGGTTACATAGAAAAAACCA

ATTCATTCCTTCTTTTTTTCATTTTTAGCTGGAACATTCATTTAAAGAAAACCATGTTCA

GTGTATTGTTTAGCATAATACCATCCCTGATTCGTATATAGAAGTCCACTGCCTCACTAG

TGTGAAACGTGTTGTCAAGCTGAAAATCAGAATTTGATTAGGAGCTAAGAGAAATAGGCT

TCTTTCTCAATTCATGAAATTTTGGAGTATGGATATAGATTGGTCTCTTTTTGTATTTTT

AATGTAACAATTGCATAGCTCCTCTTTCATATAGTTGATGTTCGTTGTAATTTTACTATA

TCGTTCACTAATGTGATTTCATGAGATTCATTTGTGCCA

>XLOC_000007 transcript=TCONS_00000010

CTTCCCAATCCCAAAATCACTCTCATATCACCTCCTAATCATCCCTTAATTGAGTCATCT

CCAACCGGCTCTCTTTGATTCATAATTCTTTTATATACACACAATATATCAACACACAAG

AAGAGACCGGAGAACCAGTACAAAATAGGGGAGAAGACCCCAATCCCCATCTTTTTCTAC

CATCATTATACACCCCAATACACACAAGAACATGATACACACACACCCCTGAGGTCATCT

TCTTCAATTCAACACATACCCCAAACATCCCTTGTCTTCTTCTCCATTTTTCAACACAGA

CTCCATTCGTTCTCTTCTTCGATTTATCATACAGTGGGATACACACACCACCACCACCTT

CTTAATTTGACGCAAGGGAATAAGGGAGATCTACAAGGCAGACGGTGCAGTGAACCACAA

CCGGCGACACTCCAGCCGGAATACAGATCGCAATAGAGGGGGGTAGTGTCTAGTGAAAGG

AGATCGAAAACAGGGGAGAAGAAACGGGGTCAGAAAAGGGGAAATCGAAAAGGGGGTCCA

GGGTGTGCAAACGCCACCAGTAGCGTGACGGCGACGCCGCAGTTTCGTTGGAAAATTTAT

GGCAGTCGCCCCCTCACATCTGCGCCACTTCTAATACTGTTCACCGGAGATGGGTCTGGC

TTCGACCAGCGAAACGCTATTGTTCCCTTCCGTCGGAATTCCTACAAAGAACGAGGTCCA

CGACAAAGATGGAATATCTATTTATTTATGTA

>XLOC_027974 transcript=TCONS_00046001

ATTTTATGTGGCTAAAGTCGAAATCCAATTTTTTATGGAGGTCTAAATTCTCCTTTTTGT

TCTAAATTTAAAGGAAAAAAGAAAAAGCTGCGAAGACAGGACAGCAATAGGGGCTTCCAA

TATTCTTCCAATTCCAAAAAGGGCAGAAGTCTTCTAGATCTGCCCCTCCGCCCCCCCCCC

TCCCACAACCCCCCTCTCTCGTCTTCCAAATCTGCTACTGAAATTTAAAAGATGGGATTG

TCTTTTACCAAGCTTTTCAGTCGGCTGTTTGCCAAGAAGGAAATGCGTATTCTCATGGTA

GGTCTCGATGCAGCTGGTAAGACAACAATATTGTACAAGCTCAAACTGGGAGAGATTGTT

ACCACCATTCCTACCATTGGATTTAACGTGGAGACTGTGGAGTACAAGAACATTAGCTTC

ACTGTCTGGGATGTTGGTGGTCAGGACAAGATTCGACCATTGTGGAGACATTACTTTCAA

AACACGCAAGGACTCATCTTCGTGGTCGACAGTAATGATAGGGACCGTGTTGTTGAGGCT

AGGGATGAGCTGCACAGGATGTTGAACGAGGATGAACTGAGGGATGCTGTGCTGCTTGTG

TTCGCCAACAAGCAAGATCTTCCGAATGCTATGAATGCTGCTGAGATTACTGATAAACTT

GGTCTTCACTCCCTCCGTCAACGTCACTGGTACATTCAGAGTACATGTGCGACCTCTGGA

GAAGGACTTTATGAAGGGCTTGATTGGCTCTCCAACAACATTGCGAGCAAGGCTTAGGAG

GCATGATCGTTAGTTTTTGCAGTTATGTTTGTTTCATGTCCTTACTGATGCTTCTTTGGC

AAGGGGATTTGGCTTTTGCAAAATACTTGTTTAGTTAACCTTCTTCCCGCCCTTCGTTCA

AACATGATGGTACTTTGCAGTTGCTTTAGGTTAAGATTCATGTAGTAATATTTTTGCTTT

CTGGCTTACATGATAGCAGCCGCTTGTCTTTTCATATATTATTTTATTGAGTAGGGTTCT

TCTCTGGCATTGCTTAAATAAAGTTTTTAATTTGCTCTTTTATGCTCTTCGCTATCAGTG

CATATGTTTTTACTATTTCCTTTATTCAAATCGTCTTCTAATCAAAATTCATTTGGAACG

TATGCTCTGCC

>XLOC_000132 transcript=TCONS_00000224

GAAAATGGTAGGAGGATGGATAAAGAGGATTTCAAGCAATGGTTCAGAATAGACCTATGT

CATTCATACATCACACGCAATGATCTGATCTTCCAAAGAGAATCCATGAGTATCTTCCTT

CTATTAGGTTAGAATAGTTTTCAAATATAGTCGAAATGAGTGGACTGCAGCTCTTACCAA

TGGTGACCAGAGAAGAGTCGTTAAGAGCTGGATTGCTATCGACAATAAACTGAATGCATC

ACGTGTTCTTTTCTTTGAACTTCTTGAAGCTTCAATGGGGGCTGCCATCTTTAATGTCAA

AGTGATACCAGAAATACCTTCTCTGGATCAAGG

>XLOC_034741 transcript=TCONS_00057120

TGGTCATATATATTAGGAAGAGAAGTTACTTACATTTATTTGTAATTGTATATAAAAATT

AAAAGAAAGCAAAAACATATATTTATTACAAAGCTAAACTAATCCCAACCCTAACCTAAA

CTTCACTCACTTATGCTCTCCAGCCGCCTCCCATCTTAACCCTCTCACAATTCTTGCAGA

AGTTATATACAGAGGGAATATATTGGGCTATGAAAGTCTTCATACCCATATATAAGGTCG

AGTCACCCAAGCATAGCTCCAGCCAATTTTGCATTTAAATTCCAGTTTCTACTAATATCA

ACTTGCTTAAGAATTCCCCACATCTTGAACTACTAATTTGTATAAGGCTAATAAGAATCG

AGTCCACCAAATCCACCGCAGAGATGCAACTTTAGAGAAAAAATTGAGTAGAGTAAAACA

AAAAACTAGACAAAACCTCAATTGTGTTTTTTTATTCTATACATCCTTAACATGTCATAG

CCCTAGTTCTATGTTCTTCACAACCACTGGTAACTGTCGACATCGATATTCCTACTAAAC

TATTATATGGAGCTTCACTGCTTGCTTATTTATGAATAGAAATTTTCTCTCATTGTTCTT

TTCCCAGAGCGCATTATAATTATAGGGTTGGAGAAATACAACAAAGCTGGCATTGCAGAT

TTACTGGCAGCTAAAGAATCACATTAGCTAGTTTTTATGTTTCAAACTTTACTATTTATG

TTGTACATAAAAATTATGTAACGTGGATGCAATGAGTTTTAGGTTTATGATTATGTATTT

TGGAATTTATATTTGAAAAACAATCAATTTCATTTCGTATGGAGA

>XLOC_016241 transcript=TCONS_00026604

CTCAAAGCTCGGTGTCTCTTCAAGAGGAGCCGGACTTGTGTTGTGCGGTAAAAATGCTAG

ATTGAAGACTTCAACAACTCCAAAAAATCCTGGACGCAGGTTCTATGGTTGTACAATCTC

AAAATAATGGCGGATGCAATTACTTTAGGTGGTTTGATGGTCCAGTACCGGAACAAACCA

GTGTAGTGATGTCAGGGTTGTTGAGAAGGGCCACAGAAGCTGAAGCGGAGGCTAATCAAC

GATCAAGTCGAGT

>XLOC_004026 transcript=TCONS_00006559

AATTATTTAATTATTTATTATTTATCTATTATCTATGTACTTATTATTTACTTATTTATT

TACTTATTATTTACTTTTTTATTTATTTATCCTATATTTTTTCTAAAATTTTAAATTATT

TTATCTTTATTAAAATTCGTAAAAATCGCCGGATTAACCGCTTTTAGCGGATTTTCGAAA

GTGCTCTTTTAATAAAAAAAGACCTTCTTTCGGAAATAAAAGAGCCACTTACTCAATCTC

TGATTTTACTAATTTCTTTTTAAGTAAGGGAATAATTGCACTCTTTAATTAGTGAATGGT

TCCTAATAATCCTAAATTAGGTGGCGACTCCAAAGAAGCCCGTTGGAGTCCCAATTTGTT

GAAAGTAAATCCCGATGCATATTTATATGTATTTTGGGAAGGTTCAACAGGTGTCACACG

TAGTTCCATGAAGTATCTAACATCACCACTGCAGATAACAACTTAAATCGTGTAGTCGGG

TAAAAGATATGCTATGTTCCATGAAAAAATCTATAATCAAAGAGTACAGAGTTTAATGTG

TTAAAAATCAAATAACTAAGGTAAACAAATCCAAATATTAAAGAACGAGGTACGAAACTT

ACTAGGTCATCATTTATAGATGAATATTTATTCACAGTAAGAGTGAAACATGTACGGTTC

CTTGTAGTGGTGTACATTCCATCCGGCCAGACATAGTCATTACTTGCATCTCCTGCATCT

TCTGGCATGTTTTAAGAGGCTTGGGTGGAGATCAAAGAGGTGTAAAAAGTAGGGGTAGAT

GATTAACTTGTTGATGGGTGTTAAAAAATTAGGAAATGAGGGCCCTATTTATTCTCTATA

GGGGAAGAAGGTCAATAGACAGGAGTTATTGGAGCCGTTGTATCAGATGAGATAGTTCAT

ACTTTGGGCAGTGCACCCTTTAAAGAACCTAGCACACGTTTCAACTTGCTGATTATCAAC

CACACAATCCACTAGCAAGCTAACTGTTGGAAATGATGTCTTCAGCCTATGCTGAGTTGG

TCTATGTTCAAAAAGTTTACAACCAACAGTAATTACTACAAATATCAATAAAGCTAGTTA

AGAGTTTTTGTCAACTTGTTAAAATCGAGAGTTAAGAACCTCAACCGCAGAAAAAAAGGG

TCTATTATCAACATATCTAATTCTAATAAACAACAAGACTAAATACTGGTATGTTGGAAG

CAAAGATACAGAACTTATTCAGTTCACAAAGTTATTACAAATGTTCAGAAAAATAAATTA

TTACACTCATTAGGGAAAAAGCAATAAATAATAAATCACAGTCTATTTTGAGCCAGAGGG

CAAGTTGTTTTTTTATGGCCGTGTCGCTTACAATTTGAACATTTATTCCTTCTCTTCTTG

GATGTAAATGATACACCGATGGATGGAATGTGTTGAAATCTTTTCCTGCCTCTTTTGATA

AATGTCTTACATGTACATGTTCTTTTATTTCCCACGAAGGCACTACGAATTTGGCTCGTA

ACCTTCTCACCATTCTTGTACAAGAGTGATTTGCAATACCATCTTCAATAATATTGCTGG

ACTCAACCATTTGATCATCAGCTGCTATGGGAGATGCCGCAACAGCATTTGAAGCTGCGG

CGGACCTCTCGACCCGCGACATATAAAGAATAGGCCTACTACCATTACCATCTAGATAAA

TCTCTACCTACTACCATTACCATCATCAGCTACTTCGTTCATTCCTAATGAACGAAGGAG

CTATTTTCCCCTAGCAAGTACAACACTGAGCATATAACTAATGGATACGTCGCCGCGATC

ACAAGTTAATTATCCGCGTTCAATGACGCTGTCAATCAACTTGTCAAATGTACCCTCCAA

TTTCAATGCAATAGGTACTGTTGAACTAGTATTGGAACGCCAAGACCAACACTTAGGCGT

CTCTACCTATTGCCCATAGTAATCAGCACCCAGTACTACATATCACTCCATAATAGCTCT

ATTTACTGATATATGAACAAGTTAATAATATACTGAAATAGGTCGATGAACAAAAACAAA

GTTCTAATGGAACTGAAGCATAGTTGTTATAATGGAACAAAAACAATAAAGGATATGATA

AAGCAGCTTACCAGCTCAATAAAGCACTTCGTATAGTACGATGAAGTTGATATCAACTCA

ATTCGAAGTAATTGCACCCACCACGATAGACGGATTAGATTCTTTTGAAATTGAAATTTT

AAAAGCTTCCCACAACCATAAATCAAGAAATGAGAGCAAACTGAAGATGGGAATTGGATT

TCTGATAGCTATGGAACAAAATGGTTGGATTTGAGCCCTAAAACGAGGTTCAACTGAAGA

AGAGGACAATGGAGGCGGCTGGAACGAGGGGGGGAGGGAGAGGGGGAATAGGGCGGCAAA

TTTTGGAGGAGGGGTAGGAAATTTTCCTTTTTTGGTAATGGGCCAAAGTGTAATATTTAG

AACTTGGGGGTTGGGACGTGTAGGTATTTTTTTGGTGTAATTAAATCCTAATCCTGATTA

AAAAAGAAGAGGTCATCTAGCCAATTTTTAAAACTTGGAGGTTATTTTTTTAATTGAAGA

CTTGGGGGAGGTCAAGGAGCAAAAAGCCCCTACTTTCTTTGCCCCCTCTTTTGCGGTTCT

TTTTTTGGTGAGAATTAGGGGTTGCTTGATCAACATGCTCTATGTTGTTATTTTGTTGTA

CGATTATATTTGGGTCAAAGTTTTAGTTTTTTCTATGTGCAGGTGTGCACCAATTTAAGT

TGATAGGCAGAAACCTATAGTTCTTACCCTTTATTATAGTTTACATTTATCCAAGGCACG

TTGGCTTTACTTGGTTTCTCCGCAATGCATTATGAATACTGATGCTAATGTGCTTGATGG

AATGCTGAAGAAACTAACGATTTTATTGTAGTTTTCCCAATAAGCTTACATTACTTACTG

TCACTGGATAAAAAATTATTGAACAATGGAATATGCATTTTTGGTTAAATAAATATTCGT

TTGAAAGTATTCTCTACAATCTTCCATTTAGAAAATTTTGCTACTTTTCTCACTTACATC

CACAGGTTCTTTTGGGAGATCTTGGTGAAAGGGACATTTGATGAATGAACAAATGATTGT

GATTCCATAAAGAAAGAGTAAGCTTTGGATTTCCAAATGGATTCTGTCAAGGTTTATCCA

ATAAAACTACAGTCTCAAATCAGTTTCTGAATGAATTGTTATGCTATTAGATTGGCGAAG

TTTTGTATTTTTCATTGCTATGAGAATAAACAGCTCTTATGAGGTTTGGACACTCAAATT

TTGACCTGCGAACAGGTGATTACAATCAGCTTAATGTAATGTTTAGGTGGAGGACTTGAC

GAAGAACTATTGTGAGACGGAAAAAAAATACCAAAGGACTCCCATCTTTTTCAAAATGAG

CATCGGCTATTTTTCCTTTGAAATAGATAGGTTTTATCTTACAGTGTATTGCAATAGATA

TTTTGCTTATTCAATAGCAATGCTTTATGGAAAATGCTAACAGGGAGAACCTCAGTATCT

ATATCAGTAGATAAGAACATTGTCAAACATGCAACCATCCATACCTTTGAAATGCAGAAT

ATAAATGAGCATGTCAGCTTCATTTACAGAAAACGAAATCTTATCTCAATTTAGTGTTGG

GTCCTGCTCAGCACGGGCTTCTATGAACACGGCTGTACATAATTGTACATAAACCTACGC

CAAGTGCACTAAAGTTTGTCCTTATCTCATTTTCCACTTTTTGCCTCTTAAACACGTGTA

CTGTCACTTTCTACATTTGTTTGCC

>XLOC_032596 transcript=TCONS_00053569

TTCATACTTCCGCTACTTTACCTCATATTCTTCCTACTTTTCCTCCATCTTCATATCCTC

CTGGTTTTTTCACTACACCCCCTATGAATTACCCTGGCATGCATTCTATGTATGGATCTG

ACCCTCCCTCTCAGTTTATTCCTACGCTTGGCATGCCACTTTCTTCCCCGAGTTATGTTG

TTCGTCCGCATGGTATGAGCTCATCCATGCCTTCGCCTTCTTCCAATCCATCACCATCCC

CATCATCATCTACTCCTATTGCATCTGGATCGCAGTTTGTTGACCCTACATCATCCCCGT

CTGTTGGTGCTTCTGAACCACTTAGTGATCAGAGTATGTACTACATTGGTGAGCTTGGCA

CATTCGATCCTCAAGGGAGGTTAAGGATTGCTCCTGAGGGAGATAACGGTTTTTATCCCT

CAGACAAGGCTACACACTTGGTTACAGAATGCATCAAACATATATATACAGAAGCATGGA

CATCCTGGGGTAAGGTACCCTCGGATAGAAGGATTGCGATGTTTAATATGTTTAAGACAA

AGTGTGTGTGGCATCCATTCTACAAAGCTAAAATACAAAGCAATTTCCATGTCAAATGTG

GAGAGAGAGTCAAAGGTACCCTTTATGCTTGTCGGAGAAGTGGAAAGCAGCCCGATTGGA

TATACCCACAAAATTGGCAAAAACTATTGGATTACTGGGGAGGTGAAGAATTTCATAAGA

AGAGCGCCCAAGGAAAGGCAGCTCGAGCTTCTGAAAAGAGTGGATCGTTGCACACTGGGG

GTTCAGTTAGTGAGCCACTCAGAAGATGAGATTGGAAAAGAAAGAAGGGCGCCCTTTGAC

TTTTCCTGAGTTTTTTAAGAAGAGGCATATATTTACGGACAAAACCGGAGAAGTGTGGGT

CGAGGAGCGCGCTAAAAAAGCATATGAAGAATATCTAGCCCTTTATGAGGAGAGAATTGC

AAGTCAGGCATCCGAATGTGAAAGCAATGCCCCAACTCAGCCGTCATTTGAGGAGGAAAC

TTCATTATGGTTTGAGGTAACGGGTGGGGTAAACAAGGGATGTGCATATGGCCTTGGATC

ACAATTTTCCTCGGGTCGTGTGAAAGCAAGTATAACTAGTAATTACTCTTCTAGTTCTTC

AATTAATCATTCAGTCGAAGTCCAGTTGCGCAATGAAATAGAAGCGCTAAGGAAAGAGCG

TGAAGAAGATCGCAAACGTACAGAGGATAATATTAAAGCGATGAAAGAGATGCATGATAT

GATGATGAAAACGATGCAGGATATTTCAAAAAAAGAGAAAAGAAATGGCAAGAGCAAAGG

CAAGGGCAAGGGCAAGAGCAAGCGCCACTACAGCTCGTCGTCCGAGTCTGACTCCGAGGA

AACGGAGTAGTTTCGGATTGTTGATGTTTTAAGACTTGTGTTTTAGACTTGAAGTTAGTT

ATGTTAAATTTGTTCGATCGTTAGACTTGTGGTTGTTATGTTAGGC

>XLOC_011021 transcript=TCONS_00017983

AAAATTTAGCCACTTGGGTTTGCCTTGTAATTTGTAAAGATTGAATCTTTATAGTGAATA

GCCACTTGGGTTTGCCTTAGATTTTGTGAAGATTGAATCTTTATAGTGAATTTTTACTTG

GGTGTGTCTTAGATTTTATAAAGATTGAAACTTTATAGACATTTTTGATATTTTGATTGC

TGAAGTACTTAGGTTTGCTGTAAAGGTTGAAACTTTATAGGGTAATTTTGAGAAACTTTG

ATTATTGAAGTTGAACTAACTATTATTATTAATAGTAGATTATTAGGAGTTGTGTGAGGT

TTGTCGATGGCTCGGTTGGTTCTACCGTGCAAGGCTTCAAGTTTATCGAAGACATGTCTA

TTGGGTTTGATCTCCAATGCTCCTTTTCAGGCAAGTTTCGGTGT

>XLOC_033750 transcript=TCONS_00055531

TTTCCTTTTGTGGCCGAAAAGAGATCAAAATGCAAATCCTCCACATTTTTACTCTGAAAA

GCCTTTGCATCGCCGCTTATCCTTTTGTTCTAATCAAGACGATCTTGCTGAGTGGAGGCC

AACATGCTCACACTCTCGTTCAATATTATTCCGGGAGGTTAGTGATAATACATGTTCTTT

AATGGGAGACGCCTCTATCATCTTTGGGAAATTCAAATTTCTTAGGGTGTTGGATTTGGA

GTTTGTCATCGTCAAATCTTTTCCCACTGAACTTGATTATCTGAGGTACCTTGCTTTTCA

AACTACTGAGCATCAGATCCCATCAACTATAAAGAACCTTTGGAATCTTCAGACGTTCAT

TGTCAAAAAGGCTGAATACTCTGTAGAGTTGCCACATACTTTTTGGGAGCTGAAAAAGTT

GAGGCATGTAAGCATTGAGAACATGGCTTCGTGTGATTATTTGAGTGTTACTGAGAGCCC

CTCAAACTTGGAAAATTTGGCAGCACTTTCCACGATCTTTTTTCCGGGGCCAAACCATGT

GGAGATGATAGTGAGCAAAATACCCAACCTTCGAAAGTTAAGCTGTATATTCTCCAATCC

ATGGCCTTCGAAAGAGAGTGAAACTGATCGGTTTCCAATATTAGACTCATTATCTCAACT

TGAAAAGCTAAAACTGGATTTCAACAATATTCAGGTAGGTCCTTCAGGACTAAACTTTCC

GACAAGTCTGAGGGAACTGACATTATGCAGTTTTCCATTTACACTTGCTGGAGCTTCAAC

CATTGGAAACCTTCACAGCCTTGAGGTACTTAAACTGCAATGGGTTCGTTTTGAAGATGA

TGAATGGGAAGTGAGTGATAATGAGTTCCCTCAGCTCAAATTCTTGAAACTAGAAAATCT

CCGACTCATAGGATGGAAAGTGTCAGACGAAGCCTTTCGTTGCCTTGAGAAACTAGTCTT

GCATAGATGCTTCGATCTTGTAGAAATCCCTTCTTGCTTTGAGGAACTGGGTTGCTTGCA

ATACATCGAGCTAAAATCATGCGGCGAAGATGTTACCAACTTAGCCAAGGATACCAGAGA

AAAACGAGTTGAAAATGGGCACAGATGTGACATTGAAGTCGTTATCTAGCATTGGAACTC

TGAGCAATGTTTGGTCCTTGTTGTTCATTGTTCACCATTGGTTGTAGCAAGGTGCTGGTT

GGTCGCTGCAGTTATTGCCGATTTGTGTTACTTCGTACTTTCATTTCGTGACATACTTTT

GTAGCCTGTGGCTATGGTAGTCTTATGAGTTTTTTAAAATAAATTATTACTTTCATGGAA

AGTTGTTGATTTGTTTGAGATATTTTACAGCATTTTTTTTTCACCTTTTCAATATTTGGA

TTATCAGGAGGCTGTTGGTAATATGTAGCATCTCATATACTTTGTACTATGCATAGCCAT

ATTGGC

>XLOC_024187 transcript=TCONS_00039789

CAAATATTGTTTAGAAGATAATCCATCAGAATTAAGATAGAAACTTAAGATGCACAATTA

CATCGTAATACAACTTATGTCATCTTGAGTAGAAGCCTGAAGTCTAAAGAGAATTAAAGA

AATTTTACTGTCAAGCCATAGCATATTTGCTTTCTGAGATATGAAGGGGATCCATGTTAA

TTCTTTTAGAAGTTTTACCATGAAATATCAAGCAAAAGTTTAAAGGGACTGTCTTCCACA

TCTTCTATTTCTTGTTAGAAATAATTGAATAATCCTATGAAGTTCCCAGTGAACGTTAAA

ATGTCCACAGATAAAGAAACTTTGCCCCGAAAAATTACGGCAAGTAATAAAATCCACGAA

GAACGTAATAAAAATGATTTTTTGCTAATTACAATTTTTTGTAACAGTATCCCCTTTTTA

AAAAAAGATATTGGATTATGTTTTTATTTTTAATGCACTCAATAAAAGAAGTCAATTGAA

CTAATACTTGTGGTGACTTGCAAAATTGCATTGCAGACCAATTCCTAATAGGTTATACTA

TTCAAGCTATGCTGATAATGGTAAATCAATGGATCCTTTCTTACGTCTTATGCAGGAAAA

TCAAGATTTTCGAAATACTTAGAGGCAACAATTGATGAGCAGAACGAGCTCGAAGAGGAG

AAATGTTTGAGGAGAGTAGAACCTCACGTCTCTAATAATAAATAGGACATTCTCTCATAA

TGAATTGTTCCATATTTCTCTCTGTTCTTTTTCTTTTTCTTTTTCTTTTCTCATAGACGG

TTTTTAATTAGATTTGATATCACACTAATGATATTAGTTAAGCTTAAATTAAATAATATA

TATAGCCTATGAGGATTATATAGCGGGCATGAACTTGTTAGGAACTGGG

>XLOC_012902 transcript=TCONS_00021121

TGTTGGGCTACATTTACAATATTGGGCCACAAAAAGAAGTTCTACCACAATCGCTAACCC

CATATATAACCATTTCCCATCTCAAAACCCTAGCAGTCACAAAGCAGCAGCAAAGGTACA

AATTTGCACTTCAGCAAAAGTAACAGTAATCAGATTGCTAGTAAAATGTCGCATGGAGAA

ATTGCTTGTACCTACGCTTGTTTAATCCTCAACGATGAGGAAATCCCAATTACCGCAGAG

AAAATATCTGCTTTAGCAAAAGCAGCAAATGTGACAGTAGAGCCATACTGGCCTCTGTTG

TTTGCCAGGCTAGCTGAGAAAAGAAACCTAAGTGATCTCATTATGAATGTTGGTGCTGGT

GGTGGCGGTGCTGCTGTCGCTGTTGCTGCCCCTGCTGGTGGGGCTGCACCTGGTGCTGCT

GCAGCTGCTCCTGCTGCTGAGGAAAAGAAGGAAGAGCCAAAGGAAGAAAGTGACGATGAT

ATGGGATTCAGTCTGTTCGACTAGGTGCTCCTTTATCAGCTTGTTTTTTGAGAAAATTGA

TGACTCTAGTAGCAATTAGCATTTTTAGTTATGAAAGTCTTTGTAATTTATGAGGCTTAT

ACTCATGTTGTGCTAGTTATATTCTTCGACTTGCCGTATTTTGAGCCATTTCTGTTCTTT

TCATTATCAA

>XLOC_026832 transcript=TCONS_00044168

GTTTGCCTTAAGCTCTTTTTTTTTCCCTTATGGGTTAAGAACCTGATTGTTGTTTATGTG

TGGGGATTCTGATATTTATTACTCCCTGTGTTCCATTTTATGTAGTACTATTTCCTTATT

ATTATGCTCCAAAAAGAATGACACATTCCTATATGCAAACACTATAGCTTAAACTTTACA

TTTAGTCTTATGCTTTAAAGACCGCAAGTTTCAAGTCTCTCTTTCTTTTGTTCTTATGTC

CAAATTCTGCCACATAATTGGAGAAAGTACTAATTTCGTACTCCTTTTAAGAGTGCACTG

ACACTGATTTGGTATGAGCATGTAGCTTCTTTGGTGAATAAAGTGTAATTGAGTATAGCA

TTGATCGTGATGGTTAATTCAAGCAGTTCAGTATTGAAATTCCATTTGCATTTTGGGTAC

TACAGGCAATTGGATCAACTGGTGCTCAGTTCCTAGATCAGATACTAACAATCATGCTTC

CGAGGTTTTATGCCACAGTAACTTTTTAAACCTGGCTGGATGTAAACGAACCAAGGTCTC

ACTAAGATGCGAGAAGTATCATAAAGGTAATCATGACCGCGATGGTGAAATATCTACACA

TCTTAAACATAAAGTTCATTAGGATCCTGCTGCTAAATACTAATTTTCAAGTTTCTTACA

TAGACGTGCCTGTAAGTCTGTTTTCTCCACGGCCTCTAAATTTTGCTTTTCCATATGCCT

GAGCATCTAGACTTAACCCTATGCTATTGTGTTAAGGCAGATATCCCTATAAGTTTGTTG

ACCTCAGTTCTTTGGTAGTTGGTGATTGAATTAGTCTTAATTTTTATATATCTTAGCTAA

GAATGGTACAGGAATATTCACCTGCTATAAATCAACTACGCCGTTCGCCCTGATCTTAGG

CCCTGATTACCCTCCATGGACAAACCTTGTGGAGGAACCCATAGGTTTTCCGGGCATTGG

ATTCTTATCAATGTTTGCATTACTGAAGCTGACATTCTTGCTTTCGCTTTGTCCATCACT

GCTTGTACTGAGGCTTCTCTCTAGCGGAATGCTCCCCTACCGATATATTTTTATATCCCA

CAGTTTCAGCGGATCGCTTTGCCCCGTTGATTTTCAGAGCAAGAGCATTCGATCAGTGAG

CTATACACACTTCTTCAAGGGTGGCTGCTTCTAGGTAAACCTCCTAGCTGTCCCTGCACC

CGAGATTCTGTAGGTGAAGAATGTCCTCTGAGATGGCTACCAGGATGTCCTTAAACATTC

ATTAAACGTTTCTGACTTGTGTGTATAATTGTGTTTGTTCAACCTGCCTTGTCTTCCCTG

CACACACCCAAGACTCTATCATACAAGATGCCTTTTTAGTTCTGACTAGTTATGTGAAGG

ATCT

>XLOC_009472 transcript=TCONS_00015407

CTTAGAAAATGTACAGAGAGGCCAAACAAAACCTTTCTTGGTCCTTTCCTTATCGAACAG

TCCTGAGATTTCCTCAATTGTTATTTACTTATCTGGGACTAATTGTACTACACGAGCAAG

CTGGAATTCAGGAAAATAGAACTCCAATTGTCAATTGAACCTAGTTGGATATACTACACT

TGTTGGAGCTATGTTTAGACTCGAGGAGCAATCGAGATTGAACCTAGTTGGATGTACTAC

ACTCGTTGGAGCTATGTTTAGACTCGAGGAGCAATCGAGGGTTAAGACAGGATAGATAAG

TACAGGAGGTTTGCTCGGTAAAGCTTAGGTACCGGGTGTCAGTCACAGCTCGTTCAGAAA

ACGGATCGTCACAGTAGCCATGGTGGTGATTCCATGGCGACACATCAGCATAAGTAGCAT

CGGCAGTGACGATGGTAATGCGATGAGGAATGTGGTGAGTTTTTTAGAACTATAGTGGAG

GTGATTGGTGGCGTAGAGGCCAGTTTGGTGGCAGCGCATTGCAGGCAGCGGGCAGCAGTG

GCGATGCAATGCACATCAAGGCTATGTTCTTGATGTATTGGTGAGACAAAAATTCAATCT

TTTTTTCTACTTAATTTGGATCATGACTAGTTGGTAAAAGTAGTGTCGTTTAGGATGCTT

ATTTACATAAATATGTTTGGTTCAATAAAAGAAATATTCATATCTGTTTTTACTTACAAT

TTCAATCACTAATGTTGGTGTTTGGTTTGAAGTGTGCAAATGGTATATGTATGTGTATGT

ACAAGTTCATATGCTTCTAAATTGAAACTGTTCGTAGGAACTAACTAGTGTAGTGTAAAC

TAGAAGCATTGTATCTTGTCAAGCCATTGAACTATAAAGATTGTAAGAAGTCGCATTTTG

TCGTTC

>XLOC_021359 transcript=TCONS_00035126
[truncated: 1,909,766 more chars]
