# Supplementary material for: Transcriptome Profiling Reveals a Petunia Transcription Factor, PhCOL4, Contributing to Antiviral RNA Silencing
Source: Front Plant Sci. 2022 Apr 14;13:876428. doi: 10.3389/fpls.2022.876428 (PMC9047179; doi:10.3389/fpls.2022.876428)
Supplement: Supplementary file 12 [file Table_12.docx]

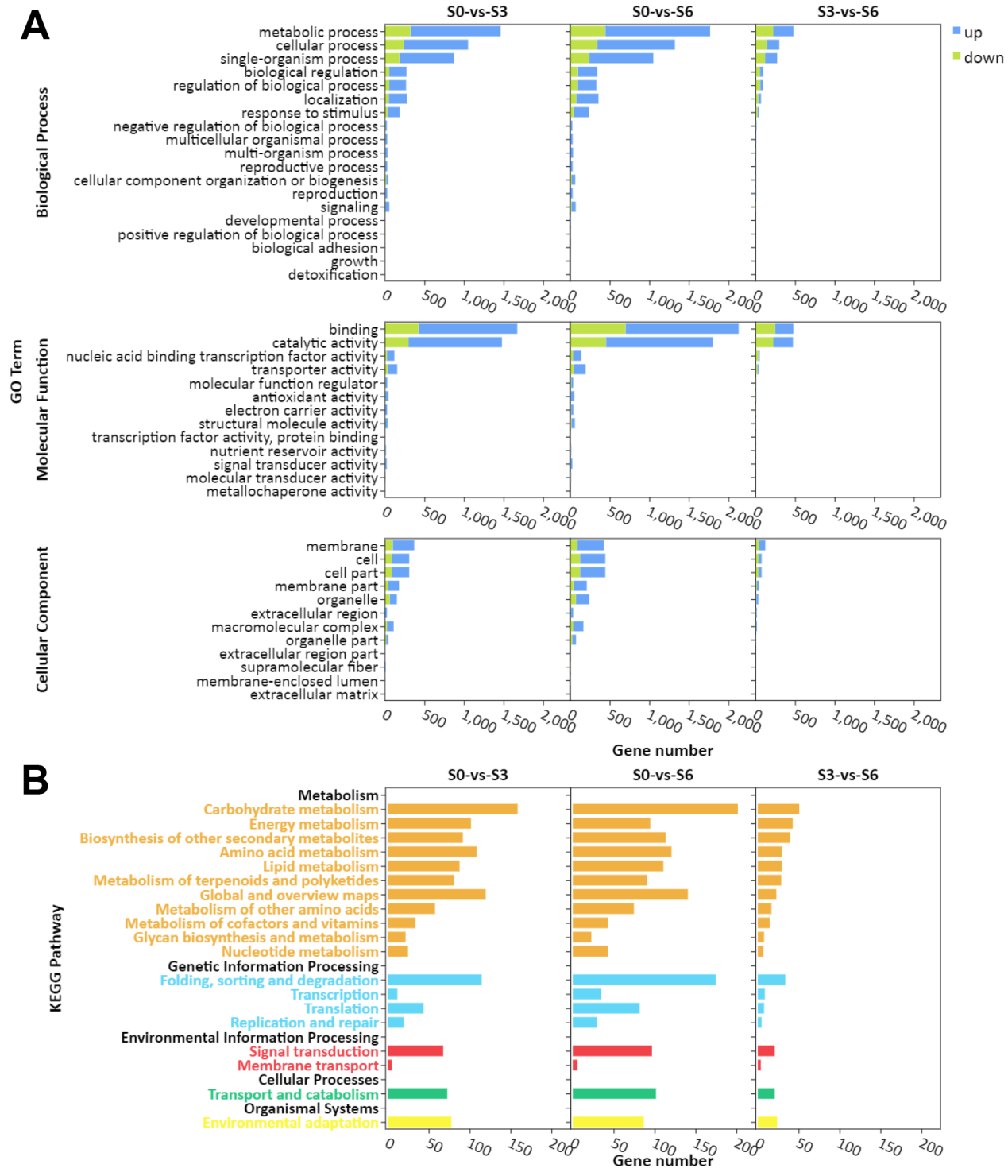


**Supplementary Figure S3** GO and KEGG pathway enrichments of differentially expressed genes. (A) Go terms as biological process, molecular function, and cellular component of differentially expressed unigenes in petunia leaves infected with TRV (PPK20). (B) KEGG pathways of transcripts with differential expression in TRV (PPK20)-infected leaves.
