# Supplementary material for: Transcriptome Profiling Reveals a Petunia Transcription Factor, PhCOL4, Contributing to Antiviral RNA Silencing
Source: Front Plant Sci. 2022 Apr 14;13:876428. doi: 10.3389/fpls.2022.876428 (PMC9047179; doi:10.3389/fpls.2022.876428)
Supplement: Supplementary file 13 [file Table_13.docx]

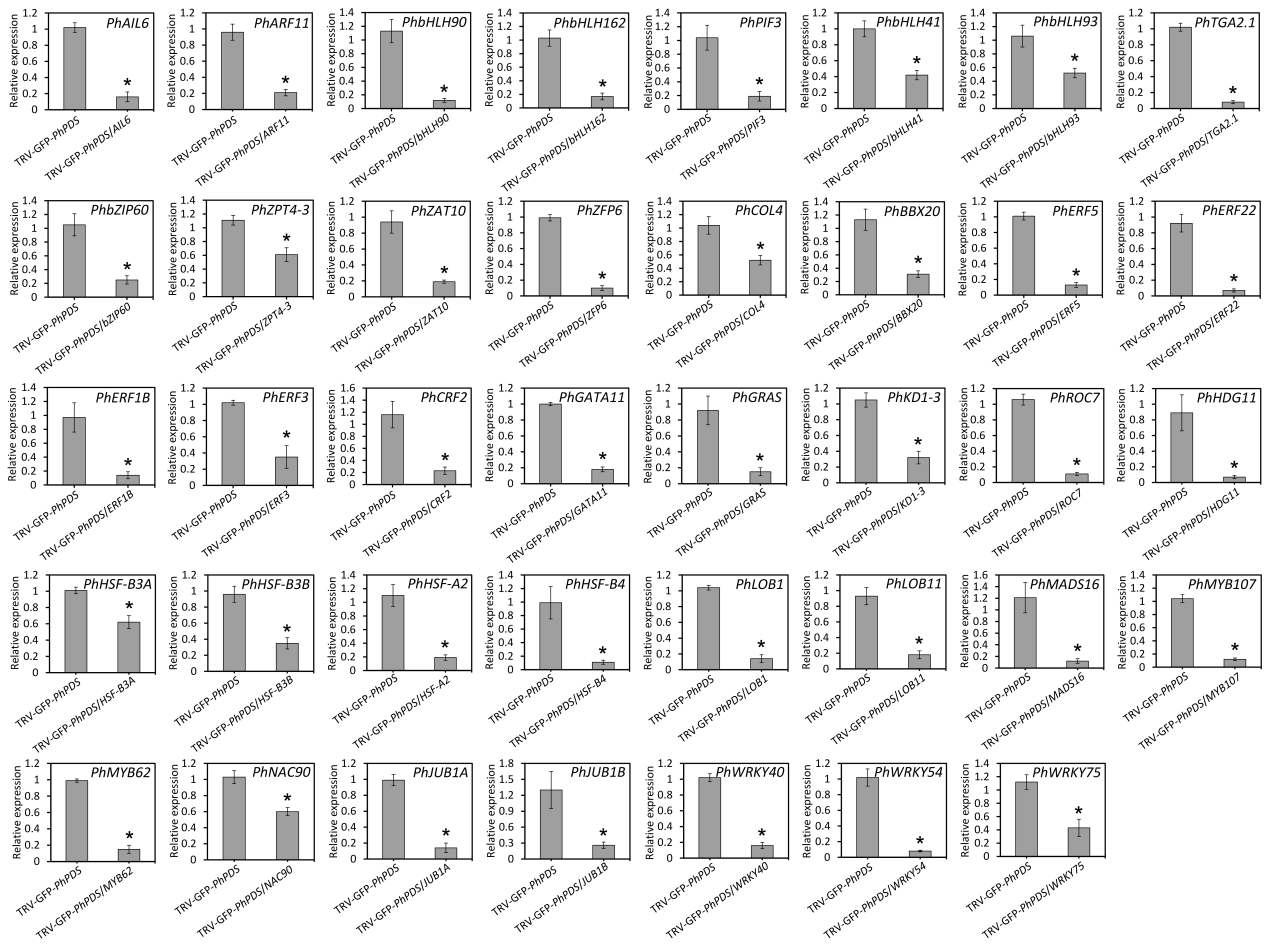


**Supplementary Figure S4** Expression of transcription factors in petunia leaves upon exposure to VIGS. RT-qPCR analysis of transcript levels of 39 transcription factors (TFs) in uppermost systemically-infected leaves at 14 days post inoculation with various TRV-GFP-*PhPDS* constructs. The TRV-GFP-*PhPDS* construct without TF insert was used for comparison. Transcript abundances were normalized to *26S rRNA*. Error bars represent standard error of the mean from three biological replicates. Significance of difference was verified using Student’s *t*-test (*P* < 0.05) as indicated by asterisks.
